# Bromophenyl-Diphenylphosphine Oxides as Novel Starting Materials in the Suzuki–Miyaura and the Hirao Cross-Couplings, as well as in the Michaelis–Arbuzov Reaction

**DOI:** 10.1021/acsomega.6c04273

**Published:** 2026-07-04

**Authors:** Bianka Huszár, Dalma Gál, Zsolt Kelemen, László Drahos, György Keglevich

**Affiliations:** † Department of Organic Chemistry and Technology, Faculty of Chemical Technology and Biotechnology, 61810 Budapest University of Technology and Economics, Műegyetem rkp. 3, 1111 Budapest, Hungary; ‡ Department of Inorganic and Analytical Chemistry, Faculty of Chemical Technology and Biotechnology, Budapest University of Technology and Economics, Műegyetem rkp. 3, 1111 Budapest, Hungary; § MS Proteomics Research Group, 280964 HUN-REN Research Centre for Natural Sciences, 1117 Budapest, Hungary

## Abstract

4- and 3-Bromophenyl-diphenylphosphine oxides prepared by us in a selective P–C coupling reaction offered themselves as excellent new starting materials in the Suzuki–Miyaura C–C and in the Hirao P–C cross-coupling reactions, as well as in the Michaelis–Arbuzov reaction. Novel *bis*(>P­(O))-functionalized aromatics are the valuable products of the two latter phosphinoylations. The three transformations outlined were performed by applying metal catalysts (mainly Pd, but Ni may also be suitable) under microwave irradiation. Twenty-three, mostly new, P-functionalized aromatics were made available and fully characterized. On the other hand, the effect of the substituents in the aromatic ring on the reactivity, as well as the mechanism and energetics of the reactions, was studied by density functional theory calculations at the B3LYP/6-31G­(d,p) level of theory. As regards the C–C cross-coupling, the 4- and 3-Ph_2_P­(O)-substitution had only a minor effect on the value of the activation energy. In the transition metal-catalyzed Arbuzov reaction, the initial oxidative addition step shows comparable activation barriers for all investigated systems. However, in the subsequent steps, including the elimination of MeBr and the reductive elimination step, both the intermediates and transition states exhibit much larger differences in relative Gibbs free energy, which probably causes the observed differences in reactivity of the 4- and 3-Ph_2_P­(O)-substituted models, as compared to the unsubstituted case. The elimination of MeBr has the highest Gibbs free energy requirement during the process. This high energy barrier may be overcome by the beneficial effects of microwave irradiation. In the Hirao cross-coupling, the 4-Ph_2_P­(O)-substitution resulted in a decrease in the barrier of the oxidative addition step, whereas both the 3-Ph_2_P­(O)-substitution and the electron-sending 4-Me- and 4-MeO-substituents led to a slight increase in the barrier. The theoretical data were in accord with the experimental findings.

## Graphical abstract

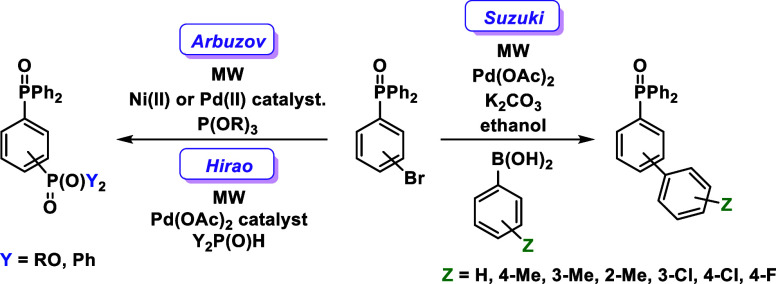

## 1 Introduction

Bromoarenes may be versatile starting materials or intermediates in organic chemical syntheses. A typical transformation is the C–C cross-coupling with arylboronic acids that is called the Suzuki–Miyaura reaction, giving rise to asymmetric biaryl products.^1–5^ Bromoarenes are important agents also toward P-functionalized arenes. On the one hand, their Michaelis–Arbuzov reaction with trialkyl phosphites may lead to arylphosphonates. However, the decreased reactivity of bromoarenes requires the application of a Ni-salt catalyst.^6,7^ On the other hand, the Hirao P–C cross-coupling reaction with >P­(O)H reagents, such as dialkyl phosphites, alkyl phenyl-*H*-phosphinates, and secondary phosphine oxides, is a more general approach to synthesize dialkyl arylphosphonates, alkyl aryl-phenylphosphinates, and tertiary phosphine oxides, respectively.^8–10^ The Keglevich group developed a generally applicable microwave (MW)-assisted P–C coupling methodology, where the trivalent tautomeric form (>P–OH) of the >P­(O)H reagent served as the P-ligand to Pd­(OAc)_2._ ^11^ The mechanism was evaluated by theoretical calculations.^12^ Newer results include decarbonylative Hirao reactions of carboxylic acid derivatives, such as anhydrides and amides.^13,14^

In one of our earlier projects, 4- and 3-P-functionalized bromoarenes were prepared in a selective manner.^15^ In this work, we wished to utilize the 4- and 3-P­(O)<-bromoarenes as new reagents in the Suzuki–Miyaura and Hirao cross-coupling reactions, as well as in the Michaelis–Arbuzov reaction. The use of MW assistance was attractive to develop selective transformations. It was another purpose to evaluate theoretically the mechanism, and how the nature of the substituents affects the reactivity and the mechanism.

## 2 Results and Discussion

### 2.1 Syntheses

#### 2.1.1 Application of the Bromophenyl-Diphenylphosphine Oxides in the Suzuki–Miyaura Coupling Reaction

Our new model reactions, the coupling of 4- and 3-bromophenyl-diphenylphosphine oxides (**2A** and **2B**) with phenylboronic acid (**1a**), were performed in the presence of 10% of Pd­(OAc)_2_ and 1.1 equiv of potassium carbonate in ethanol at 120 °C under MW irradiation. The corresponding products were obtained in 74% and 77% yields, respectively, after purification by column chromatography (Scheme 1).

**Scheme 1:**
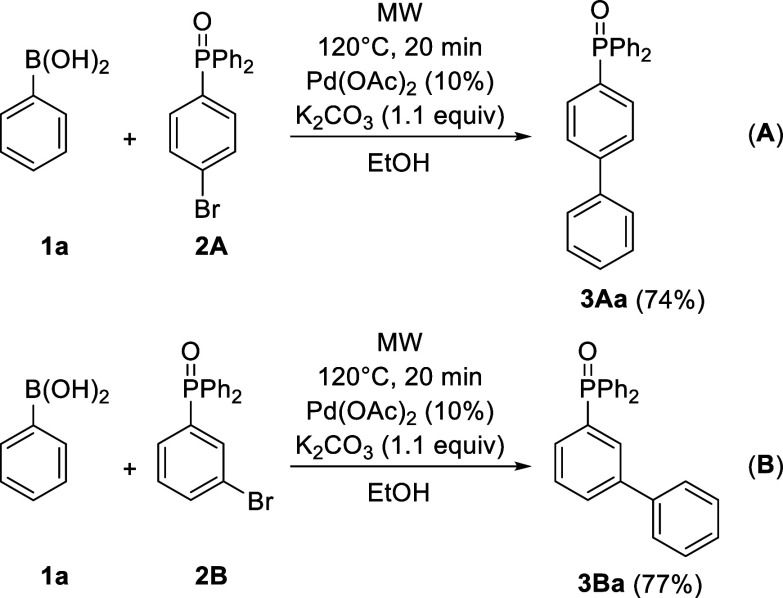
Coupling Reaction of 4- and 3-Bromophenyl-diphenylphosphine Oxides (**2A** and **2B**) with Phenylboronic Acid (**1a**)

Both transformations represent a new option for the preparation of products **3Aa** and **3Ba**. Earlier syntheses involved oxidative couplings^16,17^ or a C–C coupling of chlorophenyl-diphenylphosphine oxide with phenylboronic acid.^18^ The new approach developed offers a fast and efficient method for the preparation of biphenyl-diphenylphosphine oxides **3Aa** and **3Ba**.

Then, as an extension of the new protocol, phosphine oxides **2A** and **2B** were reacted with a series of substituted arylboronic acids (**3**) under similar conditions (Table 1). As regards the coupling of 4-bromophenyl-diphenylphosphine oxide (**2A**) with arylboronic acids with 2-Me, 3-Me, 4-Me, or 4-F substituents in the aromatic ring (**1b**–**d**, **1g**), these reactions were performed as described above, applying a 120 °C/20 min set of conditions (Table 1, entries 1–4 and 7). However, the reaction of the 3-Cl- and 4-Cl-phenylboronic acids (**1e** and **1f**) with bromoarene **2A** required somewhat harsher conditions involving an irradiation of 135 °C for 60 min (Table 1, entries 5 and 6). The situation was similar for the coupling of 3-bromophenyl-diphenylphosphine oxide (**2B**) with arylboronic acids **1d**–**g** (Table 1, entries 9–12); however, the reaction of the 3-Cl- and 4-Cl-phenylboronic acids (**1e** and **1f**) needed an even longer reaction time of 90 min at 135 °C. It is noted that in almost all cases, on average, 12% of triphenylphosphine oxide was also present in the crude mixtures. The biarylphosphine oxides (**3Aa**–**g** and **3Ba,d**–**g**) were obtained in 64–83% yields after purification by column chromatography. The structures of the products were identified by ^31^P, ^13^C and ^1^H NMR, as well as HRMS. From among the 14 biaryl-diphenylphosphine oxides prepared, nine were new products. Three compounds were synthesized by another approach,^16,17^ while only two species were made available by the Suzuki reaction, but starting from chloroarenes.^18^

**Table 1:**
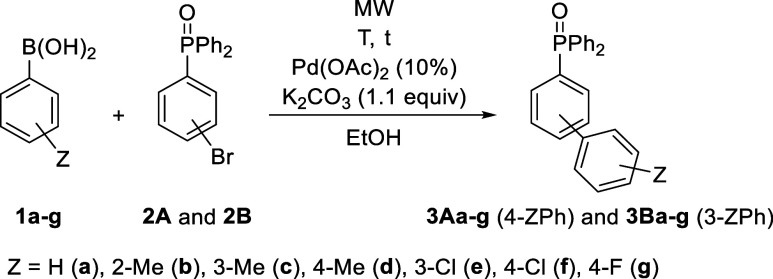
Suzuki Coupling Reaction of 4- and 3-Bromophenyl-Diphenylphosphine Oxides (**2A** and **2B**) with Different Arylboronic Acids (**1a–g**)

						product^a^ (%)		
entry	P-reagent	*Z*	*T* (°C)	*t* (min)	conversion (%)^a^	**3A** or **3B**	Ph_3_PO^b^	yield (%)
1	**2A**	4-Ph	120	20	100	94	6	74 (**3Aa**)
2		2-MePh	120	20	100	100	–	76 (**3Ab**)
3		3-MePh	120	20	100	88	12	69 (**3Ac**)
4		4-MePh	120	20	100	94	6	72 (**3Ad**)
5		3-ClPh	135	60	100	88	12	69 (**3Ae**)
6		4-ClPh	135	60	100	90	10	71 (**3Af**)
7		4-FPh	120	20	100	94	6	73 (**3Ag**)
8	**2B**	3-Ph	120	20	90	85	6	77 (**3Ba**)
9		4-MePh	120	20	100	86	14	78 (**3Bd**)
10		3-ClPh	135	90	100	72	28	64 (**3Be**)
11		4-ClPh	135	90	100	84	16	69 (**3Bf**)
12		4-FPh	120	20	100	100	–	83 (**3Bg**)

a Based on ^31^P NMR.

b δ_P_ (CDCl_3_, 121.5 MHz) 29.2,: δ_P_ lit.^[^ ^19^ ^]^ (CDCl_3_, 162 MHz) 29.5; [M + H]^+^ = 279.0941, C_18_H_16_OP requires 279.0939.

#### 2.1.2 Application of the Bromophenyl-Diphenylphosphine Oxides in the Michaelis–Arbuzov Reaction

Then, as another new model reaction, the bromophenyl-diphenylphosphine oxides (**2A** and **2B**) were taken in a Michaelis–Arbuzov reaction with trialkyl phosphite. It is known that in general, the Michaelis–Arbuzov reaction of bromoarenes needs to be catalyzed. A typical catalyst may be NiCl_2_.^6^ Applying bromoarene **2A**, 1.2 equiv of triethyl phosphite, and 10% of NiCl_2_ at 160 °C for 4 h under MW irradiation, the conversion was only 70% (Table 2, entry 1). An increase in the quantity of the catalyst to 20% and then an elevated temperature to 180 °C were harmful, as the conversion was 90%, but the proportion of the triphenylphosphine oxide byproduct increased to 61% and 58%, respectively (Table 2, entries 2 and 3, respectively). The problem could be solved by the application of 10% of Pd­(OAc)_2_ as a novel catalyst in the Arbuzov reaction. In this case, under MW irradiation at 160 °C, the conversion was complete after 2 h, and the desired phosphine oxide-phosphonate (**4Aa**) was formed selectively (Table 2, entry 4). The optimum conditions found could be applied successfully also for the Michaelis–Arbuzov reaction of aryl bromide **2A** with trimethyl phosphite (Table 2, entry 5). Changing for the reaction of 3-bromophenyl-diphenylphosphine oxide (**2B**) and triethyl phosphite, NiCl_2_ did not promote the Arbuzov fission at all (Table 2, entry 6), and Pd­(OAc)_2_ was less efficient as in the above cases, since the formation of the triphenylphosphine oxide byproduct was inevitable (Table 2, entries 7 and 8). At 160 °C/4 h, the conversion was almost quantitative (95%), covering 59% of the expected product (**4Ba**) and 36% of the byproduct. With trimethyl phosphite as the P-reagent, there was need for a temperature of 180 °C. The 95% conversion obtained after a 1 h irradiation covered 82% of the expected product **4Bb** and 13% of the byproduct (Table 2, entry 9). One can see that the 3-Ph_2_P­(O)-bromobenzene (**2B**) is less reactive in the Michaelis–Arbuzov reaction than the 4-Ph_2_P­(O) counterpart (**2A**). On the other hand, the Ph_2_P­(O)-substitution decreases the reactivity as compared to the unsubstituted model. This is supported by our earlier experiences,^6^ where NiCl_2_ was a suitable catalyst. In the present case with Ph_2_P­(O)-substitution, there was need for the more active Pd­(OAc)_2_ catalyst. The most successful experiments marked by entries 4, 5, 8, and 9 resulted in the formation of phosphine-oxide-phosphonates **4Aa**, **4Ab**, **4Ba**, and **4Bb** after purification by column chromatography in yields of 75%, 71%, 49%, and 69%, respectively. The products (**4Aa**, **4Ab**, **4Ba**, and **4Bb**) were characterized by ^31^P, ^13^C and ^1^H NMR, as well as HRMS. The ^31^P NMR spectra exhibited two signals characteristic of the phosphine oxide moiety (δ_P_ ∼ 28–29) and the phosphonate function (δ_P_ ∼ 17–20). From among the four new bis­(P=O-functionalized)­arenes prepared, two were also synthesized by us via the Hirao P–C cross-coupling reaction (see Subchapter [Sec sec2.1.3]).

**Table 2:**
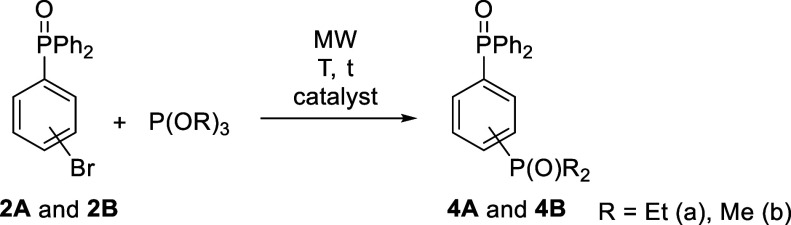
Michaelis–Arbuzov Reaction of 4- and 3-Bromophenyl-Diphenylphosphine Oxides (**2A** and **2B**) with Trialkyl Phosphites

		(RO)_3_P						products (%)		
entry	BrPhP(O)Ph_2_	*R*	equiv	catalyst	*T* (°C)	*t* (h)	conversion^a^ (%)	**4A** or **4B**	Ph_3_PO^b^	yield (%)
1	**2A**	Et	1.2	NiCl_2_ (10%)	160	4	70	64	6	51 (**4Aa**)
2		Et	1.2	NiCl_2_ (20%)	160	4	90	29	61	–
3		Et	1.2	NiCl_2_ (20%)	180	2	90	32	58	–
4		Et	1.5	Pd(OAc)_2_ (10%)	160	2	100	100	–	75 (**4Aa**)
5		Me	1.5	Pd(OAc)_2_ (10%)	160	2	90	88	2	71 (**4Ab**)
6	**2B**	Et	1.2	NiCl_2_ (10%)	160	2	–	–	–	–
7			1.5	Pd(OAc)_2_ (10%)	160	2	64	23	41	29 (**4Ba**)
8			1.5	Pd(OAc)_2_ (10%)	160	4	95	59	36	49 (**4Ba**)
9		Me	1.5	Pd(OAc)_2_ (10%)	180	1	95	82	13	69 (**4Bb**)

a Based on the relative ^31^P NMR intensities.

b δ_P_ (CDCl_3_, 121.5 MHz) 29,2,: δ_P_ lit.^19^ (CDCl_3_, 162 MHz) 29.5; [M + H]^+^ = 279.0941, C_18_H_16_OP requires 279.0939.

#### 2.1.3 Application of the Bromophenyl-Diphenylphosphine Oxides in the Hirao Reaction

Finally, the bromophenyl-diphenylphosphine oxides (**2A** and **2B**) were taken into a new type of MW-assisted Hirao reaction with different >P­(O)H reagents, applying 10% of Pd­(OAc)_2_ as the catalyst, triethylamine as the base, and ethyl alcohol as the solvent. The temperature was in all cases set to 150 °C. Our earlier studies confirmed the suitability of MW assistance in P–C coupling reactions to overcome the relatively high barriers.^12^ The beneficial effect is the consequence of the statistically occurring local overheatings in the bulk of the mixture.^20^ Coupling 4-bromophenyl-diphenylphosphine oxide (**2A**) with diethyl phosphite and dibutyl phosphite with an irradiation time of 30 and 60 min, respectively, the conversion was 95% and 75%, respectively, covering 76% of product **4Aa** and 60% of species **4Ac**, respectively (Table 3, entries 1 and 2). Ethyl phenyl-*H*-phosphinate revealed a somewhat lower reactivity, allowing a conversion of only 70% covering phosphine oxide—phosphinate **4Ad** in a proportion of 55% (Table 3, entry 3). As regards the Hirao reaction of 3-bromophenyl-diphenylphosphine oxide (**2B**), the results were similar (Table 3, entries 4 and 5). In this case, diphenylphosphine oxide was also coupled with reagent **2B** under similar conditions (Table 3, entry 6). The P–C couplings were in all cases accompanied by debromination of the reagent leading to triphenylphosphine oxide up to 22%. The products with two P-functions (**4Aa**, **4Ac**, **4Ad**, **4Ba**, **4Bc**, and **4Be**) were obtained in yields of 46–67% after purification by column chromatography and were characterized by ^31^P, ^13^C and ^1^H NMR, as well as HRMS spectral data. Of the six products, four are new, and two have also been prepared by the Michaelis–Arbuzov reaction by us (see Subchapter [Sec sec2.1.2]).

**Table 3:**
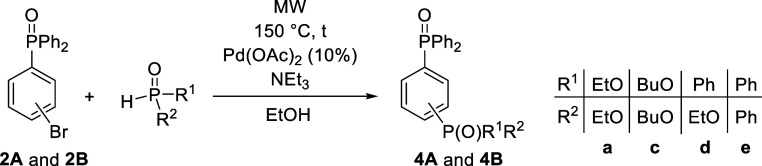
Hirao Reaction of 4- and 3-Bromophenyl-Diphenylphosphine Oxides (**2A** and **2B**) with Different >-P­(O)­H Reagents

						products (%)		
entry	BrPhP(O)Ph_2_	*R* ^1^	*R* ^2^	*t* (min)	conversion^a^ (%)	**4A** or **4B**	Ph_3_P(O)^b^	yield (%)
1	**2A**	EtO	EtO	30	95	76	19	51 (**4Aa**)
2		BuO	BuO^c^	60	75	60	15	49 (**4Ac**)
3		EtO	Ph	30	70	55	15	46 (**4Ad**)
4	**2B**	EtO	EtO	30	100	87	13	64 (**4Ba**)
5		BuO	BuO^c^	60	70	48	22	47 (**4Bc**)
6		Ph	Ph	30	94	85	9	67 (**4Be**)

a Based on ^31^P NMR.

b δ_P_ (CDCl_3_, 121.5 MHz) 29,2,: δ_P_ lit.^19^ (CDCl_3_, 162 MHz) 29.5; [M + H]^+^ = 279.0941, C_18_H_16_OP requires 279.0939.

c There was no transesterification side-reaction.

### 2.2 Theoretical Calculations

In order to better understand the catalytic reactions and the effect of the substituents, we performed DFT calculations. (Computational Details can be found in Subchapter 4.5, while the Cartesian coordinates and total energies of the optimized structures are listed in the Supporting Information.)

#### 2.2.1 A Mechanistic Study on the Suzuki–Miyaura Cross-Coupling Reaction

In the case of the theoretical study of the Suzuki–Miyaura cross-coupling reaction between bromobenzene or 4-bromophenyl-diphenylphosphine oxide and phenylboronic acid, a pretransmetalation complex containing a Pd–O–B connected motif (**S_I**, Figure 1) was selected as the starting point in agreement with literature data.^21–23^ This species is ready for the transfer of the aryl substituent from the boron atom to the palladium center, which process has a barrier above 30 kJ mol^–1^ (**S_TS1**). Following elimination of the boronic acid from species **S_II**, **S_III** is the intermediate that undergoes reductive elimination via transition state **S_TS2** to provide the final product (**S_IV**). It is noteworthy that the Ph_2_P­(O)-substitution in positions 4 or 3 does not have a significant effect on the value of the barrier and on the relative stability of the intermediates and the products. The slight effect of the 4- and 3-Ph_2_P­(O)-substitution on the height of transition states **S_TS1** and **S_TS2** was opposite. Table 4 contains relative Gibbs free energy values also for the coupling with the Me- and MeO-substituted bromoarenes. These theoretical data are in agreement with the experimental data: the reactivity of the 4-Ph_2_P­(O)- and 3-Ph_2_P­(O)-bromobenzenes (**2A** and **2B**) was rather similar.

**Figure 1:**
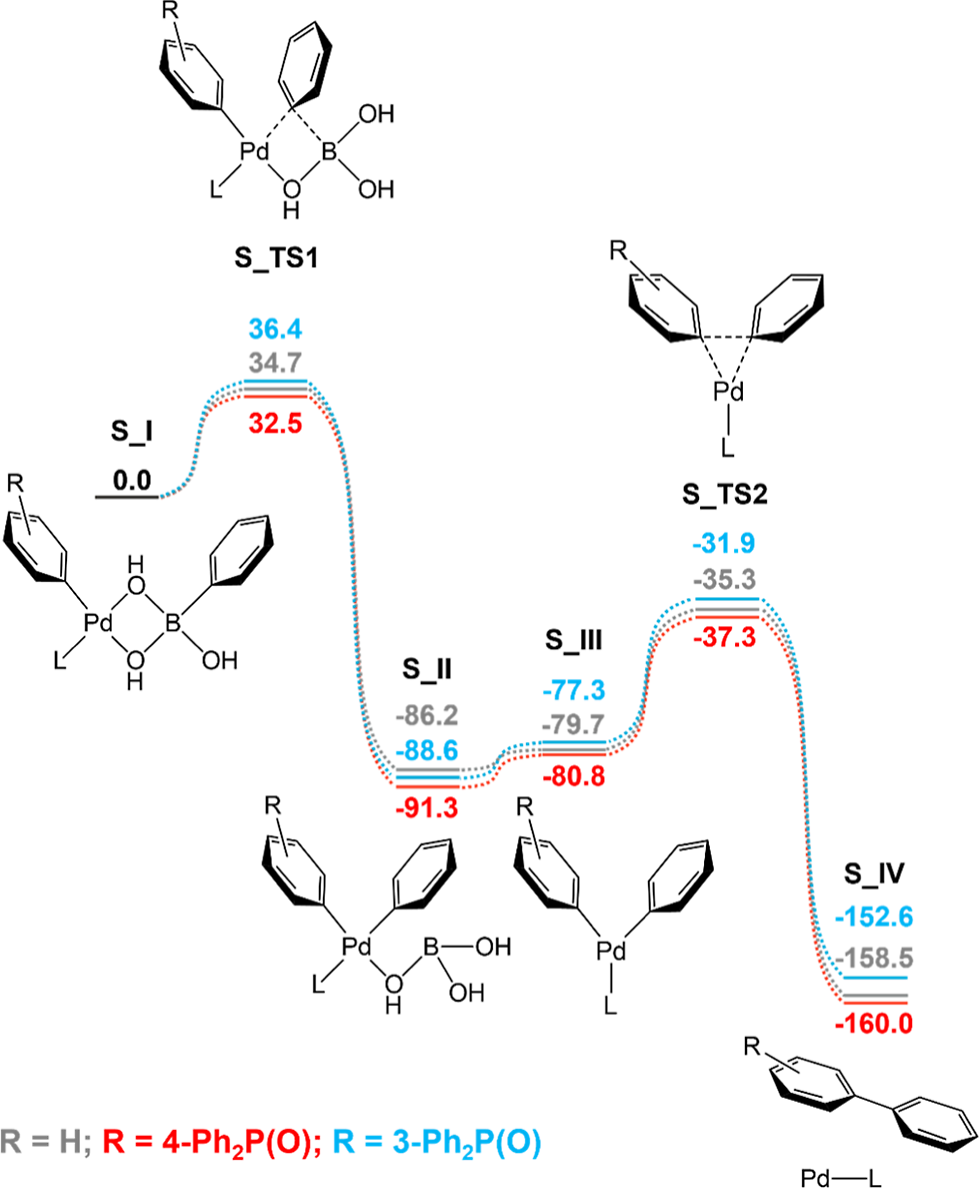
Calculated Gibbs free energy profile for the Suzuki–Miyaura cross-coupling reaction of bromobenzene and 4-bromophenyl- and 3-bromophenyl-diphenylphosphine oxides with phenylboronic acid. Relative Gibbs free energy values computed at the B3LYP/6-31G­(d,p) level of theory are given in kJ mol^–1^.

**Table 4:** Relative Gibbs Free Energies of the Intermediates and Transition States of the Suzuki–Miyaura Reaction with Different Bromoarenes and Phenylboronic Acid Computed at the B3LYP/6-31G­(d,p) Level of Theory

	*R*	Δ*G* (kJ/mol)					
		**S_I**	**S_TS1**	**S_II**	**S_III**	**S_TS2**	**S_IV**
	H	0.0	34.7	–86.2	–79.7	–35.3	–158.5
*para*	P(O)Ph_2_	0.0	32.5	–91.3	–80.8	–37.3	–160.0
	Me	0.0	35.0	–94.9	–79.2	–35.4	–156.5
	MeO	0.0	35.4	–96.6	–81.9	–36.5	–159.7
*meta*	P(O)Ph_2_	0.0	36.4	–88.6	–77.3	–31.9	–152.6
	Me	0.0	35.3	–86.5	–79.2	–35.2	–156.7
	MeO	0.0	36.2	–93.6	–77.6	–33.5	–158.2

#### 2.2.2 A Mechanistic Study on the Transition Metal-Catalyzed Michaelis–Arbuzov Reaction

In case of the Michaelis–Arbuzov reaction, the interaction of trimethyl phosphite with bromobenzene and with 4-bromophenyl-diphenylphosphine oxide was investigated computationally. The transformation is started by the oxidative addition of the aryl halide on the Ni(0) species, generating an aryl–nickel­(II) intermediate (**A_IIa**, Figure 2). This step is critical as it enables the activation of the aryl–Br bond that is otherwise resistant to a nucleophilic attack. The initially formed nickel­(II) intermediate (**A_IIa**) undergoes isomerization to furnish the thermodynamically more stable *trans*-configured complex (**A_IIb**). The transformation of **A_IIa** to **A_IIb** was not further investigated. Assuming a dissociative mechanism for the *cis–trans* isomerization, the dissociation of the ligand appears to be barrierless based on our potential energy surface scan calculations, with the energy of the system increasing continuously along the reaction coordinate. In the subsequent step, a bromide anion leaves the Ni center, generating a coordinatively unsaturated species (**A_III**), which is stabilized in the next step by a trimethyl phosphite molecule, which is attached to the central metal, resulting in **A_IV**. Formation of **A_III** has a high Gibbs free energy requirement of ca 160 kJ mol^–1^. In the following step, the bromide anion acts as a nucleophile and attacks one of the methyl groups in an S_N_2 fashion, providing intermediate **A_V** and MeBr. This (**A_IV → A_TS2)** step has a rather high activation barrier (Figure 2, 199.2–207.0 kJ mol^–1^), and hence, this is the rate-determining step of the overall process. Without any doubt, this high barrier may be overcome by the beneficial effect of MW irradiation. In the following reductive elimination step, the phenyl group in species **A_V** migrates from the Ni center to the phosphorus atom via transition state **A_TS3**, affording the aryl phosphonate and regenerating the catalytically active Ni(0) species. In this case, there is a significant Gibbs free energy difference (21.8 kJ mol^–1^) between the unsubstituted and the 4-Ph_2_P­(O)-substituted counterpart, meaning an increase in the barrier. As a comparison, the similar difference for the 3-Ph_2_P­(O)-substituted case is only 7.8 kJ mol^–1^. In contrast to 4-Ph_2_P­(O)-substitution, the presence of electron-donating Me- and MeO-substituents somewhat decreased the reaction barrier of the reductive elimination step (as characterized by 79.1–87.2 kJ mol^–1^). The sole exception was the case of the 3-MeO-substituted system, where the barrier (118.2 kJ mol^–1^) was higher than that for the unsubstituted counterpart (91.2 kJ mol^–1^). The effect of the substitution is less significant on the oxidative addition step; only a difference of a few kJ mol^–1^ was found. Relative Gibbs free energy values for the investigated species are listed in Table 5.

**Figure 2:**
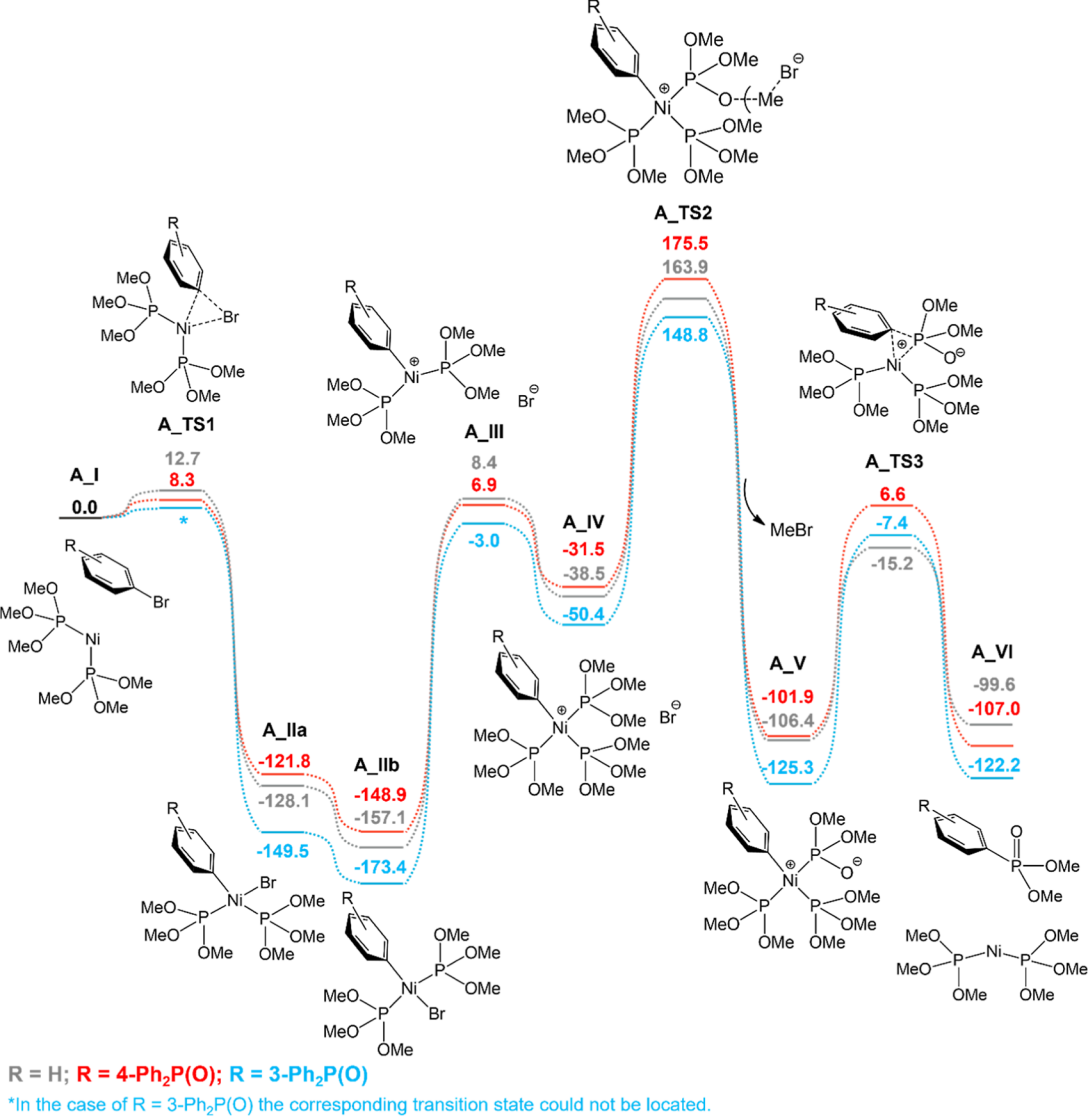
Calculated relative Gibbs free energy profile for the Michaelis–Arbuzov reaction of bromobenzene and 4-bromophenyl- and 3-bromophenyl-diphenylphosphine oxides with trimethyl phosphite. The Gibbs free energy values computed at the B3LYP/6-31G­(d,p) level with the PCM solvent model are given in kJ mol^–1^.

**Table 5:** Relative Gibbs Free Energies of the Intermediates and Transition States of the Michaelis–Arbuzov Reaction of Different Bromoarenes and Trimethyl Phosphite Computed at the B3LYP/6-31G­(d,p) Level of Theory (PCM = Acetonitrile Solvent Model Was Applied)

	*R*	Δ*G* (kJ mol^–1^)									
		**A_I**	**A_TS1**	**A_IIa**	**A_IIb**	**A_III**	**A_IV**	**A_TS2**	**A_V**	**A_TS3**	**A_VI**
	H	0.0	12.7	–128.1	–157.1	8.4	–38.5	163.9	–106.4	–15.2	–99.6
*para*	Ph_2_P(O)	0.0	8.3	–121.8	–148.9	6.9	–31.5	175.5	–101.9	6.6	–107.0
	Me	0.0	6.0	–131.1	–160.6	0.6	–48.1	162.5	–108.5	–21.3	–101.8
	OMe	0.0	7.8	–126.7	–156.6	9.7	–46.0	163.4	–107.1	–28.0	–97.8
*meta*	Ph_2_P(O)	0.0	–^a^	–149.5	–173.4	–3.0	–50.4	148.8	–125.3	–7.4	–122.2
	Me	0.0	9.2	–121.8	–147.8	14.0	–41.9	171.3	–98.8	–20.7	–91.9
	OMe	0.0	8.5	–114.6	–143.4	15.3	–34.8	178.3	–91.7	26.5	–101.8

a The corresponding transition state was not located.

#### 2.2.3 A Mechanistic Study on the Hirao Reaction

In the context of the Hirao cross-coupling, the reactions of differently substituted bromoarenes with diphenylphosphine oxide were investigated. The Gibbs free energy diagram (Figure 3) shows the situation for the P–C coupling of 4-bromophenyl-diphenylphosphine oxide and, as a comparison, for the reaction of bromobenzene (see the red dotted line and black dotted line, respectively). The catalytic cycle begins with the oxidative addition step, where the active Pd(0) species engages with bromobenzene. Initially, a weakly associated complex is formed through the interaction between the palladium center and the aromatic π-system of the phenyl ring (**H_I**). The Pd atom then undergoes insertion into the C–Br bond, affording the oxidative addition product **H_IIa** via transition state **H_TS1**. The species (**H_IIa**) containing the P ligands in the *cis* position is rearranged into the thermodynamically more stable *trans* isomer (**H_IIb**); similarly to the above-discussed Arbuzov reaction, this isomerization step was not further investigated. Following the oxidative addition, the dissociation of the bromide anion occurs in an endothermic step, providing the cationic system **H_III**. In order to obtain an appropriate model and to avoid accounting for charge separation, the bromide ion remained associated with the complex as a counterion during our computations. This state may be stabilized by the ethanol solvent molecules. In the next step, the vacant coordination site is occupied by a third P ligand to afford species **H_IV**. One of the P–OH groups of intermediate **H_IV** is then deprotonated by the base present in the reaction mixture, forming the zwitterionic species **H_V**. Considering the reaction conditions (the presence of a base and polar medium), this deprotonation proceeds probably via a low barrier. Reductive elimination via **H_TS2** gives product complex **H_VI**, whose dissociation (probably again via a low energy barrier) affords the product ArPh_2_P­(O) and regenerates the catalyst. It can be seen that the presence of the 4-Ph_2_P­(O) group slightly decreases the barriers and relative energy of the intermediates as compared to the unsubstituted case. Table 6 contains relative Gibbs free energy values for other model compounds as well. In contrast to the effect of the 4-Ph_2_P­(O) group, the electron-donating 4-methyl- and 4-methoxy substituents increase the activation barrier (**H_TS1**) for the oxidative addition. In case of the 3-Ph_2_P­(O)-substituted system, the barrier (**H_TS1**) slightly increases, while for the 3-methyl and 3-methoxy cases, the values remain practically unchanged. In summary, the energy profile of the substituted derivatives is similar to the unsubstituted counterpart; however, in most cases, the barrier somewhat increased. The, in most cases, negative effect of the electron-donating substituents in the aromatic ring is in accord with our earlier experiences.^19^ The comparably high values of the barriers for the oxidative addition and reductive elimination (86.4–101.6 kJ mol^–1^) justify the application of MW assistance.

**Figure 3:**
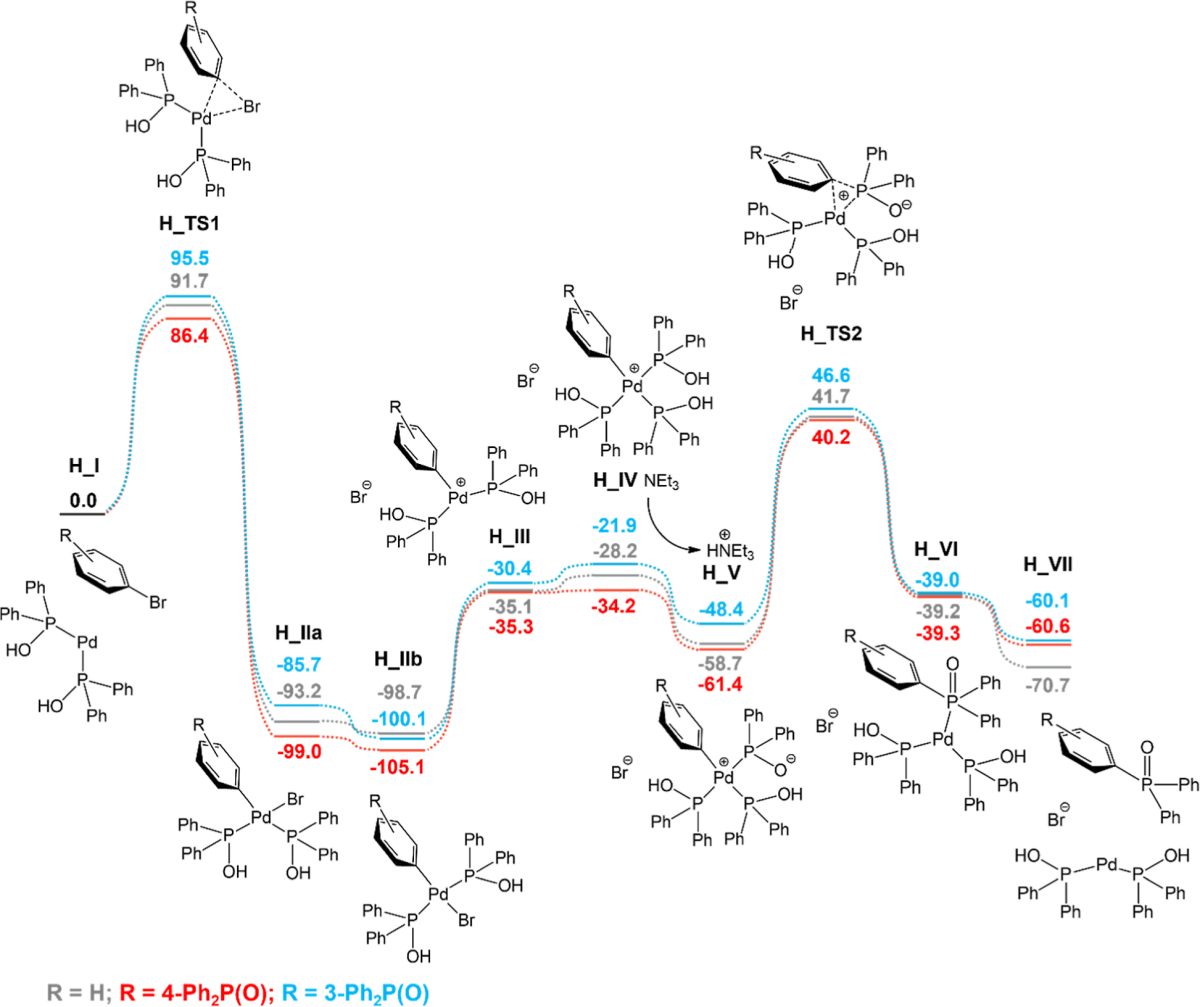
Calculated Gibbs free energy profile for the Hirao reaction of bromobenzene and 4-bromophenyl- and 3-bromophenyl-diphenylphosphine oxides with diphenylphosphine oxide. The relative Gibbs free energy values computed at the B3LYP/6-31G­(d,p) level with the PCM solvent model are given in kJ mol^–1^.

**Table 6:** Relative Gibbs Free Energies of the Intermediates and Transition States for the Hirao Reaction of Different Bromoarenes with Diphenylphosphine Oxide Computed at the B3LYP/6-31G­(d,p) Level of Theory (PCM = Acetonitrile Solvent Model Was Applied)^a^

	*R*	Δ*G* (kJ mol^–1^)									
		**H_1**	**H_TS1**	**H_IIa**	**H_IIb**	**H_III**	**H_IV**	**H_V**	**H_TS2**	**H_VI**	**H_VII**
	H	0.0	91.7	–93.2	–98.7	–35.1	–28.2	–58.7	41.7	–39.2	–70.7
*para*	Ph_2_P(O)	0.0	86.4	–99.0	–105.1	–35.3	–34.2	–61.4	40.2	–39.3	–60.6
	Me	0.0	97.7	–89.8	–96.1	–31.4	–24.6	–53.0	39.5	–34.6	–68.6
	OMe	0.0	94.5	–85.7	–94.9	–29.2	–27.0	–53.6	41.2	–32.8	–66.1
*meta*	Ph_2_P(O)	0.0	95.5	–85.7	–100.1	–30.4	–21.9	–48.4	46.6	–39.0	–60.1
	Me	0.0	90.6	–100.2	–100.6	–35.7	–28.5	–59.6	34.4	–38.0	–48.2
	OMe	0.0	92.7	–91.2	–97.9	–26.3	–25.7	–52.2	45.8	–39.4	–58.7

a For the Pd atom, the MWB28 basis set was applied.

## 3 Conclusions

The reactivity of 4- and 3-bromophenyl-diphenylphosphine oxides was evaluated in three catalytic reactions, namely, in the Suzuki–Miyaura and the Hirao cross-couplings, as well as in the Michaelis–Arbuzov reaction. The effect of reaction conditions was investigated, and the optimum conditions were explored, providing insights into a better understanding. The best conditions for the Suzuki–Miyaura reaction involved the use of 10% Pd­(OAc)_2_ in the presence of K_2_CO_3_ in EtOH at 120 °C under MW irradiation to afford the arylphenyl-diphenylphosphine oxides in 64–83% yields. As regards the Arbuzov reaction of bromophenyl-diphenylphosphine oxides with trialkyl phosphites at 160 °C, Pd­(OAc)_2_ was introduced by us as a novel catalyst. There was a need for the more active Pd catalyst to overcome the decreased reactivity of the phosphinoyl-bromobenzenes. The other route leading to bis­(P=O-functionalized) arenes was the Hirao P–C cross-coupling with different >P­(O)­H reagents, applying 10% of Pd­(OAc)_2_ and triethylamine at 150 °C in EtOH. The latter methods provided the target compounds in ca. 60% yields. The main route was accompanied by the formation of triphenylphosphine oxide as the minor byproduct. The 23 compounds synthesized were fully characterized. Complementary DFT calculations at the B3LYP/6-31G­(d,p) level of theory were carried out to interpret the experimental findings and to gain deeper understanding of the underlying structural and electronic features. In the Suzuki–Miyaura reactions, the 4- and 3-Ph_2_P­(O)-substitution had only a minor effect on the course of the reaction. In contrast, in the transition metal-catalyzed Michaelis–Arbuzov reaction, there are more significant differences in the energy barriers. The rate-limiting step is the elimination of MeBr, which has a high Gibbs free energy requirement. The activation barrier is slightly increased in the 4-Ph_2_P­(O)-substituted system, whereas substitution at the 3-position reduces this barrier. The energy barrier of the reductive elimination step is higher for the 4- and 3-Ph_2_P­(O)-substituted cases than that for the unsubstituted instance. At the same time, the presence of electron-donating substituents generally decreases the barrier of this step. In the Hirao cross-couplings, the 4-Ph_2_P­(O)-substituent leads to a slight decrease in the energy barrier of the oxidative addition, whereas the 3-Ph_2_P­(O)-substitution and the electron-donating 4-Me and 4-MeO groups result in a slight increase. The latter trend is in good accord with our earlier experiences. In all, our results expand the current knowledge of the studied systems and lay the groundwork for further mechanistic and functional investigations.

## 4 Experimental Section

### 4.1 General Information

The reactions were carried out in a CEM Discover Model SP (300 W) focused microwave reactor (Buckingham, UK) equipped with a stirrer and a pressure controller using 80–100 W irradiation under isothermal conditions. The reaction mixtures were irradiated in sealed glass vessels (with a volume of 10 mL) available from the supplier of CEM. The reaction temperature was monitored by an external IR sensor.

The ^31^P, ^13^C, and ^1^H NMR spectra were taken in CDCl_3_ solution on a Bruker Avance 300/500 spectrometer (Rheinstetten, Germany) operating at 121.5/202.4, 75.5/125.7, and 300/500 MHz, respectively. The ^31^P chemical shifts are downfield relative to those of H_3_PO_4_, while the reference for ^13^C and ^1^H chemical shifts was TMS. The couplings are given in hertz. The exact mass measurements were performed using an Agilent 6545 Q-TOF mass spectrometer (Santa Clara, CA, USA) in high-resolution, positive electrospray mode.

### 4.2 General Procedure for the Suzuki–Miyaura Coupling of 4- and 3-Bromophenyl-Diphenylphosphine Oxides (**2A** and **2B**) with Arylboronic Acids (**1a–g**)

To a MW glass vessel were added 1 mL of ethanol, 0.025 mmol (0.0055 g) of Pd­(OAc)_2_, 0.205 mmol (0.073 g) of 4-bromophenyl-diphenylphosphine oxide (**2A**) or 3-bromophenyl-diphenylphosphine oxide (**2B**), 0.246 mmol of arylboronic acid [phenylboronic acid (**1a**): 0.030 g, *o*-tolylboronic acid (**1b**): 0.034 g, *m*-tolylboronic acid (**1c**): 0.034 g, *p*-tolylboronic acid (**1d**): 0.034 g, 3-chlorophenylboronic acid (**1e**): 0.038 g, 4-chlorophenylboronic acid (**1f**): 0.038 g, 4-fluorophenylboronic acid (**1g**): 0.034 g], and 0.27 mmol (0.037 g) of potassium carbonate. The mixture was irradiated in a closed vial in the MW reactor at 120–135 °C for 20–90 min (Table 1). Then, the reaction mixture was diluted with 3 mL of EtOH and filtered, and the residue obtained after evaporation of the filtrate was passed through a thin (2–3 cm) layer of silica gel using ethyl acetate as the eluent. The crude mixture was analyzed by ^31^P NMR spectroscopy, and then it was purified by column chromatography (silica gel and dichloromethane–methanol 97:3 as the eluent) to afford products **3Aa–g** and **3Ba–g**.

The following products were thus prepared:

#### (1,1′-Biphenyl)-4-yl-diphenylphosphine oxide (**3Aa**)

White crystals; mp. 159–160 °C, mp.^16^ 157–158 °C; ^31^P NMR (CDCl_3_, 202.4 MHz):: δ 29.4; δ_P_^17^ (CDCl_3_, 162 MHz) 29.0; ^13^C NMR (CDCl_3_, 125.7 MHz): δ 144.8 (d, *J* = 2.8, C_4_), 139.9 (s, C_1_″), 132.6 (d, *J* = 10.1, C_2_)^a^, 132.5 (d, *J* = 105.2, C_1_′), 132.1 (d, *J* = 10,0, C_2_′)^b^, 132.0 (d, *J* = 2.8, C_4_′), 131.0 (d, *J* = 105.3, C_1_), 129.0 (s, C_3_″)^c^, 128.6 (d, *J* = 12.2, C_3_′)^b^, 128.2 (s, C_4_″), 127.3 (s, C_2_″)^c^, 127.2 (d, *J* = 12.5, C_3_)^a^, ^a,b,c^ may be reversed,: δ_C_^17^ (CDCl_3_, 100.6 MHz) 144.7 (d, *J* = 2.8), 139.9, 132.6 (d, *J* = 10.2), 132.5 (d, *J* = 107.3), 132.1 (d, *J* = 9.9), 132.0 (d, *J* = 2.7), 131.2 (d, *J* = 9.9), 131.1 (d, *J* = 105.2), 129.0, 128.6 (d, *J* = 12.1), 127.7 (d, *J* = 106.3), 127.3; ^1^H NMR (CDCl_3_, 500 MHz): δ 7.78–7.70 (m, 8H), 7.64–7.57 (m, 4H), 7.53–7.47 (m, 6H), 7.43–7.40 (m, 1H), δ_H_^17^ (CDCl_3_, 400 MHz) 7.78–7.64 (m, 8H), 7.59 (d, *J* = 7.6, 1H), 7.53 (d, *J* = 6.9, 1H), 7.48–7.42 (m, 6H), 7.37 (dd, *J* = 7.2, *J* = 7.2); [M + H]^+^ = 355.1256, C_24_H_20_OP requires 355.1252.

#### (2′-Methyl-[1,1′-biphenyl]-4-yl)­diphenylphosphine oxide (**3Ab**)

Pale yellow oil; ^31^P NMR (CDCl_3_, 202.4 MHz): δ 29.5; ^13^C NMR (CDCl_3_, 125.7 MHz): δ 145.8 (d, *J* = 2.8, C_4_), 140.7 (s, C_2_″), 135.2 (s, C_1_″), 132.5 (d, *J* = 104.3, C_1_′), 132.1 (d, *J* = 9.9, C_2_′)^a^, 132.0 (s, C_4_′), 131.96 (d, *J* = 13.4, C_2_)^b^, 130.8 (d, *J* = 105.0, C_1_), 130.5 (s, C_3_″)^c^, 129.6 (s, C_4_″)^c^, 129.4 (d, *J* = 12.4, C_3_)^b^, 128.6 (d, *J* = 12.1, C_3_′)^a^, 127.9 (s, C_6_″)^d^, 125.9 (s, C_5_″)^d^, 20.4 (s, CH_3_), ^a,b,c.d^ may be reversed; ^1^H NMR (CDCl_3_, 500 MHz): δ 7.76–7.68 (m, 6H), 7.58–7.55 (m, 2H), 7.51–7.47 (m, 4H), 7.43–7.41 (m, 2H), 7.29–7.21 (m, 4H), 2.26 (s, 3H, CH_3_); [M + H]^+^ = 369.1412, C_25_H_22_OP requires 369.1408.

#### (3′-Methyl-[1,1′-biphenyl]-4-yl)­diphenylphosphine oxide (**3Ac**)

White crystals; mp. 131–132 °C; ^31^P NMR (CDCl_3_, 202.4 MHz): δ 29.4; ^13^C NMR (CDCl_3_, 125.7 MHz): δ 144.9 (d, *J* = 2.7, C_4_), 139.9 (s, C_1_″), 138.6 (s, C_3_″), 132.5 (d, *J* = 103.9 C_1_′), 132.6 (d, *J* = 10.1, C_2_)^a^, 132.1 (d, *J* = 10.1, C_2_′)^b^, 132.0 (d, *J* = 2.8, C_4_′), 130.8 (d, *J* = 105.5, C_1_), 128.93 (s, C_5_″)^c^, 128.87 (s, C_4_″)^c^, 128.6 (d, *J* = 12.0, C_3_′)^b^, 128.1 (s, C_2_″)^c^, 127.2 (d, *J* = 12.4, C_3_)^a^, 124.4 (s, C_6_″), 21.5 (s, CH_3_), ^a,b,c.d^ may be reversed; ^1^H NMR (CDCl_3_, 500 MHz): δ 7.77–7.68 (m, 8H), 7.60–7.56 (m, 2H), 7.52–7.48 (m, 4H), 7.43–7.41 (m, 2H), 7.36 (t, *J* = 7.6, 1H), 7.22 (d, *J* = 7.5, 1H), 2.44 (s, 3H, CH_3_); [M + H]^+^ = 369.1398, C_25_H_22_OP requires 369.1408.

#### (4′-Methyl-[1,1′-biphenyl]-4-yl)­diphenylphosphine oxide (**3Ad**)

White crystals; mp. 159–160 °C; ^31^P NMR (CDCl_3_, 202.4 MHz): δ 29.5; δ_P_^23^ (CDCl_3_, 101 MHz) 30.1; δ_C_^23^ (CDCl_3_, 100 MHz) 144.7, 138.2, 132.8 (d, *J* = 104.0), 132.6 (d, *J* = 10.0), 132.3, 132.2 (d, *J* = 9.5), 131.9, 130.8 (d, *J* = 103.1), 129.7, 128.5 (d, *J* = 12.9), 127.1, 127.0 (d, *J* = 12.7), 21.2; δ_H_^23^ (CDCl_3_, 400 MHz) 7.76–7.64 (m, 8H), 7.58–7.44 (m 8H), 7.27 (d, *J* = 8.4, 2H), 2.40 (s, 3H); [M + H]^+^ = 369.1406, C_25_H_22_OP requires 369.1408.

#### (3′-Chloro-[1,1′-biphenyl]-4-yl)­diphenylphosphine oxide (**3Ae**)

White crystals; mp. 175–176 °C; ^31^P NMR (CDCl_3_, 202.4 MHz): δ 28.9; ^13^C NMR (CDCl_3_, 125.7 MHz): δ 143.3 (d, *J* ∼ 2.3, C_4_), 141.7 (s, C_1_″), 134.9 (s, C_3_″), 132.7 (d, *J* = 10.2, C_2_)^a^, 132.3 (d, *J* = 103.5, C_1_′), 132.1 (d, *J* = 10.0, C_2_′)^b^, 132.08 (d, *J* = 2.7, C_4_′), 131.9 (d, *J* = 104.3, C_1_), 130.2 (s, C_5_″), 128.6 (d, *J* = 12.2, C_3_′)^b^, 128.2 (s, C_2_″)^c^, 127.4 (s, C_4_″)^c^, 127.2 (d, *J* = 12,5, C_3_)^a^, 125.4 (s, C_6_″), ^a,b,c^ may be reversed; ^1^H NMR (CDCl_3_, 500 MHz): δ 7.79–7.70 (m, 6H), 7.68–7.65 (m, 2H), 7.59–7.56 (m, 3H), 7.51–7.47 (m, 5H), 7.42–7.35 (m, 2H); [M + H]^+^ = 389.0865, C_24_H_19_ClOP requires 389.0862.

#### (4′-Chloro-[1,1′-biphenyl]-4-yl)­diphenylphosphine oxide (**3Af**)

Colorless oil; ^31^P NMR (DMSO, 121.5 MHz): δ 25.5; ^13^C NMR (DMSO, 75.4 MHz): δ 142.7 (d, *J* = 2.9, C_4_), 138.2 (s, C_1_″), 133.7 (s, C_4_″), 133.1 (d, *J* = 105.5, C_1_′), 132.7 (d, *J* = 10.5, C_2_)^a^, 132.6 (d, *J* ∼ 2.0, C_4_′), 132.3 (d, *J* = 103.4, C_1_), 132.0 (d, *J* = 9.8, C_2_′)^b^, 129.5 (s, C_3_″)^c^, 129.3 (d, *J* = 11.8, C_3_′)^b^, 129.2 (s, C_2_″)^c^, 127.4 (d, *J* = 12.1, C_3_)^a^, ^a,b,c^ may be reversed; ^1^H NMR (DMSO, 300 MHz): δ 7.87–7.53 (m, 13H), 7.43–7.26 (m, 5H); [M + H]^+^ = 389.0865, C_24_H_19_ClOP-re requires 389.0862.

#### (4′-Fluoro-[1,1′-biphenyl]-4-yl)­diphenylphosphine oxide (**3Ag**)

White crystals; mp. 175–176 °C; ^31^P NMR (CDCl_3_, 202.4 MHz): δ 29.0; δ_P_^23^ (CDCl_3_, 101 MHz) 29.9; ^13^C NMR (CDCl_3_, 125.7 MHz): δ 162.9 (d, *J* = 248.0, C_4_″), 143.7 (d, *J* = 2.7, C_4_), 136.0 (d, *J* = 3.3 C_1_″), 132.7 (d, *J* = 10.2, C_2_)^a^, 132.5 (d, *J* = 104.4, C_1_′), 132.1 (d, *J* = 10.3, C_2_′)^b^, 132.0 (d, *J* ∼ 2.9, C_4_′), 131.2 (d, *J* = 105.0, C_1_), 128.9 (d, *J* = 8.2, C_2_″), 128.6 (d, *J* = 12.0, C_3_′)^a^, 127.0 (d, *J* = 12.4, C_3_)^b^, 115.9 (d, *J* = 21.7, C_3_″), ^a,b^ may be reversed; ^1^H NMR (CDCl_3_, 500 MHz): δ 7.76–7.69 (m, 6H), 7.64 (dd, *J*_1_ = 8.3, *J*_2_ = 2.6, 2H), 7.59–7.54 (m, 4H), 7.51–7.47 (m, 4H), 7.15 (t, *J* = 8.6, 2H),: δ_H_^23^ (CDCl_3_, 400 MHz) 7.76–7.69 (m, 6H), 7.63 (dd, *J* = 8.1, *J* = 2.0, 2H), 7.59–7.54 (m, 4H), 7.50–7.46 (m, 4H), 7.15 (dd, *J* = 8.7, *J* = 8.7, 2H); [M + H]^+^ = 373.1160, C_24_H_19_FOP requires 373.1158.

#### (1,1′-Biphenyl)-3-yl-diphenylphosphine oxide (**3Ba**)

White crystals; mp. 175–176 °C; ^31^P NMR (CDCl_3_, 202.4 MHz): δ 29.4; δ_P_^18^ (CDCl_3_, 121.5 MHz) 30.5; ^13^C NMR (CDCl_3_, 125.7 MHz): δ 141.6 (d, *J* = 11.8, C_3_), 140.0 (s, C_1_″), 133.1 (d, *J* = 103.4, C_1_), 132.4 (d, *J* = 104.2, C_1_′), 132.13 (d, *J* = 10.0, C_2_′)^a^, 132.06 (d, *J* = 2.6, C_4_′), 130.9 (d, *J* = 10.0, C_6_), 130.70 (d, *J* ∼ 2.0, C_4_), 130.67 (d, *J* = 13.2, C_2_), 128.93 (d, *J* = 12.7, C_5_), 128.90 (s, C_2_″)^b^, 128.6 (d, *J* = 12.1, C_3_′)^a^, 127.9 (s, C_4_″), 127.2 (s, C_3_″)^b^, ^a,b^ may be reversed,: δ_C_^18^ (CDCl_3_, 75.5 MHz) 141.6 (d, *J* = 11.9), 140.0, 133.9, 133.2, 132.2, 132.1, 131.8, 130.8.1 (d, *J* = 10.1), 130.7 (d, *J* = 2.9), 130.6, 128.9, 128.8, 128.6 (d, *J* = 12.1), 127.9, 127,23; ^1^H NMR (CDCl_3_, 500 MHz): δ 7.99 (dt, *J* = 12.7, *J* = 1.7, 1H), 7.79 (dd, *J* = 7.7, *J* = 1.7, 1H), 7.76–7.72 (m, 4H), 7.64–7.53 (m, 6H), 7.51–7.48 (m, 4H), 7.43 (t, *J* = 7.5, 2H), 7.36 (t, *J* = 7.3, 1H),: δ_H_^18^ (CDCl_3_, 300 MHz) 7.91–6.96 (m, 19H); [M + H]^+^ = 355.1252, C_24_H_20_OP requires 355.1252.

#### (4′-Methyl-[1,1′-biphenyl]-3-yl)­diphenylphosphine oxide (**3Bd**)

Colorless oil; ^31^P NMR (CDCl_3_, 202.4 MHz): δ 29.4; δ_P_^24^ (CDCl_3_, 162 MHz) 30.6; ^13^C NMR (CDCl_3_, 125.7 MHz): δ 141.5 (d, *J* = 12.1, C_3_), 137.7 (s, C_4_″), 137.1 (s, C_1_″), 133.0 (d, *J* = 102.9, C_1_), 132.5 (d, *J* = 104.3, C_1_′), 132.1 (d, *J* = 10.0, C_2_′)^a^, 132.0 (d, *J* = 2.8, C_4_′), 130.6 (d, *J* = 10.4, C_6_)^b^, 130.47 (d, *J* = 2.8, C_4_), 130.46 (d, *J* = 10.0, C_2_)^b^, 129.6 (s, C_2_″)^c^, 128.9 (d, *J* = 12.9, C_5_), 128.5 (d, *J* = 12.3, C_3_′)^a^, 127.0 (s, C_3_″)^c^, 21.1 (s, CH_3_), ^a,b,c^ may be reversed,: δ_C_^24^ (CDCl_3_) 141.3 (*J* = 11.2), 137.5, 136.9, 133.0 (*J* = 103.7), 132.5 (*J* = 103.8), 131.9 (*J* = 10.3), 131.8 (*J* = 2.0), 130.3 (*J* = 18.9), 130.3 (*J* = 2.0), 130.2 (*J* = 5.8), 129.4, 128.7 (*J* = 13.2), 128.4 (*J* = 12.2), 126.9, 20.9 (CH_3_); ^1^H NMR (CDCl_3_, 500 MHz): δ 7.96 (dt, *J* = 12.7, *J* = 1.7, 1H), 7.79–7.71 (m, 5H), 7.61–7.45 (m, 10H), 7.26–7.25 (d, *J* = 7.9, 2H), 2.40 (s, 3H, CH_3_),: δ_H_^24^ (CDCl_3_) 8.00–6.90 (ArH), 2.40 (s, 3H, CH_3_); [M + H]^+^ = 369.1405, C_25_H_22_OP requires 369.1408.

#### (3′-Chloro-[1,1′-biphenyl]-3-yl)­diphenylphosphine oxide (**3Be**)

Colorless oil; ^31^P NMR (DMSO, 121.5 MHz): δ 25.7; ^13^C NMR (DMSO, 75.4 MHz): δ 141.7 (d, *J* = 11.6, C_3_), 139.5 (s, C_1_″), 134.3 (s, C_3_″), 133.2 (s, C), 132.77 (d, *J* = 101.0, C_1_), 132.75 (d, *J* = 2.4, C_4_′), 132.1 (d, *J* = 102.3, C_1_′), 132.0 (d, *J* = 9.9, C_2_′)^a^, 130.91 (d, *J* = 9.6, C_2_)^b^, 130.86 (d, *J* = 2.8, C_4_), 130.1 (d, *J* = 12.6, C_5_), 129.9 (d, *J* = 10.0, C_6_)^b^, C_5_″ is overlapped, 129.3 (d, *J* = 11.9, C_3_′)^a^, 128.4 (s, C_4_″), 127.0 (s, C_2_″)^c^, 126.1 (s, C_6_″)^c^, ^a,b,c^ may be reversed; ^1^H NMR (DMSO, 300 MHz): δ 7.72–7.11 (m, 18H); [M + H]^+^ = 389.0862, C_24_H_19_ClOP requires 389.0862.

#### (4′-Chloro-[1,1′-biphenyl]-3-yl)­diphenylphosphine oxide (**3Bf**)

Colorless oil; ^31^P NMR (CDCl_3_, 202.4 MHz): δ 27.9; ^13^C NMR (CDCl_3_, 125.7 MHz): δ 146.4 (d, *J* = 8.4, C_3_), 138.6 (d, *J* = 4.2, C_1_″), 134.0 (d, *J* = 12.1, C_6_), 133.3 (s, C_4_″), 132.7 (d, *J* = 104.5, C_1_′), 131.94 (d, *J* = 101.6, C_1_), 131.86 (d, *J* = 2.7, C_4_), 131.79 (d, *J* = 9.9, C_5_), 131.6 (d, *J* = 9.3, C_2_′)^a^, 131.5 (s, C_2_″)^b^, 131.4 (d, *J* = 2.8, C_4_′), 128.2 (d, *J* = 12.0, C_3_′)^a^, 127.3 (s, C_3_″)^b^, 126.9 (d, *J* = 12.6, C_2_), ^a,b^ may be reversed; ^1^H NMR (CDCl_3_, 500 MHz): δ 7.62–7.56 (m, 5H), 7.47–7.28 (m, 9H), 7.16–7.13 (m, 2H), 7.04–7.01 (m, 2H); [M + H]^+^ = 389.0861, C_24_H_19_ClOP requires 389.0862.

#### (4′-Fluoro-[1,1′-biphenyl]-3-yl)­diphenylphosphine oxide (**3Bg**)

White crystals; mp. 124–125 °C; ^31^P NMR (CDCl_3_, 202.4 MHz): δ 29.2; ^13^C NMR (CDCl_3_, 125.7 MHz): δ 162.7 (d, *J* = 247.5, C_4_″), 140.6 (d, *J* = 12.0, C_3_), 136.1 (d, *J* = 3.2, C_1_″), 133.3 (d, *J* = 103.1, C_1_), 132.4 (d, *J* = 104.1, C_1_′), 132.09 (d, *J* = 2.7, C_4_′), 132.08 (d, *J* = 9.9, C_2_′)^a^, 130.8 (d, *J* = 10.4, C_6_)^b^, 130.5 (d, *J* = 2.1, C_4_), 130.47 (d, *J* = 9.8, C_2_)^b^, 129.0 (d, *J* = 12.9, C_5_), 128.8 (d, *J* = 8.2, C_2_″), 128.6 (d, *J* = 12.2, C_3_′)^a^, 115.8 (d, *J* = 21.4, C_3_″), ^a,b^ may be reversed; ^1^H NMR (CDCl_3_, 500 MHz): δ 7.95 (dt, *J* = 12.5, *J* = 1.7, 1H), 7.73–7.69 (m, 5H), 7.59–7.47 (m, 10H), 7.11 (t, *J* = 8.6, 2H); [M + H]^+^ = 373.1157, C_24_H_19_FOP requires 373.1158.

### 4.3 General Procedure for the Michaelis–Arbuzov Reaction of 4- and 3-Bromophenyl-Diphenylphosphine Oxides (**2A** and **2B**) with Trialkyl Phosphites

To a MW glass vessel were added 0.035 mmol (0.0079 g) of Pd­(OAc)_2_, 0.36 mmol (0.13 g) of 4-bromophenyl-diphenylphosphine oxide (**2A**) or 3-bromophenyl-diphenylphosphine oxide (**2B**), and 0.54 mmol (0.092 mL) of triethyl phosphite or 0.54 mmol (0.064 mL) of trimethyl phosphite. The mixture was irradiated in a closed vial in the MW reactor at 160–180 °C for 1–4 h (Table 2). Then, the reaction mixture was diluted with 3 mL of dichloromethane and filtered, and the residue obtained after evaporation of the filtrate was passed through a thin (2–3 cm) layer of silica gel using dichloromethane/methanol = 97:3 as the eluent. The crude mixture was analyzed by ^31^P NMR spectroscopy, and then it was purified by column chromatography (silica gel and dichloromethane–methanol 97:3 as the eluent) to afford products **4Aa**, **4Ab**, **4Ba**, and **4Bb**.

The following products were thus prepared.

#### Diethyl (4-Diphenylphosphinoyl-phenyl)­phosphonate (**4Aa**)

Colorless oil; ^31^P NMR (CDCl_3_, 202.4 MHz): δ_P_ 28.9 (P­(C_6_H_5_)_2_), 16.7 (P­(OCH_2_CH_3_)_2_), d, *J* = 3.8,: δ_P_^15^ (CDCl_3_, 202.4 MHz) 28.4 (m, P­(C_6_H_5_)_2_), 16.8 (d, *J* = 3.7, P­(OCH_2_CH_3_)_2_); ^13^C NMR (CDCl_3_, 125.7 MHz): δ 137.3 (dd, *J*_*1*_ = 100.5, *J*_*2*_ = 3.0, C_1_), 132.6 (dd, *J*_1_ = 186.9, *J*_2_ = 2.9, C_4_), 132.3 (d, *J* = 2.8, C_4_′), 132.1 (d, *J* = 10.0, C_2_′)^a^, 132.0 (dd, *J*_1_ = 14.9, *J*_2_ = 9.8, C_2_), 131.7 (d, *J* ∼ 105, C_1_′), 131.6 (dd, *J*_1_ = 11.8, *J*_2_ = 10.0, C_3_), 128.7 (d, *J* = 12.2, C_3_′)^a^, 62.5 (d, *J* = 5.6, OCH_2_), 16.4 (d, *J* = 6.4, CH_3_), ^a^ may be reversed; ^1^H NMR (CDCl_3_, 500 MHz): δ 7.90 (ddd, *J*_1_ = 12.8, *J*_2_ = 8.3, *J*_3_ = 2.5, 2H), 7.78 (ddd, *J*_1_ = 11.6, *J*_2_ = 8.1, *J*_3_ = 3.9, 2H), 7.67 (ddd, *J*_1_ = 12.1, *J*_2_ = 7.8, *J*_3_ = 1.3, 4H), 7.58 (tm, *J* = 7.5, 2H), 7.49 (td, *J*_1_ = 7.6, *J*_2_ = 2.9, 4H), 4.18 (m), 4.10 (m, 4H, OCH_2_), 1.34 (t, *J* = 7.1, 6H, CH_3_); [M + H]^+^ = 415.1228, C_22_H_25_O_4_P_2_ requires 415.1228.

#### Dimethyl (4-Diphenylphosphinoyl-phenyl)­phosphonate (**4Ab**)

Pale yellow oil; ^31^P NMR (CDCl_3_, 202.4 MHz): δ_P_ 28.7 (P­(C_6_H_5_)_2_), 19.6 (P­(OCH_3_)_2_), d, *J* = 3.7; ^13^C NMR (CDCl_3_, 125.7 MHz): δ 137.6 (dd, *J*_1_ = 100.1, *J*_2_ = 2.6, C_1_), C_4_ is overlapped, 132.3 (d, *J* = 2.6, C_4_′), 132.0 (d, *J* = 10.0, C_2_′)^a^, C_2_ is overlapped, 131.6 (d, *J* = 103.7, C_1_′), C_3_ is overlapped, 128.7 (d, *J* = 12.3, C_3_′)^a^, 52.9 (d, *J* = 5.7, OCH_3_), ^a^ may be reversed; ^1^H NMR (CDCl_3_, 500 MHz): δ 7.93–7.88 (m, 2H), 7.83–7.78 (m, 2H), 7.70–7.66 (m, 4H), 7.61–7.57 (m, 2H), 7.52–7.48 (m, 4H), 3.80 (d, *J* = 11.1, 6H, OCH_3_); [M + H]^+^ = 387.0911, C_20_H_21_O_4_P_2_ requires 387.0915.

#### Diethyl (3-Diphenylphosphinoyl-phenyl)­phosphonate (**4Ba**)

Colorless oil; ^31^P NMR (CDCl_3_, 202.4 MHz): δ_P_ 29.1 (P­(C_6_H_5_)_2_), 16.6 (P­(OCH_2_CH_3_)_2_, d, *J* = 4.9,: δ_P_^15^ (CDCl_3_, 202.4 MHz) 28.3 (m, P­(C_6_H_5_)_2_), 16.7 (d, *J* = 5.0, P­(OCH_2_CH_3_)_2_); ^13^C NMR (CDCl_3_, 125.7 MHz): δ 135.8 (dd, *J*_1_ = 9.7, *J*_2_ = 2.8, C_6_), 135.2 (dd, *J*_1_ = 10.0, *J*_2_ = 2.6, C_4_), 135.0 (t, *J* = 10.6, C_2_), 133.6 (dd, *J*_1_ = 102.1, *J*_2_ = 13.7, C_1_), 132.3 (d, *J* = 2.9, C_4_′), 132.1 (d, *J* = 10.0, C_2_′)^a^, 132.0 (d, *J* = 104.8, C_1_′), 129.5 (dd, *J*_1_ = 188.6, *J*_2_ = 11.3, C_3_), 128.75 (dd, *J*_1_ = 14.2, *J*_2_ = 11.2, C_5_), 128.7 (d, *J* = 12.3, C_3_′)^a^, 62.5 (d, *J* = 5.7, CH_2_), 16.3 (d, *J* = 6.4, CH_3_), ^a^ may be reversed; ^1^H NMR (CDCl_3_, 500 MHz): δ 8.06 (t, *J* = 12.6, 1H), 8.01 (ddq, *J*_1_ = 13.1, *J*_2_ = 7.7, *J*_3_ = 1.5, 1H), 7.89 (ddq, *J*_1_ = 11.7, *J*_2_ = 7.8, *J*_3_ = 1.5, 1H), 7.67 (ddd, *J*_1_ = 12.1, *J*_2_ = 8.0, *J*_3_ = 1.4, 4H), 7.59 (tt, *J*_1_^b^, *J*_2_ = 3.3, 1H), 7.57 (tq, *J*_1_ = 7.5, *J*_2_ = 1.5, 2H), 7.48 (td, *J*_1_ = 7.6, *J*_2_ = 2.9, 4H), 4.12 (m), 4.05 (m, 4H, CH_2_), 1.27 (t, *J* = 7.1, 6H, CH_3_), ^b^ the coupling could not be detected due to overlapping signals; [M + H]^+^ = 415.1233, C_22_H_25_O_4_P_2_ requires 415.1228.

#### Dimethyl (3-Diphenylphosphinoyl-phenyl)­phosphonate (**4Bb**)

Pale yellow oil; ^31^P NMR (CDCl_3_, 202.4 MHz): δ 28.5 (P­(C_6_H_5_)_2_), 19.5 (P­(OCH_3_)_2_), d, *J* = 5.0; ^13^C NMR (CDCl_3_, 125.7 MHz): δ 136.0 (dd, *J*_1_ = 10.0, *J*_2_ = 2.6, C_6_), 135.2 (dd, *J*_1_ = 10.5, *J*_2_ = 2.5, C_4_), 135.0 (t, *J* = 10.3, C_2_), 133.6 (dd, *J*_1_ = 103.3, *J*_2_ = 13.5, C_1_), 132.3 (d, *J* = 2.8, C_4_′), 132.0 (d, *J* = 10.1, C_2_′)^a^, 131.7 (d, *J* = 105.1, C_1_′), 128.8 (dd, *J*_1_ = 14.3, *J*_2_^b^, C_5_), 128.7 (d, *J* = 12.3, C_3_′)^a^, 128.1 (dd, *J*_1_ = 189.1, *J*_2_ = 11.2, C_3_), 52.9 (d, *J* = 5.8, OCH_3_), ^a^ may be reversed, ^b^ the coupling could not be detected due to overlapping signals; ^1^H NMR (CDCl_3_, 500 MHz): δ 8.11 (t, *J* = 12.4, 1H), 8.03–7.83 (m, 2H), 7.70–7.46 (m, 11H), 3.74 (t, *J* = 11.1, 6H, OCH_3_); [M + Na]^+^ = 409.0733, C_20_H_20_O_4_P_2_Na requires 409.0735.

### 4.4 General Procedure for the Hirao Cross-Coupling of 4- and 3-Bromophenyl-Diphenylphosphine Oxides (**2A** and **2B**) with >P­(O)H Compounds

To a MW glass vessel were added 0.029 mmol (0.0064 g) of Pd­(OAc)_2_, 0.29 mmol (0.10 g) of 4-bromophenyl-diphenylphosphine oxide (**2A**) or 3-bromophenyl-diphenylphosphine oxide (**2B**), 0.37 mmol of >P­(O)H compounds [diethyl phosphite: 0.048 mL; dibutyl phosphite: 0.072 mL; ethyl phenyl *H*-phosphinate: 0.056 mL; diphenylphosphine oxide: 0.075 g], 0.31 mmol (0.044 mL) of triethylamine, and 1 mL of ethanol. The mixture was irradiated in a closed vial in the MW reactor at 150 °C for 30–60 min (Table 3). The mixture was diluted with 3 mL of EtOH and filtered, and the residue obtained after evaporation of the volatile components was passed through a thin (2–3 cm) layer of silica gel using ethyl acetate as the eluent. The crude mixture was analyzed by ^31^P NMR spectroscopy, then it was purified by column chromatography (silica gel and dichloromethane - methanol 97:3 as the eluent) to afford products **4Aa,c,d** and **4Ba,c–e**.

The following products were thus prepared:

#### Dibutyl (4-Diphenylphosphinoyl-phenyl)­phosphonate (**4Ac**)

Colorless oil.; ^31^P NMR (CDCl_3_, 202.4 MHz): δ 28.5 (P­(C_6_H_5_)_2_), 16.8 (P­(OCH_2_CH_2_CH_2_CH_3_)_2_), d, *J* = 3.7; ^13^C NMR (CDCl_3_, 125.7 MHz): δ 137.1 (dd, *J*_1_ = 100.4, *J*_2_ = 1.7, C_1_), 132.5 (dd, *J*_1_ = 186.7, *J*_2_ = 2.6, C_4_), 132.3 (d, *J* = 2.7, C_4_′), 132.1 (d, *J* = 9.9, C_2_′)^a^, C_2_ and C_3_are overlapped, 131.7 (d, *J* = 105.3, C_1_′), 131.5 (dd, *J*_*1*_ = 10.9, *J*_*2*_ ∼ 2.9, C_3_), 128.7 (d, *J* = 12.2, C_3_′)^a^, 66.2 (d, *J* = 5.9, CH_2_), 32.4 (d, *J* = 6.4, CH_2_), 18.7, (s, CH_2_), 13.5 (s, CH_3_), ^a^ may be reversed; ^1^H NMR (CDCl_3_, 500 MHz): δ 7.94–7.46 (m, 14H), 4.16–3.98 (m, 4H, OCH_2_), 1.71–1.62 (m, 4H, CH_2_), 1.45–1.32 (m, 4H, CH_2_), 0.91 (t, *J* = 7.4, 6H, CH_3_); [M + H]^+^ = 471.1849, C_26_H_32_O_4_P_2_ requires 471.1854.

#### Ethyl (4-Diphenylphosphinoyl-phenyl-)­phenylphosphinate (**4Ad**)

Colorless oil; ^31^P NMR (CDCl_3_, 202.4 MHz): δ 29.1 (P­(OCH_2_CH_3_) (C_6_H_5_)), 30.1 (P­(C_6_H_5_)_2_), d, *J* = 3.5; ^13^C NMR (CDCl_3_, 125.7 MHz): δ 137.3 (dd, *J*_*1*_ = 100.5, *J*_*2*_ = 3.0, C_1_), 132.6 (d, *J* = 2.8, C_4_″), C_1"_ and C_4_ are overlapped, 132.3 (d, *J* = 2.8, C_4_′), 132.1 (d, *J* = 9.9, C_2_′)^a^, 132.0 (dd, *J*_1_ = 14.9, *J*_2_ = 9.8, C_2_), 131.7 (d, *J* ∼ 105, C_1_′), 131.8 (d, *J* = 10.2, C_2_″)^b^, 131.6 (dd, *J*_1_ = 11.8, *J*_2_ = 10.0, C_3_), 128.71 (d, *J* = 13.4, C_3_″)^b^, 128.68 (d, *J* = 12.3, C_3_′)^a^, 61.2 (d, *J* = 6.0, OCH_2_), 16.5 (d, *J* = 6.5, CH_3_), ^a,b^ may be reversed; ^1^H NMR (CDCl_3_, 500 MHz): δ 7.95–7.72 (m, 6H), 7.69–7.45 (m, 13H), 4.19–4.08 (m, 2H, OCH_2_), 1.39 (t, *J* = 7.0, 3H, CH_3_); [M + H]^+^ = 447.1293, C_26_H_25_O_3_P_2_ requires 447.1274.

#### Dibutyl (3-Diphenylphosphinoyl-phenyl)­phosphonate (**4Bc**)

Colorless oil; ^31^P NMR (CDCl_3_, 202.4 MHz): δ 28.6 (P­(C_6_H_5_)_2_), 16.7 (P­(OCH_2_CH_2_CH_2_CH_3_)_2_), d, *J* = 5.0; ^13^C NMR (CDCl_3_, 125.7 MHz): δ 135.8 (dd, *J*_1_ = 9.7, *J*_2_ = 2.7, C_6_), 135.2 (dd, *J*_1_ = 10.4, *J*_2_ = 2.4, C_4_), 134.9 (t, *J* = 10.7, C_2_), 133.5 (dd, *J*_1_ = 102.0, *J*_2_ = 13.7, C_1_), 132.2 (d, *J* = 2.8, C_4_′), 132.1 (d, *J* = 10.0, C_2_′)^a^, 131.8 (d, *J* = 104.9, C_1_′), 129.4 (dd, *J*_1_ = 189.4, *J*_2_ = 11.2, C_3_), 128.8 (dd, *J*_1_ = 14.6, *J*_2_^b^, C_5_), 128.6 (d, *J* = 12.3, C_3_′)^a^, ^a^ may be reversed, ^b^ the coupling could not be detected due to overlapping signals; ^1^H NMR (CDCl_3_, 500 MHz): δ 8.06–7.90 (m, 2H), 7.71–7.46 (m, 11H), 4.10–3.93 (m, 4H, OCH_2_), 1.64–1.55 (m, 4H, CH_2_), 1.39–1.26 (m, 4H, CH_2_), 0.88 (t, *J* = 7.4, 6H, CH_3_); [M + H]^+^ = 471.1860, C_26_H_32_O_4_P_2_ requires 471.1854.

#### 1,3-Phenylenebis­(diphenylphosphine oxide) (**4Be**)

Colorless oil; ^31^P NMR (CDCl_3_, 202.4 MHz) 28.5,: δ_P_^25^ (CDCl_3_, 162 MHz) 30.5; ^13^C NMR (CDCl_3_, 125.7 MHz): δ 135.5 (dd, *J*_*1*_ = 10.1, *J*_2_ = 3.1, C_3_), 135.4 (t, *J* = 11.2, C_1_), 133.6 (dd, *J*_1_ = 101.8, *J*_2_ = 10.7, C_2_), 132.2 (d, *J* = 2.3, C_4_′)^a^, 131.95 (d, *J* = 10.2, C_2_′)^a^, 131.7 (d, *J* = 105.1, C_1_′), 128.95 (t, *J* = 11.3, C_4_), 128.6 (d, *J* = 12.5, C_3_′)^a^, ^a^ may be reversed,: δ_C_^25^ (CDCl_3_, 100 MHz) 135.2–135.4 (m, 2C), 135.1, 133.5 (dd, *J* = 101.7, 10.7), 132.0, 131.8 (d, *J* = 10.3), 131.5 (d, *J* = 104.9), 128.8 (t, *J* = 11.2), 128.4 (d, *J* = 12.6), 127.1; ^1^H NMR (CDCl_3_, 500 MHz): δ 7.96 (ddm, *J*_1_ = 12.5, *J*_2_ = 7.7, 2H), 7.69 (tt, *J*_1_ = 11.7, *J*_2_ = 1.5, 1H), 7.62 (tt, *J*_1_ = 7.7, *J*_2_ = 2.5, 1H), 7.58 (dd, *J*_1_ = 12.1, *J*_2_ = 7.9, 8H), 7.53 (t, *J* = 7.4, 4H), 7.41 (td, *J*_1_ = 7.7, *J*_2_ = 2.8, 8H),: δ_H_^25^ (CDCl_3_, 400 MHz) 7.93–7.98 (m, 2H), 7.71 (t, *J* = 11.7 Hz, 1H), 7.50–7.63 (m, 13H), 7.38–7.43 (m, 8H); [M + H]^+^ = 479.1327, C_30_H_25_O_2_P_2_ requires 479.1330.

### 4.5 Computational Details

All calculations were carried out with the Gaussian16 quantum chemistry package.^26^ Geometry optimizations in succession with frequency calculations were performed at the B3LYP/6-31G­(d,p) level of theory (for Pd atoms, the MWB28 basis set was applied), which was used in one of the earlier studies. When it was noted, the PCM model was used in order to take account of the effect of solvent media.^24,25,27,28^ For better comparison with previous results, acetonitrile was used as a model solvent (dielectric constant = 35.688).^12^

## Supplementary Material



## Notes

Copies of ^31^P, ^13^C, and ^1^H NMR spectra, as well as XYZ coordinates and total energies of the optimized structures (PDF)
