# Supplementary material for: Bromophenyl-Diphenylphosphine Oxides as Novel Starting Materials in the Suzuki–Miyaura and the Hirao Cross-Couplings, as well as in the Michaelis–Arbuzov Reaction
Source: ACS Omega. 2026 Jul 4;11(28):42772–82. doi: 10.1021/acsomega.6c04273 (PMC13393206; doi:10.1021/acsomega.6c04273)
Supplement: Supplementary file 1 [file ao6c04273_si_001.pdf]

Supporting Information

Bromophenyl-diphenylphosphine oxides as novel starting materials in the Suzuki–Miyaura- and the Hirao cross-couplings, as well as in Michaelis–Arbuzov reaction

Bianka Huszár[1], Dalma Gál,[2] Zsolt Kelemen,[2] László Drahos[3] and György Keglevich[1]*

[1] *Department of Organic Chemistry and Technology, Faculty of Chemical Technology and Biotechnology, Budapest University of Technology and Economics. 1111 Budapest, Műegyetem rkp. 3, Hungary; keglevich.gyorgy@vbk.bme.hu*

[2] *Department of Inorganic and Analytical Chemistry, Faculty of Chemical Technology and Biotechnology, Budapest University of Technology and Economics. 1111 Budapest, Műegyetem rkp. 3, Hungary*

[3] *MS Proteomics Research Group, HUN-REN Research Centre for Natural Sciences, 1117 Budapest, Hungary*

Table of contents

1. NMR spectra for groups of compounds **3A, 3B, 4A and 4B** ……………. 1–57
2. Tables containing XYZ coordinates of computed species …………...…. 58–433

1. NMR spectra for groups of compounds 3A, 3B, 4A and 4B

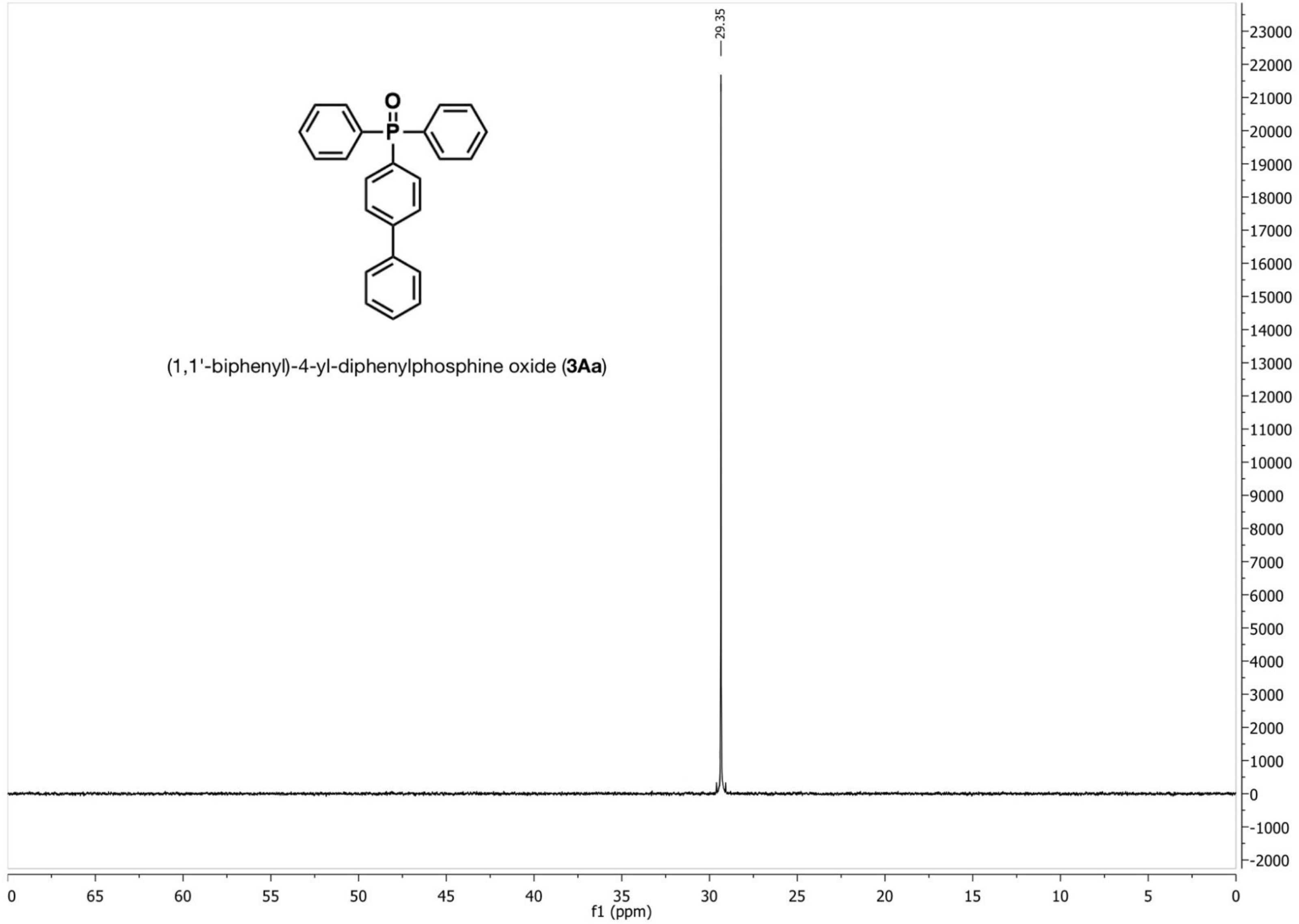

Figure S1. ^{31}P NMR ($CDCl_3$, 202.4 MHz) spectrum of (1,1'-biphenyl)-4-yl-diphenylphosphine oxide (**3Aa**).

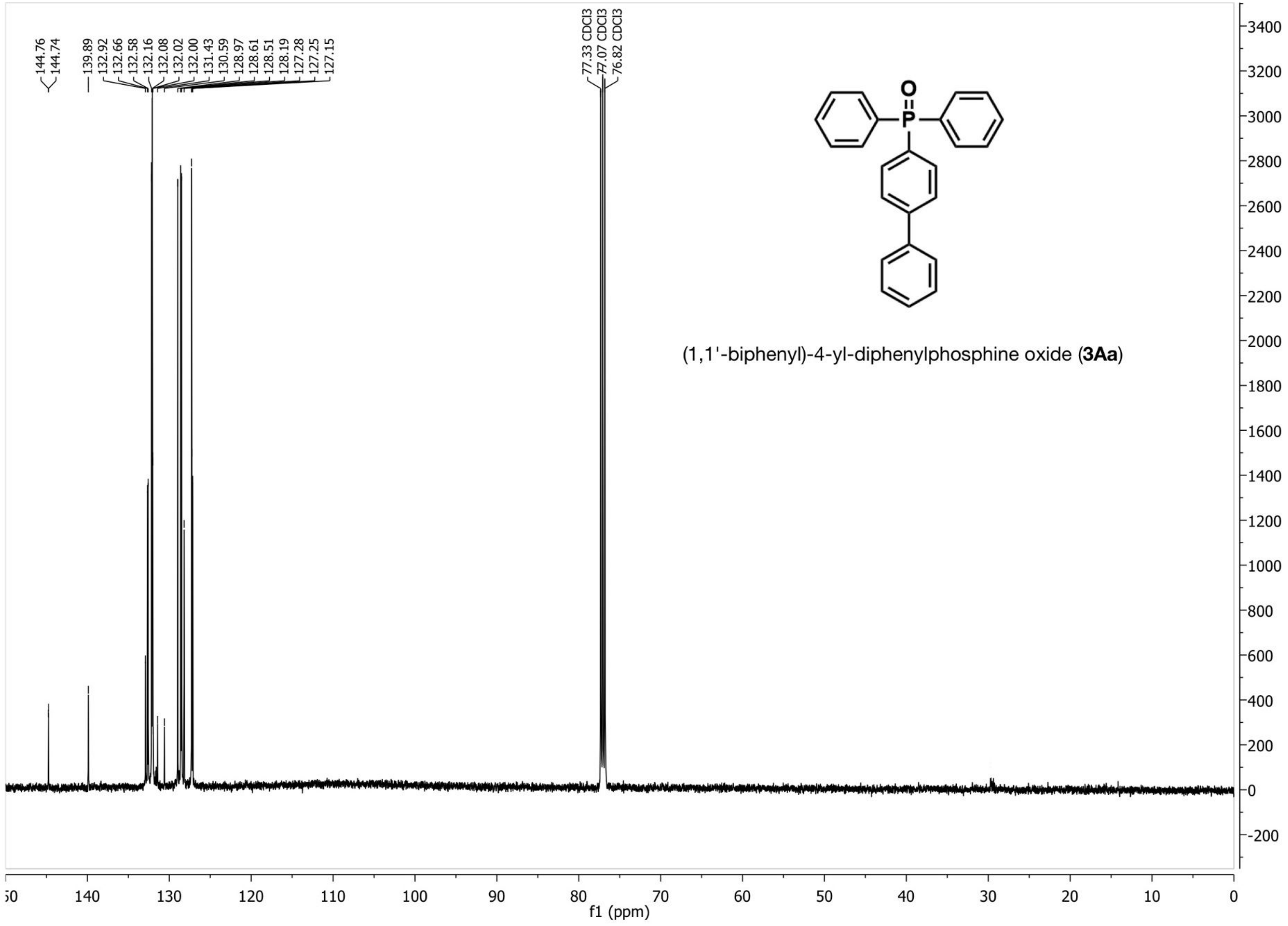

Figure S2. ^{13}C NMR ($CDCl_3$, 125.7 MHz) spectrum of (1,1'-biphenyl)-4-yl-diphenylphosphine oxide (**3Aa**).

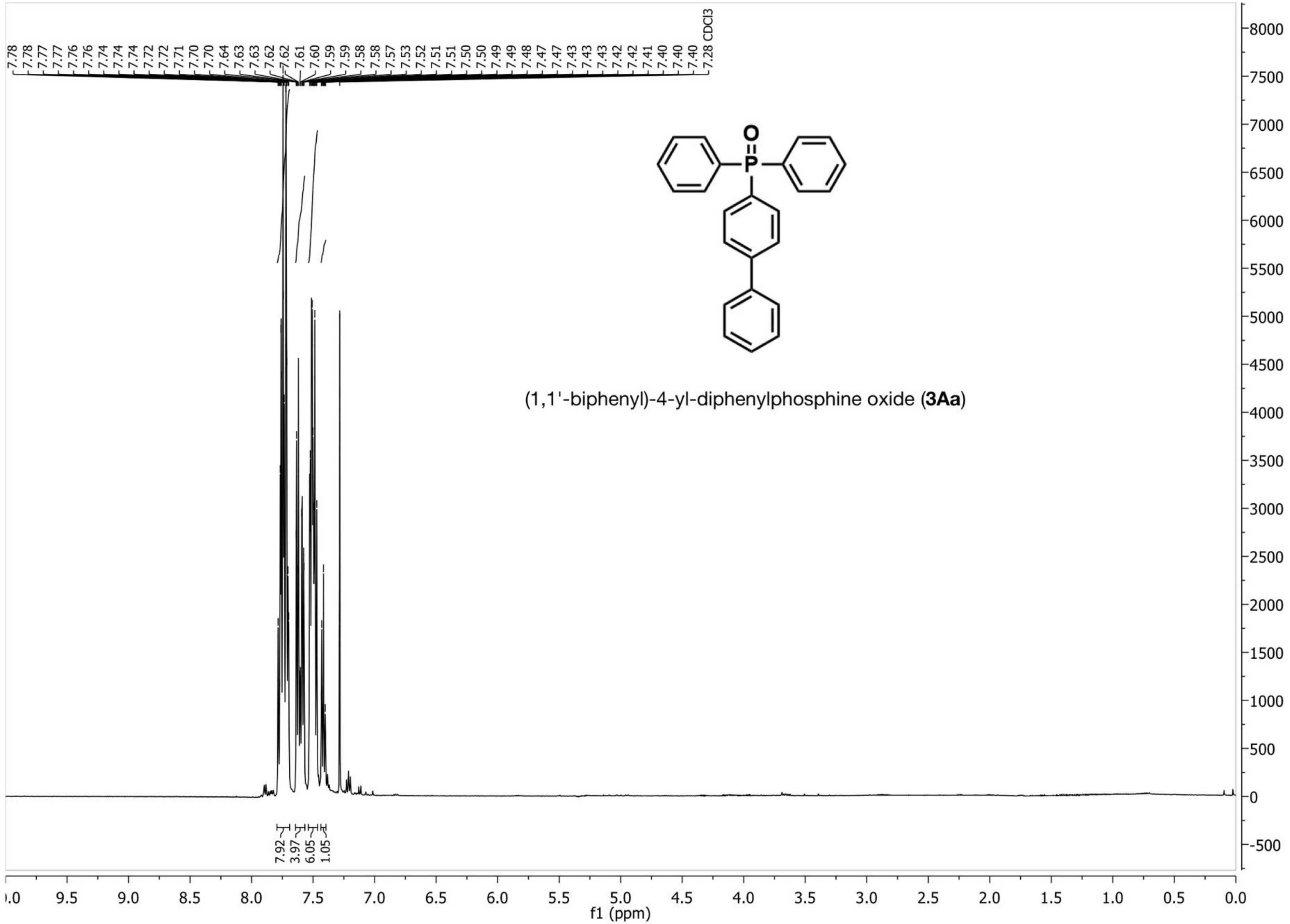

Figure S3. ^{1}H NMR ($CDCl_3$, 500 MHz) spectrum of (1,1'-biphenyl)-4-yl-diphenylphosphine oxide (**3Aa**).

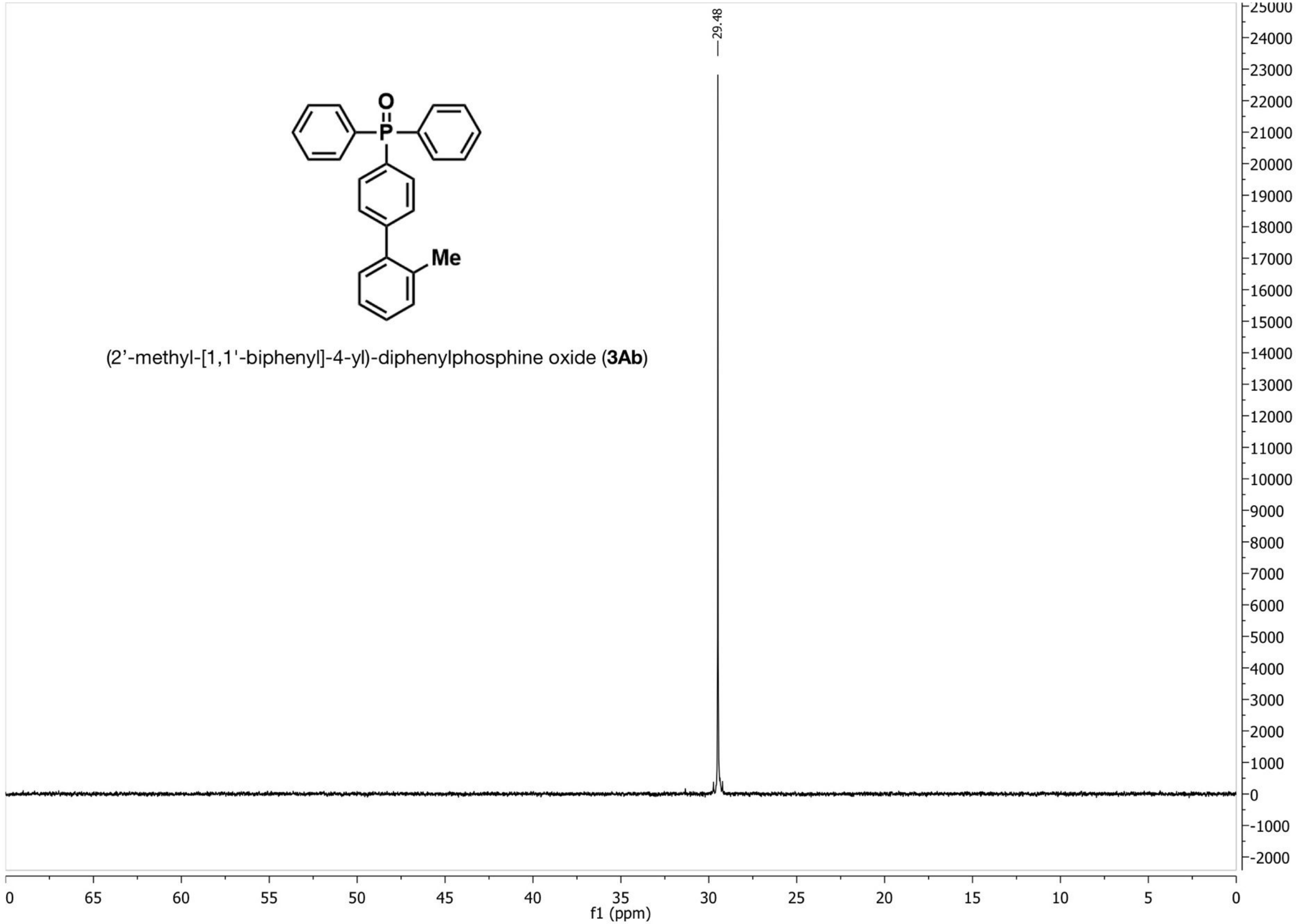

Figure S4. ^{31}P NMR ($CDCl_3$, 202.4 MHz) spectrum of (2'-methyl-[1,1'-biphenyl]-4-yl)-diphenylphosphine oxide (**3Ab**).

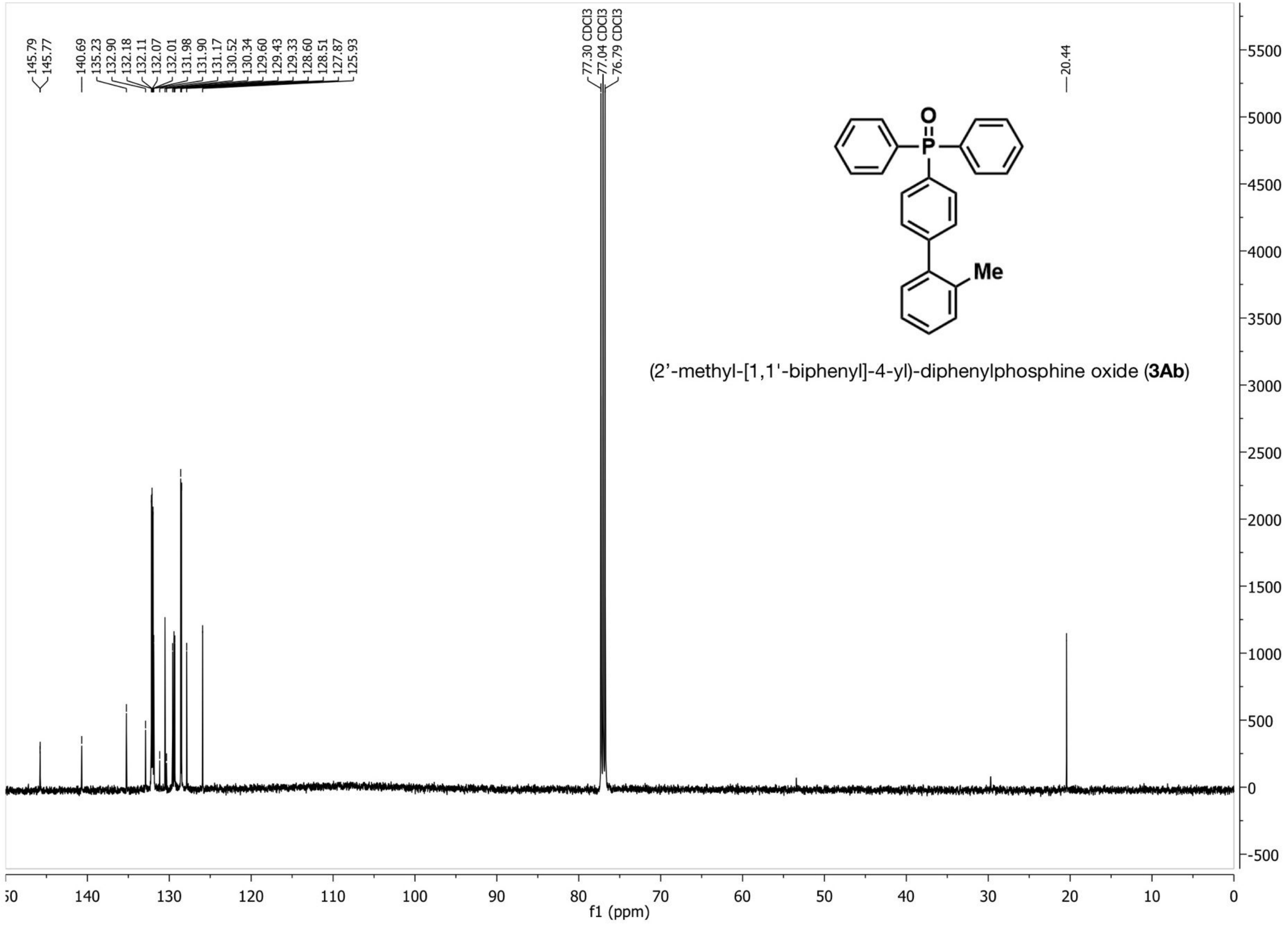

Figure S5. ^{13}C NMR ($CDCl_3$, 125.7 MHz) spectrum of (2'-methyl-[1,1'-biphenyl]-4-yl)-diphenylphosphine oxide (**3Ab**).

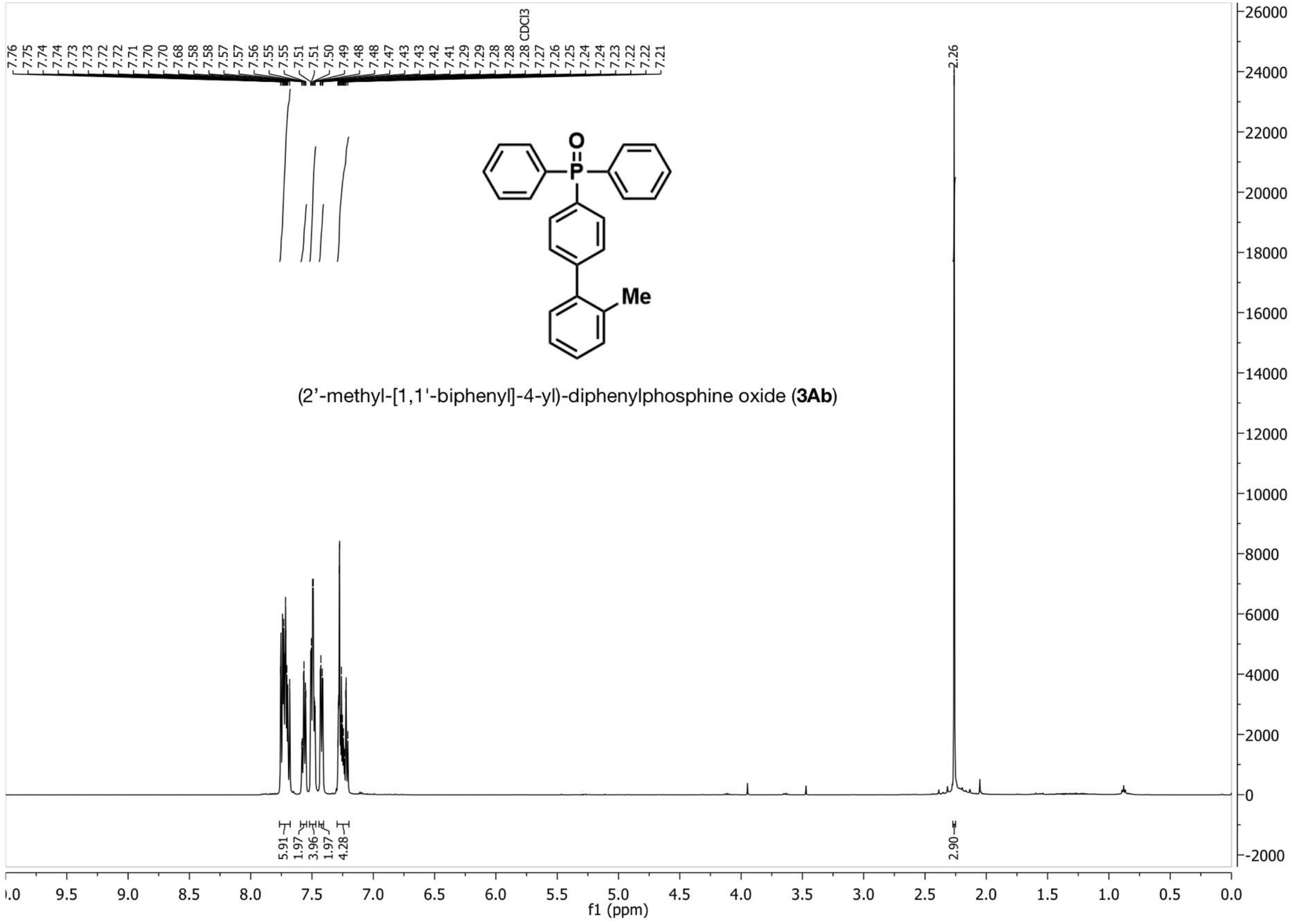

Figure S6. ^{1}H NMR ($CDCl_3$, 500 MHz) spectrum of (2'-methyl-[1,1'-biphenyl]-4-yl)-diphenylphosphine oxide (**3Ab**).

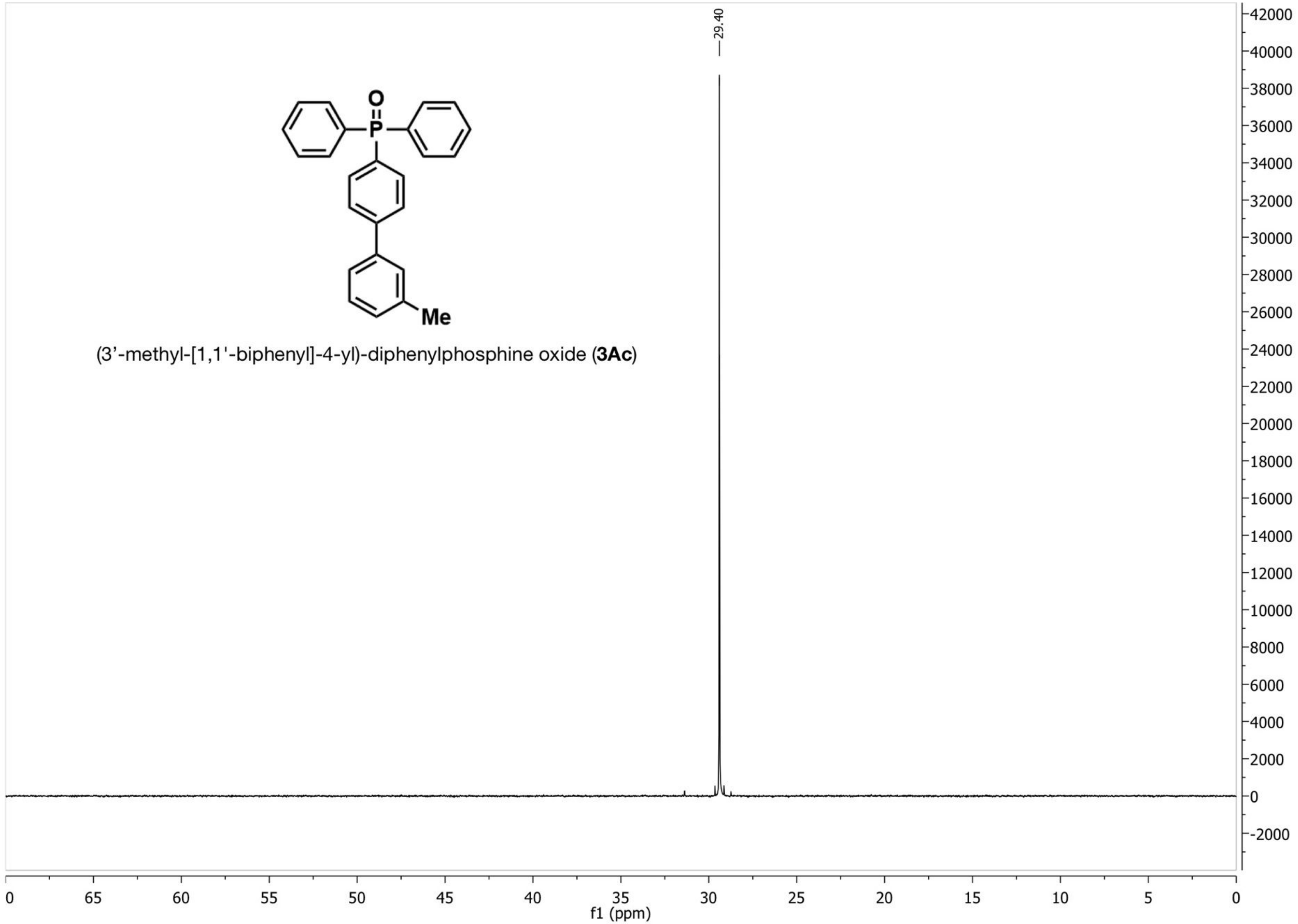

Figure S7. ^{31}P NMR ($CDCl_3$, 202.4 MHz) spectrum of (3'-methyl-[1,1'-biphenyl]-4-yl)-diphenylphosphine oxide (**3Ac**).

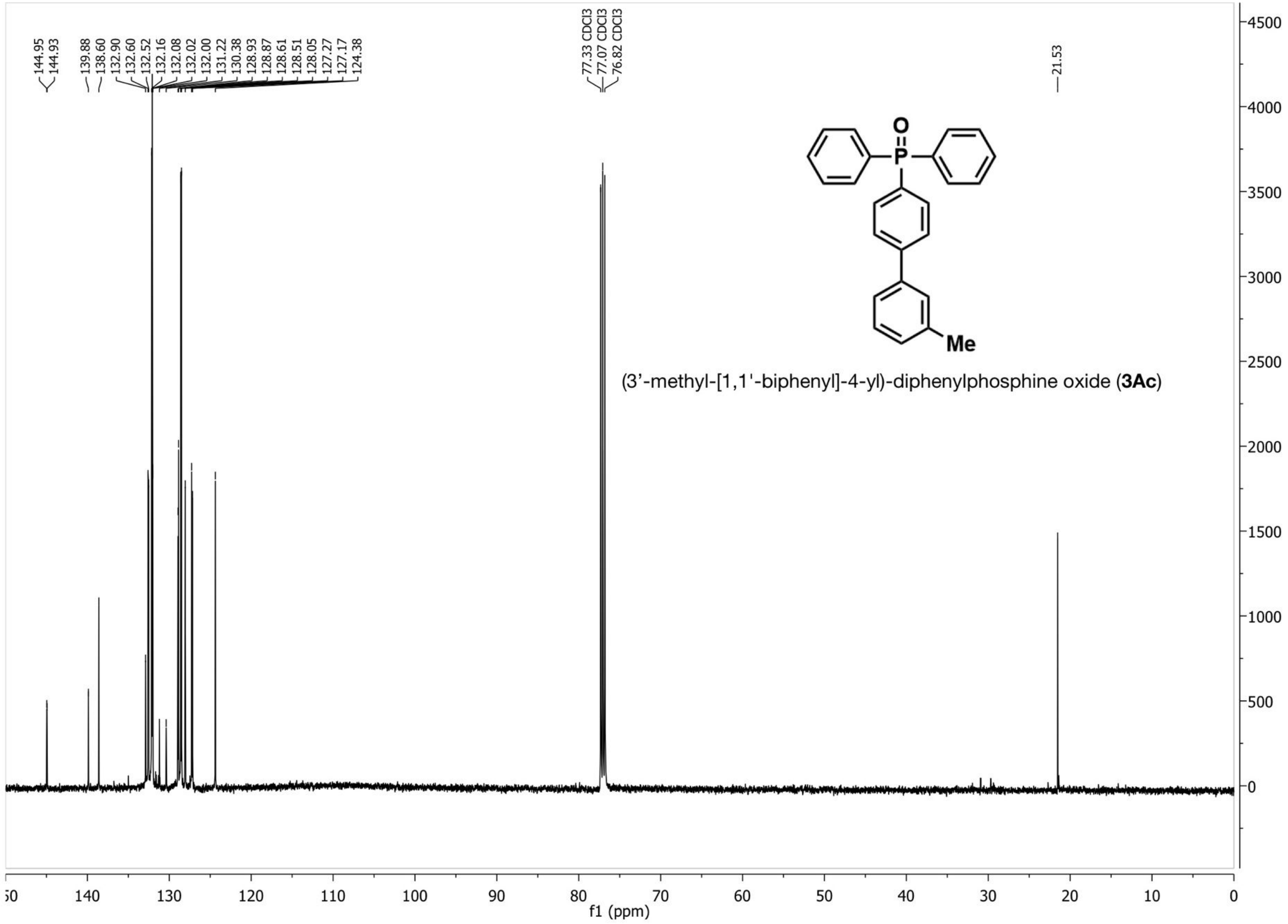

Figure S8. ^{13}C NMR spectrum ($CDCl_3$, 125.7 MHz) of (3'-methyl-[1,1'-biphenyl]-4-yl)-diphenylphosphine oxide (**3Ac**).

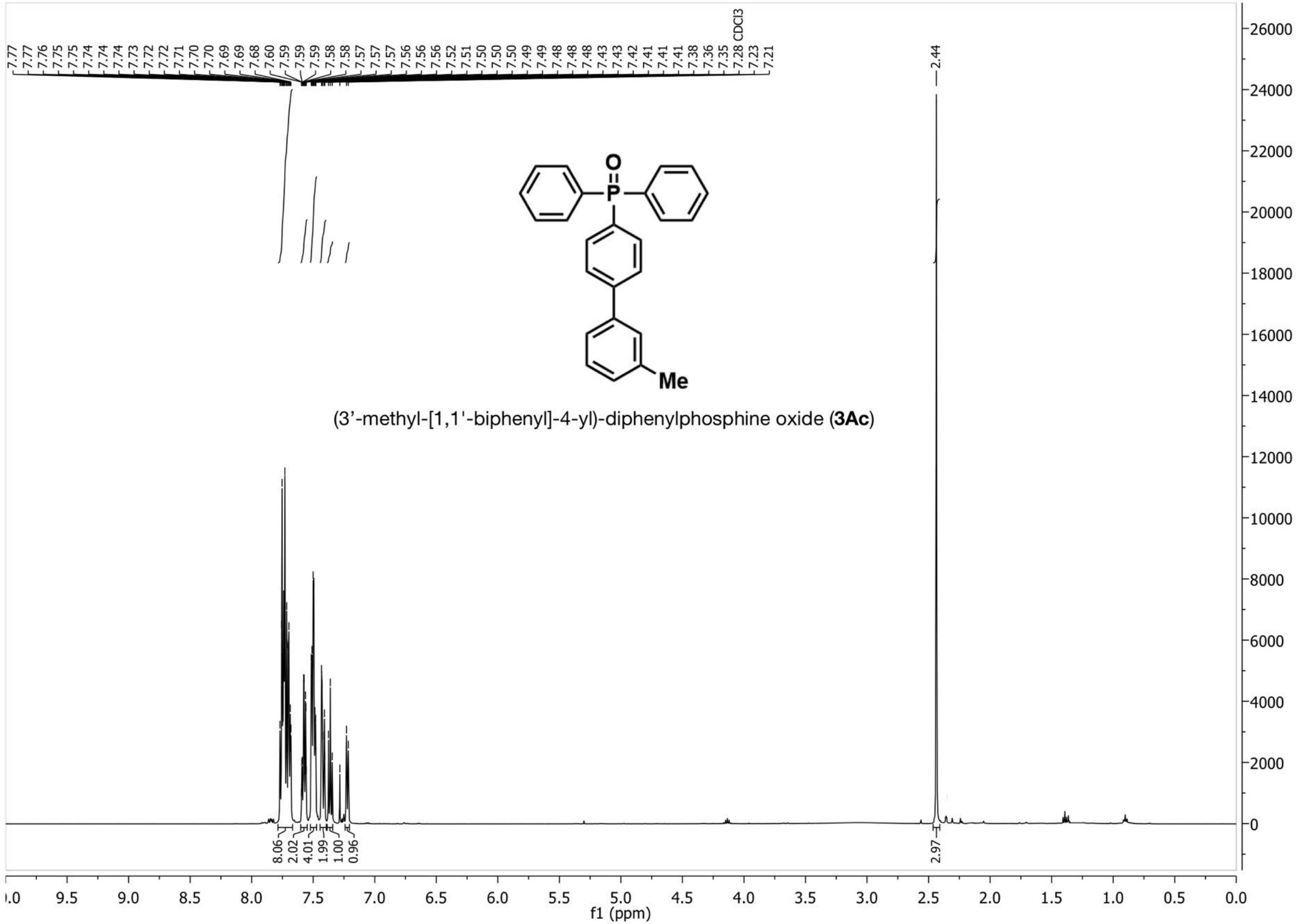

Figure S9. ^{1}H NMR ($CDCl_3$, 500 MHz) spectrum of (3'-methyl-[1,1'-biphenyl]-4-yl)-diphenylphosphine oxide (**3Ac**).

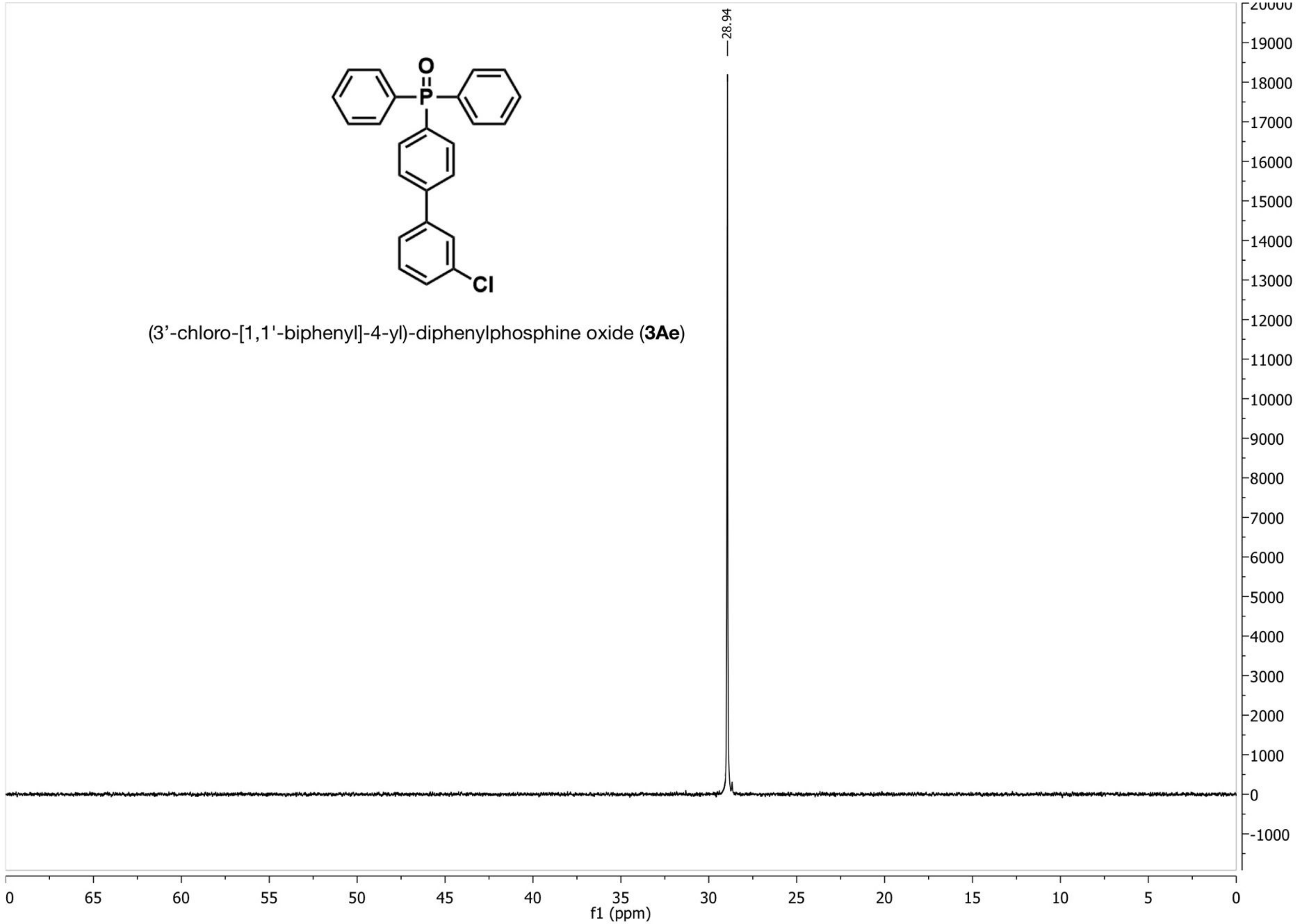

Figure S10. ^{31}P NMR ($CDCl_3$, 202.4 MHz) spectrum of (3'-chloro-[1,1'-biphenyl]-4-yl)-diphenylphosphine oxide (**3Ae**).

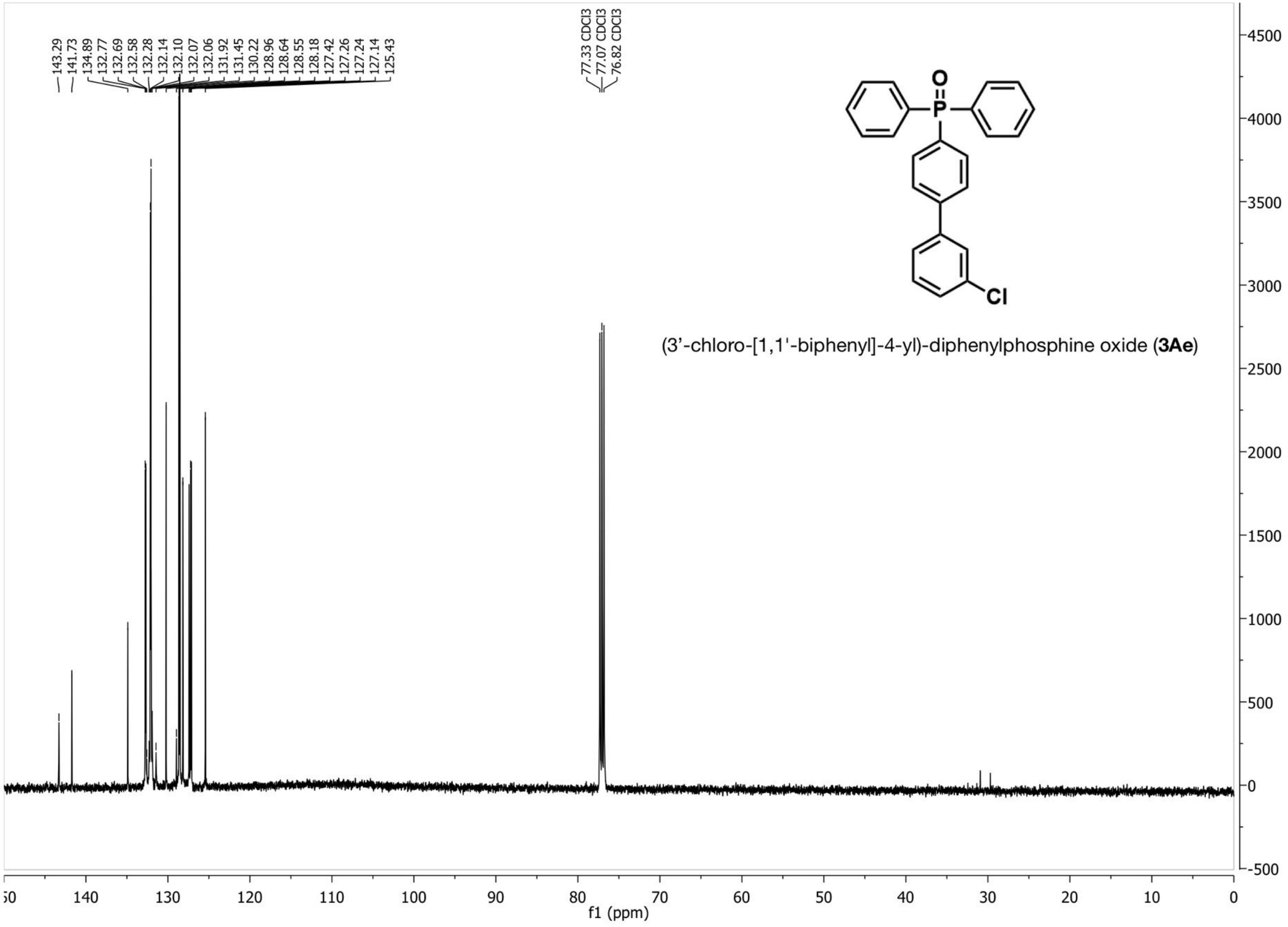

Figure S11. ^{13}C NMR ($CDCl_3$, 125.7 MHz) spectrum of (3'-chloro-[1,1'-biphenyl]-4-yl)-diphenylphosphine oxide (**3Ae**).

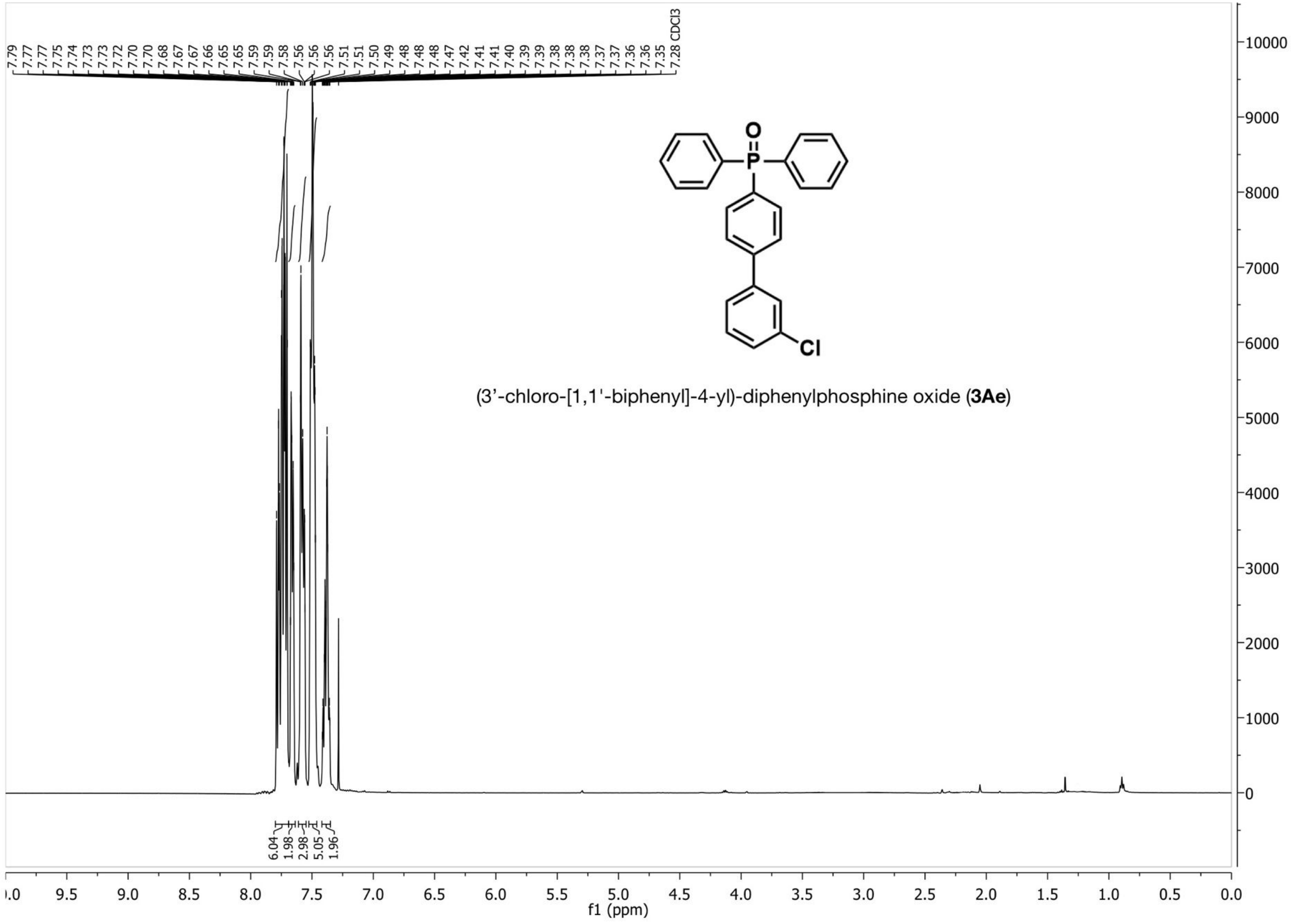

Figure S12. ^{1}H NMR ($CDCl_3$, 500 MHz) spectrum of (3'-chloro-[1,1'-biphenyl]-4-yl)-diphenylphosphine oxide (**3Ae**).

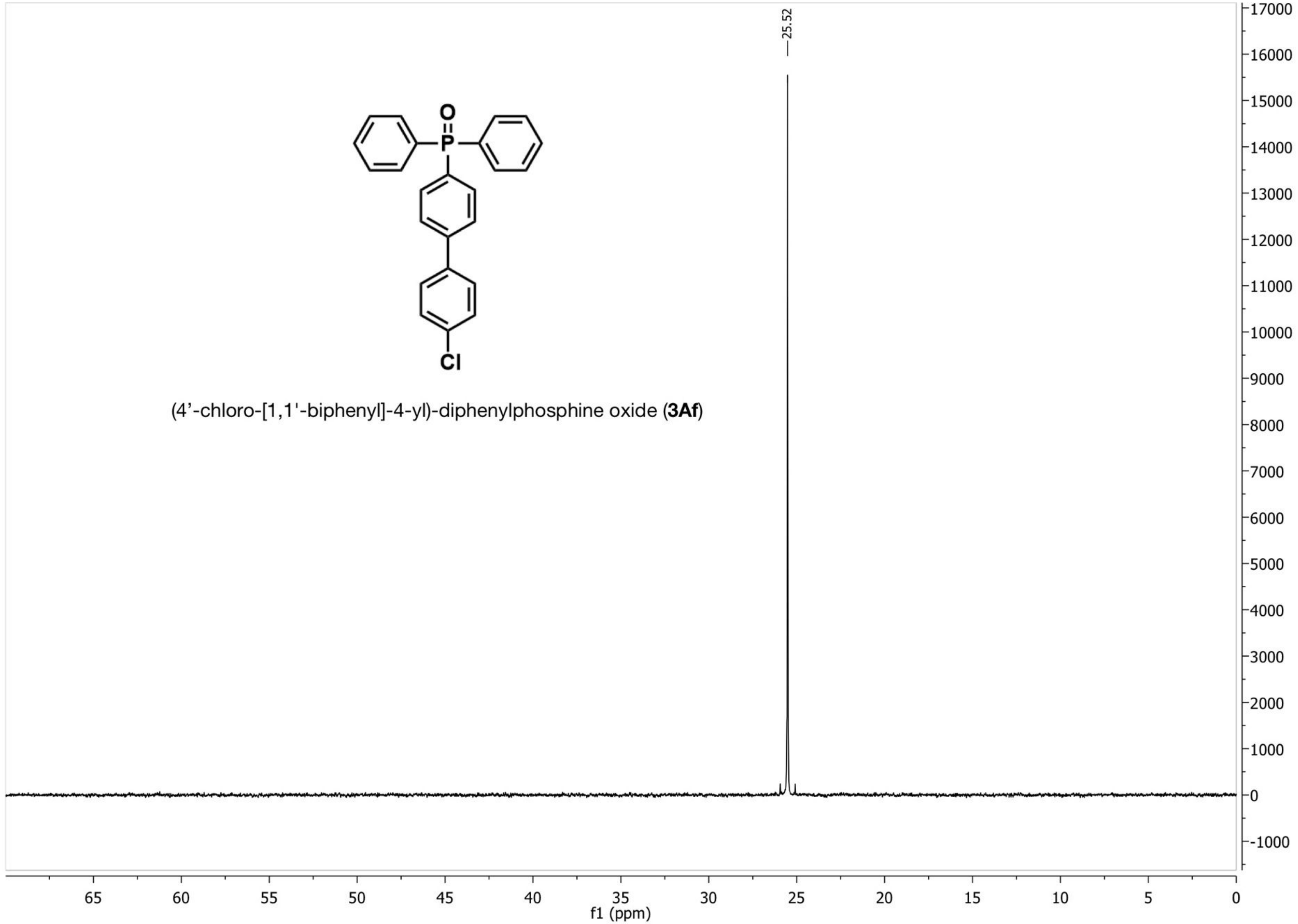

Figure S13. ^{31}P NMR (DMSO, 121.5 MHz) spectrum of (4'-chloro-[1,1'-biphenyl]-4-yl)-diphenylphosphine oxide (**3Af**).

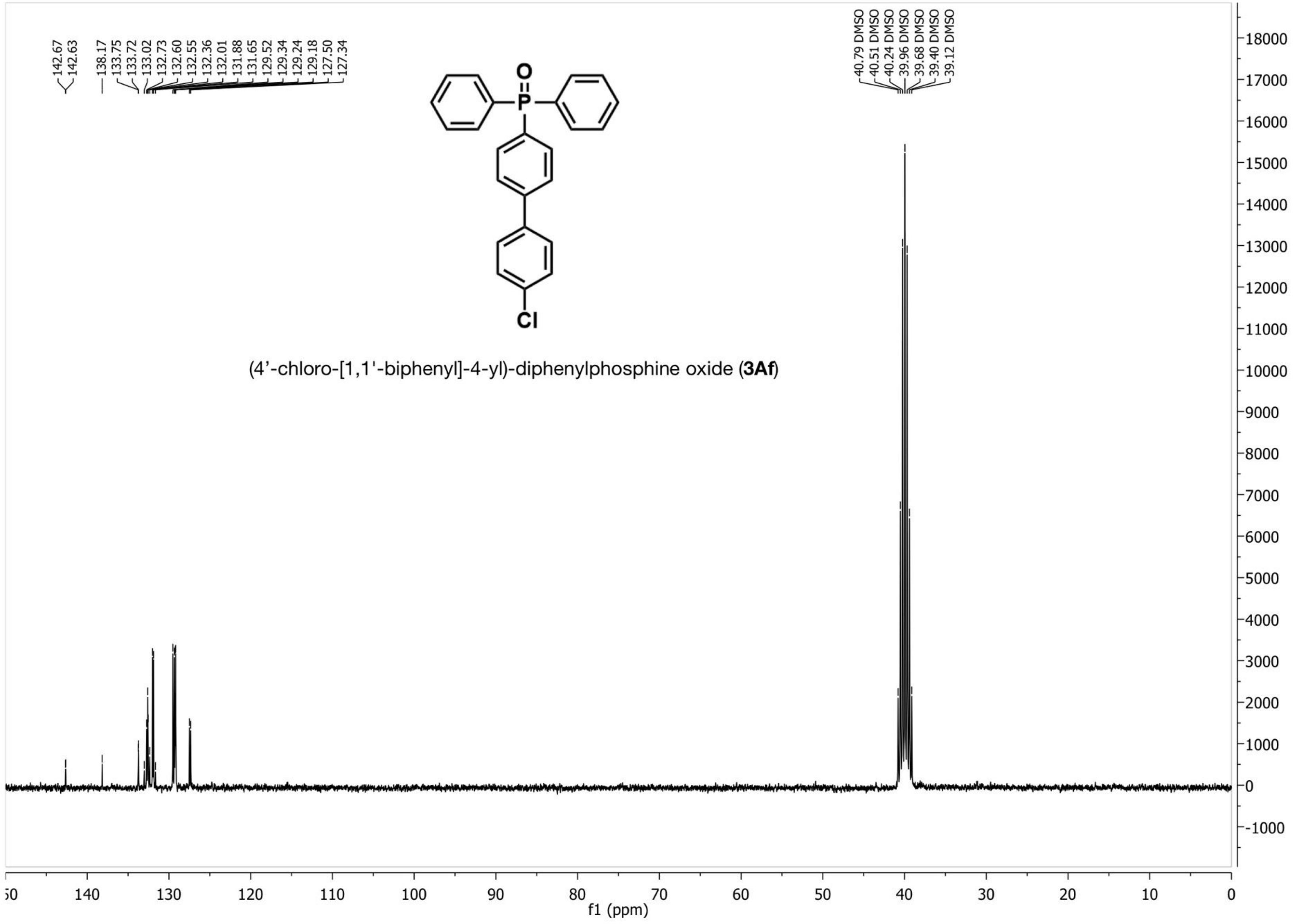

Figure S14. ^{13}C NMR (DMSO, 75.4 MHz) spectrum of (4'-chloro-[1,1'-biphenyl]-4-yl)-diphenylphosphine oxide (**3Af**).

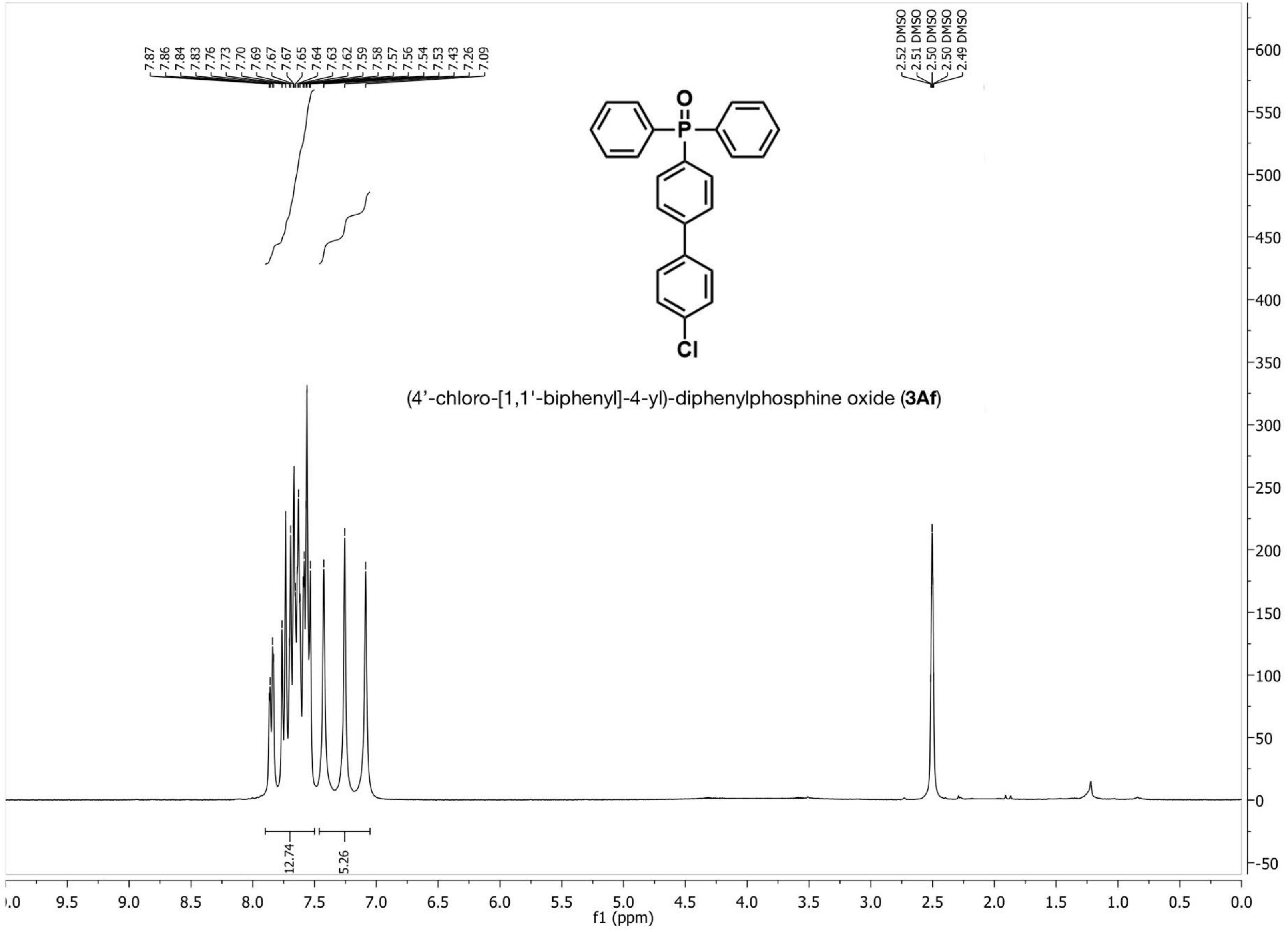

Figure S15. ^{1}H NMR (DMSO, 300 MHz) spectrum of (4'-chloro-[1,1'-biphenyl]-4-yl)-diphenylphosphine oxide (**3Af**).

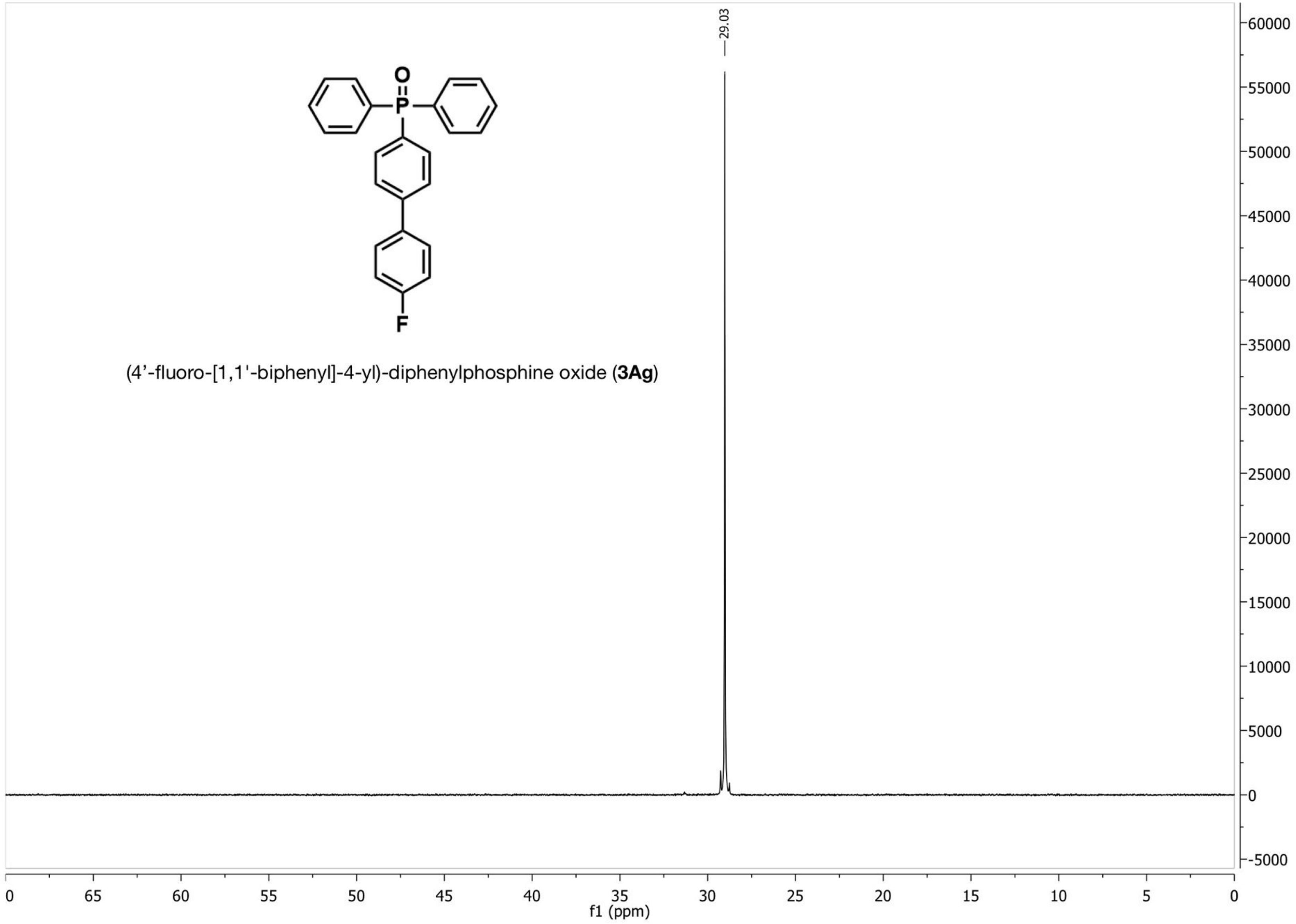

Figure S16. ^{31}P NMR ($CDCl_3$, 202.4 MHz) spectrum of (4'-fluoro-[1,1'-biphenyl]-4-yl)-diphenylphosphine oxide (**3Ag**).

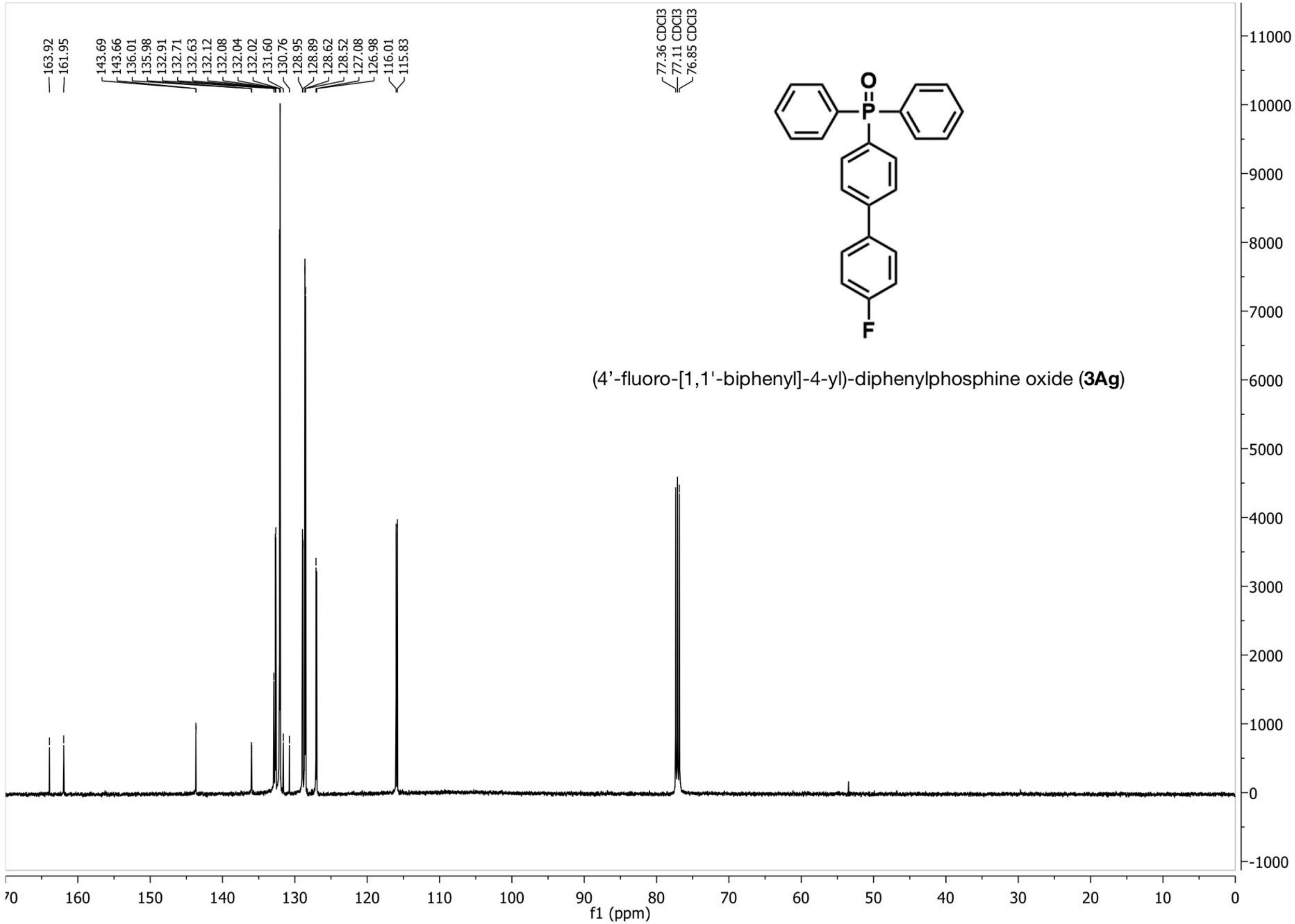

Figure S17. ^{13}C NMR ($CDCl_3$, 125.7 MHz) spectrum of (4'-fluoro-[1,1'-biphenyl]-4-yl)-diphenylphosphine oxide (**3Ag**).

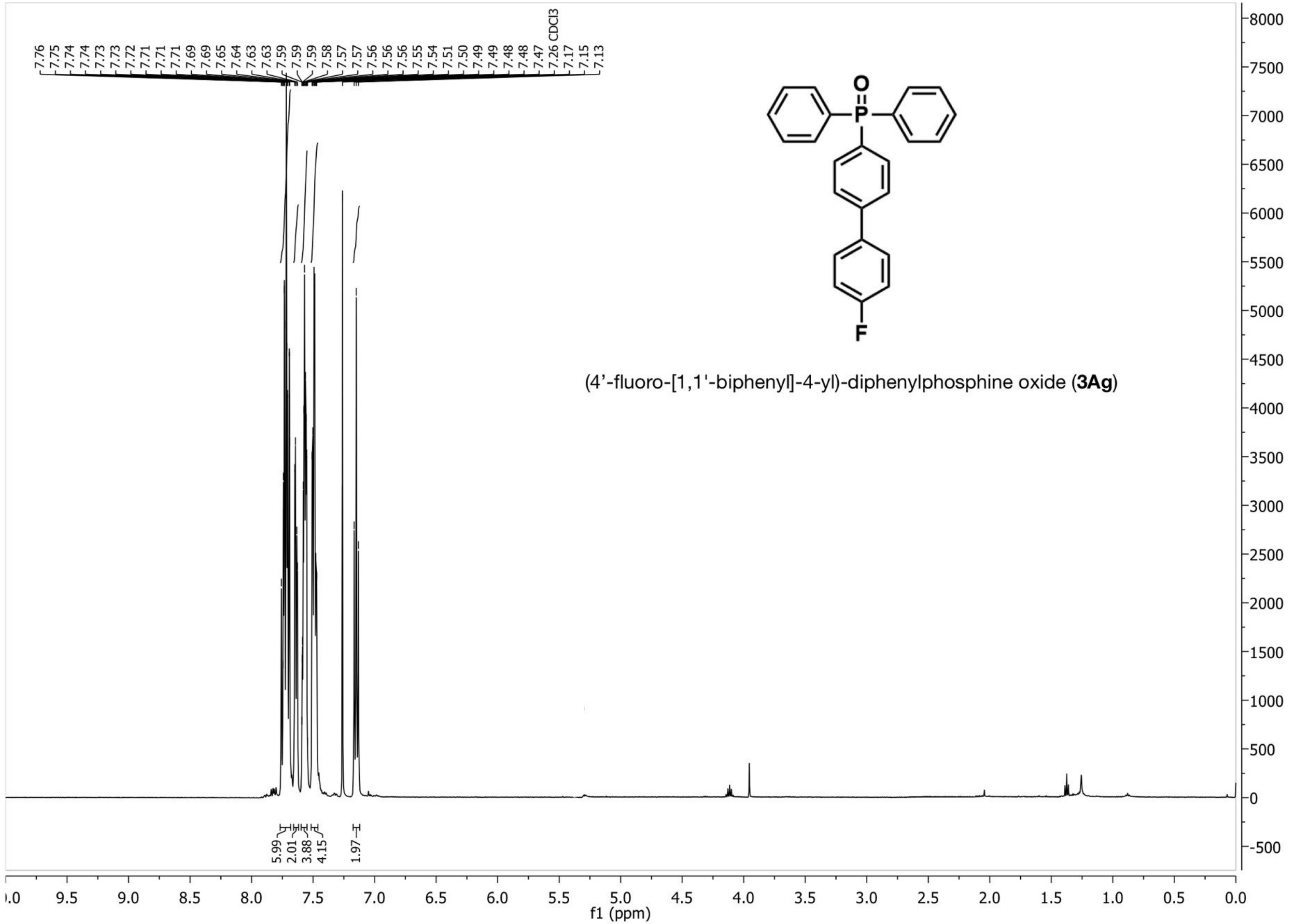

Figure S18. ^{1}H NMR ($CDCl_3$, 500 MHz) spectrum of (4'-fluoro-[1,1'-biphenyl]-4-yl)-diphenylphosphine oxide (**3Ag**).

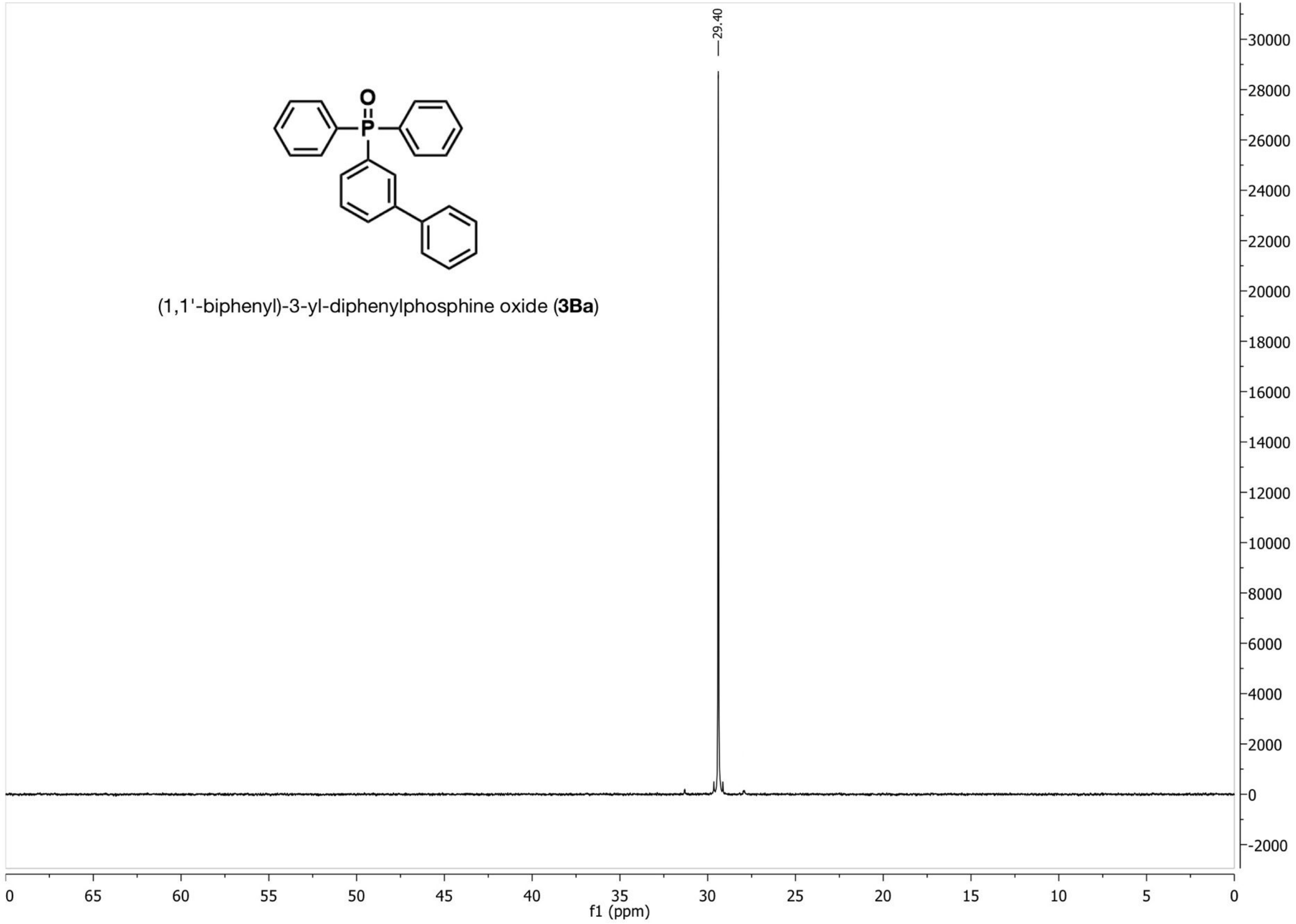

Figure S19. ^{31}P NMR ($CDCl_3$, 202.4 MHz) spectrum of (1,1'-biphenyl)-3-yl-diphenylphosphine oxide (**3Ba**).

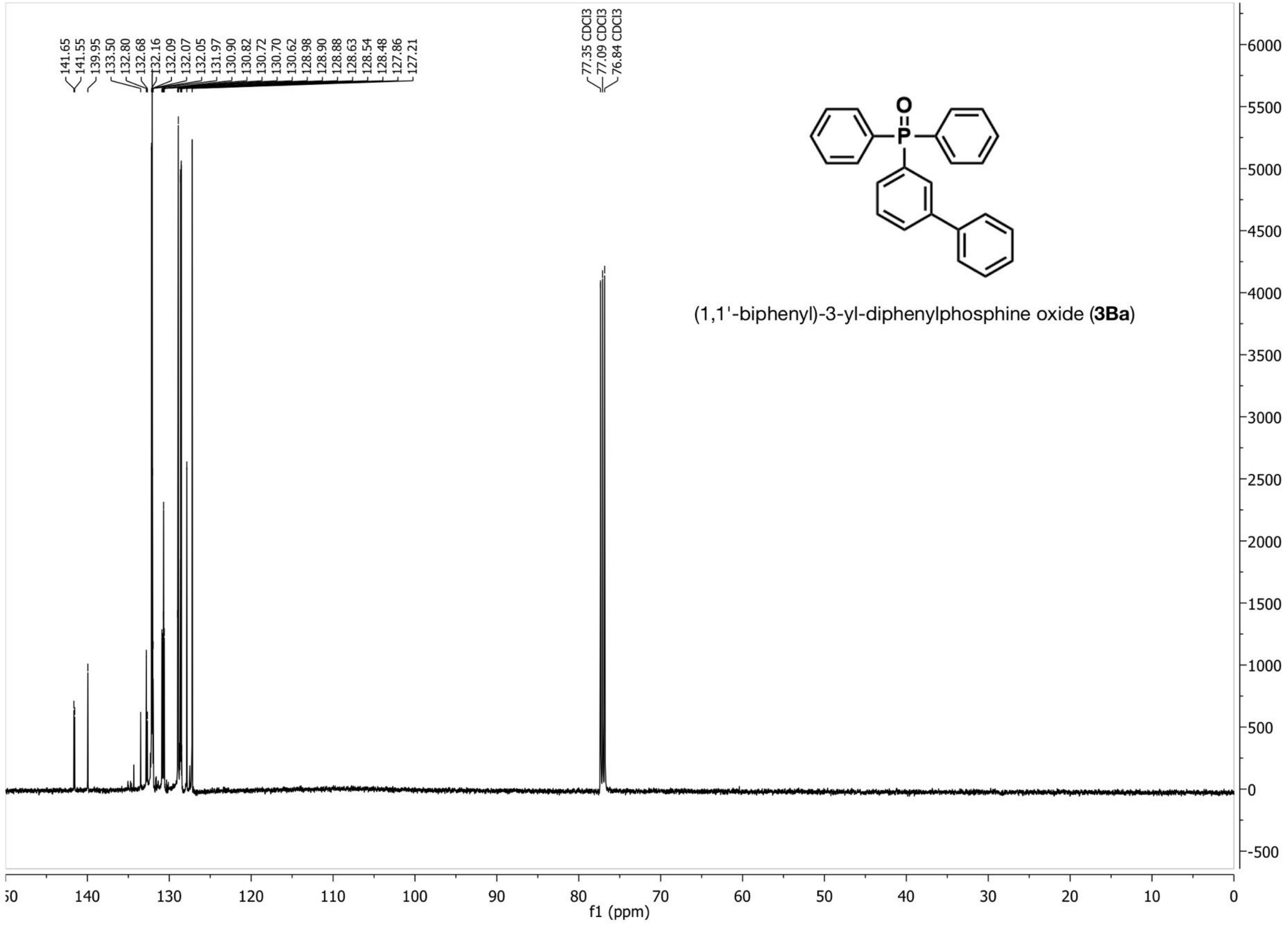

Figure S20. ^{13}C NMR ($CDCl_3$, 125.7 MHz) spectrum of (1,1'-biphenyl)-3-yl-diphenylphosphine oxide (**3Ba**).

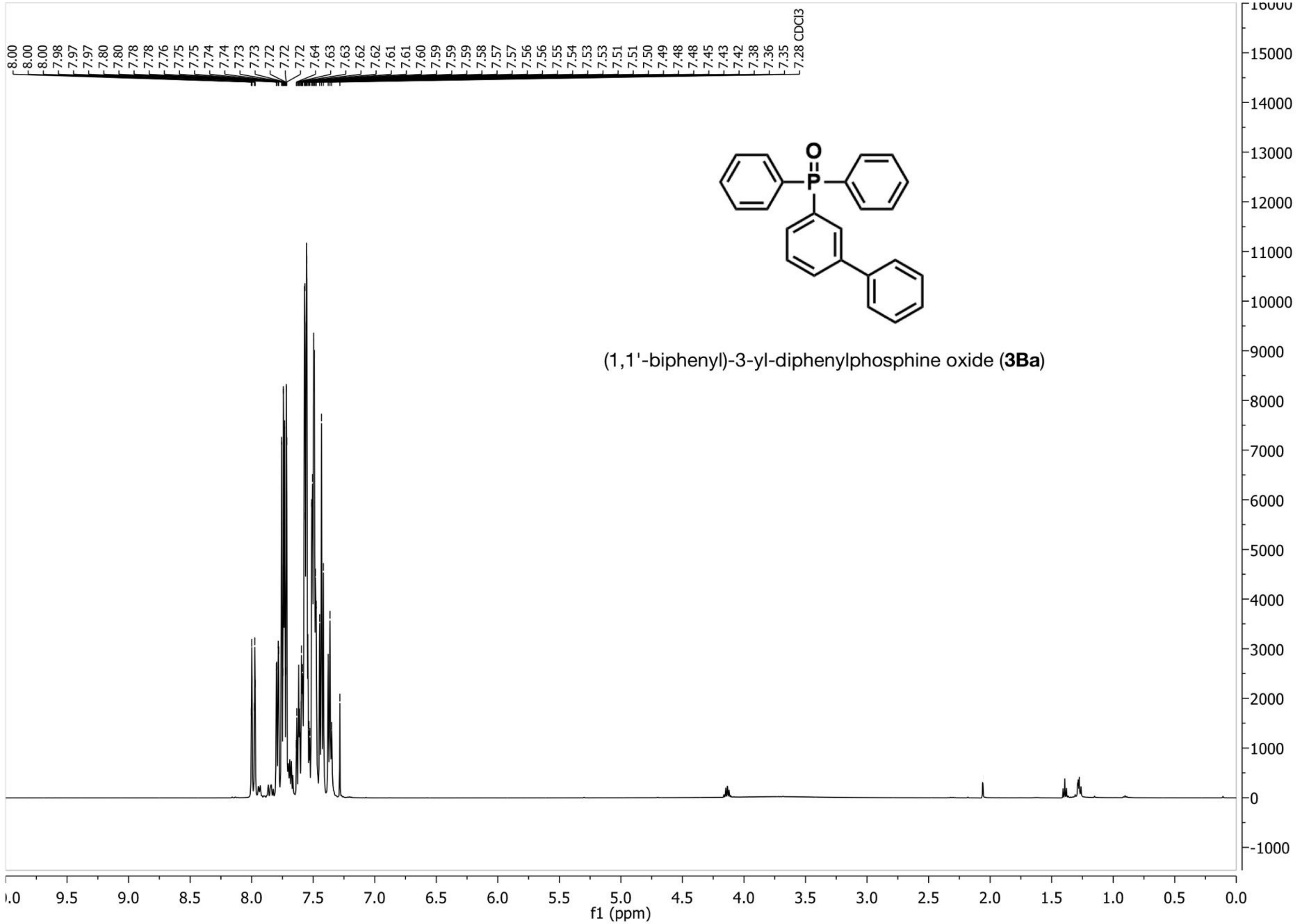

Figure S21. ^{1}H NMR ($CDCl_3$, 500 MHz) spectrum of (1,1'-biphenyl)-3-yl-diphenylphosphine oxide (**3Ba**).

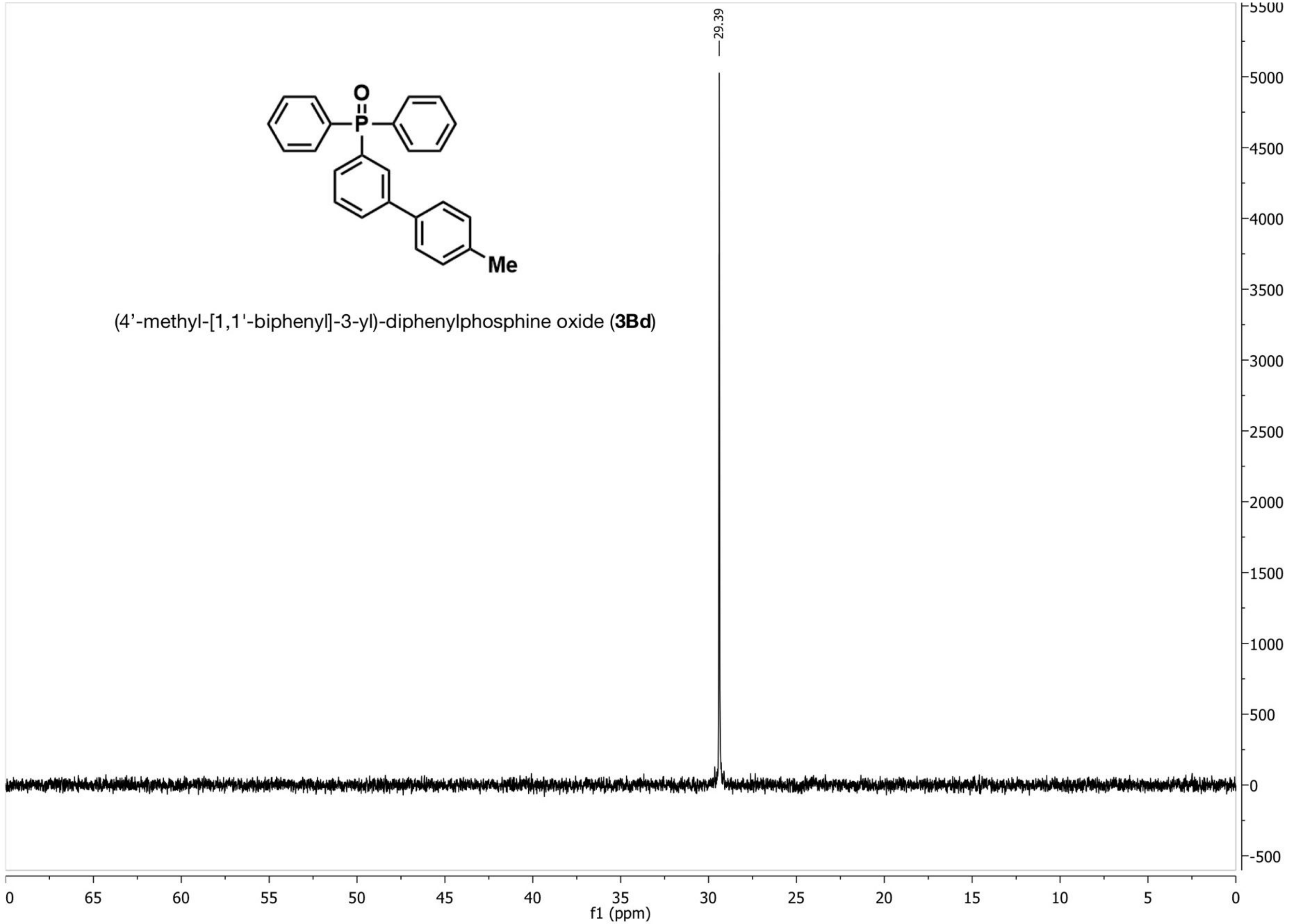

Figure S22. ^{31}P NMR ($CDCl_3$, 202.4 MHz) spectrum of (4'-methyl-[1,1'-biphenyl]-3-yl)-diphenylphosphine oxide (**3Bd**).

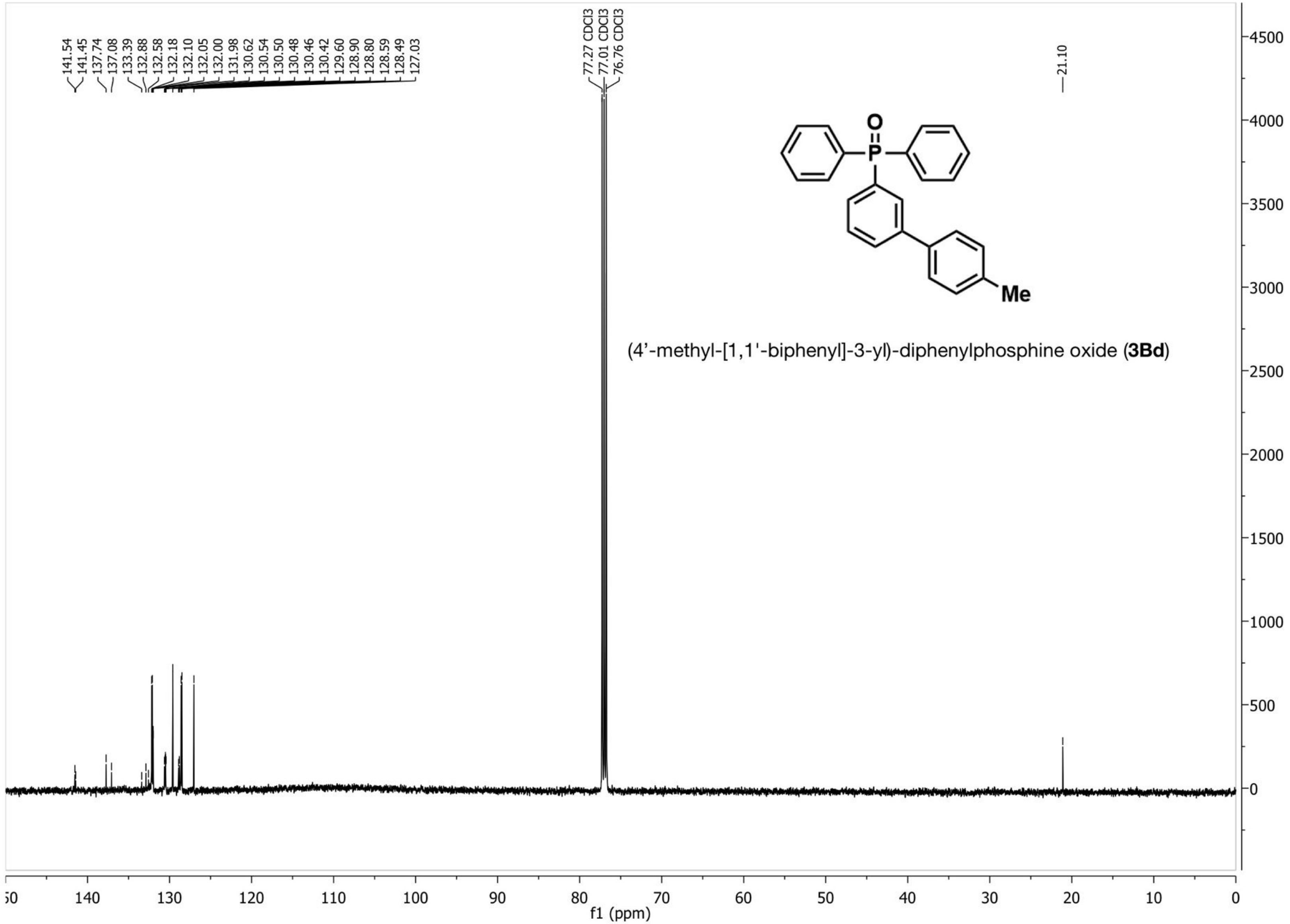

Figure S23. ^{13}C NMR ($CDCl_3$, 125.7 MHz) spectrum of (4'-methyl-[1,1'-biphenyl]-3-yl)-diphenylphosphine oxide (**3Bd**).

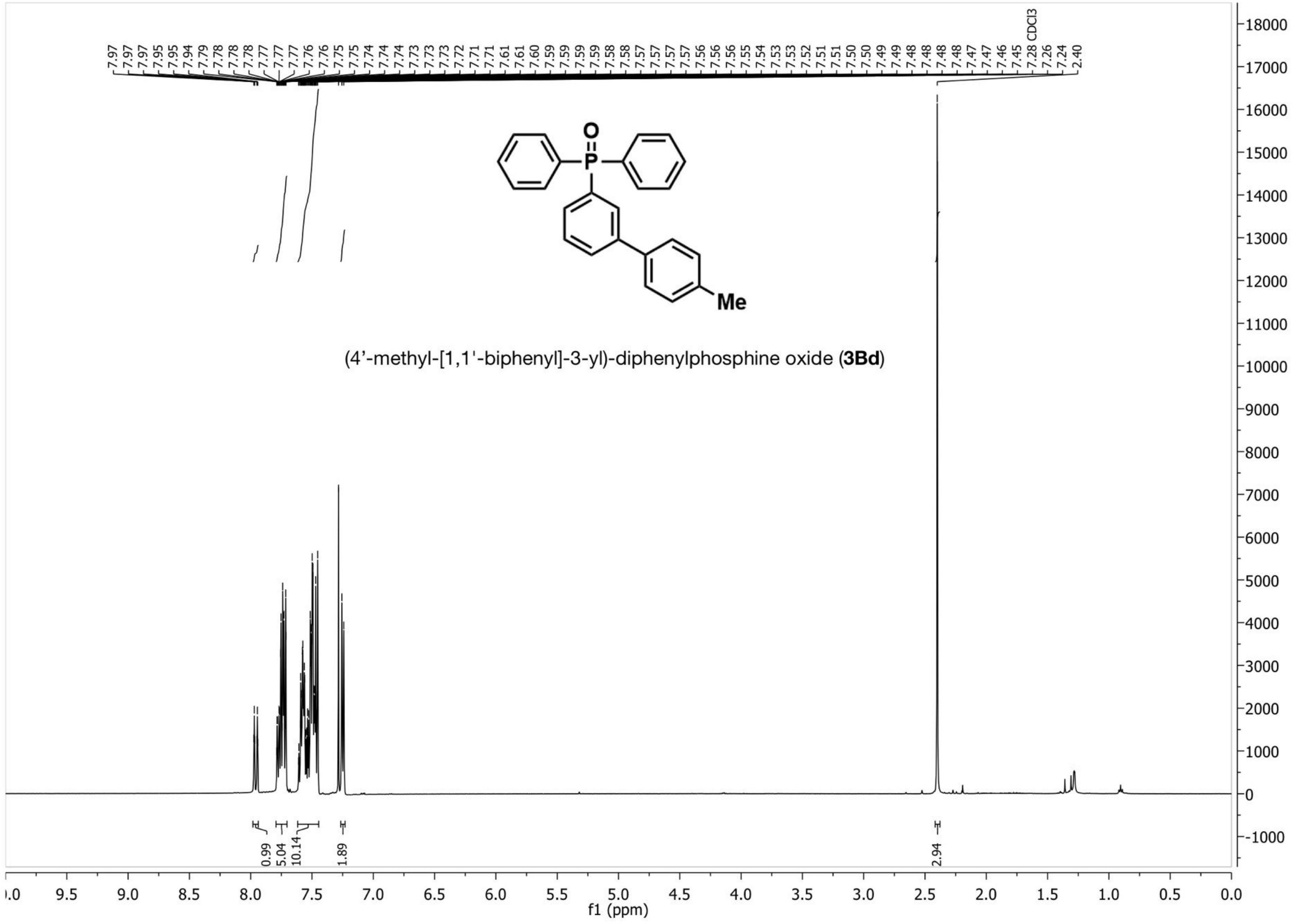

Figure S24. ^{1}H NMR ($CDCl_3$, 500 MHz) spectrum of (4'-methyl-[1,1'-biphenyl]-3-yl)-diphenylphosphine oxide (**3Bd**).

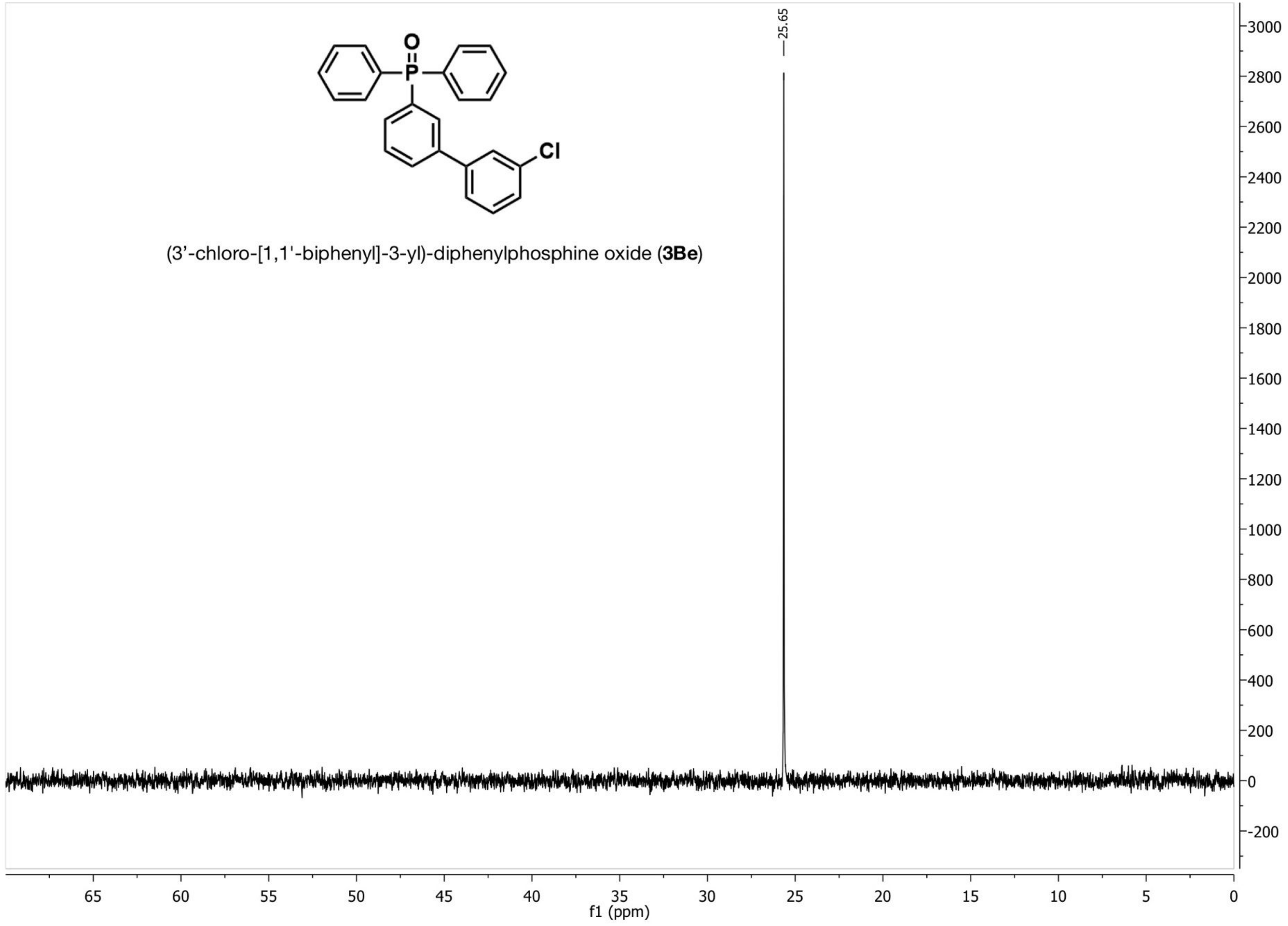

Figure S25. ^{31}P NMR (DMSO, 121.5 MHz) spectrum of (3'-chloro-[1,1'-biphenyl]-3-yl)-diphenylphosphine oxide (**3Be**).

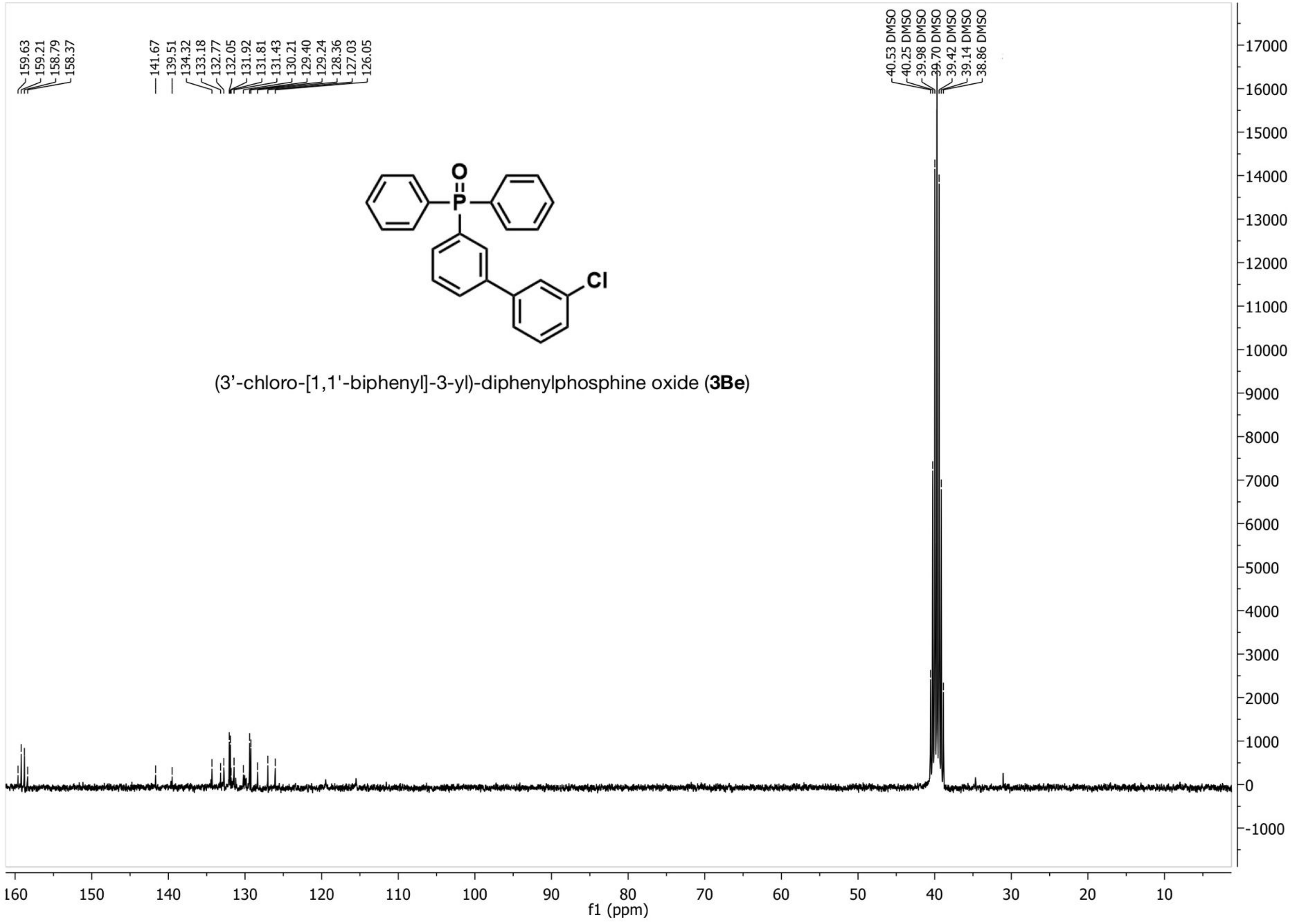

Figure S26. ^{13}C NMR (DMSO, 75.4 MHz) spectrum of (3'-chloro-[1,1'-biphenyl]-3-yl)-diphenylphosphine oxide (**3Be**).

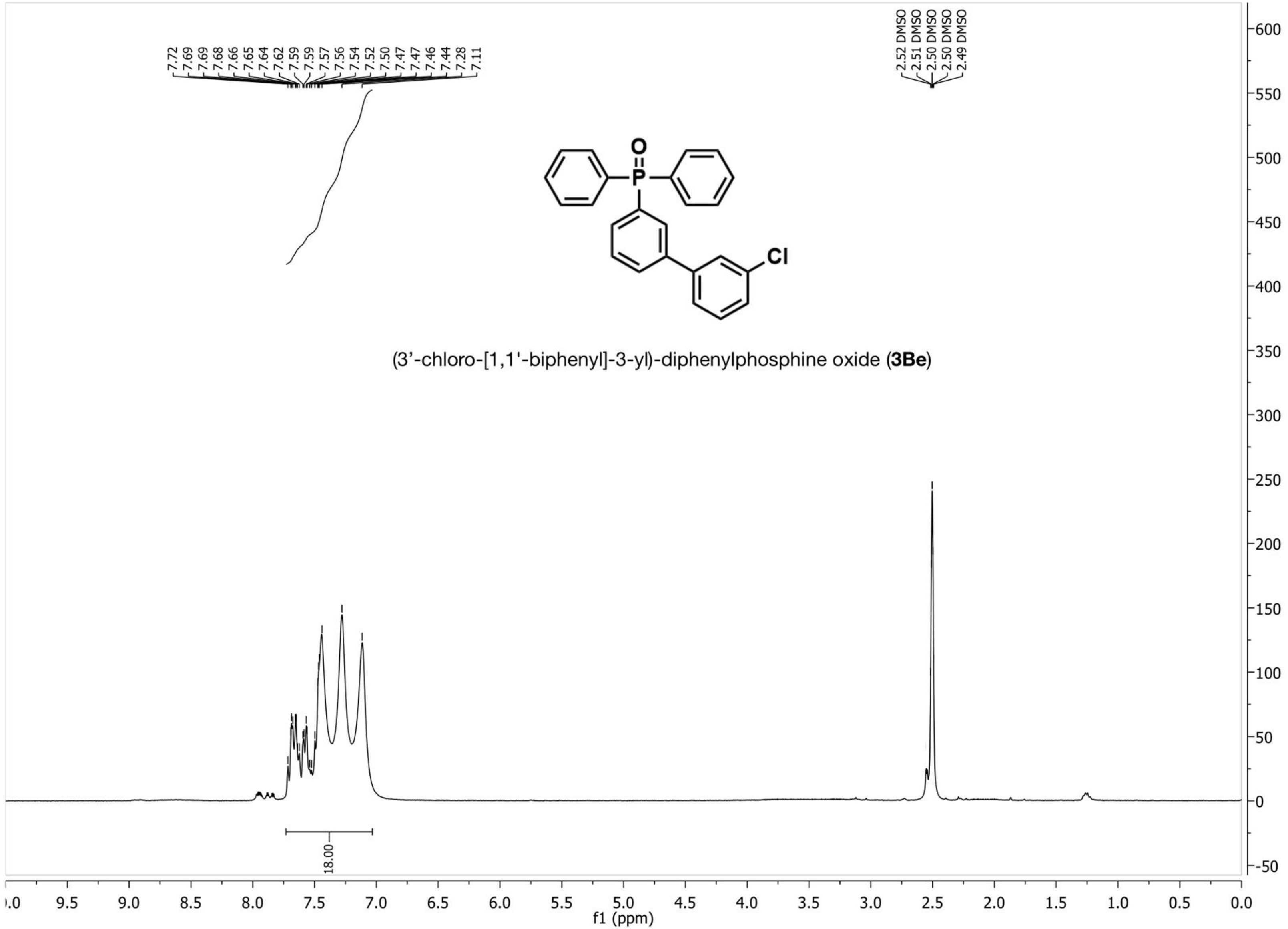

Figure S27. ^{1}H NMR (DMSO, 300 MHz) spectrum of (3'-chloro-[1,1'-biphenyl]-3-yl)-diphenylphosphine oxide (**3Be**).

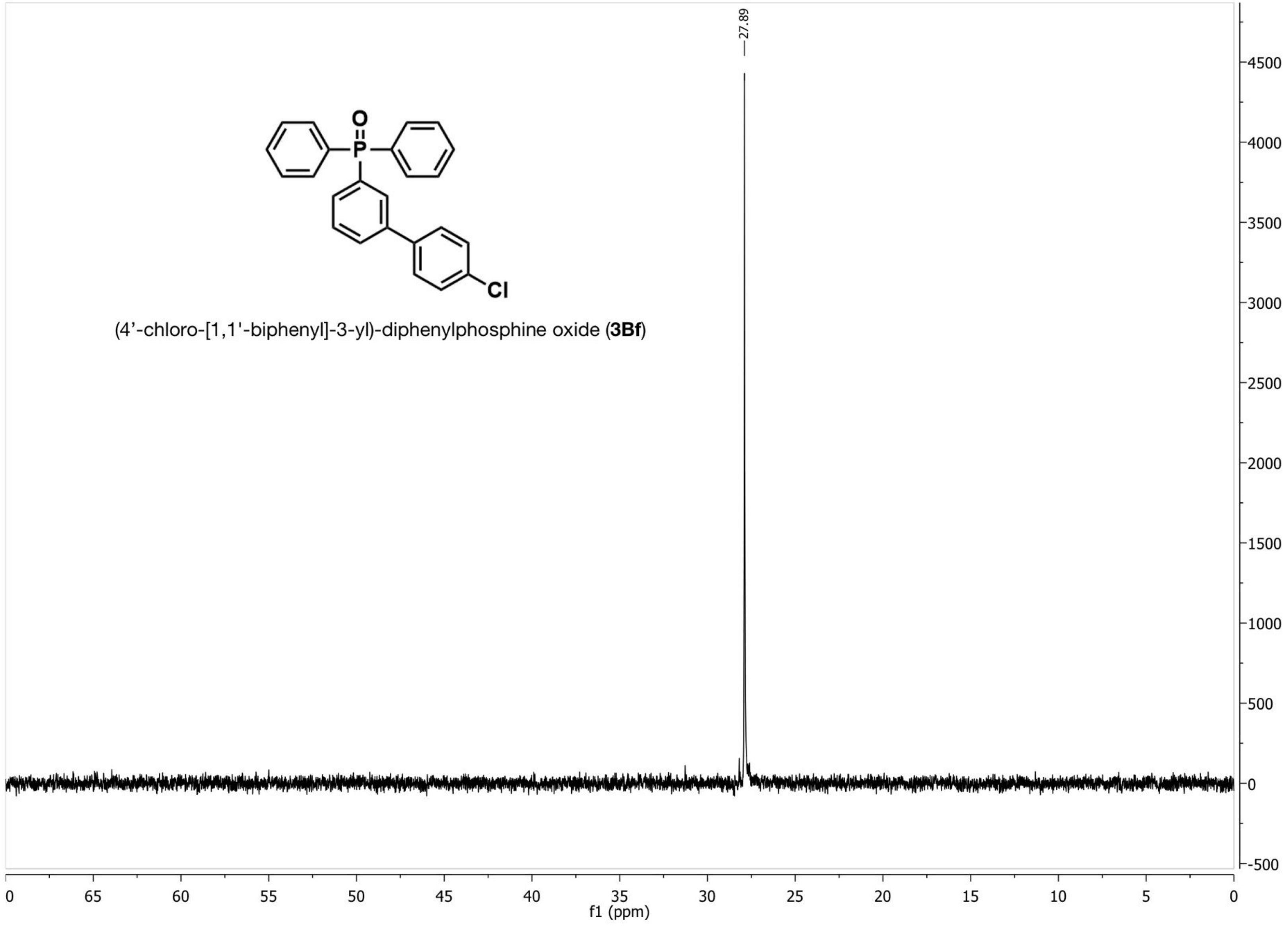

Figure S28. ^{31}P NMR ($CDCl_3$, 202.4 MHz) spectrum of (4'-chloro-[1,1'-biphenyl]-3-yl)-diphenylphosphine oxide (**3Bf**).

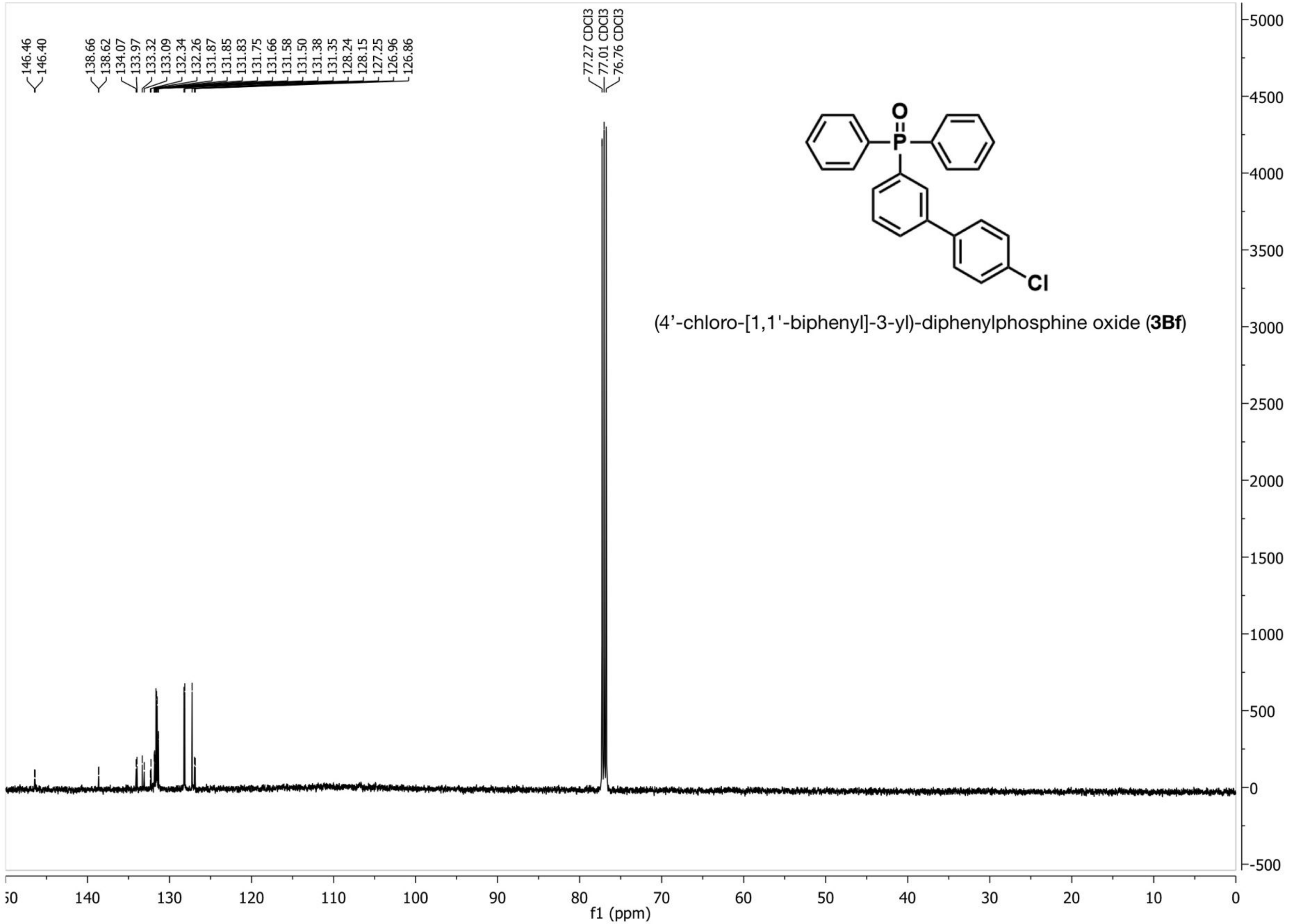

Figure S29. ^{13}C NMR ($CDCl_3$, 125.7 MHz) spectrum of (4'-chloro-[1,1'-biphenyl]-3-yl)-diphenylphosphine oxide (**3Bf**).

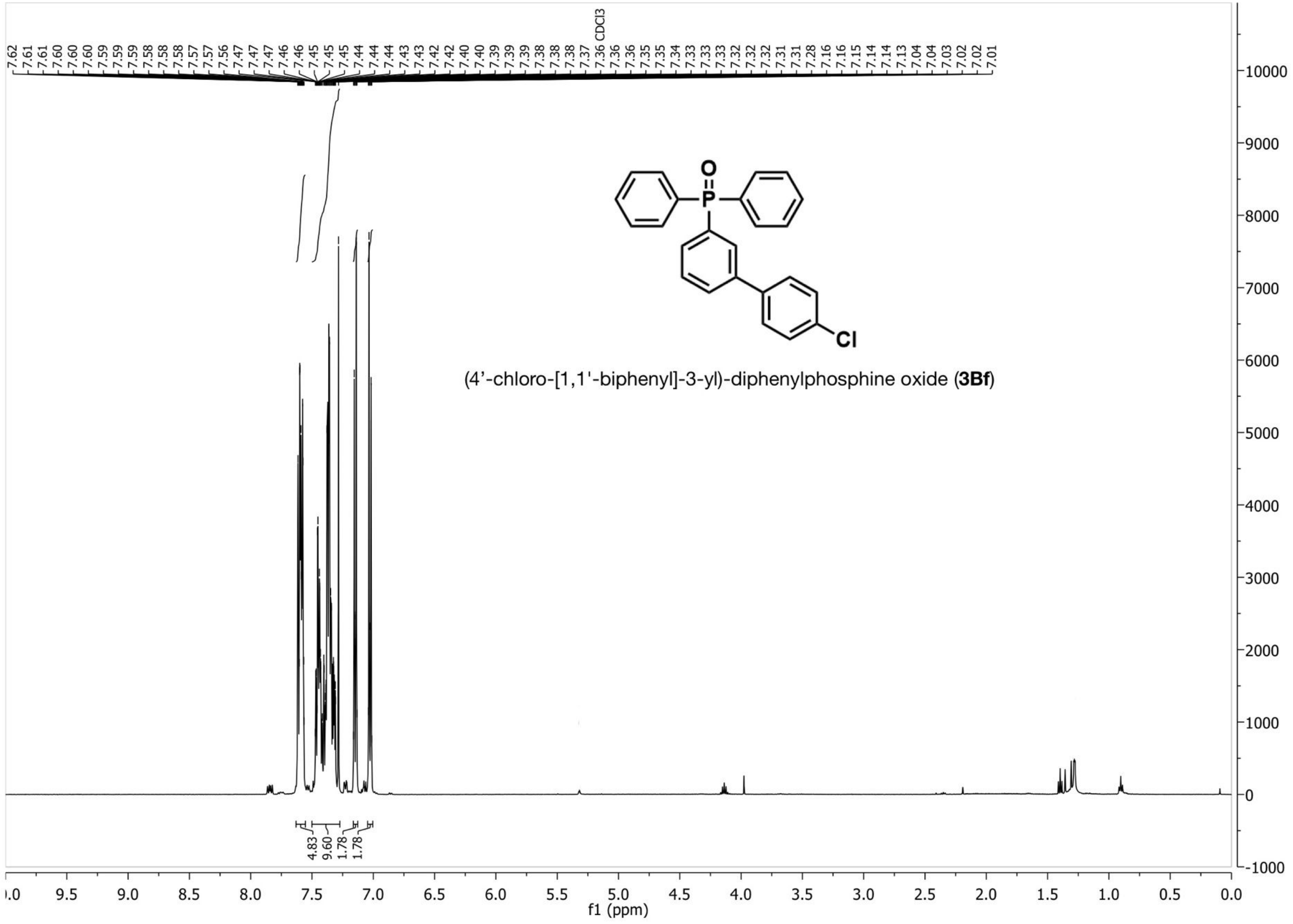

Figure S30. ^{1}H NMR ($CDCl_3$, 500 MHz) spectrum of (4'-chloro-[1,1'-biphenyl]-3-yl)-diphenylphosphine oxide (**3Bf**).

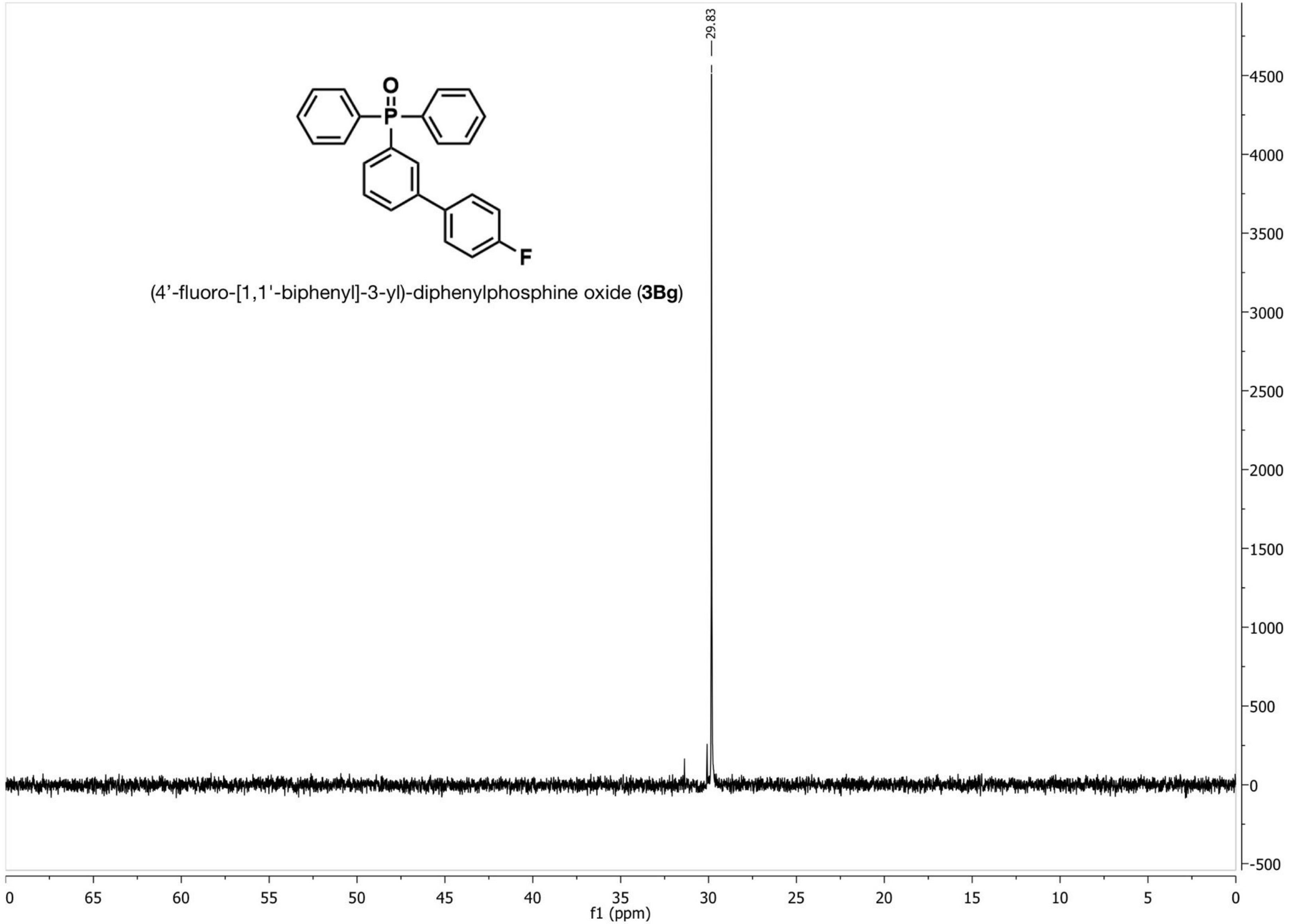

Figure S31. ^{31}P NMR ($CDCl_3$, 202.4 MHz) spectrum of (4'-fluoro-[1,1'-biphenyl]-3-yl)-diphenylphosphine oxide (**3Bg**).

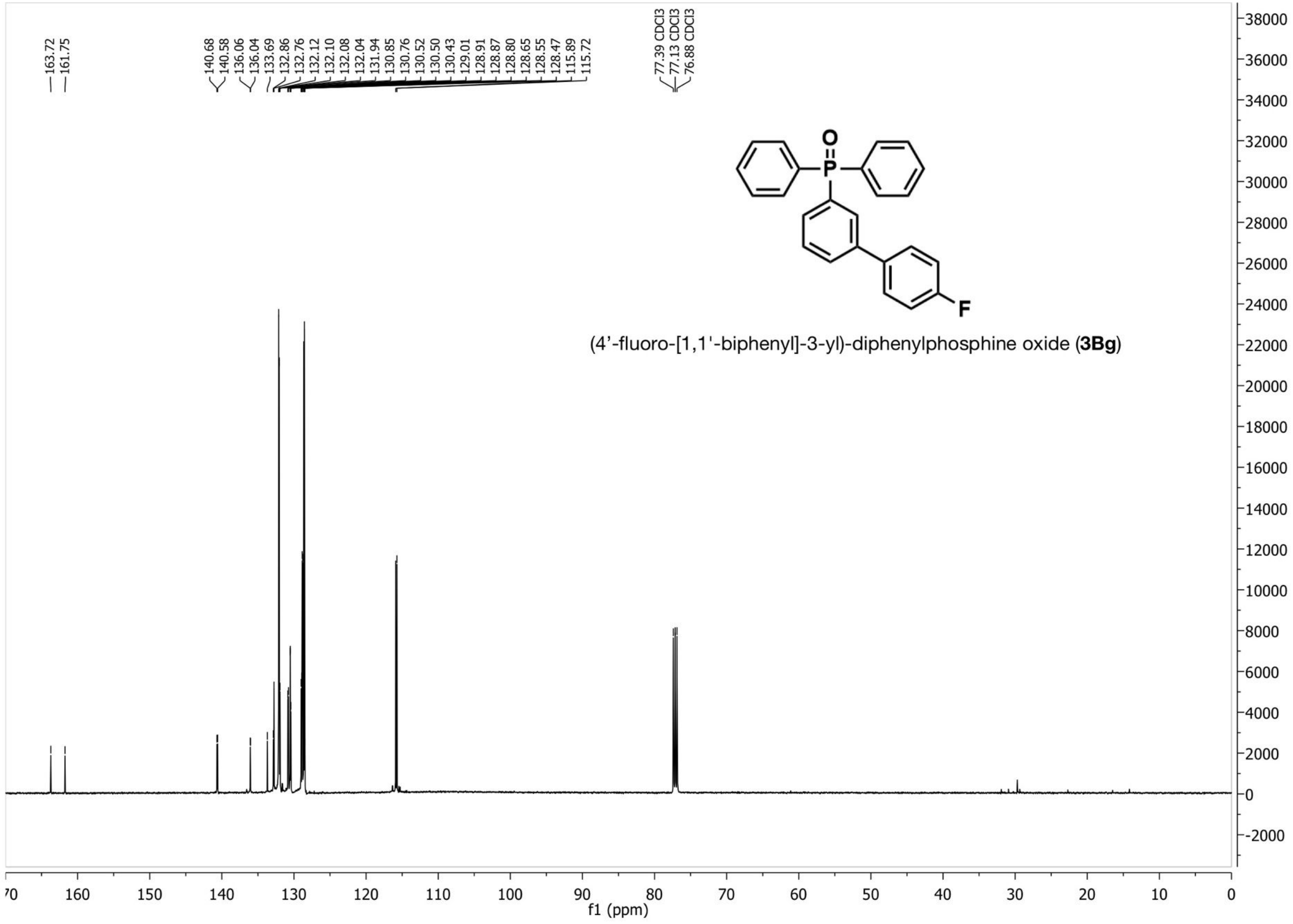

Figure S32. ^{13}C NMR ($CDCl_3$, 125.7 MHz) spectrum of (4'-fluoro-[1,1'-biphenyl]-3-yl)-diphenylphosphine oxide (**3Bg**).

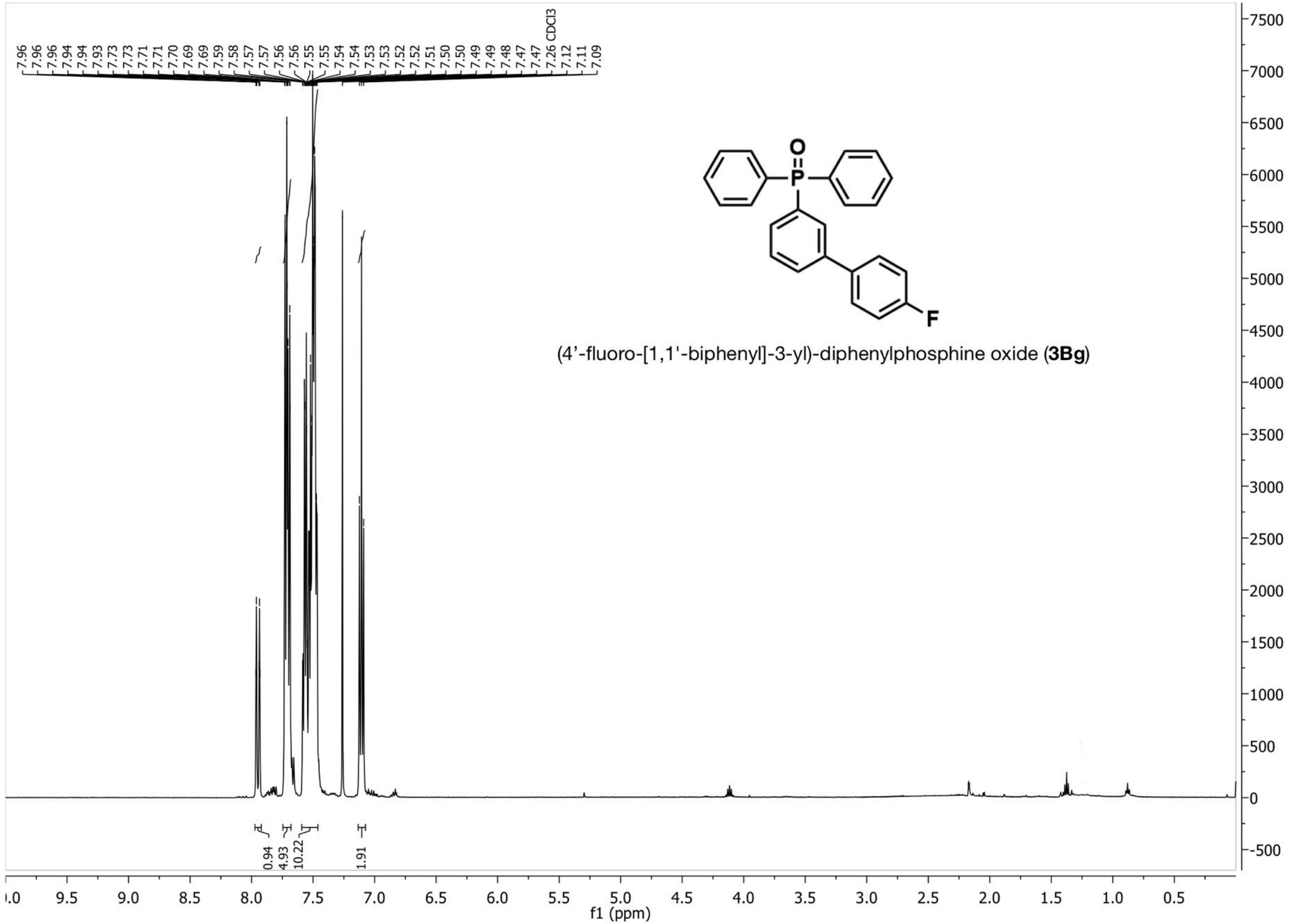

Figure S33. ^{1}H NMR ($CDCl_3$, 500 MHz) spectrum of (4'-fluoro-[1,1'-biphenyl]-3-yl)-diphenylphosphine oxide (**3Bg**).

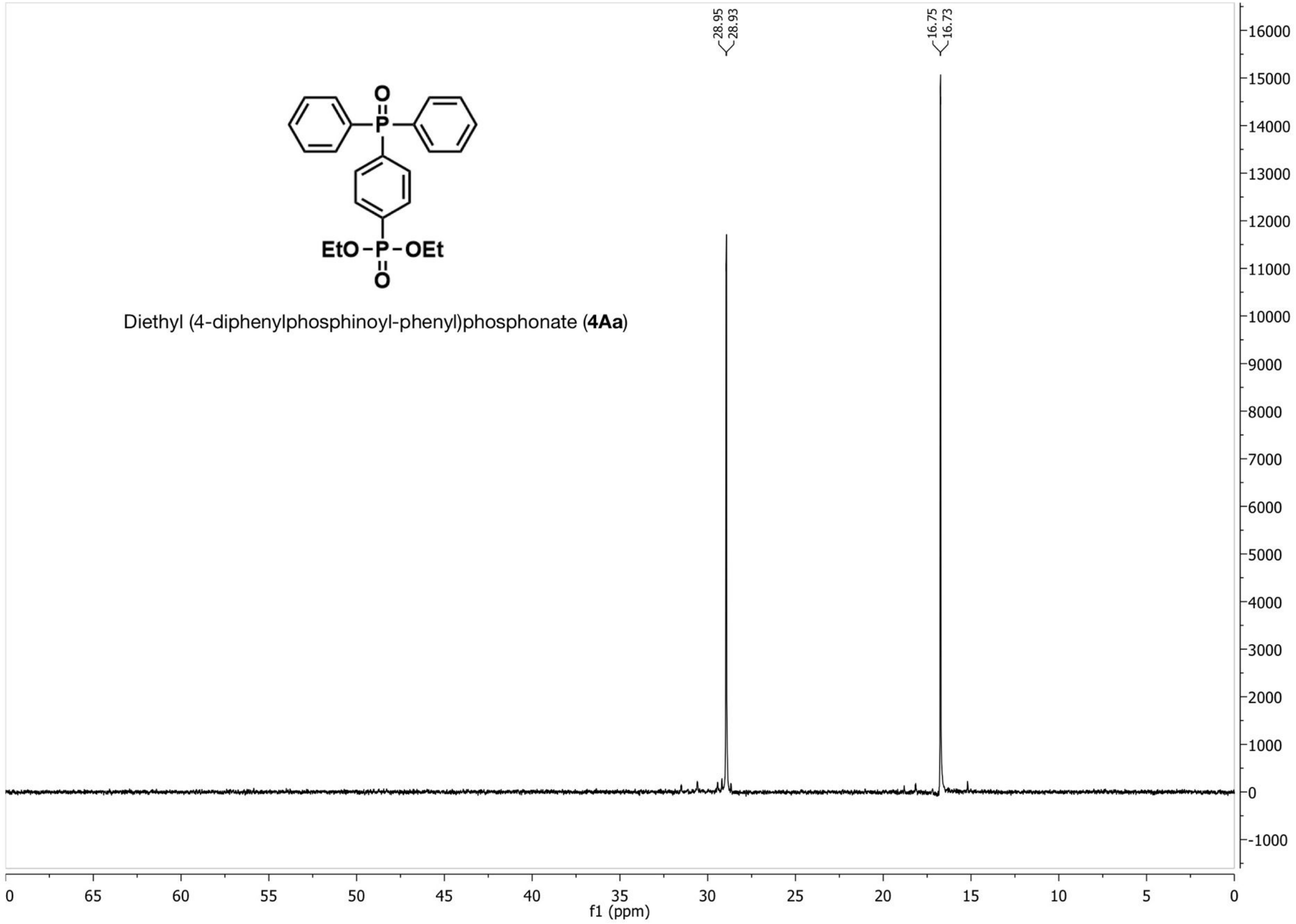

Figure S34. ^{31}P NMR ($CDCl_3$, 202.4 MHz) spectrum of diethyl (4-diphenylphosphinoyl-phenyl)phosphonate (**4Aa**).

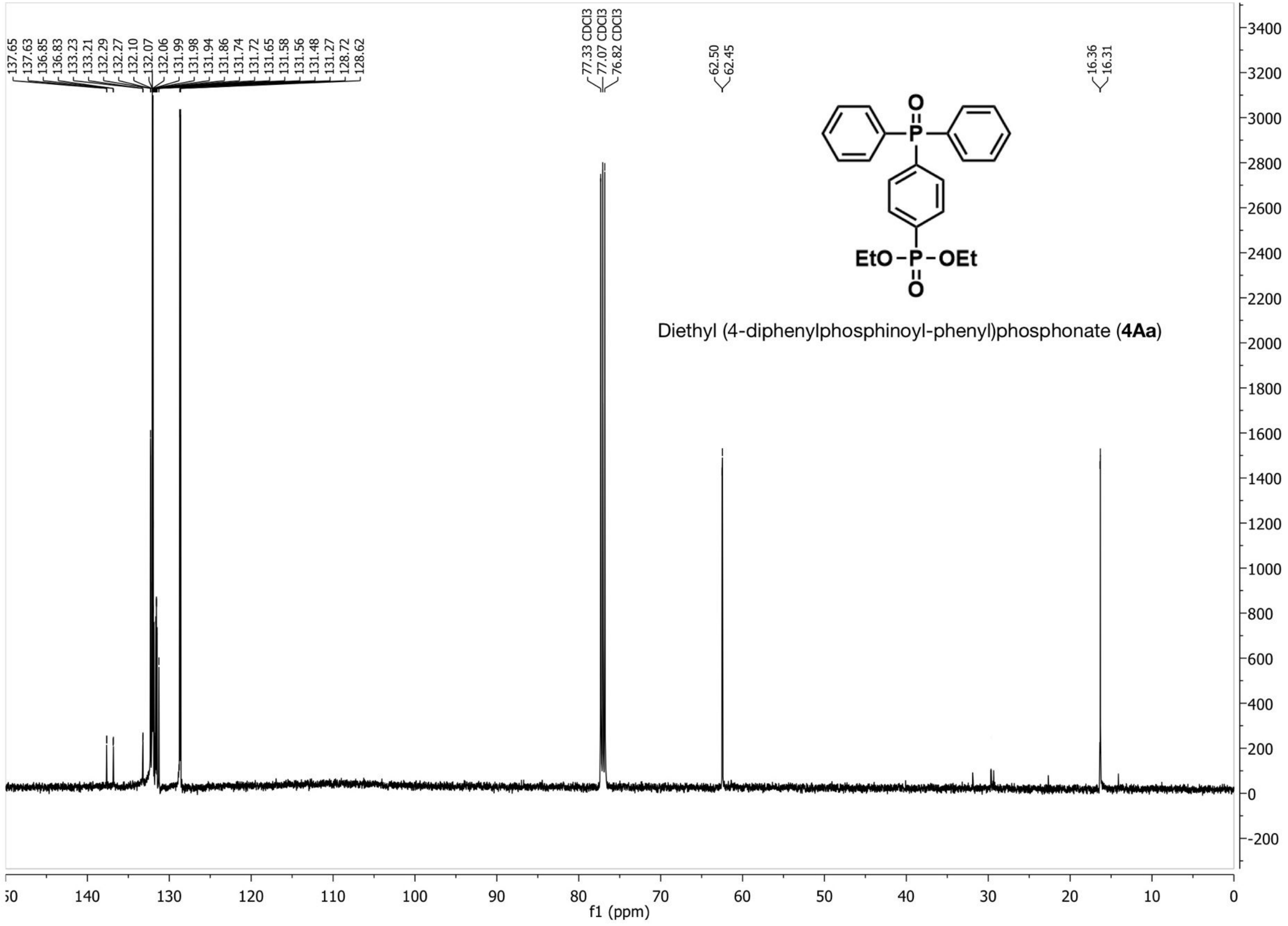

Figure S35. ^{13}C NMR ($CDCl_3$, 125.7 MHz) spectrum of diethyl (4-diphenylphosphinoyl-phenyl)phosphonate (**4Aa**).

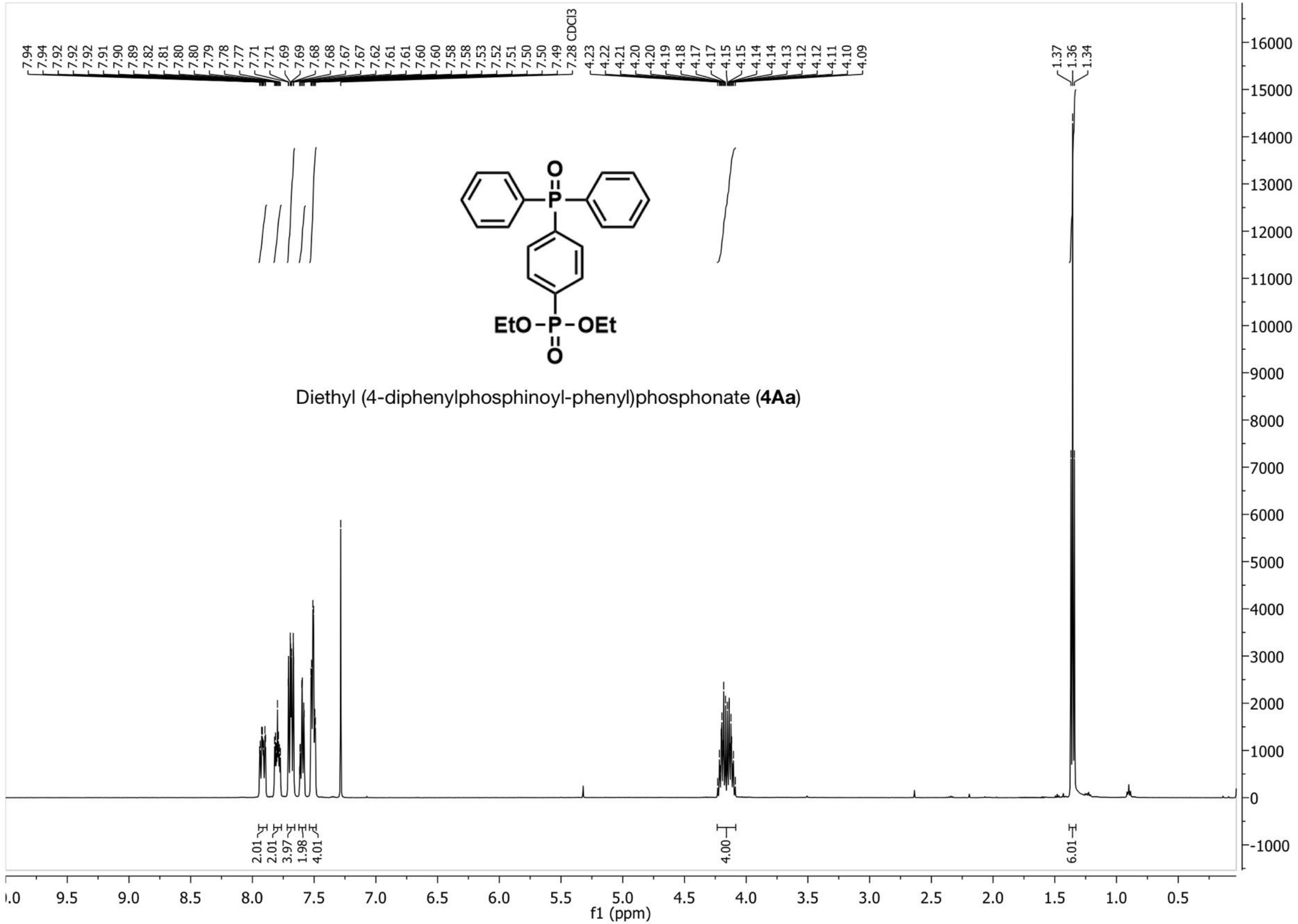

Figure S36. ^{1}H NMR ($CDCl_3$, 500 MHz) spectrum of diethyl (4-diphenylphosphinoyl-phenyl)phosphonate (**4Aa**).

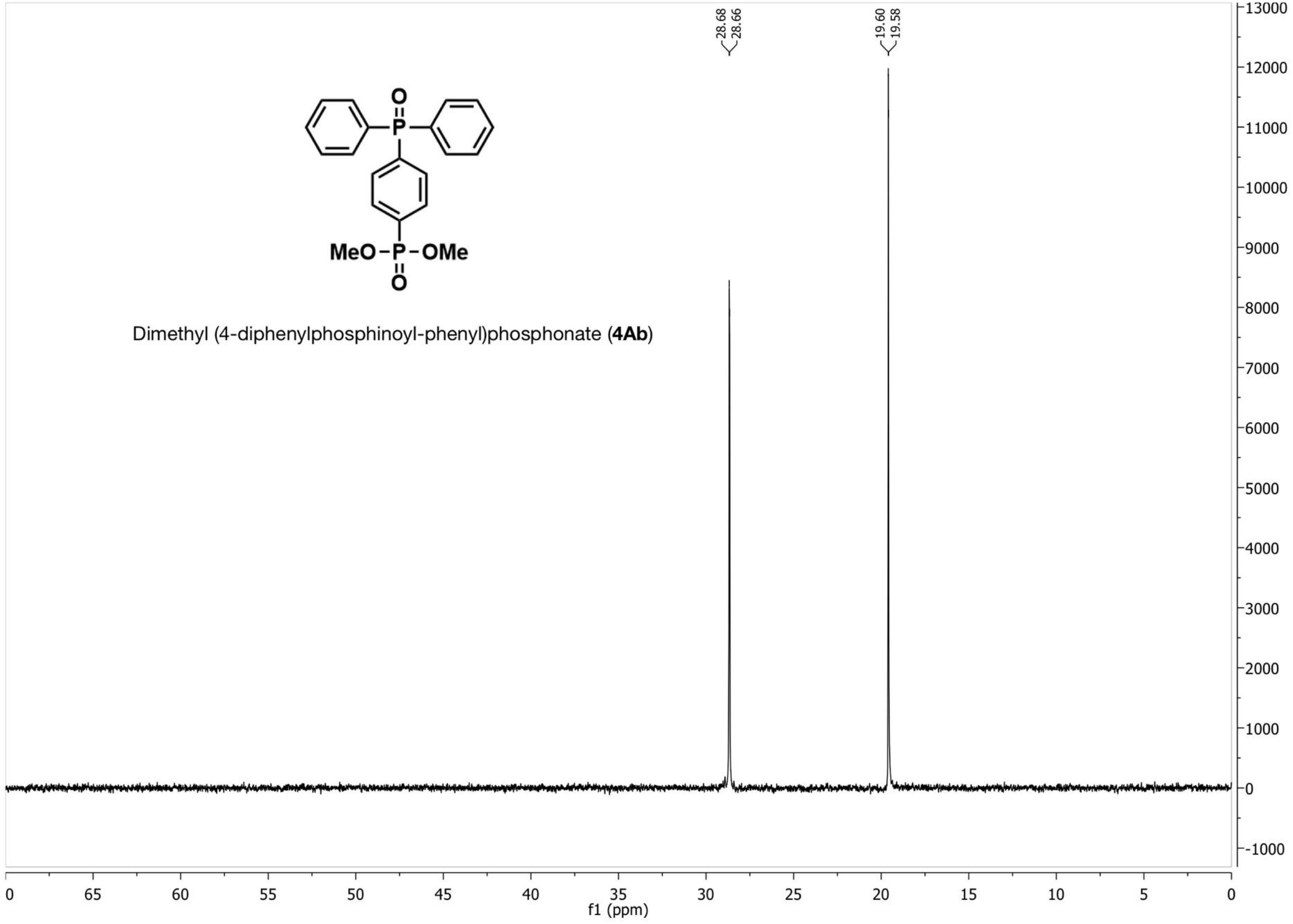

Figure S37. ^{31}P NMR ($CDCl_3$, 202.4 MHz) spectrum of dimethyl (4-diphenylphosphinoyl-phenyl)phosphonate (**4Ab**).

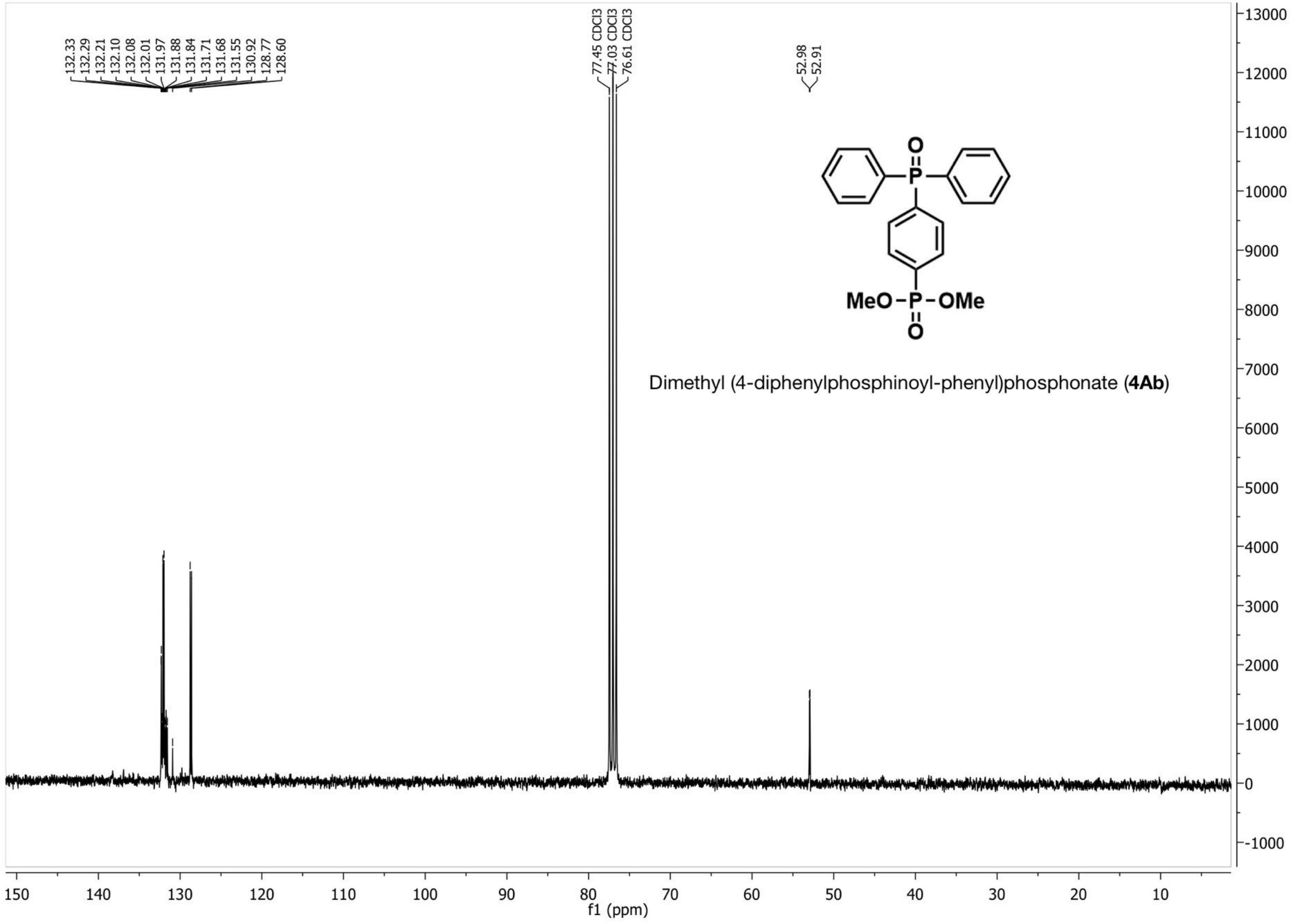

Figure S38. ^{13}C NMR ($CDCl_3$, 125.7 MHz) spectrum of dimethyl (4-diphenylphosphinoyl-phenyl)phosphonate (**4Ab**).

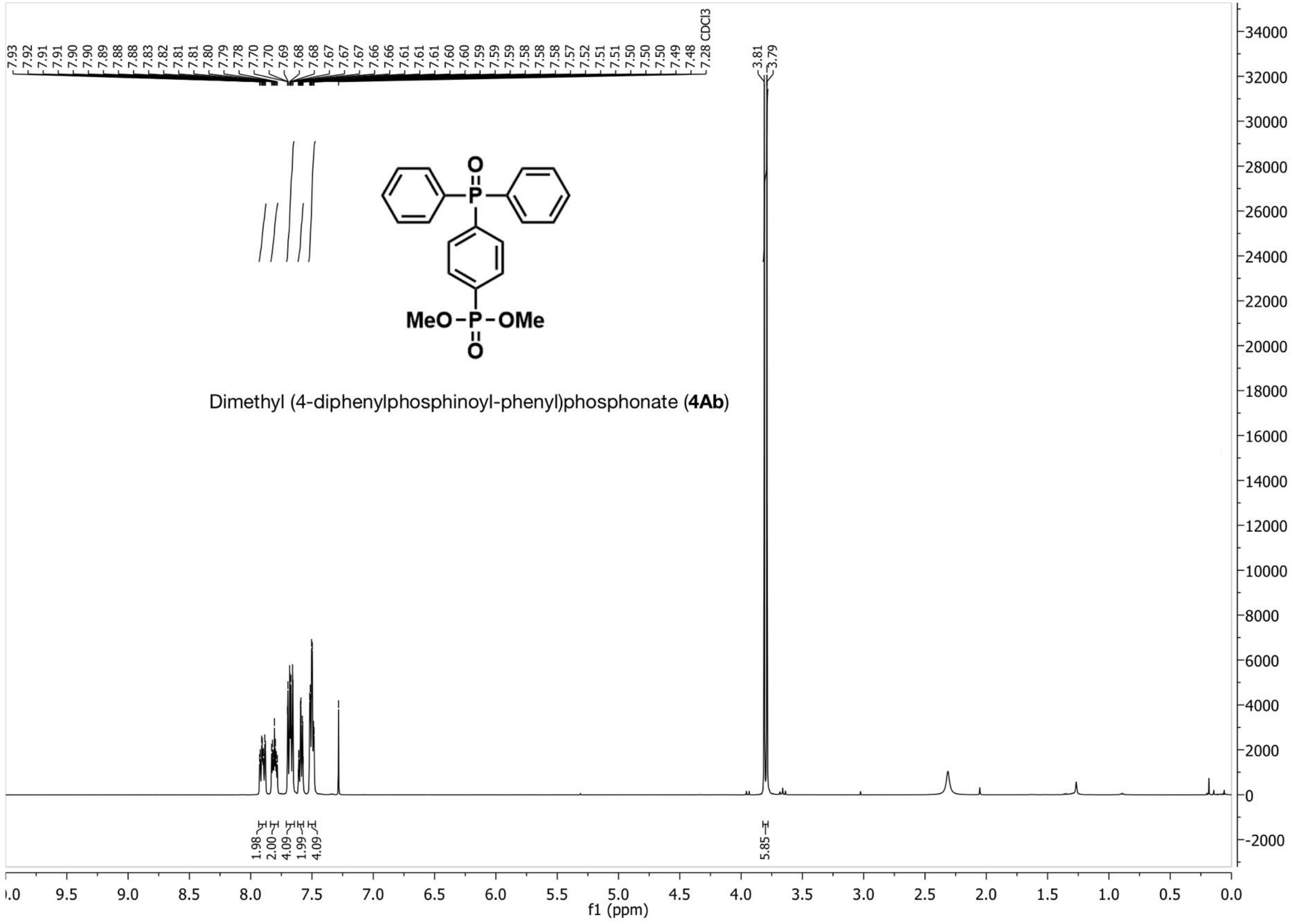

Figure S39. ^{1}H NMR ($CDCl_3$, 500 MHz) spectrum of dimethyl (4-diphenylphosphinoyl-phenyl)phosphonate (**4Ab**).

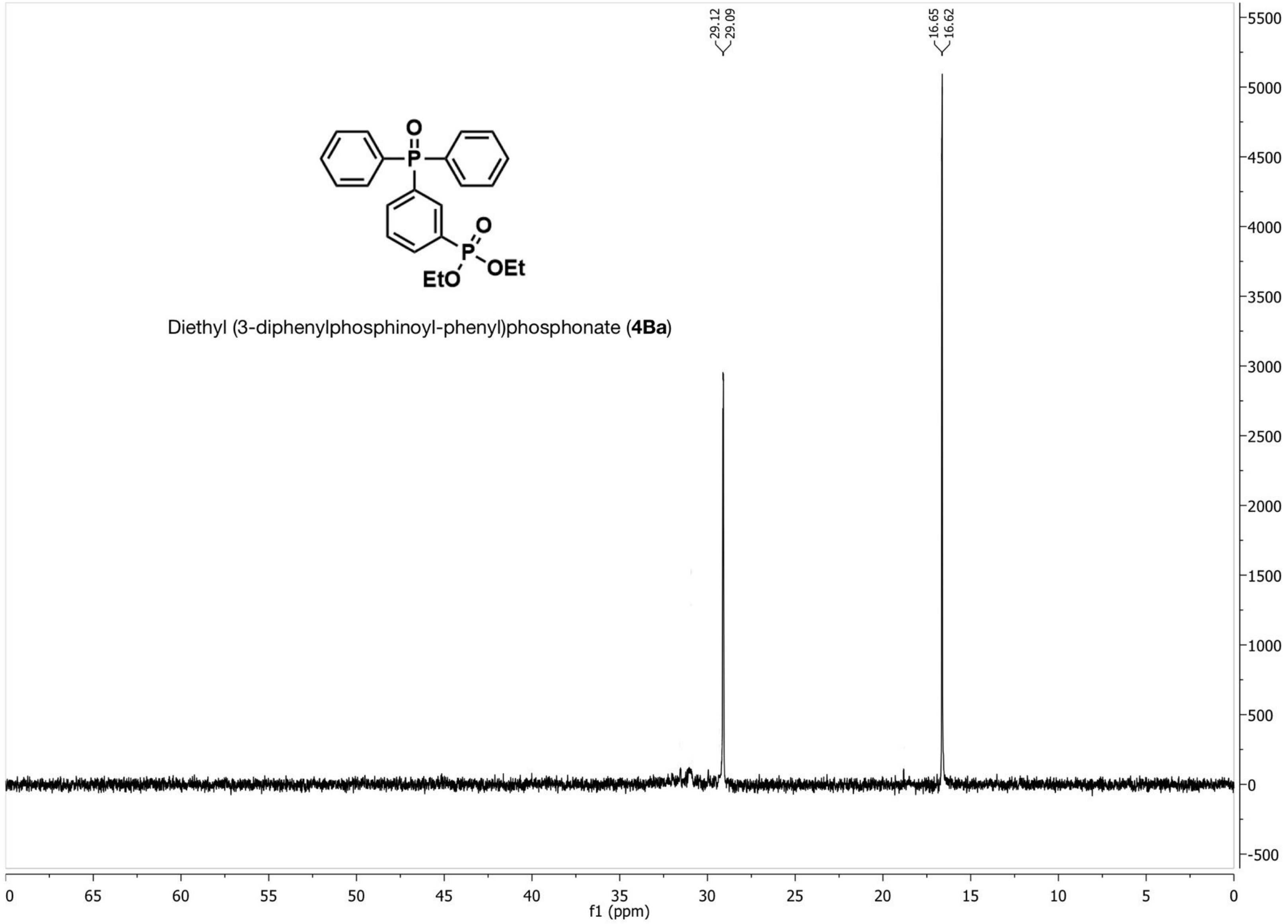

Figure S40. ^{31}P NMR ($CDCl_3$, 202.4 MHz) spectrum of diethyl (3-diphenylphosphinoyl-phenyl)phosphonate (**4Ba**).

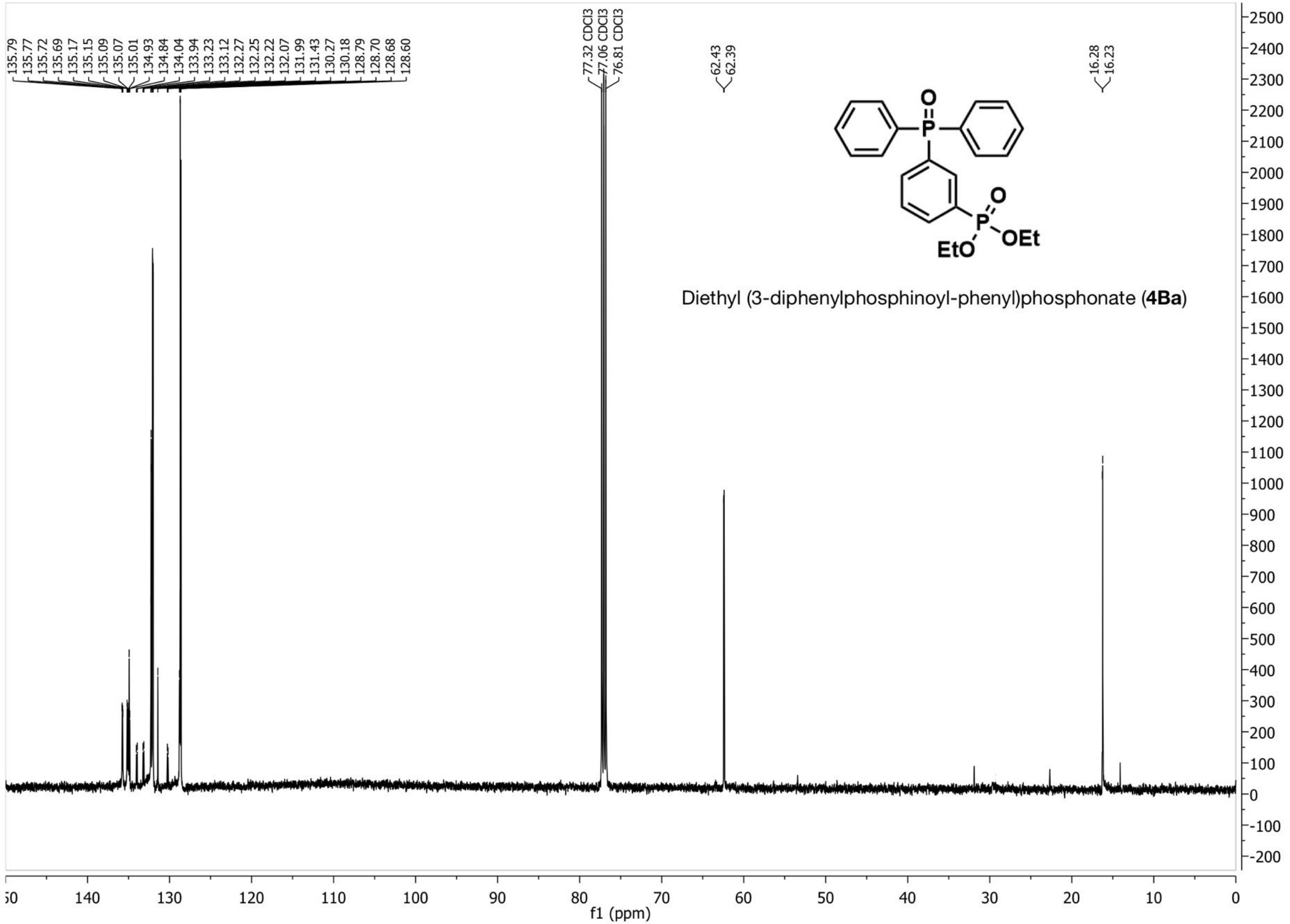

Figure S41. ^{13}C NMR ($CDCl_3$, 125.7 MHz) spectrum of diethyl (3-diphenylphosphinoyl-phenyl)phosphonate (**4Ba**).

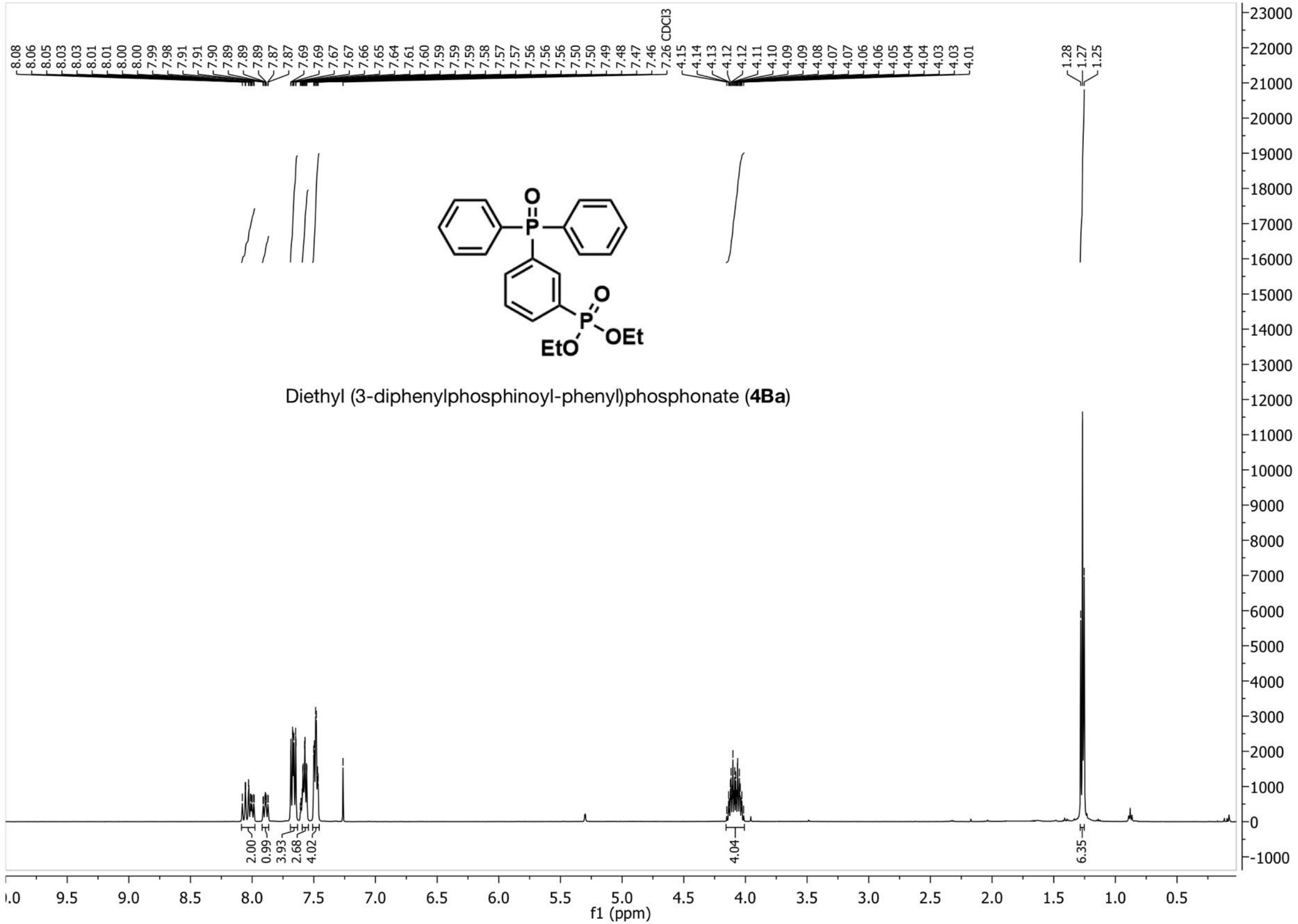

Figure S42. ^{1}H NMR ($CDCl_3$, 500 MHz) spectrum of diethyl (3-diphenylphosphinoyl-phenyl)phosphonate (**4Ba**).

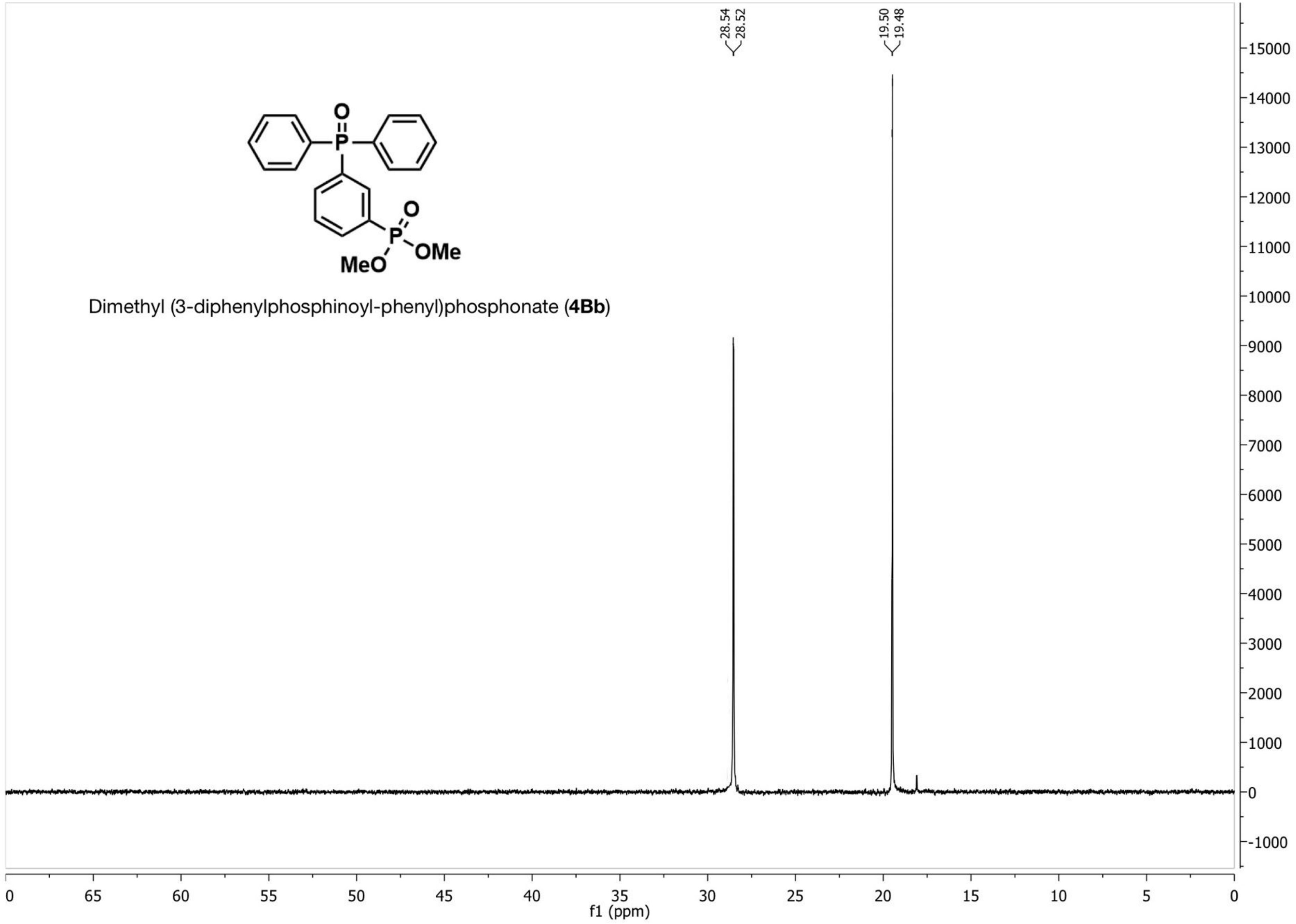

Figure S43. ^{31}P NMR($CDCl_3$, 202.4 MHz) spectrum of dimethyl (3-diphenylphosphinoyl-phenyl)phosphonate (**4Bb**).

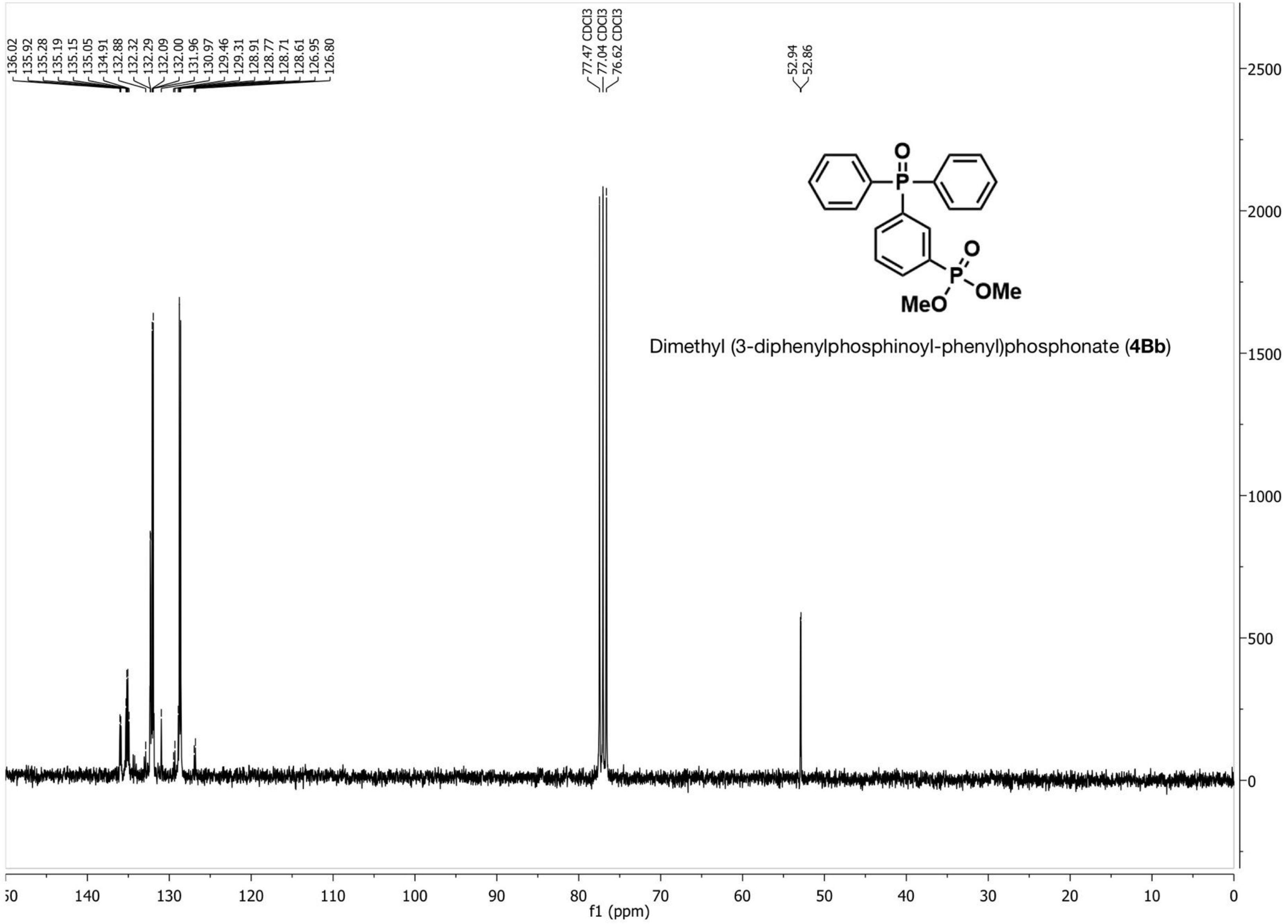

Figure S44. ^{13}C NMR ($CDCl_3$, 125.7 MHz) spectrum of dimethyl (3-diphenylphosphinoyl-phenyl)phosphonate (**4Bb**).

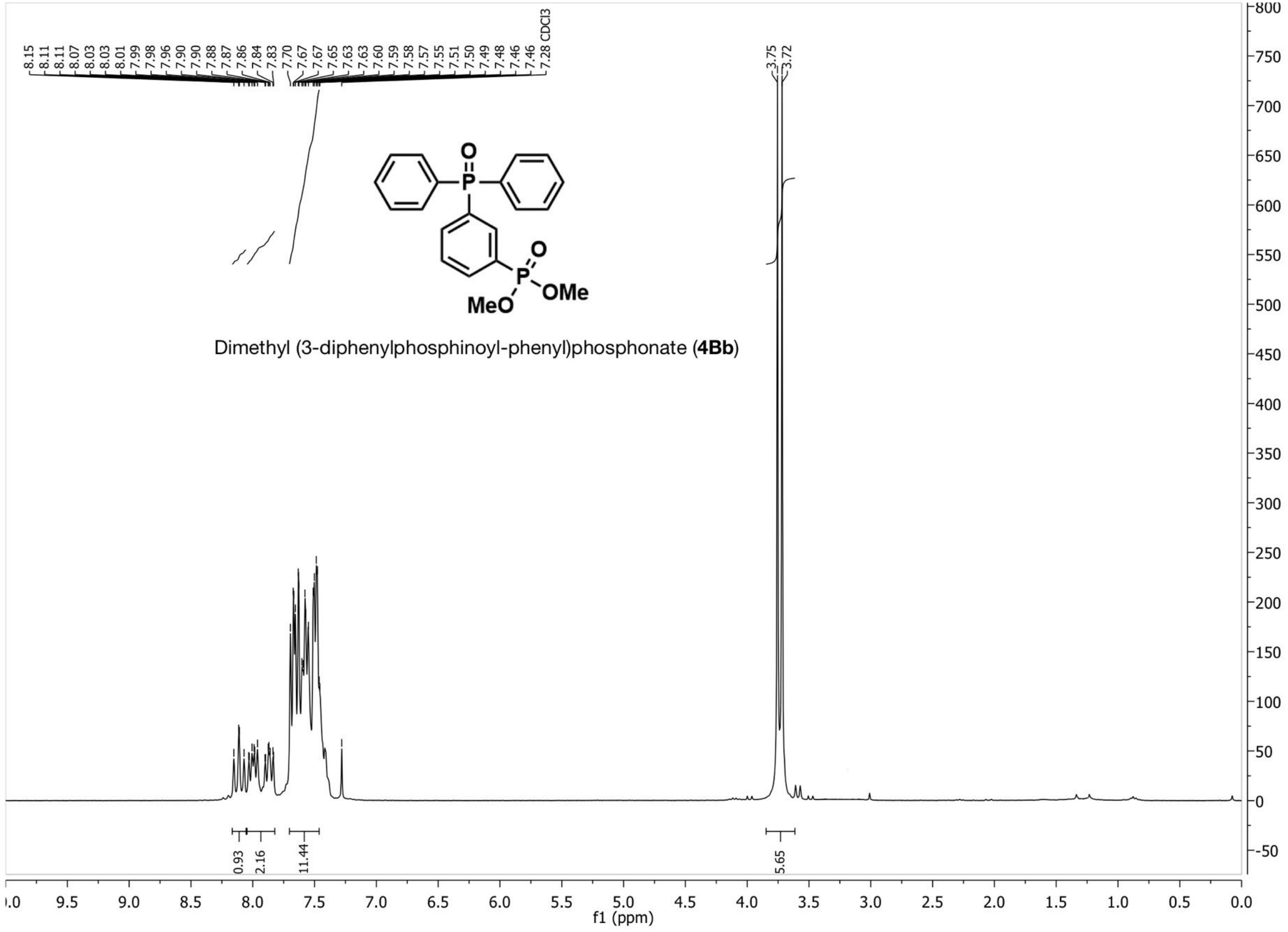

Figure S45. ^{1}H NMR ($CDCl_3$, 500 MHz) spectrum of dimethyl (3-diphenylphosphinoyl-phenyl)phosphonate (**4Bb**).

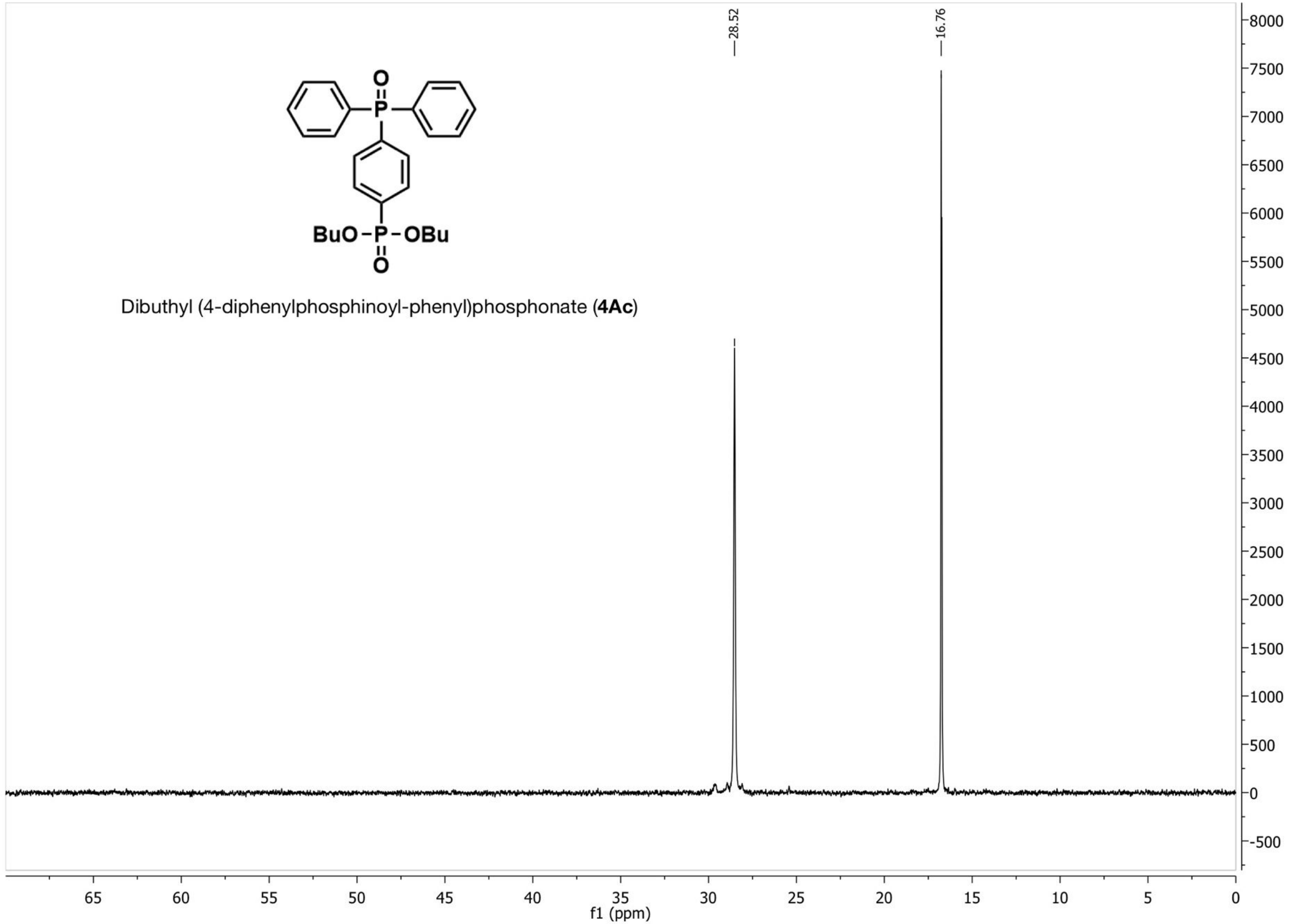

Figure S46. ³¹P NMR ($CDCl_3$, 202.4 MHz) spectrum of dibutyl (4-diphenylphosphinoyl-phenyl)phosphonate (**4Ac**).

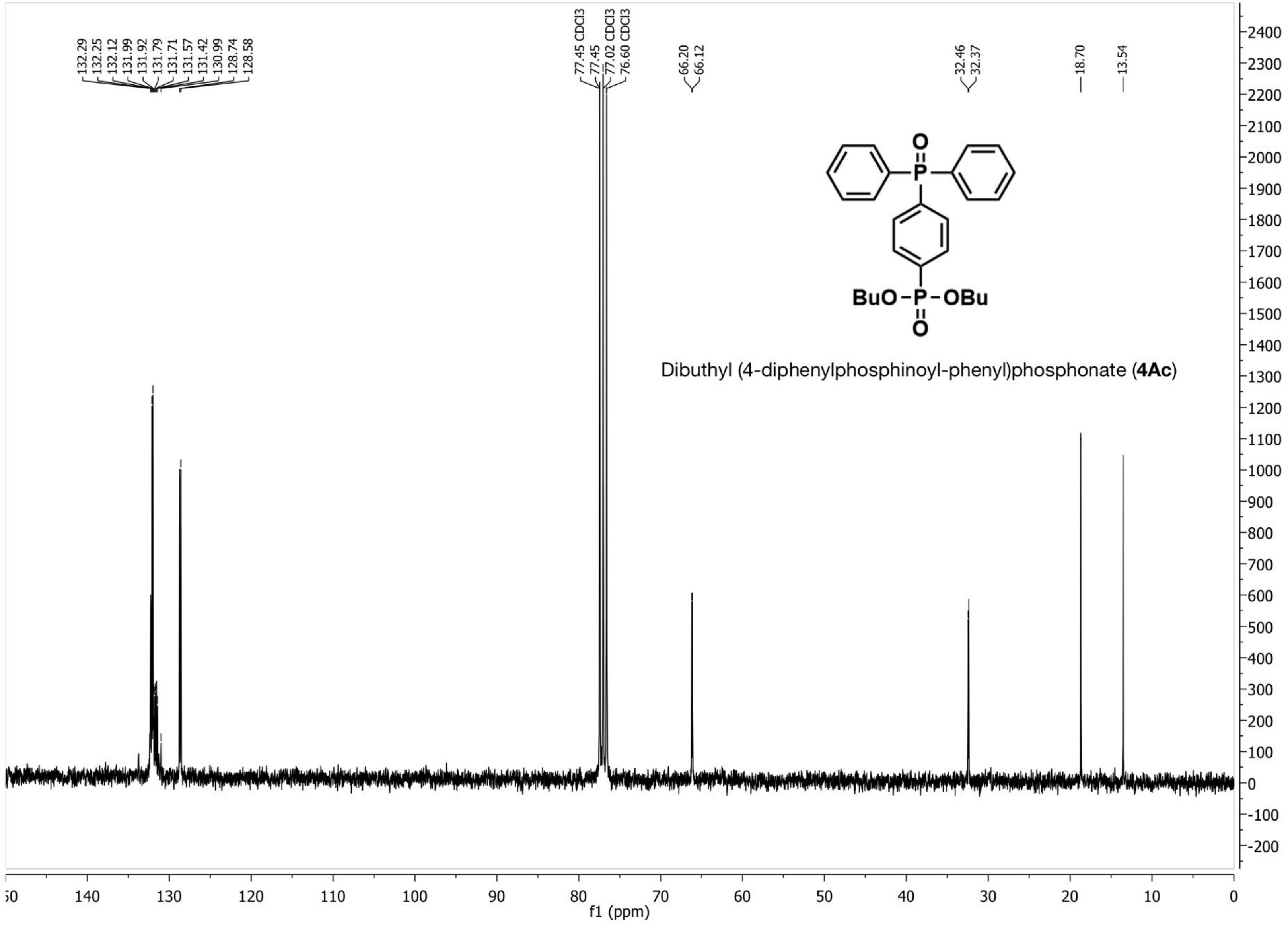

Figure S47. ^{13}C NMR ($CDCl_3$, 125.7 MHz) spectrum of dibutyl (4-diphenylphosphinoyl-phenyl)phosphonate (**4Ac**).

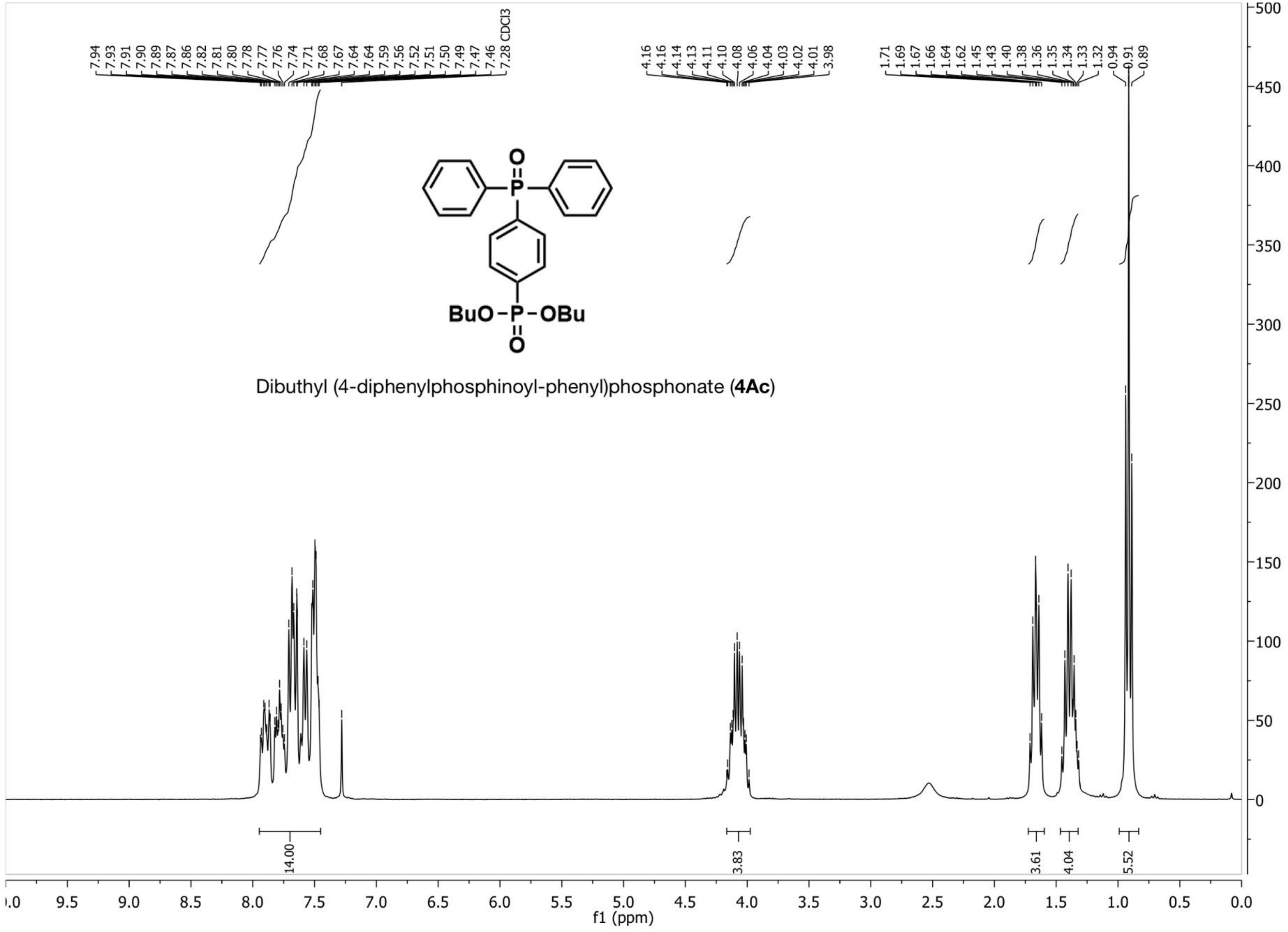

Figure S48. ^{1}H NMR ($CDCl_3$, 500 MHz) spectrum of dibutyl (4-diphenylphosphinoyl-phenyl)phosphonate (**4Ac**).

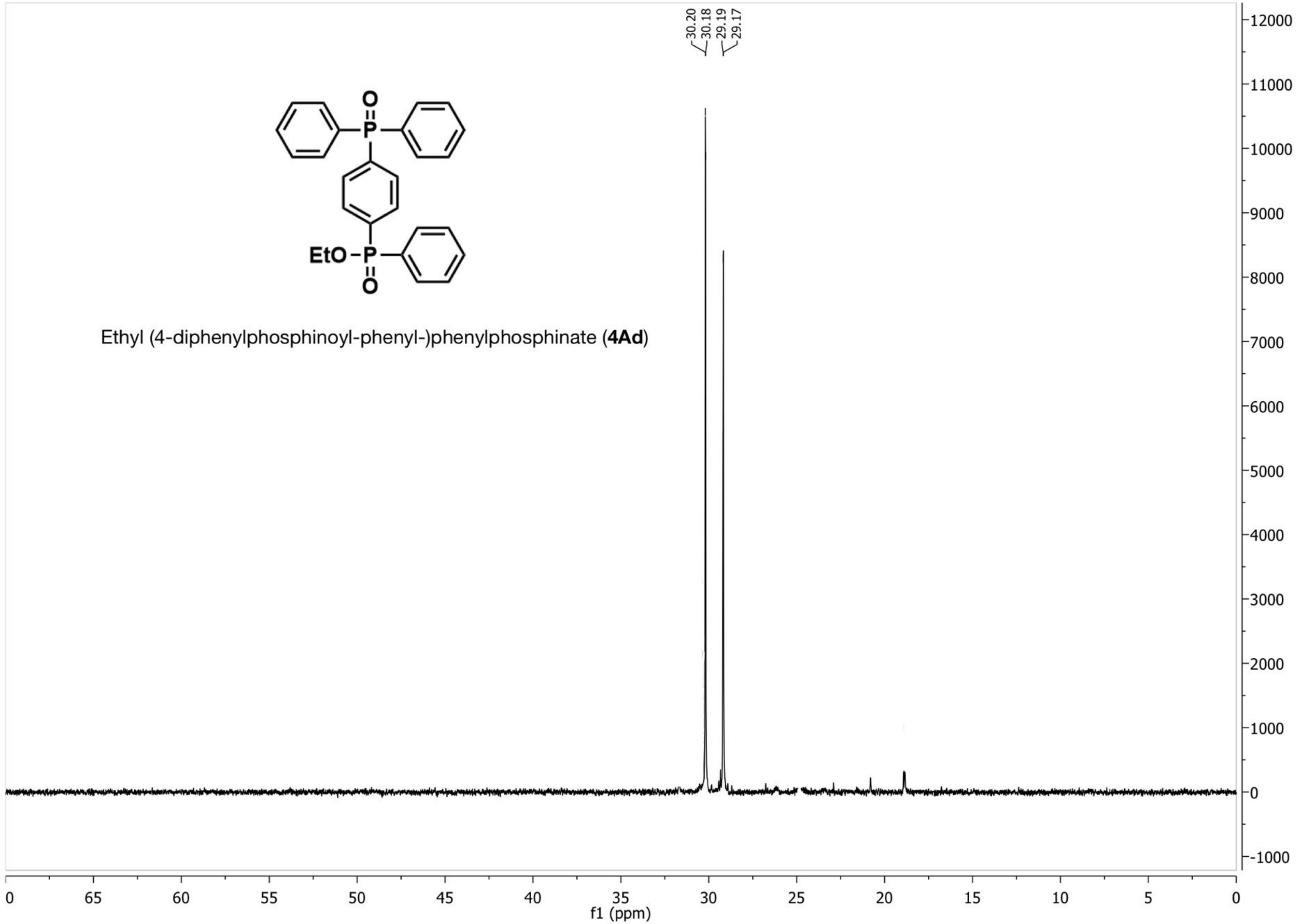

Figure S49. ^{31}P NMR ($CDCl_3$, 202.4 MHz) spectrum of ethyl (4-diphenylphosphinoyl-phenyl-)phenylphosphinate (**4Ad**).

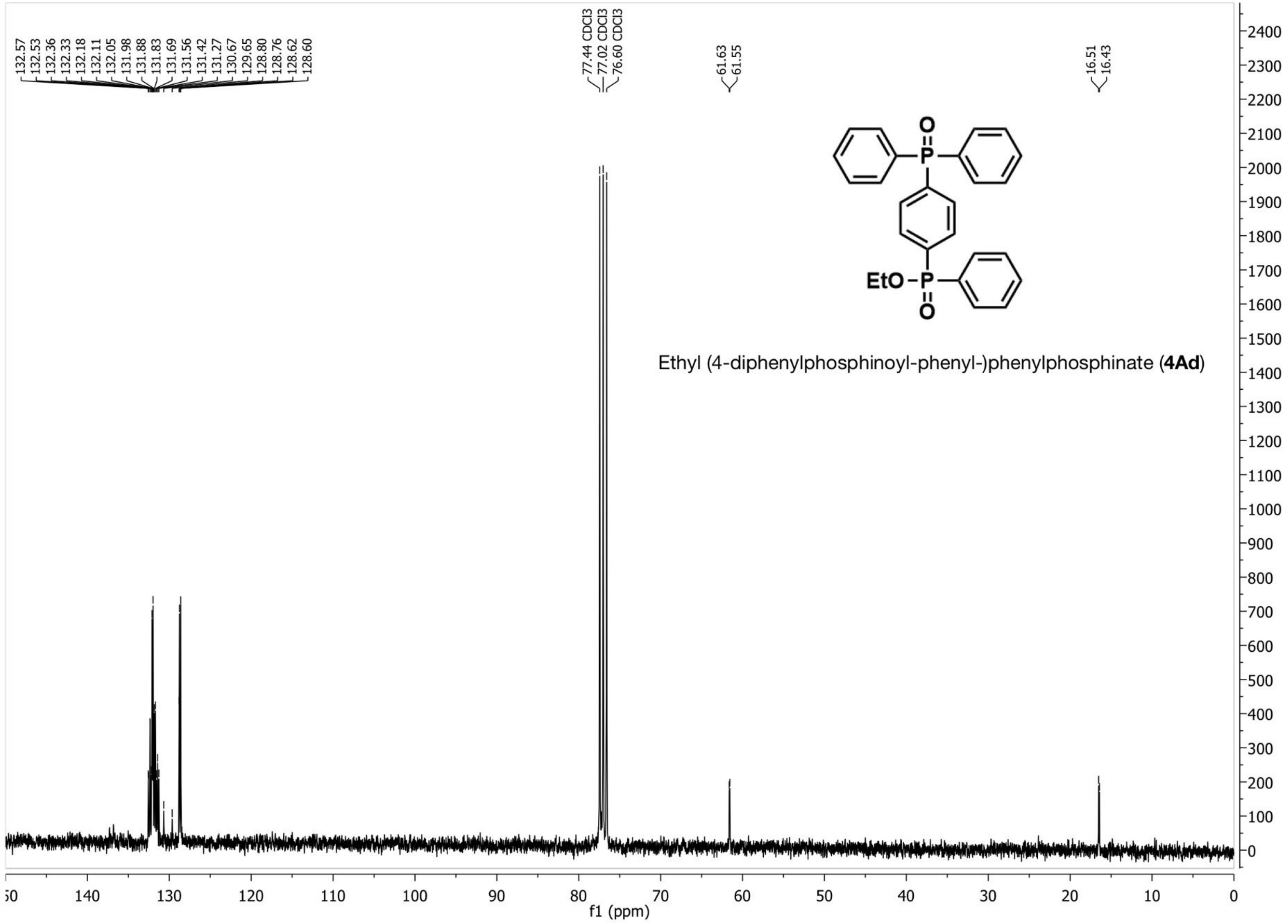

Figure S50. ^{13}C NMR ($CDCl_3$, 125.7 MHz) spectrum of ethyl (4-diphenylphosphinoyl-phenyl-)phenylphosphinate (**4Ad**).

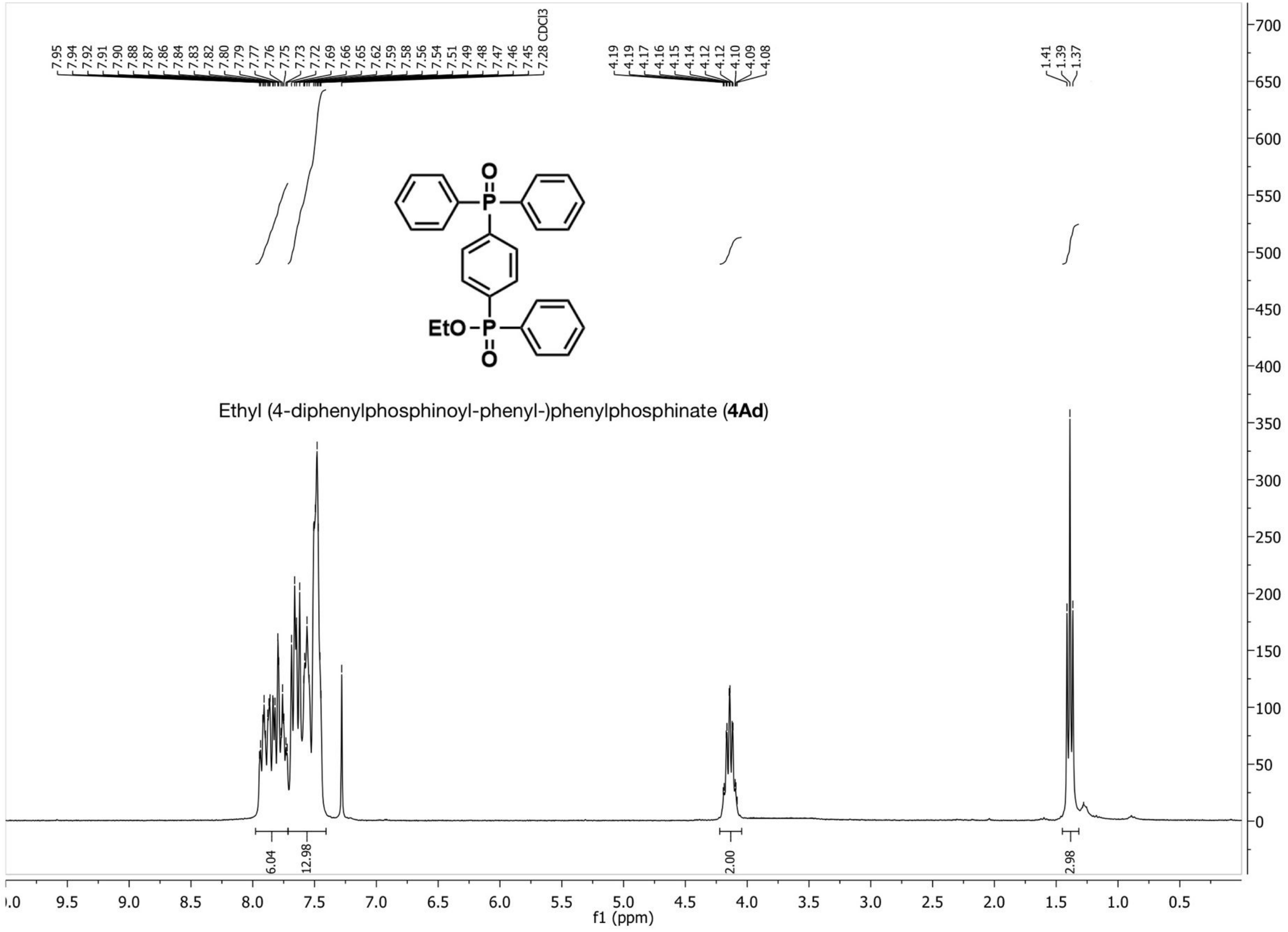

Figure S51. ^{1}H NMR ($CDCl_3$, 500 MHz) spectrum of ethyl (4-diphenylphosphinoyl-phenyl-)phenylphosphinate (**4Ad**).

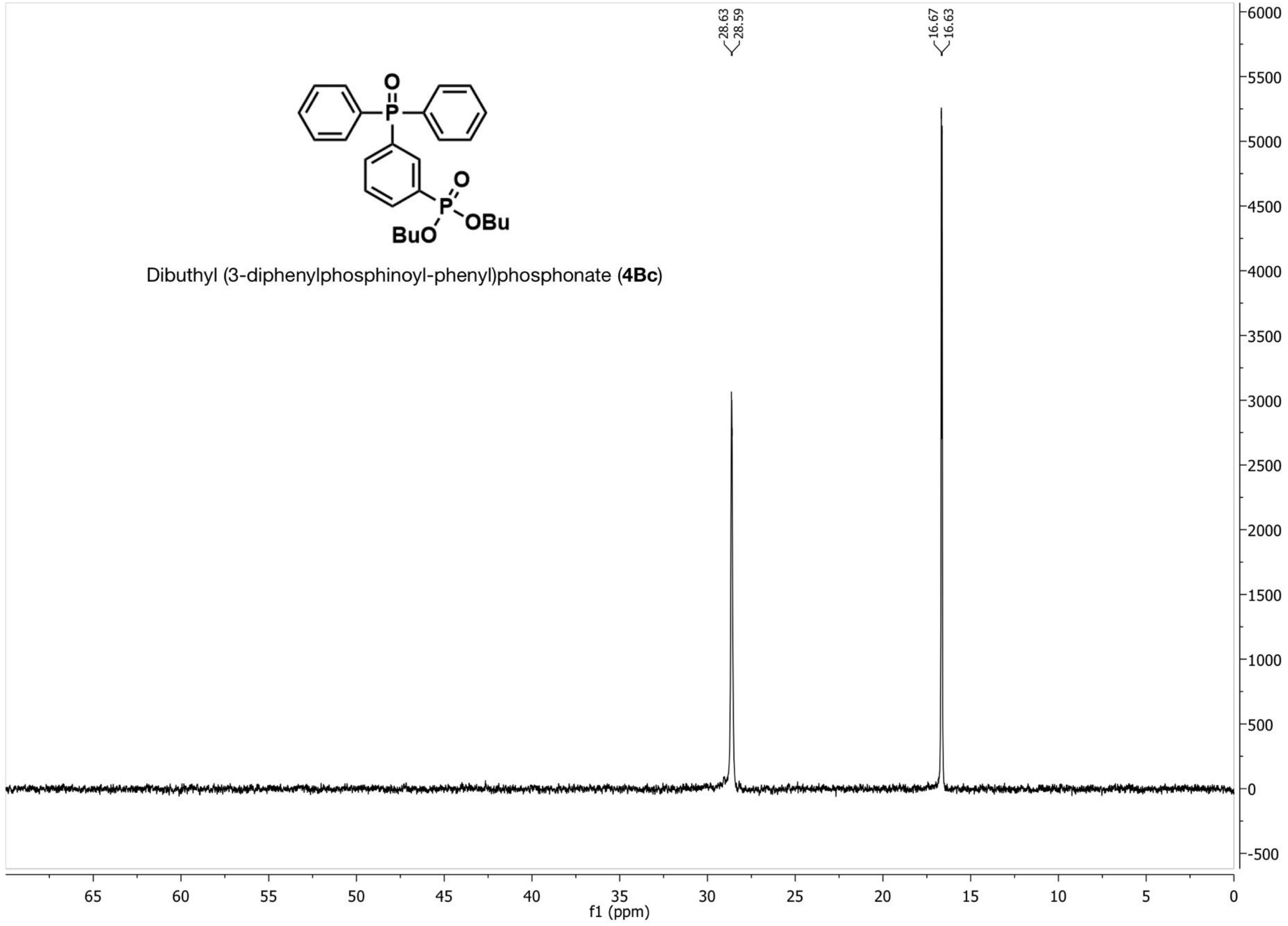

Figure S52. ^{31}P NMR ($CDCl_3$, 202.4 MHz) spectrum of dibutyl (3-diphenylphosphinoyl-phenyl)phosphonate (**4Bc**).

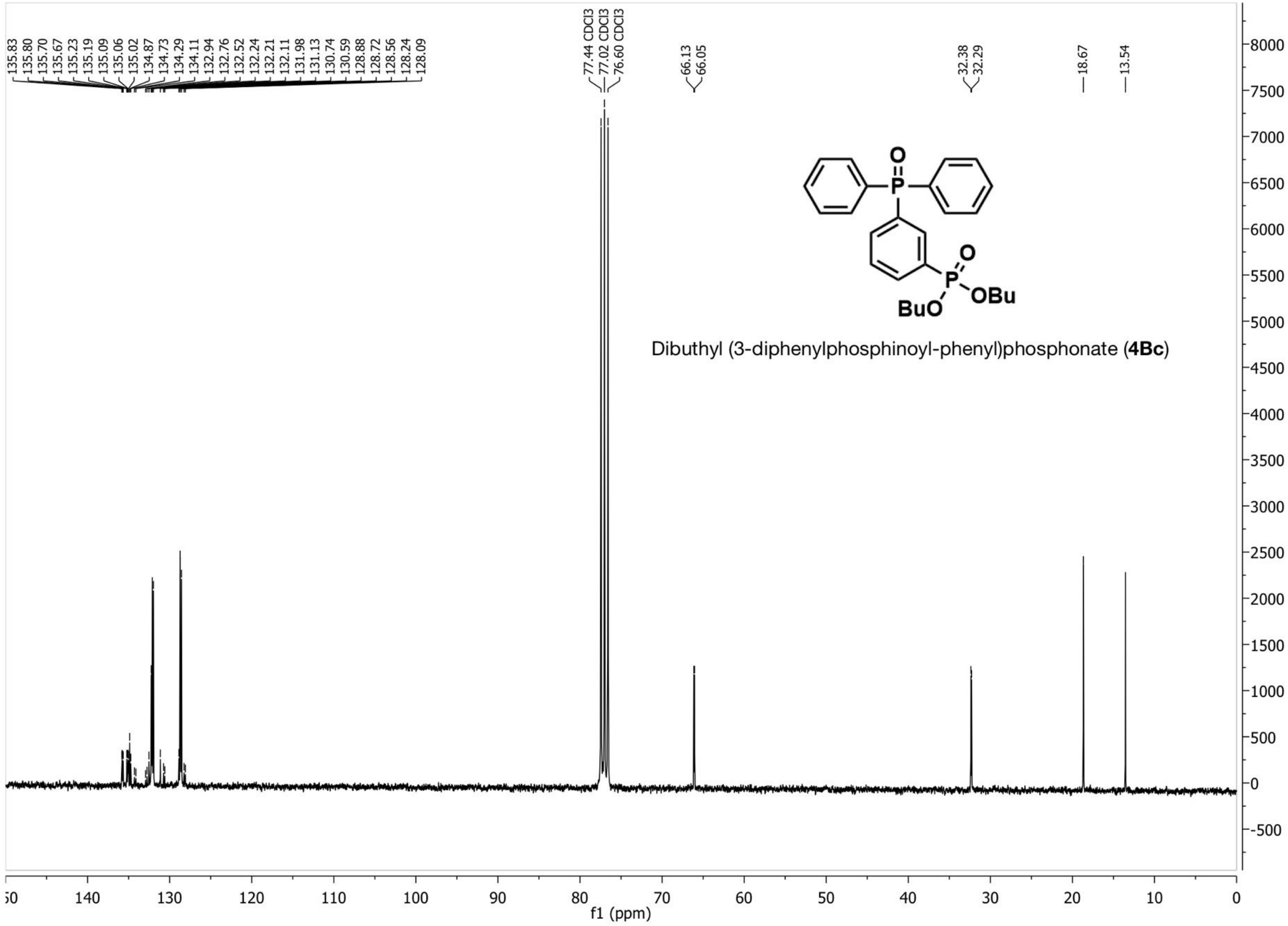

Figure S53. ^{13}C NMR ($CDCl_3$, 125.7 MHz) spectrum of dibutyl (3-diphenylphosphinoyl-phenyl)phosphonate (**4Bc**).

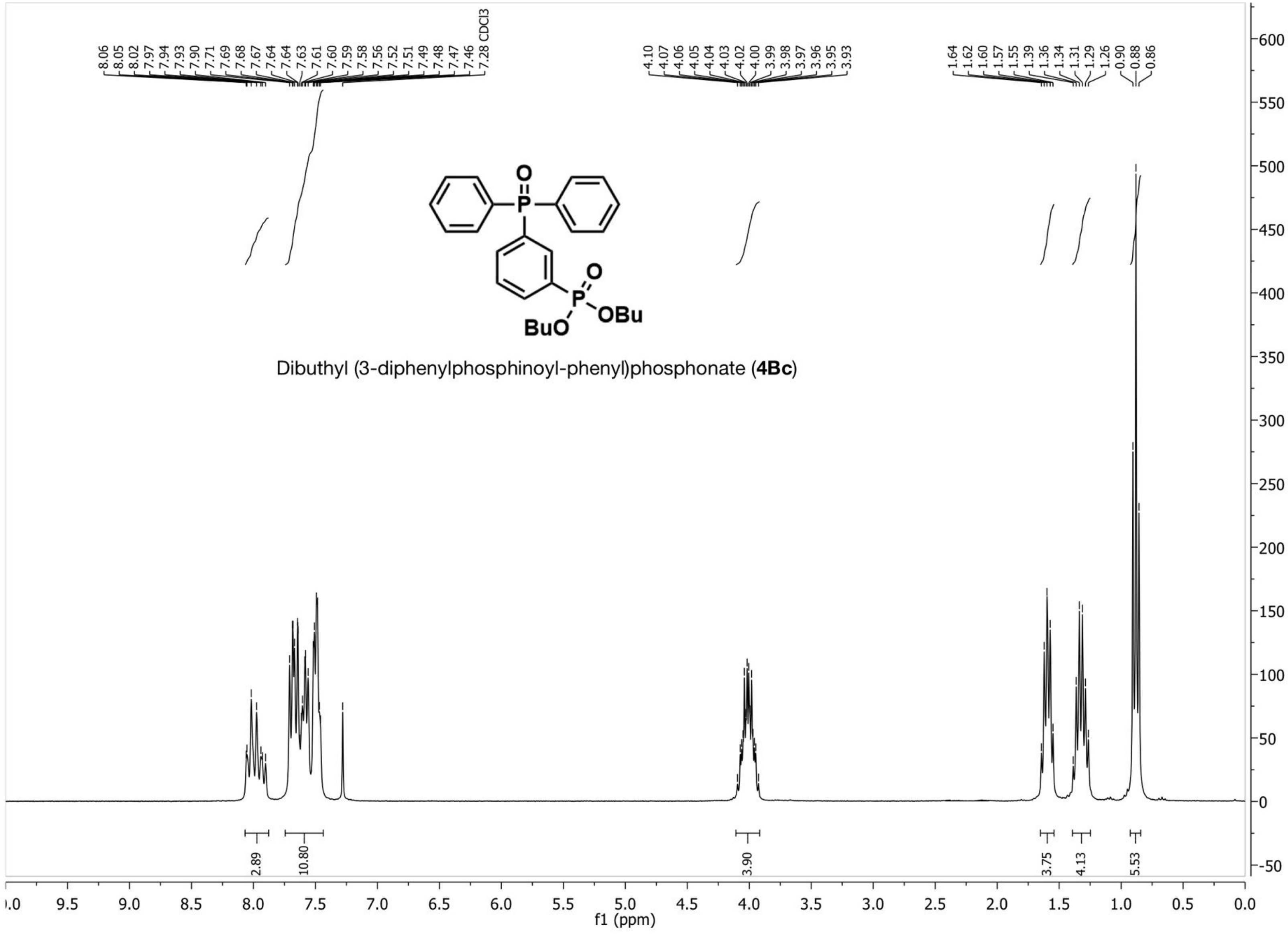

Figure S54. ^{1}H NMR ($CDCl_3$, 500 MHz) spectrum of dibutyl (3-diphenylphosphinoyl-phenyl)phosphonate (**4Bc**).

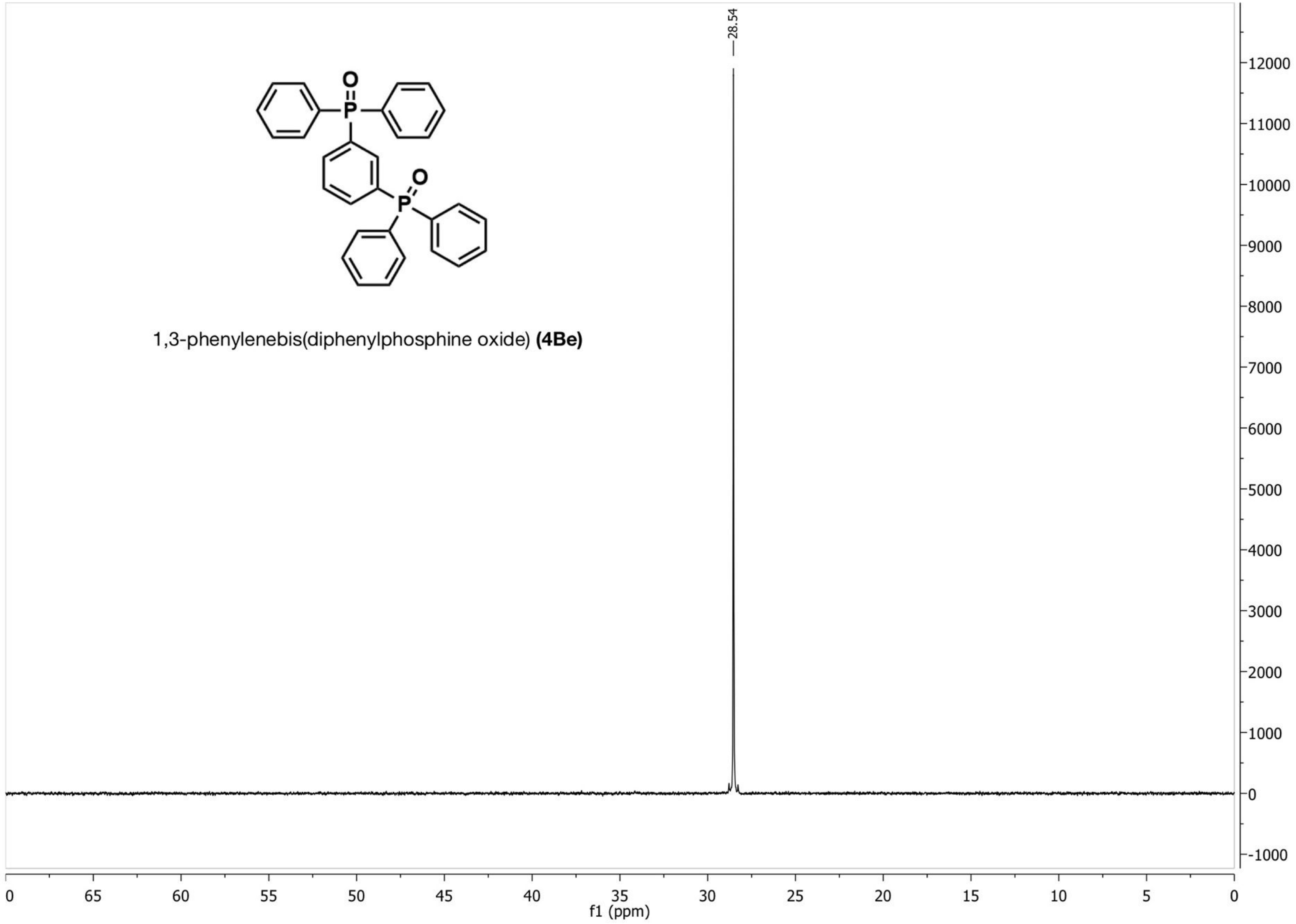

Figure S55. ^{31}P NMR ($CDCl_3$, 202.4 MHz) spectrum of 1,3-phenylenebis(diphenylphosphine oxide) (**4Be**).

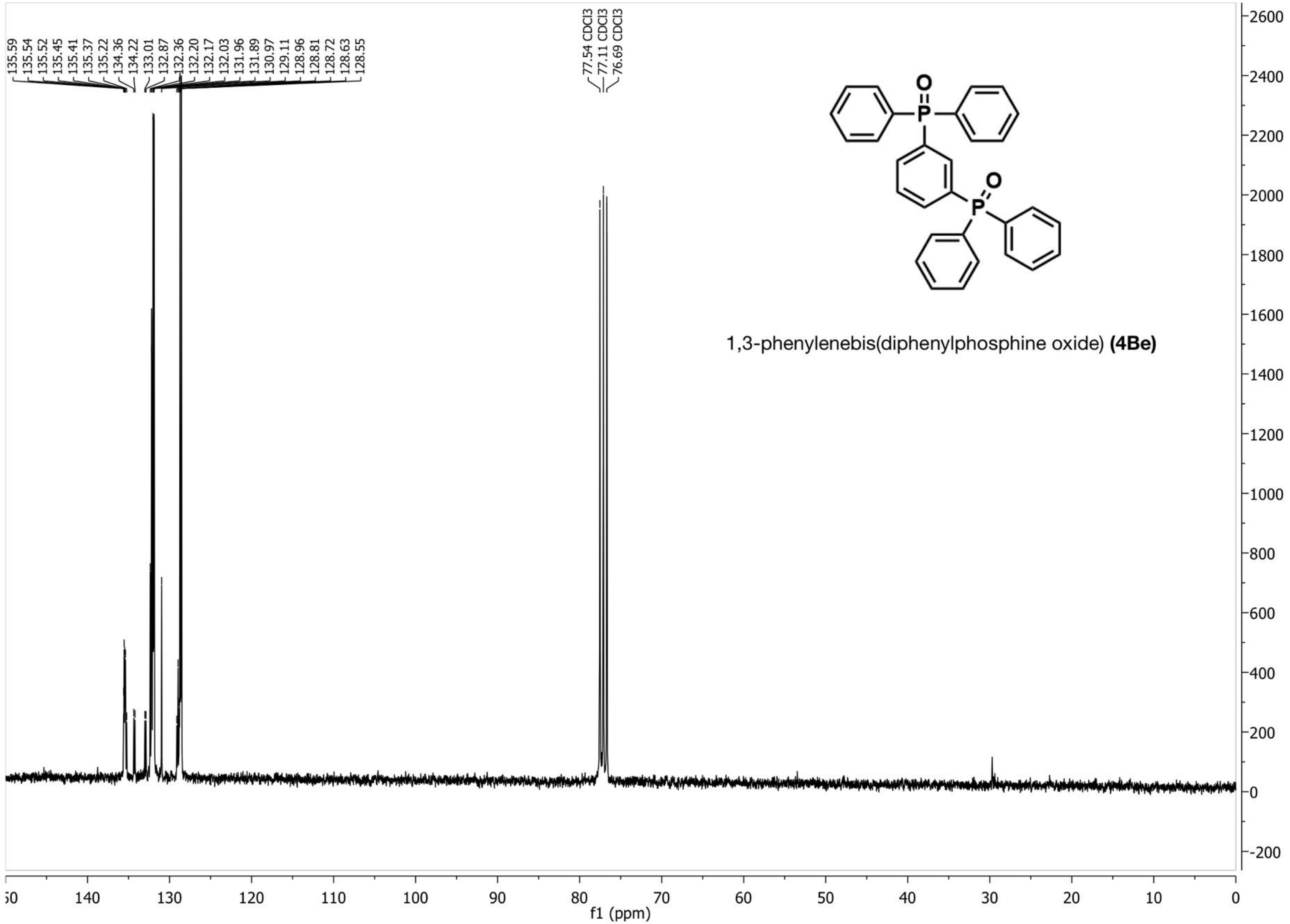

Figure S56. ^{13}C NMR ($CDCl_3$, 125.7 MHz) spectrum of 1,3-phenylenebis(diphenylphosphine oxide) (**4Be**).

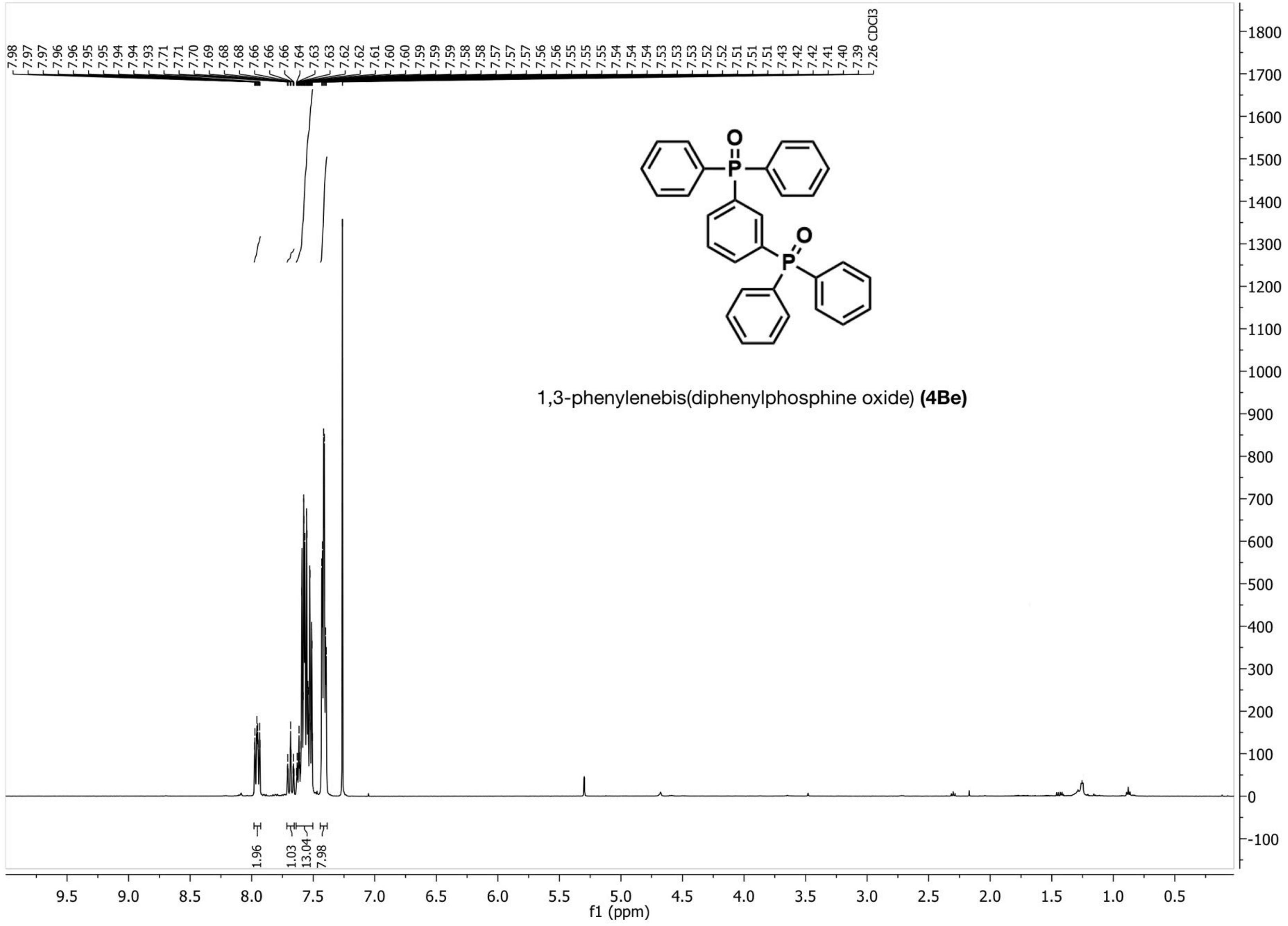

Figure S57. ^{1}H NMR ($CDCl_3$, 500 MHz) spectrum of 1,3-phenylenebis(diphenylphosphine oxide) (**4Be**).

2. Tables Containing XYZ Coordinates and total energies of the optimized geometries

Table S1. XYZ Coordinates of S_I_mono

39
scf done: -998.746855

H	-0.074037	0.072316	0.015417
O	-0.010424	-0.004663	3.403978
C	0.566607	0.047209	2.066933
Pd	1.230101	0.145107	5.148800
O	0.964241	-1.789365	6.139590
B	2.085697	-1.535552	7.173285
C	3.458395	-2.285064	6.783771
C	4.183231	-2.997071	7.756127
C	5.377961	-3.649983	7.447941
C	5.884934	-3.609488	6.146739
C	5.186892	-2.912125	5.159446
C	3.991908	-2.262506	5.480364
C	1.720865	1.998141	4.652823
C	3.067300	2.367782	4.517614
C	3.409741	3.679491	4.169779
C	2.413846	4.635076	3.961250
C	1.072081	4.274226	4.099227
C	0.724755	2.962799	4.441767
H	2.681342	5.654087	3.696409
O	2.204495	0.026863	6.937924
O	1.682640	-1.800908	8.496970
C	-0.523185	0.002262	1.011241
H	6.815087	-4.116994	5.904483
H	4.457206	3.951091	4.065136
H	3.857800	1.637500	4.675099
H	-0.323801	2.697489	4.548872
H	0.290202	5.013899	3.945194
H	3.122469	0.326011	6.940937
H	0.856243	-1.344252	8.691831
H	3.464110	-1.720761	4.694412
H	3.789955	-3.034285	8.767820
H	5.915362	-4.192558	8.221947
H	1.162663	-2.609996	5.673500
H	5.572493	-2.874619	4.143259
H	-0.546260	-0.806401	3.488350
H	1.113534	0.989763	2.046813
H	1.281866	-0.775386	1.953365
H	-1.087128	-0.937115	1.052433
H	-1.221011	0.834830	1.135031

Table S2. XYZ Coordinates of S_TS1_mono

39

scf done: -998.731530

C	-0.140700	-0.080877	-0.112926
C	-0.260726	-0.149711	1.280129
C	0.881179	-0.219187	2.082040
C	2.143354	-0.205964	1.490956
C	2.310599	-0.162577	0.084659
C	1.123264	-0.088745	-0.694340
H	-1.246442	-0.152659	1.738585
Pd	3.723745	1.246189	-0.656945
O	5.282976	2.496391	-1.631113
C	2.971098	2.774986	0.362057
C	3.424472	3.070892	1.657704
C	3.006759	4.234864	2.312404
C	2.119631	5.114646	1.688912
C	1.649248	4.821505	0.407324
C	2.066562	3.658021	-0.249645
H	1.791906	6.015882	2.199832
O	4.464900	-0.580225	-1.566919
B	3.665710	-1.353520	-0.568358
O	4.410037	-1.657064	0.604209
O	2.995947	-2.387377	-1.272649
H	3.371783	4.448695	3.314401
H	4.106226	2.392371	2.164854
H	1.683130	3.443390	-1.244162
H	0.950878	5.495071	-0.084054
H	4.104112	-0.766817	-2.444907
H	2.573018	-2.987732	-0.649352
H	3.029758	-0.271371	2.112894
H	1.201631	-0.074069	-1.778537
H	-1.031131	-0.028639	-0.733761
H	5.223777	-2.105193	0.341874
H	0.783542	-0.275091	3.162910
C	5.752064	3.783478	-1.156599
H	6.034993	1.891757	-1.708704
C	6.766832	4.377517	-2.119142
H	4.853661	4.396826	-1.085646
H	6.164351	3.672772	-0.147196
H	7.082878	5.364167	-1.764854
H	7.663877	3.751543	-2.195339
H	6.337436	4.490360	-3.118737

Table S3. XYZ Coordinates of S_II_mono

39

scf done: -998.776893

C	-0.306437	-1.081843	0.228023
C	0.261690	-0.088345	1.046842
C	1.635405	0.176067	0.908131
C	2.416473	-0.542902	-0.002589

C	1.840365	-1.528611	-0.806339
C	0.474808	-1.793320	-0.689361
Pd	-0.903190	0.760270	2.416369
B	-3.595710	1.533098	4.196655
O	-4.509655	2.544071	4.150646
C	-0.368608	2.582108	1.815580
C	0.164952	3.473953	2.770288
C	0.411373	4.817467	2.456177
C	0.132575	5.300886	1.178327
C	-0.386627	4.430151	0.216147
C	-0.629099	3.089778	0.528022
O	-1.695893	-1.216356	3.210763
C	-0.796681	-2.115130	3.889842
C	-1.481599	-3.428402	4.238797
O	-2.266104	1.826330	4.012678
O	-3.860882	0.198609	4.431890
H	0.324078	6.341567	0.930869
H	2.447670	-2.080917	-1.518628
H	3.478616	-0.324171	-0.087504
H	2.100140	0.950322	1.510438
H	-1.366407	-1.308969	0.307633
H	0.011769	-2.555110	-1.312960
H	-2.083599	2.752655	3.788337
H	-5.430855	2.302213	4.285863
H	0.419346	3.115553	3.766543
H	-1.018117	2.428188	-0.239985
H	-0.600308	4.795355	-0.785920
H	-4.789589	-0.035391	4.521454
H	0.829331	5.479525	3.211424
H	-2.437080	-0.978562	3.795311
H	0.025626	-2.272798	3.189791
H	-0.390960	-1.628692	4.786662
H	-0.770524	-4.109162	4.718866
H	-2.314006	-3.272765	4.935316
H	-1.870024	-3.913281	3.338441

Table S4. XYZ Coordinates of S_III_mono

32

scf done: -746.267353

C	-0.224254	-0.235245	0.489701
C	-0.084213	-0.374377	1.879553
C	1.205006	-0.479669	2.425304
C	2.328739	-0.475403	1.590350
C	2.181874	-0.353801	0.207716
C	0.903210	-0.230790	-0.339132
Pd	-1.689099	-0.620242	3.022454
O	-1.709618	-2.884933	2.774194
C	-2.288020	-3.457962	1.573891
C	-3.784601	-3.217249	1.600056
C	-1.781332	1.318844	3.398475

C	-1.576935	1.751194	4.722930
C	-1.836474	3.075126	5.093296
C	-2.284355	3.995143	4.144164
C	-2.468749	3.585914	2.821441
C	-2.210394	2.263740	2.449647
H	-2.479634	5.025072	4.430372
H	3.056096	-0.345270	-0.437164
H	1.340226	-0.551156	3.500419
H	-1.209962	-0.122086	0.046529
H	3.320658	-0.557380	2.028090
H	0.777851	-0.123808	-1.413947
H	-1.213165	1.055235	5.475251
H	-2.344701	1.968850	1.412619
H	-1.679192	3.386839	6.123140
H	-2.808863	4.298908	2.074108
H	-0.763211	-3.087726	2.788445
H	-2.065634	-4.531655	1.563481
H	-1.827856	-2.992623	0.694553
H	-4.246150	-3.648583	0.706294
H	-4.237961	-3.679340	2.481865
H	-4.001944	-2.144330	1.615071

Table S5. XYZ Coordinates of S_TS2_mono

32

scf done: -746.252218

C	-0.243774	0.192870	0.169954
C	-0.151178	0.250514	1.562396
C	1.093743	0.189898	2.190813
C	2.276478	0.050420	1.438446
C	2.169021	0.013236	0.034390
C	0.922139	0.074462	-0.589969
Pd	3.804296	-0.973429	2.287230
O	4.960434	-2.714949	3.099188
H	-1.212662	0.250090	-0.317876
C	3.889999	1.057724	2.158927
C	3.656472	1.799844	3.332865
C	4.273626	3.035969	3.533328
C	5.123501	3.569077	2.561654
C	5.348667	2.852766	1.384040
C	4.733698	1.616231	1.179286
H	5.595429	4.535291	2.715347
H	2.983820	1.414636	4.093108
H	4.906884	1.087172	0.247054
H	4.082999	3.586349	4.451408
H	6.001394	3.259400	0.615449
H	1.144317	0.256725	3.273401
H	3.064047	-0.057570	-0.576364
H	-1.051063	0.349005	2.164434
H	0.863527	0.035318	-1.674862
C	6.260120	-3.116848	2.599515

H	4.997209	-2.632038	4.060510
H	6.579371	-4.010492	3.149626
C	6.136693	-3.409921	1.118117
H	6.982928	-2.312478	2.781875
H	7.112636	-3.698048	0.714850
H	5.430562	-4.226039	0.940728
H	5.785402	-2.522117	0.582845

Table S6. XYZ Coordinates of S_IV_mono

32

scf done: -746.301160

C	1.437135	1.180960	0.134710
C	1.135165	0.579788	1.368166
C	2.006373	-0.417613	1.845501
C	3.124530	-0.806123	1.111257
C	3.403565	-0.208497	-0.120694
C	2.554201	0.787666	-0.603173
Pd	-1.518806	-0.590503	1.917674
O	-2.536242	-2.578383	1.836929
C	-3.796484	-2.825467	2.504209
C	-3.615705	-2.559024	3.984941
C	-0.050159	1.015985	2.171034
C	0.087062	1.216064	3.584725
C	-0.846570	1.936903	4.308267
C	-1.984442	2.479981	3.675836
C	-2.178625	2.275380	2.320757
C	-1.232394	1.544534	1.548539
H	-2.702835	3.056945	4.250977
H	4.274735	-0.512708	-0.693652
H	1.787725	-0.908406	2.789040
H	0.803020	1.977616	-0.242174
H	3.776934	-1.583760	1.499215
H	2.766062	1.271605	-1.552828
H	0.984948	0.854123	4.076202
H	-1.295819	1.603142	0.465097
H	-0.690177	2.098788	5.371219
H	-3.042889	2.701797	1.818818
H	-2.616959	-2.811033	0.903318
H	-4.083415	-3.869852	2.326675
H	-4.568689	-2.170816	2.081550
H	-4.563612	-2.716569	4.509466
H	-2.864678	-3.230441	4.410936
H	-3.290481	-1.526033	4.144905

Table S7. XYZ Coordinates of S_I_para_P(O)PH$_2$

62

scf done: -1878.050884

C	4.298794	1.974446	-0.066676
C	4.541320	1.026049	-1.071160
C	5.455671	1.324443	-2.092252

C	6.131279	2.545066	-2.096400
C	5.897830	3.478246	-1.084029
C	4.979179	3.193439	-0.071471
P	3.724475	-0.613053	-1.155099
O	3.702359	-1.163166	-2.554709
C	2.048229	-0.395766	-0.460756
C	0.994781	-0.276259	-1.378209
C	-0.316975	-0.095704	-0.931195
C	-0.599457	-0.023695	0.441442
C	0.453904	-0.155812	1.358957
C	1.764053	-0.348489	0.912032
Pd	-2.454626	0.233323	1.080192
O	-1.883516	2.083493	2.003808
C	-1.496255	3.229797	1.191326
C	-1.059980	4.387103	2.071168
O	-3.259824	-1.523221	0.430959
B	-4.792421	-1.177696	0.652917
O	-4.566989	0.034174	1.590429
C	-5.496460	-0.754466	-0.732455
C	-6.573552	-1.501111	-1.242384
C	-7.198535	-1.159766	-2.442955
C	-6.760242	-0.052017	-3.172678
C	-5.694171	0.709515	-2.691167
C	-5.076800	0.357796	-1.487900
O	-5.446883	-2.248869	1.289738
C	4.629821	-1.698304	0.009891
C	5.262339	-1.244181	1.176236
C	5.913292	-2.146575	2.019322
C	5.944822	-3.505350	1.699346
C	5.330769	-3.961484	0.530794
C	4.677392	-3.062955	-0.312590
H	-7.244926	0.215097	-4.108105
H	1.209681	-0.345605	-2.440742
H	-1.116584	-0.012612	-1.663625
H	0.256048	-0.124900	2.427142
H	2.558178	-0.484996	1.641491
H	-3.070744	-1.762390	-0.485448
H	-5.004208	-2.469074	2.117327
H	-4.243381	0.964672	-1.131333
H	-6.919186	-2.361025	-0.676169
H	-8.029021	-1.756953	-2.811446
H	-5.194556	0.724706	1.346123
H	-5.344937	1.573451	-3.251715
H	-2.593127	2.346377	2.607639
H	-0.679078	2.857934	0.573112
H	-2.331762	3.504351	0.537467
H	-0.743637	5.226784	1.444211
H	-1.879498	4.741812	2.707215
H	-0.221655	4.097260	2.710459
H	5.613704	0.598833	-2.884218

H	6.836145	2.769706	-2.891816
H	6.423940	4.428761	-1.088209
H	4.786788	3.923182	0.710168
H	3.567537	1.771241	0.710403
H	4.214411	-3.403366	-1.233705
H	5.365124	-5.016285	0.273672
H	6.454950	-4.205627	2.354770
H	6.403395	-1.787274	2.919804
H	5.266696	-0.185725	1.419393

Table S8. XYZ Coordinates of S_TS1_para_P(O)PH_2

62

scf done: -1878.037260

C	3.385055	-2.339136	-0.355266
C	4.041236	-1.498151	0.555181
C	5.148963	-1.983339	1.266194
C	5.605121	-3.284295	1.053722
C	4.957830	-4.111614	0.133080
C	3.846369	-3.639614	-0.568083
P	3.532109	0.229922	0.890293
C	4.314245	1.246068	-0.420446
C	4.705732	2.543887	-0.059955
C	5.296882	3.387070	-1.001210
C	5.505355	2.940616	-2.308037
C	5.130779	1.645040	-2.670409
C	4.541951	0.798087	-1.729768
C	1.732062	0.306369	0.603313
C	1.141560	0.491994	-0.655301
C	-0.249503	0.521175	-0.788881
C	-1.084808	0.386151	0.332210
C	-0.487164	0.195779	1.589811
C	0.902526	0.163551	1.726293
Pd	-3.044714	0.652616	0.188185
O	-5.179620	0.748088	-0.147532
B	-5.087518	-0.549957	-0.887181
O	-4.879580	-0.364444	-2.281259
C	-3.460289	-1.374398	-0.320917
C	-2.699200	-1.926672	-1.381379
C	-2.088113	-3.174536	-1.275615
C	-2.185573	-3.895760	-0.082590
C	-2.906078	-3.375966	0.999038
C	-3.531502	-2.139163	0.876092
O	-2.927470	2.793328	0.756164
C	-1.743005	3.629423	0.696323
C	-2.034864	5.018830	1.237024
O	-6.150947	-1.370054	-0.429230
O	3.927806	0.685131	2.268860
H	-1.703531	-4.866100	0.005579
H	1.762274	0.629818	-1.536730
H	-0.680673	0.660328	-1.777130

H	-1.106105	0.081158	2.476223
H	1.354626	0.043368	2.706902
H	-5.718419	0.602975	0.642874
H	-6.210298	-2.165093	-0.969741
H	-2.633987	-1.371537	-2.311191
H	-4.121130	-1.764858	1.709122
H	-2.980425	-3.940162	1.924690
H	-5.591046	0.189154	-2.626513
H	-1.532073	-3.584683	-2.114470
H	-3.634370	3.204625	0.238144
H	-1.003392	3.107058	1.303289
H	-1.372260	3.662487	-0.334453
H	-1.122442	5.623586	1.218271
H	-2.787063	5.537179	0.630508
H	-2.394863	4.968837	2.268542
H	4.558279	2.870686	0.964915
H	5.601000	4.389600	-0.713547
H	5.968023	3.596691	-3.040083
H	5.305924	1.290036	-3.682107
H	4.278783	-0.216840	-2.013208
H	5.631059	-1.338238	1.994393
H	6.461754	-3.654400	1.609914
H	5.312806	-5.125301	-0.030736
H	3.332115	-4.286446	-1.273384
H	2.503322	-1.988080	-0.883631

Table S9. XYZ Coordinates of S_II_para_P(O)PH$_2$

62

scf done: -1878.081810

C	-5.709941	-1.420501	-0.554786
C	-4.434525	-1.557432	0.013280
C	-3.777579	-2.795207	-0.079631
C	-4.379184	-3.867767	-0.737187
C	-5.645988	-3.718467	-1.306101
C	-6.310888	-2.494738	-1.212423
P	-3.687252	-0.211746	1.007513
C	-4.387351	1.347618	0.333475
C	-5.074508	2.171083	1.235360
C	-5.621249	3.382777	0.807150
C	-5.484364	3.780188	-0.523558
C	-4.798198	2.964854	-1.428248
C	-4.251312	1.754629	-1.002419
C	-1.912678	-0.215650	0.595556
C	-1.013478	0.039494	1.640529
C	0.360287	0.097945	1.394569
C	0.878547	-0.079963	0.099367
C	-0.030436	-0.359076	-0.938350
C	-1.404055	-0.424221	-0.696158
Pd	2.805719	0.210624	-0.272283
O	5.095654	0.471227	-0.650362

B	5.902424	1.328544	0.061079
O	7.075355	0.855086	0.569874
C	3.149819	-1.735176	-0.010858
C	2.952713	-2.419271	1.202210
C	3.394056	-3.734619	1.370236
C	4.035287	-4.406899	0.326415
C	4.226221	-3.753667	-0.890766
C	3.785187	-2.434216	-1.058750
O	2.679247	2.445638	-0.650686
C	1.634719	3.292387	-0.134247
C	1.707999	4.688765	-0.734191
O	-3.937718	-0.309491	2.489503
O	5.421866	2.617288	0.170122
H	4.373660	-5.430923	0.459046
H	-1.403079	0.175650	2.645622
H	1.032680	0.284975	2.228439
H	0.335141	-0.539235	-1.945299
H	-2.074398	-0.665682	-1.517497
H	5.394204	-0.451970	-0.643148
H	7.612019	1.474152	1.073989
H	2.440829	-1.924775	2.022490
H	3.917518	-1.958312	-2.029222
H	4.708882	-4.269562	-1.717838
H	5.980034	3.234447	0.652474
H	3.231698	-4.238060	2.320564
H	3.548585	2.788631	-0.378985
H	0.701730	2.794862	-0.402579
H	1.688661	3.326600	0.961901
H	0.883260	5.304163	-0.359376
H	2.645709	5.191021	-0.468347
H	1.639778	4.645017	-1.825036
H	-5.166924	1.845490	2.267016
H	-6.153335	4.015003	1.512590
H	-5.909758	4.722688	-0.857378
H	-4.689560	3.272320	-2.464663
H	-3.718158	1.130800	-1.713792
H	-2.791640	-2.917356	0.358638
H	-3.858861	-4.818795	-0.806190
H	-6.113429	-4.553830	-1.820006
H	-7.297424	-2.375272	-1.651402
H	-6.234094	-0.472453	-0.485537

Table S10. XYZ Coordinates of S_III_para_P(O)PH$_2$

55

scf done: -1625.572673

C	-0.497546	-0.494817	0.536009
C	-1.267415	-0.350857	-0.631698
C	-0.589610	-0.214860	-1.859118
C	0.805272	-0.251183	-1.920566
C	1.562732	-0.402406	-0.750349

C	0.896911	-0.533446	0.477778
Pd	-3.231576	-0.570896	-0.695818
O	-5.446045	-0.966537	-0.980948
C	-6.210102	-1.605868	0.074567
C	-5.717240	-3.031052	0.228508
C	-3.493958	1.037327	0.437818
C	-3.723642	0.888786	1.814517
C	-4.068045	1.996660	2.597411
C	-4.176773	3.262656	2.019359
C	-3.933477	3.417953	0.653648
C	-3.586999	2.314868	-0.135466
P	3.378751	-0.397255	-0.934447
H	-4.440231	4.121903	2.629590
H	-3.378996	2.457982	-1.191372
H	-3.629679	-0.086202	2.285337
H	-4.002394	4.401784	0.196096
H	-4.245189	1.866015	3.662229
H	-1.148785	-0.083805	-2.782788
H	-0.986940	-0.586697	1.501185
H	1.315957	-0.172012	-2.876088
H	1.463002	-0.680269	1.393803
H	-5.803728	-0.079709	-1.129825
H	-7.268771	-1.586886	-0.209601
H	-6.082597	-1.039741	1.004411
H	-6.284375	-3.536802	1.016147
H	-5.844059	-3.589920	-0.703220
H	-4.657987	-3.045188	0.504930
C	4.041588	-1.447837	0.413756
O	3.833911	-0.861437	-2.291275
C	3.933855	1.309346	-0.561962
C	5.097017	1.752250	-1.209336
C	5.590487	3.034423	-0.968113
C	4.925375	3.885518	-0.082501
C	3.759484	3.456205	0.554732
C	3.261281	2.174428	0.313314
H	5.593146	1.089717	-1.912011
H	6.490194	3.371612	-1.475106
H	5.309170	4.884841	0.103270
H	3.231401	4.121817	1.231723
H	2.338988	1.857889	0.791725
C	4.377259	-2.767220	0.075723
C	4.875239	-3.636537	1.046474
C	5.045861	-3.195151	2.360502
C	4.726525	-1.879125	2.701164
C	4.230516	-1.006092	1.731270
H	4.259755	-3.092059	-0.953577
H	5.135372	-4.656189	0.776573
H	5.435746	-3.872046	3.115642
H	4.872273	-1.529008	3.719236
H	4.009455	0.022994	1.999159

Table S11. XYZ Coordinates of S_TS2_para_P(O)PH$_2$

55

scf done: -1625.557371

C	1.342928	0.173868	0.581001
C	0.846261	0.544179	-0.678014
C	-0.526922	0.583760	-0.916130
C	-1.449650	0.245018	0.094049
C	-0.941292	-0.087700	1.366181
C	0.430783	-0.131423	1.603188
Pd	-3.192400	-0.609446	-0.469917
O	-4.577686	-2.185012	-1.248828
P	3.116732	0.062408	0.991664
C	-3.156135	1.345694	0.105694
C	-3.243959	2.325513	-0.901618
C	-3.841488	3.561105	-0.646247
C	-4.347642	3.853462	0.622137
C	-4.249042	2.897551	1.635525
C	-3.652630	1.660429	1.385029
H	-4.802929	4.819195	0.821683
H	-2.839507	2.127067	-1.889414
H	-3.567567	0.940612	2.193265
H	-3.904544	4.299566	-1.441547
H	-4.630837	3.115843	2.629643
H	-0.881705	0.893608	-1.894226
H	-1.622257	-0.306876	2.182823
H	1.529149	0.825050	-1.475181
H	0.808283	-0.383300	2.589997
C	-5.657190	-2.767284	-0.474907
H	-4.910940	-1.914742	-2.113797
H	-6.132775	-3.547055	-1.081930
C	-5.078248	-3.350728	0.797723
H	-6.399451	-1.992040	-0.250082
H	-5.880278	-3.781241	1.405514
H	-4.352181	-4.136420	0.570660
H	-4.578199	-2.571567	1.381675
O	3.393122	0.255030	2.457875
C	3.686873	-1.572461	0.389225
C	3.970524	1.309128	-0.045722
C	4.333308	1.100214	-1.384161
C	4.966764	2.112687	-2.107054
C	5.250428	3.336034	-1.496472
C	4.906142	3.544585	-0.159092
C	4.270691	2.535804	0.565098
H	4.140299	0.142849	-1.859394
H	5.246843	1.942667	-3.142883
H	5.746959	4.121503	-2.059386
H	5.137177	4.491109	0.321350
H	4.016451	2.678802	1.610880
C	4.732895	-2.178518	1.100906

C	5.231361	-3.417597	0.698268
C	4.687743	-4.062956	-0.414753
C	3.637319	-3.471827	-1.119774
C	3.134138	-2.233102	-0.717577
H	5.132762	-1.677069	1.976957
H	6.039815	-3.882029	1.255844
H	5.075275	-5.028867	-0.726632
H	3.202740	-3.978690	-1.976828
H	2.299585	-1.792319	-1.255179

Table S12. XYZ Coordinates of S_IV_para_P(O)PH$_2$

55

scf done: -1625.606546

C	1.014579	-1.279439	0.976303
C	1.994506	-1.107967	-0.056698
C	1.515190	-0.717163	-1.354255
C	0.124228	-0.512485	-1.566428
C	-0.797884	-0.707311	-0.543658
C	-0.334991	-1.107951	0.734540
C	3.373837	-1.650746	0.144342
C	4.032637	-1.508902	1.380232
C	5.306759	-2.035357	1.579801
C	5.957154	-2.719522	0.549481
C	5.313115	-2.876261	-0.678288
C	4.035247	-2.352727	-0.877255
Pd	2.266962	1.057635	-0.390950
O	3.028941	3.118609	0.005228
C	2.403242	4.063014	0.906001
C	0.982469	4.305113	0.438903
P	-2.551161	-0.433296	-0.956336
H	6.950860	-3.129466	0.704644
H	3.538794	-2.508092	-1.830002
H	3.547857	-0.960059	2.182197
H	5.800626	-3.418094	-1.484072
H	5.795798	-1.907174	2.541724
H	2.159933	-0.799490	-2.225849
H	1.340897	-1.635153	1.948793
H	-0.232069	-0.251623	-2.558363
H	-1.044381	-1.320266	1.529083
H	3.969504	3.050090	0.211318
H	2.983971	4.993874	0.887758
H	2.418156	3.659934	1.926486
H	0.483800	5.006236	1.115796
H	0.972842	4.728070	-0.569555
H	0.416103	3.368928	0.427098
C	-3.502664	-1.691293	-0.021577
C	-3.020196	1.189664	-0.243123
O	-2.819931	-0.498383	-2.435247
C	-3.977158	1.934054	-0.948279
C	-4.400826	3.172595	-0.465248

C	-3.871776	3.679025	0.723780
C	-2.909000	2.949378	1.424759
C	-2.479189	1.711644	0.941484
H	-4.367078	1.537726	-1.880795
H	-5.140711	3.743952	-1.018644
H	-4.203166	4.642927	1.099957
H	-2.488524	3.345287	2.345116
H	-1.712648	1.162631	1.480577
C	-3.860937	-2.853202	-0.721701
C	-4.569239	-3.869887	-0.081227
C	-4.929580	-3.733956	1.261316
C	-4.588791	-2.574452	1.960520
C	-3.881920	-1.554153	1.321465
H	-3.594021	-2.938881	-1.770597
H	-4.844767	-4.765459	-0.630895
H	-5.482860	-4.525403	1.759122
H	-4.881068	-2.459469	3.000533
H	-3.645650	-0.644880	1.866332

Table S13. XYZ Coordinates of S_I_para_Me

42

scf done: -1038.066454

H	-0.044453	0.000648	-0.002672
C	-0.035771	-0.009129	1.084586
C	1.179072	0.007628	1.772023
C	1.232841	-0.004424	3.177254
C	0.002519	-0.037010	3.862264
C	-1.220045	-0.053921	3.185484
C	-1.241359	-0.039698	1.789874
B	2.651398	0.022084	3.942161
O	2.784287	1.313951	4.848838
Pd	2.341280	0.472745	6.654684
O	2.050855	-0.712429	8.420583
C	0.769168	-0.716183	9.113578
C	0.840889	-1.568095	10.367928
C	2.104989	2.127992	7.715334
C	1.152713	3.087832	7.345020
C	0.991199	4.255431	8.097936
C	1.776812	4.504848	9.230110
C	2.726048	3.540597	9.593409
C	2.892072	2.367925	8.850329
C	1.627135	5.786703	10.016655
O	2.718954	-1.012602	5.089011
O	3.743690	-0.068018	3.056469
H	-2.188487	-0.052615	1.256732
H	0.241029	4.983560	7.795851
H	0.520116	2.931206	6.474078
H	3.639858	1.642836	9.161098
H	3.349841	3.708118	10.469393
H	2.198331	2.024084	4.558127

H	4.572864	-0.058387	3.548218
H	-0.003184	-0.045299	4.952988
H	2.114435	0.027505	1.220397
H	2.075161	-1.707315	4.907643
H	-2.152766	-0.077855	3.744242
H	2.300845	-1.623476	8.209497
H	0.585863	0.333139	9.344369
H	-0.009208	-1.064596	8.425158
H	-0.120314	-1.535150	10.890738
H	1.051159	-2.617971	10.131865
H	1.615879	-1.200149	11.045777
H	1.867210	5.639214	11.074313
H	2.299397	6.567268	9.638136
H	0.607651	6.179615	9.953342

Table S14. XYZ Coordinates of S_TS1_para_Me

42

scf done: -1038.050941

C	-0.000818	0.014512	-0.000539
C	-0.001257	-0.004198	1.421323
C	1.266228	-0.015782	2.054479
C	2.453466	0.032679	1.325850
C	2.409769	0.054648	-0.070352
C	1.179056	0.042038	-0.737327
Pd	-1.412513	-1.446059	2.099666
O	-3.097725	-2.728102	2.779279
C	-2.975144	-4.061292	3.335210
C	-4.344505	-4.663468	3.602674
B	-1.278644	1.146469	2.272646
O	-1.487439	2.242566	1.395738
C	-0.164338	-2.980467	1.926137
C	0.668942	-3.362033	2.988846
C	1.428874	-4.533821	2.918527
C	1.397457	-5.354505	1.783782
C	0.574142	-4.964636	0.719704
C	-0.189493	-3.794200	0.782940
C	2.251066	-6.598949	1.695746
O	-2.560537	0.381689	2.342514
O	-0.704745	1.360571	3.555639
H	3.334239	0.085406	-0.641567
H	2.061444	-4.810276	3.760394
H	0.729328	-2.747299	3.883857
H	-0.811513	-3.523685	-0.066960
H	0.530512	-5.581555	-0.176244
H	-3.094396	0.625682	1.573576
H	-0.728222	2.834606	1.425251
H	1.300005	-0.021388	3.138757
H	-0.949979	0.044383	-0.529711
H	1.148664	0.060707	-1.823505
H	-1.369513	1.771065	4.122513

H	3.410898	0.045283	1.839809
H	-3.567908	-2.162150	3.408311
H	-2.423980	-4.625562	2.582773
H	-2.362424	-4.024111	4.243157
H	-4.234497	-5.682580	3.987703
H	-4.898961	-4.085903	4.351900
H	-4.939710	-4.702646	2.685881
H	1.786833	-7.360364	1.060789
H	3.237542	-6.378975	1.267387
H	2.419907	-7.040722	2.683040

Table S15. XYZ Coordinates of S_II_para_Me

42

scf done: -1038.099214

C	-0.215951	-1.053394	-0.062490
C	-0.040648	-1.387450	1.285797
C	1.053277	-0.909714	2.015182
C	2.018445	-0.081158	1.416068
Pd	3.626628	0.386502	2.490016
C	3.245228	2.316817	2.186396
B	6.711292	1.049919	2.819023
O	6.681026	0.638792	1.523084
C	2.445707	3.016767	3.108449
C	2.284566	4.403297	3.016936
C	2.909588	5.120309	1.995006
C	3.692988	4.438733	1.062537
C	3.853978	3.051683	1.153909
C	0.743029	-0.222930	-0.656614
C	1.833446	0.265209	0.068445
O	4.150027	-1.770446	3.054761
C	4.117805	-2.879605	2.127084
C	4.316598	-4.206124	2.842571
O	5.619667	0.763835	3.645697
O	7.777109	1.694439	3.368388
C	-1.415972	-1.542130	-0.840641
H	2.780727	6.196748	1.921320
H	0.632673	0.055617	-1.703240
H	2.540374	0.925449	-0.424640
H	1.152908	-1.196229	3.059622
H	-0.769988	-2.029238	1.777271
H	5.609384	1.285450	4.458516
H	8.454640	1.891916	2.711098
H	4.459810	2.546654	0.405258
H	1.932607	2.476939	3.900055
H	1.660054	4.920605	3.741680
H	5.778196	0.374219	1.264521
H	4.178533	4.983990	0.256363
H	5.014019	-1.735368	3.488594
H	3.133162	-2.818652	1.661742
H	4.873997	-2.728933	1.346024

H	4.251901	-5.031980	2.126423
H	5.301192	-4.260703	3.322206
H	3.550192	-4.352475	3.609080
H	-1.797993	-2.485941	-0.438643
H	-2.239757	-0.817519	-0.803698
H	-1.173122	-1.699182	-1.896640

Table S16. XYZ Coordinates of S_III_para_Me

35

scf done: -785.587022

C	0.022207	-0.018731	-0.004641
C	0.017563	-0.010759	1.399242
C	1.244320	-0.022251	2.082128
C	2.449348	-0.070621	1.371145
C	2.446446	-0.095189	-0.024361
C	1.230228	-0.066095	-0.709313
Pd	-1.693425	-0.185083	2.391254
C	-1.863318	1.777724	2.550607
C	-1.779078	2.361709	3.827979
C	-2.096909	3.709006	4.025480
C	-2.489901	4.525528	2.958435
C	-2.546319	3.951116	1.680946
C	-2.230976	2.606652	1.476175
C	-2.856324	5.975210	3.176586
O	-1.637171	-2.464175	2.376382
C	-2.092306	-3.171892	1.195396
C	-3.590564	-2.978564	1.067582
H	3.383294	-0.127135	-0.573485
H	1.267144	0.020317	3.167007
H	-0.913386	0.019530	-0.555987
H	3.391123	-0.078697	1.914541
H	1.216559	-0.073224	-1.796601
H	-1.460232	1.768248	4.681702
H	-2.269127	2.204317	0.467517
H	-2.027511	4.132747	5.025650
H	-2.833639	4.567644	0.831055
H	-0.690074	-2.629252	2.489889
H	-1.844673	-4.233623	1.313239
H	-1.566923	-2.781569	0.316079
H	-3.960512	-3.512043	0.186428
H	-4.109499	-3.364474	1.949882
H	-3.833463	-1.917132	0.953750
H	-3.939644	6.099134	3.304384
H	-2.376052	6.379529	4.072970
H	-2.559815	6.596715	2.325394

Table S17. XYZ Coordinates of S_TS2_para_Me

35

scf done: -785.572012

H	0.114904	0.103519	-0.071857

C	0.071906	0.053001	1.020724
C	1.476160	0.053777	1.589847
O	1.386989	0.010308	3.035647
Pd	0.307777	1.557399	4.250586
C	0.434831	3.534437	4.722013
C	1.456491	3.936508	5.604192
C	2.024641	5.208302	5.510117
C	1.576821	6.114848	4.546597
C	0.551209	5.736025	3.677536
C	-0.019972	4.465291	3.767838
C	-1.013414	2.368886	5.552771
C	-0.845362	2.074607	6.919279
C	-1.942930	1.990811	7.775745
C	-3.246806	2.217379	7.314862
C	-3.413316	2.526323	5.958117
C	-2.321990	2.612067	5.093936
C	-4.427249	2.169718	8.255772
H	2.013874	7.107123	4.480951
H	1.806531	3.255935	6.374357
H	-0.830460	4.200399	3.095579
H	2.816990	5.492276	6.198419
H	0.186981	6.434091	2.927693
H	0.149896	1.909393	7.321160
H	-2.493559	2.872791	4.053727
H	-1.781646	1.748536	8.824362
H	-4.413926	2.707646	5.570528
H	2.271104	-0.097742	3.408610
H	2.035209	-0.827589	1.252369
H	2.021791	0.955739	1.287403
H	-0.463379	-0.856588	1.307594
H	-0.488091	0.918336	1.389100
H	-5.341144	1.863664	7.736784
H	-4.255310	1.469013	9.078970
H	-4.623273	3.152807	8.703418

Table S18. XYZ Coordinates of S_IV_para_Me

35

scf done: -785.621035

H	0.262752	-1.421658	0.601510
C	0.861426	-0.900751	1.359732
O	1.005743	-1.772044	2.505901
Pd	2.133805	-1.147090	4.332287
C	2.712296	-0.594526	6.371406
C	3.879798	-0.601704	5.537232
C	4.786127	-1.698089	5.583716
C	4.571967	-2.791100	6.409430
C	3.401278	-2.802203	7.207317
C	2.498611	-1.756948	7.184505
C	5.538560	-3.949541	6.458864
C	0.173978	0.372448	1.809827

C	1.947009	0.659522	6.654562
C	2.582288	1.912986	6.659199
C	1.871491	3.079425	6.943860
C	0.509939	3.021106	7.243223
C	-0.133224	1.780557	7.256813
C	0.574711	0.616534	6.966455
H	0.075557	1.061287	0.964695
H	-0.042645	3.928684	7.468471
H	3.647373	1.973862	6.457577
H	0.056139	-0.337399	6.955441
H	2.388844	4.035121	6.943544
H	-1.193319	1.719571	7.488009
H	4.210813	0.315340	5.056695
H	1.642506	-1.775490	7.852123
H	5.683493	-1.652616	4.971014
H	3.216154	-3.648199	7.865447
H	1.383552	-2.614367	2.223337
H	1.850645	-0.682647	0.938520
H	-0.823528	0.156562	2.203000
H	0.759506	0.859212	2.596476
H	5.092995	-4.860266	6.038504
H	6.449120	-3.735207	5.891966
H	5.831568	-4.184844	7.488730

Table S19. XYZ Coordinates of S_I_para_OMe

43

scf done: -1113.269287

H	-0.114373	-0.120966	-0.011182
C	-0.091676	-0.094503	1.075597
C	1.132002	-0.096106	1.747371
C	1.203857	-0.062721	3.151455
C	-0.017722	-0.030022	3.851966
C	-1.248967	-0.027865	3.190914
C	-1.288187	-0.060035	1.795989
B	2.632411	-0.060179	3.897820
O	2.818938	1.251318	4.765853
Pd	2.373195	0.475640	6.599238
O	2.069006	-0.647176	8.402809
C	0.794340	-0.592135	9.106372
C	0.856410	-1.398572	10.390974
C	2.202411	2.166709	7.617803
C	1.259769	3.141129	7.246336
C	1.143550	4.332873	7.958650
C	1.977194	4.582997	9.057861
C	2.923797	3.625263	9.437262
C	3.028157	2.427460	8.715834
O	1.786408	5.783266	9.687047
C	2.621372	6.100666	10.786723
O	2.681739	-1.064229	5.072837
O	3.709702	-0.210667	3.001979

H	-2.242164	-0.058418	1.275049
H	0.414949	5.087323	7.677763
H	0.600077	2.975336	6.397703
H	3.773005	1.698521	9.024582
H	3.585328	3.795299	10.279313
H	2.253285	1.972579	4.462623
H	4.544974	-0.214119	3.483369
H	-0.009366	-0.001365	4.942345
H	2.060148	-0.126631	1.184165
H	2.011566	-1.740673	4.920189
H	-2.174504	-0.000854	3.761341
H	2.289746	-1.571177	8.216829
H	0.640090	0.469498	9.298689
H	0.000676	-0.945762	8.438344
H	-0.098598	-1.321675	10.920497
H	1.037942	-2.461500	10.192477
H	1.647012	-1.025803	11.047853
H	2.312698	7.091070	11.125696
H	2.499730	5.385184	11.611175
H	3.680249	6.133070	10.497961

Table S20. XYZ Coordinates of S_TS1_para_OMe

43

scf done: -1113.253801

C	-0.035469	0.044351	-0.003943
C	-0.015815	0.004568	1.417360
C	1.260371	-0.035460	2.031815
C	2.437738	0.005805	1.286853
C	2.374296	0.049978	-0.108003
C	1.134037	0.065935	-0.757205
Pd	-1.430942	-1.430086	2.098943
O	-3.113892	-2.706143	2.802131
C	-2.982131	-4.042285	3.348332
C	-4.345676	-4.634894	3.662838
B	-1.265247	1.157410	2.302225
O	-1.476144	2.267634	1.443314
C	-0.199507	-2.975437	1.887743
C	0.629830	-3.392803	2.944846
C	1.381599	-4.562549	2.854586
C	1.336154	-5.343125	1.691107
C	0.532798	-4.936544	0.621195
C	-0.219641	-3.756694	0.726555
O	2.112229	-6.473688	1.700925
O	-2.554827	0.406167	2.382697
O	-0.669326	1.348803	3.578901
H	3.290895	0.075809	-0.692020
H	2.019305	-4.887088	3.671752
H	0.694784	-2.800325	3.854297
H	-0.829134	-3.457061	-0.122457
H	0.485632	-5.514746	-0.295062

H	-3.097728	0.665244	1.625151
H	-0.709652	2.850460	1.468015
H	1.309421	-0.057607	3.115261
H	-0.991673	0.096587	-0.518527
H	1.088521	0.102351	-1.842436
H	-1.319064	1.763896	4.159697
H	3.402470	-0.004470	1.786961
H	-3.562949	-2.141151	3.447353
H	-2.462864	-4.607851	2.574532
H	-2.336325	-4.013511	4.233277
H	-4.230039	-5.656458	4.039609
H	-4.867950	-4.056234	4.434042
H	-4.974152	-4.665343	2.768145
C	2.123216	-7.281829	0.538850
H	2.800417	-8.111243	0.751382
H	1.126325	-7.682833	0.309904
H	2.492432	-6.732164	-0.337450

Table S21. XYZ Coordinates of S_II_para_OMe

43

scf done: -1113.302328

C	-0.000708	0.095013	-0.007576
C	0.003683	0.104387	1.392748
C	1.256364	0.153084	2.037443
C	2.445472	0.214430	1.312809
C	2.417055	0.223914	-0.088074
C	1.186625	0.164371	-0.749526
O	3.637679	0.290076	-0.709584
Pd	-1.624630	0.192980	2.533853
O	-3.537761	0.525888	3.826433
B	-4.599581	1.109285	3.127353
O	-5.855636	0.958757	3.630273
C	-2.301429	-1.466669	1.668454
C	-1.920779	-2.723116	2.174181
C	-2.498219	-3.901083	1.689060
C	-3.460144	-3.851637	0.678297
C	-3.837278	-2.614421	0.154347
C	-3.260695	-1.435468	0.640513
O	-0.946690	2.025338	3.732991
C	-0.352945	3.205730	3.144805
C	0.192554	4.141684	4.211379
O	-4.331658	1.831256	2.006282
H	-3.904867	-4.767319	0.298117
H	1.133761	0.159757	-1.832667
H	-0.940587	0.023795	-0.546113
H	1.310677	0.146739	3.123477
H	3.408985	0.251079	1.812913
H	-3.818861	-0.156623	4.449307
H	-6.522034	1.351601	3.054384
H	-3.568379	-0.487366	0.205831

H	-1.158437	-2.787456	2.945882
H	-2.186108	-4.859773	2.096937
H	-3.418662	1.670274	1.701081
H	-4.579795	-2.561876	-0.638736
H	-1.695996	2.281899	4.288456
H	0.441799	2.821852	2.503915
H	-1.090459	3.711481	2.508376
H	0.668477	5.008835	3.741644
H	-0.605051	4.515339	4.864552
H	0.936529	3.632636	4.830765
C	3.663355	0.274945	-2.124862
H	4.715832	0.325123	-2.410594
H	3.224307	-0.646039	-2.531372
H	3.134165	1.137257	-2.552923

Table S22. XYZ Coordinates of S_III_para_OMe

36

scf done: -860.790366

O	-0.323902	-0.339235	0.149233
Pd	-0.207935	-0.169197	2.418597
C	-0.274484	-0.119909	4.393006
C	-0.188638	1.071418	5.128565
C	-0.448118	1.109228	6.504163
C	-0.781473	-0.070689	7.178466
C	-0.845860	-1.277746	6.468750
C	-0.584925	-1.300285	5.101220
C	-0.338363	0.865617	-0.656914
C	-1.642227	1.596748	-0.404036
C	1.764739	0.050917	2.423654
C	2.331317	1.325605	2.263172
C	3.713232	1.471911	2.098558
C	4.547212	0.352256	2.102353
C	3.992403	-0.916398	2.277829
C	2.610895	-1.069392	2.444465
O	-1.051274	-0.153493	8.518458
C	-0.983020	1.034054	9.286995
H	5.620310	0.468486	1.978992
H	2.200260	-2.062082	2.603255
H	1.700897	2.210617	2.272844
H	4.633894	-1.794149	2.296047
H	4.135908	2.466073	1.974148
H	-0.627993	-2.254019	4.581062
H	0.084788	1.998097	4.631307
H	-1.094016	-2.185339	7.010975
H	-0.380023	2.054257	7.031505
H	0.487379	-0.830956	-0.043489
H	-0.251801	0.575990	-1.711045
H	0.522664	1.489589	-0.390332
H	-1.681123	2.508083	-1.008905
H	-2.498105	0.968611	-0.667819

H	-1.726435	1.879640	0.650224
H	-1.227678	0.747104	10.311379
H	0.022529	1.475152	9.267864
H	-1.706152	1.785375	8.942219

Table S23. XYZ Coordinates of S_TS2_para_OMe

36

scf done: -860.775282

H	0.094197	0.120191	-0.069043
C	0.073612	0.044643	1.022742
C	1.485461	0.112776	1.568629
O	1.423903	0.033519	3.014306
Pd	0.311147	1.530259	4.266106
C	0.440202	3.512222	4.714014
C	1.482267	3.925830	5.567069
C	2.043589	5.198442	5.447969
C	1.569464	6.095539	4.488009
C	0.524332	5.705352	3.647907
C	-0.040183	4.433467	3.762896
C	-0.988357	2.346772	5.585705
C	-0.796896	2.076299	6.950788
C	-1.869461	2.005538	7.845303
C	-3.172652	2.224509	7.384803
C	-3.384234	2.516690	6.029115
C	-2.310001	2.585252	5.150857
O	-4.293400	2.189631	8.168024
C	-4.135188	1.908178	9.547499
H	2.001556	7.088490	4.402905
H	1.854071	3.253002	6.333948
H	-0.865357	4.160355	3.112123
H	2.851943	5.490560	6.113992
H	0.139361	6.395348	2.900914
H	0.206090	1.922751	7.337640
H	-2.502716	2.833359	4.111479
H	-1.674173	1.787099	8.889093
H	-4.400395	2.693106	5.689723
H	2.319542	-0.031254	3.369539
H	2.087067	-0.728228	1.202388
H	1.973936	1.050552	1.277705
H	-0.404797	-0.899441	1.298544
H	-0.527190	0.869317	1.419009
H	-5.138005	1.927824	9.977910
H	-3.692393	0.916947	9.712735
H	-3.514105	2.662714	10.048308

Table S24. XYZ Coordinates of S_IV_para_OMe

36

scf done: -860.824269

C	0.141890	1.527439	-0.696034
C	-0.191045	1.000053	0.552023

C	0.634107	0.054621	1.162220
C	1.821746	-0.377081	0.547127
Pd	4.404215	-0.275361	1.958157
O	6.238891	0.980098	2.179296
C	6.229483	2.240410	2.889637
C	5.224956	3.158616	2.223214
C	2.696846	-1.406497	1.189952
C	2.759852	-1.572430	2.612778
C	3.264880	-2.774816	3.189235
C	3.736555	-3.792507	2.375201
C	3.722566	-3.632694	0.968390
C	3.231798	-2.479071	0.397815
C	1.311139	1.096164	-1.328418
C	2.139406	0.158769	-0.715733
O	4.234836	-4.987815	2.817184
C	4.244350	-5.225502	4.213402
H	5.179268	4.112112	2.759170
H	-0.502639	2.260015	-1.173189
H	0.342826	-0.367967	2.119011
H	3.058769	-0.145511	-1.206979
H	-1.104592	1.313937	1.050029
H	1.583927	1.497740	-2.300706
H	2.195158	-0.906812	3.260317
H	3.181437	-2.404045	-0.683960
H	3.250442	-2.874274	4.268062
H	4.096084	-4.446539	0.355262
H	6.923989	0.408353	2.547809
H	7.238982	2.669276	2.848289
H	5.963568	2.067085	3.939855
H	5.507575	3.354219	1.184924
H	4.231075	2.699665	2.231709
H	4.665549	-6.223088	4.349748
H	4.867869	-4.494757	4.745622
H	3.230599	-5.200869	4.635434

Table S25. XYZ Coordinates of S_I_meta_P(O)Ph$_2$

62

scf done: -1878.053984

C	0.666216	0.237387	0.120863
C	-0.491561	0.308563	0.905130
C	-0.433611	-0.162193	2.225675
C	0.748637	-0.715773	2.731863
C	1.893981	-0.796013	1.938801
C	1.858021	-0.297745	0.628841
Pd	-2.181422	1.034013	0.155033
O	-3.465731	0.283212	1.552170
B	-4.842261	0.683524	0.849759
O	-5.676498	1.356203	1.762539
O	-1.116725	2.005094	-1.433228

C	-1.121716	1.483433	-2.796449
C	0.074530	2.000257	-3.576057
O	-4.248847	1.627321	-0.216794
C	-5.537051	-0.617514	0.204499
C	-6.883966	-0.915952	0.476992
C	-7.513663	-2.030990	-0.078247
C	-6.806949	-2.885886	-0.927820
C	-5.468157	-2.616140	-1.215815
C	-4.848542	-1.495803	-0.655411
H	0.024355	1.625546	-4.603882
H	2.795448	-1.254962	2.334555
H	-7.295146	-3.755290	-1.360466
H	0.770209	-1.091718	3.751619
H	-1.309566	-0.100870	2.865176
H	0.676168	0.593755	-0.899786
P	3.272704	-0.259881	-0.527948
H	-3.408873	-0.678399	1.633408
H	-5.261724	2.173794	2.060144
H	-3.800779	-1.307118	-0.892454
H	-7.434895	-0.254913	1.139614
H	-8.556390	-2.236179	0.151622
H	-4.655600	1.433689	-1.069347
H	-4.908144	-3.276700	-1.873358
H	-1.281171	2.957726	-1.444972
H	-1.076521	0.400150	-2.676156
H	-2.075454	1.741137	-3.271671
H	0.077038	3.095527	-3.623490
H	1.011863	1.654298	-3.130707
O	2.830108	-0.065524	-1.957301
C	4.391366	1.077629	0.033339
C	4.190435	-1.824892	-0.290023
C	3.933070	-2.860244	-1.200531
C	4.576068	-4.090578	-1.063823
C	5.481287	-4.295380	-0.020155
C	5.750915	-3.264626	0.882584
C	5.111874	-2.031187	0.746924
H	3.241193	-2.683190	-2.018193
H	4.374349	-4.887609	-1.773760
H	5.982432	-5.253486	0.084833
H	6.465270	-3.417316	1.686565
H	5.347379	-1.226291	1.437289
C	5.206211	1.668938	-0.944862
C	6.095478	2.685658	-0.598516
C	6.176200	3.124293	0.725348
C	5.359230	2.550317	1.700953
C	4.466381	1.532993	1.357462
H	5.122037	1.336440	-1.975008
H	6.721778	3.139114	-1.361517
H	6.867940	3.917621	0.994285
H	5.409293	2.899596	2.728330

H	3.817099	1.109893	2.117723

Table S26. XYZ Coordinates of S_TS1_meta_P(O)Ph_2

62

scf done: -1878.036075

C	4.147325	1.573247	0.903804
C	3.715567	1.239950	-0.389046
C	3.986481	2.114002	-1.450944
C	4.685826	3.301520	-1.224439
C	5.116836	3.624377	0.063171
C	4.845710	2.759666	1.127570
P	2.775502	-0.284914	-0.795846
C	3.886045	-1.684588	-0.394514
C	5.280988	-1.539218	-0.390498
C	6.106536	-2.646241	-0.186891
C	5.547295	-3.909437	0.010929
C	4.159098	-4.065894	0.001886
C	3.333082	-2.961360	-0.201139
C	1.403902	-0.377740	0.407787
C	0.134300	0.011384	-0.054253
C	-0.971711	0.049166	0.807880
C	-0.788182	-0.343077	2.144939
C	0.471626	-0.745594	2.607901
C	1.568211	-0.764816	1.746594
Pd	-2.698768	0.789236	0.160488
O	-4.599673	1.366526	-0.715772
B	-4.783318	-0.018740	-1.213182
O	-4.295163	-0.213622	-2.526483
C	-3.533954	-1.123118	-0.145026
C	-2.769102	-2.015605	-0.934328
C	-2.569141	-3.343957	-0.554290
C	-3.109250	-3.808386	0.647473
C	-3.872614	-2.957454	1.455626
C	-4.077802	-1.638995	1.061787
O	-2.065292	2.907875	0.409644
C	-0.708519	3.375175	0.613730
C	-0.676067	4.887081	0.760461
O	-6.078135	-0.453608	-0.854177
O	2.295245	-0.250108	-2.222385
H	-2.943505	-4.838792	0.953906
H	2.534212	-1.104246	2.109347
H	0.033518	0.271807	-1.104615
H	-1.628754	-0.348051	2.831467
H	0.589829	-1.055618	3.643239
H	-5.294554	1.548979	-0.069061
H	-6.294409	-1.263450	-1.329317
H	-2.350311	-1.653984	-1.866465
H	-4.692894	-0.993136	1.684940
H	-4.300357	-3.327593	2.383502
H	-4.562868	0.541583	-3.075102

H	-1.984241	-4.009736	-1.182074
H	-2.431708	3.338400	-0.375925
H	-0.376001	2.874872	1.524210
H	-0.073497	3.032097	-0.208672
H	0.349728	5.220379	0.946784
H	-1.023820	5.385134	-0.153398
H	-1.304831	5.214130	1.593819
H	5.724178	-0.560614	-0.545368
H	7.185585	-2.521078	-0.183151
H	6.190478	-4.770290	0.170579
H	3.720305	-5.047962	0.153286
H	2.254585	-3.088938	-0.205961
H	3.637058	1.848051	-2.444003
H	4.893514	3.972924	-2.052951
H	5.661473	4.547807	0.239818
H	5.177547	3.010609	2.131196
H	3.934879	0.912705	1.739225

Table S27. XYZ Coordinates of S_II_meta_P(O)Ph$_2$

62
scf done: -1878.085979

C	-3.218643	-2.518594	0.237769
C	-3.486643	-1.394664	-0.567465
C	-4.589894	-1.468869	-1.443692
C	-5.396910	-2.613003	-1.503342
C	-5.118963	-3.710566	-0.689000
C	-4.026102	-3.657875	0.180510
Pd	-2.535807	0.342946	-0.381007
O	-4.585984	1.252072	0.289183
B	-4.749840	1.965470	1.454832
O	-3.893412	3.035737	1.603772
C	-0.797857	-0.461819	-0.918809
C	0.341039	0.051041	-0.274501
C	1.638251	-0.338318	-0.649801
C	1.813752	-1.265855	-1.685904
C	0.690212	-1.796750	-2.321499
C	-0.597972	-1.405072	-1.943079
O	-1.636019	2.380138	-0.005641
C	-0.963604	3.204606	-0.982759
C	0.043996	4.135382	-0.324796
O	-5.723253	1.578037	2.328004
H	-5.742199	-4.599603	-0.734234
H	2.809008	-1.557435	-2.007007
H	0.817589	-2.514836	-3.128201
H	-1.451879	-1.831400	-2.458953
H	0.231593	0.793580	0.508784
P	3.001086	0.464647	0.256639
H	-5.157122	0.470980	0.220445
H	-5.815978	2.098970	3.131430
H	-4.812907	-0.635745	-2.108090

H	-2.367802	-2.504372	0.912310
H	-3.798289	-4.510178	0.816793
H	-3.961556	3.515833	2.434585
H	-6.236887	-2.644226	-2.193784
H	-2.271558	2.905720	0.508124
H	-0.461397	2.502932	-1.650747
H	-1.711347	3.757719	-1.567047
H	0.515824	4.766548	-1.086528
H	-0.443350	4.799754	0.399230
H	0.827508	3.562451	0.180036
C	4.500380	0.329394	-0.789528
O	2.707865	1.897881	0.625465
C	3.327397	-0.573762	1.730564
C	4.888646	1.480223	-1.490428
C	6.012499	1.454784	-2.316910
C	6.758933	0.281865	-2.447390
C	6.385307	-0.864697	-1.742754
C	5.263183	-0.841128	-0.913057
H	4.310310	2.390285	-1.364336
H	6.308344	2.350968	-2.855065
H	7.634854	0.262716	-3.089887
H	6.971842	-1.774734	-1.832431
H	4.993378	-1.730555	-0.351057
C	3.851976	0.060929	2.866079
C	4.137092	-0.677642	4.014489
C	3.898067	-2.053800	4.039212
C	3.364444	-2.689810	2.916515
C	3.075187	-1.952817	1.766374
H	4.014667	1.134222	2.842451
H	4.541019	-0.179631	4.891533
H	4.118948	-2.628063	4.934724
H	3.164119	-3.757342	2.938511
H	2.637065	-2.447152	0.903931

Table S28. XYZ Coordinates of S_III_meta_P(O)Ph_2

55

scf done: -1625.573125

C	-0.047768	-0.488573	0.023814
C	1.083576	-0.508803	0.859034
C	0.888556	-0.356392	2.243450
C	-0.397282	-0.236125	2.776960
C	-1.515222	-0.242654	1.940529
C	-1.342746	-0.357512	0.554453
Pd	2.799758	-0.983517	0.001002
O	4.721707	-1.648129	-1.002722
C	4.826833	-1.606191	-2.447535
C	3.850163	-2.608437	-3.031186
C	3.681359	0.683425	0.619745
C	3.357866	1.947016	0.101284
C	4.119579	3.066681	0.450028

C	5.202557	2.945396	1.324330
C	5.521088	1.695234	1.854279
C	4.764549	0.567565	1.508314
H	-2.510885	-0.171633	2.368381
H	5.787735	3.819697	1.595032
H	5.013225	-0.393821	1.951693
H	2.511827	2.061372	-0.568231
H	6.354174	1.590041	2.545036
H	3.859017	4.038889	0.038944
H	1.744204	-0.343156	2.911899
H	0.051787	-0.592095	-1.053686
H	-0.529139	-0.143407	3.852191
P	-2.714939	-0.329971	-0.652303
H	5.348540	-1.013419	-0.622341
H	5.856743	-1.860461	-2.724833
H	4.606501	-0.591727	-2.800526
H	3.929175	-2.613077	-4.122623
H	4.061695	-3.616803	-2.663836
H	2.819194	-2.347329	-2.769491
O	-2.392116	-1.066534	-1.924629
C	-4.177058	-1.031783	0.201080
C	-3.104164	1.435702	-0.952834
C	-3.618092	1.765640	-2.215892
C	-3.951539	3.086487	-2.515596
C	-3.771134	4.088488	-1.559274
C	-3.247292	3.769445	-0.304883
C	-2.910102	2.448679	-0.002468
H	-3.734081	0.981953	-2.958348
H	-4.346945	3.334992	-3.496468
H	-4.029452	5.117357	-1.794050
H	-3.091866	4.549297	0.435326
H	-2.478966	2.211961	0.965915
C	-4.469575	-2.379470	-0.055554
C	-5.556918	-2.993339	0.566412
C	-6.362668	-2.266569	1.445693
C	-6.085309	-0.920972	1.695516
C	-4.999457	-0.303160	1.072616
H	-3.848021	-2.927913	-0.756704
H	-5.778589	-4.036828	0.361115
H	-7.210418	-2.744768	1.928390
H	-6.719171	-0.349563	2.367781
H	-4.806589	0.750414	1.252809

Table S29. XYZ Coordinates of S_TS2_meta_P(O)Ph$_2$

55

scf done: -1625.557328

C	-3.437322	1.783446	1.312261
C	-2.719314	1.280123	0.210311
C	-2.352061	2.177309	-0.810908
C	-2.725183	3.521021	-0.748294

C	-3.455462	4.003587	0.340228
C	-3.808142	3.128300	1.369756
Pd	-3.028178	-0.668990	-0.295860
O	-4.438467	-2.036644	-1.366989
C	-5.815828	-2.274041	-0.985100
C	-5.844152	-2.727712	0.460220
C	-1.322986	-0.122705	0.645808
C	-0.158300	-0.083163	-0.143650
C	1.098028	-0.378467	0.403106
C	1.207813	-0.719363	1.759442
C	0.062233	-0.739643	2.556895
C	-1.185967	-0.433660	2.012942
H	2.170905	-0.978745	2.188196
H	-3.736478	5.051616	0.390945
H	-1.764976	1.829044	-1.654962
H	-3.705869	1.124700	2.132710
H	-2.436059	4.193628	-1.551848
H	-4.370097	3.492497	2.226271
H	-0.214455	0.160639	-1.199475
H	-2.059869	-0.441790	2.657185
P	2.522602	-0.275717	-0.739495
H	0.140671	-1.000561	3.609242
H	-4.391450	-1.856446	-2.314516
H	-6.227900	-3.046584	-1.645567
H	-6.392954	-1.350989	-1.118429
H	-6.878783	-2.896347	0.775168
H	-5.284197	-3.658645	0.586285
H	-5.401728	-1.964132	1.107761
O	2.138835	-0.504429	-2.176425
C	3.278705	1.372447	-0.475779
C	3.752485	-1.498784	-0.147671
C	3.931251	1.951812	-1.574414
C	4.543834	3.198701	-1.450077
C	4.506365	3.878726	-0.230572
C	3.846744	3.314309	0.862986
C	3.230609	2.067322	0.741451
H	3.935083	1.424019	-2.523232
H	5.045136	3.642376	-2.305656
H	4.981634	4.851042	-0.135146
H	3.802304	3.848531	1.807857
H	2.696719	1.646797	1.588610
C	4.653932	-1.258544	0.899427
C	5.547985	-2.253364	1.299280
C	5.554225	-3.490217	0.651149
C	4.669824	-3.729995	-0.402901
C	3.773571	-2.738814	-0.803180
H	4.673558	-0.290674	1.391927
H	6.244975	-2.059218	2.109684
H	6.253325	-4.261935	0.961115
H	4.681917	-4.687110	-0.916647

H	3.095461	-2.905626	-1.634510

Table S30. XYZ Coordinates of S_IV_meta_P(O)Ph$_2$

55

scf done: -1625.605360

C	-2.522300	1.278176	-1.103797
C	-2.190862	1.243264	0.294060
C	-2.921151	2.121442	1.161842
C	-3.865940	3.003821	0.669071
C	-4.158126	3.048929	-0.710156
C	-3.504661	2.191713	-1.578392
C	-0.938653	0.589976	0.787563
C	0.201735	0.502784	-0.026273
C	1.382223	-0.094444	0.434720
C	1.436314	-0.621960	1.731581
C	0.314200	-0.525124	2.557559
C	-0.854829	0.073245	2.094619
Pd	-3.524696	-0.426875	-0.173543
O	-4.748982	-2.296829	-0.134901
C	-6.150554	-2.294007	-0.496903
C	-6.887705	-1.364918	0.446239
H	-4.890352	3.756804	-1.087567
H	2.332946	-1.117685	2.090502
H	-1.726675	0.112048	2.740323
H	0.183420	0.897963	-1.036454
H	0.346420	-0.934736	3.563420
P	2.789946	-0.116652	-0.734626
H	-2.669116	2.140770	2.217757
H	-1.873439	0.796527	-1.830329
H	-4.374334	3.679032	1.351749
H	-3.707130	2.229980	-2.645125
H	-4.278718	-2.938485	-0.682368
H	-6.531702	-3.319760	-0.413237
H	-6.258428	-1.960740	-1.536369
H	-7.948268	-1.327446	0.177537
H	-6.801175	-1.712511	1.479715
H	-6.471293	-0.354516	0.381854
C	3.781204	-1.601200	-0.321638
O	2.351669	-0.105341	-2.173611
C	3.833016	1.328605	-0.311861
C	4.544188	1.923750	-1.364525
C	5.370985	3.020590	-1.121460
C	5.491273	3.533825	0.171929
C	4.775666	2.954734	1.221907
C	3.945109	1.858650	0.981759
H	4.426016	1.529426	-2.369167
H	5.916507	3.478363	-1.941761
H	6.133610	4.389602	0.359929
H	4.855486	3.362171	2.225788
H	3.370680	1.430927	1.798374

C	3.570876	-2.738889	-1.114971
C	4.276188	-3.914633	-0.857861
C	5.198995	-3.962970	0.189370
C	5.423446	-2.830069	0.974286
C	4.721283	-1.651087	0.717895
H	2.866520	-2.682440	-1.939259
H	4.110095	-4.791003	-1.477926
H	5.749425	-4.878389	0.387717
H	6.151797	-2.860621	1.779732
H	4.921503	-0.767103	1.316273

Table S31. XYZ Coordinates of boronic acid

7

scf done: -252.484727

B	0.474753	0.187642	0.086977
O	-0.010962	-0.221502	1.295558
O	1.437405	-0.604583	-0.486953
O	-0.030783	1.351575	-0.460192
H	1.759576	-0.292095	-1.337263
H	0.341213	1.602610	-1.311164
H	-0.677970	0.393183	1.621370

Table S32. XYZ Coordinates of S_I_meta_Me

42

scf done: -1038.066707

C	0.002395	-0.174960	0.010442
C	-0.004124	-0.020716	1.403620
C	1.214356	0.105133	2.084630
C	2.441109	0.076192	1.401324
C	2.426211	-0.083354	0.009686
C	1.219030	-0.205078	-0.678674
Pd	-1.715828	0.024633	2.398099
O	-3.436764	0.561029	3.644497
B	-2.712388	1.747337	4.320836
O	-3.474375	2.932739	4.313217
H	3.365876	-0.110194	-0.536921
C	-2.167605	1.373928	5.791361
C	-2.363967	2.254382	6.870263
C	-1.897584	1.958628	8.152341
C	-1.216611	0.762815	8.393398
C	-1.007889	-0.132050	7.342663
C	-1.479662	0.175339	6.063624
O	-1.520462	1.853619	3.283221
O	-2.285930	-1.850962	1.522468
C	-1.543901	-3.069273	1.818562
C	-2.087663	-4.236384	1.014523
H	-2.065326	2.659911	8.966200
H	-0.852481	0.530460	9.390835
C	3.743868	0.244495	2.149506
H	1.225264	0.219934	3.167220

H	-0.931012	-0.269532	-0.537680
H	1.222409	-0.323645	-1.759609
H	-0.674928	2.024172	3.716931
H	-3.789681	3.120172	3.421647
H	-1.303263	-0.536352	5.256066
H	-2.896529	3.183125	6.687485
H	-3.747701	-0.036788	4.334350
H	-0.479404	-1.065977	7.519482
H	-3.223770	-1.995066	1.714036
H	-0.516226	-2.830524	1.545100
H	-1.584223	-3.260240	2.897059
H	-1.500321	-5.135375	1.227070
H	-3.130077	-4.455288	1.274571
H	-2.030452	-4.031568	-0.057956
H	4.554685	-0.315759	1.673406
H	4.050744	1.297726	2.177427
H	3.659102	-0.096424	3.185827

Table S33. XYZ Coordinates of S_TS1_meta_Me

42

scf done: -1038.051180

C	0.033504	0.004477	0.045439
C	0.013553	-0.021245	1.466967
C	1.272025	-0.011620	2.117792
C	2.468182	0.062982	1.406182
C	2.443671	0.091058	0.009605
C	1.222830	0.058240	-0.674581
Pd	-1.380171	-1.490906	2.123250
O	-3.045515	-2.806497	2.790341
C	-2.893302	-4.137025	3.345191
C	-4.248559	-4.760504	3.634432
B	-1.297438	1.102964	2.304959
O	-1.513367	2.198705	1.429555
C	-0.100867	-3.001149	1.964843
C	0.711558	-3.367940	3.047988
C	1.508338	-4.523631	3.014596
C	1.484578	-5.319959	1.862160
C	0.694065	-4.960108	0.770322
C	-0.091488	-3.803240	0.813131
C	2.398849	-4.879483	4.183773
O	-2.565737	0.314786	2.353673
O	-0.745099	1.321820	3.596679
H	3.375278	0.142245	-0.548383
H	2.092872	-6.220563	1.818216
H	0.732585	-2.747104	3.941822
H	-0.696907	-3.534857	-0.048812
H	0.688846	-5.582209	-0.121926
H	-3.093043	0.550950	1.577810
H	-0.764428	2.803040	1.470328
H	1.290779	-0.022122	3.202382

H	-0.908594	0.018765	-0.496861
H	1.207246	0.081729	-1.760973
H	-1.422979	1.724005	4.153942
H	3.417940	0.091603	1.933616
H	-3.525376	-2.250975	3.421422
H	-2.345238	-4.693278	2.584515
H	-2.266334	-4.088682	4.242709
H	-4.116516	-5.777492	4.018084
H	-4.800168	-4.191275	4.392136
H	-4.857473	-4.809720	2.727161
H	2.522553	-5.963042	4.279385
H	3.401347	-4.448801	4.064686
H	1.994359	-4.499946	5.127371

Table S34. XYZ Coordinates of S_II_meta_Me

42
scf done: -1038.096384

C	0.360739	1.830529	-0.437811
C	-0.117492	0.890869	0.483664
C	0.807429	0.186095	1.270364
C	2.190085	0.404960	1.163556
Pd	3.540222	-0.436384	2.356464
C	2.875702	-2.258927	1.906087
B	6.461245	-1.228395	3.744382
O	6.784199	0.105496	3.895958
C	2.916601	-2.808195	0.609827
C	2.598404	-4.149600	0.381754
C	2.220097	-4.980071	1.440456
C	2.158958	-4.455011	2.730777
C	2.481540	-3.110479	2.960188
C	1.731861	2.053895	-0.567915
C	2.643266	1.348998	0.223895
O	4.497714	1.538605	2.946150
C	3.736415	2.498423	3.705230
C	4.505511	3.798756	3.887754
O	5.113382	-1.493260	3.754341
O	7.339060	-2.263028	3.607294
C	-1.598509	0.609582	0.606337
H	1.969104	-6.021685	1.258767
H	-0.341947	2.385217	-1.055914
H	0.435051	-0.550207	1.977364
H	3.706812	1.543261	0.114185
H	2.095078	2.784141	-1.288087
H	4.881027	-2.420869	3.589817
H	8.274858	-2.040150	3.607884
H	2.397161	-2.718780	3.972693
H	3.192995	-2.178060	-0.230080
H	2.642016	-4.547308	-0.629761
H	7.720955	0.318693	3.848146
H	1.852379	-5.085190	3.562852

H	5.309338	1.298208	3.426733
H	2.825441	2.656870	3.125490
H	3.452200	2.066361	4.673813
H	3.894251	4.525728	4.433126
H	5.426154	3.641920	4.462310
H	4.772054	4.229702	2.918288
H	-2.198184	1.472706	0.299909
H	-1.896651	-0.235566	-0.027513
H	-1.874279	0.353197	1.634279

Table S35. XYZ Coordinates of S_III_meta_Me

35

scf done: -785.586986

H	0.488395	0.065551	-0.160890
C	0.363450	0.048581	0.919099
C	1.493800	0.028032	1.741914
C	1.354336	0.009757	3.137963
C	0.061503	-0.015530	3.690614
C	-1.066782	0.002851	2.859889
C	-0.918838	0.038868	1.473539
Pd	2.893302	0.238344	4.373669
O	2.086457	2.268832	5.026022
C	2.689461	3.481061	4.513784
C	4.102681	3.581530	5.054452
C	3.633955	-1.533448	3.906747
C	4.617767	-1.652120	2.908931
C	5.315074	-2.850285	2.694249
C	4.992737	-3.960851	3.485672
C	4.004034	-3.870728	4.465506
C	3.319884	-2.669780	4.673562
C	6.401724	-2.936001	1.646523
H	5.512287	-4.903003	3.325441
H	-1.793688	0.049865	0.829589
H	-0.075538	-0.074575	4.767870
H	2.481531	0.017533	1.292419
H	-2.059225	-0.021021	3.303537
H	2.540008	-2.622781	5.428368
H	4.854200	-0.796284	2.279077
H	3.755094	-4.744197	5.063652
H	1.182991	2.197847	4.680106
H	2.085809	4.334717	4.844853
H	2.690197	3.454719	3.417457
H	4.573404	4.503667	4.699739
H	4.100377	3.591814	6.148301
H	4.709320	2.736409	4.712109
H	6.423466	-3.921948	1.170959
H	6.261576	-2.185740	0.862185
H	7.393608	-2.767283	2.085311

Table S36. XYZ Coordinates of S_TS2_meta_Me

35
scf done: -785.571904

H	-0.005661	0.016172	0.019213
C	-0.013385	0.003442	1.112806
C	1.399027	0.002822	1.661329
O	2.074098	-1.190870	1.192989
Pd	1.334741	-3.269452	1.599820
C	1.048516	-4.565643	3.145144
C	-0.092297	-4.382637	3.950488
C	-0.079066	-4.732464	5.302017
C	1.065227	-5.288419	5.878279
C	2.197651	-5.493088	5.086813
C	2.188866	-5.144843	3.734879
C	0.673979	-5.149146	1.232454
C	1.595196	-6.066667	0.693984
C	1.161194	-7.078130	-0.163960
C	-0.190371	-7.204712	-0.485365
C	-1.132674	-6.313838	0.047614
C	-0.687698	-5.304748	0.910099
H	1.883002	-7.778779	-0.576636
H	-0.518972	-8.005165	-1.143987
H	1.070816	-5.568618	6.927758
H	3.073755	-5.332156	3.134339
H	-1.000001	-3.971456	3.519297
H	3.092887	-5.932271	5.520217
H	-0.970506	-4.574714	5.904227
H	2.647908	-5.999216	0.950223
H	-1.422602	-4.632164	1.344503
C	-2.595686	-6.429349	-0.315763
H	3.006404	-1.151153	1.441229
H	1.957336	0.876995	1.304500
H	1.394319	0.009795	2.757969
H	-0.548907	0.889107	1.469090
H	-0.551583	-0.888951	1.447572
H	-2.909038	-7.476099	-0.386595
H	-3.232355	-5.935599	0.424545
H	-2.802504	-5.964020	-1.287900

Table S37. XYZ Coordinates of S_IV_meta_Me

35
scf done: -785.621028

C	0.281162	-0.945034	0.380591
C	0.697061	-0.934090	1.698031
C	1.813607	-0.152803	2.109221
C	2.511868	0.645472	1.142961
Pd	3.652394	-1.188913	1.547706
C	3.429618	1.754489	1.552596
C	4.510579	2.139808	0.737098
C	3.216352	2.471172	2.742336

C	4.060442	3.518523	3.113030
C	5.132531	3.883269	2.297880
C	5.349752	3.189234	1.104626
C	0.973745	-0.195735	-0.602370
C	2.065190	0.567726	-0.217693
O	5.189367	-2.812663	1.504256
C	6.014345	-3.111537	2.654963
C	6.802830	-1.868506	3.014657
C	0.516748	-0.233591	-2.041517
H	7.459249	-1.573687	2.190992
H	0.150786	-1.499431	2.448333
H	-0.585392	-1.534072	0.090006
H	5.787745	4.700693	2.584784
H	2.369987	2.219580	3.374114
H	4.706655	1.592386	-0.179857
H	3.870189	4.058148	4.036933
H	6.181052	3.461834	0.459959
H	1.963139	0.018425	3.171980
H	2.561722	1.188495	-0.958309
H	4.732090	-3.614250	1.220551
H	6.686374	-3.939188	2.394130
H	5.376866	-3.425321	3.490787
H	7.417178	-2.060530	3.900181
H	6.121665	-1.038966	3.230113
H	-0.554225	-0.016058	-2.127405
H	1.057555	0.493752	-2.653552
H	0.677863	-1.224768	-2.483890

Table S38. XYZ Coordinates of S_I_meta_OMe

43

scf done: -1113.270516

C	-0.052160	-0.143567	-0.044606
C	-0.027224	-0.032110	1.356610
C	1.197613	0.054993	2.019763
C	2.403720	0.029092	1.296961
C	2.382230	-0.080275	-0.097984
C	1.149352	-0.164585	-0.753088
Pd	-1.718209	0.014390	2.385162
O	-3.417260	0.552295	3.655883
B	-2.688571	1.752470	4.306133
O	-3.457389	2.933236	4.290890
H	3.299510	-0.096705	-0.674275
C	-2.125512	1.400594	5.774574
C	-2.329479	2.285605	6.848282
C	-1.849511	2.007174	8.129188
C	-1.146510	0.824959	8.374026
C	-0.929438	-0.073813	7.328362
C	-1.415261	0.215965	6.050517
O	-1.511367	1.850004	3.251468
O	-2.295459	-1.869488	1.534757

C	-1.532876	-3.079215	1.813236
C	-2.084764	-4.253176	1.024883
H	-2.023433	2.711550	8.939084
H	-0.771273	0.606326	9.370453
O	3.540405	0.115312	2.051264
H	1.262295	0.139600	3.101139
H	-0.996385	-0.204718	-0.577469
H	1.132518	-0.242833	-1.837534
H	-0.658401	2.022938	3.669823
H	-3.783767	3.106886	3.400537
H	-1.231652	-0.498449	5.246993
H	-2.878454	3.204096	6.662307
H	-3.705980	-0.041552	4.358778
H	-0.382775	-0.996644	7.507832
H	-3.225979	-2.022166	1.753315
H	-0.515115	-2.830080	1.513352
H	-1.543259	-3.268812	2.892667
H	-1.482611	-5.145588	1.223295
H	-3.117762	-4.482760	1.311919
H	-2.057391	-4.049138	-0.048924
C	4.790457	0.117176	1.382465
H	5.550018	0.202018	2.161402
H	4.952998	-0.812548	0.821236
H	4.882296	0.968653	0.695641

Table S39. XYZ Coordinates of S_TS1_meta_OMe

43

scf done: -1113.255133

C	0.026422	0.002422	0.037248
C	0.031867	-0.030091	1.458888
C	1.301788	-0.041141	2.087337
C	2.485862	0.021254	1.354532
C	2.436352	0.058210	-0.041089
C	1.203079	0.045382	-0.703651
Pd	-1.360447	-1.491381	2.136325
O	-3.019751	-2.795759	2.835239
C	-2.870034	-4.148467	3.335171
C	-4.222777	-4.750949	3.675872
B	-1.251577	1.101902	2.321310
O	-1.478022	2.200680	1.451721
C	-0.094101	-3.009252	1.955152
C	0.694555	-3.398632	3.040463
C	1.482589	-4.560418	2.977110
C	1.493867	-5.339618	1.814883
C	0.713265	-4.937891	0.725569
C	-0.071674	-3.785723	0.781187
O	2.203408	-4.844227	4.107259
O	-2.524474	0.321988	2.393807
O	-0.673914	1.316191	3.602540
H	3.358386	0.100523	-0.615544

H	2.097821	-6.236390	1.743215
H	0.727220	-2.818179	3.958306
H	-0.659493	-3.493732	-0.084285
H	0.726444	-5.536981	-0.182166
H	-3.067186	0.564129	1.630570
H	-0.726587	2.802480	1.483182
H	1.340088	-0.059620	3.171230
H	-0.925219	0.032012	-0.487488
H	1.168490	0.075806	-1.789452
H	-1.340494	1.715686	4.175101
H	3.445112	0.032712	1.865034
H	-3.457896	-2.254463	3.507678
H	-2.377495	-4.689128	2.526616
H	-2.194182	-4.146491	4.197482
H	-4.095225	-5.783577	4.016902
H	-4.718578	-4.196809	4.481824
H	-4.880856	-4.754145	2.802189
C	3.042371	-5.985167	4.095305
H	3.526206	-6.016802	5.073305
H	2.470594	-6.911415	3.947938
H	3.812346	-5.916711	3.315349

Table S40. XYZ Coordinates of S_II_meta_OMe

43

scf done: -1113.303064

C	-0.106483	0.101548	1.040437
C	0.219940	0.371221	2.500501
O	1.446510	-0.285086	2.895974
Pd	2.195009	-0.398825	5.055114
O	4.187991	0.183743	3.993859
B	4.567604	1.517471	4.178349
O	5.876279	1.840769	3.987950
C	0.410471	-0.766099	5.853383
C	-0.313213	-1.867800	5.350100
C	-1.621845	-2.098065	5.774044
C	-2.244302	-1.253965	6.699705
C	-1.526350	-0.165408	7.206247
C	-0.204734	0.067024	6.790045
O	-2.020712	0.731095	8.117095
C	-3.335230	0.532315	8.603290
C	3.021182	-0.576135	6.857578
C	3.586664	-1.809246	7.230603
C	4.317874	-1.938127	8.416377
C	4.491530	-0.839817	9.260362
C	3.922797	0.386383	8.912337
C	3.189914	0.514572	7.727470
O	3.615847	2.435686	4.494190
H	5.056034	-0.940585	10.183375
H	-3.261420	-1.455298	7.014480
H	0.319133	0.906981	7.233498

H	0.142052	-2.542697	4.630554
H	-2.172449	-2.951012	5.383527
H	4.933165	-0.430537	4.009419
H	6.049093	2.768027	4.188994
H	2.743804	1.477745	7.491446
H	3.447658	-2.683334	6.599295
H	4.743161	-2.902878	8.683424
H	2.791628	1.998459	4.778237
H	4.042167	1.247447	9.565900
H	2.181374	0.052503	2.365102
H	-0.538988	-0.044748	3.164413
H	0.296411	1.447393	2.701670
H	-1.055998	0.577668	0.774292
H	0.664607	0.506809	0.374310
H	-0.194344	-0.972681	0.854187
H	-3.521084	1.338303	9.315740
H	-4.081401	0.586015	7.798769
H	-3.438440	-0.432508	9.117669

Table S41. XYZ Coordinates of S_III_meta_OMe

36

scf done: -860.790826

H	0.507430	-0.019208	-0.161043
C	0.378397	-0.007786	0.918520
C	1.505623	-0.027211	1.745618
C	1.360182	-0.009039	3.141009
C	0.065456	0.000118	3.689551
C	-1.059504	0.016960	2.854227
C	-0.905862	0.017234	1.468048
Pd	2.897259	0.223473	4.377829
O	2.108009	2.267741	5.003060
C	2.725511	3.463789	4.469684
C	4.141086	3.554745	5.005705
C	3.628105	-1.555512	3.922824
C	4.637732	-1.662195	2.958558
C	5.321629	-2.872696	2.759403
C	4.984520	-4.000579	3.515371
C	3.961377	-3.899056	4.464306
C	3.279358	-2.700721	4.668375
O	6.297744	-2.846515	1.798316
H	5.493866	-4.946359	3.372655
H	-1.778111	0.027263	0.820540
H	-0.076245	-0.031360	4.767446
H	2.494732	-0.066229	1.301019
H	-2.053787	0.020055	3.294394
H	2.479491	-2.656499	5.401098
H	4.924897	-0.818069	2.337816
H	3.692769	-4.778105	5.045472
H	1.204184	2.201309	4.657072
H	2.133371	4.330212	4.788109

H	2.723314	3.419181	3.373995
H	4.622877	4.464726	4.634869
H	4.141513	3.582932	6.099252
H	4.735972	2.696481	4.675644
C	7.016067	-4.040290	1.544074
H	7.732221	-3.801824	0.755477
H	7.562073	-4.385057	2.432478
H	6.356558	-4.847420	1.198053

Table S42. XYZ Coordinates of S_TS2_meta_OMe

36

scf done: -860.775519

H	-0.009280	-0.074313	-0.068093
C	-0.039294	-0.050968	1.024920
C	1.361240	-0.008303	1.601499
O	2.066020	-1.205274	1.188232
Pd	1.343113	-3.284064	1.614242
C	1.046114	-4.581741	3.155779
C	-0.107659	-4.410396	3.944797
C	-0.108282	-4.757956	5.296752
C	1.034472	-5.300946	5.888729
C	2.179206	-5.495261	5.112795
C	2.184610	-5.148802	3.760170
C	0.700804	-5.168595	1.241483
C	1.637363	-6.084707	0.715967
C	1.217647	-7.090348	-0.149346
C	-0.128745	-7.232940	-0.503250
C	-1.063718	-6.337410	0.028197
C	-0.650133	-5.320170	0.900136
H	1.946623	-7.786515	-0.556854
H	-0.429102	-8.032636	-1.169459
H	1.029083	-5.579718	6.938617
H	3.079044	-5.327268	3.171278
H	-1.014455	-4.012270	3.499802
H	3.073084	-5.924666	5.558631
H	-1.009430	-4.609820	5.886756
H	2.685494	-6.013552	0.986778
H	-1.414405	-4.664480	1.303583
O	-2.406849	-6.371545	-0.236829
H	2.991467	-1.142961	1.456411
H	1.912313	0.862724	1.226186
H	1.333685	0.036343	2.696958
H	-0.596980	0.836455	1.340542
H	-0.569399	-0.941103	1.378115
C	-2.889291	-7.375514	-1.111788
H	-3.966991	-7.219699	-1.187426
H	-2.443285	-7.293164	-2.111971
H	-2.699059	-8.383652	-0.720023

Table S43. XYZ Coordinates of S_IV_meta_OMe

36			
scf done: -860.825279			
C	0.077259	-0.368803	0.099559
C	0.028848	0.027863	1.455079
C	1.116057	0.597074	2.084273
C	2.343610	0.821552	1.373916
Pd	3.421639	-1.098291	1.200934
C	3.355746	1.769170	1.937741
C	4.011957	2.706606	1.122139
C	3.646636	1.779688	3.315251
C	4.566200	2.679137	3.849857
C	5.221595	3.595266	3.022776
C	4.937124	3.603740	1.656802
C	1.253836	-0.189328	-0.612503
C	2.406575	0.388163	0.007979
O	4.531626	-2.905982	1.900593
C	5.815109	-2.817591	2.562873
C	6.778258	-2.088444	1.648045
O	1.418652	-0.478271	-1.936128
C	0.337719	-1.083458	-2.625468
H	6.911936	-2.635562	0.710460
H	-0.897553	-0.108624	2.006443
H	-0.803386	-0.794923	-0.365168
H	5.939166	4.296185	3.439396
H	3.161900	1.058571	3.966436
H	3.783420	2.746489	0.061693
H	4.775638	2.661792	4.916169
H	5.427564	4.319591	1.002610
H	1.028818	0.945417	3.107983
H	3.187967	0.739244	-0.660627
H	3.924414	-3.423805	2.443818
H	6.167977	-3.835721	2.771591
H	5.700767	-2.282723	3.513929
H	7.752389	-1.988642	2.137505
H	6.395091	-1.089279	1.417146
H	0.683009	-1.246061	-3.647650
H	0.064934	-2.047527	-2.176822
H	-0.546979	-0.433446	-2.642128

Table S44. XYZ Coordinates of A_mono_I

45			
scf done: -5685.163227			
C	-0.010052	0.008935	0.009721
C	-0.002956	0.009459	1.452072
C	1.298927	0.002991	2.099619
C	2.494145	0.117692	1.311263
C	2.416767	0.050010	-0.054832
C	1.150694	-0.014368	-0.717429

Ni	0.402888	-1.596149	2.539243
P	-1.404010	-2.723246	2.403451
O	-1.543449	-3.824739	1.199147
C	-1.559645	-3.386596	-0.172922
Br	1.344124	0.995174	3.859870
H	1.116866	-0.013043	-1.802922
P	1.757941	-2.926050	3.498114
O	3.399751	-2.788542	3.365606
C	4.066927	-1.705773	4.038618
O	1.509663	-3.112986	5.097336
C	2.165390	-4.148602	5.863138
O	1.671690	-4.490809	3.003852
C	1.994837	-4.790558	1.635471
O	-2.836080	-1.937594	2.155631
C	-3.238931	-0.981105	3.151228
O	-1.727429	-3.674799	3.686481
C	-2.867638	-4.561857	3.716412
H	-2.982813	-4.882623	4.752221
H	3.329142	0.073844	-0.644149
H	3.454009	0.221485	1.805627
H	-0.844005	0.505558	1.930719
H	-0.968630	0.074556	-0.499293
H	-4.022841	-0.365887	2.707796
H	-3.631484	-1.489465	4.036977
H	-2.404990	-0.337107	3.450770
H	-1.556830	-4.287784	-0.787262
H	-2.462477	-2.805826	-0.378918
H	-0.676461	-2.784715	-0.406189
H	-3.772847	-4.046337	3.386839
H	-2.686498	-5.428216	3.076568
H	4.985353	-1.497356	3.487089
H	3.451479	-0.803477	4.072058
H	4.320584	-2.001203	5.061482
H	1.709670	-5.829140	1.462916
H	1.432947	-4.148190	0.948814
H	3.066439	-4.668866	1.456644
H	2.068871	-3.867456	6.912381
H	1.676158	-5.109310	5.688908
H	3.222615	-4.223715	5.597653

Table S45. XYZ Coordinates of A_mono_TS1

45

scf done: -5685.159017

C	0.001789	0.011503	0.009290
C	0.001493	0.007141	1.431958
C	1.277914	0.003355	2.071743
C	2.468806	0.288382	1.362659
C	2.410239	0.311767	-0.015650
C	1.180107	0.135713	-0.700865
Ni	0.569514	-1.488478	2.884327

P	-1.251112	-2.488891	3.426693
O	-1.656351	-3.866749	2.637112
C	-1.776294	-3.828826	1.204554
Br	1.098423	0.681172	4.102047
P	2.114961	-2.944065	2.943914
O	3.725917	-2.554896	2.982676
C	4.175894	-1.731929	4.073181
O	1.974748	-4.037753	4.152499
C	2.840391	-5.188963	4.248508
O	2.201384	-3.992632	1.676922
C	2.466421	-3.479080	0.361584
O	-2.695639	-1.704615	3.225407
C	-2.894291	-0.487571	3.968198
O	-1.322763	-3.013539	4.966547
C	-2.441322	-3.779983	5.465910
H	1.162907	0.166363	-1.785754
H	-2.370906	-3.758293	6.553885
H	3.325807	0.450478	-0.583716
H	3.411841	0.392125	1.886894
H	-0.906391	0.314164	1.934402
H	-0.949975	0.003480	-0.515568
H	-3.688361	0.069979	3.469618
H	-3.196241	-0.711942	4.995772
H	-1.984538	0.121582	3.991478
H	-1.866343	-4.862326	0.867972
H	-2.666929	-3.268233	0.907358
H	-0.889645	-3.377003	0.748009
H	-3.389728	-3.339910	5.148637
H	-2.378786	-4.810752	5.110249
H	5.182504	-1.391759	3.824660
H	3.523826	-0.864500	4.213374
H	4.208974	-2.308905	5.003083
H	2.347214	-4.313100	-0.331801
H	1.762585	-2.683800	0.093922
H	3.487671	-3.093369	0.296283
H	2.700185	-5.600780	5.248858
H	2.564413	-5.934336	3.499056
H	3.886435	-4.903956	4.111273

Table S46. XYZ Coordinates of A_mono_IIa

45

scf done: -5685.213072

C	0.008888	-0.008594	0.012521
C	0.022617	-0.001714	1.413035
C	1.235012	0.009343	2.103516
C	2.431672	0.015298	1.379653
C	2.409028	0.004631	-0.017238
C	1.199075	-0.017371	-0.732688
H	1.249763	0.016303	3.189841
Ni	1.163108	-0.248365	-2.622031

P	0.846552	1.833185	-2.711818
O	1.112413	2.580477	-4.135822
C	2.456628	2.688252	-4.643286
Br	1.723133	-2.499454	-2.283032
P	0.987206	-0.702093	-4.817905
O	0.445664	0.363812	-5.936666
C	-0.916675	0.825577	-5.852237
O	0.005280	-1.975123	-5.054254
C	-0.251871	-2.571467	-6.344384
O	2.450278	-0.995750	-5.447201
C	2.786904	-1.020539	-6.855166
O	-0.710461	2.199770	-2.469969
C	-1.236617	3.533219	-2.696141
O	1.715142	2.847051	-1.782848
C	1.407505	3.181076	-0.412074
H	3.384110	0.023803	1.904397
H	3.354925	0.000931	-0.554312
H	-0.952349	-0.005967	-0.497015
H	-0.916493	-0.002156	1.961197
H	1.553500	4.258571	-0.311524
H	0.376718	2.924169	-0.164488
H	2.083008	2.644013	0.253034
H	3.871947	-0.929742	-6.906479
H	2.480017	-1.969660	-7.299985
H	2.316993	-0.188904	-7.380590
H	-1.009366	1.649743	-6.559401
H	-1.612791	0.028309	-6.125445
H	-1.149699	1.187085	-4.847782
H	-1.235799	-3.037836	-6.284474
H	-0.250942	-1.822428	-7.139521
H	0.501939	-3.335592	-6.550098
H	-2.224781	3.548799	-2.237466
H	-0.600041	4.288906	-2.231685
H	-1.313829	3.723618	-3.767512
H	2.379429	3.148050	-5.628301
H	3.062343	3.315606	-3.985883
H	2.918455	1.701103	-4.744270

Table S47. XYZ Coordinates of A_mono_IIb

45

scf done: -5685.222075

C	-0.029234	0.009028	0.002195
C	-0.019653	0.011329	1.400829
C	1.181147	0.008921	2.131349
C	2.378053	0.011461	1.393945
C	2.378741	0.002869	-0.003799
C	1.172460	0.000385	-0.708406
Ni	1.160134	0.019403	4.016587
P	0.733997	-2.115317	3.912061
O	1.200647	-2.958200	5.220569

C	0.853620	-4.352817	5.386433
H	1.169747	-0.004832	-1.794851
Br	0.975715	0.096747	6.363054
P	1.400498	2.192175	4.021173
O	2.573367	2.837278	4.950905
C	3.905655	2.306861	4.791611
O	1.638722	2.876261	2.566961
C	1.805954	4.305584	2.429608
O	0.193030	3.067079	4.678064
C	-1.150327	2.798341	4.226162
O	1.258110	-3.033988	2.672104
C	2.678771	-3.254358	2.561560
O	-0.836679	-2.481133	3.679096
C	-1.836721	-1.894087	4.538602
H	-1.453781	1.783340	4.498878
H	3.323269	0.004208	-0.542599
H	3.335243	0.039492	1.911378
H	-0.973030	0.013590	1.925458
H	-0.977035	0.011819	-0.530778
H	-1.793672	3.517640	4.732632
H	-1.234524	2.930534	3.143700
H	1.952533	4.494773	1.366272
H	0.915836	4.832183	2.781208
H	2.678273	4.647093	2.991340
H	4.556480	2.918660	5.416220
H	3.949417	1.267006	5.128773
H	4.232689	2.373887	3.749781
H	1.514344	-4.743428	6.160455
H	-0.186362	-4.444786	5.708230
H	1.000236	-4.910149	4.458688
H	-2.796226	-2.279009	4.193518
H	-1.675556	-2.184481	5.579985
H	-1.829244	-0.803723	4.461637
H	2.824700	-3.871360	1.674959
H	3.210349	-2.308807	2.432544
H	3.062270	-3.780409	3.439795

Table S48. XYZ Coordinates of A_mono_III

45

scf done: -5685.157792

C	0.032285	-0.014238	-0.013789
C	0.016194	0.003131	1.384970
C	1.216439	-0.001372	2.108817
C	2.432021	-0.030561	1.410153
C	2.444027	-0.057681	0.011361
C	1.244798	-0.047171	-0.704855
Ni	1.201164	0.034097	3.947974
P	0.487079	-2.030360	4.124351
O	-1.089864	-2.118690	3.823924
C	-1.868858	-3.319303	4.081044

H	1.256060	-0.062722	-1.790569
P	1.806912	2.147155	4.029267
O	0.857642	3.278374	3.389040
C	0.757626	3.451450	1.946259
O	3.306879	2.377764	3.487591
C	4.006445	3.651359	3.612586
O	1.759814	2.748221	5.541441
C	2.464653	2.038543	6.587813
O	1.193897	-3.270041	3.356136
C	1.167043	-3.410614	1.913945
O	0.575652	-2.576768	5.649466
C	1.856479	-2.959175	6.206511
Br	2.548046	6.825679	1.801775
H	1.687271	-3.134551	7.268151
H	3.394030	-0.080630	-0.515594
H	3.375439	-0.021408	1.947954
H	-0.938511	0.017106	1.902757
H	-0.907015	-0.005059	-0.559999
H	5.000594	3.480616	3.200960
H	3.495465	4.439598	3.051951
H	4.084072	3.930385	4.665365
H	2.457692	2.695254	7.457113
H	1.955982	1.102854	6.838652
H	3.498091	1.834024	6.295526
H	-0.275340	3.254259	1.657092
H	1.042122	4.484445	1.732059
H	1.421120	2.766513	1.416154
H	1.452797	-4.441816	1.707538
H	0.163646	-3.222204	1.526580
H	1.877555	-2.723869	1.453326
H	2.215435	-3.871564	5.727615
H	2.592309	-2.159445	6.082365
H	-2.830482	-3.162718	3.594583
H	-1.374235	-4.195619	3.657994
H	-2.003315	-3.446655	5.155947

Table S49. XYZ Coordinates of A_mono_IV

61

scf done: -6371.995970

C	-0.633605	1.976143	0.530960
C	-0.949515	2.324260	1.858466
C	-0.927808	3.655040	2.289326
C	-0.578423	4.674545	1.401596
C	-0.254975	4.349130	0.083194
C	-0.285142	3.016677	-0.342633
H	-0.556922	5.708415	1.733776
Ni	-0.764384	0.098756	0.161916
P	-2.547750	0.610358	-0.970072
O	-2.211132	1.521332	-2.260403
C	-2.863714	2.766024	-2.607155

P	-1.213595	-2.060957	0.307849
O	-2.729431	-2.209982	0.867727
C	-3.211572	-3.304515	1.685503
P	1.298622	-0.014428	0.830343
O	1.474002	0.003211	2.437398
C	2.187858	1.015078	3.189023
O	-0.396451	-2.916682	1.422775
C	0.497594	-4.011287	1.131934
O	-1.079229	-2.880461	-1.080173
C	-1.920383	-3.990487	-1.470735
O	2.344753	1.141952	0.402408
C	2.627113	1.381474	-1.000875
O	1.983423	-1.397495	0.337157
C	3.339572	-1.768570	0.712004
O	-3.743107	1.473810	-0.288098
C	-4.360203	0.978848	0.924086
O	-3.273550	-0.691321	-1.616244
C	-4.330292	-0.563331	-2.596994
Br	6.193697	-0.101398	-1.397295
H	0.020706	5.131521	-0.619171
H	-0.041015	2.795479	-1.377003
H	-1.209518	1.553139	2.581538
H	-1.179702	3.891509	3.319825
H	-4.689184	-1.572920	-2.794373
H	-5.146695	0.048333	-2.205711
H	-3.937897	-0.122294	-3.515870
H	-5.266751	1.566397	1.066584
H	-4.613732	-0.078839	0.825583
H	-3.688815	1.125649	1.773528
H	-2.412861	3.081225	-3.548010
H	-3.936845	2.620157	-2.739960
H	-2.679191	3.515739	-1.836452
H	-4.162924	-2.971030	2.099064
H	-3.369931	-4.196456	1.075149
H	-2.507120	-3.522523	2.488281
H	-1.661433	-4.219111	-2.504037
H	-1.727330	-4.865882	-0.845369
H	-2.970870	-3.700688	-1.408613
H	1.246633	-4.021516	1.923609
H	-0.059808	-4.951725	1.143065
H	0.984105	-3.876320	0.166003
H	3.424232	-2.835520	0.505730
H	4.072583	-1.214878	0.118576
H	3.494036	-1.591908	1.778913
H	2.163686	0.682865	4.226791
H	3.219475	1.096308	2.845612
H	1.683133	1.978133	3.094335
H	2.562646	2.458050	-1.161467
H	3.635833	1.018912	-1.217528
H	1.902406	0.879820	-1.649100

Table S50. XYZ Coordinates of A_mono_TS2

61
scf done: -6371.917244

C	-2.498404	1.831369	-1.450144
C	-1.916581	1.549395	-0.198309
C	-2.271833	2.378733	0.875622
C	-3.167692	3.441080	0.715338
C	-3.734595	3.703255	-0.534029
C	-3.394407	2.891866	-1.619467
Ni	-0.744009	0.047396	-0.242958
P	-2.380540	-1.061233	0.641057
O	-3.771817	-1.193236	-0.175092
C	-4.937915	-0.366421	0.049952
H	-4.430522	4.527732	-0.660503
P	0.993132	1.373922	-0.229295
O	0.848151	2.969115	0.183941
C	0.361216	3.951745	-0.744155
O	1.891568	0.819488	0.895031
O	1.774295	1.482277	-1.671671
C	3.097850	2.062051	-1.733602
P	0.324779	-1.731257	-1.021253
O	1.400659	-1.534048	-2.225946
C	2.824791	-1.528643	-1.983698
O	-0.798726	-2.566461	-1.838953
C	-0.466416	-3.742216	-2.613494
O	1.057677	-2.835622	-0.080651
C	1.417637	-2.567416	1.296616
O	-2.931960	-0.509738	2.076166
C	-2.010161	-0.422002	3.181902
O	-2.002931	-2.609568	0.955834
C	-2.966631	-3.530569	1.519951
C	3.402614	1.976284	2.009500
Br	4.767056	-0.154232	1.029048
H	-3.421669	4.064088	1.569366
H	-1.843514	2.209898	1.859522
H	-2.258405	1.217207	-2.316715
H	-3.824767	3.083297	-2.599239
H	3.090444	-0.966741	-1.086482
H	3.184277	-2.556729	-1.892713
H	3.279141	-1.057067	-2.855666
H	-1.393010	-4.065321	-3.087461
H	0.279339	-3.500940	-3.372817
H	-0.091474	-4.531324	-1.957287
H	0.557912	-2.183046	1.849497
H	1.714060	-3.528868	1.717553
H	2.248646	-1.860380	1.343376
H	3.067840	3.128646	-1.490946
H	3.771544	1.543015	-1.046117
H	3.442938	1.936656	-2.761150

H	0.789512	4.911214	-0.444790
H	0.676907	3.723355	-1.765545
H	-0.727711	4.009746	-0.702069
H	-4.722086	0.673098	-0.200785
H	-5.709471	-0.755646	-0.614675
H	-5.265622	-0.446390	1.087111
H	-1.121753	0.158293	2.914103
H	-2.540650	0.084094	3.988273
H	-1.708206	-1.420006	3.510428
H	-2.418288	-4.444401	1.747152
H	-3.404525	-3.122987	2.434034
H	-3.754656	-3.736904	0.793070
H	3.221199	1.401723	2.904607
H	4.425267	2.239710	1.795483
H	2.639830	2.665781	1.688732

Table S51. XYZ Coordinates of A_mono_V

56

scf done: -3760.362634

C	3.031907	0.465955	0.408603
C	1.945758	0.147094	-0.419669
C	2.208320	0.042542	-1.801631
C	3.487039	0.251247	-2.327530
C	4.550197	0.572774	-1.480006
C	4.314790	0.676603	-0.107712
Ni	0.092928	-0.108855	-0.030047
P	-0.062472	2.040413	0.220447
O	-1.556394	2.519632	0.657448
C	-1.860224	3.912755	0.912388
H	5.545995	0.735626	-1.882960
P	0.511459	-2.176051	0.576251
O	-0.650052	-2.954562	1.166098
P	-2.044120	-0.479255	-0.399472
O	-3.182408	-0.519936	0.760802
C	-2.808476	-0.684388	2.151716
O	1.722396	-2.060149	1.697864
C	1.986591	-3.200830	2.528242
O	1.323476	-3.125621	-0.526832
C	0.549237	-3.797992	-1.527104
O	-2.226393	-1.847495	-1.257303
C	-3.493665	-2.196765	-1.853793
O	-2.692279	0.603171	-1.435760
C	-3.890437	1.365224	-1.192648
O	0.257078	3.047112	-1.010875
C	1.571124	3.582910	-1.292701
O	0.891067	2.695214	1.376888
C	0.796518	2.179840	2.720610
H	5.131713	0.922106	0.566809
H	2.890604	0.541123	1.482028
H	1.406878	-0.209764	-2.495320

H	3.651076	0.160915	-3.398642
H	-3.351566	-3.165101	-2.333599
H	-4.268526	-2.277835	-1.087041
H	-3.782708	-1.452960	-2.599980
H	-3.921047	2.134153	-1.965661
H	-4.775738	0.729354	-1.275090
H	-3.860384	1.831651	-0.208205
H	-2.280242	0.206415	2.502845
H	-3.743345	-0.792911	2.702331
H	-2.183212	-1.570657	2.272058
H	1.237926	-4.428261	-2.094343
H	-0.225389	-4.422196	-1.072408
H	0.071741	-3.086421	-2.209597
H	2.708209	-2.884219	3.284173
H	1.072530	-3.552723	3.014482
H	2.416293	-4.018462	1.939686
H	1.440352	4.266407	-2.132105
H	1.950992	4.127793	-0.427200
H	2.259738	2.783030	-1.568448
H	0.933798	1.094414	2.740773
H	1.594258	2.654558	3.291950
H	-0.171596	2.432252	3.161862
H	-2.879346	3.938327	1.297344
H	-1.173306	4.328621	1.652641
H	-1.794717	4.485362	-0.014682

Table S52. XYZ Coordinates of A_mono_TS3

56

scf done: -3760.3307711

C	2.147397	-1.821006	3.433407
C	1.337483	-2.646957	2.647031
C	0.930810	-2.229563	1.381499
C	1.285043	-0.954136	0.885464
C	2.158990	-0.166050	1.666991
C	2.560946	-0.582590	2.936594
Ni	-0.078284	0.055004	-0.035565
O	0.859199	-0.330035	-2.336027
P	1.552475	-0.778334	-1.051266
O	1.858062	-2.385186	-1.156340
C	2.334120	-2.897811	-2.417029
P	-0.041058	2.191521	0.143569
O	1.437504	2.891199	0.264826
C	1.600684	4.312840	0.459227
P	-2.034683	-0.845008	-0.060486
O	-2.987319	-0.685197	1.271458
C	-2.566848	-1.278768	2.512864
O	-0.691945	2.830301	1.515655
C	-2.119028	2.803738	1.699744
O	-0.809934	3.152360	-0.953467
C	-0.993383	2.680606	-2.304035

O	-2.294898	-2.466594	-0.286049
C	-1.751999	-3.077145	-1.471058
O	-3.017420	-0.166969	-1.175357
C	-4.402776	-0.553136	-1.318331
O	3.060992	-0.144961	-0.991893
C	3.392844	0.970946	-1.843031
H	2.469981	-2.150332	4.416770
H	1.024628	-3.618563	3.018527
H	0.313679	-2.885948	0.780709
H	2.510659	0.782624	1.280551
H	3.206110	0.057107	3.531955
H	2.669048	4.515783	0.375669
H	1.058486	4.869999	-0.308637
H	1.242821	4.606285	1.448408
H	-2.309264	3.170359	2.709626
H	-2.610087	3.457172	0.973820
H	-2.514635	1.787877	1.604124
H	-1.789580	1.931809	-2.339808
H	-1.279357	3.550086	-2.898321
H	-0.074663	2.246337	-2.705744
H	4.461391	1.145671	-1.706519
H	3.186428	0.739983	-2.889756
H	2.835695	1.860751	-1.541631
H	2.457418	-3.973449	-2.284765
H	1.614268	-2.699049	-3.213807
H	3.299136	-2.450338	-2.673920
H	-3.315620	-1.007605	3.258265
H	-2.517963	-2.367205	2.422876
H	-1.591069	-0.893519	2.824589
H	-0.676407	-2.896441	-1.555100
H	-1.930829	-4.149802	-1.383230
H	-2.254669	-2.699536	-2.367074
H	-4.746933	-0.127744	-2.261550
H	-4.505006	-1.640446	-1.343998
H	-4.992343	-0.149814	-0.491915

Table S53. XYZ Coordinates of A_mono_VI

56

scf done: -3760.363177

C	0.851562	-1.495701	1.145051
C	1.700571	-0.339002	0.853944
C	2.282013	0.385042	1.973669
C	1.974093	0.066447	3.263676
C	1.113854	-1.044868	3.551039
C	0.610536	-1.810643	2.536735
Ni	-0.187549	-0.034816	0.361349
P	-2.018049	-0.975316	-0.115675
O	-2.106927	-2.590579	-0.459034
C	-1.946114	-3.566364	0.583603
P	2.685697	-0.309662	-0.645785

O	3.827835	-1.434707	-0.351463
C	4.974591	-1.519182	-1.225633
H	0.909647	-1.308187	4.585030
P	-0.434552	2.048770	0.004519
O	-1.775218	2.742897	0.661039
C	-3.049324	2.688393	-0.002324
O	-0.559615	2.702858	-1.500628
C	0.328514	2.237020	-2.537633
O	0.670485	2.977213	0.767129
C	0.566588	4.415596	0.796752
O	3.230928	1.022477	-1.043720
O	1.806913	-0.925522	-1.868636
C	1.597097	-2.327837	-2.133796
O	-2.627042	-0.452987	-1.543395
C	-3.410543	-1.233380	-2.471749
O	-3.199398	-0.778460	1.004776
C	-4.565039	-1.206405	0.820960
H	0.853602	-2.346567	0.467604
H	2.962180	1.203053	1.758370
H	0.025427	-2.696102	2.771060
H	2.406706	0.634397	4.082297
H	-4.611282	-2.233222	0.449189
H	-5.046135	-1.152691	1.798204
H	-5.079819	-0.537409	0.125917
H	-0.896201	-3.854197	0.680204
H	-2.313807	-3.191364	1.542391
H	-2.530712	-4.442389	0.295810
H	-3.624827	-0.571937	-3.312172
H	-2.846086	-2.101179	-2.815800
H	-4.349516	-1.566020	-2.023854
H	0.120941	2.848438	-3.417143
H	1.374099	2.350305	-2.241493
H	0.138793	1.185348	-2.770642
H	1.506985	4.785322	1.207673
H	0.425347	4.814334	-0.211171
H	-0.265083	4.725855	1.432776
H	-3.790320	3.036854	0.718958
H	-3.051431	3.344192	-0.876524
H	-3.297867	1.671113	-0.313005
H	1.761854	-2.485640	-3.201566
H	2.290152	-2.949193	-1.563489
H	0.566374	-2.590047	-1.882944
H	5.665721	-2.223930	-0.762661
H	4.684699	-1.889567	-2.213514
H	5.451437	-0.541830	-1.327462

Table S54. XYZ Coordinates of A_para_I_P(O)Ph_2

68
scf done: -6564.493931

C	0.996178	-0.844573	1.099102

C	1.904937	-0.334471	0.199713
C	1.623243	-0.408368	-1.209382
C	0.466488	-0.976551	-1.666491
C	-0.551031	-1.403910	-0.744092
C	-0.269435	-1.383176	0.686978
P	3.457358	0.330230	0.871465
O	3.335467	0.718035	2.328702
Ni	-1.790721	-0.275977	0.102154
P	-3.002912	0.470696	-1.478313
O	-4.331451	-0.375327	-1.924361
C	-5.164114	-0.938370	-0.891090
Br	-1.619636	-2.982639	-1.383689
C	3.907632	1.759422	-0.181393
C	4.620837	1.632345	-1.382854
C	4.910490	2.761520	-2.151218
C	4.497511	4.025059	-1.722866
C	3.796575	4.160450	-0.521922
C	3.503152	3.033405	0.246142
C	4.766125	-0.920728	0.597732
C	4.630755	-1.991433	-0.297898
C	5.669351	-2.912001	-0.455917
C	6.846105	-2.774702	0.282204
C	6.984944	-1.715904	1.183917
C	5.951104	-0.793905	1.341661
P	-2.607456	0.491270	1.929975
O	-2.463903	-0.434484	3.288447
C	-3.060200	-1.744061	3.255535
O	-2.027799	1.894417	2.537634
C	-0.643575	1.988330	2.927724
O	-4.196424	0.840786	1.889172
C	-4.884438	1.407549	3.027286
O	-3.538897	1.993943	-1.201961
C	-4.481268	2.637413	-2.086964
O	-2.423893	0.569975	-3.014461
C	-1.366096	1.521451	-3.236133
H	2.339629	-0.019389	-1.926184
H	0.284806	-1.068840	-2.732070
H	-0.691557	-2.147201	1.336003
H	1.247970	-0.865167	2.155913
H	2.971508	3.136677	1.186835
H	3.482519	5.142740	-0.181938
H	4.727062	4.902532	-2.319939
H	5.464104	2.654576	-3.079120
H	4.962257	0.657027	-1.716142
H	6.056068	0.020093	2.052531
H	7.896044	-1.610887	1.765179
H	7.651196	-3.493023	0.159733
H	5.555215	-3.738065	-1.151277
H	3.715636	-2.114603	-0.868068
H	-2.580772	-2.337086	4.035259

H	-4.134322	-1.680065	3.452987
H	-2.905825	-2.233374	2.287328
H	-0.477529	3.021355	3.235047
H	-0.434683	1.318710	3.765973
H	0.018619	1.747409	2.091698
H	-5.950990	1.296382	2.830001
H	-4.621258	0.878286	3.946062
H	-4.632757	2.465333	3.128896
H	-5.898808	-1.566099	-1.396240
H	-4.576183	-1.549892	-0.200521
H	-5.672163	-0.150873	-0.328622
H	-0.955598	1.308492	-4.223879
H	-1.750753	2.544930	-3.216636
H	-0.572625	1.415425	-2.490799
H	-4.470175	3.698728	-1.836637
H	-4.196423	2.502948	-3.133316
H	-5.484048	2.232188	-1.930142

Table S55. XYZ Coordinates of A_para_TS1_P(O)Ph$_2$

68

scf done: -6564.487182

C	4.959586	1.755167	-0.550096
C	4.180821	1.471063	0.581555
C	3.830229	2.512862	1.454751
C	4.241071	3.819895	1.192030
C	5.006770	4.097323	0.056581
C	5.367008	3.064826	-0.811814
P	3.574730	-0.207714	0.986615
C	4.774399	-1.377508	0.250767
C	4.660453	-1.875056	-1.055875
C	5.617836	-2.759359	-1.557078
C	6.689938	-3.158683	-0.756523
C	6.803952	-2.675847	0.549695
C	5.851142	-1.789940	1.052088
C	1.999360	-0.406932	0.096474
C	1.730532	0.213279	-1.159899
C	0.526302	0.034353	-1.803198
C	-0.480764	-0.715795	-1.149124
C	-0.190322	-1.491472	0.016438
C	1.051609	-1.257371	0.653267
Ni	-1.997267	-0.310261	-0.212103
P	-3.359153	-0.916143	1.350263
O	-3.289172	-0.171419	2.804586
C	-2.027971	-0.124567	3.496651
Br	-1.971149	-1.709589	-2.346721
O	3.423917	-0.416200	2.476512
P	-2.337649	1.729345	-0.695308
O	-3.381899	2.490617	0.319664
C	-3.694508	3.892017	0.169577
O	-1.078064	2.784948	-0.779999

C	-0.244632	2.883545	0.387393
O	-2.889589	2.126923	-2.195815
C	-4.175259	1.604357	-2.578941
O	-4.933124	-0.718699	0.994591
C	-5.979259	-1.014281	1.947992
O	-3.278863	-2.469504	1.907939
C	-3.481583	-3.530157	0.954487
H	2.470400	0.869492	-1.607998
H	0.310724	0.541874	-2.736857
H	-0.719417	-2.402215	0.266140
H	1.290622	-1.804125	1.561170
H	3.248358	2.292250	2.344207
H	3.968607	4.619473	1.874246
H	5.327365	5.114573	-0.147115
H	5.970191	3.276200	-1.689492
H	5.258248	0.957519	-1.223243
H	5.930656	-1.425518	2.071521
H	7.631852	-2.991720	1.177220
H	7.431357	-3.849248	-1.147068
H	5.521572	-3.140429	-2.569175
H	3.822142	-1.584340	-1.681206
H	-3.073117	-4.440659	1.394629
H	-4.549029	-3.667357	0.756976
H	-2.968191	-3.321124	0.010583
H	-2.172590	0.514209	4.368551
H	-1.728990	-1.124897	3.821641
H	-1.246997	0.303669	2.859829
H	-6.913159	-1.032354	1.385760
H	-5.813137	-1.985301	2.420334
H	-6.020212	-0.235951	2.712887
H	-4.174494	1.514935	-3.666051
H	-4.361808	0.617993	-2.142055
H	-4.969172	2.290090	-2.268567
H	0.585535	3.541156	0.127079
H	-0.798451	3.313504	1.228059
H	0.152244	1.905704	0.676987
H	-4.570860	4.084392	0.789576
H	-2.860835	4.511115	0.510749
H	-3.920552	4.134046	-0.871774

Table S56. XYZ Coordinates of A_para_IIa_P(O)Ph$_2$

68

scf done: -6564.541773

C	-4.325864	-2.832891	-0.075309
C	-5.060001	-1.640685	-0.182693
C	-6.403772	-1.627388	0.226294
C	-6.996519	-2.779723	0.742477
C	-6.255453	-3.958710	0.852154
C	-4.920938	-3.984293	0.441492
P	-4.335498	-0.177841	-1.005232

C	-5.037916	1.283289	-0.152353
C	-5.467090	2.352470	-0.951113
C	-5.970717	3.514586	-0.363573
C	-6.049073	3.616969	1.026437
C	-5.626358	2.554012	1.829708
C	-5.123031	1.392049	1.244826
C	-2.551576	-0.201429	-0.639036
C	-2.025213	-0.401877	0.648848
C	-0.648901	-0.367667	0.860818
C	0.256899	-0.136645	-0.191129
C	-0.284954	0.074655	-1.469475
C	-1.663947	0.038086	-1.695822
Ni	2.137118	-0.297794	0.027650
P	4.357383	-0.637331	0.007696
O	4.874147	-1.198965	1.434849
C	6.249674	-1.249372	1.885693
P	2.148580	1.804477	0.181434
O	1.083011	2.531766	1.170775
O	3.497304	2.525173	0.739781
C	3.883032	2.364479	2.119288
O	2.013634	2.509541	-1.266214
C	2.215716	3.933946	-1.459707
Br	1.846129	-2.617482	0.140604
O	5.459466	0.552905	-0.207619
C	5.464621	1.280751	-1.451725
O	4.733771	-1.677778	-1.180960
O	-4.601622	-0.118097	-2.494835
C	-0.256277	2.923626	0.797947
H	-2.684782	-0.602211	1.488844
H	-0.276216	-0.538938	1.867615
H	0.370449	0.273503	-2.314113
H	-2.060178	0.193779	-2.694793
H	-5.407706	2.261397	-2.030837
H	-6.303046	4.336512	-0.990571
H	-6.441529	4.520053	1.484275
H	-5.692467	2.628208	2.910925
H	-4.812181	0.569298	1.881350
H	-6.987172	-0.715336	0.147781
H	-8.034354	-2.756329	1.060515
H	-6.716733	-4.854577	1.256804
H	-4.342107	-4.899017	0.525162
H	-3.287101	-2.861007	-0.388506
H	-0.358492	3.987156	1.025261
H	-0.440185	2.753276	-0.263485
H	-0.968697	2.344934	1.385181
H	6.206374	-1.393276	2.965231
H	6.767237	-2.094249	1.426348
H	6.767112	-0.318535	1.652750
H	6.144278	2.121753	-1.315534
H	5.822489	0.648245	-2.268122

H	4.468200	1.659840	-1.691521
C	6.068750	-2.165091	-1.441914
H	6.109457	-2.416677	-2.502151
H	6.822751	-1.407426	-1.217154
H	6.251518	-3.062959	-0.846328
H	1.875325	4.150135	-2.471729
H	1.631783	4.512146	-0.740760
H	3.274241	4.175615	-1.356001
H	4.826029	2.897238	2.239371
H	3.126249	2.794128	2.778802
H	4.034425	1.308594	2.362545

Table S57. XYZ Coordinates of A_para_IIb_P(O)Ph$_2$

68

scf done: -6564.551057

C	-4.102118	2.087024	0.330345
C	-4.693487	1.188753	-0.570683
C	-5.878613	1.553853	-1.227879
C	-6.468638	2.793165	-0.979132
C	-5.880116	3.678505	-0.072591
C	-4.697093	3.325259	0.580290
P	-3.988104	-0.459402	-0.941772
O	-4.330176	-0.917155	-2.342285
C	-2.194700	-0.333635	-0.655979
C	-1.386320	0.076581	-1.729028
C	-0.008992	0.212464	-1.566522
C	0.623516	-0.061169	-0.340057
C	-0.200820	-0.471519	0.722034
C	-1.582261	-0.608583	0.575061
Ni	2.480005	0.114288	-0.094279
Br	4.771687	0.356574	0.378267
P	2.601962	-2.050472	-0.324458
O	1.527645	-2.856121	-1.246387
C	1.592267	-2.650512	-2.672536
O	2.267513	-2.892383	1.028232
C	2.953321	-2.559594	2.254651
O	4.036791	-2.596024	-0.852375
P	2.236401	2.248846	0.308353
O	3.150161	3.296641	-0.538929
C	3.156615	3.158740	-1.975766
O	2.701825	2.791507	1.770852
C	2.181154	2.113512	2.933198
O	0.723539	2.814286	0.137596
C	-4.652954	-1.597447	0.328596
C	-4.848747	-2.935927	-0.044777
C	-5.340469	-3.859922	0.877663
C	-5.641911	-3.454907	2.180128
C	-5.457684	-2.122657	2.556987
C	-4.969130	-1.195170	1.635081
H	-1.838844	0.283822	-2.694750

H	0.576448	0.553300	-2.417606
H	0.235965	-0.698002	1.691977
H	-2.177335	-0.938919	1.421399
C	0.409694	4.207063	0.367307
H	-4.626306	-3.243429	-1.061638
H	-5.491152	-4.893117	0.579606
H	-6.025514	-4.173818	2.897877
H	-5.700302	-1.803007	3.565892
H	-4.846451	-0.158873	1.934332
H	-6.325919	0.870038	-1.942385
H	-7.383728	3.069076	-1.494432
H	-6.338965	4.643705	0.119764
H	-4.233226	4.015173	1.278729
H	-3.172988	1.827827	0.828700
H	2.564694	2.652329	3.799303
H	1.087702	2.135174	2.942744
H	2.530934	1.077595	2.964649
H	-0.660089	4.311560	0.186960
H	0.642643	4.488751	1.396670
H	0.967016	4.845350	-0.321833
H	3.745943	3.990821	-2.360612
H	3.623430	2.214114	-2.270008
H	2.141377	3.213893	-2.379162
C	4.335442	-4.011343	-0.904290
H	5.206028	-4.116988	-1.551500
H	4.571544	-4.380988	0.096183
H	3.497115	-4.577887	-1.315420
H	2.540168	-3.215266	3.020764
H	4.028074	-2.734141	2.158816
H	2.778805	-1.516265	2.529216
H	0.728980	-3.163448	-3.095793
H	1.534121	-1.588074	-2.921438
H	2.510672	-3.076244	-3.084672

Table S58. XYZ Coordinates of A_para_III_P(O)Ph$_2$

68

scf done: -6564.487518

C	-5.892867	-0.735563	-1.747401
C	-4.801464	-0.443912	-0.914588
C	-4.827320	0.716599	-0.126548
C	-5.936820	1.563411	-0.160829
C	-7.022467	1.262652	-0.986365
C	-6.998289	0.114480	-1.781983
P	-3.397920	-1.617104	-0.922170
O	-3.267158	-2.333687	-2.246883
C	-3.680585	-2.776987	0.464040
C	-4.384943	-2.430859	1.627090
C	-4.551104	-3.364738	2.651384
C	-4.023139	-4.650920	2.519357
C	-3.330063	-5.005686	1.359420

C	-3.159609	-4.073985	0.335348
C	-1.910996	-0.646546	-0.502618
C	-1.201956	-0.051126	-1.558368
C	-0.067316	0.718819	-1.307463
C	0.389504	0.914369	0.004471
C	-0.306265	0.302629	1.055798
C	-1.445682	-0.467193	0.807192
Ni	1.868652	1.956361	0.321656
P	3.150864	0.201204	0.206937
O	4.678557	0.709983	0.115362
C	5.725558	0.092595	-0.688366
P	0.739542	3.852977	0.420715
O	1.575418	5.147938	-0.092018
C	2.078658	5.131623	-1.449531
O	0.406615	4.468180	1.883122
C	-0.300674	3.650728	2.846196
O	-0.644471	3.844346	-0.404698
C	-1.496015	5.017659	-0.492244
O	3.039013	-0.751704	-1.086542
C	2.243766	-1.966916	-1.136274
O	3.009890	-0.712939	1.527151
C	4.046912	-1.610693	2.017533
Br	5.488202	-3.857434	-0.722676
H	-1.533632	-0.200787	-2.581656
H	0.458500	1.165802	-2.146245
H	0.037195	0.410423	2.080398
H	-1.959965	-0.933934	1.641783
H	-2.633763	-4.350923	-0.572876
H	-2.925848	-6.007541	1.250906
H	-4.156827	-5.376725	3.315863
H	-5.098310	-3.089288	3.547776
H	-4.813574	-1.439325	1.733675
H	-5.863970	-1.620670	-2.375047
H	-7.837359	-0.116530	-2.431237
H	-7.882513	1.924925	-1.014669
H	-5.949455	2.460230	0.451046
H	-3.980850	0.968122	0.505084
H	-2.407430	4.690931	-0.990579
H	-1.728360	5.395691	0.504901
H	-1.006441	5.796294	-1.080264
H	2.670800	6.038263	-1.565504
H	2.713249	4.257319	-1.619772
H	1.254227	5.134570	-2.166807
H	-0.568977	4.315378	3.666500
H	-1.206662	3.224951	2.407983
H	0.345860	2.851982	3.217934
H	2.942591	-2.806644	-1.141628
H	1.571097	-2.031429	-0.279458
H	1.660688	-1.930586	-2.056729
H	6.666259	0.427726	-0.251100

H	5.661464	-0.998144	-0.669614
H	5.645111	0.453391	-1.715711
H	3.615910	-2.111447	2.884150
H	4.334999	-2.342299	1.256717
H	4.915512	-1.023355	2.323776

Table S59. XYZ Coordinates of A_para_IV_P(O)Ph$_2$

84

scf done: -7251.323424

C	-5.414996	2.007258	-0.698164
C	-5.196562	1.629674	0.636141
C	-5.311102	2.592159	1.650135
C	-5.631942	3.913141	1.334997
C	-5.840763	4.283199	0.004709
C	-5.734054	3.329284	-1.010502
P	-4.710280	-0.061283	1.135553
C	-5.613614	-1.196752	0.024379
C	-6.982449	-0.986447	-0.214707
C	-7.716058	-1.901420	-0.968872
C	-7.092436	-3.038262	-1.489580
C	-5.734656	-3.259445	-1.251862
C	-4.997943	-2.344920	-0.497357
C	-2.936955	-0.237667	0.739567
C	-2.395811	0.028645	-0.526929
C	-1.026780	-0.114123	-0.755192
C	-0.147314	-0.517643	0.262423
C	-0.703522	-0.770746	1.530707
C	-2.073984	-0.638406	1.767735
Ni	1.740828	-0.818758	0.170904
P	1.248836	-2.573846	-1.022681
O	0.610444	-2.147798	-2.442822
C	-0.583296	-2.714622	-3.035757
P	3.801945	-1.453441	0.649667
O	3.711025	-2.958934	1.245646
O	4.545336	-0.684184	1.872309
C	5.765475	0.077167	1.750569
O	4.834904	-1.436419	-0.593686
C	5.894401	-2.399643	-0.801718
P	1.955754	1.256258	0.777922
O	1.689658	1.528830	2.347995
C	0.637860	2.368658	2.884815
O	0.988616	2.366605	0.110109
C	1.000033	2.569918	-1.327371
O	3.461511	1.781049	0.501166
O	0.150163	-3.651656	-0.503695
C	0.362313	-4.311623	0.767498
O	2.551762	-3.452162	-1.428874
O	-4.973347	-0.307019	2.605832
Br	3.122923	5.756173	-1.915681
H	-3.034994	0.347685	-1.345313

H	-0.646761	0.087664	-1.751299
H	-0.068878	-1.069039	2.362948
H	-2.481790	-0.838727	2.753710
H	-7.475149	-0.104155	0.182507
H	-8.771812	-1.726459	-1.151776
H	-7.663791	-3.748962	-2.079059
H	-5.247448	-4.142218	-1.654476
H	-3.942888	-2.524833	-0.318305
H	-5.156894	2.295479	2.682628
H	-5.721564	4.650833	2.126665
H	-6.091140	5.310886	-0.240768
H	-5.904804	3.612441	-2.044637
H	-5.355228	1.272039	-1.494698
C	2.481558	-4.509982	-2.415136
H	3.461315	-4.986264	-2.423674
H	1.718112	-5.241761	-2.140989
H	2.264841	-4.090168	-3.399825
H	-0.323448	-5.158037	0.790264
H	1.392010	-4.665389	0.853503
H	0.130703	-3.627208	1.587001
H	-0.696605	-2.217053	-3.998600
H	-0.473631	-3.790075	-3.184062
H	-1.450000	-2.510180	-2.405531
C	4.615468	-3.522163	2.227645
H	4.121930	-4.414885	2.610602
H	5.562192	-3.799169	1.758438
H	4.792746	-2.813174	3.036470
H	6.303605	-2.191997	-1.789783
H	6.680070	-2.284147	-0.050701
H	5.486511	-3.411568	-0.775312
H	5.734939	0.839841	2.528507
H	6.624816	-0.578412	1.914266
H	5.836985	0.550627	0.771446
C	3.899982	3.118608	0.874154
H	4.987372	3.070595	0.932450
H	3.599008	3.846104	0.115196
H	3.496633	3.387505	1.852813
H	0.787242	2.373056	3.964309
H	0.714906	3.382594	2.491408
H	-0.339533	1.945941	2.646163
H	-0.036432	2.720533	-1.630599
H	1.598181	3.456072	-1.558186
H	1.401645	1.698262	-1.852906

Table S60. XYZ Coordinates of A_para_TS2_P(O)Ph_2

84

scf done: -7251.245786

C	-6.095707	-1.990548	1.234946
C	-5.729960	-0.663267	0.961815
C	-5.920716	0.318426	1.945766

C	-6.453629	-0.027446	3.189046
C	-6.807327	-1.351796	3.456146
C	-6.631250	-2.332402	2.476769
P	-5.017181	-0.313503	-0.687700
C	-5.463502	1.417290	-1.083918
C	-4.706523	2.521923	-0.665498
C	-5.114842	3.817178	-0.989517
C	-6.275243	4.018872	-1.739995
C	-7.027658	2.923065	-2.169906
C	-6.624484	1.627959	-1.844071
C	-3.205929	-0.369279	-0.499367
C	-2.533046	-0.020283	0.682003
C	-1.140044	-0.065724	0.742328
C	-0.359769	-0.475928	-0.352486
C	-1.053776	-0.837005	-1.524489
C	-2.446761	-0.782053	-1.604361
Ni	1.541758	-0.616903	-0.414446
P	1.209500	-2.720459	0.041131
O	2.548817	-3.467214	0.583812
C	2.594478	-4.894627	0.811201
O	-5.519724	-1.280030	-1.736732
P	1.695094	1.555512	-0.159652
O	2.359357	1.730201	1.218680
P	3.668367	-0.930377	-0.979629
O	4.813771	-1.215617	0.135125
C	4.611838	-0.984100	1.551757
O	0.354563	2.518023	-0.057609
C	-0.427375	2.787413	-1.230912
O	2.508714	2.297778	-1.377242
C	2.923926	3.676572	-1.245987
O	4.416341	0.106758	-1.980230
C	5.384623	1.076547	-1.524868
O	3.673533	-2.238314	-1.938520
C	4.880900	-2.711961	-2.578711
O	0.667680	-3.590016	-1.206714
C	-0.468015	-4.488012	-1.181333
O	0.100407	-3.107235	1.172023
C	0.341812	-2.692391	2.532992
C	2.442847	3.571036	2.423547
Br	5.039346	3.021499	1.862491
H	-3.092140	0.281222	1.563397
H	-0.660834	0.230407	1.671397
H	-0.505124	-1.182665	-2.397726
H	-2.949839	-1.074686	-2.521311
H	-7.199687	0.774237	-2.188104
H	-7.925500	3.076730	-2.760907
H	-6.588887	5.026880	-1.994350
H	-4.523204	4.666509	-0.661552
H	-3.793437	2.375381	-0.097133
H	-5.970994	-2.747022	0.466625

H	-6.914183	-3.361162	2.678381
H	-7.225484	-1.617669	4.422370
H	-6.598953	0.738576	3.944670
H	-5.667317	1.354423	1.743317
H	5.092378	1.546542	-0.583843
H	6.357962	0.593313	-1.408733
H	5.442485	1.835531	-2.305614
H	4.586198	-3.564692	-3.189898
H	5.310527	-1.930293	-3.207610
H	5.606645	-3.028797	-1.825841
H	3.675872	-1.434970	1.887512
H	5.445955	-1.476650	2.052964
H	4.623351	0.087136	1.764713
H	2.064621	4.329689	-1.067468
H	3.641806	3.773523	-0.427349
H	3.393324	3.949562	-2.192519
H	-1.215262	3.481467	-0.932541
H	0.179787	3.248649	-2.014991
H	-0.882539	1.871913	-1.616526
H	-1.388405	-3.920252	-1.035500
H	-0.480848	-4.974061	-2.156710
H	-0.361990	-5.235828	-0.394294
H	0.587651	-1.628070	2.584361
H	-0.582453	-2.874252	3.080624
H	1.153919	-3.276040	2.973228
H	3.558236	-5.101943	1.275428
H	1.788162	-5.210752	1.477582
H	2.520014	-5.425527	-0.140799
H	2.557766	2.998947	3.331130
H	3.014761	4.480440	2.340193
H	1.485768	3.546073	1.930367

Table S61. XYZ Coordinates of A_para_V_P(O)Ph$_2$

79

scf done: -4639.691535

C	1.612917	0.176831	-1.623499
C	2.551853	0.297994	-0.587662
C	2.087157	0.303897	0.736450
C	0.725774	0.170579	1.011091
C	-0.231190	0.044628	-0.009727
C	0.252576	0.061027	-1.334818
P	4.309102	0.424966	-1.042253
O	4.515565	1.033980	-2.411446
Ni	-2.125917	-0.140461	0.118690
P	-2.226161	2.009380	-0.173956
O	-3.640354	2.639095	0.340094
C	-3.960295	4.038488	0.155194
C	5.126053	1.406004	0.270456
C	5.583452	0.851990	1.475532
C	6.180851	1.664922	2.440697

C	6.333670	3.033395	2.207169
C	5.891277	3.589829	1.004519
C	5.290688	2.780535	0.039879
C	5.018703	-1.259581	-0.930326
C	6.148145	-1.540187	-1.715537
C	6.744727	-2.800000	-1.666939
C	6.218311	-3.791388	-0.834818
C	5.090205	-3.522723	-0.056974
C	4.488873	-2.263401	-0.106045
P	-1.813735	-2.136000	1.005962
O	-2.995118	-3.072332	1.167839
P	-4.223527	-0.552186	-0.436546
O	-5.447822	-0.728021	0.617893
C	-5.217297	-0.906481	2.036939
O	-1.176489	-1.763563	2.486372
C	-1.076969	-2.802206	3.474684
O	-0.521431	-3.000560	0.409745
C	-0.715413	-3.664259	-0.843185
O	-4.214636	-1.871658	-1.381365
C	-5.394301	-2.292370	-2.101490
O	-4.891709	0.514988	-1.477599
C	-6.064550	1.302460	-1.198126
O	-2.036819	2.490995	-1.706714
C	-1.133961	3.527316	-2.160657
O	-1.126156	2.966000	0.560257
C	-1.104606	2.997667	2.002141
H	2.785231	0.415592	1.561505
H	0.412164	0.141671	2.048969
H	-0.438947	-0.012426	-2.172042
H	1.949493	0.191178	-2.656231
H	6.547018	-0.773114	-2.371912
H	7.616569	-3.009121	-2.279515
H	6.682353	-4.772449	-0.797718
H	4.672968	-4.294175	0.583121
H	3.602101	-2.069680	0.489360
H	4.958454	3.206829	-0.901478
H	6.017140	4.651868	0.816555
H	6.802204	3.663146	2.957549
H	6.532722	1.227958	3.370323
H	5.486434	-0.213423	1.660262
H	-5.118092	-3.200704	-2.636693
H	-6.208223	-2.508983	-1.404970
H	-5.704323	-1.521476	-2.810058
H	-6.107334	2.065737	-1.976256
H	-6.962495	0.681745	-1.243548
H	-5.994321	1.779543	-0.220309
H	-4.713044	-0.029224	2.452351
H	-6.204922	-1.002193	2.488890
H	-4.621470	-1.803358	2.208207
H	0.169109	-4.278573	-1.025120

H	-1.602525	-4.304906	-0.820340
H	-0.814540	-2.941575	-1.662742
H	-0.833800	-2.318484	4.422646
H	-2.022354	-3.343165	3.567952
H	-0.281976	-3.507975	3.213228
H	-1.314230	3.628298	-3.230844
H	-1.336510	4.474674	-1.658883
H	-0.099984	3.226261	-1.984042
H	-1.063909	1.987760	2.418323
H	-0.203116	3.539753	2.286881
H	-1.984777	3.517564	2.388665
H	-4.893070	4.216258	0.689742
H	-3.173256	4.676317	0.564094
H	-4.096425	4.252178	-0.907631

Table S62. XYZ Coordinates of A_para_TS3_P(O)Ph_2

79

scf done: -4639.650089

C	1.682244	-0.007845	0.776259
C	2.342684	0.098549	-0.452460
C	1.661835	0.661353	-1.550477
C	0.353879	1.102837	-1.418186
C	-0.346828	0.974415	-0.192230
C	0.369664	0.453354	0.913688
P	4.060835	-0.469384	-0.729834
C	5.144686	0.951675	-0.349421
C	6.379789	1.015538	-1.013021
C	7.259434	2.067776	-0.759885
C	6.912408	3.064094	0.155982
C	5.681307	3.011306	0.813555
C	4.796995	1.961262	0.560553
Ni	-2.103583	0.147600	-0.010866
P	-2.193814	-1.404992	-1.489693
O	-3.684987	-2.056152	-1.633929
C	-3.962014	-3.138852	-2.551498
P	-1.573564	2.311001	0.383156
O	-1.401060	2.707860	1.822109
C	4.395685	-1.751805	0.529696
C	4.796760	-1.464135	1.843087
C	5.022183	-2.500748	2.750098
C	4.856437	-3.829354	2.351839
C	4.467800	-4.122827	1.042761
C	4.238647	-3.089375	0.133975
O	4.264027	-0.988745	-2.134259
P	-3.638068	-0.211906	1.409870
O	-4.092284	-1.781973	1.690235
C	-3.069451	-2.739936	2.002104
O	-3.085076	2.431000	-0.271851
C	-3.642816	3.668966	-0.779419
O	-3.374470	0.318680	2.932694

C	-4.350895	0.141179	3.980348
O	-5.137923	0.386068	1.158995
C	-5.733561	0.305697	-0.151485
O	-0.958863	3.524003	-0.578471
C	0.240865	4.202801	-0.197111
O	-1.784363	-1.086912	-3.060525
C	-2.457858	0.012398	-3.700178
O	-1.345744	-2.801392	-1.319300
C	0.089173	-2.812412	-1.401251
H	4.347312	-5.154816	0.727553
H	2.162622	0.734069	-2.510878
H	-0.143245	1.543697	-2.273560
H	-0.093117	0.436013	1.893257
H	2.188980	-0.426753	1.639490
H	4.946035	-0.436412	2.159244
H	5.333061	-2.270559	3.764500
H	5.036178	-4.633583	3.058868
H	3.950882	-3.312806	-0.888269
H	6.639234	0.247943	-1.735185
H	8.211803	2.112250	-1.279330
H	7.597172	3.883582	0.352209
H	5.405473	3.789932	1.518009
H	3.835066	1.938951	1.063355
H	-6.716740	0.771528	-0.067364
H	-5.123788	0.843420	-0.881205
H	-5.847580	-0.737241	-0.457049
H	-3.578650	-3.658482	2.300205
H	-2.444605	-2.944490	1.127539
H	-2.443510	-2.395164	2.831825
H	-3.856173	0.409762	4.914802
H	-5.208392	0.798404	3.815818
H	-4.689684	-0.896449	4.028800
H	-5.046915	-3.234501	-2.598783
H	-3.567010	-2.919245	-3.545815
H	-3.522590	-4.066710	-2.180211
H	-1.877996	0.275830	-4.585626
H	-3.466885	-0.283212	-4.003156
H	-2.526699	0.885137	-3.041920
H	0.401152	-3.845672	-1.243294
H	0.422017	-2.477606	-2.386947
H	0.534047	-2.180362	-0.628809
H	-4.701791	3.469346	-0.950004
H	-3.532439	4.473253	-0.049570
H	-3.159320	3.954800	-1.714938
H	0.280309	5.125954	-0.779565
H	0.234799	4.445574	0.869136
H	1.122086	3.596056	-0.430905

Table S63. XYZ Coordinates of A_para_VI_P(O)Ph_2

79
scf done: -4639.696743

C	-4.484295	1.552980	-1.318890
C	-3.914568	1.737748	-0.049818
C	-3.694555	3.040442	0.421741
C	-4.029525	4.141931	-0.367863
C	-4.589685	3.950201	-1.632468
C	-4.818316	2.655450	-2.106050
P	-3.439836	0.352243	1.049528
C	-4.725815	-0.933324	0.849882
C	-4.710767	-1.865177	-0.199311
C	-5.736495	-2.804477	-0.320069
C	-6.780325	-2.824160	0.607798
C	-6.796434	-1.906170	1.660851
C	-5.773704	-0.965012	1.782261
C	-1.910120	-0.362077	0.384503
C	-1.406651	-1.576271	0.984726
C	-0.241206	-2.133571	0.554038
C	0.578977	-1.501528	-0.470589
C	0.063186	-0.278324	-1.094909
C	-1.206090	0.227658	-0.637000
Ni	1.638191	0.072176	0.010470
P	2.122818	2.093135	-0.411264
O	1.567217	3.157212	0.701437
C	1.885810	4.565144	0.699712
P	1.553519	-2.635418	-1.465473
O	2.459550	-1.630731	-2.354369
C	3.295484	-2.167402	-3.404415
O	-3.326704	0.810050	2.488796
P	2.924718	-0.531999	1.603126
O	2.343718	-1.807116	2.435315
C	2.960394	-2.264361	3.657329
O	3.060998	0.505262	2.869855
C	3.994566	1.599368	2.856984
O	4.524253	-0.854167	1.399574
C	4.941198	-1.613900	0.246253
O	0.595186	-3.232504	-2.646021
C	-0.232565	-4.378585	-2.370127
O	2.280857	-3.708135	-0.718959
O	1.728248	2.813583	-1.840974
C	0.357792	3.104117	-2.161207
O	3.738401	2.320726	-0.529972
C	4.402201	3.290114	-1.370489
H	0.311967	-0.070023	-2.133935
H	0.107446	-3.063819	0.990814
H	-1.615320	1.099209	-1.137658
H	-1.980187	-2.066722	1.765488
H	-3.271477	3.181938	1.411004
H	-3.857265	5.146826	0.005746

H	-4.852752	4.806613	-2.246116
H	-5.260669	2.503953	-3.085992
H	-4.676590	0.552442	-1.693475
H	-5.774701	-0.258405	2.606252
H	-7.602362	-1.925656	2.388310
H	-7.575817	-3.557383	0.514085
H	-5.716567	-3.523476	-1.133449
H	-3.893274	-1.868352	-0.914107
H	1.763277	4.996429	-0.296918
H	1.192682	5.044990	1.391281
H	2.910924	4.719291	1.047368
H	-0.098579	2.259464	-2.683567
H	-0.221920	3.341170	-1.265132
H	0.361815	3.972231	-2.822987
H	5.470571	3.147857	-1.203872
H	4.162030	3.115118	-2.420040
H	4.125139	4.311180	-1.099611
H	6.008859	-1.800127	0.370591
H	4.402223	-2.562415	0.182508
H	4.775770	-1.044263	-0.672797
H	2.487524	-3.215765	3.903809
H	4.033919	-2.413912	3.516318
H	2.792593	-1.545164	4.461493
H	3.758479	2.216945	3.724865
H	5.018232	1.226232	2.938910
H	3.898346	2.197547	1.948518
H	3.954442	-2.945028	-3.010225
H	2.677523	-2.575185	-4.207309
H	3.891186	-1.335203	-3.778321
H	-1.037713	-4.114378	-1.677066
H	-0.663219	-4.689422	-3.322667
H	0.361058	-5.193414	-1.948704

Table S64. XYZ Coordinates of A_para_I_Me

48

scf done: -5724.483846

C	-0.210432	0.240006	0.269519
C	0.055736	0.126378	1.690886
C	1.405882	-0.047817	2.148084
C	2.405699	-0.283401	1.244483
C	2.171925	-0.248233	-0.173322
C	0.908657	0.045522	-0.621335
Ni	-1.143586	-1.243642	1.199659
P	-1.348877	-2.622089	2.805605
O	-2.772425	-2.552416	3.595751
C	-3.177060	-3.564382	4.544870
Br	-1.085615	1.329621	2.844559
C	3.328435	-0.461710	-1.119617
P	-2.347753	-2.075482	-0.353262
O	-2.855614	-1.111965	-1.596241

C	-3.677988	0.017313	-1.255466
O	-3.727478	-2.790256	0.139741
C	-4.609983	-3.473037	-0.778064
O	-1.718388	-3.288482	-1.257206
C	-0.572817	-3.035870	-2.092702
O	-1.334059	-4.207271	2.370884
C	-0.141848	-4.733795	1.764243
O	-0.232391	-2.762241	4.017371
C	-0.130289	-1.711813	4.995251
H	-5.549556	-3.627792	-0.246640
H	3.413156	-0.475390	1.606048
H	1.619406	-0.027475	3.211471
H	-0.982833	0.918795	-0.084331
H	0.739518	0.183147	-1.687382
H	-3.687018	0.681608	-2.120513
H	-4.699797	-0.303461	-1.031459
H	-3.277936	0.559670	-0.392044
H	-0.273527	-3.998064	-2.510116
H	-0.833550	-2.350280	-2.903198
H	0.254463	-2.617331	-1.512166
H	-4.786806	-2.870615	-1.672271
H	-4.181999	-4.436146	-1.064660
H	0.892704	-1.719546	5.375592
H	-0.349816	-0.730901	4.566462
H	-0.822741	-1.901905	5.821295
H	-0.388683	-5.728793	1.391584
H	0.181638	-4.109135	0.924375
H	0.664954	-4.805911	2.498460
H	-3.983237	-3.130469	5.137441
H	-3.538550	-4.449420	4.016823
H	-2.347684	-3.843034	5.199297
H	4.153413	0.229104	-0.907899
H	3.736567	-1.476084	-1.029197
H	3.024731	-0.316237	-2.159944

Table S65. XYZ Coordinates of A_para_TS1_Me

48

scf done: -5724.479551

C	-0.117832	0.093137	0.100202
C	-0.006305	0.048395	1.510321
C	1.252889	-0.057612	2.058812
C	2.431812	-0.046028	1.256878
C	2.298001	0.148248	-0.106958
C	1.024529	0.290593	-0.726379
C	3.785961	-0.169660	1.912922
Ni	-0.542693	-1.135359	-1.209769
P	-0.244422	-1.674169	-3.263748
O	-1.614597	-1.909554	-4.113603
C	-1.600323	-2.373672	-5.481785
Br	-1.852588	0.995653	-0.769832

P	-1.275538	-2.861520	-0.218270
O	-0.176121	-4.028573	0.162300
C	0.885777	-3.714122	1.077210
O	-2.018764	-2.808777	1.263729
C	-3.193362	-1.986927	1.377581
O	-2.300460	-3.779376	-1.105621
C	-2.769852	-5.065497	-0.648611
O	0.546862	-3.068493	-3.608208
C	1.872050	-3.251712	-3.081400
O	0.648518	-0.688245	-4.251692
C	0.155278	0.644204	-4.478400
H	-2.591221	-2.169949	-5.889028
H	1.349861	-0.169369	3.136103
H	-0.892552	0.009098	2.133297
H	0.983395	0.823245	-1.667113
H	3.189079	0.281386	-0.716308
H	0.998174	1.248774	-4.816230
H	-0.621186	0.638662	-5.249466
H	-0.258787	1.082286	-3.564028
H	2.150028	-4.287695	-3.279083
H	2.580550	-2.582224	-3.577100
H	1.892358	-3.073613	-2.001353
H	-0.844931	-1.843810	-6.066982
H	-1.400062	-3.446939	-5.511977
H	-3.410631	-1.882055	2.441848
H	-3.030778	-0.996760	0.941049
H	-4.045550	-2.461572	0.880287
H	1.559532	-4.572410	1.084761
H	1.438076	-2.824417	0.756155
H	0.492348	-3.550113	2.084287
H	-3.615427	-5.332019	-1.284271
H	-1.981138	-5.814738	-0.748848
H	-3.094810	-5.015352	0.393565
H	3.933604	0.602117	2.677321
H	3.898862	-1.138144	2.414733
H	4.592351	-0.077282	1.180655

Table S66. XYZ Coordinates of A_para_IIa_Me

48

scf done: -5724.533299

C	-0.016404	0.081119	0.049589
C	0.004768	0.033909	1.447278
C	1.259999	-0.015264	2.074433
C	2.440012	-0.028868	1.329831
C	2.426371	0.004118	-0.075552
C	1.170061	0.072125	-0.695117
C	-1.271734	0.019074	2.256161
Ni	4.010297	-0.240732	-1.104439
Br	3.973178	-2.504095	-0.495447
P	5.760572	-0.703847	-2.437885

O	5.399727	-1.943452	-3.425037
C	6.334876	-2.540948	-4.349411
P	3.975282	1.850056	-1.352100
O	2.942585	2.289731	-2.517448
C	2.889943	3.643766	-3.036181
O	7.071495	-1.053624	-1.553332
C	8.438485	-1.094686	-2.028643
O	6.438703	0.372269	-3.469240
C	5.644824	0.882795	-4.557746
O	3.695427	2.829360	-0.083662
C	2.386929	3.187053	0.411047
O	5.338765	2.575931	-1.874537
C	6.493351	2.627048	-1.014510
H	1.312441	-0.046976	3.161312
H	3.387987	-0.075035	1.861430
H	1.098069	0.125730	-1.779447
H	-0.972077	0.130005	-0.468869
H	2.413558	4.258018	0.622966
H	1.613483	2.976427	-0.328720
H	2.176882	2.626243	1.321274
H	9.067506	-1.041503	-1.139959
H	8.628069	-2.034131	-2.552488
H	8.646081	-0.249091	-2.684684
H	6.211660	1.703399	-4.997359
H	5.477637	0.108138	-5.310637
H	4.683444	1.261190	-4.201933
H	5.744433	-2.976446	-5.156204
H	7.022269	-1.798020	-4.760454
H	6.896417	-3.329431	-3.842006
H	1.982614	3.700695	-3.636726
H	2.843106	4.371932	-2.223935
H	3.766506	3.837995	-3.655832
H	7.293204	3.083683	-1.597119
H	6.283549	3.233645	-0.130953
H	6.801123	1.621765	-0.710510
H	-2.128712	0.337866	1.655748
H	-1.494010	-0.986293	2.635646
H	-1.203881	0.679601	3.127141

Table S67. XYZ Coordinates of A_para_IIb_Me

48

scf done: -5724.542306

C	0.016756	-0.022475	0.009577
C	0.023193	-0.014220	1.413954
C	1.282984	0.006087	2.035630
C	2.468718	0.001213	1.296903
C	2.451795	-0.016749	-0.104319
C	1.200736	-0.020622	-0.733615
Ni	-1.589274	-0.002896	2.392524
Br	-3.671742	0.075259	3.491788

C	3.733488	-0.051210	-0.904317
P	-1.477239	2.170696	2.577949
O	-0.134398	2.861752	1.978646
C	0.060851	4.292800	2.037993
O	-2.692281	3.031360	1.915911
C	-3.040111	2.746897	0.545191
O	-1.622830	2.827296	4.063165
C	-0.750005	2.322130	5.094963
P	-1.717737	-2.137926	1.980246
O	-0.385919	-3.052103	1.764942
C	0.469759	-3.257410	2.907141
O	-2.355492	-2.502573	0.525396
C	-3.606232	-1.899940	0.129754
O	-2.576922	-2.987345	3.068424
C	-2.886774	-4.384860	2.861160
H	-3.410324	1.722235	0.445265
H	3.423618	0.020582	1.818946
H	1.354302	0.049304	3.121017
H	-0.928844	-0.027202	-0.529179
H	1.149567	-0.022169	-1.820839
H	-3.832216	3.446318	0.278398
H	-2.182000	2.895297	-0.116603
H	1.021609	4.489702	1.562451
H	-0.734042	4.812507	1.498270
H	0.082821	4.635657	3.074906
H	-0.926577	2.939160	5.975945
H	-0.988206	1.279782	5.327264
H	0.299226	2.405260	4.796656
H	-3.345860	-4.735911	3.785233
H	-3.589134	-4.499191	2.032357
H	-1.982620	-4.961752	2.654031
H	-3.822682	-2.279541	-0.868735
H	-4.409668	-2.182992	0.814730
H	-3.524701	-0.810069	0.098786
H	1.290509	-3.888059	2.565380
H	0.871560	-2.307292	3.266436
H	-0.068143	-3.763426	3.713271
H	3.606727	0.419308	-1.884211
H	4.545862	0.464288	-0.382597
H	4.067930	-1.081417	-1.082226

Table S68. XYZ Coordinates of A_para_III_Me

48

scf done: -5724.478279

C	-0.161640	-0.538315	0.200985
C	-0.124104	0.013713	1.487693
C	1.099820	0.493420	1.973733
C	2.253766	0.421451	1.187858
C	2.222433	-0.117661	-0.105858
C	0.995388	-0.596894	-0.581832

Ni	-1.649697	0.116274	2.512448
P	-1.843911	2.284540	2.198252
O	-0.666103	3.118794	2.915478
C	-0.676810	4.573491	3.018879
C	3.463849	-0.153805	-0.965759
P	-1.733743	-2.043762	2.865825
O	-0.472805	-2.882515	3.443938
C	0.752258	-3.062458	2.690939
O	-2.847160	-2.438843	3.978369
C	-2.585371	-2.192679	5.381009
O	-2.268253	-2.822351	1.564630
C	-2.624656	-4.231706	1.589428
O	-3.248418	2.872134	2.775715
C	-3.612807	2.565939	4.142596
O	-2.041871	2.864405	0.709736
C	-0.935672	2.915236	-0.236183
H	-3.515949	-2.403285	5.906531
H	3.192286	0.797521	1.588736
H	1.164909	0.934785	2.963939
H	-1.089545	-0.932689	-0.203238
H	0.939306	-1.027462	-1.579002
H	0.202699	4.831362	3.607896
H	-0.620014	5.039996	2.030957
H	-1.579666	4.902295	3.537478
H	-4.455015	3.213475	4.384506
H	-3.921602	1.520746	4.240690
H	-2.785907	2.776440	4.826208
H	-1.199987	2.291590	-1.090977
H	-0.815738	3.961129	-0.528296
H	-0.011455	2.546354	0.210841
H	1.292437	-3.869715	3.185091
H	0.532802	-3.343977	1.658931
H	1.345160	-2.147604	2.707098
H	-1.795022	-2.855561	5.736952
H	-2.300790	-1.149939	5.548838
H	-2.732905	-4.532288	0.548332
H	-1.838151	-4.818240	2.067905
H	-3.567279	-4.362814	2.122382
H	3.415181	-0.958844	-1.704612
H	3.590865	0.786221	-1.517065
H	4.365027	-0.297555	-0.362214
Br	-0.519626	6.731058	-0.269123

Table S69. XYZ Coordinates of A_para_IV_Me

64

scf done: -6411.316383

C	1.243800	2.264042	-1.550285
C	1.415433	3.625062	-1.817439
C	1.153989	4.594822	-0.840139
C	0.720363	4.147180	0.413678

C	0.555196	2.784433	0.681637
C	0.814826	1.807712	-0.289122
C	1.310005	6.067199	-1.139437
Ni	0.711947	-0.103521	-0.139039
P	0.939156	-2.265905	-0.522986
O	0.088304	-2.921913	-1.743369
C	-0.942531	-3.918896	-1.582921
P	-1.306026	0.114793	-0.901601
O	-2.237434	1.310043	-0.334155
C	-2.525080	1.387308	1.085680
P	2.472884	0.073096	1.127014
O	3.775856	0.902409	0.622499
C	4.414492	0.511028	-0.616017
O	-2.166130	-1.235409	-0.653262
C	-3.539379	-1.375790	-1.113995
O	-1.402261	0.386674	-2.492329
C	-1.945819	1.581037	-3.105598
O	3.053469	-1.365258	1.611761
C	4.074693	-1.467642	2.631984
O	2.143122	0.832836	2.513200
C	2.905648	1.927197	3.076441
O	0.685274	-3.216239	0.761299
C	1.403039	-4.441826	1.033346
O	2.451294	-2.491771	-1.065715
C	2.865434	-3.534652	-1.982200
Br	-6.131124	-0.067031	1.508575
H	0.510660	4.872625	1.196835
H	0.229328	2.490008	1.674502
H	1.447023	1.556872	-2.352719
H	1.755751	3.936046	-2.802894
H	4.347920	-2.520860	2.688528
H	4.950857	-0.873634	2.361277
H	3.678317	-1.135381	3.594049
H	5.390701	0.995233	-0.623045
H	4.537379	-0.573219	-0.663748
H	3.823996	0.859099	-1.466776
H	2.419653	2.167008	4.021984
H	3.940427	1.630828	3.255754
H	2.872869	2.790825	2.410783
H	3.833572	-3.222234	-2.372694
H	2.973011	-4.484359	-1.453347
H	2.147022	-3.639263	-2.795186
H	1.069540	-4.778628	2.014357
H	1.169382	-5.206337	0.288093
H	2.476506	-4.245374	1.055051
H	-1.634372	-3.787881	-2.414833
H	-0.497602	-4.916349	-1.631048
H	-1.472494	-3.790705	-0.639307
H	-3.724911	-2.447985	-1.180809
H	-4.230852	-0.922337	-0.398430

H	-3.653734	-0.927511	-2.103404
H	-1.919719	1.398563	-4.179845
H	-2.972498	1.749665	-2.779480
H	-1.325747	2.444761	-2.859590
H	-2.475824	2.441478	1.359942
H	-3.527505	0.990897	1.270256
H	-1.788430	0.832891	1.675025
H	1.461760	6.647395	-0.224739
H	0.417696	6.469619	-1.635625
H	2.158309	6.252900	-1.805453

Table S70. XYZ Coordinates of A_para_TS2_Me

64

scf done: -6411.237551

Br	4.865604	-0.665939	-0.975194
C	3.115348	-2.430626	-2.059758
O	1.848787	-1.039361	-0.907349
P	0.838580	-1.444296	0.185385
O	1.558330	-1.758447	1.629971
C	2.738500	-2.592657	1.682860
Ni	-0.600631	0.199296	0.229480
P	0.797717	1.705647	1.068485
O	-0.146278	2.732238	1.895951
C	0.405473	3.789861	2.714007
C	-2.052517	-1.032749	0.126729
C	-2.555586	-1.740657	-0.973386
C	-3.645668	-2.607969	-0.853254
C	-4.286591	-2.805832	0.376873
C	-3.787801	-2.105919	1.484505
C	-2.697600	-1.239877	1.361210
C	-5.484173	-3.718845	0.501181
P	-1.957194	1.633590	-0.659132
O	-2.550191	1.235467	-2.128442
C	-1.619054	0.980154	-3.199899
O	-1.270488	3.083715	-0.913760
C	-2.011255	4.190742	-1.479938
O	-3.324941	2.028747	0.110658
C	-4.614229	1.421827	-0.140334
O	0.387426	-2.962951	-0.291448
C	-0.303615	-3.859564	0.593099
O	1.759845	2.661771	0.173666
C	2.090444	2.364358	-1.204982
O	1.784729	1.256536	2.282012
C	3.185500	0.977287	2.067034
H	-4.001992	-3.141296	-1.732389
H	-2.091483	-1.634483	-1.949863
H	-2.352574	-0.721939	2.255085
H	-4.254995	-2.241861	2.457823
H	3.357928	0.399022	1.157537
H	3.742535	1.916250	2.016093

H	3.516135	0.401587	2.932193
H	-0.444740	4.291373	3.175858
H	1.059929	3.375885	3.482967
H	0.959062	4.497267	2.091896
H	1.185392	2.161727	-1.781435
H	2.570120	3.263045	-1.594558
H	2.774487	1.514699	-1.258327
H	2.500299	-3.623177	1.402150
H	3.513286	-2.196479	1.020513
H	3.085366	-2.573066	2.717298
H	-0.082834	-4.873967	0.252667
H	0.043615	-3.745607	1.623458
H	-1.380067	-3.686129	0.548343
H	-4.609201	0.376738	0.172205
H	-5.329129	1.985631	0.459451
H	-4.871849	1.501231	-1.197136
H	-0.863027	0.246030	-2.903851
H	-2.202187	0.581359	-4.029831
H	-1.126330	1.905988	-3.508599
H	-1.282778	4.977453	-1.674583
H	-2.498085	3.896382	-2.412534
H	-2.760329	4.541152	-0.767220
H	3.073371	-1.793203	-2.929285
H	4.057764	-2.907925	-1.847372
H	2.221230	-2.961312	-1.778680
H	-5.531291	-4.185466	1.489932
H	-6.422962	-3.168300	0.359716
H	-5.460502	-4.514067	-0.249951

Table S71. XYZ Coordinates of A_para_V_Me

59

scf done: -3799.683050

C	1.782982	-0.053646	-0.203920
C	2.154460	-0.193324	-1.557150
C	3.487814	-0.126239	-1.967073
C	4.520896	0.083171	-1.041458
C	4.160798	0.213614	0.304669
C	2.823616	0.147536	0.711269
C	5.961959	0.177349	-1.486964
Ni	-0.112546	-0.114789	0.002610
P	0.032436	-2.197605	0.669362
O	1.061145	-2.135513	1.965332
C	1.073042	-3.246090	2.874532
P	-2.218728	-0.274831	-0.615018
O	-2.415779	-1.614151	-1.515483
C	-3.623818	-1.836554	-2.274010
P	-0.071241	2.028854	0.319599
O	0.520710	3.052693	-0.792049
C	1.923235	3.383645	-0.914767
O	-2.629775	0.868213	-1.706478

C	-3.748342	1.767166	-1.580153
O	-3.495758	-0.216235	0.389878
C	-3.324934	-0.439286	1.812000
O	0.778160	2.516155	1.630446
C	0.489083	1.907496	2.906498
O	-1.549505	2.664571	0.573170
C	-1.729308	4.069950	0.873993
O	0.927354	-3.236907	-0.277450
C	0.261917	-3.864683	-1.379505
O	-1.261886	-2.873765	1.082949
H	4.937541	0.367239	1.051561
H	2.605031	0.238077	1.770424
H	1.396202	-0.367309	-2.320343
H	3.729640	-0.244274	-3.021769
H	-3.512855	-2.810379	-2.750935
H	-4.494247	-1.849071	-1.612852
H	-3.744701	-1.062615	-3.035509
H	-3.620587	2.518219	-2.360836
H	-4.690570	1.235585	-1.735610
H	-3.750703	2.248162	-0.602196
H	-2.772339	0.393977	2.254787
H	-4.330345	-0.472704	2.232739
H	-2.799957	-1.380020	1.986654
H	0.985172	-4.533688	-1.851187
H	-0.602107	-4.444003	-1.040500
H	-0.075339	-3.127952	-2.117373
H	1.716626	-2.965278	3.710828
H	0.066332	-3.465175	3.240689
H	1.480312	-4.140947	2.391714
H	1.979799	4.167782	-1.670491
H	2.308875	3.757466	0.035014
H	2.494819	2.512612	-1.237794
H	0.599426	0.819773	2.860660
H	1.210953	2.315644	3.614130
H	-0.524318	2.159404	3.231603
H	-2.783064	4.197789	1.120667
H	-1.110408	4.364463	1.724272
H	-1.472113	4.675046	0.002588
H	6.194423	-0.567768	-2.255073
H	6.185905	1.161042	-1.918894
H	6.649153	0.025619	-0.649470

Table S72. XYZ Coordinates of A_para_TS3_Me

59

scf done: -3799.6511847

C	3.455310	1.307800	-0.551868
C	2.747287	0.405547	0.222161
C	1.697968	-0.354823	-0.349573
C	1.502393	-0.279229	-1.750352
C	2.200083	0.661032	-2.511832

C	3.180781	1.465136	-1.931862
Ni	-0.233447	-0.134219	0.155132
O	0.124628	-1.951600	1.603549
C	0.301199	-2.403980	2.974327
P	1.211389	-2.063652	0.396663
O	1.002236	-3.143362	-0.623292
C	3.981277	2.432917	-2.765233
P	-2.040897	-0.671920	-0.775825
O	-2.396868	-2.045604	-1.645596
C	-2.296364	-3.309710	-0.954390
P	-0.373834	1.777495	1.012892
O	0.645817	2.307714	2.212133
C	0.678124	1.502362	3.400348
O	-3.315375	-0.605778	0.255768
C	-4.676519	-0.704280	-0.206928
O	-2.588999	0.394273	-1.918665
C	-2.007162	0.380199	-3.228385
O	2.518625	-2.363765	1.346661
C	3.731676	-2.849605	0.746560
O	-0.036686	3.092782	0.075156
C	-0.650672	3.149620	-1.225326
O	-1.846313	2.148267	1.663488
C	-2.117701	3.441487	2.236849
H	-0.623288	-2.139880	3.486909
H	4.233984	1.909188	-0.092606
H	2.971981	0.304193	1.279786
H	0.841788	-0.984530	-2.240259
H	2.009354	0.734217	-3.578928
H	-5.291966	-0.903672	0.671963
H	-4.988039	0.236169	-0.669858
H	-4.792624	-1.517376	-0.927846
H	-2.454598	1.207980	-3.783013
H	-0.924024	0.530813	-3.186559
H	-2.224755	-0.561692	-3.738213
H	-1.284564	-3.464299	-0.575399
H	-3.014197	-3.352574	-0.129930
H	-2.536758	-4.084831	-1.683712
H	-3.173524	3.445509	2.510876
H	-1.506442	3.610775	3.127497
H	-1.922421	4.235951	1.511364
H	1.398261	1.961247	4.080372
H	-0.303313	1.468474	3.884712
H	0.998902	0.476675	3.182403
H	-1.691046	2.817227	-1.192577
H	-0.605826	4.191854	-1.550809
H	-0.095649	2.527120	-1.933965
H	0.448213	-3.484121	2.998277
H	1.153507	-1.904451	3.436528
H	4.405455	-3.114584	1.564211
H	3.537784	-3.727474	0.127198

H	4.199483	-2.067994	0.134388
H	4.123727	3.383913	-2.242669
H	4.979313	2.030474	-2.976967
H	3.495337	2.634738	-3.723405

Table S73. XYZ Coordinates of A_para_VI_Me

59

scf done: -3799.684279

C	1.721999	-0.390555	0.511618
C	2.335655	0.033923	1.759773
C	2.110951	-0.632461	2.926391
C	1.307392	-1.828717	2.964227
C	0.779741	-2.303232	1.792993
C	0.930441	-1.618734	0.526005
C	1.123841	-2.551435	4.275483
Ni	-0.202139	-0.052468	0.206534
P	-0.542321	2.035734	0.437370
O	-0.757424	3.066177	-0.828691
C	0.126789	2.962577	-1.963397
P	2.631906	0.074067	-0.962850
O	3.148487	1.474988	-0.996878
P	-2.018704	-0.920032	-0.423425
O	-2.736454	-0.059447	-1.618650
C	-3.546918	-0.595615	-2.686243
O	-3.134895	-1.099728	0.765519
C	-4.492742	-1.531162	0.538071
O	-2.072837	-2.382002	-1.195583
C	-1.810425	-3.595694	-0.472306
O	1.706207	-0.192663	-2.274573
C	1.469439	-1.474760	-2.891706
O	3.794389	-1.066139	-1.044302
C	4.893747	-0.883710	-1.962923
O	0.566298	2.772248	1.383215
C	0.416047	4.141709	1.810538
O	-1.873655	2.469528	1.303291
C	-3.175006	2.534190	0.696132
H	0.926315	-2.261753	-0.351277
H	2.972756	0.912754	1.745971
H	0.236048	-3.245310	1.802041
H	2.570078	-0.279463	3.846591
H	-4.524656	-2.416669	-0.101924
H	-4.913836	-1.774291	1.514276
H	-5.074006	-0.724469	0.083048
H	-0.743967	-3.833317	-0.495233
H	-2.147625	-3.523285	0.565034
H	-2.364021	-4.392622	-0.972673
H	-3.837257	0.258544	-3.299390
H	-2.973778	-1.305195	-3.284626
H	-4.443502	-1.086769	-2.301714
H	-0.144293	3.774596	-2.639846

H	1.171572	3.063415	-1.659487
H	-0.001209	2.003397	-2.472943
H	1.362435	4.427488	2.271639
H	0.212850	4.793945	0.957202
H	-0.393551	4.228736	2.538197
H	-3.889838	2.675178	1.508472
H	-3.231987	3.379736	0.006164
H	-3.413616	1.613042	0.159780
H	1.541778	-1.331709	-3.971625
H	2.208349	-2.212356	-2.572535
H	0.463693	-1.818852	-2.637923
H	5.602284	-1.687578	-1.761721
H	4.551151	-0.951826	-2.999817
H	5.372513	0.084344	-1.799721
H	0.553688	-3.475727	4.148168
H	2.089536	-2.807154	4.728519
H	0.592129	-1.925903	5.003286

Table S74. XYZ Coordinates of A_para_I_OMe

49

scf done: -5799.689157

C	-0.142077	0.207149	0.283626
C	0.141588	0.158213	1.702562
C	1.533391	0.123236	2.103701
C	2.564584	0.265560	1.114121
C	2.243298	0.252842	-0.212734
C	0.873876	0.211337	-0.638644
Ni	0.717016	-1.484969	2.662983
P	-1.115119	-2.546343	2.888384
O	-1.551350	-3.615883	1.725633
C	-1.816094	-3.144875	0.391242
Br	1.912679	1.032243	3.862911
O	0.716304	0.248243	-2.000160
C	-0.611247	0.283157	-2.509511
P	2.200326	-2.878732	3.281822
O	3.789436	-2.759953	2.836841
C	4.589045	-1.707016	3.403838
O	2.270311	-3.140370	4.889221
C	3.047600	-4.215650	5.461609
O	1.982484	-4.417156	2.744716
C	2.018167	-4.649714	1.326733
O	-2.539041	-1.709278	2.964572
C	-2.688526	-0.771893	4.044499
O	-1.197383	-3.518453	4.195206
C	-2.340919	-4.362656	4.453074
H	-2.249269	-4.701345	5.485599
H	3.015491	0.297426	-0.974366
H	3.600943	0.346066	1.423345
H	-0.588510	0.653962	2.337404
H	-1.177573	0.290937	-0.025459

H	-3.512837	-0.107035	3.783118
H	-2.922822	-1.293423	4.977420
H	-1.780596	-0.176337	4.189082
H	-1.969351	-4.029759	-0.227745
H	-2.717116	-2.525935	0.374642
H	-0.971262	-2.569535	0.001260
H	-3.273606	-3.807305	4.328402
H	-2.334074	-5.221383	3.778190
H	5.382539	-1.479557	2.689749
H	4.002185	-0.804522	3.592828
H	5.036949	-2.042552	4.344463
H	1.655631	-5.665209	1.161906
H	1.365511	-3.947572	0.796326
H	3.039000	-4.557151	0.946474
H	3.173292	-3.980176	6.519047
H	2.514072	-5.162411	5.352658
H	4.026573	-4.291020	4.982235
H	-0.520613	0.287683	-3.596367
H	-1.184756	-0.597981	-2.196282
H	-1.142176	1.186697	-2.185771

Table S75. XYZ Coordinates of A_para_TS1_OMe

49

scf done: -5799.684692

C	-0.021069	-0.004175	-0.036182
C	-0.017078	0.001522	1.384264
C	1.197102	0.010109	2.113952
C	2.368257	-0.235888	1.438341
C	2.376813	-0.421822	0.026073
C	1.207006	-0.285543	-0.701857
Ni	-1.387016	-1.221860	1.170730
P	-1.070809	-2.781404	2.572077
O	-2.421330	-3.596076	3.014036
C	-2.366176	-4.780840	3.836313
Br	-1.686184	1.086461	2.157456
O	3.612458	-0.665952	-0.505720
P	-2.919147	-1.869821	-0.175206
O	-3.178892	-1.046322	-1.590888
C	-3.588574	0.328991	-1.489022
O	-4.409978	-1.955954	0.478996
C	-5.541522	-2.472290	-0.255715
O	-2.814531	-3.367363	-0.836728
C	-1.626645	-3.707789	-1.571216
O	-0.170411	-4.069199	2.073226
C	1.196896	-3.854617	1.690761
O	-0.290519	-2.555715	4.019785
C	-0.840362	-1.570477	4.911061
H	-6.432642	-2.178740	0.300145
H	3.310549	-0.312485	1.971776
H	1.192968	0.123886	3.191756

H	-0.799524	0.468880	-0.618869
H	1.219327	-0.295516	-1.785350
H	-3.353922	0.809083	-2.440205
H	-4.665152	0.395044	-1.304002
H	-3.060024	0.848479	-0.682659
H	-1.676357	-4.778285	-1.774460
H	-1.585789	-3.158209	-2.515914
H	-0.727766	-3.494463	-0.983558
H	-5.579578	-2.050487	-1.263018
H	-5.484458	-3.561285	-0.317572
H	-0.096090	-1.388036	5.688060
H	-1.054566	-0.633968	4.386928
H	-1.761631	-1.939745	5.373562
H	1.565895	-4.801658	1.293718
H	1.277139	-3.083768	0.916619
H	1.799782	-3.561604	2.554893
H	-3.386895	-4.977678	4.167251
H	-1.994985	-5.629004	3.256334
H	-1.721873	-4.623821	4.704997
C	3.706588	-0.834499	-1.916870
H	4.752644	-1.060644	-2.125320
H	3.078186	-1.663937	-2.262746
H	3.420140	0.079737	-2.450226

Table S76. XYZ Coordinates of A_para_IIa_OMe

49

scf done: -5799.739101

C	-0.052640	0.050186	-0.027629
C	-0.005121	-0.033816	1.366119
C	1.241730	-0.115203	2.006110
C	2.413822	-0.118458	1.255798
C	2.399740	-0.044398	-0.150384
C	1.144437	0.050234	-0.761502
O	-1.101452	-0.040773	2.189689
C	-2.388253	0.035270	1.586539
Ni	3.993927	-0.279235	-1.169342
Br	3.925044	-2.560434	-0.629770
P	5.785303	-0.707375	-2.455677
O	5.476962	-1.938505	-3.470823
C	6.448803	-2.506016	-4.375989
P	3.949386	1.815342	-1.381009
O	2.923868	2.261246	-2.550529
C	2.870123	3.619340	-3.058132
O	7.069591	-1.050045	-1.530150
C	8.450966	-1.083013	-1.962833
O	6.482601	0.390691	-3.450452
C	5.715830	0.905477	-4.556310
O	3.652322	2.776110	-0.102148
C	2.338726	3.121544	0.386818
O	5.310479	2.561512	-1.882161

C	6.454647	2.616426	-1.008848
H	1.268066	-0.180522	3.090265
H	3.360673	-0.191647	1.785828
H	1.070064	0.130874	-1.843742
H	-0.998572	0.119002	-0.552710
H	2.373741	4.179848	0.653586
H	1.577742	2.959627	-0.377807
H	2.103763	2.518354	1.263296
H	9.052270	-1.015082	-1.056153
H	8.664046	-2.026167	-2.470679
H	8.671135	-0.242176	-2.620916
H	6.286588	1.736789	-4.969871
H	5.578660	0.137709	-5.322200
H	4.740704	1.270420	-4.224514
H	5.890435	-2.929639	-5.211484
H	7.141770	-1.747684	-4.747510
H	7.001397	-3.299873	-3.867129
H	1.961366	3.681300	-3.656086
H	2.825658	4.341366	-2.240289
H	3.745214	3.818225	-3.678389
H	7.258644	3.079521	-1.580556
H	6.230776	3.218739	-0.125813
H	6.764997	1.612011	-0.704584
H	-3.108316	0.011742	2.405508
H	-2.515161	0.966519	1.021130
H	-2.572410	-0.815335	0.919209

Table S77. XYZ Coordinates of A_para_IIb_OMe

49

scf done: -5799.747989

H	-0.528893	1.293480	0.276272
C	-0.282893	1.250059	1.341166
O	1.023120	0.669648	1.535673
P	2.309188	1.511120	0.993724
O	3.403997	0.361741	1.367170
C	4.777325	0.620958	1.009956
Ni	2.224025	2.233355	-1.066691
P	1.923308	3.073180	-3.052789
O	0.400248	3.580750	-3.337895
C	-0.696808	2.680095	-3.074254
C	2.375700	4.014591	-0.458641
C	3.614950	4.596905	-0.159038
C	3.739506	5.913873	0.302787
C	2.591553	6.691568	0.486248
C	1.337159	6.134050	0.205616
C	1.241858	4.823562	-0.258274
O	2.587000	7.988290	0.933714
C	3.839122	8.594501	1.233030
Br	1.929605	0.006827	-1.771005
O	2.538949	2.649292	2.130644

C	2.589642	2.304846	3.533549
O	2.286640	2.070154	-4.280221
C	2.037574	2.438328	-5.656486
O	2.611319	4.478660	-3.506204
C	4.048417	4.510011	-3.619411
H	4.724774	6.310950	0.519173
H	4.529013	4.015131	-0.262969
H	0.249310	4.430257	-0.467006
H	0.449500	6.742372	0.354568
H	-0.988976	0.594229	1.850359
H	-0.337368	2.252694	1.774987
H	2.783663	3.234071	4.069172
H	1.637286	1.880453	3.859374
H	3.392997	1.590362	3.726801
H	5.357104	-0.222299	1.385101
H	4.888722	0.684417	-0.076706
H	5.134937	1.544794	1.474063
H	2.236679	1.546807	-6.251128
H	0.999247	2.748969	-5.791201
H	2.702961	3.246728	-5.968299
H	-1.604862	3.223789	-3.334379
H	-0.617596	1.778982	-3.687997
H	-0.727839	2.397835	-2.018191
H	4.301560	5.481844	-4.043450
H	4.512979	4.411641	-2.635885
H	4.408756	3.718782	-4.282608
H	3.612882	9.608812	1.564423
H	4.364921	8.062141	2.035012
H	4.487919	8.639915	0.349848

Table S78. XYZ Coordinates of A_para_III_OMe

49

scf done: -5799.680579

C	0.058645	-0.704736	0.269497
C	0.022291	-0.082257	1.528157
C	1.225028	0.386398	2.065860
C	2.438652	0.241248	1.381194
C	2.456255	-0.372744	0.123478
C	1.255586	-0.841900	-0.429229
Ni	-1.555200	0.109204	2.466657
P	-1.673182	2.293455	2.169844
O	-0.372529	3.099493	2.682399
C	-0.274784	4.546615	2.591593
O	3.576299	-0.560583	-0.635690
C	4.824057	-0.109316	-0.115078
P	-1.777220	-2.042385	2.790972
O	-0.625892	-2.929923	3.508426
C	0.669023	-3.155534	2.899323
O	-3.015426	-2.353833	3.793744
C	-2.889511	-2.083263	5.210246

O	-2.219888	-2.803105	1.452930
C	-2.660592	-4.197302	1.444738
O	-2.952105	2.960075	2.921420
C	-3.010400	2.863447	4.365015
O	-2.049751	2.875257	0.708101
C	-1.182826	2.614716	-0.423062
H	-3.871786	-2.267221	5.643762
H	3.348842	0.614802	1.835206
H	1.241139	0.880920	3.032378
H	-0.849493	-1.097911	-0.177620
H	1.279438	-1.318749	-1.404314
H	0.503324	4.842367	3.293964
H	0.007665	4.835669	1.577704
H	-1.221327	5.017529	2.863497
H	-4.027497	3.129612	4.649177
H	-2.794241	1.846284	4.705659
H	-2.303415	3.560201	4.821268
H	-1.475458	3.325163	-1.195512
H	-0.134581	2.770044	-0.157361
H	-1.323166	1.593677	-0.780987
H	1.140311	-3.947097	3.481379
H	0.555735	-3.478778	1.862376
H	1.272626	-2.248108	2.940508
H	-2.151786	-2.751991	5.656839
H	-2.604426	-1.041573	5.387186
H	-2.700574	-4.498760	0.401286
H	-1.953831	-4.827023	1.984883
H	-3.648870	-4.274660	1.895009
H	5.571764	-0.358288	-0.868595
H	4.822774	0.974619	0.049090
H	5.074334	-0.615730	0.824468
Br	-3.798768	-7.487120	1.089163

Table S79. XYZ Coordinates of A_para_IV_OMe

65

scf done: -6486.525886

C	4.191849	0.853908	-1.102368
C	4.746398	1.332544	0.091328
C	3.945711	1.396613	1.240135
C	2.617735	0.974813	1.194450
C	2.036734	0.478398	0.016636
C	2.854187	0.441871	-1.124792
O	6.037126	1.759371	0.234916
C	6.888872	1.718713	-0.906826
Ni	0.267856	-0.211984	-0.260303
P	-0.291218	1.864566	-0.461253
O	-1.802306	2.057130	-0.982525
C	-2.485398	3.339378	-0.975354
P	1.021420	-1.970605	0.789683
O	-0.008612	-3.226367	0.835919

C	0.210426	-4.358175	1.709478
P	-1.508806	-1.224561	-1.106850
O	-2.540378	-1.751603	0.020439
C	-3.466118	-2.845539	-0.163264
O	1.267801	-1.612701	2.346524
C	2.458688	-1.929540	3.105753
O	2.452735	-2.638238	0.398283
C	2.620463	-3.198291	-0.924183
O	-0.891943	-2.477156	-1.934253
C	-1.532747	-3.171528	-3.031266
O	-2.426059	-0.573448	-2.273485
C	-3.711353	0.057793	-2.058079
O	0.524457	2.857129	-1.445815
C	1.602380	3.730940	-1.039876
O	-0.204674	2.693145	0.934684
C	-0.896080	2.193832	2.108054
Br	-4.738256	1.540104	1.475416
H	4.379560	1.779715	2.158986
H	2.031337	1.035086	2.106183
H	2.454697	0.095790	-2.076755
H	4.779245	0.802670	-2.011618
H	-0.566930	-5.084169	1.472795
H	1.193629	-4.800576	1.531381
H	0.122311	-4.049266	2.753590
H	3.536775	-3.787398	-0.893419
H	1.776053	-3.840929	-1.183956
H	2.722777	-2.398741	-1.661737
H	2.259638	-1.586505	4.120946
H	2.648205	-3.004197	3.108336
H	3.318678	-1.398683	2.694828
H	-0.733852	-3.668828	-3.581286
H	-2.230493	-3.918522	-2.645874
H	-2.055300	-2.469763	-3.681114
H	-4.158909	-2.807764	0.676902
H	-4.024659	-2.743180	-1.096906
H	-2.918952	-3.790724	-0.151042
H	-3.730972	0.955927	-2.675193
H	-4.490095	-0.633508	-2.392149
H	-3.870757	0.330442	-1.013186
H	-2.754021	3.581126	-2.005328
H	-3.375385	3.206693	-0.356886
H	-1.849673	4.126415	-0.566182
H	1.809387	4.366226	-1.901670
H	1.295827	4.345120	-0.192470
H	2.489513	3.150617	-0.785615
H	-0.755351	2.949866	2.880943
H	-1.963433	2.056591	1.906661
H	-0.452640	1.250391	2.440800
H	7.856785	2.094387	-0.573543
H	7.008091	0.696226	-1.283996

H	6.511316	2.357897	-1.713642

Table S80. XYZ Coordinates of A_para_TS2_OMe

65

scf done: -6486.443402

Br	4.878838	-1.223836	-0.981162
C	2.965026	-2.873434	-1.964899
O	1.838760	-1.284989	-0.927695
P	0.822965	-1.493999	0.213153
O	1.541352	-1.814666	1.657200
C	2.605691	-2.789739	1.736090
Ni	-0.389459	0.330208	0.208654
P	1.172867	1.652790	1.059815
O	0.363284	2.806705	1.862379
C	1.043645	3.814467	2.645396
C	-1.984022	-0.703078	-0.015793
C	-2.392627	-1.455970	-1.130961
C	-3.578883	-2.184521	-1.138100
C	-4.412589	-2.189309	-0.010208
C	-4.038692	-1.450173	1.116684
C	-2.840896	-0.719669	1.095797
O	-5.555504	-2.937490	-0.111565
C	-6.431350	-2.974222	1.010392
P	-1.566249	1.947078	-0.623814
O	-2.337918	1.666010	-2.034611
C	-1.542385	1.330391	-3.189662
O	-0.675720	3.267982	-0.958612
C	-1.277103	4.486895	-1.452498
O	-2.787561	2.509433	0.274752
C	-4.192134	2.385892	-0.048744
O	0.168390	-2.970639	-0.147444
C	-0.633013	-3.672039	0.815667
O	2.247082	2.459558	0.145767
C	2.516946	2.112302	-1.234784
O	2.098319	1.114994	2.284640
C	3.458663	0.673997	2.083151
H	-3.876626	-2.761501	-2.009003
H	-1.770677	-1.494433	-2.021018
H	-2.591093	-0.147986	1.987907
H	-4.658618	-1.430122	2.005559
H	3.575150	0.089978	1.168323
H	4.122815	1.541492	2.051930
H	3.707344	0.052524	2.943973
H	0.263101	4.418762	3.107206
H	1.661165	3.347791	3.414800
H	1.661918	4.440452	1.997441
H	1.586011	2.026905	-1.799149
H	3.105276	2.937781	-1.637382
H	3.085136	1.181085	-1.288722
H	2.242122	-3.788173	1.474334

H	3.426392	-2.508023	1.070720
H	2.947547	-2.792193	2.772465
H	-0.846225	-4.654614	0.390252
H	-0.095503	-3.798632	1.759671
H	-1.572366	-3.146861	0.999604
H	-4.499299	1.340194	0.010283
H	-4.721750	2.970276	0.703947
H	-4.397163	2.784805	-1.042658
H	-0.848729	0.513432	-2.969768
H	-2.238841	1.011262	-3.964727
H	-0.980128	2.201856	-3.534926
H	-0.454883	5.166022	-1.676711
H	-1.857652	4.296079	-2.358289
H	-1.921994	4.922224	-0.685950
H	2.986403	-2.296514	-2.876443
H	3.854730	-3.427356	-1.714582
H	2.022344	-3.292266	-1.655065
H	-7.264186	-3.616948	0.722648
H	-5.937776	-3.396500	1.893954
H	-6.813681	-1.976329	1.256777

Table S81. XYZ Coordinates of A_para_V_OMe

60

scf done: -3874.888218

C	3.878480	-0.367153	0.902558
C	2.500841	-0.422934	1.101667
C	1.582543	-0.139585	0.074827
C	2.135688	0.202869	-1.170793
C	3.518648	0.253806	-1.400584
C	4.400219	-0.029417	-0.353551
Ni	-0.334910	-0.162164	0.079003
P	-2.409353	-0.179178	-0.672141
O	-2.865878	1.177988	-1.461502
C	-3.968212	2.017122	-1.069674
O	5.768478	-0.004502	-0.452311
C	6.338390	0.328749	-1.712787
P	-0.202135	1.978951	0.390853
O	0.891958	2.556190	1.457356
C	0.788341	2.131562	2.831184
P	-0.295610	-2.333169	0.460610
O	0.909031	-3.197298	-0.301163
C	0.678534	-3.555237	-1.666608
O	-1.601802	2.603150	0.956032
C	-1.757936	4.022908	1.185894
O	0.174693	2.871078	-0.907756
C	1.296203	3.778970	-1.007571
O	-1.589617	-3.120455	0.367646
O	0.305710	-2.418217	2.001178
C	0.243231	-3.674865	2.694391
O	-3.732379	-0.441221	0.236670

C	-3.639517	-0.962826	1.584995
O	-2.470568	-1.232929	-1.906405
C	-3.627822	-1.318454	-2.766306
H	4.569423	-0.591852	1.710546
H	2.139633	-0.724238	2.079176
H	1.488046	0.448926	-2.010671
H	3.884506	0.518133	-2.386274
H	-3.412119	-2.101224	-3.493422
H	-4.513862	-1.589557	-2.186511
H	-3.793831	-0.368750	-3.279280
H	-3.851032	2.948846	-1.624850
H	-4.919112	1.549121	-1.335870
H	-3.944860	2.223238	0.000555
H	-3.110647	-0.254283	2.229136
H	-4.666978	-1.069299	1.934391
H	-3.128685	-1.926314	1.578137
H	1.499727	-4.206348	-1.974726
H	-0.270057	-4.088971	-1.782514
H	0.672440	-2.669449	-2.314413
H	0.510830	-3.479732	3.734736
H	-0.763989	-4.097699	2.645775
H	0.954090	-4.388306	2.265018
H	1.195523	4.269417	-1.976010
H	1.271838	4.522480	-0.209860
H	2.233452	3.220487	-0.969923
H	0.754306	1.041422	2.903912
H	1.681362	2.497642	3.337618
H	-0.101827	2.558475	3.300612
H	-2.734406	4.156171	1.651109
H	-0.977246	4.398309	1.851480
H	-1.723756	4.561268	0.235753
H	7.419933	0.288735	-1.576638
H	6.046119	-0.386955	-2.490851
H	6.052322	1.338483	-2.031878

Table S82. XYZ Coordinates of A_para_TS3_OMe

60

scf done: -3874.8575024

C	-2.386995	-1.093141	-1.841195
C	-3.398304	-1.402436	-0.925628
C	-3.527753	-0.651356	0.265073
C	-2.634019	0.360701	0.549263
C	-1.547575	0.638886	-0.325223
C	-1.502341	-0.046456	-1.559642
O	-4.326598	-2.372794	-1.101350
C	-4.257450	-3.185242	-2.275770
Ni	0.355727	0.259903	0.161545
P	1.932539	-0.137263	-1.144572
O	3.383461	0.452653	-0.600833
C	4.642266	-0.033783	-1.098211

P	-0.662878	2.367108	-0.317082
O	-1.768126	3.260746	0.504369
C	-2.967313	3.668615	-0.173330
O	0.528981	2.475532	0.789769
C	0.579556	3.403461	1.905834
P	0.356957	-0.996405	1.848175
O	-0.279359	-2.518390	1.745424
C	0.072058	-3.328958	0.608730
O	-0.374917	2.962095	-1.660763
O	1.875708	-1.257056	2.446654
C	2.115203	-2.140879	3.559319
O	-0.494270	-0.707010	3.244591
C	-0.263069	0.544819	3.900702
O	2.197218	-1.757940	-1.305452
C	2.588484	-2.425462	-2.520088
O	2.013471	0.205358	-2.768634
C	2.214995	1.573357	-3.170516
H	1.447905	3.104322	2.492280
H	-4.337487	-0.895652	0.944503
H	-2.740480	0.925641	1.468174
H	-0.788418	0.261304	-2.313520
H	-2.306733	-1.619160	-2.784294
H	5.424821	0.491136	-0.547980
H	4.737085	-1.109229	-0.923588
H	4.752884	0.173538	-2.167339
H	2.717401	-3.476569	-2.254781
H	1.813218	-2.331929	-3.283425
H	3.526611	-2.034899	-2.920148
H	1.429374	2.220569	-2.774656
H	3.191678	1.936805	-2.835172
H	2.187183	1.583079	-4.261747
H	1.671524	-1.744173	4.476408
H	1.703878	-3.134169	3.362050
H	3.197682	-2.209878	3.678021
H	-0.763364	0.492352	4.869198
H	0.805767	0.721671	4.061668
H	-0.684359	1.377862	3.329097
H	1.139344	-3.267778	0.384562
H	-0.192219	-4.355830	0.869041
H	-0.493286	-3.021020	-0.275676
H	0.708964	4.420638	1.532206
H	-0.327693	3.343992	2.505033
H	-3.458608	4.401576	0.467920
H	-2.725905	4.121649	-1.137760
H	-3.637331	2.815871	-0.326311
H	-5.065862	-3.909837	-2.182501
H	-4.405155	-2.587230	-3.181334
H	-3.299048	-3.711845	-2.337112

Table S83. XYZ Coordinates of A_para_VI_OMe

60

scf done: -3874.887498

C	-2.525791	0.539812	1.072799
C	-2.559274	1.808157	1.577128
C	-1.849409	2.861611	0.907505
C	-1.169636	2.606083	-0.257570
C	-1.054491	1.274239	-0.799582
C	-1.735430	0.192342	-0.090403
O	-1.859671	4.162408	1.343262
C	-2.620505	4.493308	2.501065
Ni	0.240296	0.082901	0.071113
P	2.048306	0.633439	-0.859113
O	2.959110	1.695892	0.000255
C	4.299704	2.083332	-0.364456
P	-2.318704	-1.253023	-0.976969
O	-1.214515	-1.687627	-2.088925
C	-1.014189	-1.050494	-3.367958
O	-3.566111	-0.664609	-1.845888
C	-4.465352	-1.588257	-2.497488
O	-2.668518	-2.432987	-0.130974
P	0.703920	-1.270658	1.645212
O	-0.484268	-1.413881	2.757928
C	-0.295710	-2.150114	3.983787
O	1.901066	-0.814383	2.680758
C	3.284979	-1.019215	2.350920
O	1.215449	-2.815090	1.387182
C	0.567118	-3.601030	0.366787
O	2.104857	1.269752	-2.387250
C	1.650170	2.611265	-2.628445
O	3.006781	-0.660070	-1.167442
C	3.914543	-0.801105	-2.280669
H	-0.944623	1.219556	-1.879721
H	-3.092051	-0.244843	1.564368
H	-0.721269	3.441725	-0.786288
H	-3.145041	2.015458	2.464141
H	4.357389	2.365775	-1.418881
H	4.559467	2.942353	0.255338
H	4.997616	1.266114	-0.162188
H	0.582790	2.619667	-2.863502
H	1.840368	3.258050	-1.767859
H	2.206854	2.988944	-3.488413
H	4.362800	-1.790304	-2.178037
H	3.376254	-0.733541	-3.227192
H	4.700603	-0.043064	-2.256590
H	1.025705	-4.590481	0.402813
H	-0.506652	-3.680017	0.552287
H	0.722632	-3.162224	-0.622981
H	-1.276705	-2.223444	4.455236
H	0.087434	-3.152939	3.777703

H	0.396141	-1.623268	4.644337
H	3.866436	-0.458007	3.084283
H	3.538694	-2.080282	2.415541
H	3.517706	-0.652474	1.348526
H	-0.895740	-1.843380	-4.108992
H	-1.866967	-0.424743	-3.638203
H	-0.103333	-0.447816	-3.332220
H	-5.285628	-0.991770	-2.897502
H	-3.961615	-2.110192	-3.316570
H	-4.849730	-2.317676	-1.781073
H	-2.484509	5.565264	2.650417
H	-3.687692	4.284470	2.361529
H	-2.262253	3.960658	3.389825

Table S84. XYZ Coordinates of A_meta_I_P(O)Ph$_2$

68

scf done: -6564.486480

C	-4.332766	-0.844362	0.724685
C	-3.655628	-0.880912	-0.503400
C	-3.535634	-2.106280	-1.182383
C	-4.069418	-3.272169	-0.635066
C	-4.735724	-3.226940	0.592944
C	-4.869055	-2.013258	1.269479
P	-3.064052	0.639788	-1.325592
C	-3.003224	1.960921	-0.063802
C	-2.171402	1.903451	1.063974
C	-2.145709	2.964201	1.969577
C	-2.949171	4.088128	1.757697
C	-3.780282	4.149728	0.637428
C	-3.807238	3.090699	-0.272128
C	-1.338987	0.310464	-1.853819
C	-0.456457	-0.633960	-1.206435
C	0.851201	-0.771820	-1.806184
C	1.168923	-0.203489	-3.074312
C	0.292252	0.689446	-3.634448
C	-0.953660	0.966048	-3.008242
Ni	1.206269	-0.199938	-0.064874
P	2.864513	1.127315	-0.187349
O	3.883365	1.184323	-1.490923
C	4.658377	0.005552	-1.773649
Br	1.748185	-2.537023	-1.370250
O	-3.935934	1.058909	-2.490435
P	0.895291	-0.701085	2.002519
O	0.038914	0.326263	2.933945
C	-0.127334	0.120568	4.356814
O	2.234834	-0.851947	2.935996
C	3.254652	-1.778624	2.526614
O	0.298674	-2.196303	2.375008
C	-0.963008	-2.638594	1.849543
O	2.451708	2.718092	-0.178851

C	1.656495	3.253959	-1.250751
O	3.898398	1.134066	1.076374
C	4.944293	2.121300	1.206198
H	-0.927892	0.789655	4.672981
H	-1.651276	1.641061	-3.492915
H	-0.923981	-1.469348	-0.702239
H	2.109390	-0.444736	-3.556313
H	0.541458	1.178945	-4.570839
H	-1.363831	-3.366495	2.557473
H	-1.673969	-1.813721	1.755146
H	-0.826154	-3.124070	0.879335
H	4.075157	-1.675573	3.237885
H	2.876379	-2.804500	2.549007
H	3.619168	-1.540877	1.522626
H	-0.405336	-0.914426	4.567565
H	0.796758	0.368857	4.882251
H	1.431341	4.287936	-0.985734
H	2.212464	3.229377	-2.191796
H	0.720520	2.698632	-1.366364
H	5.184190	0.188148	-2.711913
H	5.389060	-0.177437	-0.979326
H	4.019246	-0.875516	-1.882230
H	4.521483	3.082981	1.505137
H	5.619753	1.756787	1.981201
H	5.490278	2.239094	0.267040
H	-3.020980	-2.151171	-2.138020
H	-3.966138	-4.213884	-1.165216
H	-5.151311	-4.135153	1.018763
H	-5.389860	-1.973998	2.221259
H	-4.441715	0.093900	1.258191
H	-4.443019	3.132211	-1.150251
H	-4.405459	5.021541	0.469307
H	-2.926106	4.913294	2.463371
H	-1.494485	2.909927	2.836313
H	-1.542037	1.040807	1.248952

Table S85. XYZ Coordinates of A_meta_IIa_P(O)Ph$_2$

68

scf done: -6564.542336

C	2.345531	0.782599	-0.469048
C	2.614140	1.461670	-1.665079
C	1.555291	1.825561	-2.500108
C	0.241641	1.507253	-2.152185
C	-0.059820	0.820936	-0.962617
C	1.012302	0.485801	-0.125622
H	3.634148	1.702845	-1.945578
Ni	-1.799445	0.150079	-0.587989
Br	-1.411563	-1.496373	-2.207263
P	-3.742281	-0.870467	-0.106425

O	-3.489302	-2.449077	0.177586
C	-4.540586	-3.403156	0.446275
P	-2.018920	1.835693	0.659133
O	-1.298368	1.604873	2.087173
C	-1.481387	2.511356	3.206458
O	-4.796305	-0.677960	-1.319566
C	-6.223385	-0.917352	-1.258080
O	-4.712955	-0.442763	1.139510
C	-4.216890	-0.576766	2.486186
O	-1.558601	3.301943	0.127607
C	-0.217996	3.831512	0.219422
O	-3.520951	2.241273	1.139051
C	-4.466614	2.746710	0.175543
H	1.758573	2.350079	-3.429987
H	-0.560440	1.787736	-2.830627
H	0.817918	-0.031867	0.810265
P	3.670796	0.404177	0.722437
H	-0.304658	4.849905	0.603835
H	0.398809	3.235328	0.893354
H	0.233978	3.841448	-0.772046
H	-6.651183	-0.404961	-2.119748
H	-6.429713	-1.987275	-1.331382
H	-6.643794	-0.514414	-0.336486
H	-4.941097	-0.082532	3.133494
H	-4.134728	-1.630447	2.764851
H	-3.244409	-0.091386	2.599156
H	-4.094903	-4.202445	1.039170
H	-5.361413	-2.948111	1.005185
H	-4.911734	-3.811621	-0.496961
H	-0.715205	2.247835	3.934649
H	-1.354415	3.549495	2.893354
H	-2.475020	2.373312	3.634735
H	-5.411194	2.868713	0.704930
H	-4.131752	3.709246	-0.216562
H	-4.604991	2.037668	-0.646389
C	3.338152	-1.286869	1.338871
C	5.229936	0.301156	-0.232793
O	3.794310	1.398161	1.858566
C	3.705882	-1.575382	2.661439
C	3.519596	-2.856703	3.181961
C	2.963559	-3.860328	2.385745
C	2.589477	-3.579874	1.069159
C	2.774454	-2.299226	0.546101
H	4.126163	-0.787968	3.279033
H	3.804380	-3.069741	4.207915
H	2.816604	-4.856982	2.791023
H	2.148341	-4.355532	0.450611
H	2.460219	-2.088857	-0.471361
C	6.270934	1.167797	0.126422
C	7.490057	1.124283	-0.553508

C	7.676172	0.214272	-1.595269
C	6.642470	-0.654710	-1.957584
C	5.424692	-0.613596	-1.279673
H	6.116052	1.868384	0.940555
H	8.291729	1.799258	-0.269185
H	8.623981	0.179196	-2.124089
H	6.785660	-1.364382	-2.766751
H	4.631040	-1.295418	-1.569753

Table S86. XYZ Coordinates of A_meta_IIb_P(O)Ph$_2$

68
scf done: -6564.551698

C	0.650157	-0.010160	2.654818
C	0.537225	0.020997	1.253425
C	-0.716650	0.363535	0.724406
C	-1.819349	0.645656	1.547677
C	-1.675812	0.598421	2.940850
C	-0.434822	0.273307	3.488029
Ni	1.999925	-0.367779	0.127766
Br	3.796143	-0.787727	-1.329492
H	-2.522282	0.827914	3.580060
P	2.707687	1.690856	0.314351
O	1.834759	2.636632	1.305296
C	2.173754	4.028595	1.503596
O	4.247342	1.940110	0.783842
C	4.681784	1.292749	1.998168
O	2.828885	2.556522	-1.058537
C	1.693876	2.571963	-1.949190
P	1.073040	-2.314987	-0.196052
O	-0.134303	-2.290534	-1.294444
C	0.131763	-1.752366	-2.608780
O	0.177997	-3.031260	0.960016
C	0.857766	-3.464421	2.156517
O	2.082065	-3.489584	-0.682327
C	1.597995	-4.809643	-1.026804
H	4.642028	0.204425	1.892442
P	-3.473278	1.018193	0.876792
H	-0.839493	0.424206	-0.353925
H	1.608049	-0.240829	3.116475
H	-0.309914	0.246842	4.567317
H	5.713800	1.603761	2.159159
H	4.068981	1.605674	2.848447
H	1.462679	4.414368	2.233853
H	3.191339	4.126268	1.888249
H	2.082012	4.582295	0.566557
H	1.944366	3.266133	-2.751198
H	1.522514	1.576870	-2.369538
H	0.792376	2.916936	-1.435313
H	2.417631	-5.309104	-1.543138
H	1.333527	-5.368213	-0.126118

H	0.729588	-4.744894	-1.685563
H	0.117131	-3.995927	2.753820
H	1.684876	-4.138028	1.916913
H	1.229792	-2.606507	2.720902
H	-0.805995	-1.820464	-3.159242
H	0.447520	-0.707374	-2.547554
H	0.903584	-2.335460	-3.117543
O	-4.345035	1.768686	1.861414
C	-3.187929	1.939948	-0.677976
C	-4.232044	-0.581033	0.408421
C	-3.806298	1.581921	-1.884251
C	-3.635245	2.368225	-3.026119
C	-2.849269	3.519813	-2.972231
C	-2.232468	3.888260	-1.772608
C	-2.400092	3.103701	-0.632378
H	-4.420144	0.688928	-1.936515
H	-4.116352	2.079407	-3.955622
H	-2.716922	4.130162	-3.860479
H	-1.623008	4.785648	-1.726151
H	-1.913985	3.395278	0.294157
C	-5.594585	-0.755753	0.691626
C	-6.235299	-1.951721	0.362927
C	-5.518700	-2.981879	-0.249033
C	-4.159470	-2.816085	-0.528729
C	-3.514928	-1.622678	-0.201421
H	-6.141257	0.045582	1.178125
H	-7.290123	-2.079012	0.587121
H	-6.015591	-3.913472	-0.503162
H	-3.597368	-3.618336	-0.997269
H	-2.455821	-1.518430	-0.414524

Table S87. XYZ Coordinates of A_meta_III_P(O)Ph_2

68

scf done: -6564.483865

C	0.783822	-0.295706	-2.981682
C	-0.165994	-1.059491	-2.295623
C	-0.286465	-0.964583	-0.902267
C	0.571653	-0.100257	-0.209235
C	1.535479	0.661102	-0.889979
C	1.632757	0.564021	-2.287432
Ni	-1.560181	-1.935749	0.009202
P	-0.536259	-3.844969	-0.333382
O	-0.644787	-4.295056	-1.871241
C	-0.237427	-5.613712	-2.330087
H	2.367518	1.151242	-2.829232
P	-2.816803	-0.156394	0.389075
O	-3.595523	0.519086	-0.847635
C	-2.872388	1.265455	-1.867808
O	-2.045505	1.003477	1.194156
C	-2.696097	2.230789	1.638875

O	-4.158678	-0.508940	1.238688
C	-4.010247	-1.062590	2.566971
O	0.988353	-4.113722	0.144147
C	2.110765	-3.357345	-0.371744
O	-1.272509	-5.070256	0.432219
C	-1.133993	-5.224647	1.866081
Br	-4.583429	4.544786	-0.946618
H	-1.796328	-6.042001	2.148195
P	2.528070	1.911926	0.001464
H	0.487320	-0.005062	0.867838
H	-0.808243	-1.729859	-2.858285
H	0.859831	-0.376325	-4.062117
H	-1.906548	2.833071	2.086573
H	-3.151294	2.765997	0.800672
H	-3.454679	1.996145	2.388498
H	-5.019089	-1.253744	2.930449
H	-3.450241	-2.001692	2.546906
H	-3.510042	-0.351502	3.229069
H	-2.920058	0.702894	-2.801332
H	-3.375058	2.231122	-1.954926
H	-1.829655	1.418365	-1.584495
H	2.997218	-3.959446	-0.173383
H	2.009679	-3.194083	-1.446612
H	2.185884	-2.399780	0.144091
H	-0.102118	-5.473146	2.119025
H	-1.438116	-4.313169	2.389121
H	-0.228565	-5.562517	-3.417745
H	0.759627	-5.856783	-1.958492
H	-0.957788	-6.358962	-1.990587
C	4.225261	1.793805	-0.664980
O	1.973191	3.314953	-0.109123
C	2.603495	1.367358	1.747339
C	1.916711	2.138423	2.695628
C	1.931166	1.773674	4.043569
C	2.633598	0.638871	4.452416
C	3.326242	-0.130550	3.512934
C	3.313816	0.231725	2.166200
H	1.381891	3.024490	2.369570
H	1.397325	2.376774	4.771706
H	2.646975	0.355900	5.500680
H	3.879850	-1.009181	3.829643
H	3.866294	-0.367603	1.449362
C	4.967925	2.981959	-0.740598
C	6.284649	2.961186	-1.201486
C	6.870220	1.754324	-1.590211
C	6.135507	0.568083	-1.523842
C	4.817344	0.585579	-1.065704
H	4.503794	3.918437	-0.448166
H	6.850744	3.885798	-1.260305
H	7.894705	1.738467	-1.949670

H	6.585332	-0.369916	-1.834457
H	4.250650	-0.340106	-1.038680

Table S88. XYZ Coordinates of A_meta_IV_P(O)Ph$_2$

84
scf done: -7251.325643

C	0.829005	-1.508677	-2.210379
C	2.117469	-1.583554	-2.745552
C	3.153583	-0.838345	-2.184177
C	2.898477	-0.025615	-1.068460
C	1.600341	0.020726	-0.530029
C	0.541311	-0.707975	-1.089197
H	4.149148	-0.887729	-2.614113
Ni	-1.301139	-0.734147	-0.543417
P	-3.453340	-1.216488	-0.329454
O	-4.560862	-0.728602	-1.404716
C	-5.509080	0.344756	-1.188134
P	-1.433086	1.187434	-1.541197
O	-0.620193	2.367137	-0.774676
C	-0.915304	2.651437	0.617891
P	-0.648451	-2.233596	0.900779
O	0.128951	-3.570152	0.397262
C	-0.562941	-4.529003	-0.435945
O	-2.942006	1.723427	-1.679039
C	-3.264407	3.070892	-2.119860
O	-0.879442	1.369084	-3.048983
C	0.439953	1.833078	-3.417804
O	-1.830937	-2.772041	1.872596
C	-1.544134	-3.544169	3.062621
O	0.437320	-1.618586	1.924056
C	1.695860	-2.235987	2.281743
O	-4.056977	-0.859951	1.125578
C	-5.175302	-1.542391	1.735784
O	-3.527878	-2.818214	-0.582668
C	-4.685305	-3.550217	-1.053239
Br	-4.727176	3.275960	1.345980
P	4.196552	1.123613	-0.490362
H	1.419167	0.645053	0.338364
H	0.038747	-2.082673	-2.690357
H	2.310755	-2.215720	-3.607560
H	-2.507866	-3.845946	3.471496
H	-0.955030	-4.431007	2.816303
H	-1.009630	-2.928266	3.788994
H	0.098376	-5.390951	-0.518943
H	-1.508894	-4.825733	0.021062
H	-0.748187	-4.111926	-1.428395
H	2.195209	-1.523834	2.937227
H	1.531958	-3.177891	2.808666
H	2.300640	-2.408818	1.390571
H	-4.306172	-4.498778	-1.433251

H	-5.374989	-3.736534	-0.227026
H	-5.191855	-3.001980	-1.847323
H	-5.422008	-0.981918	2.636885
H	-6.042383	-1.558043	1.070805
H	-4.881396	-2.560340	2.000462
H	-5.591472	0.885565	-2.130778
H	-6.476232	-0.098296	-0.934727
H	-5.187390	1.029755	-0.401249
H	-3.845976	2.991398	-3.039965
H	-3.853481	3.519053	-1.317209
H	-2.361174	3.655119	-2.303603
H	0.353603	2.199300	-4.441451
H	0.761810	2.641366	-2.761313
H	1.150509	1.007074	-3.377917
H	-0.310064	3.519503	0.879064
H	-1.978040	2.874225	0.757121
H	-0.625254	1.807324	1.250793
C	3.816291	1.549350	1.249495
C	5.759930	0.177630	-0.471335
O	4.300669	2.371291	-1.341818
C	3.195462	2.784199	1.492041
C	2.879880	3.171300	2.795707
C	3.189043	2.332301	3.867924
C	3.819206	1.106819	3.635614
C	4.132055	0.715640	2.333071
H	2.975041	3.441879	0.657526
H	2.399642	4.128773	2.973112
H	2.947949	2.634797	4.882503
H	4.074390	0.458777	4.468632
H	4.635578	-0.231440	2.168582
C	6.933860	0.871639	-0.801013
C	8.168007	0.221394	-0.773358
C	8.239698	-1.127147	-0.416975
C	7.074843	-1.827206	-0.093773
C	5.838518	-1.180110	-0.122779
H	6.869784	1.916168	-1.088594
H	9.071076	0.765381	-1.033016
H	9.200019	-1.633537	-0.396751
H	7.126852	-2.877995	0.174228
H	4.938102	-1.739789	0.111003

Table S89. XYZ Coordinates of A_meta_TS2_P(O)Ph$_2$

84

scf done: -7251.246796

C	-3.935786	1.731071	-1.077243
C	-4.843072	0.722847	-0.716696
C	-6.218421	1.004306	-0.701905
C	-6.679935	2.278894	-1.030558
C	-5.772189	3.282557	-1.378137
C	-4.403510	3.006668	-1.402468

P	-4.329195	-0.972570	-0.258726
O	-5.384345	-1.998708	-0.607035
C	-2.738750	-1.296391	-1.095764
C	-1.504458	-0.864410	-0.587419
C	-0.295836	-1.116135	-1.255821
C	-0.375501	-1.822204	-2.470803
C	-1.598427	-2.258961	-2.992120
C	-2.784245	-2.003454	-2.306913
Ni	1.451829	-0.600821	-0.663037
P	1.560031	-2.643419	0.091058
O	1.741097	-3.767171	-1.054560
C	0.943114	-4.967487	-1.192066
P	0.813776	1.485516	-0.879072
O	-0.538670	1.885813	-1.750596
C	-0.517673	1.728454	-3.177349
O	0.481612	1.944332	0.551238
C	-0.285258	3.876681	1.274532
O	1.926703	2.422701	-1.636554
C	1.774006	3.857175	-1.707224
P	3.627840	-0.237952	-0.416784
O	4.400471	0.921816	-1.249715
C	4.801108	2.173172	-0.649668
O	4.335301	-1.532708	-1.090363
C	5.773506	-1.656798	-1.182172
O	4.308021	-0.099535	1.052416
C	3.540805	0.125373	2.260014
O	0.270248	-3.240095	0.889401
C	-0.110319	-2.616383	2.134442
O	2.774461	-2.871736	1.149430
C	3.100508	-4.178321	1.676218
C	-3.984105	-0.940353	1.539053
C	-3.513562	0.194886	2.215421
C	-3.252981	0.138670	3.586036
C	-3.468053	-1.046837	4.292410
C	-3.949240	-2.177925	3.627767
C	-4.207083	-2.125495	2.257293
Br	2.340346	3.919801	1.980486
H	-3.735064	-2.356213	-2.693304
H	-1.486193	-0.316165	0.349816
H	0.532073	-2.058348	-3.021736
H	-1.621726	-2.806236	-3.930698
H	4.031997	2.580764	0.009404
H	5.730460	2.029652	-0.093157
H	4.970121	2.864134	-1.476051
H	5.964633	-2.591841	-1.708173
H	6.193409	-0.818965	-1.741434
H	6.211626	-1.697539	-0.182095
H	2.751121	-0.621430	2.359560
H	4.246329	0.011409	3.083912
H	3.124951	1.136177	2.266473

H	0.762066	4.131417	-2.018727
H	1.998490	4.294361	-0.731602
H	2.488414	4.210130	-2.453182
H	-1.525860	1.947345	-3.533246
H	0.189336	2.422111	-3.641204
H	-0.257760	0.703616	-3.457479
H	0.969867	-5.564755	-0.279634
H	-0.087299	-4.704473	-1.436297
H	1.390299	-5.525533	-2.014517
H	-0.171010	-1.529370	2.031228
H	-1.095853	-3.004526	2.388970
H	0.603825	-2.869080	2.922117
H	3.862982	-4.021610	2.438739
H	2.222264	-4.649993	2.123739
H	3.497997	-4.811985	0.879862
H	-0.488005	3.460423	2.249019
H	0.098867	4.882703	1.238138
H	-0.901714	3.554101	0.452854
H	-3.359105	1.126709	1.680414
H	-2.889732	1.022176	4.102092
H	-3.269061	-1.086922	5.359118
H	-4.129232	-3.097373	4.176683
H	-4.595471	-2.997119	1.740093
H	-6.922567	0.218796	-0.445884
H	-7.745527	2.487416	-1.019124
H	-6.131426	4.274594	-1.635334
H	-3.696258	3.781950	-1.681845
H	-2.869425	1.530579	-1.119075

Table S90. XYZ Coordinates of A_meta_V_P(O)Ph$_2$

79
scf done: -4639.692553

C	3.808988	-1.999365	-0.302756
C	4.629679	-0.878939	-0.505476
C	6.025091	-1.024952	-0.439482
C	6.591735	-2.268582	-0.162166
C	5.770005	-3.379138	0.048293
C	4.382441	-3.243013	-0.024984
P	3.976178	0.790516	-0.876796
O	4.859651	1.549243	-1.842668
C	2.278920	0.565482	-1.502279
C	2.090928	0.587054	-2.891856
C	0.813934	0.371838	-3.406615
C	-0.263297	0.140356	-2.544695
C	-0.105209	0.113556	-1.144647
C	1.187883	0.336798	-0.649951
Ni	-1.715486	-0.264039	-0.184840
P	-2.151003	1.853360	-0.371023
O	-3.277030	2.351081	0.699364
C	-3.766649	3.712921	0.706401

P	-0.803114	-2.148195	0.515333
O	-1.675907	-3.239909	1.102015
P	-3.784192	-0.910702	0.223927
O	-4.416914	-1.150086	1.702239
C	-3.583573	-1.314266	2.875795
O	0.252465	-1.575194	1.655352
C	0.865894	-2.504106	2.565146
O	0.322911	-2.853414	-0.492850
C	-0.188580	-3.607494	-1.596248
O	-4.032682	-2.268550	-0.629888
C	-5.351855	-2.841839	-0.766285
O	-4.945070	0.034038	-0.429245
C	-5.966829	0.715224	0.323307
O	-2.664411	2.330719	-1.829269
C	-2.125906	3.436637	-2.592057
O	-0.939460	2.920667	-0.132861
C	-0.310493	2.955571	1.165360
C	3.832247	1.653059	0.732178
C	3.921345	3.053920	0.724605
C	3.797013	3.774537	1.912995
C	3.585532	3.101944	3.119640
C	3.505815	1.707780	3.136435
C	3.631841	0.984355	1.948661
H	2.931837	0.779008	-3.550561
H	1.353700	0.306829	0.421102
H	-1.246264	-0.010786	-2.986845
H	0.652676	0.389097	-4.481363
H	-5.232449	-3.746129	-1.362732
H	-5.757964	-3.099260	0.215205
H	-6.020343	-2.144476	-1.275581
H	-6.413345	1.438034	-0.360811
H	-6.729257	0.008604	0.659545
H	-5.541685	1.236546	1.181134
H	-3.019312	-0.397554	3.069985
H	-4.268694	-1.498338	3.703765
H	-2.903153	-2.155197	2.738720
H	0.664978	-4.070283	-2.096018
H	-0.876930	-4.388559	-1.258026
H	-0.705425	-2.958137	-2.313880
H	1.279124	-1.919195	3.389210
H	0.130138	-3.215032	2.950507
H	1.673495	-3.051143	2.068798
H	-2.743101	3.506371	-3.487746
H	-2.182793	4.368985	-2.028334
H	-1.090271	3.231533	-2.868382
H	-0.077916	1.948041	1.520688
H	0.617309	3.515094	1.049062
H	-0.957706	3.457021	1.889480
H	-4.401372	3.809799	1.586828
H	-2.939891	4.424438	0.767211

H	-4.353641	3.902419	-0.195488
H	4.101885	3.572713	-0.211689
H	3.871331	4.857763	1.898466
H	3.490890	3.662574	4.044781
H	3.352170	1.182046	4.073966
H	3.581422	-0.099711	1.974575
H	6.664136	-0.165797	-0.619659
H	7.671605	-2.372172	-0.114648
H	6.210884	-4.348345	0.261914
H	3.741280	-4.106451	0.125913
H	2.728390	-1.916734	-0.368623

Table S91. XYZ Coordinates of A_meta_TS3_P(O)Ph$_2$

79

scf done: -4639.6481807

C	-1.021088	0.163932	-0.595744
C	-2.001427	-0.419098	-1.396454
C	-1.942877	-0.264633	-2.787761
C	-0.919111	0.502263	-3.362917
C	0.047200	1.088176	-2.561803
C	0.058385	0.888131	-1.156149
P	-3.410325	-1.327447	-0.666622
O	-3.954399	-2.409310	-1.575779
Ni	1.693011	0.470185	-0.220633
P	2.362947	-1.024636	-1.563757
O	2.840814	-0.551259	-3.072642
C	3.899849	0.411373	-3.154148
P	0.515486	2.388344	-0.078479
O	0.037308	2.228046	1.473234
C	-0.630708	3.297040	2.192678
C	-4.655507	-0.054455	-0.246414
C	-5.961717	-0.512422	0.001095
C	-6.976432	0.387465	0.324598
C	-6.698749	1.756855	0.390634
C	-5.408213	2.219719	0.132728
C	-4.385695	1.320707	-0.187435
C	-2.765171	-2.000169	0.905499
C	-2.794369	-1.290058	2.116260
C	-2.241646	-1.847577	3.271475
C	-1.649868	-3.111712	3.226042
C	-1.618372	-3.826211	2.022768
C	-2.188597	-3.278874	0.872875
P	2.748358	0.361468	1.614265
O	2.172089	-0.719000	2.721436
C	2.206986	-2.133147	2.452107
O	4.311249	-0.189387	1.573120
C	5.262164	0.566402	0.811252
O	2.838543	1.683114	2.560152
C	3.541846	1.675833	3.819224
O	1.753623	3.144909	-0.502300

O	-0.793386	3.296246	-0.528785
C	-0.741120	4.175008	-1.656185
O	3.628295	-1.932283	-1.040122
C	4.258055	-2.921841	-1.879261
O	1.329080	-2.167755	-2.152929
C	0.783245	-3.117405	-1.214213
H	-0.622980	2.996323	3.240745
H	-2.697403	-0.732112	-3.413561
H	-1.078129	0.076003	0.485605
H	0.846208	1.669267	-3.017335
H	-0.881247	0.626501	-4.441686
H	1.559916	-2.608349	3.192209
H	1.852950	-2.368913	1.444471
H	3.225566	-2.511573	2.556936
H	5.570903	1.468234	1.352173
H	6.126327	-0.078777	0.660458
H	4.857346	0.864992	-0.160620
H	4.532932	1.227631	3.716462
H	3.643644	2.719777	4.120061
H	2.972251	1.124161	4.568591
H	3.512194	-3.547724	-2.375089
H	4.885873	-2.447578	-2.638110
H	4.875963	-3.534005	-1.221177
H	0.081176	-3.734169	-1.778687
H	1.573584	-3.753044	-0.804850
H	0.240392	-2.631889	-0.397751
H	4.834572	0.013591	-2.743656
H	4.042146	0.628284	-4.213931
H	3.644145	1.337597	-2.630461
H	-0.091963	4.240953	2.077923
H	-1.654140	3.408813	1.836409
H	-1.628158	4.807761	-1.596461
H	0.163528	4.786802	-1.630586
H	-0.766086	3.604341	-2.590756
H	-2.179246	-3.843698	-0.055286
H	-1.164451	-4.811735	1.984898
H	-1.217635	-3.542472	4.124459
H	-2.266458	-1.293519	4.204683
H	-3.257179	-0.308727	2.159520
H	-6.186649	-1.573267	-0.060765
H	-7.980256	0.024252	0.522108
H	-7.489895	2.458602	0.638408
H	-5.192115	3.283026	0.175403
H	-3.387024	1.705133	-0.381708

Table S92. XYZ Coordinates of A_meta_VI_P(O)Ph_2

79

scf done: -4639.695242

C	-3.284493	1.598615	1.283387
C	-3.381763	0.198276	1.295108

C	-3.938066	-0.441034	2.413034
C	-4.397640	0.306956	3.497761
C	-4.304259	1.700380	3.475728
C	-3.745670	2.344662	2.369271
P	-2.863438	-0.853013	-0.112918
C	-1.470690	0.018481	-0.900316
C	-1.489280	0.237430	-2.257772
C	-0.491181	1.041015	-2.890218
C	0.465332	1.667304	-2.141790
C	0.582428	1.440345	-0.714213
C	-0.367636	0.510749	-0.078636
Ni	1.453793	-0.208226	-0.162538
P	1.300637	-1.967882	1.012351
O	0.326705	-1.845143	2.309947
C	-0.546439	-2.885016	2.811805
P	1.193738	2.841448	0.229841
O	2.381755	3.555458	-0.333025
C	-4.265752	-0.816194	-1.296630
C	-4.926259	0.364570	-1.671070
C	-5.999721	0.317563	-2.559793
C	-6.425116	-0.907819	-3.081667
C	-5.776402	-2.086347	-2.710264
C	-4.701814	-2.041488	-1.819236
O	-2.590554	-2.277827	0.326004
P	3.433955	-0.155899	-0.989911
O	3.978677	-1.544347	-1.683179
C	4.798505	-2.496551	-0.980766
O	4.774966	0.209447	-0.112562
C	4.692324	1.224712	0.908643
O	3.526771	0.796636	-2.312564
C	4.718567	0.855426	-3.122874
O	1.359256	2.220074	1.714331
C	1.690269	3.091000	2.818997
O	-0.065252	3.847817	0.505482
C	-0.395559	4.847851	-0.477017
O	2.750880	-2.366182	1.690531
C	2.866432	-3.363811	2.724491
O	0.753372	-3.390434	0.400850
C	1.381022	-3.926454	-0.777743
H	-2.304853	-0.143847	-2.862813
H	-0.606335	0.676655	0.969212
H	1.166290	2.349125	-2.612381
H	-0.543756	1.209462	-3.961357
H	2.291651	-4.470111	-0.510000
H	0.667432	-4.614665	-1.232576
H	1.629867	-3.137100	-1.494064
H	-0.967134	-2.493065	3.739522
H	-1.339682	-3.072475	2.088149
H	0.007310	-3.801788	3.026183
H	3.931941	-3.498231	2.914589

H	2.372484	-3.023673	3.638291
H	2.432574	-4.314167	2.400915
H	5.698161	1.330232	1.317666
H	4.357042	2.176501	0.489697
H	4.006535	0.918527	1.704080
H	4.545565	1.629497	-3.871504
H	5.585901	1.122310	-2.513290
H	4.896301	-0.104142	-3.612906
H	4.884478	-3.366768	-1.633771
H	5.790970	-2.080704	-0.793350
H	4.339799	-2.789438	-0.034097
H	2.596413	3.661436	2.600067
H	0.861951	3.772774	3.023548
H	1.861784	2.444377	3.679046
H	-0.808026	4.382207	-1.377860
H	-1.151157	5.491738	-0.025449
H	0.484897	5.438643	-0.740507
H	-3.999532	-1.524407	2.429186
H	-4.824978	-0.197122	4.359404
H	-4.660323	2.282641	4.320350
H	-3.662080	3.427074	2.353495
H	-2.833331	2.109479	0.438318
H	-4.198355	-2.953564	-1.516069
H	-6.106679	-3.040273	-3.110421
H	-7.262226	-0.941836	-3.772585
H	-6.505826	1.235483	-2.843157
H	-4.610113	1.322028	-1.268773

Table S93. XYZ Coordinates of A_meta_I_Me

48

scf done: -5724.487399

C	0.793683	-0.579706	-0.303335
C	0.232447	-0.123472	0.938036
C	1.099675	0.144714	2.070323
C	2.509265	-0.170977	1.938265
C	2.979490	-0.716867	0.769280
C	2.120462	-0.912187	-0.357267
Ni	-0.290130	-1.212316	2.391855
P	0.161709	-1.669452	4.429729
O	-0.845579	-2.499960	5.438803
C	-2.220090	-2.086938	5.526123
Br	-1.274774	1.203046	0.708698
C	3.425086	0.159557	3.087655
P	-1.829347	-2.533993	1.756313
O	-2.001511	-2.992406	0.179202
C	-0.890018	-3.677962	-0.426133
O	-1.744222	-3.962949	2.544636
C	-2.740388	-4.993124	2.366157
O	-3.398416	-2.155124	2.054072
C	-4.138700	-1.275268	1.185477

O	0.540670	-0.345555	5.303453
C	0.959659	-0.433312	6.683450
O	1.503533	-2.574266	4.697102
C	1.527028	-3.931639	4.216328
H	0.989389	0.588803	7.062178
H	4.036459	-0.950975	0.673180
H	0.863139	0.967023	2.743489
H	0.153555	-0.689378	-1.172533
H	2.535273	-1.314730	-1.277482
H	-2.709583	-2.773527	6.218473
H	-2.297978	-1.067337	5.917245
H	-2.713485	-2.145176	4.552009
H	2.543493	-4.298321	4.363807
H	0.824560	-4.550777	4.780159
H	1.274568	-3.978339	3.152148
H	0.250262	-1.023723	7.267750
H	1.952861	-0.882887	6.749422
H	-5.082536	-1.064678	1.690346
H	-3.596272	-0.341977	1.015588
H	-4.334739	-1.765195	0.228745
H	-2.326002	-5.903040	2.801625
H	-3.662315	-4.719052	2.883411
H	-2.952319	-5.155495	1.306518
H	0.048555	-3.146973	-0.244808
H	-0.813654	-4.700558	-0.044356
H	-1.081256	-3.707118	-1.499838
H	3.128237	-0.388775	3.988364
H	4.464797	-0.087982	2.857156
H	3.375561	1.227320	3.335917

Table S94. XYZ Coordinates of A_meta_TS1_Me

48

scf done: -5724.483043

C	0.131862	-0.171362	0.279041
C	0.368809	-0.206212	1.682522
C	1.728586	-0.240902	2.124132
C	2.742280	-0.110238	1.189320
C	2.454276	0.098961	-0.183774
C	1.156923	0.108804	-0.652127
Ni	-0.896035	-1.639396	0.679874
P	-0.686532	-3.008248	-0.925129
O	-1.145174	-4.531301	-0.531417
C	-1.124364	-5.607677	-1.492914
C	2.020416	-0.315022	3.602055
Br	-1.867940	0.564681	-0.115089
P	-1.936249	-2.480482	2.359031
O	-2.420694	-1.325792	3.408223
C	-3.098451	-1.646388	4.642589
O	-1.126955	-3.448562	3.404756
C	-0.654249	-4.733273	2.958141

O	-3.283320	-3.415344	2.172420
C	-4.267852	-2.982040	1.217477
O	-1.571079	-2.847596	-2.306983
C	-1.350183	-1.711127	-3.160625
O	0.779858	-3.250219	-1.656610
C	1.905527	-3.543296	-0.813562
H	-3.459766	-0.702142	5.051331
H	3.777456	-0.108700	1.518636
H	-0.382028	0.089729	2.405733
H	0.935420	0.222584	-1.707021
H	3.273982	0.227191	-0.885405
H	-4.931813	-3.828322	1.036802
H	-4.847395	-2.142702	1.614668
H	-3.799234	-2.680790	0.274415
H	0.020172	-5.103671	3.731231
H	-1.491119	-5.426103	2.837025
H	-0.115343	-4.649537	2.010117
H	-3.942176	-2.315533	4.457490
H	-2.405544	-2.114859	5.345072
H	-2.077463	-1.781324	-3.971166
H	-1.508694	-0.776940	-2.614060
H	-0.339304	-1.732012	-3.577739
H	-1.266822	-6.530144	-0.928372
H	-1.933787	-5.486866	-2.216370
H	-0.167402	-5.642113	-2.019353
H	2.027912	-2.786583	-0.032062
H	1.797699	-4.530440	-0.352096
H	2.789685	-3.540775	-1.453257
H	1.594010	-1.221578	4.046036
H	3.095542	-0.311148	3.798423
H	1.576035	0.536191	4.132103

Table S95. XYZ Coordinates of A_meta_IIa_Me

48

scf done: -5724.533358

C	0.027680	0.036402	0.017180
C	0.022515	0.040963	1.423308
C	1.250647	0.030628	2.094042
C	2.446658	0.010702	1.370694
C	2.428080	-0.002686	-0.024738
C	1.213334	0.001383	-0.731206
H	1.272034	0.042597	3.181227
Ni	1.138761	-0.237750	-2.619352
P	0.888098	1.850322	-2.709145
O	1.098431	2.579624	-4.152099
C	2.412680	2.644940	-4.738204
Br	1.704524	-2.489986	-2.291780
P	0.843269	-0.702046	-4.798133
O	0.260604	0.367697	-5.892509
C	-1.080864	0.868065	-5.730143

O	-0.183781	-1.951255	-4.959394
C	-0.531386	-2.555375	-6.224426
O	2.255336	-1.040974	-5.515690
C	2.501760	-1.074814	-6.942078
O	-0.635970	2.283466	-2.382931
C	-1.118248	3.636001	-2.590593
O	1.853382	2.829117	-1.840234
C	1.636751	3.189612	-0.458829
H	3.396818	0.004787	1.899928
H	3.372050	-0.025124	-0.564107
H	-0.933104	0.065859	-0.494651
C	-1.282351	0.034498	2.186993
H	1.745395	4.274427	-0.392292
H	0.641196	2.896084	-0.123753
H	2.384280	2.696179	0.161380
H	3.585102	-1.050030	-7.059940
H	2.110144	-1.998888	-7.372862
H	2.052048	-0.211323	-7.432665
H	-1.199293	1.677049	-6.450913
H	-1.813873	0.083735	-5.936127
H	-1.237015	1.260086	-4.722252
H	-1.518071	-3.002368	-6.098663
H	-0.566093	-1.814150	-7.026132
H	0.194078	-3.335650	-6.468063
H	-2.087938	3.688742	-2.096589
H	-0.437058	4.365975	-2.148630
H	-1.226497	3.831572	-3.658338
H	2.294313	3.124886	-5.709511
H	3.081504	3.237174	-4.110063
H	2.827810	1.642791	-4.882679
H	-2.048360	0.627533	1.677284
H	-1.679305	-0.983640	2.287296
H	-1.157378	0.434957	3.197410

Table S96. XYZ Coordinates of A_meta_IIb_Me

48

scf done: -5724.542551

C	-0.205680	-0.024615	0.072171
C	-0.094532	0.029192	1.471207
C	1.200933	0.058758	2.012802
C	2.355190	0.021824	1.216774
C	2.204872	-0.033572	-0.175714
C	0.930166	-0.053730	-0.742022
Ni	-1.627130	0.086389	2.568174
Br	-3.596954	0.222855	3.851465
H	3.085546	-0.054943	-0.813371
P	-1.503209	2.265912	2.642093
O	-0.218003	2.925794	1.898373
C	-0.032119	4.358727	1.856573
O	-2.772465	3.094179	2.042621

C	-3.238149	2.738197	0.724596
O	-1.527093	2.994451	4.099876
C	-0.579322	2.532213	5.084281
P	-1.793138	-2.063510	2.255982
O	-0.479240	-2.993342	2.000589
C	0.442633	-3.154022	3.097670
O	-2.521038	-2.488781	0.861152
C	-3.796331	-1.900679	0.527881
O	-2.587544	-2.859348	3.429983
C	-2.913615	-4.262964	3.304660
H	-3.662948	1.730070	0.725629
C	3.729090	0.023607	1.847949
H	1.332300	0.135385	3.091668
H	-1.186696	-0.041861	-0.397733
H	0.819783	-0.089442	-1.823359
H	-4.014840	3.458774	0.468769
H	-2.428800	2.797407	-0.008761
H	0.908460	4.529376	1.332973
H	-0.851016	4.836568	1.314016
H	0.025514	4.769839	2.866927
H	-0.694431	3.183336	5.950766
H	-0.795735	1.499415	5.373666
H	0.444944	2.605309	4.707074
H	-3.240564	-4.590179	4.291644
H	-3.722496	-4.399997	2.583271
H	-2.043284	-4.843813	2.991321
H	-4.072182	-2.305142	-0.445832
H	-4.555022	-2.169509	1.267605
H	-3.721486	-0.811809	0.465732
H	1.245745	-3.793679	2.731710
H	0.859143	-2.190126	3.398942
H	-0.044833	-3.632429	3.951305
H	4.463394	0.523262	1.208544
H	3.723250	0.528061	2.818958
H	4.091200	-0.998590	2.016468

Table S97. XYZ Coordinates of A_meta_III_Me

48

scf done: -5724.482775

C	-0.054304	0.100556	0.199372
C	-0.028319	-0.034028	1.588487
C	1.187832	-0.072248	2.276740
C	2.392826	0.033767	1.566477
C	2.355291	0.160898	0.173980
C	1.138286	0.201351	-0.527862
Ni	3.991415	0.065294	2.478130
P	4.462755	2.150746	1.905848
O	5.743162	2.728773	2.732920
C	7.028378	2.098145	2.531059
C	1.135008	0.416423	-2.022101

P	3.844171	-2.017684	3.121322
O	5.214149	-2.517671	3.833061
C	6.398988	-2.750867	3.033825
O	2.764185	-2.225000	4.293780
C	2.628781	-3.478379	5.018146
O	3.643739	-3.226415	2.060205
C	2.449641	-3.357140	1.249379
O	3.487851	3.372662	2.303250
C	2.158296	3.515328	1.732521
O	4.834022	2.273660	0.345397
C	5.269483	3.520394	-0.276786
Br	2.031470	4.325733	-2.143300
H	-1.006214	0.132308	-0.324063
H	7.205842	-2.953811	3.736883
H	3.279129	0.254784	-0.389843
H	1.184473	-0.187239	3.356293
H	-0.960217	-0.113245	2.142181
H	6.183428	3.294610	-0.827900
H	4.475522	3.847215	-0.952725
H	5.472177	4.284500	0.476137
H	7.439921	2.372303	1.556787
H	7.679249	2.468252	3.322324
H	6.959004	1.008643	2.604701
H	1.739626	4.406756	2.199699
H	2.208617	3.657391	0.648648
H	1.542638	2.648504	1.976968
H	2.491643	-4.354867	0.813271
H	1.553099	-3.261292	1.865548
H	2.440146	-2.602199	0.462606
H	6.247200	-3.610198	2.378489
H	6.648791	-1.867373	2.438603
H	1.699798	-3.400913	5.581115
H	2.575918	-4.318702	4.323517
H	3.474059	-3.604089	5.696013
H	0.157059	0.191182	-2.456853
H	1.377144	1.463604	-2.247906
H	1.883681	-0.209227	-2.519289

Table S98. XYZ Coordinates of A_meta_IV_Me

64

scf done: -6411.320330

C	-0.545888	1.859500	0.133737
C	-0.202007	2.624982	1.258135
C	-0.085433	4.014762	1.170840
C	-0.290737	4.666920	-0.045924
C	-0.614907	3.933059	-1.194146
C	-0.746728	2.539513	-1.079651
C	-0.799022	4.619951	-2.527957
Ni	-0.802538	-0.038731	0.109451
P	-2.914216	0.410203	0.371151

O	-3.625908	0.691246	-1.048969
C	-4.634998	1.700057	-1.303120
P	-1.113463	-2.202270	-0.257788
O	-1.131056	-3.081314	1.094271
C	-0.479219	-4.362647	1.275281
P	1.365934	-0.242985	0.108995
O	1.790916	-1.585288	0.906340
C	3.011268	-1.750055	1.681211
O	-0.021955	-3.028713	-1.144187
C	0.065427	-2.779293	-2.566267
O	-2.507948	-2.475489	-1.041277
C	-3.049298	-3.806551	-1.211210
O	2.304811	0.827533	0.866895
C	2.890976	1.990517	0.229597
O	1.909200	-0.343873	-1.412275
C	3.179717	-0.948208	-1.783856
O	-3.410598	1.714756	1.206441
C	-3.217305	1.739830	2.637165
O	-3.699439	-0.807823	1.104948
C	-5.142271	-0.866444	1.185098
Br	6.244137	0.127922	0.242314
H	-0.197007	5.748519	-0.105942
H	-1.023580	1.981732	-1.973322
H	-0.009146	2.142940	2.212755
H	0.169549	4.591527	2.056553
H	-5.380934	-1.676730	1.873460
H	-5.553886	0.071792	1.565307
H	-5.558383	-1.084658	0.198706
H	-3.449418	2.754155	2.960259
H	-3.889927	1.032490	3.127479
H	-2.182630	1.507368	2.898258
H	-4.874633	1.614481	-2.362605
H	-5.530096	1.518453	-0.705736
H	-4.238023	2.692238	-1.086381
H	-0.664620	-4.642296	2.311980
H	-0.902742	-5.115299	0.607850
H	0.591749	-4.264921	1.096963
H	-3.896176	-3.710061	-1.889848
H	-2.306262	-4.480881	-1.644163
H	-3.388565	-4.193676	-0.247680
H	0.897778	-3.382195	-2.928325
H	-0.854839	-3.083380	-3.069416
H	0.269427	-1.724318	-2.762388
H	3.268255	-0.811064	-2.861481
H	4.019499	-0.471781	-1.269512
H	3.152538	-2.014839	-1.548815
H	3.184679	-2.825437	1.731565
H	3.864936	-1.248063	1.220455
H	2.850364	-1.353717	2.685797
H	2.647829	2.855976	0.845467

H	3.969515	1.820776	0.187366
H	2.487509	2.135724	-0.773889
H	-1.460454	4.048153	-3.185488
H	-1.220552	5.622491	-2.406777
H	0.159044	4.733850	-3.050532

Table S99. XYZ Coordinates of A_meta_TS2_Me

64

scf done: -6411.237764

Br	-4.806434	-0.495336	1.118359
C	-3.123447	-2.468092	1.912242
O	-1.827240	-1.038324	0.844329
P	-0.909307	-1.375794	-0.348021
O	-1.727866	-1.480943	-1.770202
C	-2.956919	-2.239595	-1.842610
Ni	0.628385	0.178352	-0.309656
P	-0.715735	1.842514	-0.899037
O	0.240165	2.907898	-1.661456
C	-0.294764	4.080118	-2.318608
C	1.999382	-1.141395	-0.423359
C	2.511904	-1.985524	0.569887
C	3.540563	-2.908540	0.321103
C	4.066945	-2.990310	-0.974815
C	3.574544	-2.162463	-1.986026
C	2.553552	-1.247874	-1.714251
H	4.859490	-3.702402	-1.191729
P	2.130060	1.438239	0.610591
O	2.822928	0.864659	1.974021
C	1.977586	0.563119	3.102716
O	1.547854	2.883912	1.068424
C	2.394668	3.893054	1.667756
O	3.447810	1.843507	-0.237372
C	4.722649	1.164587	-0.151437
O	-0.533333	-2.962600	-0.070395
C	0.051647	-3.791211	-1.087615
O	-1.575157	2.739544	0.148597
C	-1.850680	2.311962	1.505105
O	-1.787011	1.582054	-2.095976
C	-3.190910	1.363875	-1.836299
C	4.083301	-3.773831	1.435524
H	2.102477	-1.945343	1.576413
H	2.191903	-0.617016	-2.524788
H	3.983644	-2.233870	-2.990863
H	-3.357073	0.708804	-0.979256
H	-3.685150	2.323690	-1.665834
H	-3.596679	0.897318	-2.734554
H	0.555047	4.586439	-2.775917
H	-1.016728	3.792311	-3.084781
H	-0.768087	4.738921	-1.586575
H	-0.931553	1.996680	2.003544

H	-2.252316	3.188372	2.015164
H	-2.582475	1.501217	1.508495
H	-2.766793	-3.306089	-1.688022
H	-3.670502	-1.877043	-1.097512
H	-3.355727	-2.087629	-2.846923
H	-0.211005	-4.823701	-0.845560
H	-0.344741	-3.544831	-2.076266
H	1.137423	-3.682347	-1.091438
H	4.641375	0.152520	-0.550555
H	5.411762	1.749677	-0.760938
H	5.070765	1.138897	0.881822
H	1.150793	-0.094208	2.816347
H	2.605281	0.053579	3.833579
H	1.577330	1.482223	3.538816
H	1.729816	4.686946	2.007133
H	2.947257	3.483459	2.516482
H	3.094052	4.281514	0.924899
H	-2.993948	-1.933294	2.840442
H	-4.105226	-2.861681	1.706661
H	-2.282498	-3.018364	1.524922
H	4.509945	-4.704194	1.049073
H	4.878430	-3.256909	1.987592
H	3.303548	-4.030074	2.159276

Table S100. XYZ Coordinates of A_meta_V_Me

59

scf done: -3799.682471

C	2.874014	0.102981	0.376810
C	1.737747	0.171936	-0.443009
C	1.944081	0.519416	-1.792841
C	3.227107	0.771136	-2.289652
C	4.336158	0.698253	-1.446905
C	4.168492	0.365903	-0.095890
Ni	-0.101889	-0.148611	-0.019460
P	-0.265099	2.000707	0.216950
O	-1.580547	2.425927	1.085745
C	-1.924953	3.813938	1.304899
C	5.356848	0.306721	0.837673
P	0.387042	-2.271548	0.325377
O	-0.741833	-3.273237	0.482733
P	-2.258452	-0.528520	-0.292512
O	-3.286921	-0.995040	0.877946
C	-2.819968	-1.488777	2.157119
O	1.288531	-2.217000	1.712806
C	1.570808	-3.447763	2.397760
O	1.545616	-2.931035	-0.674929
C	1.117383	-3.316870	-1.984017
O	-2.401328	-1.600803	-1.504375
C	-3.684988	-1.907886	-2.090816
O	-3.108751	0.709469	-0.939115

C	-4.217584	1.361610	-0.293020
O	-0.356603	2.854204	-1.156465
C	0.537973	3.923290	-1.542357
O	0.931195	2.813702	0.975686
C	1.208491	2.480029	2.350681
H	5.332034	0.896384	-1.836641
H	2.764134	-0.197411	1.414667
H	1.101949	0.599294	-2.478065
H	3.359798	1.025859	-3.338662
H	-3.498990	-2.657581	-2.859801
H	-4.360891	-2.316366	-1.335158
H	-4.123846	-1.014262	-2.539655
H	-4.405973	2.272273	-0.863392
H	-5.104558	0.724062	-0.317132
H	-3.976153	1.619693	0.738392
H	-2.263331	-0.708496	2.684417
H	-3.716830	-1.737462	2.725332
H	-2.193014	-2.368985	2.011514
H	1.952849	-3.835925	-2.459299
H	0.253774	-3.988067	-1.938575
H	0.859513	-2.440023	-2.591215
H	1.974503	-3.183708	3.377237
H	0.661744	-4.042505	2.521329
H	2.312808	-4.034745	1.846628
H	0.140926	4.319197	-2.477300
H	0.560709	4.708764	-0.785831
H	1.543212	3.529216	-1.702952
H	1.357401	1.404013	2.472048
H	2.127359	3.001798	2.617857
H	0.394290	2.813287	2.999448
H	-2.780510	3.817792	1.979889
H	-1.092440	4.355191	1.760390
H	-2.198066	4.285237	0.357789
H	5.161147	-0.346924	1.692914
H	6.251929	-0.060673	0.325619
H	5.600523	1.299603	1.236991

Table S101. XYZ Coordinates of A_meta_TS3_Me

59

scf done: -4639.6481807

C	2.728536	-0.047018	-0.251126
C	1.525326	-0.731659	-0.577551
C	1.115687	-0.827695	-1.937298
C	1.814236	-0.092865	-2.912050
C	2.944021	0.634892	-2.561008
C	3.431523	0.656266	-1.227847
Ni	-0.255360	-0.056860	-0.134191
P	0.090085	1.901503	0.639426
O	0.942571	3.007953	-0.252378
C	0.517681	3.286672	-1.594113

P	0.854978	-2.162656	0.471237
O	2.269482	-2.671156	1.206275
C	3.215631	-3.430117	0.443565
C	4.666655	1.450181	-0.880822
P	-2.243291	-0.566170	-0.513484
O	-3.225661	-0.924306	0.770339
C	-3.282262	-0.007979	1.880120
O	0.158420	-1.589463	1.832209
C	0.271150	-2.274721	3.108762
O	-3.241067	0.547578	-1.234011
C	-2.838491	1.035947	-2.521186
O	-2.563158	-1.892111	-1.429044
C	-3.909282	-2.324670	-1.709919
O	0.217824	-3.324514	-0.244974
O	-1.276360	2.755026	1.002999
C	-1.221027	4.119516	1.470372
O	1.041048	2.149975	1.967015
C	0.573179	1.619157	3.223829
H	-0.476530	-1.816798	3.754218
H	3.491739	1.176871	-3.326469
H	3.064305	-0.027409	0.782274
H	0.334800	-1.517894	-2.232827
H	1.496947	-0.137667	-3.949821
H	-4.096442	-0.340907	2.528120
H	-2.342131	-0.024087	2.440252
H	-3.482067	1.009740	1.541945
H	-3.582866	1.769711	-2.836510
H	-1.855572	1.519674	-2.475967
H	-2.801503	0.225196	-3.255552
H	-3.836998	-3.094198	-2.479888
H	-4.369934	-2.744666	-0.812081
H	-4.519875	-1.495916	-2.076156
H	-2.253613	4.455316	1.575894
H	-0.717486	4.180554	2.440230
H	-0.697662	4.758058	0.754961
H	1.335443	1.855547	3.966290
H	-0.376804	2.082071	3.519424
H	0.445762	0.537759	3.166526
H	1.005401	4.217350	-1.890204
H	0.821117	2.485908	-2.274231
H	-0.566120	3.426055	-1.647221
H	0.055528	-3.340203	2.996603
H	1.271839	-2.135158	3.518381
H	3.936662	-3.840854	1.151905
H	2.728798	-4.250390	-0.092369
H	3.742244	-2.788118	-0.271875
H	4.965803	1.293074	0.159542
H	5.508919	1.173540	-1.523939
H	4.493619	2.522466	-1.021595

Table S102. XYZ Coordinates of A_meta_VI_Me

59

scf done: -3799.683678

C	1.072376	-1.402472	0.845621
C	1.757059	-0.112751	0.758583
C	2.143055	0.547471	1.995586
C	1.795872	0.023579	3.205817
C	1.102083	-1.228805	3.288036
C	0.780477	-1.944667	2.163954
P	2.858587	0.199296	-0.625434
O	3.396844	1.586041	-0.739564
Ni	-0.117863	-0.018603	0.151515
P	-1.750200	-1.244577	-0.400471
O	-1.294675	-2.673392	-1.048751
C	-2.067979	-3.891173	-1.105554
C	0.136994	-3.304034	2.248528
O	2.110928	-0.214010	-2.011693
C	1.999093	-1.553903	-2.537800
O	4.008921	-0.941751	-0.427484
C	5.242115	-0.829277	-1.169756
P	-0.740522	2.012673	0.061792
O	-1.571428	2.372620	1.427909
C	-2.765221	3.178326	1.493638
O	-1.840481	2.564109	-1.037116
C	-1.498813	2.407434	-2.425891
O	0.426107	3.160284	-0.022615
C	0.144994	4.568186	0.094454
O	-2.777066	-0.576407	-1.494967
C	-3.908767	-1.263984	-2.063090
O	-2.774451	-1.822664	0.758087
C	-3.504911	-0.865312	1.548055
H	0.876166	-1.642937	4.267691
H	1.239521	-2.152615	0.074219
H	2.707421	1.472904	1.931995
H	2.073504	0.537586	4.121872
H	-3.937859	-1.412720	2.386020
H	-2.844357	-0.079627	1.927907
H	-4.304964	-0.408847	0.957588
H	-2.705081	-3.897916	-1.993373
H	-1.349966	-4.710121	-1.171948
H	-2.679220	-4.009076	-0.210154
H	-4.601251	-0.498773	-2.417488
H	-3.589898	-1.880526	-2.908053
H	-4.409633	-1.887534	-1.318039
H	-2.319833	2.835999	-3.002488
H	-0.573416	2.941600	-2.662453
H	-1.394400	1.348557	-2.678916
H	1.058387	5.096775	-0.181783
H	-0.665333	4.865259	-0.576706
H	-0.119746	4.818299	1.125964

H	-2.572480	4.204960	1.173471
H	-3.556346	2.749878	0.875172
H	-3.073159	3.176990	2.540179
H	2.131118	-1.484082	-3.619192
H	2.766104	-2.207541	-2.118276
H	1.007411	-1.954545	-2.315344
H	5.923703	-1.571928	-0.753923
H	5.078748	-1.039892	-2.231088
H	5.666943	0.170708	-1.058044
H	-0.863109	-3.288545	1.801604
H	0.720827	-4.051666	1.696701
H	0.044237	-3.640958	3.284641

Table S103. XYZ Coordinates of A_meta_I_OMe

49

scf done: -5799.694772

C	0.586859	-0.526770	-0.807051
C	0.056193	-0.474769	0.452470
C	0.905318	-0.112364	1.556137
C	2.264957	0.334797	1.314194
C	2.732369	0.313237	-0.056466
C	1.935785	-0.136744	-1.082769
Ni	2.105818	-1.251831	2.457215
P	0.993033	-2.835372	3.342835
O	0.562666	-2.735682	4.922773
C	-0.625388	-2.035801	5.342955
Br	-0.053122	0.920605	3.011414
O	3.984886	0.830490	-0.208410
C	4.512387	0.932335	-1.528821
P	4.174735	-1.623123	2.865510
O	5.070211	-2.223864	1.631431
C	4.757823	-3.538446	1.133005
O	4.712336	-2.662859	4.028227
C	4.195653	-2.520746	5.362330
O	5.002954	-0.259038	3.192389
C	6.432795	-0.262139	3.399873
O	-0.461576	-3.326425	2.737684
C	-0.474791	-3.823376	1.386411
O	1.859489	-4.219665	3.382880
C	1.364598	-5.410842	4.032530
H	6.691670	0.727275	3.778035
H	2.289311	-0.150599	-2.105982
H	2.697956	1.124434	1.923917
H	-0.977552	-0.745772	0.636983
H	-0.031674	-0.867400	-1.633151
H	4.656027	-3.307331	5.962016
H	4.462875	-1.545829	5.782631
H	3.109282	-2.643139	5.381492
H	5.360689	-3.684626	0.236050
H	5.008773	-4.300278	1.875518

H	3.697251	-3.620854	0.874130
H	6.718363	-1.023501	4.129308
H	6.950473	-0.445860	2.455755
H	-0.590418	-1.995739	6.432518
H	-0.647699	-1.021281	4.937800
H	-1.517485	-2.579237	5.023071
H	2.005508	-6.230352	3.705662
H	1.425118	-5.300405	5.117284
H	0.330420	-5.614848	3.744504
H	0.038837	-3.136964	0.707727
H	-0.007940	-4.811532	1.334692
H	-1.521234	-3.902606	1.088337
H	5.495853	1.391887	-1.426912
H	4.618588	-0.055078	-1.992593
H	3.877632	1.561323	-2.163534

Table S104. XYZ Coordinates of A_meta_TS1_OMe

49

scf done: -5799.690662

C	0.026355	-0.028119	-0.024778
C	0.034392	-0.037221	1.387467
C	1.237544	-0.050991	2.151241
C	2.443132	-0.344551	1.439433
C	2.437352	-0.468109	0.057676
C	1.226637	-0.278282	-0.656842
Ni	-0.466350	-1.197229	2.708314
P	0.012675	-1.707504	4.741975
O	-1.115820	-2.325090	5.773704
C	-2.436206	-1.754905	5.742422
Br	-1.475620	1.127293	2.464720
O	3.556593	-0.387220	2.219812
P	-1.701924	-2.660776	1.790681
O	-1.325334	-3.258269	0.293674
C	0.012412	-3.737029	0.080257
O	-1.864966	-4.017710	2.692598
C	-2.690029	-5.123251	2.266962
O	-3.298077	-2.378645	1.496718
C	-3.675423	-1.363194	0.549723
O	0.606300	-0.419940	5.550470
C	1.048991	-0.512008	6.922277
O	1.214733	-2.776943	5.046192
C	1.052930	-4.153174	4.654796
H	1.228893	0.509815	7.257852
H	3.350517	-0.656730	-0.492015
H	1.356888	0.476419	3.089652
H	-0.901310	0.050391	-0.578942
H	1.244093	-0.374837	-1.739061
H	-3.089521	-2.434945	6.290464
H	-2.444650	-0.773070	6.226092
H	-2.798888	-1.652902	4.714134

H	2.026624	-4.628755	4.778701
H	0.317262	-4.647746	5.294415
H	0.735806	-4.232925	3.611139
H	0.280594	-0.975475	7.545684
H	1.972487	-1.091950	6.984415
H	-4.765292	-1.311089	0.563765
H	-3.263700	-0.391393	0.837057
H	-3.337720	-1.628793	-0.456066
H	-2.450572	-5.959661	2.924922
H	-3.747452	-4.866705	2.362809
H	-2.473218	-5.395527	1.231153
H	0.756934	-2.993031	0.381061
H	0.182661	-4.666899	0.632744
H	0.113322	-3.930288	-0.988980
C	4.824648	-0.533810	1.580699
H	5.567239	-0.509076	2.378305
H	4.894036	-1.489181	1.048909
H	5.012448	0.286247	0.878706

Table S105. XYZ Coordinates of A_meta_IIa_OMe

49

scf done: -5799.740135

C	0.096048	0.038630	0.062248
C	0.157973	0.044833	1.467520
C	1.395937	0.002201	2.115266
C	2.558055	-0.046095	1.333734
C	2.494223	-0.056402	-0.057471
C	1.251400	-0.024582	-0.720810
H	1.469981	0.008290	3.195967
Ni	1.116425	-0.251175	-2.604850
P	0.863115	1.840766	-2.684287
O	1.087858	2.579322	-4.119738
C	2.407633	2.641376	-4.693898
Br	1.611948	-2.520279	-2.288257
P	0.815223	-0.700183	-4.789176
O	0.259572	0.385509	-5.881541
C	-1.081948	0.891292	-5.736955
O	-0.223284	-1.935839	-4.975098
C	-0.552656	-2.530189	-6.249637
O	2.233450	-1.048174	-5.490077
C	2.502449	-1.067675	-6.912645
O	-0.669303	2.257893	-2.375951
C	-1.162076	3.606568	-2.586670
O	1.803511	2.826964	-1.796515
C	1.572951	3.163998	-0.411272
H	3.524424	-0.080443	1.831305
H	3.417597	-0.105646	-0.628486
H	-0.892293	0.084430	-0.388572
O	-1.051492	0.099844	2.109482
H	1.829618	4.219538	-0.301962

H	0.528290	3.009317	-0.137432
H	2.210036	2.550952	0.225491
H	3.587649	-1.046688	-7.012939
H	2.113531	-1.985302	-7.359302
H	2.064557	-0.196951	-7.401059
H	-1.176520	1.723668	-6.434184
H	-1.814598	0.119324	-5.986148
H	-1.261416	1.252142	-4.721268
H	-1.545367	-2.968997	-6.144401
H	-0.566145	-1.784434	-7.047807
H	0.170332	-3.315574	-6.484148
H	-2.124010	3.658660	-2.077640
H	-0.477534	4.344043	-2.162963
H	-1.288577	3.791537	-3.654335
H	2.300383	3.127710	-5.663265
H	3.074006	3.226336	-4.056326
H	2.818640	1.637823	-4.840579
C	-1.054703	0.102637	3.533083
H	-2.102570	0.144639	3.833033
H	-0.599075	-0.808339	3.939317
H	-0.528421	0.976118	3.936500

Table S106. XYZ Coordinates of A_meta_IIb_OMe

49

scf done: -5799.749261

C	-0.199524	-0.034901	0.052247
C	-0.084268	0.028837	1.454560
C	1.204018	0.066126	1.997144
C	2.348476	0.033196	1.184810
C	2.220348	-0.033806	-0.207112
C	0.934883	-0.063774	-0.757736
Ni	-1.608335	0.081676	2.561824
Br	-3.555349	0.209138	3.880371
H	3.088360	-0.058801	-0.854685
P	-1.485924	2.260522	2.642117
O	-0.220134	2.923120	1.868620
C	-0.044518	4.356851	1.812092
O	-2.769217	3.091615	2.079079
C	-3.275777	2.735056	0.776081
O	-1.472129	2.981503	4.103841
C	-0.466715	2.547377	5.042989
P	-1.778916	-2.065735	2.231706
O	-0.469168	-2.993040	1.949997
C	0.467104	-3.169818	3.032548
O	-2.525585	-2.477553	0.843111
C	-3.809765	-1.893145	0.539142
O	-2.558414	-2.871270	3.408745
C	-2.889959	-4.272818	3.273671
H	-3.758381	1.754104	0.808027
O	3.545779	0.077931	1.850533

H	1.364329	0.147223	3.069831
H	-1.180359	-0.063508	-0.415829
H	0.826388	-0.112355	-1.838609
H	-4.013101	3.494343	0.515571
H	-2.476935	2.731151	0.028915
H	0.907634	4.528058	1.310009
H	-0.853307	4.819652	1.242099
H	-0.015617	4.783173	2.817396
H	-0.592464	3.162517	5.933850
H	-0.608915	1.494606	5.305491
H	0.537916	2.694383	4.636080
H	-3.222228	-4.604282	4.257402
H	-3.696300	-4.402485	2.548114
H	-2.020693	-4.855266	2.960329
H	-4.088503	-2.266286	-0.446149
H	-4.559640	-2.196353	1.274674
H	-3.746987	-0.802332	0.515281
H	1.248554	-3.830049	2.656278
H	0.910579	-2.214030	3.320503
H	-0.016726	-3.632627	3.896765
C	4.742710	0.055656	1.080042
H	5.564093	0.100325	1.796365
H	4.827201	-0.865645	0.491326
H	4.804951	0.917984	0.405442

Table S107. XYZ Coordinates of A_meta_III_OMe

49

scf done: -5799.684950

C	-0.079858	-0.019336	-0.014820
C	-0.080451	-0.019788	1.380712
C	1.141748	-0.004809	2.072013
C	2.338579	-0.000821	1.355228
C	2.329740	-0.009815	-0.051035
C	1.112208	-0.015785	-0.744042
Ni	1.172546	0.022154	3.910053
P	0.456451	-2.035880	4.111650
O	-1.120366	-2.145541	3.819419
C	-1.884562	-3.350025	4.101805
H	1.082033	-0.015900	-1.826423
P	1.826769	2.124678	4.007270
O	0.875593	3.281514	3.416736
C	0.706430	3.454950	1.980511
O	3.313610	2.344462	3.427013
C	4.021710	3.614907	3.534122
O	1.831716	2.699209	5.530284
C	2.569653	1.973118	6.542046
O	1.174184	-3.283490	3.366845
C	1.185639	-3.423925	1.924414
O	0.552384	-2.552822	5.646663
C	1.837057	-2.902638	6.216029

Br	2.587910	6.787487	1.670890
H	1.658035	-3.101108	7.271996
O	3.557992	-0.002273	-0.644241
H	3.302230	0.028027	1.853628
H	-1.024316	-0.033866	1.915918
H	-1.025319	-0.024789	-0.550505
H	5.018004	3.429385	3.134354
H	3.520241	4.394499	2.953290
H	4.092166	3.914693	4.581710
H	2.614515	2.627335	7.412143
H	2.055153	1.046701	6.813549
H	3.584878	1.750528	6.203018
H	-0.346897	3.294305	1.747284
H	1.014432	4.476468	1.745782
H	1.315571	2.745031	1.418961
H	1.488534	-4.451612	1.725411
H	0.190464	-3.246296	1.511578
H	1.899890	-2.728444	1.482767
H	2.233707	-3.794596	5.728428
H	2.546394	-2.075896	6.116760
H	-2.848229	-3.215383	3.612814
H	-1.379475	-4.228809	3.696648
H	-2.017103	-3.456626	5.179195
C	3.619002	0.026239	-2.069034
H	4.679130	0.040524	-2.322855
H	3.152926	-0.862916	-2.508690
H	3.137333	0.924128	-2.472563

Table S108. XYZ Coordinates of A_meta_IV_OMe

65

scf done: -6486.523115

C	-0.869253	1.930829	2.016132
C	-0.796009	3.236666	2.501016
C	-0.431778	4.300084	1.669227
C	-0.140837	4.031066	0.327707
C	-0.222974	2.715777	-0.162107
C	-0.584554	1.649976	0.661854
H	-0.377617	5.305194	2.068968
Ni	-0.779261	-0.199537	0.193503
P	-1.305582	-2.347077	0.231567
O	-0.515550	-3.294600	1.290679
C	0.359446	-4.384674	0.932404
P	1.280036	-0.423759	0.849304
O	2.362212	0.726255	0.503871
C	2.642652	1.067166	-0.878796
P	-2.532040	0.431375	-0.926945
O	-3.680604	1.344024	-0.228707
C	-4.325396	0.852600	0.970633
O	1.917904	-1.788627	0.252678
C	3.262336	-2.228640	0.594353

O	1.458919	-0.532495	2.452252
C	2.206342	0.395741	3.275578
O	-3.331510	-0.811511	-1.601671
C	-4.378696	-0.599993	-2.578024
O	-2.135465	1.350870	-2.193719
C	-2.735825	2.623870	-2.532592
O	-1.211631	-3.092985	-1.200350
C	-2.076234	-4.169459	-1.631390
O	-2.820508	-2.467842	0.799048
C	-3.343311	-3.590778	1.551157
Br	6.160528	-0.480799	-1.391729
O	0.227074	4.976416	-0.589512
H	0.001569	2.564088	-1.212645
H	-1.139940	1.133571	2.704571
H	-1.020510	3.437070	3.545471
H	-4.803533	-1.580708	-2.789644
H	-5.152715	0.058339	-2.176400
H	-3.959150	-0.172509	-3.491376
H	-5.213374	1.467685	1.114150
H	-4.614170	-0.194438	0.854339
H	-3.656099	0.964970	1.826917
H	-2.269777	2.927703	-3.469728
H	-3.813736	2.523718	-2.668855
H	-2.522682	3.359080	-1.755299
H	-4.274534	-3.242969	1.997356
H	-3.547495	-4.433351	0.886767
H	-2.641364	-3.891120	2.329158
H	-1.829726	-4.357688	-2.675815
H	-1.895125	-5.074798	-1.046406
H	-3.120567	-3.862980	-1.548134
H	1.129075	-4.436195	1.702419
H	-0.209984	-5.317818	0.920285
H	0.821955	-4.216098	-0.040004
H	3.319212	-3.274146	0.291472
H	4.013034	-1.643340	0.055811
H	3.416165	-2.154006	1.673331
H	2.182714	-0.019279	4.283132
H	3.236589	0.478052	2.928419
H	1.726017	1.375734	3.264898
H	2.618227	2.154824	-0.951879
H	3.634659	0.684599	-1.134610
H	1.893476	0.647980	-1.556867
C	0.330466	6.330080	-0.155852
H	0.633728	6.904291	-1.031886
H	-0.630464	6.709697	0.210799
H	1.085796	6.443831	0.630490

Table S109. XYZ Coordinates of A_meta_TS2_OMe

65			
scf done: -6486.444621			
Br	-4.770159	-1.283595	1.162640
C	-2.747393	-2.951249	1.862009
O	-1.746514	-1.278783	0.824671
P	-0.800518	-1.417962	-0.385016
O	-1.607646	-1.635871	-1.800631
C	-2.690507	-2.591678	-1.874946
Ni	0.435209	0.384349	-0.325656
P	-1.189458	1.793637	-0.860838
O	-0.447351	3.023833	-1.612570
C	-1.188575	4.102577	-2.228260
C	2.015729	-0.663691	-0.506553
C	2.702145	-1.412623	0.449128
C	3.874116	-2.120125	0.130067
C	4.382537	-2.087489	-1.173403
C	3.699161	-1.338643	-2.138821
C	2.537421	-0.637566	-1.819313
H	5.283550	-2.624803	-1.443063
P	1.697488	1.875220	0.613886
O	2.499184	1.401193	1.955534
C	1.738355	0.906070	3.076270
O	0.869366	3.179117	1.115155
C	1.526615	4.305302	1.743715
O	2.910331	2.535151	-0.230516
C	4.281702	2.074912	-0.188708
O	-0.140830	-2.916976	-0.150890
C	0.561228	-3.607258	-1.196668
O	-2.174977	2.502134	0.219616
C	-2.331254	2.009843	1.573228
O	-2.221414	1.374922	-2.047178
C	-3.556093	0.898581	-1.767262
O	4.445664	-2.809175	1.165647
H	2.352600	-1.481956	1.474630
H	2.040358	-0.066995	-2.601214
H	4.086242	-1.309429	-3.154296
H	-3.582291	0.214476	-0.917223
H	-4.214687	1.749173	-1.574098
H	-3.886358	0.375391	-2.665207
H	-0.448324	4.752830	-2.693902
H	-1.873668	3.713027	-2.983216
H	-1.744144	4.658131	-1.468935
H	-1.356695	1.870230	2.045580
H	-2.881244	2.785027	2.108117
H	-2.893974	1.073961	1.575388
H	-2.318938	-3.612797	-1.745582
H	-3.443793	-2.371140	-1.113537
H	-3.124587	-2.490676	-2.870984
H	0.081549	-3.450613	-2.166527

H	1.599654	-3.275417	-1.245461
H	0.529398	-4.670977	-0.950193
H	4.359106	1.069979	-0.605970
H	4.848254	2.775995	-0.801959
H	4.655639	2.090928	0.835749
H	1.064164	0.098714	2.773983
H	2.460868	0.520731	3.795507
H	1.159092	1.712683	3.533557
H	0.732977	4.951903	2.117141
H	2.157032	3.974416	2.572168
H	2.132223	4.840041	1.009487
H	-2.704420	-2.432639	2.807217
H	-3.645330	-3.507400	1.648694
H	-1.826264	-3.327002	1.449295
C	5.634984	-3.548596	0.905338
H	5.914467	-4.013291	1.851581
H	5.467500	-4.330004	0.154626
H	6.449543	-2.896473	0.568523

Table S110. XYZ Coordinates of A_meta_V_OMe

60

scf done: -3874.889294

C	2.683921	-0.035322	0.348343
C	1.555311	0.096475	-0.463226
C	1.767630	0.471178	-1.809856
C	3.054630	0.689116	-2.301338
C	4.177206	0.558526	-1.476979
C	3.980066	0.193902	-0.140974
Ni	-0.292104	-0.154701	-0.031039
P	-0.379501	1.999563	0.217222
O	-1.662576	2.455824	1.117870
C	-1.961236	3.851315	1.356122
O	4.991261	0.025671	0.770801
C	6.329488	0.231565	0.332677
P	0.110979	-2.295405	0.313498
O	-1.057850	-3.250289	0.469315
P	-2.464843	-0.449677	-0.282948
O	-3.499762	-0.884060	0.893519
C	-3.043744	-1.395069	2.169779
O	1.011187	-2.274017	1.702326
C	1.258466	-3.515915	2.381159
O	1.243079	-3.001776	-0.684396
C	0.802626	-3.365882	-1.995652
O	-2.659565	-1.507107	-1.500456
C	-3.958655	-1.757607	-2.079906
O	-3.271469	0.825270	-0.913128
C	-4.352004	1.512154	-0.255079
O	-0.473685	2.864517	-1.148609
C	0.437656	3.916981	-1.541226
O	0.857179	2.769844	0.954620

C	1.154389	2.418511	2.321272
H	5.167985	0.737331	-1.876853
H	2.595103	-0.356030	1.380524
H	0.926614	0.602179	-2.487387
H	3.194605	0.971301	-3.342178
H	-3.808466	-2.508746	-2.855272
H	-4.645458	-2.143611	-1.322285
H	-4.363711	-0.843703	-2.519560
H	-4.504140	2.438149	-0.811368
H	-5.264087	0.911379	-0.286980
H	-4.098983	1.744411	0.779687
H	-2.451009	-0.638036	2.691499
H	-3.945794	-1.605384	2.745209
H	-2.455322	-2.300757	2.020400
H	1.619996	-3.911183	-2.472965
H	-0.083727	-4.007002	-1.954291
H	0.575888	-2.477572	-2.598623
H	1.654501	-3.268250	3.367968
H	0.335346	-4.092054	2.487911
H	1.995160	-4.113700	1.834678
H	0.031118	4.332436	-2.463504
H	0.494511	4.693179	-0.776978
H	1.429732	3.500938	-1.726493
H	1.269395	1.337455	2.432961
H	2.096584	2.906937	2.569533
H	0.367158	2.775725	2.990270
H	-2.798462	3.874046	2.053353
H	-1.102046	4.365104	1.793452
H	-2.245529	4.337606	0.419944
H	6.964184	0.044796	1.200006
H	6.603539	-0.462815	-0.470794
H	6.487799	1.259686	-0.014951

Table S111. XYZ Coordinates of A_meta_TS3_OMe

60

scf done: -3874.847088

C	-1.845775	0.056966	-2.883936
C	-3.078458	-0.442565	-2.462532
C	-3.459637	-0.311121	-1.114792
C	-2.592922	0.297449	-0.203987
C	-1.326341	0.757865	-0.614403
C	-0.981051	0.687345	-1.987343
O	-4.640711	-0.758971	-0.601302
C	-5.559327	-1.405947	-1.481003
Ni	0.351414	0.016602	0.097288
P	-0.161005	-1.672882	1.299763
O	-0.761983	-3.028128	0.583675
C	-0.619328	-3.215968	-0.835609
P	-0.437409	2.169897	0.312886
O	-1.675552	2.605599	1.309158

C	-2.733511	3.433302	0.804399
P	2.310563	0.116791	-0.665591
O	3.459426	0.046983	0.502521
C	4.824450	-0.392513	0.346961
O	0.650986	1.944143	1.540232
C	0.643388	2.751709	2.743638
O	2.839438	-1.155741	-1.586498
C	2.265872	-1.336223	-2.891808
O	2.721492	1.396662	-1.618658
C	4.024658	1.596100	-2.199245
O	-0.061306	3.267502	-0.636829
O	0.991354	-2.312822	2.303948
C	2.108598	-2.993319	1.713265
O	-1.350681	-1.586052	2.434952
C	-1.179033	-0.685054	3.540725
H	1.585526	2.535908	3.249394
H	-3.739786	-0.909308	-3.182452
H	-2.891604	0.366988	0.834319
H	-0.076304	1.160556	-2.344840
H	-1.574875	-0.018455	-3.932661
H	5.148456	-0.754029	1.324659
H	4.897324	-1.197602	-0.385360
H	5.462886	0.441673	0.043837
H	2.741571	-2.216023	-3.327427
H	1.186313	-1.503559	-2.826965
H	2.454817	-0.467762	-3.529193
H	3.893076	2.245247	-3.066849
H	4.684775	2.089250	-1.479918
H	4.471789	0.651537	-2.519479
H	2.875663	-3.084032	2.484223
H	1.809937	-3.992325	1.380052
H	2.520322	-2.435456	0.867359
H	-2.092568	-0.741082	4.134511
H	-0.325335	-0.982623	4.156094
H	-1.041963	0.343525	3.194637
H	-1.134065	-4.148031	-1.074358
H	-1.078841	-2.398684	-1.397782
H	0.434587	-3.306018	-1.115443
H	0.592550	3.814403	2.496764
H	-0.197573	2.488252	3.387061
H	-3.365578	3.686623	1.657048
H	-2.331509	4.345351	0.356557
H	-3.332325	2.897308	0.060395
H	-6.413611	-1.687232	-0.865162
H	-5.893249	-0.731647	-2.277760
H	-5.121416	-2.306285	-1.926795

Table S112. XYZ Coordinates of A_meta_VI_OMe

60

scf done: -3874.895782

C	-0.713084	-1.595412	-0.355216
C	-1.598341	-0.463718	-0.624959
C	-2.077659	-0.269730	-1.987653
C	-1.659963	-1.085236	-2.995079
C	-0.787955	-2.199620	-2.749538
C	-0.369619	-2.459651	-1.470862
Ni	0.225599	0.105337	-0.148133
P	2.015466	-0.551863	0.765841
O	1.849046	-1.575563	2.041353
C	1.924993	-3.007857	1.901077
P	-2.728260	0.091452	0.654848
O	-3.321909	1.448493	0.467213
O	0.377956	-3.541794	-1.098798
C	0.752953	-4.479236	-2.105020
P	0.359172	2.191374	-0.570285
O	1.661407	2.677891	-1.449512
C	2.920813	3.011336	-0.839295
O	0.432730	3.336980	0.609051
C	-0.460856	3.221091	1.735123
O	-0.793230	2.730372	-1.592493
C	-0.778048	4.075439	-2.112198
O	-1.973869	0.026115	2.094012
C	-1.788635	-1.156564	2.899266
O	-3.826996	-1.112955	0.709347
C	-5.057780	-0.909448	1.436070
O	2.835011	0.633890	1.550093
C	3.977783	0.344124	2.384424
O	3.175839	-1.378162	-0.059350
C	3.400842	-1.072606	-1.447603
H	-0.515973	-2.848127	-3.573072
H	-0.773794	-2.118377	0.596382
H	-2.757751	0.551283	-2.185640
H	-2.006955	-0.914389	-4.010386
H	3.863431	-0.087725	-1.561887
H	4.082083	-1.834924	-1.828079
H	2.466791	-1.105838	-2.016759
H	1.493926	-3.428351	2.811335
H	1.365599	-3.357910	1.031409
H	2.967853	-3.322393	1.812754
H	4.404506	1.306525	2.669122
H	3.668052	-0.205401	3.275439
H	4.720237	-0.237973	1.832450
H	-0.285446	4.098346	2.359518
H	-1.503352	3.198687	1.407911
H	-0.249408	2.314982	2.310317
H	-1.727316	4.217534	-2.630446
H	-0.691233	4.802954	-1.300911

H	0.050645	4.207340	-2.811194
H	3.628222	3.163835	-1.656200
H	2.829990	3.932282	-0.258043
H	3.274483	2.207514	-0.189618
H	-2.098505	-0.911016	3.917363
H	-2.390166	-1.988957	2.528992
H	-0.729753	-1.424120	2.893387
H	-5.685661	-1.777127	1.231642
H	-4.869351	-0.842628	2.511847
H	-5.557874	0.000239	1.096219
H	1.320279	-5.258736	-1.596071
H	-0.128409	-4.920502	-2.584207
H	1.381895	-4.010523	-2.870468

Table S113. XYZ Coordinates of H_mono_I

63

scf done: -4692.072337

C	0.991295	3.306368	1.254854
C	0.381008	3.281548	2.511364
C	-0.973774	3.600090	2.646801
C	-1.723866	3.948264	1.518645
C	-1.127816	3.976641	0.253126
C	0.222022	3.651648	0.144605
H	-1.440355	3.586622	3.626710
Br	1.046956	3.685997	-1.591558
O	-2.772581	0.663382	1.698697
P	-2.303890	-0.415123	0.508950
C	-3.430641	0.027317	-0.878204
C	-4.491710	0.929459	-0.713731
C	-5.320440	1.245795	-1.793677
C	-5.098759	0.663298	-3.043492
C	-4.039292	-0.232803	-3.215985
C	-3.204767	-0.542832	-2.141675
Pd	-0.059580	-0.498129	0.002473
P	2.186268	-0.558729	-0.504671
C	2.843406	-2.085671	-1.285497
C	4.219825	-2.350727	-1.373623
C	4.674544	-3.504111	-2.012820
C	3.760694	-4.407612	-2.564659
C	2.390256	-4.154771	-2.478205
C	1.933688	-2.998414	-1.841301
C	3.335129	-0.281006	0.908057
C	3.108241	-0.964719	2.113868
C	3.954034	-0.770291	3.206137
C	5.026618	0.122214	3.111496
C	5.250424	0.815802	1.920460
C	4.410198	0.615248	0.821289
O	2.730994	0.558609	-1.620784
C	-3.062700	-1.939000	1.200509
C	-4.452523	-2.133605	1.253215

C	-4.979679	-3.291788	1.824933
C	-4.125954	-4.270026	2.344345
C	-2.742577	-4.087541	2.292746
C	-2.213653	-2.927214	1.722887
H	-2.167300	1.422394	1.717341
H	2.172422	1.354572	-1.593373
H	2.040620	3.058196	1.144915
H	4.936006	-1.659751	-0.939147
H	0.867620	-2.796675	-1.768910
H	5.740737	-3.700988	-2.077200
H	1.677786	-4.855761	-2.903120
H	4.117646	-5.307387	-3.057293
H	4.583146	1.153987	-0.104798
H	2.263441	-1.644468	2.199236
H	6.079552	1.513500	1.844095
H	3.771762	-1.307023	4.132688
H	5.679974	0.279855	3.964627
H	-5.123311	-1.384662	0.843325
H	-1.137241	-2.780484	1.676203
H	-6.055848	-3.433796	1.861666
H	-2.076236	-4.846577	2.692016
H	-4.539438	-5.173002	2.784083
H	-4.660581	1.384918	0.256804
H	-2.368674	-1.223774	-2.284460
H	-6.138564	1.947324	-1.657376
H	-3.857430	-0.681244	-4.188394
H	-5.743248	0.911313	-3.881760
H	0.970325	3.015673	3.383351
H	-1.704409	4.246208	-0.624111
H	-2.773517	4.207521	1.617338

Table S114. XYZ Coordinates of H_mono_TS1

63

scf done: -4692.041611

C	-2.447254	3.029795	-1.458362
C	-2.502841	2.021803	-0.485521
C	-2.724012	2.379847	0.855792
C	-2.898647	3.716792	1.211328
C	-2.847666	4.715939	0.233711
C	-2.621691	4.369760	-1.099270
P	-2.180252	0.258750	-0.908461
C	-3.772785	-0.551756	-0.447113
C	-3.743746	-1.905558	-0.074066
C	-4.926110	-2.582599	0.232375
C	-6.150264	-1.913304	0.170713
C	-6.190912	-0.566045	-0.199884
C	-5.010535	0.112065	-0.506532
Pd	-0.130420	-0.669326	-0.091829
Br	0.069901	-3.390842	-0.188912
C	0.750401	-1.977231	1.359679

C	2.118760	-2.045498	1.687640
C	2.508456	-1.756996	2.990401
C	1.557091	-1.449141	3.979674
C	0.202829	-1.458105	3.660911
C	-0.221200	-1.772552	2.357871
H	1.878646	-1.239204	4.994961
P	1.670889	0.697450	-0.808175
O	1.203795	1.553559	-2.167636
C	3.279666	-0.035922	-1.340644
C	3.275573	-1.370036	-1.781174
C	4.444121	-1.962932	-2.264012
C	5.631380	-1.228788	-2.310909
C	5.647452	0.099179	-1.875312
C	4.480161	0.692892	-1.392449
C	2.193041	2.048682	0.331819
C	1.704157	3.352913	0.155109
C	2.020707	4.359104	1.071427
C	2.821140	4.074592	2.179220
C	3.305922	2.776773	2.367978
C	2.993824	1.770309	1.453841
O	-2.234073	0.256638	-2.577336
H	3.566111	-1.768210	3.239847
H	2.854500	-2.276566	0.925948
H	-1.276489	-1.890326	2.138192
H	-0.544023	-1.272054	4.427842
H	-2.766762	1.612843	1.625232
H	-2.265167	2.769921	-2.496302
H	-3.075045	3.979299	2.250401
H	-2.580673	5.140396	-1.863677
H	-2.981728	5.757120	0.511772
H	3.378726	0.767672	1.613261
H	1.075641	3.582037	-0.699626
H	3.931200	2.548259	3.226304
H	1.640109	5.364744	0.917256
H	3.065965	4.857052	2.891418
H	-2.790981	-2.426037	-0.023725
H	-4.890235	-3.628822	0.521639
H	-7.069989	-2.437600	0.413249
H	-7.141326	-0.042408	-0.246320
H	-5.054415	1.161026	-0.783871
H	-3.093846	0.537839	-2.930029
H	4.507586	1.723314	-1.051296
H	6.569286	0.672550	-1.910085
H	6.541910	-1.689603	-2.682644
H	4.427793	-2.996233	-2.598094
H	2.354892	-1.946093	-1.734722
H	1.911679	2.119082	-2.517004

Table S115. XYZ Coordinates of H_mono_IIa

63

scf done: -4692.115961

C	3.102362	1.385946	-0.725205
C	2.303662	0.596074	-1.566516
C	2.071356	1.019999	-2.886602
C	2.638369	2.205138	-3.356497
C	3.437740	2.982968	-2.514854
C	3.667464	2.571034	-1.200713
P	1.478969	-0.927431	-0.971583
C	2.695034	-1.728787	0.140384
C	4.066138	-1.769050	-0.166471
C	4.952225	-2.452707	0.665245
C	4.480370	-3.104090	1.809434
C	3.119325	-3.075792	2.118786
C	2.229809	-2.393372	1.285807
Pd	-0.823612	-0.920215	-0.159099
C	-2.805264	-1.073301	0.381191
C	-3.182004	-1.764150	1.547642
C	-4.534757	-1.939075	1.875850
C	-5.531267	-1.425708	1.044435
C	-5.169052	-0.735092	-0.115582
C	-3.819853	-0.555771	-0.440478
H	-6.578993	-1.559821	1.297638
P	-0.930439	1.055757	1.002114
O	-1.865458	0.975671	2.357276
C	-1.664679	2.407615	0.014489
C	-1.353270	2.531111	-1.348791
C	-1.908680	3.566211	-2.102216
C	-2.786220	4.474699	-1.504364
C	-3.106360	4.349736	-0.150175
C	-2.547635	3.321232	0.610544
C	0.577238	1.753295	1.768291
C	1.326180	0.930126	2.626561
C	2.453668	1.431839	3.275182
C	2.846067	2.758555	3.070419
C	2.106697	3.580422	2.218315
C	0.975661	3.082097	1.567443
Br	-1.140530	-3.164282	-1.378216
O	1.546024	-1.810387	-2.366080
H	4.441640	-1.263447	-1.050714
H	-2.546270	0.284695	2.244517
H	0.772841	-2.419576	-2.362865
H	-4.802105	-2.480969	2.779500
H	-2.422882	-2.189984	2.200352
H	-3.562954	-0.007731	-1.343362
H	-5.937267	-0.328617	-0.768785
H	1.169192	-2.379451	1.521364
H	6.010290	-2.477335	0.422134
H	2.749321	-3.584018	3.004154

H	5.173545	-3.633725	2.456263
H	1.465375	0.411176	-3.549145
H	3.294541	1.077789	0.297057
H	2.459627	2.516852	-4.381347
H	4.288300	3.169751	-0.541272
H	3.880653	3.903942	-2.882261
H	-2.799271	3.220538	1.661113
H	-0.680428	1.822107	-1.822190
H	-3.791844	5.051743	0.314972
H	-1.662317	3.657774	-3.155696
H	-3.223265	5.275117	-2.093849
H	1.028817	-0.100979	2.790332
H	0.406882	3.730567	0.910372
H	3.025041	0.788819	3.937609
H	2.406664	4.611623	2.058773
H	3.725168	3.148550	3.574608

Table S116. XYZ Coordinates of H_mono_IIb

63

scf done: -4692.117551

C	3.352939	1.644832	-1.381625
C	3.076127	1.640856	-0.002916
C	3.383341	2.773786	0.764891
C	3.974464	3.889079	0.165673
C	4.257791	3.883652	-1.200943
C	3.945501	2.760139	-1.972841
P	2.241313	0.196622	0.756989
Pd	0.079797	-0.158452	-0.112035
Br	1.017258	-1.322502	-2.259471
C	3.431103	-1.188870	0.621762
C	2.944566	-2.497560	0.779222
C	3.823023	-3.580384	0.774644
C	5.193956	-3.367085	0.604801
C	5.685176	-2.069537	0.444748
C	4.809670	-0.981877	0.455123
O	2.246395	0.537137	2.376106
P	-2.127494	-0.420531	-0.914205
C	-3.088288	-1.729912	-0.068182
C	-2.388517	-2.753464	0.591745
C	-3.077705	-3.813523	1.182728
C	-4.472316	-3.859718	1.120215
C	-5.176474	-2.847531	0.462357
C	-4.490143	-1.787286	-0.130783
C	-0.686001	0.837288	1.486170
C	-0.663031	2.240635	1.496804
C	-1.176400	2.956502	2.584048
C	-1.726419	2.281476	3.676713
C	-1.756147	0.885012	3.674041
C	-1.238738	0.167494	2.587635
H	-2.127324	2.837072	4.519870

O	-2.121052	-0.878610	-2.495189
C	-3.232538	1.034269	-0.975921
C	-3.419667	1.712460	-2.190305
C	-4.221491	2.854853	-2.240766
C	-4.839660	3.330363	-1.082703
C	-4.653592	2.661096	0.130096
C	-3.851405	1.521331	0.187701
H	-1.145229	4.043364	2.573603
H	-0.239209	2.786964	0.657950
H	-1.277551	-0.917826	2.607748
H	-2.182865	0.346849	4.517084
H	3.140996	0.521554	2.753087
H	-1.192615	-1.107540	-2.734984
H	3.159890	2.786883	1.826388
H	3.100318	0.780596	-1.988915
H	4.211576	4.760130	0.769209
H	4.161344	2.752964	-3.037092
H	4.716890	4.751467	-1.664983
H	1.877613	-2.664818	0.894277
H	5.200661	0.022640	0.329579
H	3.438191	-4.588541	0.894960
H	6.749599	-1.901908	0.310968
H	5.877192	-4.211101	0.594338
H	-2.949853	1.336940	-3.092617
H	-3.710217	1.014264	1.136673
H	-4.364862	3.368731	-3.186675
H	-5.132325	3.026406	1.033724
H	-5.464326	4.217784	-1.123825
H	-1.303463	-2.718037	0.641018
H	-5.046221	-1.005074	-0.637165
H	-2.527313	-4.598385	1.692734
H	-6.260556	-2.882937	0.411022
H	-5.009833	-4.681913	1.583242

Table S117. XYZ Coordinates of H_mono_III

63

scf done: -4692.087580

C	-4.691420	2.286195	0.039948
C	-3.436320	1.960380	-0.500966
C	-2.585199	2.992928	-0.937294
C	-2.984876	4.325353	-0.835272
C	-4.233473	4.639839	-0.292931
C	-5.084761	3.620929	0.142326
P	-2.860709	0.236250	-0.706202
O	-3.271556	-0.197202	-2.243131
Pd	-0.531507	0.048846	-0.317191
C	-0.467911	-1.749769	-1.186278
C	-0.026530	-1.869151	-2.508539
C	-0.018196	-3.127081	-3.123430
C	-0.440210	-4.260582	-2.424556

C	-0.874264	-4.134097	-1.103460
C	-0.891160	-2.880098	-0.478950
H	-0.429372	-5.234378	-2.905244
P	1.779128	0.002017	0.065300
C	2.412868	-1.231215	1.252794
C	3.747579	-1.666217	1.212174
C	4.200149	-2.602752	2.142681
C	3.333867	-3.104983	3.117758
C	2.005862	-2.674489	3.161908
C	1.542890	-1.747153	2.226630
C	2.187258	1.644575	0.769917
C	2.384263	1.841025	2.145922
C	2.609093	3.125371	2.645241
C	2.638298	4.221228	1.779636
C	2.440697	4.033189	0.408561
C	2.209604	2.753583	-0.095418
O	2.621155	-0.133267	-1.308748
C	-3.925402	-0.776477	0.378306
C	-4.741649	-1.788547	-0.145139
C	-5.502378	-2.587725	0.712018
C	-5.446986	-2.387410	2.092147
C	-4.625905	-1.386005	2.620389
C	-3.863852	-0.586223	1.769953
Br	5.669219	0.532490	-1.405585
H	0.324324	-3.214197	-4.151182
H	0.316411	-0.999744	-3.059711
H	-1.237425	-2.797250	0.546409
H	-1.204631	-5.008725	-0.549339
H	-4.180444	0.049041	-2.480785
H	3.605825	0.101782	-1.275003
H	4.431443	-1.270859	0.465099
H	0.504811	-1.426288	2.251636
H	5.232090	-2.939152	2.106980
H	1.328539	-3.066694	3.914484
H	3.692278	-3.833475	3.839008
H	2.064317	2.612372	-1.162329
H	2.375294	0.995028	2.825594
H	2.468020	4.881785	-0.268280
H	2.769505	3.267130	3.709762
H	2.816846	5.218218	2.170981
H	-4.782241	-1.956268	-1.216288
H	-3.228999	0.189331	2.190479
H	-6.135468	-3.366803	0.298427
H	-4.578764	-1.228423	3.693563
H	-6.038107	-3.010703	2.756202
H	-1.613167	2.756792	-1.362650
H	-5.358942	1.502478	0.382026
H	-2.321723	5.115100	-1.174448
H	-6.056110	3.864444	0.561726
H	-4.542361	5.677382	-0.208739

Table S118. XYZ Coordinates of H_mono_IV

88			
scf done: -5572.626270			
C	-1.609040	-3.623739	-1.009860
C	-1.592781	-2.273817	-1.389014
C	-1.620057	-1.942453	-2.753225
C	-1.666343	-2.946477	-3.720581
C	-1.680243	-4.289881	-3.336556
C	-1.651958	-4.625695	-1.981198
P	-1.480685	-0.921125	-0.156022
C	-2.311998	-1.587140	1.334606
C	-3.664248	-1.969698	1.288934
C	-4.284463	-2.473898	2.431852
C	-3.564993	-2.609184	3.623320
C	-2.223633	-2.226717	3.676003
C	-1.601099	-1.710153	2.537381
Pd	0.633210	0.035549	0.286371
P	2.909732	0.688985	0.470053
C	4.070468	-0.333468	1.448819
C	4.292455	-0.012370	2.798312
C	5.141537	-0.800453	3.577376
C	5.771948	-1.914280	3.019609
C	5.547997	-2.242913	1.680173
C	4.697695	-1.462600	0.896776
P	-0.296920	2.197498	0.917422
O	0.599574	2.971116	2.063217
C	-0.367025	3.384483	-0.480887
C	-0.797841	2.965139	-1.750292
C	-0.823676	3.869588	-2.813916
C	-0.407512	5.189786	-2.623796
C	0.030401	5.608364	-1.364699
C	0.050054	4.711585	-0.295038
C	-1.924225	2.329673	1.752094
C	-3.057771	2.847500	1.111457
C	-4.273467	2.927930	1.793060
C	-4.367249	2.493523	3.116948
C	-3.240479	1.975613	3.760967
C	-2.025080	1.888984	3.081734
C	1.446551	-1.810838	-0.224423
C	1.867315	-2.090769	-1.533442
C	2.420966	-3.334583	-1.861926
C	2.553987	-4.325953	-0.886882
C	2.134660	-4.061891	0.418786
C	1.591552	-2.814241	0.746434
H	2.980010	-5.292350	-1.141691
O	-2.426045	0.245698	-0.783103
O	3.020680	2.162608	1.258700
C	3.728750	1.002196	-1.133683
C	5.127587	1.018068	-1.268017

C	5.709927	1.347931	-2.492172
C	4.905811	1.665669	-3.589520
C	3.514653	1.658332	-3.462700
C	2.927166	1.328750	-2.241167
Br	-5.320169	-0.346704	-1.862567
H	2.745563	-3.525325	-2.881959
H	1.770528	-1.340695	-2.313088
H	1.288043	-2.632856	1.773902
H	2.232424	-4.823808	1.188277
H	-3.344374	-0.022004	-1.107379
H	1.553952	2.790418	1.915775
H	-4.235796	-1.856963	0.370791
H	-0.563012	-1.395637	2.586769
H	-5.330690	-2.762408	2.392451
H	-1.663662	-2.321656	4.601501
H	-4.051785	-3.006549	4.509123
H	-1.623032	-0.900962	-3.058615
H	-1.592796	-3.897060	0.039827
H	-1.696013	-2.679139	-4.772742
H	-1.669262	-5.668089	-1.677089
H	-1.716765	-5.071001	-4.090159
H	0.393991	5.034934	0.682035
H	-1.128645	1.942171	-1.897999
H	0.356853	6.633214	-1.214177
H	-1.165198	3.541191	-3.791176
H	-0.422034	5.889276	-3.454424
H	5.763619	0.770947	-0.424471
H	1.845030	1.326216	-2.147750
H	6.791298	1.355500	-2.587669
H	2.886979	1.906641	-4.312924
H	5.362727	1.918902	-4.541406
H	3.812646	0.855180	3.239157
H	4.524741	-1.735399	-0.138714
H	5.310965	-0.539486	4.617550
H	6.033950	-3.109426	1.242343
H	6.434357	-2.524800	3.625826
H	-2.995454	3.190471	0.084718
H	-1.153822	1.488591	3.590580
H	-5.145450	3.333709	1.288915
H	-3.306862	1.637682	4.790835
H	-5.313779	2.558967	3.645354
H	3.915650	2.535853	1.312291

Table S119. XYZ Coordinates of H_mono_V

87

scf done: -5572.173126

C	3.433952	0.919438	-2.051243
C	3.645455	1.374772	-0.740778
C	4.417298	2.528021	-0.534803
C	4.980361	3.204576	-1.618820

C	4.776735	2.737154	-2.920056
C	4.002667	1.593753	-3.134552
P	2.916971	0.524272	0.728711
O	3.021818	1.517500	1.938974
Pd	0.670627	-0.131785	0.280020
C	1.368090	-2.013684	-0.250239
C	1.566544	-2.373768	-1.594140
C	2.010850	-3.652242	-1.952908
C	2.248897	-4.615868	-0.970062
C	2.045064	-4.283219	0.371463
C	1.618953	-2.996528	0.722754
H	2.586468	-5.611522	-1.245367
P	-1.541655	-0.886375	-0.193363
O	-2.363922	0.314729	-0.930126
C	-2.465593	-1.345727	1.326746
C	-1.747041	-1.748641	2.462807
C	-2.419110	-2.126714	3.627514
C	-3.814022	-2.086424	3.671006
C	-4.534969	-1.672160	2.546977
C	-3.868869	-1.307654	1.375969
C	-1.815464	-2.323386	-1.307865
C	-1.859546	-2.109705	-2.694614
C	-2.022835	-3.184555	-3.569426
C	-2.136528	-4.484368	-3.070085
C	-2.092242	-4.704379	-1.691269
C	-1.934685	-3.630359	-0.813364
P	-0.068707	2.094368	0.951693
O	0.885799	2.726661	2.095073
C	4.161605	-0.784620	1.113096
C	4.373830	-1.108044	2.461978
C	5.298973	-2.091021	2.819755
C	6.025599	-2.761453	1.832898
C	5.824716	-2.440831	0.487698
C	4.898555	-1.459343	0.129354
C	-1.718235	2.339532	1.734972
C	-2.835242	2.813962	1.033636
C	-4.064362	2.961244	1.679724
C	-4.192285	2.636087	3.031873
C	-3.083347	2.163935	3.738867
C	-1.854559	2.014761	3.094603
C	-0.033412	3.317365	-0.423341
C	0.548730	4.576765	-0.214997
C	0.607644	5.505946	-1.255578
C	0.086777	5.186283	-2.512159
C	-0.489672	3.931711	-2.727422
C	-0.544913	2.996298	-1.691399
Br	-5.342196	0.080152	-1.976119
H	2.160395	-3.895506	-3.002571
H	1.356055	-1.659372	-2.386794
H	1.495156	-2.763267	1.777888

H	2.224451	-5.022085	1.149494
H	-3.304684	0.132805	-1.233270
H	1.826899	2.235343	2.080472
H	-4.441777	-0.990296	0.507536
H	-0.661313	-1.759781	2.436390
H	-5.620053	-1.634920	2.581096
H	-1.853480	-2.441314	4.499700
H	-4.338475	-2.372191	4.578218
H	-1.781936	-1.101558	-3.089191
H	-1.910042	-3.813540	0.256110
H	-2.064038	-3.005701	-4.639968
H	-2.187717	-5.711754	-1.296556
H	-2.263303	-5.320653	-3.751356
H	0.959912	4.817548	0.760102
H	-0.996131	2.023401	-1.860687
H	1.061343	6.478297	-1.085453
H	-0.893650	3.678417	-3.703564
H	0.133857	5.909735	-3.321025
H	4.564298	2.890284	0.478122
H	2.825579	0.037637	-2.225193
H	5.576158	4.097055	-1.448444
H	3.836409	1.229751	-4.144574
H	5.213946	3.264444	-3.763084
H	3.824035	-0.573992	3.230612
H	4.757800	-1.218798	-0.919394
H	5.455398	-2.328556	3.868396
H	6.392954	-2.951863	-0.284527
H	6.747479	-3.524492	2.109757
H	-2.750405	3.071096	-0.016070
H	-0.994995	1.657285	3.652848
H	-4.921027	3.332436	1.124651
H	-3.173322	1.913329	4.791982
H	-5.149126	2.752526	3.532570

Table S120. XYZ Coordinates of H_mono_TS2

87

scf done: -5572.134444

C	-2.513361	2.227961	2.065385
C	-1.202923	2.705557	2.236383
C	-0.868749	3.378383	3.420557
C	-1.830486	3.575648	4.415371
C	-3.133291	3.105623	4.237034
C	-3.471986	2.432649	3.059091
P	0.063711	2.412455	0.925740
C	-0.474683	3.583442	-0.395658
C	0.065792	4.875677	-0.473963
C	-0.349756	5.752854	-1.477958
C	-1.309183	5.349130	-2.410469
C	-1.847474	4.061550	-2.339703
C	-1.428883	3.177462	-1.342225

Pd	0.520196	0.128903	0.448735
P	-1.511499	-1.062504	-0.127350
O	-2.634332	-0.016012	-0.730574
P	2.916722	0.370905	0.236510
C	4.489114	-0.601457	0.026184
C	5.375911	-0.647257	1.113286
C	6.574870	-1.355469	1.021687
C	6.902632	-2.032099	-0.156392
C	6.027527	-1.989436	-1.243451
C	4.828706	-1.277043	-1.154389
C	2.668964	0.992926	-1.490827
C	2.033942	0.254690	-2.504834
C	1.882323	0.800457	-3.781631
C	2.353890	2.086324	-4.058471
C	2.979722	2.827024	-3.052593
C	3.136719	2.285838	-1.775114
O	3.313244	1.505733	1.210594
C	1.961205	-1.362395	1.106907
C	2.080933	-1.287969	2.508821
C	2.193614	-2.451617	3.277624
C	2.214910	-3.701411	2.656670
C	2.129928	-3.785088	1.261065
C	2.017832	-2.628582	0.490956
C	-1.345218	-2.312215	-1.476280
C	-1.403547	-1.893546	-2.816538
C	-1.175707	-2.799205	-3.854209
C	-0.876569	-4.134283	-3.569363
C	-0.811454	-4.559169	-2.240133
C	-1.042984	-3.655740	-1.200459
C	-2.385594	-2.013288	1.189885
C	-1.685182	-2.330345	2.363992
C	-2.305895	-3.052515	3.387613
C	-3.637070	-3.449420	3.253059
C	-4.346063	-3.125592	2.091456
C	-3.726741	-2.415425	1.062483
O	1.370751	3.171473	1.563247
Br	-5.375377	-0.885959	-2.146398
H	2.312171	-4.605139	3.251086
H	2.147963	-4.755717	0.772734
H	1.957804	-2.716328	-0.589036
H	2.097620	-0.317867	2.997200
H	2.275251	-2.376547	4.358521
H	-3.454545	-0.403427	-1.150112
H	2.170375	2.554060	1.464577
H	-4.291244	-2.168154	0.165950
H	-0.653742	-2.010082	2.477574
H	-5.384109	-3.428972	1.986779
H	-1.751099	-3.296139	4.289253
H	-4.123429	-4.004736	4.050031
H	-1.647365	-0.861246	-3.047306

H	-0.997092	-4.002345	-0.172607
H	-1.237383	-2.462449	-4.885277
H	-0.587125	-5.597057	-2.010382
H	-0.699968	-4.838607	-4.377056
H	0.814518	5.184881	0.248293
H	-1.842264	2.172701	-1.300483
H	0.075659	6.751281	-1.531981
H	-2.588646	3.741091	-3.066748
H	-1.631082	6.032286	-3.191356
H	3.616618	2.864153	-0.991834
H	1.648689	-0.740744	-2.306731
H	3.347803	3.827467	-3.261518
H	1.392350	0.219626	-4.557652
H	2.233018	2.508564	-5.051643
H	5.128459	-0.115536	2.026103
H	4.170619	-1.243662	-2.016322
H	7.254093	-1.376421	1.869353
H	6.278386	-2.504657	-2.166362
H	7.835534	-2.583767	-0.227878
H	-2.784510	1.689856	1.160876
H	0.143136	3.745272	3.555814
H	-4.482996	2.061498	2.915699
H	-1.560288	4.098642	5.328704
H	-3.879729	3.259080	5.011122

Table S121. XYZ Coordinates of H_mono_VI

87

scf done: -5572.167470

C	-2.256123	3.350788	-1.471587
C	-0.855828	3.451239	-1.399464
C	-0.191659	4.357213	-2.240400
C	-0.914584	5.156935	-3.129616
C	-2.306732	5.062950	-3.187944
C	-2.973693	4.159115	-2.355935
P	0.086661	2.364427	-0.236107
C	-0.226241	3.194377	1.389420
C	-1.488942	3.106608	2.001340
C	-1.713371	3.714802	3.238271
C	-0.683171	4.403969	3.885462
C	0.575724	4.483880	3.287004
C	0.804216	3.883798	2.045291
Pd	-0.029636	0.003901	-0.420454
P	2.219595	-0.641749	-0.134579
O	3.164440	0.644197	-0.608232
C	-2.074575	-0.813093	-1.081649
C	-1.115439	-1.885210	-1.034128
C	-0.615200	-2.429191	-2.260606
C	-0.990066	-1.904450	-3.475844
C	-1.908502	-0.818023	-3.531913
C	-2.445314	-0.300410	-2.377185

P	-3.290467	-0.526012	0.250007
O	-3.599613	0.932843	0.531156
H	-0.604588	-2.330831	-4.397632
C	-2.722942	-1.400406	1.756990
C	-2.237173	-0.625795	2.821413
C	-1.823357	-1.237025	4.007399
C	-1.888556	-2.626240	4.139367
C	-2.377759	-3.404023	3.086152
C	-2.800168	-2.794999	1.903140
C	-4.808553	-1.409484	-0.282313
C	-6.044986	-0.920549	0.167811
C	-7.230981	-1.562789	-0.190722
C	-7.193164	-2.699468	-1.002671
C	-5.967442	-3.189169	-1.460180
C	-4.779460	-2.546070	-1.105638
O	1.624325	2.885884	-0.561249
C	2.922650	-2.027556	-1.124947
C	3.456653	-1.788173	-2.401023
C	3.876745	-2.850766	-3.204353
C	3.759276	-4.166096	-2.749643
C	3.222521	-4.414742	-1.482870
C	2.806012	-3.354215	-0.676805
C	2.783019	-1.050487	1.571467
C	4.140739	-1.198313	1.906324
C	4.509508	-1.505854	3.215932
C	3.532310	-1.679564	4.202180
C	2.182753	-1.534007	3.878321
C	1.810772	-1.213766	2.569499
Br	6.331352	0.635186	-0.769405
H	-1.570175	-3.100938	5.062819
H	-2.442123	-4.483120	3.189185
H	-3.203500	-3.409432	1.104206
H	-2.196874	0.453503	2.722123
H	-1.457037	-0.627701	4.828413
H	4.164417	0.545253	-0.612793
H	2.241585	2.118345	-0.600064
H	-6.071967	-0.029586	0.787270
H	-3.834137	-2.927641	-1.479706
H	-8.182905	-1.174792	0.159864
H	-5.935155	-4.067268	-2.098427
H	-8.116205	-3.198706	-1.282636
H	-1.013956	-2.483589	-0.134350
H	-3.175043	0.502254	-2.432213
H	0.065119	-3.274375	-2.212117
H	-2.206116	-0.413474	-4.495217
H	0.889153	4.433227	-2.193564
H	-2.785476	2.636653	-0.845156
H	-0.387955	5.855059	-3.774743
H	-4.056435	4.076795	-2.400685
H	-2.867711	5.685112	-3.879610

H	3.562232	-0.768598	-2.757560
H	2.398259	-3.561895	0.308796
H	4.298764	-2.648854	-4.184835
H	3.134121	-5.434521	-1.118969
H	4.085673	-4.991553	-3.375449
H	4.909441	-1.054346	1.151018
H	0.760743	-1.085390	2.319439
H	5.560737	-1.612088	3.468714
H	1.419020	-1.663420	4.639516
H	3.824982	-1.923434	5.219500
H	-2.292111	2.554322	1.519614
H	1.779946	3.953107	1.576145
H	-2.694494	3.645413	3.700500
H	1.382509	5.016368	3.783414
H	-0.860368	4.871334	4.849975

Table S122. XYZ Coordinates of H_mono_VII

87

scf done: -5572.167978

C	3.846964	-2.381227	2.377207
C	2.701567	-2.262203	1.576735
C	1.434124	-2.450797	2.156083
C	1.318208	-2.755971	3.511298
C	2.464905	-2.874308	4.302942
C	3.726522	-2.687412	3.735648
P	2.765576	-1.807999	-0.192242
C	2.231085	-3.292819	-1.112723
C	1.606247	-3.101901	-2.355054
C	1.193012	-4.200197	-3.109989
C	1.402116	-5.496368	-2.631349
C	2.019739	-5.693733	-1.393928
C	2.430242	-4.596929	-0.633394
H	2.373763	-3.111107	5.358889
O	1.910288	-0.598102	-0.516145
C	4.521682	-1.525184	-0.614444
C	4.976786	-0.197726	-0.649315
C	6.308545	0.080311	-0.960978
C	7.193943	-0.963187	-1.241346
C	6.746163	-2.286612	-1.213256
C	5.414739	-2.568706	-0.903630
C	2.210833	2.096981	1.706156
C	1.522207	3.314911	1.568991
C	1.735669	4.336037	2.505637
C	2.633569	4.146881	3.560434
C	3.322955	2.939387	3.687457
C	3.110111	1.915969	2.758163
P	0.329381	3.502897	0.181278
C	1.419375	3.891623	-1.247795
C	1.001248	3.495781	-2.528104
C	1.770147	3.803603	-3.652547

C	2.966486	4.509122	-3.506641
C	3.392642	4.906099	-2.235444
C	2.625795	4.597577	-1.111382
Pd	-1.280733	1.890378	-0.045897
P	-3.115850	0.478061	-0.129220
C	-3.460404	-0.316473	-1.756013
C	-4.687957	-0.936076	-2.047262
C	-4.897244	-1.522199	-3.296175
C	-3.885979	-1.506417	-4.262387
C	-2.663696	-0.893733	-3.980687
C	-2.456023	-0.293690	-2.735712
C	-3.048124	-0.967225	1.018942
C	-3.478009	-0.812025	2.347019
C	-3.355006	-1.860745	3.260036
C	-2.793304	-3.076870	2.861806
C	-2.357914	-3.239143	1.543615
C	-2.483246	-2.191704	0.627784
O	-4.500659	1.250914	0.301804
O	-0.300563	5.025860	0.488628
Br	-7.277291	-0.263736	0.826107
H	1.079262	-6.350813	-3.218810
H	2.175422	-6.699911	-1.016495
H	2.893940	-4.760033	0.334848
H	1.436005	-2.093280	-2.718495
H	0.706123	-4.044530	-4.068034
H	-5.314070	0.685064	0.436367
H	-1.239573	4.925290	0.702153
H	4.284316	0.611310	-0.439843
H	5.074210	-3.599491	-0.896980
H	6.652867	1.109828	-0.988023
H	7.430940	-3.098870	-1.437756
H	8.229566	-0.746099	-1.485944
H	0.536414	-2.359051	1.551423
H	4.831976	-2.237346	1.945774
H	0.333838	-2.900328	3.946386
H	4.618806	-2.777985	4.347815
H	1.196356	5.272504	2.407797
H	2.042971	1.288912	0.996332
H	2.792097	4.943487	4.281972
H	3.637337	0.971256	2.857615
H	4.018106	2.793050	4.509231
H	-3.926502	0.125374	2.661388
H	-2.149561	-2.335178	-0.395681
H	-3.702079	-1.729031	4.280928
H	-1.928035	-4.184166	1.224017
H	-2.701762	-3.894255	3.571157
H	-5.483015	-0.953592	-1.304827
H	-1.511457	0.199661	-2.517348
H	-5.850740	-1.994251	-3.516245
H	-1.877090	-0.873197	-4.729539

H	-4.053696	-1.966074	-5.232269
H	0.071062	2.943136	-2.635507
H	2.968095	4.903201	-0.127255
H	1.437602	3.490417	-4.637948
H	4.323994	5.453018	-2.120048
H	3.567915	4.746489	-4.379414

Table S123. XYZ Coordinates of H_para_I_P(O)Ph$_2$

86

scf done: -5571.382643

C	0.424284	3.464091	-4.399167
C	1.473882	3.030521	-3.586930
C	1.217156	2.558663	-2.296223
C	-0.097132	2.515430	-1.809833
C	-1.149491	2.943230	-2.636427
C	-0.889212	3.421081	-3.920790
P	-0.489978	1.882335	-0.127177
C	-0.683924	3.419567	0.859409
C	0.080819	4.572202	0.616564
C	-0.072844	5.700765	1.422063
C	-0.994363	5.692628	2.473879
C	-1.761629	4.552346	2.720920
C	-1.607248	3.421062	1.916626
Pd	-2.169402	0.315825	0.027879
P	-3.863658	-1.229522	0.243946
C	-4.607309	-1.402648	1.915689
C	-4.389553	-2.543915	2.699915
C	-4.916015	-2.621166	3.992821
C	-5.658965	-1.561039	4.514558
C	-5.877927	-0.418178	3.738380
C	-5.354008	-0.337705	2.448672
O	1.013946	1.392322	0.412943
O	-3.433104	-2.817408	-0.037135
C	-5.339177	-1.018714	-0.831690
C	-6.519272	-1.755764	-0.634735
C	-7.598999	-1.600245	-1.503219
C	-7.514956	-0.703458	-2.573893
C	-6.347567	0.034596	-2.776992
C	-5.263349	-0.124437	-1.910114
Br	-0.442995	-3.183488	-1.959997
C	1.094208	-2.534561	-1.017998
C	1.011184	-2.286740	0.352219
C	2.276802	-2.335931	-1.727865
C	3.402364	-1.868933	-1.047630
C	3.348751	-1.602564	0.327973
H	1.051515	0.423516	0.436271
H	-2.563613	-2.841459	-0.468342
H	0.088561	-2.458425	0.894109
C	2.147507	-1.821056	1.019418
P	4.761099	-0.934348	1.288813

H	-6.595081	-2.447447	0.199262
H	-4.349953	0.445917	-2.062427
H	-8.507415	-2.174116	-1.344584
H	-6.279963	0.732319	-3.606584
H	-8.359471	-0.580974	-3.245851
H	-3.816589	-3.370499	2.293877
H	-5.531826	0.554748	1.853639
H	-4.744936	-3.512441	4.589821
H	-6.456492	0.409724	4.137803
H	-6.065540	-1.622370	5.519751
H	0.793184	4.589960	-0.202727
H	-2.203834	2.530762	2.101226
H	0.523025	6.587803	1.227667
H	-2.480529	4.543697	3.534976
H	-1.115315	6.574711	3.095974
H	2.033566	2.222693	-1.665175
H	-2.174499	2.898455	-2.275300
H	2.495325	3.059527	-3.955640
H	-1.709977	3.751830	-4.550634
H	0.626177	3.829048	-5.401977
H	2.107043	-1.638426	2.088621
H	2.325143	-2.544386	-2.790282
H	4.324169	-1.733394	-1.604026
O	4.533097	-1.107354	2.773580
C	6.246458	-1.776221	0.648221
C	4.903664	0.829074	0.830861
C	4.719307	1.774645	1.849418
C	4.783306	3.139841	1.561719
C	5.030520	3.567711	0.256164
C	5.216643	2.629701	-0.764584
C	5.153142	1.265340	-0.480699
H	4.527242	1.431740	2.860957
H	4.640664	3.866124	2.356197
H	5.080695	4.629095	0.031982
H	5.415126	2.960424	-1.779574
H	5.307793	0.549756	-1.282514
C	7.464555	-1.089857	0.518886
C	8.621184	-1.773307	0.140334
C	8.573563	-3.146784	-0.107875
C	7.367446	-3.839799	0.026133
C	6.209814	-3.160087	0.404218
H	7.511661	-0.022493	0.708734
H	9.557153	-1.232562	0.038290
H	9.473767	-3.676612	-0.404798
H	7.327582	-4.907793	-0.165156
H	5.277762	-3.708058	0.504681

Table S124. XYZ Coordinates of H_para_TS1_P(O)Ph_2

86

scf done: -5571.355924

C	-2.817308	-2.566620	1.673772
C	-3.615655	-1.410971	1.617462
C	-4.277717	-0.983162	2.776810
C	-4.153640	-1.704212	3.968206
C	-3.364185	-2.854295	4.014137
C	-2.695967	-3.284416	2.863082
P	-3.675772	-0.429596	0.061911
O	-4.904638	0.661162	0.347033
Pd	-1.631042	0.658435	-0.573664
Br	-1.414930	1.580426	-3.163669
P	-0.981312	2.071708	1.216906
O	-2.309627	2.310304	2.202190
C	0.256524	1.415262	2.412991
C	-0.179281	0.675312	3.525139
C	0.744138	0.095848	4.398077
C	2.114219	0.241566	4.169900
C	2.557328	0.970226	3.062073
C	1.637508	1.549373	2.186263
C	-0.385977	3.790039	0.905828
C	-0.650590	4.363090	-0.348657
C	-0.288006	5.685256	-0.616654
C	0.339876	6.450178	0.368158
C	0.605643	5.891203	1.621785
C	0.247424	4.569678	1.889711
C	0.057561	0.643542	-1.859104
C	1.264392	1.354343	-1.681448
C	2.446388	0.648965	-1.511394
C	2.473069	-0.763795	-1.555390
C	1.278081	-1.451182	-1.806388
C	0.078130	-0.761817	-2.012986
P	4.012060	-1.731861	-1.463691
O	4.353325	-2.459942	-2.746158
C	-4.438171	-1.612554	-1.129525
C	-4.139316	-1.464598	-2.494179
C	-4.732463	-2.298324	-3.444468
C	-5.629378	-3.289852	-3.040995
C	-5.934261	-3.445559	-1.685736
C	-5.342925	-2.613351	-0.734571
C	5.345310	-0.574833	-0.981841
C	6.260644	-0.196920	-1.975546
C	7.303181	0.682191	-1.675872
C	7.440859	1.187086	-0.381125
C	6.537111	0.808896	0.616073
C	5.494260	-0.069485	0.319177
C	3.787413	-2.887755	-0.065373
C	4.401536	-4.146386	-0.151564
C	4.286259	-5.059048	0.898193

C	3.559280	-4.720886	2.042600
C	2.942226	-3.470498	2.134328
C	3.051003	-2.557667	1.083461
H	3.362965	1.203103	-1.335863
H	1.259739	2.437705	-1.639136
H	-0.810031	-1.299093	-2.326526
H	1.275818	-2.534353	-1.895779
H	-2.293954	-2.912245	0.785719
H	-4.887643	-0.085874	2.751379
H	-2.081428	-4.179464	2.891729
H	-4.675436	-1.364757	4.858320
H	-3.267725	-3.412920	4.940384
H	1.998469	2.118115	1.335248
H	-1.242230	0.559752	3.712881
H	3.621027	1.100034	2.885318
H	0.391094	-0.465314	5.258415
H	2.832129	-0.204430	4.851716
H	4.956066	-4.407724	-1.047501
H	4.760508	-6.033043	0.821905
H	3.469888	-5.431479	2.859076
H	2.372871	-3.204738	3.020006
H	2.555362	-1.594572	1.160551
H	4.806542	-0.365579	1.105023
H	6.647782	1.193057	1.625783
H	8.253109	1.868883	-0.146825
H	8.007915	0.968593	-2.450890
H	6.155097	-0.604189	-2.975948
H	-3.438926	-0.695701	-2.809704
H	-4.491481	-2.175639	-4.496280
H	-6.087624	-3.941866	-3.778848
H	-6.629626	-4.217366	-1.369007
H	-5.580952	-2.750054	0.315999
H	-5.761252	0.233020	0.507314
H	0.469266	4.145856	2.864598
H	1.094036	6.483732	2.389885
H	0.623542	7.477840	0.160820
H	-0.494296	6.114700	-1.592619
H	-1.134633	3.767909	-1.118229
H	-2.109766	2.856041	2.980161

Table S125. XYZ Coordinates of H_para_IIa_P(O)Ph_2

86

scf done: -5571.429715

C	4.439353	2.276033	3.651411
C	3.751057	1.060447	3.720660
C	2.759080	0.766763	2.786170
C	2.443791	1.689956	1.774538
C	3.136776	2.906663	1.710470
C	4.130758	3.196135	2.648150
P	1.087007	1.275596	0.621645

C	0.932277	2.696051	-0.517028
C	0.122318	3.791451	-0.180005
C	0.007482	4.870126	-1.058655
C	0.699890	4.863953	-2.272093
C	1.506361	3.774421	-2.611994
C	1.618703	2.689478	-1.741722
Pd	1.096329	-0.732937	-0.491593
Br	0.668752	-2.923438	-1.768146
P	3.483827	-1.222673	-0.665196
C	4.719431	0.085472	-1.005689
C	4.939941	0.480153	-2.336735
C	5.834152	1.511339	-2.626726
C	6.513090	2.163184	-1.594086
C	6.294084	1.780110	-0.269383
C	5.400250	0.749128	0.026820
C	-0.943992	-0.478672	-0.516002
C	-1.756264	-1.035988	0.488814
C	-3.151806	-0.935145	0.430385
C	-3.771221	-0.267972	-0.636061
C	-2.966576	0.287522	-1.643467
C	-1.575387	0.186991	-1.580205
O	-0.171387	1.443722	1.673312
O	3.730786	-2.157197	-2.003737
C	4.183998	-2.198491	0.718439
C	5.554677	-2.501249	0.792103
C	6.041373	-3.308629	1.819529
C	5.167033	-3.823447	2.782014
C	3.802929	-3.534691	2.712882
C	3.312948	-2.727939	1.683476
H	6.241376	-2.103192	0.051591
H	-0.930469	0.909044	1.375542
H	2.879195	-2.605497	-2.209860
H	-3.747534	-1.397951	1.212171
H	-1.307536	-1.578098	1.317852
H	-0.981603	0.631216	-2.374507
H	-3.436217	0.794238	-2.481753
P	-5.574911	-0.028088	-0.762234
H	2.249856	-2.511896	1.624895
H	7.101998	-3.535866	1.870388
H	3.120790	-3.936368	3.456107
H	5.549827	-4.449880	3.582270
H	4.428149	-0.034183	-3.143069
H	5.245104	0.460399	1.061017
H	6.003659	1.800711	-3.659639
H	6.818897	2.281362	0.538104
H	7.210616	2.963935	-1.821151
H	-0.418677	3.793313	0.760437
H	2.238534	1.840843	-2.015808
H	-0.623500	5.713639	-0.795373
H	2.041425	3.764789	-3.556677

H	0.607496	5.704017	-2.953908
H	2.229988	-0.179327	2.845556
H	2.903104	3.629557	0.936766
H	3.987241	0.342113	4.499697
H	4.661373	4.141724	2.592888
H	5.213240	2.503463	4.378362
O	-6.020710	0.209479	-2.189125
C	-6.336296	-1.499728	0.007801
C	-5.988129	1.399616	0.305688
C	-6.841924	2.378467	-0.222632
C	-7.187876	3.496890	0.539016
C	-6.682248	3.644779	1.832030
C	-5.827153	2.674507	2.363789
C	-5.479065	1.556976	1.604838
H	-7.222846	2.258485	-1.231734
H	-7.848818	4.250998	0.122063
H	-6.949667	4.514946	2.424348
H	-5.429197	2.790012	3.367524
H	-4.807159	0.815528	2.026794
C	-7.395149	-1.390450	0.921898
C	-8.023075	-2.536653	1.414192
C	-7.601533	-3.800403	0.996602
C	-6.550562	-3.919365	0.082402
C	-5.922118	-2.776588	-0.410879
H	-7.729562	-0.412774	1.253104
H	-8.839324	-2.440874	2.123913
H	-8.089297	-4.690938	1.381893
H	-6.220030	-4.900787	-0.244135
H	-5.104516	-2.879021	-1.118640

Table S126. XYZ Coordinates of H_para_IIb_P(O)Ph_2

86

scf done: -5571.432295

C	0.104383	5.009975	3.352947
C	-0.565774	3.790514	3.472400
C	-0.984433	3.109818	2.327982
C	-0.735763	3.643296	1.052569
C	-0.066333	4.873439	0.942272
C	0.351419	5.550803	2.088142
P	-1.395119	2.766939	-0.413444
O	-2.754559	3.602161	-0.813644
Pd	-1.892563	0.483768	-0.050949
P	-2.295006	-1.812719	0.308162
C	-3.561901	-2.238861	1.558773
C	-3.647150	-1.440777	2.712202
C	-4.534644	-1.774090	3.734436
C	-5.351591	-2.901563	3.610267
C	-5.276045	-3.697137	2.464997
C	-4.383551	-3.370981	1.441802
C	0.091039	0.080296	-0.180554

C	0.611159	-0.398831	-1.394412
C	1.966832	-0.703943	-1.524321
C	2.844413	-0.531316	-0.442154
C	2.333743	-0.037096	0.767438
C	0.972936	0.259187	0.896092
Br	-4.445584	1.025325	-0.049368
O	-0.951143	-2.618078	0.837897
C	-2.779194	-2.704669	-1.217203
C	-1.949633	-3.699180	-1.755919
C	-2.304074	-4.340418	-2.945540
C	-3.482830	-3.991393	-3.606345
C	-4.309038	-2.994370	-3.078229
C	-3.959865	-2.348166	-1.892861
C	-0.840587	3.615145	-3.022180
C	-0.009273	3.905396	-4.105993
C	1.376811	3.797127	-3.976491
C	1.932796	3.396895	-2.758355
C	1.107463	3.103599	-1.672611
P	4.592823	-0.980683	-0.693580
H	2.350845	-1.066036	-2.473854
H	-0.041829	-0.539781	-2.251565
H	0.611186	0.641446	1.845865
H	2.992907	0.129141	1.615000
H	-1.153055	-3.517395	1.142724
H	-3.527227	3.010872	-0.655023
H	-1.028777	-3.970766	-1.250856
H	-4.598938	-1.565620	-1.494511
H	-1.656850	-5.111181	-3.353123
H	-5.225371	-2.717720	-3.591052
H	-3.756455	-4.490754	-4.530961
H	-3.025717	-0.554444	2.801094
H	-4.331689	-3.995242	0.555772
H	-4.594987	-1.151144	4.621679
H	-5.911303	-4.572099	2.365823
H	-6.047716	-3.157087	4.403592
C	-0.287492	3.216273	-1.795415
H	-1.916305	3.711399	-3.120505
H	1.551380	2.792431	-0.732509
H	-0.446730	4.219769	-5.048919
H	3.010057	3.312389	-2.651790
H	2.021936	4.024458	-4.819952
H	-1.508077	2.162738	2.425227
H	0.132450	5.300886	-0.035105
H	-0.760674	3.367922	4.453434
H	0.869899	6.500129	1.993247
H	0.433614	5.538329	4.242739
C	5.576487	0.064857	0.440931
C	4.785039	-2.702689	-0.105335
O	5.029757	-0.822134	-2.132685
C	6.104903	1.257309	-0.078786

C	6.855276	2.108127	0.733464
C	7.085882	1.774779	2.070859
C	6.570548	0.585995	2.592929
C	5.821766	-0.269255	1.781574
H	5.936792	1.505954	-1.122104
H	7.262624	3.027210	0.322677
H	7.671220	2.436364	2.702659
H	6.756649	0.319813	3.629145
H	5.440911	-1.198907	2.193130
C	5.792550	-3.480235	-0.698072
C	5.997870	-4.798081	-0.288260
C	5.198739	-5.350808	0.716118
C	4.189567	-4.585718	1.305719
C	3.979117	-3.267528	0.895119
H	6.402450	-3.052720	-1.487816
H	6.777413	-5.393585	-0.754157
H	5.357896	-6.377027	1.034008
H	3.561265	-5.015706	2.080059
H	3.181249	-2.686712	1.347883

Table S127. XYZ Coordinates of H_para_III_P(O)Ph$_2$

86

scf done: -5571.401352

C	4.533688	5.687159	-1.471030
C	4.406033	4.386473	-1.965055
C	3.325201	3.596760	-1.573270
C	2.359506	4.105321	-0.685666
C	2.493859	5.415212	-0.195767
C	3.578298	6.199770	-0.589230
P	0.945076	3.028555	-0.265739
C	0.211403	3.733110	1.251092
C	-1.117120	4.179955	1.273512
C	-1.674336	4.659102	2.461801
C	-0.914384	4.687483	3.632105
C	0.408288	4.233187	3.617536
C	0.969581	3.753258	2.435057
Pd	1.578864	0.747050	-0.106175
P	2.338045	-1.463211	0.011947
C	4.165290	-1.325031	0.027106
C	4.853345	-1.238515	-1.195697
C	6.233673	-1.037138	-1.209983
C	6.936670	-0.907256	-0.009000
C	6.257038	-0.984780	1.209291
C	4.876867	-1.191208	1.230894
C	-0.296107	0.130359	-0.375079
C	-0.710243	-0.307345	-1.639306
C	-2.031093	-0.715976	-1.834369
C	-2.952667	-0.697738	-0.775562
C	-2.523984	-0.269247	0.488731
C	-1.203687	0.147362	0.690035

O	1.947716	-2.328558	-1.293829
C	1.887941	-2.414802	1.503746
C	1.917988	-3.819019	1.511802
C	1.585305	-4.512254	2.676193
C	1.228588	-3.816786	3.835119
C	1.196792	-2.420684	3.830921
C	1.516244	-1.720323	2.666006
O	-0.179241	3.277918	-1.445225
Br	3.346060	-5.039672	-1.921618
P	-4.656345	-1.248701	-1.131870
H	-2.342969	-1.068786	-2.813029
H	-0.011857	-0.347597	-2.468440
H	-0.897099	0.476615	1.677579
H	-3.209400	-0.264566	1.331147
H	-0.318900	4.217613	-1.647275
H	2.440272	-3.202899	-1.442954
H	2.204829	-4.367587	0.617936
H	1.473950	-0.634267	2.659496
H	1.606714	-5.597993	2.678745
H	0.913930	-1.877173	4.727315
H	0.971518	-4.362608	4.738074
H	4.312688	-1.348147	-2.130370
H	4.360472	-1.262674	2.183236
H	6.759629	-0.984489	-2.158451
H	6.801236	-0.891989	2.144299
H	8.010887	-0.749554	-0.022709
H	-1.716288	4.152915	0.369415
H	1.998634	3.403356	2.434394
H	-2.702453	5.008002	2.469924
H	1.002090	4.252662	4.526228
H	-1.350222	5.059165	4.554472
H	3.225861	2.586170	-1.960712
H	1.758132	5.820805	0.490688
H	5.147003	3.986630	-2.650273
H	3.676778	7.211241	-0.207292
H	5.377365	6.300858	-1.771978
C	-5.343996	-1.874539	0.443153
C	-5.615024	0.248547	-1.561532
O	-4.704897	-2.287008	-2.229521
C	-5.232205	-3.251794	0.692847
C	-5.726233	-3.794148	1.879582
C	-6.337096	-2.966884	2.825845
C	-6.458901	-1.597061	2.580985
C	-5.968203	-1.051337	1.392836
H	-4.769757	-3.894738	-0.049596
H	-5.637819	-4.860661	2.063969
H	-6.722816	-3.389743	3.748826
H	-6.941659	-0.953540	3.310375
H	-6.082883	0.011826	1.205524
C	-6.701502	0.089646	-2.436288

C	-7.481011	1.189050	-2.797561
C	-7.181718	2.455881	-2.289432
C	-6.097945	2.623062	-1.423962
C	-5.313622	1.525374	-1.063178
H	-6.922941	-0.893389	-2.839952
H	-8.317961	1.057937	-3.476993
H	-7.787775	3.311661	-2.571900
H	-5.858055	3.608020	-1.034711
H	-4.464345	1.669461	-0.402186

Table S128. XYZ Coordinates of H_para_IV_P(O)Ph$_2$

111

scf done: -6451.940371

C	6.776726	-1.256006	-2.083537
C	6.734528	-1.055909	-0.702125
C	5.758868	-0.229894	-0.141048
C	4.815790	0.402663	-0.961840
C	4.859042	0.194085	-2.349897
C	5.838281	-0.628462	-2.907000
P	3.514147	1.507306	-0.294748
C	4.086910	1.990093	1.379717
C	4.871312	3.141664	1.547472
C	5.282296	3.528858	2.824183
C	4.917502	2.770059	3.939232
C	4.136630	1.622694	3.777119
C	3.714422	1.234986	2.503930
Pd	1.164973	0.871052	-0.350447
P	1.641672	-1.430179	-0.099171
C	0.384854	-2.499565	0.698047
C	0.318309	-2.535593	2.100137
C	-0.652419	-3.308456	2.737868
C	-1.568399	-4.045730	1.983409
C	-1.508017	-4.012421	0.588232
C	-0.535872	-3.243649	-0.053894
C	-0.842463	0.334851	-0.450086
C	-1.650287	0.241195	0.695997
C	-2.993624	-0.131977	0.608114
C	-3.569162	-0.430902	-0.636882
C	-2.771797	-0.338192	-1.785502
C	-1.431235	0.045981	-1.690729
P	0.331886	3.094149	-0.426066
C	-0.888569	3.543394	-1.712568
C	-0.423563	3.985348	-2.962579
C	-1.328714	4.285739	-3.981654
C	-2.700928	4.143814	-3.765110
C	-3.168416	3.698519	-2.526220
C	-2.268937	3.394167	-1.504264
O	1.576489	4.161508	-0.761464
C	-0.342604	3.696236	1.160144
C	-1.139311	4.851929	1.238545

C	-1.560971	5.327969	2.479873
C	-1.193064	4.658997	3.650329
C	-0.396828	3.513596	3.581369
C	0.028806	3.033923	2.342307
O	2.931726	-1.554335	0.882169
C	2.033152	-2.275732	-1.675813
C	2.597826	-3.563275	-1.676639
C	2.884804	-4.196111	-2.885940
C	2.605046	-3.559764	-4.099375
C	2.048029	-2.279911	-4.104332
C	1.769822	-1.636755	-2.896190
O	3.839692	2.819595	-1.235791
Br	4.015603	-4.323749	1.882988
P	-5.290791	-0.998998	-0.849501
H	-3.585243	-0.184589	1.518164
H	-1.238167	0.456819	1.677192
H	-0.851374	0.122290	-2.606184
H	-3.208639	-0.564310	-2.753633
H	3.222330	-2.481901	1.160107
H	3.093102	3.456331	-1.187195
H	2.830138	-4.062142	-0.738645
H	1.354199	-0.633849	-2.902754
H	3.326646	-5.188139	-2.881307
H	1.837116	-1.778534	-5.044122
H	2.826474	-4.059411	-5.037880
H	1.035115	-1.975333	2.691880
H	-0.495238	-3.228918	-1.138036
H	-0.690169	-3.336860	3.822851
H	-2.218636	-4.584216	-0.000716
H	-2.327702	-4.642952	2.478777
H	5.151582	3.732119	0.681270
H	3.114069	0.339578	2.378797
H	5.887240	4.422391	2.947146
H	3.852081	1.030491	4.641866
H	5.238687	3.073118	4.931414
H	-1.435675	5.378060	0.336947
H	0.651505	2.145654	2.291881
H	-2.177524	6.219929	2.532063
H	-0.107383	2.993537	4.489208
H	-1.526092	5.031064	4.614494
H	0.640581	4.106366	-3.137749
H	-2.642122	3.046259	-0.546998
H	-0.959690	4.633470	-4.941650
H	-4.234454	3.587479	-2.353556
H	-3.403946	4.379563	-4.558207
H	5.736976	-0.079066	0.932504
H	4.135194	0.679689	-2.996903
H	7.463146	-1.539417	-0.058351
H	5.866601	-0.779730	-3.981916
H	7.537777	-1.897934	-2.517247

O	-5.642885	-1.156348	-2.313655
C	-5.438110	-2.588697	0.045006
C	-6.362175	0.180549	0.044299
C	-7.638330	-0.226255	0.470382
C	-8.517184	0.691853	1.045180
C	-8.133675	2.026970	1.198170
C	-6.870430	2.442761	0.772044
C	-5.989989	1.525389	0.195896
H	-7.945064	-1.261864	0.358936
H	-9.498817	0.365233	1.375019
H	-8.817639	2.740413	1.648230
H	-6.568927	3.479348	0.888919
H	-5.009723	1.855741	-0.130985
C	-5.463421	-3.761285	-0.724628
C	-5.530473	-5.010689	-0.104647
C	-5.574964	-5.097679	1.289056
C	-5.555255	-3.933266	2.062445
C	-5.486473	-2.683523	1.444790
H	-5.440589	-3.684451	-1.807040
H	-5.554506	-5.913010	-0.708462
H	-5.631761	-6.068861	1.771796
H	-5.599826	-3.997536	3.145523
H	-5.488113	-1.787210	2.057601
H	1.323991	5.099042	-0.779654

Table S129. XYZ Coordinates of H_para_V_P(O)Ph_2

110

scf done: -6451.489131

C	6.703186	-1.599830	-1.970764
C	6.667135	-1.307413	-0.605889
C	5.712601	-0.421662	-0.100791
C	4.782733	0.181449	-0.957864
C	4.824045	-0.119257	-2.329292
C	5.779421	-1.002507	-2.832688
P	3.503437	1.375409	-0.386141
C	4.052122	1.883177	1.294754
C	4.745245	3.091390	1.461276
C	5.137730	3.508099	2.735195
C	4.842411	2.723016	3.852285
C	4.148963	1.519928	3.693354
C	3.748166	1.102228	2.422462
Pd	1.143226	0.787165	-0.447962
P	1.542106	-1.537070	-0.075120
C	0.253336	-2.547807	0.758500
C	0.181729	-2.527158	2.160801
C	-0.812375	-3.244177	2.827404
C	-1.750583	-3.982332	2.101506
C	-1.687029	-4.005812	0.706366
C	-0.690096	-3.293542	0.036921
C	-0.888211	0.362864	-0.509197

C	-1.650064	0.225878	0.666980
C	-3.010512	-0.085889	0.632241
C	-3.661387	-0.289245	-0.595122
C	-2.916108	-0.166576	-1.776266
C	-1.558224	0.161320	-1.728188
P	0.630930	3.082155	-0.843159
C	-0.966298	3.482805	-1.676602
C	-0.980618	3.493247	-3.080249
C	-2.153602	3.788135	-3.777228
C	-3.328289	4.079700	-3.079631
C	-3.320961	4.079609	-1.682546
C	-2.148374	3.780948	-0.984253
O	1.712965	3.796671	-1.725763
C	0.531963	4.002145	0.754541
C	1.161732	5.252142	0.848398
C	1.123292	5.974994	2.042636
C	0.456830	5.456180	3.155800
C	-0.166541	4.208195	3.073242
C	-0.123040	3.481132	1.881391
O	2.840864	-1.724838	0.895476
C	1.875920	-2.452887	-1.630518
C	2.438434	-3.740828	-1.616251
C	2.678792	-4.409950	-2.816627
C	2.354212	-3.809466	-4.037259
C	1.796738	-2.529859	-4.058163
C	1.565306	-1.851438	-2.859155
O	3.848038	2.640134	-1.335281
Br	3.820147	-4.544160	1.961320
P	-5.412193	-0.767507	-0.740332
H	-3.556346	-0.173287	1.568246
H	-1.177552	0.342148	1.638989
H	-1.024431	0.269863	-2.668703
H	-3.408746	-0.323382	-2.731575
H	3.076521	-2.658335	1.184531
H	2.958609	3.179080	-1.545498
H	2.702946	-4.214486	-0.673462
H	1.147753	-0.849018	-2.874322
H	3.119436	-5.402599	-2.799663
H	1.549656	-2.056157	-5.003718
H	2.540423	-4.336698	-4.968436
H	0.913614	-1.964521	2.731999
H	-0.647277	-3.324743	-1.047019
H	-0.852124	-3.227539	3.912714
H	-2.414310	-4.578221	0.138343
H	-2.528493	-4.535675	2.618680
H	4.967649	3.700729	0.591278
H	3.213601	0.165016	2.301706
H	5.673414	4.445617	2.854550
H	3.916983	0.906750	4.559568
H	5.148026	3.048240	4.842566

H	1.684759	5.645793	-0.017683
H	-0.600798	2.508255	1.828055
H	1.614783	6.941937	2.104747
H	-0.681300	3.797708	3.937362
H	0.428059	6.018444	4.084695
H	-0.065656	3.284554	-3.626027
H	-2.157836	3.793856	0.100761
H	-2.148384	3.794924	-4.863600
H	-4.227128	4.321367	-1.134239
H	-4.241127	4.312572	-3.620182
H	5.696874	-0.202063	0.961031
H	4.112502	0.342291	-3.007596
H	7.384320	-1.765794	0.068885
H	5.801590	-1.224029	-3.895787
H	7.446863	-2.288356	-2.361378
O	-5.846356	-0.873450	-2.187609
C	-5.601946	-2.369762	0.126130
C	-6.382984	0.439316	0.230647
C	-7.669735	0.094567	0.679203
C	-8.471371	1.038668	1.320737
C	-7.998710	2.338907	1.519191
C	-6.724376	2.693173	1.071209
C	-5.920759	1.749838	0.427803
H	-8.044716	-0.914073	0.533168
H	-9.461940	0.759612	1.667313
H	-8.622112	3.072755	2.021522
H	-6.354123	3.702692	1.223587
H	-4.932378	2.033772	0.081426
C	-5.690781	-3.524438	-0.665668
C	-5.790755	-4.783345	-0.069945
C	-5.805304	-4.898378	1.322433
C	-5.722187	-3.752164	2.118300
C	-5.620012	-2.493038	1.524396
H	-5.691265	-3.425876	-1.746607
H	-5.863695	-5.671098	-0.691272
H	-5.887839	-5.876769	1.786614
H	-5.743310	-3.837605	3.200682
H	-5.572188	-1.610285	2.154813

Table S130. XYZ Coordinates of H_para_TS2_P(O)Ph_2

110

scf done: -6451.447989

C	1.993433	2.723745	2.143781
C	1.677878	3.332556	0.916261
C	2.538990	4.310580	0.393141
C	3.691520	4.678168	1.089934
C	3.998124	4.073688	2.311814
C	3.147347	3.097249	2.836255
P	0.183300	2.909011	-0.083887
O	0.240858	3.487033	-1.515371

Pd	1.066461	0.643018	-0.281132
P	2.563394	1.084106	-2.078117
O	2.094328	2.390321	-2.954497
C	-0.994282	1.139819	0.195270
C	-1.786752	0.941768	-0.956119
C	-3.063766	0.387791	-0.860879
C	-3.595393	0.043257	0.390424
C	-2.821225	0.259538	1.542884
C	-1.546844	0.812802	1.450144
P	-5.261435	-0.676269	0.611703
O	-5.571354	-0.898527	2.076247
C	-5.292811	-2.245683	-0.326772
C	-5.317986	-2.306302	-1.729634
C	-5.299788	-3.541550	-2.379167
C	-5.259729	-4.724737	-1.634956
C	-5.240117	-4.671354	-0.239409
C	-5.255911	-3.437005	0.412738
C	-6.423961	0.450308	-0.233631
C	-6.165681	1.829053	-0.303100
C	-7.114933	2.697558	-0.844176
C	-8.331328	2.199955	-1.316941
C	-8.600571	0.830734	-1.244975
C	-7.653987	-0.039854	-0.704544
P	1.530133	-1.489766	0.753826
C	0.614592	-2.975358	0.158220
C	1.080694	-4.283172	0.378571
C	0.353959	-5.374995	-0.097564
C	-0.845912	-5.178051	-0.788880
C	-1.315025	-3.882781	-1.013199
C	-0.582551	-2.786156	-0.549449
C	-1.124831	3.927600	0.756466
C	-2.204816	4.365193	-0.027162
C	-3.222153	5.136839	0.535567
C	-3.176391	5.478627	1.890401
C	-2.103668	5.051830	2.674715
C	-1.081910	4.281967	2.111745
C	1.281765	-1.570748	2.581099
C	2.288035	-1.085850	3.434291
C	2.086434	-1.037973	4.814672
C	0.873223	-1.463052	5.363255
C	-0.135141	-1.941147	4.523142
C	0.066130	-1.994678	3.141893
O	3.127177	-1.857773	0.578798
C	4.352966	1.424063	-1.779260
C	5.060533	0.664499	-0.834007
C	6.417498	0.910685	-0.610265
C	7.075046	1.922401	-1.314882
C	6.371637	2.688334	-2.248528
C	5.017357	2.440543	-2.481772
C	2.635031	-0.221550	-3.380147

Br	4.621292	-4.268557	2.065073
H	-3.231056	-0.009956	2.511406
H	-0.978483	0.974773	2.360255
H	-1.406760	1.240246	-1.928647
H	-3.643811	0.248310	-1.768303
H	3.485185	-2.635209	1.095484
H	2.012059	-4.451342	0.914984
H	-0.940802	-1.778646	-0.739164
H	0.723147	-6.382492	0.073373
H	-2.244631	-3.723911	-1.551779
H	-1.410145	-6.031793	-1.153352
H	3.237550	-0.763653	3.018078
H	-0.723032	-2.377953	2.502358
H	2.879093	-0.673011	5.461802
H	-1.078264	-2.280411	4.942244
H	0.716946	-1.425634	6.437391
H	4.467927	3.037419	-3.202648
H	6.877293	3.479308	-2.795683
H	6.958899	0.316767	0.120997
H	8.128374	2.116731	-1.133773
H	2.305001	4.774514	-0.559787
H	1.350435	1.956524	2.563271
H	4.349281	5.437927	0.677741
H	3.380029	2.621988	3.784621
H	4.894967	4.361168	2.852377
H	-2.238405	4.110669	-1.081371
H	-0.250061	3.972800	2.735641
H	-4.047198	5.475506	-0.084801
H	-2.054825	5.321641	3.725775
H	-3.968575	6.078389	2.328941
H	-5.371627	-1.396954	-2.320441
H	-5.325229	-3.580330	-3.464020
H	-5.250120	-5.684628	-2.142832
H	-5.215812	-5.588639	0.341251
H	-5.249204	-3.386710	1.496839
H	-7.871656	-1.102418	-0.655615
H	-9.545379	0.440425	-1.611094
H	-9.067515	2.876750	-1.740434
H	-6.903080	3.761200	-0.897795
H	-5.223449	2.227647	0.059798
H	1.356619	2.859608	-2.443473
C	2.290833	0.066817	-4.708575
C	2.333734	-0.935642	-5.681485
C	2.721824	-2.232474	-5.338308
C	3.064373	-2.526332	-4.014906
C	3.017004	-1.529894	-3.038658
H	1.992374	1.075637	-4.973168
H	2.065271	-0.701057	-6.707924
H	2.754447	-3.010705	-6.095401
H	3.363669	-3.534170	-3.740990

H	3.271367	-1.773407	-2.010370
H	4.552837	-0.116658	-0.273552

Table S131. XYZ Coordinates of H_para_VI_P(O)Ph$_2$

110
scf done: -6451.482394

C	0.697338	-2.822648	-2.627925
P	-0.163985	-3.234403	-1.064881
Pd	0.896134	-0.282973	0.131843
P	2.316680	-1.030168	1.889363
C	1.628775	-1.982281	3.317771
C	1.699883	-1.469193	4.621964
C	1.160434	-2.185647	5.693638
C	0.546352	-3.420754	5.476115
C	0.468064	-3.934347	4.178459
C	0.997981	-3.219762	3.101981
C	0.015964	-2.467018	-3.803314
C	0.726007	-2.193616	-4.973708
C	2.120470	-2.287514	-4.985970
C	2.804074	-2.656308	-3.824898
C	2.096963	-2.921055	-2.649677
C	-1.652107	-4.186370	-1.558796
C	-1.562547	-5.587155	-1.561799
C	-2.656792	-6.363109	-1.946807
C	-3.850251	-5.747107	-2.332415
C	-3.949754	-4.353644	-2.326525
C	-2.857546	-3.574960	-1.937966
C	-0.801044	-1.686959	-0.328569
C	-0.956629	-0.473268	-1.096200
C	-1.965172	0.468267	-0.719178
C	-2.765465	0.270489	0.390514
C	-2.575928	-0.908792	1.185714
C	-1.649575	-1.853303	0.828684
O	0.713711	-4.060018	-0.143638
P	-3.937153	1.549490	0.931373
O	-3.362038	2.568501	1.894179
P	1.772301	1.875003	-0.270140
O	2.394567	2.437265	1.163657
C	0.665181	3.251412	-0.790455
C	-0.005089	4.022346	0.172436
C	-0.922972	4.999530	-0.218769
C	-1.192600	5.210529	-1.572916
C	-0.537273	4.440250	-2.537676
C	0.384812	3.466819	-2.150151
C	3.152271	1.991784	-1.483497
C	3.978643	3.125339	-1.579947
C	5.010284	3.161099	-2.518134
C	5.222639	2.076631	-3.376119
C	4.406299	0.948355	-3.286863
C	3.380359	0.903236	-2.338665

O	2.938301	0.267504	2.705005
C	3.859411	-1.976041	1.500544
C	3.787505	-3.233980	0.876918
C	4.957539	-3.930538	0.566687
C	6.208615	-3.379517	0.859967
C	6.284784	-2.125606	1.469229
C	5.117319	-1.426705	1.789225
C	-4.592259	2.355731	-0.576872
C	-4.760138	3.747492	-0.540299
C	-5.308586	4.421256	-1.633675
C	-5.692673	3.709611	-2.772076
C	-5.526662	2.322193	-2.817173
C	-4.980747	1.646714	-1.725084
C	-5.353306	0.664296	1.683531
C	-5.870291	1.169845	2.884644
C	-6.975335	0.564404	3.487035
C	-7.571151	-0.550102	2.893242
C	-7.059253	-1.061891	1.696915
C	-5.955727	-0.458349	1.093357
Br	3.578205	5.333425	1.622562
H	2.670420	-2.084690	-5.900193
H	0.189769	-1.919940	-5.877415
H	-1.068432	-2.417655	-3.816470
H	2.626028	-3.216980	-1.750296
H	3.886431	-2.745209	-3.833996
H	2.761235	3.373364	1.210009
H	2.765597	1.101800	2.209197
H	-0.638097	-6.062676	-1.249414
H	-2.952352	-2.493304	-1.923316
H	-2.579034	-7.446361	-1.942636
H	-4.878610	-3.872228	-2.617770
H	-4.702028	-6.351050	-2.631201
H	-0.572143	-0.414445	-2.109581
H	-1.552758	-2.758550	1.420937
H	-2.099329	1.344999	-1.345316
H	-3.193910	-1.072389	2.062580
H	2.179410	-0.511695	4.792298
H	0.909276	-3.620274	2.095041
H	1.223135	-1.777393	6.698705
H	-0.014150	-4.892052	4.001443
H	0.127286	-3.976789	6.309992
H	0.205582	3.872340	1.226277
H	0.896412	2.883573	-2.910780
H	-1.425707	5.596492	0.536599
H	-0.737392	4.602175	-3.593056
H	-1.908076	5.969487	-1.874988
H	3.828345	3.971849	-0.914024
H	2.756184	0.017055	-2.257740
H	5.649219	4.037371	-2.582276
H	4.567751	0.101173	-3.946838

H	6.024792	2.111890	-4.107760
H	2.821732	-3.664700	0.624582
H	5.176466	-0.455934	2.269721
H	4.891581	-4.904676	0.089486
H	7.252834	-1.689199	1.699849
H	7.116230	-3.922576	0.612217
H	-4.452562	4.293207	0.345796
H	-5.433635	5.499428	-1.596477
H	-6.118073	4.233065	-3.623317
H	-5.819847	1.766659	-3.703028
H	-4.847765	0.570553	-1.777983
H	-5.396042	2.032883	3.340949
H	-7.367910	0.961318	4.418610
H	-8.429948	-1.022119	3.361513
H	-7.517069	-1.932374	1.236505
H	-5.560729	-0.873944	0.171074

Table S132. XYZ Coordinates of H_para_VII_P(O)Ph$_2$

110

scf done: -6451.479640

C	0.922233	2.619217	-1.620089
C	1.519792	2.769681	-0.359389
C	0.791387	3.399712	0.664971
C	-0.497694	3.878212	0.430007
C	-1.082500	3.728824	-0.831242
C	-0.370230	3.097618	-1.853917
P	3.177704	2.060832	0.042749
O	3.904421	3.251971	0.913146
Pd	3.146146	0.053495	1.200477
P	3.253845	-1.752179	2.603540
O	4.242187	-1.489600	3.932030
C	4.027362	2.039317	-1.592170
C	4.385619	3.216081	-2.272335
C	5.036504	3.142767	-3.504643
C	5.330738	1.900221	-4.075357
C	4.979599	0.725973	-3.405881
C	4.337007	0.796845	-2.166386
C	3.956609	-3.337275	1.991871
C	4.736166	-3.315053	0.824719
C	5.328310	-4.485335	0.344665
C	5.145285	-5.689597	1.027563
C	4.369459	-5.722318	2.190501
C	3.775709	-4.554200	2.669336
C	1.689236	-2.281015	3.413258
C	1.569390	-2.358319	4.807986
C	0.353670	-2.728503	5.390606
C	-0.749085	-3.022269	4.586402
C	-0.634975	-2.943889	3.194576
C	0.574573	-2.570988	2.606965
Br	3.834154	6.334788	0.025871

C	-2.268713	-0.208818	-0.924941
C	-3.282561	0.415147	-0.199292
C	-4.247913	-0.350425	0.469859
C	-4.169145	-1.749816	0.419629
C	-3.149968	-2.374228	-0.298954
C	-2.194893	-1.609109	-0.986549
P	-5.618260	0.388294	1.438725
O	-6.059316	-0.514657	2.566516
P	-0.748721	-2.390062	-1.790159
C	-1.279391	-4.043799	-2.357997
C	-0.401273	-5.112313	-2.125048
C	-0.721297	-6.396006	-2.571261
C	-1.919623	-6.620638	-3.251852
C	-2.801210	-5.560863	-3.484433
C	-2.484443	-4.276740	-3.040881
C	-6.965675	0.727112	0.251985
C	-6.747521	1.033842	-1.100075
C	-7.829461	1.295869	-1.942881
C	-9.133830	1.246354	-1.445135
C	-9.358210	0.930593	-0.102612
C	-8.279604	0.670912	0.743463
C	-5.006236	2.004264	2.032222
C	-4.394633	2.036811	3.295995
C	-3.889332	3.234959	3.801144
C	-3.992246	4.409630	3.050561
C	-4.607417	4.386041	1.796345
C	-5.116516	3.188827	1.288224
O	0.462109	-2.474955	-0.882008
C	-0.400773	-1.390693	-3.281102
C	-1.392497	-1.031228	-4.207922
C	-1.056487	-0.291266	-5.341619
C	0.269375	0.096779	-5.558384
C	1.259433	-0.253219	-4.637962
C	0.925987	-0.995291	-3.502397
H	0.527416	0.674135	-6.441264
H	-1.828831	-0.014366	-6.052807
H	-2.428766	-1.312823	-4.047173
H	1.684320	-1.260350	-2.772943
H	2.288698	0.054258	-4.796357
H	3.828236	4.181219	0.552826
H	4.463276	-0.547438	3.960496
H	0.525253	-4.929062	-1.590596
H	-3.185843	-3.467315	-3.218521
H	-0.036733	-7.218151	-2.385384
H	-3.736549	-5.734305	-4.007855
H	-2.169320	-7.619190	-3.598160
H	-1.531648	0.403877	-1.434494
H	-3.104140	-3.458141	-0.323364
H	-3.303724	1.498985	-0.149061
H	-4.898567	-2.348496	0.955695

H	2.426578	-2.126397	5.431537
H	0.645657	-2.505492	1.522775
H	0.270270	-2.785359	6.472349
H	-1.492816	-3.162866	2.564951
H	-1.694342	-3.305787	5.040323
H	1.241147	3.527884	1.645377
H	1.463979	2.134632	-2.425942
H	-1.047402	4.365354	1.230183
H	-0.815001	2.982462	-2.838384
H	-2.084870	4.104928	-1.014485
H	4.167224	4.188597	-1.836215
H	4.075456	-0.114339	-1.632899
H	5.314877	4.056841	-4.021631
H	5.214504	-0.241815	-3.839727
H	5.837689	1.848887	-5.034701
H	4.873875	-2.375416	0.295248
H	3.167983	-4.590003	3.568567
H	5.927714	-4.456706	-0.560650
H	4.224447	-6.658659	2.721597
H	5.602393	-6.601484	0.654285
H	-4.327433	1.126029	3.882898
H	-3.419494	3.252934	4.779881
H	-3.601645	5.342639	3.445901
H	-4.699330	5.299582	1.216694
H	-5.606143	3.184430	0.319427
H	-8.450438	0.411516	1.783557
H	-10.371681	0.883042	0.284360
H	-9.973708	1.447143	-2.103649
H	-7.653130	1.531573	-2.988034
H	-5.739796	1.058948	-1.503208

Table S133. XYZ Coordinates of H_para_I_Me

66

scf done: -4731.392468

C	4.837637	-1.094561	-3.473607
C	4.032794	0.048990	-3.449732
C	3.331442	0.385620	-2.291809
C	3.438839	-0.409421	-1.138680
C	4.244104	-1.556840	-1.171011
C	4.938947	-1.896881	-2.335369
P	2.488295	0.066797	0.364882
O	2.787919	-1.220066	1.386319
Pd	0.275435	0.599878	0.023508
P	-1.946164	1.059597	-0.367048
C	-2.365251	2.690235	-1.101715
C	-3.675510	3.195785	-1.116579
C	-3.949748	4.421840	-1.722764
C	-2.918942	5.158931	-2.314267
C	-1.612284	4.666923	-2.300234
C	-1.336877	3.437925	-1.696370

O	-2.704605	0.063280	-1.475714
C	-3.073765	0.949961	1.083560
C	-4.305387	0.282983	1.017019
C	-5.129958	0.213801	2.143399
C	-4.733364	0.810240	3.342203
C	-3.503902	1.472218	3.417090
C	-2.675105	1.535171	2.296561
C	3.562865	1.328589	1.156147
C	4.964120	1.233765	1.148611
C	5.737726	2.195842	1.797945
C	5.121613	3.265474	2.455399
C	3.729340	3.370667	2.464446
C	2.953367	2.406045	1.817503
C	-1.450915	-3.062109	-2.061116
C	-2.815082	-3.330462	-1.863544
C	-3.231670	-3.716158	-0.580449
C	-2.326594	-3.828516	0.476128
C	-0.981948	-3.548660	0.243623
C	-0.526997	-3.166978	-1.015765
C	-3.807857	-3.180933	-2.991708
Br	0.266147	-3.714344	1.696211
H	-2.244905	-0.791890	-1.511121
H	2.020008	-1.816729	1.395802
H	0.523071	-2.962408	-1.188961
H	5.451212	0.412316	0.631743
H	1.868742	2.483784	1.818814
H	6.820750	2.114470	1.787941
H	3.247796	4.201794	2.971290
H	5.726920	4.015917	2.955538
H	4.320912	-2.180411	-0.286140
H	2.692366	1.265635	-2.284368
H	5.559089	-2.788646	-2.351171
H	3.945225	0.672476	-4.334821
H	5.377423	-1.360943	-4.377620
H	-4.480525	2.635536	-0.650614
H	-0.320756	3.051045	-1.679247
H	-4.966063	4.804855	-1.730260
H	-0.808568	5.238443	-2.755161
H	-3.134805	6.115749	-2.780741
H	-4.610236	-0.184804	0.086514
H	-1.711007	2.034082	2.364594
H	-6.081556	-0.306651	2.082987
H	-3.187153	1.930138	4.349675
H	-5.374934	0.754100	4.216731
H	-1.095409	-2.779339	-3.048532
H	-2.665561	-4.131358	1.460339
H	-4.279927	-3.937613	-0.400387
H	-3.347698	-3.398276	-3.959585
H	-4.664470	-3.847098	-2.858663
H	-4.193350	-2.155052	-3.035160

Table S134. XYZ Coordinates of H_para_TS1_Me

66

scf done: -4731.361452

C	3.134797	-1.135675	-2.164792
C	4.277187	-1.665836	-2.768715
C	5.438096	-0.895448	-2.863451
C	5.454758	0.405987	-2.353531
C	4.314499	0.936210	-1.748289
C	3.140109	0.170418	-1.647883
P	1.562074	0.828720	-0.950427
C	2.148734	2.075189	0.274464
C	1.620641	3.376215	0.267179
C	1.990625	4.297436	1.250347
C	2.884449	3.929447	2.257634
C	3.409596	2.633846	2.277690
C	3.044626	1.712342	1.295904
Pd	-0.180537	-0.626641	-0.259445
P	-2.281820	0.315133	-0.912102
O	-2.427869	0.418234	-2.572417
C	0.783438	-2.009349	1.055212
C	2.163658	-2.068967	1.330258
C	2.594280	-1.878707	2.635697
C	1.687369	-1.676392	3.700540
C	0.323390	-1.698249	3.411633
C	-0.146941	-1.914686	2.103889
C	2.194358	-1.476731	5.108615
Br	0.072354	-3.326041	-0.578749
O	1.001799	1.803489	-2.189604
C	-3.835246	-0.553239	-0.423900
C	-3.765755	-1.924103	-0.126795
C	-4.921199	-2.639587	0.194772
C	-6.158249	-1.992117	0.223542
C	-6.239044	-0.628100	-0.071657
C	-5.085535	0.088447	-0.392783
C	-2.611966	2.040643	-0.359402
C	-2.736866	2.309999	1.014696
C	-2.920022	3.616736	1.465361
C	-2.973078	4.674019	0.550978
C	-2.841883	4.416257	-0.814301
C	-2.659638	3.106526	-1.268693
H	3.662713	-1.882565	2.839974
H	2.877993	-2.217400	0.528351
H	-1.208217	-2.039278	1.919551
H	-0.400370	-1.595990	4.216634
H	-2.696528	1.497123	1.735750
H	-2.551320	2.915669	-2.331655
H	-3.021327	3.810649	2.529234
H	-2.880994	5.232514	-1.529901
H	-3.113426	5.691738	0.902978

H	3.462935	0.710981	1.323525
H	0.921648	3.670084	-0.509502
H	4.108291	2.341384	3.056307
H	1.579055	5.302420	1.226645
H	3.170998	4.645805	3.021920
H	-2.802758	-2.427596	-0.148027
H	-4.854341	-3.698874	0.424963
H	-7.056841	-2.546627	0.477816
H	-7.199550	-0.121647	-0.047764
H	-5.159715	1.149733	-0.611127
H	-3.315274	0.689904	-2.857673
H	4.343521	1.945587	-1.349089
H	6.356343	1.007443	-2.425237
H	6.328107	-1.307266	-3.330261
H	4.260814	-2.678674	-3.160508
H	2.234851	-1.739813	-2.083179
H	1.675757	2.421923	-2.515723
H	1.369362	-1.403516	5.822161
H	2.840610	-2.304209	5.423304
H	2.789585	-0.559318	5.189996

Table S135. XYZ Coordinates of H_para_IIa_Me

66

scf done: -4731.435641

C	-2.017043	4.784975	-1.601567
C	-1.232950	3.776034	-2.167206
C	-0.845759	2.677651	-1.398364
C	-1.231874	2.589665	-0.051729
C	-2.021541	3.604100	0.511703
C	-2.412448	4.696560	-0.264395
P	-0.714052	1.154769	0.955014
C	0.811766	1.666930	1.823963
C	1.427088	0.737124	2.679293
C	2.563005	1.093250	3.404887
C	3.097361	2.379711	3.279997
C	2.490983	3.307193	2.431095
C	1.351707	2.954833	1.703571
Pd	-0.734954	-0.811280	-0.226184
Br	-1.187890	-3.021237	-1.467326
C	-2.747306	-0.775881	0.209282
C	-3.261571	-1.421195	1.346700
C	-4.642634	-1.464247	1.590498
C	-5.553580	-0.864660	0.713714
C	-5.037285	-0.220091	-0.421080
C	-3.662638	-0.169566	-0.666890
C	-7.039986	-0.887988	0.985491
P	1.593812	-1.018901	-0.949355
O	1.624730	-1.873064	-2.363300
C	2.693449	-1.956203	0.179187
C	4.068178	-2.099559	-0.076617

C	4.861454	-2.881861	0.761890
C	4.292439	-3.530293	1.862632
C	2.926745	-3.399969	2.121046
C	2.130048	-2.618653	1.281075
C	2.582245	0.430194	-1.477875
C	2.469984	0.884811	-2.803141
C	3.167463	2.019236	-3.220370
C	3.978591	2.715449	-2.320731
C	4.089821	2.272779	-1.001071
C	3.394498	1.138244	-0.578300
O	-1.736353	1.166072	2.247818
H	4.519397	-1.597187	-0.926577
H	-2.476505	0.550221	2.082451
H	0.802932	-2.413915	-2.394066
H	-5.010080	-1.979168	2.475690
H	-2.591981	-1.919264	2.044872
H	-3.307440	0.345850	-1.555761
H	-5.720715	0.253836	-1.123100
H	1.065650	-2.526929	1.477864
H	5.922981	-2.985529	0.557852
H	2.480810	-3.905434	2.972370
H	4.913442	-4.136894	2.515163
H	1.854639	0.338073	-3.509589
H	3.496625	0.803856	0.448715
H	3.080756	2.354773	-4.249601
H	4.719339	2.807762	-0.296578
H	4.522739	3.596740	-2.647167
H	-2.332422	3.532154	1.548699
H	-0.245958	1.890864	-1.846156
H	-3.026194	5.476945	0.175354
H	-0.929248	3.839217	-3.207720
H	-2.323499	5.635537	-2.203088
H	1.018908	-0.263934	2.778756
H	0.887023	3.684233	1.049410
H	3.030804	0.368430	4.064085
H	2.901804	4.307447	2.333476
H	3.983379	2.656361	3.843509
H	-7.609789	-1.097249	0.074189
H	-7.297766	-1.647202	1.729285
H	-7.390242	0.079071	1.367384

Table S136. XYZ Coordinates of H_para_IIb_Me

66

scf done: -4731.436947

C	5.771158	-1.805362	0.891428
C	5.320085	-3.041869	1.357652
C	3.956277	-3.248530	1.585032
C	3.045650	-2.221510	1.341156
C	3.492200	-0.972485	0.876546
C	4.863109	-0.771365	0.653310

P	2.254853	0.364646	0.683156
O	2.163993	1.009982	2.203527
Pd	0.154024	-0.259967	-0.178015
P	-2.001978	-0.825734	-0.959898
O	-1.908033	-1.660537	-2.375618
C	-0.725674	1.031380	1.124352
C	-0.758223	2.399785	0.816439
C	-1.357171	3.317491	1.685117
C	-1.946343	2.904226	2.888124
C	-1.916099	1.536095	3.189007
C	-1.314076	0.612603	2.325374
C	-2.568880	3.902142	3.837738
Br	1.241212	-1.814750	-1.982712
C	-3.165653	0.509030	-1.414551
C	-3.344349	0.842317	-2.766132
C	-4.192501	1.892310	-3.124866
C	-4.866177	2.617969	-2.140570
C	-4.689058	2.292645	-0.792876
C	-3.840689	1.247226	-0.427697
C	-2.940371	-1.938306	0.151662
C	-2.224017	-2.745543	1.050792
C	-2.891301	-3.666143	1.860090
C	-4.280495	-3.787692	1.779131
C	-5.000724	-2.991263	0.884808
C	-4.336134	-2.071154	0.073005
C	3.083529	1.663890	-0.309118
C	3.406989	2.905457	0.257165
C	4.001837	3.899708	-0.523814
C	4.273587	3.663876	-1.872390
C	3.945412	2.430630	-2.444165
C	3.348149	1.435381	-1.671206
H	-1.367604	4.372726	1.418702
H	-0.315953	2.764261	-0.107536
H	-1.321516	-0.438863	2.598030
H	-2.371587	1.180980	4.111553
H	3.033223	1.082828	2.629197
H	-0.958569	-1.874389	-2.533108
H	3.191179	3.099110	1.302593
H	3.083597	0.483587	-2.122956
H	4.250359	4.857197	-0.075895
H	4.151371	2.244566	-3.494034
H	4.735287	4.438157	-2.477808
H	1.984472	-2.389541	1.501228
H	5.223659	0.186469	0.292879
H	3.602169	-4.210507	1.943089
H	6.829770	-1.642956	0.712543
H	6.028509	-3.844277	1.540827
H	-2.831008	0.271247	-3.531808
H	-3.706925	1.008703	0.622457
H	-4.328356	2.138183	-4.173913

H	-5.210980	2.853655	-0.023430
H	-5.526889	3.432974	-2.421019
H	-1.143201	-2.651335	1.115380
H	-4.904760	-1.456272	-0.616952
H	-2.328295	-4.283459	2.553642
H	-6.080454	-3.085907	0.818798
H	-4.801105	-4.500773	2.411436
H	-3.360627	3.443281	4.437747
H	-1.825339	4.305639	4.536984
H	-2.999046	4.752550	3.299604

Table S137. XYZ Coordinates of H_para_III_Me

66

scf done: -4731.407301

C	2.758219	4.567109	1.041293
C	2.610378	3.660735	2.093848
C	2.354600	2.313762	1.830393
C	2.246206	1.863040	0.505130
C	2.388293	2.782170	-0.550045
C	2.649385	4.125404	-0.280502
P	1.794660	0.131833	0.102075
O	2.649499	-0.272677	-1.209492
Pd	-0.510172	0.172751	-0.294611
P	-2.842484	0.341121	-0.682237
C	-3.906699	-0.547260	0.506794
C	-4.816125	-1.526870	0.085845
C	-5.584167	-2.218860	1.025734
C	-5.445106	-1.942059	2.386669
C	-4.531658	-0.972564	2.812821
C	-3.760775	-0.281096	1.879366
C	-0.486806	-1.733740	-0.896502
C	-0.116582	-2.044647	-2.209055
C	-0.141397	-3.375162	-2.639660
C	-0.523236	-4.415895	-1.780908
C	-0.888910	-4.081619	-0.470924
C	-0.873286	-2.754348	-0.023782
C	-0.515282	-5.853110	-2.248482
C	2.375324	-0.882701	1.504855
C	3.697216	-1.352105	1.571942
C	4.107348	-2.114509	2.666571
C	3.211355	-2.408185	3.698359
C	1.895699	-1.943663	3.635197
C	1.474997	-1.190945	2.537181
O	-3.270744	-0.234105	-2.167332
C	-3.403021	2.082569	-0.644450
C	-2.569568	3.052637	-1.232320
C	-2.956693	4.392265	-1.257055
C	-4.174234	4.777376	-0.689866
C	-5.006816	3.821116	-0.103067
C	-4.626416	2.478749	-0.079107

Br	5.720800	0.268589	-1.358336
H	0.144115	-3.602269	-3.664530
H	0.191212	-1.265842	-2.899526
H	-1.170254	-2.533295	0.996648
H	-1.195523	-4.865722	0.217877
H	-4.168791	0.021520	-2.433688
H	3.641007	-0.066197	-1.198865
H	4.404015	-1.117531	0.779762
H	0.446147	-0.844855	2.480094
H	5.129549	-2.478104	2.714388
H	1.195164	-2.175072	4.431875
H	3.536951	-3.001668	4.547566
H	2.312730	2.442733	-1.578630
H	2.253817	1.615641	2.655357
H	2.769755	4.825704	-1.101562
H	2.701847	3.999059	3.121589
H	2.960397	5.613455	1.249457
H	-4.922489	-1.755140	-0.969593
H	-3.050239	0.467788	2.220065
H	-6.288446	-2.974586	0.691550
H	-4.417988	-0.757250	3.870881
H	-6.042295	-2.482206	3.115019
H	-1.621366	2.760894	-1.676216
H	-5.279707	1.744037	0.379280
H	-2.307600	5.133127	-1.713505
H	-5.953918	4.119218	0.336098
H	-4.473079	5.821165	-0.703894
H	-1.188766	-6.471823	-1.648409
H	-0.820135	-5.934633	-3.296449
H	0.487692	-6.290949	-2.169405

Table S138. XYZ Coordinates of H_para_IV_Me

91

scf done: -5611.945939

C	0.594470	-5.606597	-1.681695
C	0.986171	-5.073133	-2.912249
C	1.268546	-3.709191	-3.022788
C	1.155028	-2.875984	-1.907940
C	0.770365	-3.410039	-0.667176
C	0.487676	-4.780675	-0.561347
P	0.586504	-2.321184	0.798832
C	2.221161	-2.340202	1.630169
C	3.398657	-2.710711	0.967242
C	4.617363	-2.709720	1.648383
C	4.670545	-2.340344	2.994084
C	3.499844	-1.968776	3.660408
C	2.280877	-1.964175	2.982038
Pd	-0.552017	-0.222782	0.301497
P	1.449244	0.959434	-0.112688
O	2.516882	-0.079696	-0.768467

C	-1.539970	1.566801	-0.090226
C	-2.024436	1.875485	-1.369689
C	-2.698620	3.076565	-1.617074
C	-2.900843	4.020017	-0.600519
C	-2.414582	3.711616	0.676760
C	-1.750580	2.506611	0.929519
C	-3.594388	5.333567	-0.879261
P	-2.756001	-1.086666	0.479417
O	-2.725117	-2.601151	1.194626
C	-3.567999	-1.393520	-1.129040
C	-4.959586	-1.553362	-1.241920
C	-5.530079	-1.875209	-2.473701
C	-4.720651	-2.040873	-3.600350
C	-3.335964	-1.888723	-3.495551
C	-2.760541	-1.567269	-2.266059
C	-3.991846	-0.223256	1.517994
C	-4.158375	-0.621835	2.854613
C	-5.065534	0.046331	3.679054
C	-5.809645	1.116814	3.179526
C	-5.641485	1.522710	1.853161
C	-4.733776	0.862678	1.024876
O	-0.224895	-3.248560	1.892885
C	2.199278	1.666944	1.401398
C	3.474166	2.259241	1.364402
C	4.032473	2.783992	2.529780
C	3.326794	2.730562	3.735945
C	2.063517	2.138516	3.780341
C	1.504656	1.601954	2.618293
C	1.416508	2.348636	-1.309373
C	1.490546	2.057012	-2.680963
C	1.430463	3.083903	-3.623387
C	1.291369	4.410356	-3.206987
C	1.216174	4.706706	-1.844205
C	1.278373	3.682097	-0.897876
Br	5.335425	0.880471	-1.779424
H	-3.071546	3.280807	-2.618934
H	-1.889327	1.178512	-2.191839
H	-1.406429	2.308534	1.941001
H	-2.560447	4.419371	1.490554
H	3.404783	0.297589	-1.067572
H	-1.193639	-3.141843	1.767937
H	4.037036	2.291672	0.434879
H	0.529224	1.125869	2.658012
H	5.019603	3.235471	2.496686
H	1.516177	2.086885	4.716755
H	3.764514	3.144805	4.639454
H	1.613725	1.030603	-3.011486
H	1.222526	3.925423	0.157733
H	1.497504	2.847914	-4.681323
H	1.114832	5.736556	-1.514786

H	1.245796	5.209323	-3.941144
H	0.179470	-5.193278	0.393766
H	1.382782	-1.818414	-1.993561
H	0.371973	-6.665917	-1.592894
H	1.573890	-3.291566	-3.977714
H	1.068663	-5.717608	-3.782467
H	-5.598595	-1.423493	-0.374882
H	-1.683488	-1.450273	-2.189122
H	-6.606193	-1.994890	-2.552465
H	-2.704305	-2.017886	-4.368963
H	-5.168464	-2.288142	-4.558119
H	-3.590145	-1.457485	3.249637
H	-4.604360	1.195703	0.000712
H	-5.191056	-0.274141	4.708770
H	-6.216063	2.356125	1.460745
H	-6.517119	1.633849	3.820776
H	3.368604	-3.002241	-0.076697
H	1.375586	-1.678301	3.508492
H	5.523865	-3.001633	1.126629
H	3.534480	-1.681892	4.707195
H	5.619676	-2.342292	3.521873
H	-3.582990	-3.053883	1.240884
H	-4.094000	5.720395	0.014057
H	-2.879134	6.098472	-1.207511
H	-4.342402	5.231175	-1.671493

Table S139. XYZ Coordinates of H_para_V_Me

90

scf done: -5611.492460

C	1.672567	1.717792	2.551329
C	2.406424	1.465094	1.382572
C	3.800900	1.636152	1.393275
C	4.442313	2.058957	2.558161
C	3.703406	2.323180	3.715577
C	2.317709	2.153626	3.711248
P	1.517951	0.923402	-0.131419
Pd	-0.608135	-0.048599	0.334364
P	-2.784410	-0.927265	0.743608
C	-4.163747	0.231781	1.147596
C	-4.453793	0.454448	2.502159
C	-5.483894	1.320635	2.875116
C	-6.238579	1.973206	1.897587
C	-5.960214	1.751734	0.546078
C	-4.929466	0.887184	0.172801
C	1.635847	2.386700	-1.238922
C	1.678366	2.185122	-2.627588
C	1.727304	3.274721	-3.498056
C	1.728587	4.577035	-2.992145
C	1.685404	4.785040	-1.611467
C	1.640177	3.696627	-0.738218

O	2.457765	-0.180379	-0.882193
P	0.323817	-2.231329	0.898541
C	1.988483	-2.368032	1.675283
C	3.129685	-2.785232	0.977061
C	4.364017	-2.865799	1.625419
C	4.472677	-2.529499	2.976420
C	3.339866	-2.111295	3.679749
C	2.106160	-2.029006	3.033286
C	-1.480633	1.789227	-0.083358
C	-1.746352	2.209083	-1.396636
C	-2.315307	3.458628	-1.668379
C	-2.627855	4.354160	-0.637347
C	-2.348660	3.949600	0.675148
C	-1.798805	2.691537	0.945294
C	-3.264414	5.694728	-0.926440
C	0.391013	-3.390411	-0.529845
C	-0.099289	-4.696916	-0.385082
C	-0.080954	-5.579802	-1.466751
C	0.426067	-5.166483	-2.701379
C	0.910783	-3.864659	-2.853520
C	0.888163	-2.975594	-1.776380
O	-0.569064	-2.996851	2.011240
O	-2.802540	-1.965345	1.920186
C	-3.398704	-1.798063	-0.765989
C	-4.020663	-3.046264	-0.615456
C	-4.490547	-3.741371	-1.731792
C	-4.341994	-3.197614	-3.010487
C	-3.715076	-1.958919	-3.170234
C	-3.238862	-1.265790	-2.054827
Br	5.348363	0.442093	-2.012926
H	-2.508281	3.744066	-2.701307
H	-1.488755	1.569149	-2.237658
H	-1.624814	2.419342	1.984107
H	-2.571084	4.623514	1.501088
H	3.361223	0.114420	-1.209197
H	-1.546639	-2.586609	2.024755
H	4.386570	1.433476	0.499242
H	0.596896	1.567930	2.552978
H	5.521290	2.184358	2.562114
H	1.739844	2.350579	4.609531
H	4.207860	2.655491	4.618279
H	1.689939	1.176217	-3.027659
H	1.614644	3.871450	0.332595
H	1.768314	3.105724	-4.570205
H	1.693375	5.794932	-1.211731
H	1.767302	5.424880	-3.669830
H	-0.499027	-5.011219	0.573643
H	1.268156	-1.965671	-1.896627
H	-0.463398	-6.589390	-1.345482
H	1.303720	-3.538671	-3.812440

H	0.439535	-5.854026	-3.542187
H	-4.124418	-3.466829	0.380101
H	-2.742090	-0.309799	-2.187175
H	-4.970502	-4.707734	-1.604020
H	-3.590933	-1.534931	-4.162763
H	-4.706752	-3.738989	-3.878673
H	-3.880650	-0.068298	3.261234
H	-4.728984	0.723171	-0.880769
H	-5.699598	1.480550	3.927944
H	-6.549352	2.248720	-0.219638
H	-7.041945	2.645115	2.185935
H	3.059146	-3.052926	-0.071256
H	1.228985	-1.712820	3.589364
H	5.239615	-3.195222	1.073493
H	3.415455	-1.850769	4.731571
H	5.433405	-2.594427	3.478988
H	-2.964477	6.448043	-0.190926
H	-2.990300	6.062728	-1.920134
H	-4.360089	5.633758	-0.895557

Table S140. XYZ Coordinates of H_para_TS2_Me

90

scf done: -5611.454766

C	-1.405217	-2.671090	2.013934
C	-2.177319	-2.284699	0.907421
C	-3.475164	-2.805431	0.762309
C	-3.981390	-3.698782	1.707010
C	-3.200803	-4.088861	2.800314
C	-1.911869	-3.575617	2.951883
P	-1.452784	-1.088285	-0.295445
O	-2.695874	-0.094479	-0.728470
Pd	0.471961	0.219920	0.385947
P	2.812564	0.749072	0.153644
C	4.468986	-0.007417	-0.232116
C	5.390019	-0.124125	0.820840
C	6.652109	-0.677652	0.602214
C	7.010738	-1.127368	-0.671620
C	6.102200	-1.012217	-1.725310
C	4.839631	-0.453674	-1.508375
P	-0.207038	2.342337	1.210911
C	-0.929942	3.634109	0.107499
C	-0.527627	4.973927	0.213260
C	-1.078732	5.943407	-0.627300
C	-2.037331	5.584959	-1.578871
C	-2.438887	4.251263	-1.691210
C	-1.884379	3.276346	-0.858229
C	-1.433243	2.285039	2.590177
C	-1.113285	2.799097	3.855075
C	-2.038664	2.729413	4.900173
C	-3.291321	2.148324	4.692376

C	-3.615593	1.632446	3.433866
C	-2.692271	1.694185	2.388826
O	1.039773	3.139706	1.918474
C	2.079567	-1.188249	0.784868
C	2.259999	-1.308395	2.175125
C	2.532849	-2.550990	2.757248
C	2.661725	-3.703861	1.974415
C	2.509542	-3.575040	0.581376
C	2.238683	-2.343606	-0.007081
C	2.984271	-5.042618	2.592802
C	-1.216346	-2.129853	-1.801491
C	-1.357141	-1.545538	-3.071862
C	-1.082967	-2.280816	-4.226415
C	-0.654211	-3.607458	-4.129797
C	-0.506189	-4.195714	-2.871378
C	-0.784078	-3.463240	-1.715021
O	3.131037	1.772191	1.270644
C	2.436437	1.577936	-1.460433
C	2.748848	2.942360	-1.567758
C	2.484257	3.634901	-2.751050
C	1.904409	2.975294	-3.837758
C	1.587644	1.618052	-3.736913
C	1.847508	0.920820	-2.554832
Br	-5.381408	-1.061226	-2.187896
H	2.601193	-4.457006	-0.048805
H	2.130177	-2.289295	-1.085741
H	2.196832	-0.426330	2.806187
H	2.656890	-2.618545	3.835511
H	-3.486694	-0.508170	-1.178168
H	1.895792	2.635652	1.706336
H	-4.094714	-2.508895	-0.081412
H	-0.407404	-2.261696	2.141752
H	-4.987008	-4.093168	1.589811
H	-1.302419	-3.870387	3.801459
H	-3.599173	-4.786370	3.531661
H	-1.701865	-0.519664	-3.156492
H	-0.672304	-3.936282	-0.744225
H	-1.209425	-1.818463	-5.201373
H	-0.180737	-5.228881	-2.787952
H	-0.441478	-4.179303	-5.028332
H	0.220607	5.248725	0.949696
H	-2.192420	2.238780	-0.960609
H	-0.759334	6.978283	-0.539112
H	-3.179131	3.966840	-2.434025
H	-2.464659	6.339861	-2.232791
H	3.193240	3.456549	-0.721266
H	1.583421	-0.130183	-2.491170
H	2.732668	4.689939	-2.823579
H	1.134938	1.099062	-4.576788
H	1.699638	3.515309	-4.757424

H	5.117751	0.232379	1.808784
H	4.154986	-0.359046	-2.344691
H	7.356300	-0.755353	1.425899
H	6.375559	-1.350301	-2.720900
H	7.992802	-1.558983	-0.842079
H	-2.950453	1.274836	1.419985
H	-0.140518	3.252323	4.013324
H	-4.587252	1.175958	3.266724
H	-1.779583	3.131441	5.875869
H	-4.009692	2.094216	5.505360
H	2.313030	-5.824389	2.221329
H	4.006086	-5.356203	2.346774
H	2.900126	-5.010238	3.682301

Table S141. XYZ Coordinates of H_para_VI_Me

90
scf done: -5611.487763

C	-2.193936	-0.064978	2.952129
C	-2.716733	-1.008397	2.054419
C	-2.825506	-2.349639	2.455964
C	-2.400425	-2.741843	3.726635
C	-1.876264	-1.797127	4.613263
C	-1.777358	-0.458440	4.226041
P	-3.283565	-0.412652	0.416634
C	-4.797488	-1.380640	0.042070
C	-6.038383	-0.819239	0.381694
C	-7.220872	-1.517919	0.133918
C	-7.174975	-2.783987	-0.455697
C	-5.944676	-3.347461	-0.802802
C	-4.760274	-2.648507	-0.559420
C	-2.061939	-0.924558	-0.836642
C	-1.105518	-1.970884	-0.606709
C	-0.597253	-2.715245	-1.718940
C	-0.948509	-2.419707	-3.018870
C	-1.869973	-1.350071	-3.246745
C	-2.416914	-0.643351	-2.204614
C	-0.407299	-3.204229	-4.188921
Pd	-0.017835	0.009385	-0.311716
P	0.090422	2.365944	-0.527088
O	1.629847	2.833507	-0.921148
O	-3.600266	1.071477	0.435452
P	2.228007	-0.589880	0.065558
C	2.920956	-2.093441	-0.744117
C	3.360416	-2.031979	-2.076660
C	3.763188	-3.189905	-2.745041
C	3.722056	-4.426882	-2.096758
C	3.280558	-4.499026	-0.772520
C	2.882130	-3.341754	-0.100994
C	2.795023	-0.775953	1.808885
C	4.154806	-0.827579	2.163169

C	4.525079	-0.966638	3.500933
C	3.548028	-1.065424	4.497471
C	2.196316	-1.014212	4.154242
C	1.822231	-0.862456	2.816008
O	3.177101	0.620970	-0.570840
C	-0.243092	3.461828	0.928490
C	-1.510662	3.471376	1.536953
C	-1.749536	4.281747	2.648988
C	-0.729473	5.079933	3.175639
C	0.534080	5.065037	2.581888
C	0.777084	4.261794	1.463936
C	-0.844010	3.232686	-1.868514
C	-0.175239	3.977874	-2.851576
C	-0.893402	4.608803	-3.871032
C	-2.285230	4.504946	-3.919279
C	-2.956687	3.759699	-2.945632
C	-2.243869	3.119305	-1.929779
Br	6.345737	0.552143	-0.768912
H	-1.556486	-2.101964	5.605427
H	-2.490042	-3.781421	4.027484
H	-3.255323	-3.089440	1.787884
H	-2.126132	0.975956	2.655364
H	-1.381729	0.281073	4.916022
H	4.175087	0.509388	-0.580042
H	2.249236	2.073284	-0.823061
H	-6.071442	0.170056	0.827332
H	-3.810810	-3.090007	-0.847304
H	-8.176333	-1.073652	0.397282
H	-5.906099	-4.327508	-1.269159
H	-8.095235	-3.327364	-0.649340
H	-1.005018	-2.406679	0.382331
H	-3.142415	0.139474	-2.406877
H	0.085486	-3.536370	-1.517147
H	-2.159959	-1.110808	-4.267160
H	0.905342	4.061676	-2.812839
H	-2.775658	2.523313	-1.191674
H	-0.363352	5.183551	-4.625779
H	-4.039161	3.669542	-2.981282
H	-2.842452	4.996027	-4.712085
H	3.403761	-1.074100	-2.585760
H	2.549610	-3.411891	0.930866
H	4.111689	-3.124175	-3.771891
H	3.252954	-5.455805	-0.258653
H	4.034834	-5.327109	-2.617636
H	4.923574	-0.742649	1.398771
H	0.769880	-0.805116	2.550127
H	5.577744	-0.999584	3.767634
H	1.432161	-1.084918	4.922690
H	3.842070	-1.177464	5.537212
H	-2.306694	2.838667	1.151780

H	1.756471	4.256608	0.997378
H	-2.734192	4.285360	3.108927
H	1.333227	5.681141	2.985225
H	-0.918026	5.704941	4.043933
H	-1.214739	-3.682317	-4.756902
H	0.127678	-2.552094	-4.889887
H	0.284966	-3.984469	-3.861417

Table S142. XYZ Coordinates of H_para_VII_Me

90

scf done: -5611.488616

C	-3.488455	-1.064725	2.245498
C	-3.065684	-1.071625	0.906168
C	-2.431462	-2.217994	0.401127
C	-2.229264	-3.333667	1.217017
C	-2.656728	-3.318777	2.547620
C	-3.288805	-2.181900	3.058410
P	-3.227706	0.466076	-0.104135
O	-4.640996	1.125008	0.414476
Pd	-1.449025	1.938243	0.089594
P	0.156305	3.530414	0.455032
O	-0.457242	5.020181	0.917522
C	1.358180	3.193077	1.805453
C	1.597352	4.115470	2.833737
C	2.502211	3.811090	3.855099
C	3.173496	2.586785	3.856461
C	2.935159	1.661322	2.835130
C	2.027792	1.957065	1.816615
C	1.236871	4.051277	-0.938886
C	0.796966	3.801287	-2.248502
C	1.557990	4.214895	-3.343881
C	2.768945	4.879979	-3.139815
C	3.217260	5.131097	-1.839481
C	2.457753	4.717632	-0.744380
C	-3.559528	-0.192228	-1.792975
C	-4.755981	-0.851255	-2.124355
C	-4.956072	-1.329020	-3.420019
C	-3.965937	-1.165020	-4.394361
C	-2.774626	-0.512125	-4.073082
C	-2.576869	-0.020426	-2.779928
Br	-7.342246	-0.563582	0.794704
O	1.802233	-0.452559	-0.772423
P	2.773454	-1.600505	-0.576362
C	4.485963	-1.118410	-0.999223
C	4.820906	0.240897	-0.896692
C	6.113376	0.671102	-1.200259
C	7.079730	-0.251262	-1.609132
C	6.751673	-1.605101	-1.718515
C	5.459241	-2.038931	-1.418175
C	2.355969	-3.036514	-1.627081

C	1.703806	-2.788429	-2.845004
C	1.373579	-3.845619	-3.693600
C	1.693032	-5.157577	-3.333330
C	2.338099	-5.412268	-2.120653
C	2.665922	-4.356990	-1.266833
C	2.796322	-2.216840	1.140551
C	1.559523	-2.443630	1.770765
C	1.509584	-2.899524	3.084514
C	2.686003	-3.139574	3.813887
C	3.914098	-2.911932	3.180147
C	3.974249	-2.454591	1.861523
C	2.621152	-3.610847	5.246233
H	1.434675	-5.980210	-3.993620
H	2.579540	-6.431738	-1.835078
H	3.152380	-4.565412	-0.318702
H	1.447288	-1.769064	-3.115830
H	0.865282	-3.646361	-4.632290
H	-5.426012	0.511208	0.496761
H	-1.394504	4.907468	1.132321
H	4.065310	0.956069	-0.587607
H	5.211227	-3.090986	-1.519704
H	6.364040	1.724719	-1.120946
H	7.499052	-2.322740	-2.043396
H	8.084875	0.084239	-1.847109
H	0.632522	-2.264170	1.233959
H	4.942110	-2.286878	1.400255
H	0.542786	-3.072588	3.550388
H	4.837874	-3.092997	3.723267
H	1.072517	5.065272	2.832890
H	1.839759	1.225718	1.032644
H	2.680667	4.531367	4.648619
H	3.449515	0.704363	2.835471
H	3.874575	2.351340	4.652086
H	-3.990320	-0.189637	2.646896
H	-2.102617	-2.247281	-0.633526
H	-3.630691	-2.165238	4.089375
H	-1.745240	-4.216815	0.809888
H	-2.504600	-4.189111	3.179317
H	-5.535051	-0.982382	-1.376348
H	-1.657519	0.503918	-2.528511
H	-5.885834	-1.832226	-3.670512
H	-2.004887	-0.376590	-4.827451
H	-4.126341	-1.540934	-5.400862
H	-0.144180	3.279260	-2.402398
H	2.817080	4.910803	0.261898
H	1.208204	4.014724	-4.352478
H	4.159905	5.646356	-1.678910
H	3.364407	5.198935	-3.990394
H	3.583936	-4.004500	5.582388
H	2.346848	-2.786996	5.916183

H	1.865667	-4.392584	5.373703

Table S143. XYZ Coordinates of H_para_I_OMe

67

scf done: -4806.596849

C	5.000383	-0.360459	-3.958228
C	4.869459	0.776578	-3.154429
C	4.175526	0.703739	-1.947021
C	3.605536	-0.509852	-1.525351
C	3.738427	-1.645053	-2.337299
C	4.435814	-1.568839	-3.546447
P	2.614368	-0.552650	0.021632
O	2.588300	-2.200993	0.281272
Pd	0.550214	0.466770	-0.048515
P	-1.520184	1.463140	-0.181658
C	-1.620300	3.116283	-0.975980
C	-2.756546	3.934359	-0.863830
C	-2.805233	5.164374	-1.519815
C	-1.718561	5.593357	-2.288718
C	-0.583270	4.788604	-2.403235
C	-0.534746	3.555026	-1.749755
O	-2.652811	0.636702	-1.089746
C	-2.425375	1.715183	1.401110
C	-3.717984	1.210779	1.599140
C	-4.366810	1.397642	2.823462
C	-3.732296	2.089195	3.856814
C	-2.440973	2.592203	3.666898
C	-1.788743	2.401411	2.449021
C	3.812300	0.043327	1.281919
C	5.152146	-0.380566	1.297287
C	6.017917	0.060116	2.297345
C	5.557801	0.933284	3.289239
C	4.229201	1.361325	3.281763
C	3.359331	0.915248	2.283265
C	-2.340544	-2.620284	-1.965172
C	-3.705915	-2.769039	-1.658402
C	-4.087964	-3.102408	-0.351131
C	-3.113880	-3.288503	0.634249
C	-1.769316	-3.139950	0.313198
C	-1.369933	-2.807628	-0.983037
O	-4.566894	-2.573004	-2.689163
Br	-0.444921	-3.401769	1.680279
C	-5.967523	-2.698336	-2.438798
H	-2.362253	-0.281293	-1.218250
H	1.721483	-2.459411	0.636731
H	-0.320609	-2.697086	-1.229762
H	5.518749	-1.051348	0.525637
H	2.322039	1.242052	2.272355
H	7.051792	-0.272712	2.302899
H	3.869593	2.039611	4.050011

H	6.235882	1.278518	4.064330
H	3.305278	-2.586211	-2.016191
H	4.084406	1.592759	-1.328028
H	4.537779	-2.456433	-4.164366
H	5.309187	1.719177	-3.467253
H	5.540227	-0.302952	-4.898833
H	-3.600705	3.612873	-0.261005
H	0.347313	2.924352	-1.832845
H	-3.687985	5.790554	-1.428568
H	0.263519	5.120166	-2.997030
H	-1.757223	6.554140	-2.793797
H	-4.209753	0.672933	0.795289
H	-0.780425	2.785787	2.313198
H	-5.368575	1.002752	2.967147
H	-1.940930	3.127163	4.469109
H	-4.237469	2.232496	4.807507
H	-2.055157	-2.375291	-2.983203
H	-3.414527	-3.547369	1.643193
H	-5.130586	-3.223852	-0.085330
H	-6.458848	-2.498568	-3.391013
H	-6.223171	-3.709312	-2.102369
H	-6.305715	-1.968701	-1.694533

Table S144. XYZ Coordinates of H_para_TS1_OMe

67

scf done: -4806.565865

C	3.089801	-0.760888	-2.489657
C	4.240437	-1.200062	-3.148082
C	5.397480	-0.418021	-3.128093
C	5.401325	0.804003	-2.449693
C	4.252028	1.243878	-1.791046
C	3.081972	0.465605	-1.804382
P	1.492116	1.013566	-1.040562
C	2.055324	2.141657	0.304358
C	1.553236	3.449303	0.395518
C	1.910427	4.273058	1.466378
C	2.766082	3.799598	2.462464
C	3.264840	2.495564	2.384350
C	2.911407	1.671196	1.316088
Pd	-0.210998	-0.546272	-0.486208
P	-2.343856	0.441084	-0.924908
O	-2.551656	0.791978	-2.545045
C	0.801555	-2.063615	0.632509
C	2.192979	-2.142404	0.848803
C	2.675798	-2.128221	2.145887
C	1.793719	-2.086612	3.249323
C	0.415577	-2.092125	3.033010
C	-0.084852	-2.131965	1.718400
O	2.392409	-2.077303	4.474799
C	1.557340	-2.046645	5.629387

Br	0.044743	-3.173460	-1.137923
O	0.908760	2.093585	-2.178258
C	-3.874604	-0.506165	-0.517777
C	-3.785178	-1.904068	-0.417794
C	-4.924499	-2.671317	-0.165042
C	-6.165030	-2.049228	-0.008911
C	-6.265699	-0.658386	-0.107783
C	-5.128350	0.109656	-0.359707
C	-2.661978	2.061890	-0.108711
C	-2.771693	2.119446	1.291604
C	-2.938108	3.343527	1.938359
C	-2.990492	4.528373	1.196662
C	-2.875424	4.480864	-0.193380
C	-2.708966	3.254610	-0.844185
H	3.744621	-2.143279	2.336837
H	2.879334	-2.170216	0.010378
H	-1.151200	-2.240901	1.555471
H	-0.284943	-2.115626	3.859139
H	-2.733462	1.205697	1.879646
H	-2.613258	3.227170	-1.924954
H	-3.027851	3.373423	3.020382
H	-2.914886	5.396973	-0.775701
H	-3.118361	5.481470	1.701253
H	3.306517	0.660794	1.269527
H	0.882398	3.824031	-0.370919
H	3.932575	2.120608	3.154724
H	1.518402	5.284723	1.519523
H	3.042733	4.440213	3.294635
H	-2.819229	-2.387591	-0.537818
H	-4.842320	-3.751502	-0.087470
H	-7.050903	-2.644601	0.192024
H	-7.228895	-0.171879	0.015527
H	-5.217219	1.190058	-0.424737
H	-3.449088	1.097840	-2.754699
H	4.269714	2.192179	-1.262579
H	6.299777	1.414105	-2.432020
H	6.294295	-0.759843	-3.636526
H	4.234056	-2.151965	-3.671016
H	2.193236	-1.375631	-2.496657
H	1.578013	2.737641	-2.461818
H	2.230270	-2.021501	6.487043
H	0.921651	-1.153297	5.639851
H	0.925188	-2.940376	5.690749

Table S145. XYZ Coordinates of H_para_IIa_OMe

67

scf done: -4806.640204

C	-1.787895	4.816943	-1.589422
C	-1.010442	3.803766	-2.156717
C	-0.634845	2.699115	-1.391131

C	-1.026103	2.608915	-0.046127
C	-1.808777	3.627707	0.519061
C	-2.188063	4.726555	-0.253812
P	-0.523236	1.165667	0.956182
C	1.008343	1.659082	1.825867
C	1.614166	0.720469	2.678336
C	2.754874	1.062150	3.403378
C	3.303510	2.342794	3.280783
C	2.706515	3.279039	2.434831
C	1.562494	2.941185	1.707900
Pd	-0.567095	-0.796540	-0.227230
Br	-1.045061	-3.005817	-1.462196
C	-2.580608	-0.731166	0.209113
C	-3.108293	-1.403700	1.329333
C	-4.482789	-1.437722	1.575842
C	-5.373359	-0.797133	0.702602
C	-4.869460	-0.119479	-0.414529
C	-3.486977	-0.088969	-0.644388
O	-6.698453	-0.887581	1.027180
C	-7.644452	-0.258875	0.167779
P	1.756256	-1.032407	-0.951797
O	1.776044	-1.887242	-2.365481
C	2.847372	-1.981022	0.175380
C	4.220545	-2.137455	-0.081072
C	5.006398	-2.928357	0.756359
C	4.431392	-3.572467	1.856520
C	3.067087	-3.429194	2.115485
C	2.277809	-2.639191	1.276652
C	2.758585	0.406297	-1.482469
C	2.648403	0.861690	-2.807647
C	3.356193	1.989148	-3.226413
C	4.175714	2.677581	-2.328397
C	4.285038	2.234104	-1.008846
C	3.579395	1.106532	-0.584527
O	-1.543519	1.184353	2.250901
H	4.676364	-1.638595	-0.930638
H	-2.296418	0.587573	2.076537
H	0.949022	-2.420314	-2.393977
H	-4.882433	-1.962952	2.438503
H	-2.446980	-1.926823	2.016457
H	-3.125969	0.452035	-1.515395
H	-5.532172	0.389256	-1.105336
H	1.214349	-2.537250	1.473598
H	6.066809	-3.042128	0.551900
H	2.616512	-3.931304	2.966347
H	5.046647	-4.185829	2.508174
H	2.026385	0.320877	-3.512807
H	3.680145	0.771361	0.442369
H	3.270883	2.325308	-4.255556
H	4.921087	2.763035	-0.305650

H	4.727844	3.553453	-2.656009
H	-2.123198	3.554293	1.554886
H	-0.039765	1.909353	-1.839977
H	-2.796465	5.510371	0.187260
H	-0.702800	3.868689	-3.195965
H	-2.085334	5.672463	-2.188433
H	1.195014	-0.276217	2.775870
H	1.105244	3.677282	1.055998
H	3.215295	0.330575	4.060302
H	3.128425	4.274845	2.339047
H	4.193305	2.608163	3.843773
H	-8.624871	-0.456864	0.602772
H	-7.484334	0.824792	0.116108
H	-7.607571	-0.676190	-0.845629

Table S146. XYZ Coordinates of H_para_IIb_OMe

67

scf done: -4806.641639

C	-5.975335	-1.189724	-1.222121
C	-5.639684	-2.366929	-1.894009
C	-4.298422	-2.671898	-2.143682
C	-3.295217	-1.803050	-1.716770
C	-3.624992	-0.614096	-1.043932
C	-4.973866	-0.312880	-0.799615
P	-2.264673	0.533333	-0.609522
O	-2.081358	1.428392	-1.988841
Pd	-0.254383	-0.423332	0.154858
P	1.823160	-1.269573	0.890810
O	1.615070	-2.327543	2.134971
C	0.769430	1.008825	-0.868041
C	0.890599	2.293727	-0.326843
C	1.581933	3.313872	-0.995840
C	2.174731	3.050872	-2.235929
C	2.063079	1.767815	-2.791327
C	1.367922	0.764347	-2.115202
O	2.875507	3.965322	-2.976224
C	3.017596	5.281335	-2.452914
Br	-1.516862	-2.194440	1.608320
C	3.049620	-0.119990	1.609439
C	3.178827	-0.027290	3.003832
C	4.076436	0.882190	3.567492
C	4.848972	1.706214	2.746845
C	4.721687	1.620667	1.357589
C	3.824614	0.716550	0.788547
C	2.737424	-2.234846	-0.368991
C	2.016121	-2.805022	-1.430835
C	2.659367	-3.614505	-2.368351
C	4.029423	-3.860988	-2.253697
C	4.754313	-3.300837	-1.198340
C	4.113633	-2.492278	-0.258738

C	-2.979231	1.711370	0.598852
C	-3.153198	3.063147	0.268645
C	-3.657486	3.957905	1.216022
C	-3.986658	3.511815	2.496936
C	-3.807004	2.166570	2.834170
C	-3.300621	1.269167	1.894523
H	1.648185	4.295218	-0.539904
H	0.440249	2.527456	0.634560
H	1.304763	-0.218244	-2.573423
H	2.527069	1.572953	-3.754175
H	-2.932895	1.663595	-2.391093
H	0.648483	-2.502504	2.218282
H	-2.891648	3.418544	-0.722485
H	-3.150466	0.227965	2.165596
H	-3.790708	5.002391	0.950507
H	-4.058040	1.816376	3.830982
H	-4.377779	4.208914	3.231969
H	-2.252619	-2.049924	-1.895437
H	-5.244706	0.600242	-0.279742
H	-4.034365	-3.588784	-2.661907
H	-7.016380	-0.951517	-1.025821
H	-6.420700	-3.047352	-2.220085
H	2.588124	-0.675629	3.641533
H	3.731078	0.663840	-0.291310
H	4.173316	0.941693	4.647521
H	5.320571	2.258854	0.714740
H	5.547988	2.411534	3.186528
H	0.950264	-2.613578	-1.521829
H	4.686034	-2.060283	0.555649
H	2.092809	-4.048049	-3.186952
H	5.819105	-3.492907	-1.106354
H	4.531684	-4.487399	-2.984913
H	3.602282	5.837249	-3.187139
H	2.044872	5.770695	-2.320845
H	3.550067	5.279232	-1.494000

Table S147. XYZ Coordinates of H_para_III_OMe

67

scf done: -4806.611725

C	-2.471544	-4.882421	0.829221
C	-2.152411	-4.063818	1.915854
C	-1.975850	-2.691757	1.732215
C	-2.121109	-2.126458	0.454224
C	-2.434903	-2.955758	-0.635841
C	-2.614687	-4.326260	-0.444731
P	-1.771102	-0.349895	0.160887
O	-2.650367	0.087676	-1.122063
Pd	0.527921	-0.233542	-0.240591
P	2.869551	-0.237894	-0.608079
C	3.874554	0.317596	0.813185

C	4.685240	1.458085	0.730027
C	5.398834	1.893123	1.849744
C	5.301995	1.199451	3.056941
C	4.486541	0.066824	3.148364
C	3.771440	-0.371239	2.034710
C	0.403078	1.725058	-0.631817
C	-0.091996	2.161056	-1.861279
C	-0.135152	3.528755	-2.168605
C	0.313641	4.468192	-1.232722
C	0.802593	4.028109	0.006561
C	0.848380	2.666591	0.305760
O	0.315796	5.821021	-1.427943
C	-0.163394	6.320183	-2.673062
C	-2.403102	0.533882	1.629106
C	-3.757366	0.888034	1.744045
C	-4.206205	1.548028	2.888665
C	-3.316979	1.852300	3.923447
C	-1.969776	1.501758	3.812923
C	-1.510834	0.852891	2.664827
O	3.321441	0.731933	-1.862807
C	3.483373	-1.907977	-1.026743
C	2.658660	-2.724221	-1.822348
C	3.086680	-3.994661	-2.207645
C	4.338054	-4.462268	-1.798378
C	5.163361	-3.657648	-1.008141
C	4.741411	-2.385020	-0.622209
Br	-5.702174	-0.518452	-1.337352
H	-0.523125	3.837895	-3.132127
H	-0.458225	1.450751	-2.595394
H	1.239710	2.352976	1.268341
H	1.147318	4.765192	0.725670
H	4.238768	0.586638	-2.146348
H	-3.636810	-0.143851	-1.123604
H	-4.458372	0.646557	0.948552
H	-0.458617	0.597697	2.571315
H	-5.253228	1.823751	2.973107
H	-1.274676	1.742214	4.611689
H	-3.673007	2.365797	4.811681
H	-2.557298	-2.526242	-1.624820
H	-1.740849	-2.062745	2.585579
H	-2.869869	-4.958002	-1.290225
H	-2.047802	-4.491204	2.908553
H	-2.611719	-5.949199	0.975374
H	4.757773	2.007343	-0.202837
H	3.140974	-1.252858	2.116556
H	6.027935	2.774941	1.775820
H	4.407468	-0.474516	4.086168
H	5.856567	1.540295	3.925938
H	1.684236	-2.363170	-2.141890
H	5.388042	-1.768185	-0.006977

H	2.443860	-4.618383	-2.821056
H	6.136377	-4.020651	-0.691565
H	4.669498	-5.453273	-2.093530
H	-0.069346	7.405418	-2.618820
H	0.434534	5.946845	-3.513138
H	-1.215289	6.056072	-2.834926

Table S148. XYZ Coordinates of H_para_IV_OMe

92

scf done: -5687.150901

C	0.862869	3.688496	-0.973274
C	1.185476	2.377343	-1.351616
C	1.310636	2.066797	-2.715238
C	1.119300	3.053732	-3.682646
C	0.798417	4.358344	-3.299206
C	0.671492	4.673082	-1.944376
P	1.395420	1.033710	-0.121201
C	2.040676	1.869185	1.375882
C	3.222432	2.629578	1.323860
C	3.702285	3.250968	2.476568
C	3.009338	3.127332	3.685019
C	1.839436	2.368322	3.744702
C	1.360707	1.735557	2.595337
Pd	-0.439526	-0.386270	0.305535
P	-2.515638	-1.521632	0.454702
C	-3.865376	-0.829316	1.479697
C	-3.996488	-1.254640	2.812009
C	-4.991833	-0.713051	3.627537
C	-5.860289	0.256882	3.123313
C	-5.728934	0.689267	1.801129
C	-4.734170	0.155610	0.981750
P	0.941715	-2.316691	0.840209
O	0.238011	-3.316165	1.945858
C	1.277008	-3.401822	-0.601124
C	1.607243	-2.846742	-1.848256
C	1.838629	-3.679463	-2.945035
C	1.728159	-5.065786	-2.810125
C	1.390770	-5.621391	-1.573272
C	1.167091	-4.794894	-0.470698
C	2.555715	-2.116274	1.687552
C	3.777983	-2.355256	1.045364
C	4.978661	-2.189803	1.738327
C	4.969241	-1.785617	3.075033
C	3.753635	-1.544603	3.720590
C	2.551952	-1.704295	3.030224
C	-1.645796	1.265659	-0.091415
C	-2.156607	1.524962	-1.374991
C	-2.971670	2.628527	-1.624625
C	-3.289862	3.521371	-0.590469
C	-2.786701	3.287392	0.693954

C	-1.979773	2.164820	0.928053
O	-4.088953	4.576774	-0.935537
C	-4.443539	5.509026	0.080615
O	2.593408	0.128063	-0.750398
O	-2.304857	-3.028064	1.157718
C	-3.255983	-1.915532	-1.168520
C	-4.613204	-2.251777	-1.307344
C	-5.115623	-2.634676	-2.551333
C	-4.271711	-2.686668	-3.663610
C	-2.919924	-2.359377	-3.532601
C	-2.412642	-1.976162	-2.290947
Br	5.258563	1.430700	-1.797450
H	-3.367290	2.817625	-2.618582
H	-1.926164	0.860843	-2.202791
H	-1.619280	2.006965	1.940759
H	-3.014026	3.956798	1.515790
H	3.423369	0.612228	-1.061629
H	-0.732382	-3.347939	1.795404
H	3.776533	2.718184	0.392839
H	0.459882	1.131285	2.646557
H	4.618118	3.832860	2.431956
H	1.303458	2.262056	4.683082
H	3.385062	3.617310	4.578486
H	1.575412	1.059471	-3.020210
H	0.763942	3.945889	0.075856
H	1.226997	2.804496	-4.734173
H	0.428096	5.686892	-1.640509
H	0.651440	5.126578	-4.052608
H	0.901268	-5.225364	0.489281
H	1.701284	-1.770680	-1.952608
H	1.301765	-6.698445	-1.465716
H	2.101278	-3.244524	-3.904901
H	1.901923	-5.710800	-3.666395
H	-5.278858	-2.211488	-0.451518
H	-1.361088	-1.722865	-2.191129
H	-6.165885	-2.890735	-2.650931
H	-2.261612	-2.400264	-4.394843
H	-4.667173	-2.982060	-4.630751
H	-3.331143	-2.013600	3.210208
H	-4.636165	0.508480	-0.039279
H	-5.088416	-1.053634	4.653916
H	-6.400647	1.444624	1.404904
H	-6.636026	0.675130	3.757634
H	3.796869	-2.672453	0.008663
H	1.611943	-1.518234	3.540388
H	5.920581	-2.380972	1.232961
H	3.739706	-1.231404	4.760287
H	5.904704	-1.659491	3.612064
H	-3.095879	-3.591395	1.171733
H	-5.074397	6.256549	-0.402124

H	-5.007035	5.028533	0.889621
H	-3.559119	6.001178	0.502895

Table S149. XYZ Coordinates of H_para_V_OMe

91

scf done: -5686.697246

C	-1.466810	-2.136357	-2.689282
C	-1.380408	-3.176350	-3.615396
C	-1.219741	-4.493510	-3.177674
C	-1.149835	-4.766256	-1.809675
C	-1.239880	-3.727641	-0.880648
C	-1.399985	-2.403099	-1.312324
P	-1.467984	-0.991834	-0.135460
O	-2.592555	-0.015152	-0.806803
Pd	0.510305	0.251700	0.328605
P	-0.686044	2.295631	0.898728
C	-0.901784	3.421999	-0.540277
C	-0.521779	4.767764	-0.429266
C	-0.644094	5.626878	-1.523540
C	-1.146314	5.150448	-2.736956
C	-1.522379	3.809555	-2.855380
C	-1.395299	2.944661	-1.766150
C	1.600156	-1.462598	-0.116260
C	1.913466	-1.825279	-1.439329
C	2.631579	-2.983051	-1.739067
C	3.046477	-3.841292	-0.711361
C	2.737445	-3.516938	0.613518
C	2.032309	-2.335429	0.890795
O	3.738164	-4.959918	-1.103492
C	4.179727	-5.857570	-0.092819
P	2.561238	1.382619	0.758961
O	2.444328	2.414388	1.935640
C	3.094717	2.321943	-0.740798
C	3.605531	3.618176	-0.576813
C	4.013823	4.362667	-1.685397
C	3.914315	3.820881	-2.969840
C	3.398464	2.533863	-3.142896
C	2.983687	1.790890	-2.034955
C	4.061356	0.390236	1.178684
C	4.369726	0.209769	2.535475
C	5.490764	-0.531146	2.916666
C	6.318875	-1.098422	1.945493
C	6.022208	-0.917764	0.591711
C	4.900744	-0.179039	0.210215
O	0.105654	3.179517	2.000696
C	-2.350174	2.215880	1.684036
C	-3.532223	2.589339	1.031036
C	-4.759771	2.510631	1.692863
C	-4.820218	2.057839	3.012365
C	-3.646096	1.682683	3.671039

C	-2.419708	1.759131	3.010602
C	-2.210347	-1.726240	1.375401
C	-3.532490	-2.202687	1.386711
C	-4.063841	-2.757575	2.551397
C	-3.284245	-2.851024	3.708723
C	-1.971435	-2.376390	3.704827
C	-1.439530	-1.808904	2.544459
Br	-5.383845	-1.072920	-1.879648
H	2.869152	-3.244959	-2.766721
H	1.579077	-1.207789	-2.269540
H	1.834421	-2.105348	1.935116
H	3.042629	-4.158076	1.433206
H	-3.448703	-0.438274	-1.118765
H	1.125053	2.887148	2.023719
H	-4.150194	-2.128915	0.494367
H	-0.424286	-1.422957	2.544091
H	-5.088138	-3.119078	2.555784
H	-1.364568	-2.438875	4.603390
H	-3.702047	-3.287507	4.611352
H	-1.603764	-1.117115	-3.036938
H	-1.191619	-3.953726	0.179626
H	-1.442330	-2.957946	-4.677577
H	-1.031341	-5.788760	-1.462971
H	-1.153154	-5.302945	-3.898774
H	-0.126148	5.131723	0.513345
H	-1.690447	1.904061	-1.860671
H	-0.346416	6.667313	-1.428156
H	-1.912040	3.434739	-3.797654
H	-1.240707	5.819386	-3.587514
H	3.672364	4.036606	0.422869
H	2.573362	0.796280	-2.176945
H	4.407659	5.365842	-1.547311
H	3.312677	2.110443	-4.139716
H	4.231207	4.400769	-3.831928
H	3.738105	0.667965	3.289885
H	4.686165	-0.045231	-0.844967
H	5.719434	-0.659522	3.971086
H	6.666919	-1.348765	-0.169196
H	7.192701	-1.672456	2.240344
H	-3.499697	2.945654	0.007325
H	-1.511987	1.469935	3.531845
H	-5.668058	2.807111	1.176194
H	-3.684398	1.330683	4.697909
H	-5.775801	1.998508	3.525291
H	4.700677	-6.664887	-0.609931
H	4.871794	-5.371534	0.606134
H	3.338210	-6.276426	0.472993

Table S150. XYZ Coordinates of H_para_TS2_OMe

91
scf done: -5686.660280

C	-2.965029	1.129217	2.514217
C	-1.827478	1.926546	2.726256
C	-1.565870	2.416353	4.014032
C	-2.430014	2.118922	5.071470
C	-3.563055	1.332345	4.853551
C	-3.828393	0.839743	3.572140
P	-0.675624	2.286834	1.328439
C	-1.660020	3.478350	0.318948
C	-1.512237	4.861918	0.497938
C	-2.261687	5.755610	-0.269990
C	-3.166655	5.276759	-1.221151
C	-3.315189	3.899695	-1.406222
C	-2.561606	3.001723	-0.646236
Pd	0.364065	0.375819	0.372536
P	-1.318128	-1.220652	-0.355309
O	-2.746445	-0.459025	-0.671394
P	2.556616	1.337893	0.146685
C	4.316383	0.921193	-0.297703
C	5.261314	0.900648	0.740421
C	6.598716	0.601036	0.478294
C	7.011636	0.313136	-0.825572
C	6.079533	0.334011	-1.864598
C	4.740598	0.637936	-1.603567
C	2.010300	2.184114	-1.409321
C	1.533664	1.496415	-2.538886
C	1.131009	2.202467	-3.674736
C	1.192038	3.598427	-3.693757
C	1.658306	4.287658	-2.571378
C	2.064462	3.586664	-1.433985
O	2.711753	2.330782	1.325245
C	2.199516	-0.746615	0.645000
C	2.454241	-0.928373	2.021648
C	2.975636	-2.124268	2.506346
C	3.283093	-3.169031	1.620823
C	3.067139	-2.995748	0.243613
C	2.543111	-1.792624	-0.229376
O	3.795562	-4.296105	2.185724
C	4.138427	-5.388326	1.334284
C	-0.945412	-2.076789	-1.948348
C	-1.241520	-1.433314	-3.162129
C	-0.877690	-2.011014	-4.379750
C	-0.203314	-3.234896	-4.403650
C	0.100442	-3.879795	-3.202254
C	-0.266785	-3.305798	-1.982996
C	-1.747557	-2.626212	0.759908
C	-0.867289	-2.945532	1.805417
C	-1.150538	-4.004424	2.673303

C	-2.324067	-4.742668	2.513576
C	-3.212813	-4.422353	1.481968
C	-2.928079	-3.374088	0.606330
O	0.422208	3.264119	2.055399
Br	-5.273938	-1.807212	-2.110989
H	3.297786	-3.786414	-0.460634
H	2.386964	-1.686791	-1.298308
H	2.249348	-0.120759	2.718725
H	3.163694	-2.264815	3.566433
H	-3.464886	-0.983149	-1.127179
H	1.351612	2.931603	1.811718
H	-3.629738	-3.134868	-0.189835
H	0.040021	-2.364325	1.941051
H	-4.129537	-4.992409	1.358656
H	-0.458470	-4.244531	3.475496
H	-2.549452	-5.561409	3.191052
H	-1.774989	-0.487892	-3.153362
H	-0.031623	-3.822596	-1.057612
H	-1.125638	-1.507055	-5.309718
H	0.617659	-4.835158	-3.212515
H	0.079208	-3.684208	-5.351246
H	-0.805354	5.231106	1.233995
H	-2.673986	1.932031	-0.804479
H	-2.138855	6.825548	-0.125472
H	-4.012960	3.522813	-2.148998
H	-3.748571	5.972995	-1.818425
H	2.419908	4.123039	-0.559697
H	1.468326	0.412741	-2.538186
H	1.707251	5.372849	-2.580135
H	0.767492	1.659776	-4.542526
H	0.876967	4.145247	-4.577538
H	4.946133	1.132765	1.752326
H	4.036274	0.663630	-2.428410
H	7.318838	0.595418	1.291825
H	6.392636	0.120764	-2.882826
H	8.052579	0.079849	-1.030104
H	-3.173587	0.726888	1.526460
H	-0.686824	3.029838	4.180503
H	-4.706589	0.224542	3.396718
H	-2.217268	2.504447	6.064871
H	-4.233817	1.101431	5.676166
H	4.523791	-6.169293	1.990356
H	3.261959	-5.766769	0.795913
H	4.913340	-5.105513	0.612613

Table S151. XYZ Coordinates of H_para_VI_OMe

91

scf done: -5686.691250

C	-2.113844	0.498371	3.081122
C	-2.637811	-0.573491	2.342728

C	-2.718813	-1.842743	2.938365
C	-2.263071	-2.038788	4.243196
C	-1.737280	-0.967256	4.970602
C	-1.667218	0.301463	4.389947
P	-3.253917	-0.228985	0.652323
C	-4.799158	-1.206900	0.493847
C	-6.011475	-0.580629	0.823895
C	-7.214475	-1.282887	0.740259
C	-7.218137	-2.617703	0.326499
C	-6.017059	-3.247007	-0.009089
C	-4.812132	-2.545122	0.070244
C	-2.095018	-0.963880	-0.547554
C	-1.147823	-1.984512	-0.197328
C	-0.681549	-2.885828	-1.193356
C	-1.070379	-2.750668	-2.514676
C	-1.983783	-1.723116	-2.885121
C	-2.487256	-0.875336	-1.923688
O	-0.551792	-3.654478	-3.399178
C	-0.970017	-3.598564	-4.760562
Pd	-0.002427	0.031232	-0.233216
P	0.131200	2.295941	-0.889834
O	1.665056	2.637719	-1.417713
O	-3.538411	1.247326	0.446582
P	2.226002	-0.553168	0.228215
C	2.886143	-2.185159	-0.319674
C	3.363154	-2.351745	-1.629847
C	3.740821	-3.614243	-2.092324
C	3.637268	-4.729601	-1.257877
C	3.157004	-4.575085	0.045900
C	2.782785	-3.313463	0.511255
C	2.829749	-0.448233	1.967387
C	4.196400	-0.465188	2.298040
C	4.595649	-0.383983	3.632198
C	3.640800	-0.294690	4.650922
C	2.282565	-0.275474	4.331253
C	1.879832	-0.344342	2.994394
O	3.184884	0.513786	-0.619716
C	-0.122145	3.640913	0.358617
C	-1.352060	3.761155	1.029161
C	-1.534914	4.761173	1.986784
C	-0.494160	5.641458	2.298207
C	0.732984	5.518342	1.643453
C	0.919044	4.524725	0.677976
C	-0.832822	2.946324	-2.328866
C	-0.189272	3.491500	-3.450220
C	-0.935215	3.957108	-4.536539
C	-2.329791	3.885905	-4.514930
C	-2.976695	3.339224	-3.402654
C	-2.236219	2.864053	-2.318335
Br	6.354661	0.363300	-0.847036

H	-1.394000	-1.119151	5.989723
H	-2.330483	-3.023915	4.695045
H	-3.151372	-2.677561	2.395523
H	-2.069376	1.484873	2.632457
H	-1.270752	1.139196	4.956019
H	4.179816	0.383704	-0.622360
H	2.273721	1.904357	-1.167361
H	-6.007973	0.460041	1.132741
H	-3.886553	-3.040804	-0.206795
H	-8.147534	-0.788430	0.994233
H	-6.017279	-4.281737	-0.338905
H	-8.154540	-3.163982	0.260603
H	-1.005477	-2.261488	0.841590
H	-3.210604	-0.119786	-2.215898
H	-0.003443	-3.687873	-0.921124
H	-2.305089	-1.611055	-3.913455
H	0.893547	3.550689	-3.466308
H	-2.749784	2.420107	-1.468623
H	-0.424548	4.378084	-5.398493
H	-4.061407	3.275089	-3.382120
H	-2.908426	4.248436	-5.359881
H	3.457192	-1.489547	-2.282430
H	2.418398	-3.207346	1.529451
H	4.118659	-3.724589	-3.104929
H	3.079682	-5.436212	0.703757
H	3.930425	-5.711098	-1.618846
H	4.948850	-0.523963	1.515070
H	0.822524	-0.312013	2.744835
H	5.653669	-0.391777	3.879042
H	1.535376	-0.200826	5.115837
H	3.957162	-0.235885	5.688502
H	-2.162182	3.069010	0.811375
H	1.870262	4.433249	0.164104
H	-2.491125	4.849008	2.495809
H	1.547728	6.197250	1.880558
H	-0.638261	6.414166	3.048071
H	-0.449430	-4.412656	-5.266323
H	-2.051828	-3.747767	-4.857675
H	-0.691865	-2.648101	-5.230686

Table S152. XYZ Coordinates of H_para_VII_OMe

91

scf done: -5686.695529

C	0.382222	2.659952	2.413526
C	1.453760	2.946228	1.553467
C	1.707222	4.278088	1.189742
C	0.904846	5.307108	1.687538
C	-0.158844	5.014252	2.544972
C	-0.421531	3.689794	2.905261
P	2.478704	1.546307	0.977237

O	1.710695	0.237556	1.011259
C	3.072185	2.004358	-0.678837
C	2.130112	2.358908	-1.667125
C	2.529253	2.622063	-2.968035
C	3.888112	2.534037	-3.322542
C	4.836562	2.179167	-2.353514
C	4.421036	1.916843	-1.045811
O	4.176752	2.812885	-4.618630
C	5.537140	2.736831	-5.046845
C	3.942410	1.480575	2.073501
C	4.346127	0.222689	2.544127
C	5.453388	0.109092	3.387547
C	6.162878	1.250471	3.766438
C	5.763473	2.508051	3.303860
C	4.657089	2.624895	2.462230
C	3.314542	-1.950401	-2.303049
C	3.622786	-2.800127	-3.370272
C	2.845310	-3.936837	-3.599939
C	1.763623	-4.228557	-2.763876
C	1.453214	-3.382494	-1.689596
C	2.232780	-2.233728	-1.467868
P	0.021155	-3.707972	-0.581825
C	0.809841	-4.458328	0.900283
C	0.201167	-4.258266	2.149327
C	0.733831	-4.843190	3.300257
C	1.882171	-5.633016	3.212687
C	2.496870	-5.836903	1.973372
C	1.966372	-5.251406	0.823428
Pd	-1.489495	-2.012563	-0.289051
P	-3.165056	-0.416200	-0.148035
C	-3.471826	0.280272	1.530546
C	-4.564570	1.118771	1.811599
C	-4.752652	1.615661	3.101718
C	-3.852783	1.289777	4.122295
C	-2.765642	0.455960	3.852278
C	-2.581043	-0.051511	2.562691
C	-2.883273	1.094789	-1.170358
C	-2.183862	2.202727	-0.666202
C	-1.890549	3.291383	-1.491260
C	-2.290387	3.286820	-2.830195
C	-2.988109	2.188681	-3.339976
C	-3.279534	1.098676	-2.517930
O	-4.616093	-0.977116	-0.680890
O	-0.637888	-5.079665	-1.285231
Br	-7.142940	0.949231	-1.128480
H	-0.784762	5.815633	2.926333
H	1.106155	6.334571	1.399520
H	2.517784	4.514951	0.507251
H	0.171562	1.629250	2.679800
H	-1.254349	3.455477	3.561516

H	-5.347264	-0.302418	-0.782350
H	-1.519726	-4.855396	-1.615774
H	3.783929	-0.658098	2.250994
H	4.350352	3.608114	2.118543
H	5.758848	-0.868361	3.748914
H	6.310681	3.397415	3.601775
H	7.022987	1.162756	4.423741
H	1.075785	2.434312	-1.416544
H	5.169322	1.644806	-0.308603
H	1.808313	2.899491	-3.730135
H	5.888620	2.107096	-2.600289
H	1.155973	-5.109398	-2.943500
H	1.995934	-1.555562	-0.649919
H	3.077886	-4.597984	-4.430098
H	3.910761	-1.059454	-2.126899
H	4.461106	-2.572953	-4.022696
H	-3.832389	0.254616	-2.918791
H	-1.872776	2.225524	0.373647
H	-3.309448	2.181101	-4.377662
H	-1.356794	4.145061	-1.083169
H	-2.064348	4.134704	-3.470235
H	-5.275879	1.370969	1.028158
H	-1.743814	-0.712016	2.348017
H	-5.602637	2.258514	3.312822
H	-2.068780	0.192936	4.642931
H	-4.004230	1.679076	5.125090
H	-0.691026	-3.639836	2.211765
H	2.454784	-5.408455	-0.133616
H	0.255485	-4.679705	4.261582
H	3.391402	-6.449158	1.904027
H	2.300188	-6.086168	4.106964
H	5.530756	2.994777	-6.105928
H	6.166891	3.449289	-4.502495
H	5.938329	1.725088	-4.921006

Table S153. XYZ Coordinates of H_meta_I_P(O)Ph$_2$

86

scf done: -5571.384987

P	-1.555800	-2.386980	-0.112318
O	0.092860	-2.497128	-0.349127
Pd	-2.404506	-0.244983	0.002258
P	-3.319484	1.867612	0.143926
C	-4.246792	2.274711	1.675517
C	-5.121039	3.370770	1.758481
C	-5.769165	3.663745	2.958482
C	-5.556207	2.863236	4.085265
C	-4.691825	1.769041	4.011325
C	-4.040486	1.475813	2.811146
C	-2.203415	-3.475554	-1.443125
C	-2.002393	-4.865935	-1.441176

C	-2.471532	-5.645640	-2.498076
C	-3.150290	-5.047121	-3.564766
C	-3.357237	-3.666368	-3.574442
C	-2.885106	-2.883980	-2.517831
C	-1.749386	-3.421664	1.393407
C	-0.645945	-3.963346	2.066823
C	-0.832494	-4.718331	3.228835
C	-2.117935	-4.937512	3.726645
C	-3.223040	-4.397122	3.060820
C	-3.040407	-3.640292	1.903993
O	-2.232332	3.134992	0.105330
C	-4.490569	2.339976	-1.192325
C	-4.360421	3.543476	-1.899354
C	-5.261082	3.860079	-2.920546
C	-6.296915	2.980779	-3.241642
C	-6.429952	1.776743	-2.542552
C	-5.528416	1.454580	-1.528184
C	4.583122	1.877092	-0.899341
C	4.715155	2.773164	-1.970891
C	3.600725	3.134841	-2.727684
C	2.340902	2.611250	-2.426195
C	2.225471	1.729995	-1.354448
C	3.320741	1.355380	-0.581354
H	5.688636	3.193975	-2.199933
Br	0.477523	1.005515	-0.913122
H	-1.368025	2.802068	-0.185411
H	0.449696	-1.606141	-0.493870
H	3.190466	0.680409	0.256961
H	-1.483830	-5.339237	-0.612557
H	-3.044169	-1.808161	-2.517369
H	-2.311947	-6.719934	-2.489293
H	-3.886144	-3.199117	-4.399960
H	-3.518626	-5.657837	-4.383934
H	0.352931	-3.793830	1.679517
H	-3.904507	-3.216908	1.397330
H	0.029021	-5.135012	3.742814
H	-4.225406	-4.561457	3.445393
H	-2.260144	-5.522862	4.630316
H	-5.299159	3.992101	0.885924
H	-3.369358	0.622714	2.745319
H	-6.443243	4.513521	3.014413
H	-4.527017	1.143190	4.883479
H	-6.066431	3.091105	5.016617
H	-3.554267	4.225385	-1.649628
H	-5.627571	0.509952	-0.998173
H	-5.151871	4.794499	-3.463630
H	-7.230353	1.086551	-2.792943
H	-6.994735	3.228118	-4.036251
P	6.100298	1.452640	0.040833
H	1.471030	2.889007	-3.010808

H	3.706816	3.829380	-3.555133
C	5.549984	0.975765	1.715994
C	6.788389	-0.046401	-0.744505
O	7.084258	2.598432	0.060762
C	5.558680	1.972683	2.704671
C	5.136199	1.679419	4.001523
C	4.702722	0.389970	4.321244
C	4.699090	-0.608780	3.344486
C	5.124980	-0.320423	2.046259
H	5.907807	2.969879	2.455802
H	5.148030	2.454732	4.761690
H	4.374641	0.162193	5.331105
H	4.372515	-1.613975	3.593344
H	5.134542	-1.108453	1.299518
C	8.177074	-0.235613	-0.658411
C	8.768737	-1.365194	-1.224362
C	7.979898	-2.313138	-1.881503
C	6.598831	-2.127204	-1.978098
C	6.003148	-0.996456	-1.415689
H	8.789336	0.509280	-0.159784
H	9.843593	-1.503371	-1.155796
H	8.441182	-3.191541	-2.323045
H	5.985034	-2.857381	-2.496801
H	4.931214	-0.854724	-1.512663

Table S154. XYZ Coordinates of H_meta_TS1_P(O)Ph_2

86

scf done: -5571.356290

C	2.607898	3.513830	2.134743
C	1.484357	3.110128	1.399817
C	0.248997	2.978631	2.057576
C	0.140203	3.258188	3.419349
C	1.264654	3.665888	4.144497
C	2.496251	3.792643	3.500182
P	1.618142	2.637707	-0.373703
O	3.133871	3.186676	-0.800737
Pd	1.330879	0.304083	-0.863974
Br	0.852881	-0.411236	-3.484038
C	0.040542	-1.200407	-1.622538
C	0.163905	-2.594726	-1.438427
C	-0.829474	-3.277130	-0.752048
C	-1.981305	-2.612918	-0.271789
C	-2.122802	-1.242537	-0.527779
C	-1.150189	-0.537761	-1.245486
H	-2.806767	-3.128939	0.200603
P	2.946451	-0.898526	0.388737
C	2.410530	-1.622911	1.995546
C	2.489758	-0.848694	3.165434
C	2.010370	-1.345877	4.379311
C	1.440136	-2.619231	4.441162

C	1.350683	-3.394191	3.280987
C	1.827042	-2.900074	2.065481
C	3.920979	-2.265067	-0.377237
C	3.948222	-2.350970	-1.778671
C	4.726669	-3.319970	-2.416223
C	5.489411	-4.211431	-1.659441
C	5.472486	-4.133449	-0.263626
C	4.692640	-3.168581	0.374654
O	4.130420	0.173573	0.879610
C	0.492134	3.844202	-1.196285
C	-0.107204	3.470591	-2.410655
C	-0.924559	4.366314	-3.103174
C	-1.152982	5.644415	-2.588728
C	-0.561269	6.026799	-1.381424
C	0.256031	5.133427	-0.687976
H	-0.706915	-4.341860	-0.580814
H	1.044095	-3.117604	-1.795288
H	-1.331080	0.489840	-1.540643
P	-3.609002	-0.369037	-0.009891
H	-0.632819	2.663985	1.504952
H	3.570711	3.607799	1.642866
H	-0.821417	3.159192	3.914400
H	3.373393	4.108993	4.057273
H	1.179411	3.881712	5.205371
H	1.756642	-3.517730	1.175801
H	2.934792	0.140754	3.128029
H	0.921370	-4.390910	3.323290
H	2.087408	-0.738552	5.276498
H	1.072378	-3.007875	5.386092
H	0.066700	2.475150	-2.810852
H	-1.384195	4.064850	-4.039810
H	-1.792310	6.340162	-3.124205
H	-0.739130	7.019315	-0.977923
H	0.702738	5.439110	0.253328
H	3.230474	4.147664	-0.700967
H	4.681594	-3.126472	1.459711
H	6.064725	-4.825605	0.327785
H	6.094090	-4.966172	-2.153681
H	4.735035	-3.378433	-3.500657
H	3.351394	-1.661361	-2.369446
H	4.813490	-0.243709	1.429432
O	-3.489339	1.266938	-0.456284
C	-5.093593	-1.129315	-0.833034
C	-3.791074	-0.496092	1.836811
C	-6.371960	-0.616167	-0.583118
C	-7.489394	-1.188419	-1.202689
C	-7.328462	-2.273819	-2.072175
C	-6.050096	-2.786967	-2.322092
C	-4.932661	-2.214715	-1.702521
H	-6.497142	0.228120	0.093218

H	-8.483781	-0.789263	-1.008290
H	-8.197667	-2.718949	-2.554113
H	-5.924914	-3.631254	-2.998428
H	-3.938275	-2.613871	-1.896920
C	-4.879428	0.109432	2.476208
C	-5.016472	0.013799	3.866198
C	-4.065163	-0.687357	4.616792
C	-2.976809	-1.292881	3.977395
C	-2.839765	-1.197248	2.587404
H	-5.619410	0.654831	1.892353
H	-5.863055	0.484810	4.363557
H	-4.171763	-0.761745	5.698005
H	-2.236827	-1.838280	4.561249
H	-1.993182	-1.668259	2.090045

Table S155. XYZ Coordinates of H_meta_IIa_P(O)Ph$_2$

86

scf done: -5571.429455

C	-5.468258	2.007007	2.314762
C	-4.678421	2.589537	3.306963
C	-3.386639	2.107881	3.541489
C	-2.888694	1.048791	2.783920
C	-3.681216	0.453081	1.787236
C	-4.974068	0.942451	1.556929
P	-2.971810	-0.952668	0.851365
O	-2.820202	-2.022049	2.101677
Pd	-0.881157	-0.879279	-0.380092
Br	-0.685910	-3.384846	0.163897
C	0.906741	-1.083356	-1.384100
C	0.918276	-1.456361	-2.740376
C	2.122879	-1.709834	-3.411572
C	3.338438	-1.588284	-2.741628
C	3.350911	-1.205726	-1.391067
C	2.139801	-0.958928	-0.723755
H	4.272715	-1.798914	-3.252274
P	-0.733366	1.332006	-0.986160
C	0.280452	2.320549	0.170261
C	0.694614	3.615355	-0.188444
C	1.478531	4.365579	0.687153
C	1.853202	3.833065	1.925532
C	1.447791	2.546807	2.286129
C	0.667811	1.789408	1.408624
C	-4.355000	-1.613706	-0.151552
C	-5.319437	-2.454365	0.427127
C	-6.359062	-2.966453	-0.350005
C	-6.446101	-2.642605	-1.707084
C	-5.487800	-1.809836	-2.289530
C	-4.441125	-1.301963	-1.517225
O	-0.038936	1.559203	-2.463682
C	-2.245652	2.320347	-1.269108

C	-2.930458	2.170916	-2.487701
C	-4.087963	2.907464	-2.736211
C	-4.575361	3.794278	-1.771792
C	-3.902686	3.941146	-0.557685
C	-2.742616	3.206681	-0.302965
H	-5.247280	-2.712604	1.478814
H	0.554988	0.813646	-2.666541
H	-2.164205	-2.699786	1.823695
H	2.105678	-2.011332	-4.455471
H	-0.018206	-1.576322	-3.280246
H	2.159460	-0.683466	0.326261
P	4.983355	-1.055521	-0.579064
H	-3.692870	-0.664204	-1.978268
H	-7.099941	-3.618697	0.102767
H	-5.549000	-1.562157	-3.345023
H	-7.255682	-3.043264	-2.309905
H	-1.885423	0.680177	2.975369
H	-5.599880	0.493688	0.793205
H	-2.768473	2.556048	4.313624
H	-6.472712	2.376141	2.130503
H	-5.065860	3.415042	3.896655
H	0.405136	4.034342	-1.147200
H	0.364224	0.782917	1.682016
H	1.793953	5.365868	0.406307
H	1.740945	2.129539	3.244569
H	2.462138	4.420897	2.605752
H	-2.546165	1.498896	-3.248296
H	-2.228176	3.331951	0.643401
H	-4.604682	2.793426	-3.684358
H	-4.277049	4.628188	0.194650
H	-5.475320	4.369377	-1.968066
C	4.713040	-1.384454	1.199751
O	6.010244	-1.988024	-1.180417
C	5.482652	0.697632	-0.723786
C	4.904910	-2.702553	1.644004
C	4.698027	-3.031085	2.984155
C	4.300585	-2.046081	3.892474
C	4.117039	-0.730526	3.459555
C	4.325098	-0.397900	2.119126
H	5.227181	-3.462732	0.939203
H	4.850463	-4.052564	3.319728
H	4.141523	-2.301494	4.935972
H	3.819446	0.039199	4.165479
H	4.190201	0.630211	1.797893
C	6.857159	0.968832	-0.812556
C	7.309473	2.284874	-0.915240
C	6.393493	3.340022	-0.930623
C	5.023832	3.077157	-0.849992
C	4.567371	1.761110	-0.751542
H	7.564099	0.145032	-0.813736

H	8.374256	2.486254	-0.986133
H	6.745821	4.364189	-1.011107
H	4.308128	3.893381	-0.867283
H	3.499703	1.569554	-0.704766

Table S156. XYZ Coordinates of H_meta_IIb_P(O)Ph$_2$

86

scf done: -5571.432189

C	1.405944	4.142789	1.255819
C	0.517102	3.053946	1.261325
C	0.366556	2.296237	2.433369
C	1.098257	2.614981	3.579189
C	1.981454	3.696115	3.563720
C	2.132547	4.460583	2.402684
P	-0.525484	2.666640	-0.191952
Pd	-1.678491	0.612426	-0.040460
Br	-3.824610	1.883658	0.727321
C	0.528329	3.000265	-1.645907
C	1.864660	2.570778	-1.685781
C	2.634707	2.777594	-2.830569
C	2.079075	3.408912	-3.946467
C	0.749098	3.834250	-3.914356
C	-0.026272	3.629683	-2.771360
O	-1.591679	3.916432	-0.300008
P	-2.773875	-1.473341	0.041691
C	-3.818787	-1.804853	1.507927
C	-4.957180	-2.624297	1.444554
C	-5.673451	-2.919517	2.606054
C	-5.258558	-2.404138	3.836084
C	-4.124564	-1.590215	3.907148
C	-3.409296	-1.288504	2.748782
C	-0.013962	-0.323475	-0.730110
C	0.077963	-0.675602	-2.085641
C	1.203260	-1.343980	-2.579269
C	2.265462	-1.660680	-1.731635
C	2.197862	-1.301889	-0.377191
C	1.057845	-0.641507	0.114311
P	3.569555	-1.573014	0.797337
C	4.792466	-0.248840	0.474561
C	5.218556	0.522311	1.564943
C	6.136399	1.559089	1.379166
C	6.633571	1.831596	0.103577
C	6.211910	1.067275	-0.989392
C	5.294025	0.032579	-0.806904
O	-1.720641	-2.748601	0.026750
C	-3.850632	-1.773540	-1.410476
C	-3.529877	-2.775889	-2.338019
C	-4.327516	-2.967443	-3.469027
C	-5.444652	-2.159118	-3.684634
C	-5.764042	-1.152762	-2.767678

C	-4.970972	-0.954679	-1.637893
O	3.104853	-1.546656	2.238288
C	4.362381	-3.145825	0.311969
C	3.558596	-4.269836	0.053842
C	4.141624	-5.509313	-0.208579
C	5.533140	-5.641135	-0.213145
C	6.339785	-4.531561	0.047690
C	5.758566	-3.289583	0.310744
H	3.126526	-2.193519	-2.123125
H	1.247617	-1.622781	-3.628818
H	-0.735844	-0.441817	-2.767004
H	1.025243	-0.387630	1.168960
H	-2.161607	-3.595090	0.204916
H	-2.468223	3.583006	0.001530
H	-2.660097	-3.404423	-2.179111
H	-5.217256	-0.163395	-0.936152
H	-4.073167	-3.748685	-4.179071
H	-6.631030	-0.520102	-2.932949
H	-6.063450	-2.309419	-4.564236
H	-2.537397	-0.643481	2.802891
H	-5.287702	-3.029059	0.493382
H	-3.802211	-1.185039	4.861527
H	-6.554977	-3.550844	2.548480
H	-5.819036	-2.633732	4.737431
H	-1.055338	3.971658	-2.745060
H	2.308995	2.080596	-0.825836
H	0.313501	4.329541	-4.777069
H	3.668890	2.446989	-2.845911
H	2.680663	3.570139	-4.836107
H	-0.325456	1.458796	2.448371
H	1.533697	4.738463	0.357456
H	0.978065	2.020141	4.479503
H	2.817664	5.302915	2.390319
H	2.551689	3.944249	4.453983
H	4.822022	0.305039	2.551480
H	6.460827	2.151940	2.229229
H	7.346944	2.637514	-0.041652
H	6.598693	1.276351	-1.982390
H	4.972345	-0.550531	-1.664430
H	6.394032	-2.433163	0.511759
H	7.421119	-4.630722	0.045285
H	5.986511	-6.606104	-0.419794
H	3.511572	-6.370156	-0.411241
H	2.476629	-4.175776	0.051455

Table S157. XYZ Coordinates of H_meta_III_P(O)Ph_2

86

scf done: -5571.403933

C	6.545221	-0.677882	2.493199
C	6.003011	-0.144766	3.665452

C	4.627298	0.079042	3.762589
C	3.794634	-0.225366	2.685988
C	4.332687	-0.762427	1.501316
C	5.717001	-0.986540	1.413533
P	3.173725	-1.176471	0.148883
C	4.202661	-1.370011	-1.347160
C	4.316567	-2.605416	-1.999057
C	5.076745	-2.712669	-3.166417
C	5.718965	-1.590198	-3.691316
C	5.599446	-0.352720	-3.050542
C	4.840910	-0.239226	-1.886385
Pd	1.420689	0.410098	-0.011874
P	-0.258045	2.040320	-0.095765
O	-1.669876	1.494372	0.475753
C	0.306050	-0.832086	-1.105245
C	-0.713388	-1.561275	-0.486238
C	-1.490957	-2.456956	-1.239001
C	-1.233771	-2.626600	-2.607180
C	-0.213773	-1.896958	-3.216711
C	0.557066	-0.995797	-2.471800
H	-1.823047	-3.333865	-3.181776
C	-0.548922	2.824049	-1.719209
C	-1.770383	3.446062	-2.025104
C	-1.949361	4.045029	-3.272863
C	-0.918109	4.034960	-4.216283
C	0.297840	3.417326	-3.915946
C	0.479741	2.804441	-2.674748
C	0.277174	3.383803	1.029857
C	0.012416	3.264567	2.405373
C	0.480483	4.231772	3.294964
C	1.225789	5.316331	2.824071
C	1.498993	5.435308	1.459068
C	1.028861	4.474247	0.562824
O	2.608463	-2.687753	0.488539
Br	-4.037907	3.415909	1.139944
P	-2.867166	-3.417903	-0.509345
H	-0.902168	-1.438630	0.574593
H	1.349188	-0.438500	-2.961432
H	-0.008587	-2.027946	-4.275567
H	3.303623	-3.299924	0.779808
H	-2.391728	2.174781	0.681974
H	-2.574684	3.468907	-1.293860
H	1.419976	2.307515	-2.450248
H	-2.896181	4.522172	-3.507803
H	1.099191	3.401787	-4.648450
H	-1.063889	4.503435	-5.185102
H	-0.573389	2.428642	2.774588
H	1.235201	4.582903	-0.497457
H	0.260641	4.139554	4.354259
H	2.071445	6.280482	1.088981

H	1.589625	6.067725	3.518334
H	3.811499	-3.479705	-1.601875
H	4.752546	0.727171	-1.396425
H	5.163520	-3.673840	-3.663795
H	6.095337	0.522965	-3.457936
H	6.308137	-1.676188	-4.599238
H	2.724883	-0.052792	2.770580
H	6.148205	-1.396673	0.506647
H	4.203697	0.493034	4.672350
H	7.613899	-0.853833	2.418454
H	6.651995	0.097019	4.501747
C	-2.417579	-3.700411	1.241326
C	-4.328374	-2.321264	-0.518984
O	-3.115580	-4.710142	-1.253213
C	-1.694444	-4.868043	1.536031
C	-1.297134	-5.138592	2.845719
C	-1.619607	-4.247350	3.873055
C	-2.346752	-3.088893	3.589243
C	-2.748103	-2.816191	2.279327
H	-1.456262	-5.565986	0.739285
H	-0.740740	-6.045016	3.065164
H	-1.311820	-4.459141	4.892777
H	-2.609323	-2.400267	4.386767
H	-3.325856	-1.920544	2.074017
C	-5.578074	-2.916157	-0.751390
C	-6.736693	-2.137817	-0.754521
C	-6.656086	-0.762487	-0.521418
C	-5.414755	-0.161580	-0.295080
C	-4.252363	-0.935983	-0.301410
H	-5.633393	-3.983856	-0.939233
H	-7.699986	-2.604444	-0.938704
H	-7.559293	-0.158993	-0.520794
H	-5.339607	0.906599	-0.108379
H	-3.294966	-0.448517	-0.142844

Table S158. XYZ Coordinates of H_meta_IV_P(O)Ph_2

111

scf done: -6451.940187

C	-4.105578	-2.868269	3.988636
C	-3.019087	-2.071246	4.358216
C	-2.665035	-0.967305	3.582172
C	-3.404336	-0.645431	2.432395
C	-4.492485	-1.449001	2.065766
C	-4.839543	-2.555851	2.842332
P	-2.898579	0.817769	1.450874
C	-4.412070	1.353749	0.562545
C	-5.315226	2.228666	1.186673
C	-6.455738	2.659004	0.507139
C	-6.705036	2.218056	-0.795310
C	-5.809165	1.347326	-1.420414

C	-4.661256	0.920208	-0.749394
Pd	-0.874489	0.762694	0.096048
P	-1.149642	-1.516727	-0.463162
O	-2.745245	-1.746498	-0.675565
C	0.925979	0.723993	-0.946396
C	0.994209	0.936437	-2.330187
C	2.220520	0.920030	-3.006301
C	3.402828	0.682962	-2.309501
C	3.362404	0.464837	-0.921979
C	2.126254	0.492430	-0.253922
H	4.350477	0.671079	-2.840525
P	-0.515362	3.110700	0.168870
O	-1.568552	3.810494	1.265786
C	-0.919582	3.942403	-1.407503
C	-0.453652	5.232892	-1.712407
C	-0.855883	5.861893	-2.890847
C	-1.723992	5.213079	-3.772563
C	-2.196357	3.932822	-3.474098
C	-1.797302	3.299415	-2.297073
C	1.091058	3.804454	0.707578
C	1.253292	4.195595	2.047028
C	2.473374	4.714243	2.484451
C	3.540752	4.841779	1.593336
C	3.388878	4.442066	0.262661
C	2.173390	3.920348	-0.180485
O	-2.760719	1.881367	2.703235
C	-0.566833	-2.706473	0.799566
C	-0.983666	-4.048821	0.764425
C	-0.526708	-4.943843	1.731524
C	0.350704	-4.514271	2.732100
C	0.765288	-3.182134	2.772459
C	0.302232	-2.278636	1.813292
C	-0.413315	-2.111744	-2.032625
C	-1.071992	-1.822210	-3.238354
C	-0.520187	-2.223447	-4.455131
C	0.695202	-2.912313	-4.480614
C	1.354514	-3.204022	-3.284394
C	0.804042	-2.807070	-2.064142
Br	-3.922663	-4.430066	-1.787396
H	2.248236	1.090849	-4.079183
H	0.088408	1.117226	-2.900896
H	2.110146	0.344648	0.821231
P	4.958147	0.280365	-0.049666
H	-3.047184	-2.640934	-1.037641
H	-2.326894	2.702613	2.385511
H	-1.671776	-4.391246	-0.005261
H	0.612848	-1.239376	1.856400
H	-0.855481	-5.978521	1.703788
H	1.442670	-2.841494	3.548972
H	0.705759	-5.216454	3.480708

H	-2.022376	-1.298154	-3.225326
H	1.317619	-3.050581	-1.139617
H	-1.041676	-2.002300	-5.381643
H	2.294585	-3.747384	-3.299062
H	1.123870	-3.224746	-5.428177
H	-5.119232	2.574446	2.196468
H	-3.970998	0.236403	-1.232248
H	-7.149038	3.338036	0.994698
H	-6.000372	1.002657	-2.432383
H	-7.593397	2.554076	-1.321914
H	0.223622	5.746415	-1.038152
H	-2.167742	2.304978	-2.066569
H	-0.489551	6.857795	-3.119879
H	-2.872506	3.426835	-4.156211
H	-2.031826	5.704788	-4.690383
H	0.427540	4.108832	2.745321
H	2.070568	3.612185	-1.215526
H	2.584690	5.022708	3.519395
H	4.218074	4.531522	-0.431728
H	4.487225	5.251470	1.932877
H	-5.070985	-1.212975	1.179695
H	-1.823037	-0.350172	3.879062
H	-5.686573	-3.170299	2.552363
H	-2.446124	-2.308081	5.249763
H	-4.379519	-3.728322	4.592500
H	-1.549942	4.781263	1.293284
C	4.583525	-0.081552	1.704614
C	5.740474	-1.219452	-0.740441
O	5.852261	1.494183	-0.187001
C	7.142344	-1.257701	-0.793628
C	7.797267	-2.385794	-1.289603
C	7.057622	-3.484527	-1.734733
C	5.661478	-3.451777	-1.690036
C	5.003585	-2.322503	-1.198621
H	7.712064	-0.396956	-0.457625
H	8.882233	-2.406219	-1.331316
H	7.567537	-4.362267	-2.120787
H	5.084887	-4.301953	-2.042200
H	3.918298	-2.297278	-1.185282
C	4.448332	1.011243	2.577463
C	4.185034	0.803720	3.932004
C	4.066067	-0.496685	4.430319
C	4.208464	-1.588373	3.570170
C	4.462190	-1.383599	2.211922
H	4.558747	2.021432	2.195961
H	4.082050	1.655032	4.598307
H	3.871449	-0.658797	5.486388
H	4.131306	-2.600275	3.956611
H	4.576903	-2.239703	1.555023

Table S159. XYZ Coordinates of H_meta_V_P(O)Ph_2

110

scf done: -6451.488185

C	7.005014	0.425213	-0.758569
C	6.282498	1.071234	-1.764984
C	4.994741	1.543823	-1.508606
C	4.414362	1.379170	-0.240337
C	5.146812	0.731966	0.764525
C	6.434632	0.258322	0.505278
P	2.705685	2.021263	0.014392
C	2.647910	2.448994	1.802666
C	2.902371	3.766724	2.210884
C	2.838732	4.108732	3.563231
C	2.521262	3.139415	4.518206
C	2.261625	1.826286	4.116974
C	2.316275	1.480674	2.764657
Pd	0.738785	0.752557	-0.682964
P	1.825734	-1.351335	-0.409189
O	2.691329	-1.329782	0.972042
C	-0.980737	-0.249269	-1.269077
C	-1.898511	-0.754061	-0.331034
C	-3.054865	-1.451221	-0.726910
C	-3.296889	-1.670938	-2.093675
C	-2.392852	-1.187592	-3.036642
C	-1.255738	-0.482065	-2.626553
H	-4.182976	-2.209525	-2.417584
P	-0.363720	2.820767	-1.106217
O	0.607116	3.867895	-1.756237
C	-1.006446	3.559998	0.458057
C	-0.880064	4.944154	0.649758
C	-1.338784	5.539232	1.826447
C	-1.925249	4.756776	2.824997
C	-2.047301	3.376627	2.645209
C	-1.584777	2.779539	1.469994
C	-1.841460	2.824072	-2.211226
C	-1.631778	3.011410	-3.585996
C	-2.709684	3.026042	-4.473446
C	-4.011747	2.856905	-3.996980
C	-4.229789	2.677155	-2.628476
C	-3.151977	2.657956	-1.740387
O	2.818490	3.440658	-0.753930
C	2.980523	-1.688901	-1.798348
C	4.073900	-2.558320	-1.653218
C	4.914805	-2.806801	-2.738865
C	4.668862	-2.204228	-3.976452
C	3.583627	-1.338964	-4.126295
C	2.748273	-1.074759	-3.038329
C	0.875351	-2.921630	-0.285114
C	0.529366	-3.422264	0.979158
C	-0.192140	-4.612395	1.092534

C	-0.584160	-5.307683	-0.053567
C	-0.251165	-4.809438	-1.315623
C	0.476054	-3.624282	-1.432387
Br	4.394700	-3.757330	2.069081
P	-4.194127	-2.212563	0.477500
H	-1.713652	-0.614546	0.730300
H	-0.582525	-0.100863	-3.390274
H	-2.575435	-1.352488	-4.095690
H	3.198838	-2.158230	1.232967
H	1.896176	3.674503	-1.219006
H	4.275603	-3.034036	-0.696040
H	1.917554	-0.383249	-3.148501
H	5.762927	-3.474843	-2.619152
H	3.392077	-0.862008	-5.083085
H	5.324471	-2.405395	-4.818736
H	0.846319	-2.897312	1.873951
H	0.741984	-3.256455	-2.418302
H	-0.439997	-4.999148	2.076781
H	-0.549277	-5.347835	-2.210517
H	-1.144312	-6.233677	0.035958
H	3.141607	4.518172	1.465340
H	2.115071	0.459050	2.456555
H	3.036173	5.131852	3.870542
H	2.012116	1.070302	4.856124
H	2.472183	3.406903	5.569807
H	-0.411712	5.544171	-0.124526
H	-1.672797	1.704546	1.342484
H	-1.235687	6.611800	1.965697
H	-2.494731	2.761497	3.419919
H	-2.279486	5.219289	3.741780
H	-0.622497	3.162677	-3.955781
H	-3.338277	2.527742	-0.679056
H	-2.532550	3.175760	-5.534833
H	-5.241720	2.563502	-2.249786
H	-4.851764	2.872852	-4.685368
H	4.718038	0.597475	1.750956
H	4.443905	2.055794	-2.291087
H	6.991717	-0.239275	1.293730
H	6.720987	1.209768	-2.749077
H	8.007454	0.057221	-0.957253
C	-3.868884	-1.444582	2.108287
C	-5.877951	-1.688322	-0.008023
O	-4.086393	-3.721114	0.558337
C	-3.067838	-2.159369	3.012718
C	-2.798269	-1.640209	4.280420
C	-3.337054	-0.408455	4.660089
C	-4.143975	0.304322	3.768506
C	-4.406073	-0.207817	2.496242
H	-2.669389	-3.126035	2.722041
H	-2.177647	-2.200894	4.973130

H	-3.137120	-0.008925	5.649967
H	-4.578536	1.253852	4.066742
H	-5.043260	0.350267	1.817416
C	-6.932944	-2.573319	0.263271
C	-8.246477	-2.222214	-0.050954
C	-8.517419	-0.983958	-0.638935
C	-7.472107	-0.100321	-0.919025
C	-6.156424	-0.450573	-0.608455
H	-6.714988	-3.539169	0.708320
H	-9.056248	-2.914714	0.159209
H	-9.539552	-0.710764	-0.884284
H	-7.679803	0.858199	-1.385415
H	-5.348565	0.233137	-0.850528

Table S160. XYZ Coordinates of H_meta_TS2_P(O)Ph_2

110

scf done: -6451.449226

C	0.017674	-3.170208	-0.196810
C	-1.331087	-3.036879	-0.559582
C	-2.125848	-4.191226	-0.672063
C	-1.569897	-5.448138	-0.430106
C	-0.219505	-5.571221	-0.084053
C	0.577210	-4.430940	0.031381
P	-1.989825	-1.340533	-0.854018
O	-3.505663	-1.330734	-0.204885
Pd	-0.710529	0.540792	-0.013794
P	0.639140	2.513382	-0.429965
C	1.883873	3.242817	-1.603952
C	3.213614	3.331148	-1.161272
C	4.201745	3.868156	-1.987157
C	3.875816	4.320846	-3.268985
C	2.555482	4.240996	-3.714709
C	1.563705	3.707132	-2.886988
P	-1.466496	1.171216	2.150366
C	-3.111149	1.971371	2.399703
C	-3.248319	3.076794	3.252438
C	-4.497067	3.674769	3.434867
C	-5.619577	3.173401	2.770547
C	-5.487924	2.074881	1.916903
C	-4.239359	1.478403	1.724613
C	-1.507243	-0.184959	3.401984
C	-0.736476	-0.105658	4.570896
C	-0.762470	-1.145083	5.504808
C	-1.557282	-2.271425	5.281555
C	-2.325999	-2.357117	4.116914
C	-2.299447	-1.323730	3.178852
O	-0.442703	2.237762	2.860994
C	1.175138	0.534164	-1.096759
C	2.156087	0.006502	-0.234518
C	3.129841	-0.887405	-0.707233

C	3.146420	-1.228548	-2.069008
C	2.204531	-0.676147	-2.940547
C	1.232614	0.202849	-2.464399
H	3.897478	-1.911740	-2.454274
C	-2.316794	-1.340114	-2.670499
C	-3.362132	-0.549410	-3.178539
C	-3.568804	-0.443920	-4.554822
C	-2.728278	-1.117646	-5.445543
C	-1.683221	-1.901316	-4.950854
C	-1.477473	-2.012149	-3.573562
O	1.152413	2.955065	0.959852
C	-0.900199	3.402645	-0.939955
C	-1.265961	4.530866	-0.188380
C	-2.420474	5.246862	-0.510092
C	-3.222917	4.844076	-1.580702
C	-2.867414	3.719558	-2.329960
C	-1.714455	2.997974	-2.012417
Br	-5.894702	-3.235234	-1.172192
H	2.223635	-0.939803	-3.994119
H	0.508880	0.614531	-3.160172
H	2.155250	0.299585	0.810292
P	4.269868	-1.747997	0.433250
H	-4.174697	-1.969674	-0.583672
H	0.222561	2.546386	2.160637
H	-3.177603	-4.110601	-0.938369
H	0.632883	-2.282672	-0.089825
H	-2.191290	-6.335568	-0.514153
H	1.627104	-4.504429	0.301225
H	0.205852	-6.554210	0.098404
H	-4.026347	-0.031473	-2.493493
H	-0.667311	-2.632757	-3.203294
H	-4.389279	0.160713	-4.931000
H	-1.030305	-2.434295	-5.636358
H	-2.889565	-1.035440	-6.516451
H	-2.374163	3.467027	3.763619
H	-4.142787	0.631979	1.049071
H	-4.593126	4.531835	4.095873
H	-6.356782	1.685765	1.393185
H	-6.590291	3.639796	2.912897
H	-0.647028	4.840066	0.648021
H	-1.460984	2.121601	-2.600686
H	-2.691936	6.120126	0.076066
H	-3.487262	3.399625	-3.162383
H	-4.120393	5.402486	-1.829540
H	3.467340	2.990130	-0.162948
H	0.540924	3.668777	-3.247034
H	5.224857	3.937248	-1.628384
H	2.291298	4.600359	-4.705207
H	4.644843	4.738225	-3.912457
H	-2.890490	-1.408970	2.270883

H	-0.120040	0.770094	4.742598
H	-2.943883	-3.232177	3.935663
H	-0.162514	-1.072095	6.407864
H	-1.576056	-3.078962	6.007809
O	3.901722	-3.201001	0.652231
C	5.939146	-1.560671	-0.283184
C	4.248226	-0.808998	2.001905
C	3.569113	-1.379937	3.088486
C	3.498000	-0.703224	4.308087
C	4.107960	0.545136	4.450705
C	4.793720	1.115710	3.374021
C	4.865452	0.442625	2.153956
H	3.106914	-2.354978	2.972866
H	2.968966	-1.151430	5.143677
H	4.055034	1.070838	5.399524
H	5.275848	2.082218	3.485660
H	5.409673	0.891836	1.328948
C	6.858995	-2.597686	-0.063750
C	8.160971	-2.498782	-0.555940
C	8.554113	-1.364105	-1.270278
C	7.642121	-0.330415	-1.497903
C	6.337669	-0.427079	-1.009681
H	6.544803	-3.482473	0.481110
H	8.865823	-3.307057	-0.385455
H	9.567132	-1.287963	-1.654230
H	7.942824	0.547774	-2.061283
H	5.631891	0.373447	-1.210409

Table S161. XYZ Coordinates of H_meta_VI_P(O)Ph$_2$

110

scf done: -6451.483400

C	-6.521309	-1.543878	0.938443
C	-6.137845	-2.889342	0.934565
C	-4.787077	-3.228051	0.844116
C	-3.820580	-2.222174	0.756298
C	-4.195443	-0.870308	0.773393
C	-5.558645	-0.537190	0.863476
P	-2.871273	0.403782	0.669660
C	-3.073546	1.337171	2.243626
C	-2.784004	2.711224	2.278263
C	-2.815827	3.411370	3.485620
C	-3.124567	2.747594	4.675679
C	-3.406580	1.379082	4.651841
C	-3.381308	0.677692	3.444769
Pd	-0.712723	-0.313349	0.030792
P	-0.766216	0.078297	-2.316101
O	-1.880912	1.242997	-2.685801
P	1.720930	-2.628530	0.253135
C	3.254711	-3.108161	1.137425
C	4.157266	-3.953661	0.473404

C	5.330643	-4.368409	1.104843
C	5.613186	-3.942927	2.405490
C	4.723163	-3.096940	3.071550
C	3.550102	-2.677283	2.440560
C	1.270190	-0.987244	0.922244
C	0.278848	-0.818881	1.958434
C	0.388665	0.301924	2.848046
C	1.364106	1.250428	2.683632
C	2.319760	1.129973	1.620626
C	2.276330	0.034758	0.782745
H	1.444213	2.080551	3.378154
O	1.937892	-2.670634	-1.247733
C	0.449613	-3.833151	0.790183
C	0.377202	-4.327849	2.102617
C	-0.595363	-5.267450	2.448475
C	-1.495488	-5.731992	1.485287
C	-1.421210	-5.254312	0.174674
C	-0.454162	-4.307794	-0.172366
O	-3.404100	1.499142	-0.461414
C	-1.309398	-1.306491	-3.418334
C	-0.526383	-2.467373	-3.542888
C	-0.954838	-3.518653	-4.355983
C	-2.169949	-3.432282	-5.042622
C	-2.955548	-2.285442	-4.913955
C	-2.528241	-1.226628	-4.107213
C	0.682794	0.745078	-3.250710
C	0.560657	1.891739	-4.051578
C	1.662074	2.378610	-4.759959
C	2.893957	1.724592	-4.681298
C	3.021426	0.584105	-3.883507
C	1.927037	0.098660	-3.163064
Br	-6.077214	3.176299	-0.214674
H	-2.244646	-6.471045	1.753706
H	-0.642305	-5.644451	3.465732
H	1.085702	-3.995348	2.854872
H	-0.390230	-3.943647	-1.191881
H	-2.110746	-5.621466	-0.579754
H	-4.271953	1.983200	-0.304310
H	-2.471386	1.406964	-1.913110
H	3.941140	-4.272481	-0.541437
H	2.874176	-2.007793	2.964052
H	6.024585	-5.019641	0.581521
H	4.943836	-2.757666	4.079268
H	6.526778	-4.265027	2.896563
H	-0.296910	-1.674525	2.297060
H	3.033011	-0.090159	0.013536
H	-0.315810	0.380731	3.670645
P	3.582973	2.435538	1.503753
H	-0.396833	2.396532	-4.118388
H	2.042371	-0.782335	-2.535628

H	1.554980	3.266745	-5.376713
H	3.977492	0.072501	-3.815311
H	3.748702	2.102153	-5.235295
H	-2.551870	3.237078	1.357345
H	-3.612515	-0.383591	3.439369
H	-2.601473	4.476253	3.495301
H	-3.654430	0.857481	5.571937
H	-3.147871	3.293047	5.614524
H	-5.869384	0.504904	0.857998
H	-2.770032	-2.486320	0.668096
H	-7.573059	-1.279266	1.001624
H	-4.482033	-4.270230	0.835091
H	-6.891624	-3.669008	0.997362
H	0.408731	-2.556230	-2.995575
H	-3.133775	-0.330993	-4.016387
H	-0.340020	-4.409804	-4.449817
H	-3.901155	-2.209891	-5.443894
H	-2.501717	-4.254174	-5.670665
C	4.986964	1.754539	0.548065
C	2.856831	3.779243	0.497786
O	4.022120	2.931539	2.864367
C	3.209699	5.098029	0.822278
C	2.693018	6.166146	0.087304
C	1.820557	5.925380	-0.977234
C	1.460527	4.615113	-1.302848
C	1.971498	3.544252	-0.565987
H	3.877288	5.279093	1.658863
H	2.968135	7.184198	0.346989
H	1.418236	6.756669	-1.548825
H	0.782849	4.422538	-2.128880
H	1.673030	2.531626	-0.820889
C	6.050866	1.206213	1.282833
C	7.159537	0.671237	0.626490
C	7.217558	0.681665	-0.769705
C	6.166618	1.231764	-1.507097
C	5.055645	1.769329	-0.853071
H	6.010394	1.214711	2.367526
H	7.978311	0.251327	1.203253
H	8.081738	0.267946	-1.281133
H	6.212955	1.250353	-2.591836
H	4.252190	2.203144	-1.439501

Table S162. XYZ Coordinates of H_meta_VII_P(O)Ph_2

110

scf done: -6451.480096

C	-0.448283	-0.382840	-2.209630
C	-1.120041	-1.610142	-2.080621
C	-1.860797	-2.108866	-3.162846
C	-1.935860	-1.384079	-4.354419
C	-1.268826	-0.163215	-4.474972

C	-0.524098	0.336155	-3.402636
P	-0.981669	-2.488821	-0.485598
O	0.373215	-2.304639	0.164761
C	-2.329926	-1.843914	0.574161
C	-3.502803	-1.284192	0.055794
C	-4.501109	-0.791064	0.912572
C	-4.311112	-0.867319	2.299108
C	-3.138248	-1.417645	2.821770
C	-2.150364	-1.901384	1.966150
H	-5.072163	-0.490727	2.974232
C	-1.357775	-4.246564	-0.807965
C	-0.274830	-5.096499	-1.086178
C	-0.494334	-6.448247	-1.351797
C	-1.793974	-6.961808	-1.340831
C	-2.874829	-6.122971	-1.059333
C	-2.659779	-4.769701	-0.790404
C	2.267210	-3.482805	3.856502
C	3.194609	-3.702323	4.880564
C	4.534281	-3.361750	4.686857
C	4.949327	-2.803409	3.474048
C	4.023860	-2.582880	2.444678
C	2.673701	-2.922540	2.644995
P	4.520420	-1.787912	0.861579
C	4.286337	-3.142981	-0.358707
C	3.892876	-2.806350	-1.662946
C	3.733937	-3.797536	-2.634346
C	3.969211	-5.135385	-2.309321
C	4.363982	-5.480649	-1.012575
C	4.519307	-4.491242	-0.041384
Pd	3.655700	0.286048	0.418173
P	3.016202	2.492046	0.100373
C	3.209157	3.138803	-1.615225
C	3.631325	4.448283	-1.890850
C	3.770819	4.878646	-3.213743
C	3.491020	4.011320	-4.271721
C	3.074799	2.703413	-4.005536
C	2.943722	2.268447	-2.685882
C	1.249753	2.867567	0.489042
C	0.551430	3.959322	-0.052690
C	-0.770168	4.208653	0.320688
C	-1.412425	3.369482	1.236733
C	-0.731591	2.274848	1.774915
C	0.590895	2.026163	1.401158
O	3.887425	3.522884	1.039958
O	6.189512	-1.788940	1.015170
Br	3.286036	6.638231	1.406255
H	-1.325731	0.396818	-5.403688
H	-2.509988	-1.776884	-5.188112
H	-2.373631	-3.062078	-3.082691
H	0.137382	0.002030	-1.380200

H	0.001247	1.281893	-3.495887
H	3.589882	4.476085	1.085171
H	6.497274	-0.872820	0.962499
H	0.735430	-4.699391	-1.084675
H	-3.506142	-4.130021	-0.560421
H	0.348189	-7.099510	-1.564130
H	-3.884766	-6.521292	-1.042806
H	-1.963627	-8.014611	-1.546269
H	-3.636996	-1.215163	-1.019530
H	-1.231544	-2.312401	2.372222
P	-6.060238	-0.174884	0.176263
H	-2.995071	-1.463998	3.896757
H	5.991185	-2.542197	3.320970
H	1.941625	-2.753560	1.857916
H	5.259864	-3.530648	5.477635
H	1.223650	-3.747778	4.002903
H	2.872887	-4.135033	5.823529
H	1.122487	1.171157	1.812588
H	1.038382	4.617445	-0.764961
H	-1.229090	1.613330	2.478122
H	-1.297894	5.060006	-0.099752
H	-2.439430	3.569671	1.528755
H	3.840116	5.137784	-1.077242
H	2.643187	1.243257	-2.481953
H	4.098251	5.894684	-3.415749
H	2.865055	2.019907	-4.823370
H	3.602796	4.349154	-5.297953
H	3.707393	-1.763799	-1.908910
H	4.816453	-4.768771	0.965555
H	3.425086	-3.526015	-3.639585
H	4.547219	-6.520680	-0.758480
H	3.844759	-5.908049	-3.062527
C	-6.936383	0.741896	1.492740
C	-5.560638	1.051458	-1.081329
O	-6.923439	-1.273310	-0.403185
C	-6.397196	1.208822	-2.197467
C	-6.093418	2.155787	-3.176200
C	-4.952356	2.952291	-3.048901
C	-4.111563	2.797061	-1.944137
C	-4.411691	1.849705	-0.963580
H	-7.275770	0.579391	-2.298991
H	-6.743860	2.268879	-4.038363
H	-4.715642	3.687885	-3.811961
H	-3.218000	3.406068	-1.845700
H	-3.737750	1.727006	-0.121351
C	-8.131055	0.191286	1.979077
C	-8.850607	0.846444	2.980639
C	-8.381918	2.054459	3.500931
C	-7.193854	2.610882	3.017239
C	-6.473572	1.960012	2.015618

H	-8.491410	-0.743816	1.562569
H	-9.775455	0.414851	3.351612
H	-8.941754	2.564805	4.279118
H	-6.830729	3.552884	3.416967
H	-5.558058	2.408121	1.641556

Table S163. XYZ Coordinates of H_meta_I_Me

66

scf done: -4731.393090

C	2.439892	-2.670728	-1.902149
C	3.227381	-1.682092	-1.290953
C	4.626357	-1.786805	-1.353790
C	5.222923	-2.856713	-2.021560
C	4.430305	-3.835435	-2.629028
C	3.038285	-3.742069	-2.568994
P	2.376246	-0.272570	-0.475136
Pd	0.157987	-0.560123	0.068363
P	-2.064349	-0.841988	0.602849
O	-2.655241	0.070028	1.872765
O	2.730564	0.922119	-1.590617
C	3.502229	0.153516	0.917561
C	4.483191	1.148824	0.800289
C	5.312849	1.450574	1.883730
C	5.171975	0.760833	3.089891
C	4.192524	-0.229169	3.215518
C	3.356977	-0.525810	2.138240
C	-2.595116	-2.501541	1.183676
C	-3.946827	-2.875720	1.256966
C	-4.302978	-4.134362	1.741409
C	-3.314322	-5.034439	2.151618
C	-1.967663	-4.672975	2.078538
C	-1.609736	-3.411773	1.596388
C	-3.264747	-0.456379	-0.740744
C	-2.991923	-0.901285	-2.044642
C	-3.875764	-0.618855	-3.087187
C	-5.033601	0.126362	-2.841331
C	-5.303993	0.584855	-1.550329
C	-4.425280	0.294410	-0.502681
C	1.411474	4.092039	-1.041446
C	0.750162	3.702651	-2.210425
C	-0.571089	3.231021	-2.170243
C	-1.215011	3.150627	-0.925787
C	-0.538377	3.540546	0.226898
C	0.770226	4.015314	0.197698
C	-1.301559	2.842628	-3.433484
Br	-1.438935	3.420209	1.923545
H	1.260906	3.776484	-3.166485
H	2.055207	1.619617	-1.557628
H	-2.205120	0.932280	1.897469
H	-2.233432	2.782243	-0.863625

H	-4.720910	-2.187524	0.931151
H	-0.562584	-3.125206	1.534573
H	-5.350743	-4.415351	1.795157
H	-1.197103	-5.370515	2.393518
H	-3.594681	-6.015640	2.523568
H	-4.633495	0.652813	0.500380
H	-2.081578	-1.461877	-2.244363
H	-6.199103	1.168887	-1.356220
H	-3.657534	-0.971585	-4.091031
H	-5.716972	0.353464	-3.654302
H	5.250445	-1.036851	-0.877423
H	1.356421	-2.595096	-1.848342
H	6.305747	-2.929398	-2.064920
H	2.419247	-4.502192	-3.036524
H	4.897769	-4.669826	-3.143771
H	4.589137	1.687025	-0.136176
H	2.582795	-1.282193	2.246115
H	6.068542	2.224579	1.784237
H	4.073291	-0.762053	4.154433
H	5.816839	0.997722	3.931088
H	1.275851	4.314552	1.108121
H	2.430893	4.462448	-1.092002
H	-1.926174	1.958077	-3.278430
H	-1.960721	3.653525	-3.765918
H	-0.601258	2.632854	-4.246155

Table S164. XYZ Coordinates of H_meta_TS1_Me

66

scf done: -4731.361863

C	-2.389479	3.165097	-1.473979
C	-2.453596	2.115306	-0.547053
C	-2.690839	2.413604	0.806075
C	-2.873026	3.733248	1.217931
C	-2.813145	4.774518	0.285789
C	-2.570881	4.487730	-1.058374
P	-2.128845	0.371245	-1.041896
C	-3.743141	-0.444192	-0.674042
C	-3.740463	-1.813548	-0.361648
C	-4.939925	-2.490483	-0.130469
C	-6.154673	-1.805813	-0.207479
C	-6.168889	-0.442844	-0.517667
C	-4.971507	0.235294	-0.748947
Pd	-0.113138	-0.614857	-0.211000
Br	0.042366	-3.337338	-0.417670
C	0.719494	-1.989046	1.201956
C	2.079293	-2.098812	1.552883
C	2.431857	-1.869190	2.875776
C	1.458839	-1.579825	3.849846
C	0.105036	-1.539970	3.517604
C	-0.270747	-1.802564	2.180786

H	1.763345	-1.417360	4.879867
P	1.721007	0.749586	-0.841086
O	1.256094	1.733838	-2.111725
C	3.291612	0.005286	-1.464297
C	3.249662	-1.316928	-1.936227
C	4.388357	-1.912995	-2.483187
C	5.581843	-1.192809	-2.565940
C	5.635053	0.124470	-2.100951
C	4.498704	0.720252	-1.552183
C	2.316170	1.985090	0.390897
C	1.819975	3.298754	0.365406
C	2.190898	4.216471	1.351239
C	3.053439	3.832771	2.379897
C	3.546744	2.525274	2.417934
C	3.181427	1.607196	1.432923
O	-2.129758	0.446930	-2.710138
H	3.480458	-1.908952	3.158891
H	2.829625	-2.312228	0.800547
H	-1.321309	-1.898474	1.925553
C	-0.958509	-1.275624	4.557312
H	-2.739404	1.613506	1.540791
H	-2.194584	2.951307	-2.520077
H	-3.061705	3.949621	2.265447
H	-2.522460	5.291336	-1.787596
H	-2.952670	5.802229	0.607804
H	3.575642	0.596682	1.474386
H	1.146047	3.605456	-0.428384
H	4.221146	2.220477	3.213052
H	1.804787	5.231067	1.313046
H	3.340597	4.546414	3.146494
H	-2.795325	-2.346497	-0.299335
H	-4.924762	-3.549008	0.112163
H	-7.087693	-2.330476	-0.023602
H	-7.111964	0.092779	-0.575582
H	-4.994705	1.296214	-0.979565
H	-2.981115	0.737335	-3.075516
H	4.556658	1.741179	-1.186763
H	6.562080	0.687004	-2.163485
H	6.468815	-1.655732	-2.988559
H	4.343384	-2.937721	-2.840294
H	2.323537	-1.881389	-1.864231
H	1.966245	2.325275	-2.409921
H	-1.735479	-2.047801	4.535719
H	-0.532182	-1.249771	5.563490
H	-1.456079	-0.315120	4.378872

Table S165. XYZ Coordinates of H_meta_IIa_Me

66

scf done: -4731.435935

C	3.230130	1.368669	-0.807507

C	2.400814	0.582615	-1.622400
C	2.128680	1.007133	-2.934667
C	2.686661	2.189107	-3.423120
C	3.516691	2.963019	-2.607958
C	3.786133	2.550470	-1.301550
P	1.587270	-0.935560	-0.999022
C	2.836344	-1.743381	0.070867
C	4.195219	-1.794066	-0.284959
C	5.105705	-2.482444	0.515901
C	4.670555	-3.128072	1.677800
C	3.321794	-3.089085	2.035939
C	2.407819	-2.401884	1.233923
Pd	-0.685606	-0.915395	-0.108551
C	-2.648626	-1.057379	0.501348
C	-2.986405	-1.731660	1.687031
C	-4.332449	-1.888004	2.053309
C	-5.346538	-1.375357	1.247864
C	-5.035757	-0.694286	0.059764
C	-3.686043	-0.540718	-0.291605
H	-6.386492	-1.500312	1.539867
P	-0.743048	1.062576	1.055089
O	-1.642584	0.990764	2.433665
C	-1.489502	2.423095	0.088572
C	-1.188889	2.563082	-1.275592
C	-1.752796	3.605227	-2.012704
C	-2.628159	4.504492	-1.397771
C	-2.937259	4.363214	-0.042702
C	-2.369869	3.327538	0.701872
C	0.793159	1.744926	1.777607
C	1.559315	0.913144	2.611978
C	2.710829	1.402371	3.227048
C	3.110184	2.725449	3.012469
C	2.353406	3.556222	2.184610
C	1.198352	3.070270	1.567410
Br	-1.056186	-3.157782	-1.316401
O	1.602026	-1.819140	-2.394751
H	4.542174	-1.293038	-1.183370
H	-2.319212	0.290998	2.344819
H	0.826670	-2.424897	-2.364131
H	-4.581987	-2.416757	2.969983
H	-2.211296	-2.159024	2.319314
H	-3.448195	-0.004964	-1.208019
C	-6.135278	-0.136048	-0.814716
H	1.356462	-2.379172	1.507357
H	6.154144	-2.515308	0.234997
H	2.980306	-3.592740	2.935268
H	5.382681	-3.661530	2.300459
H	1.498584	0.401180	-3.577032
H	3.453391	1.059757	0.208221
H	2.476969	2.501325	-4.441935

H	4.430995	3.146101	-0.662675
H	3.952512	3.881429	-2.989966
H	-2.612172	3.214363	1.753329
H	-0.516791	1.862386	-1.762185
H	-3.620644	5.058177	0.435951
H	-1.514352	3.709838	-3.066803
H	-3.071703	5.310529	-1.974612
H	1.256552	-0.115164	2.783272
H	0.616308	3.725702	0.929106
H	3.295467	0.752457	3.870928
H	2.658474	4.584796	2.017883
H	4.008130	3.105677	3.490229
H	-5.728484	0.367595	-1.695902
H	-6.807421	-0.929861	-1.160674
H	-6.750861	0.586831	-0.266969

Table S166. XYZ Coordinates of H_meta_IIb_Me

66

scf done: -4731.437304

C	3.471992	1.641941	-1.348775
C	3.146160	1.637564	0.019206
C	3.441823	2.763534	0.801562
C	4.069821	3.872088	0.227901
C	4.401845	3.866834	-1.127702
C	4.101237	2.750408	-1.914392
P	2.267938	0.200500	0.742679
Pd	0.146946	-0.155214	-0.221232
Br	1.181162	-1.334312	-2.318435
C	3.455564	-1.191711	0.664860
C	2.956414	-2.496795	0.812877
C	3.828391	-3.584161	0.849534
C	5.205960	-3.379248	0.730294
C	5.709790	-2.085483	0.579539
C	4.840365	-0.993225	0.548932
O	2.202149	0.543921	2.360037
P	-2.021885	-0.428936	-1.118121
C	-3.022826	-1.729932	-0.306133
C	-2.358075	-2.744197	0.402334
C	-3.076550	-3.798613	0.968138
C	-4.465689	-3.848559	0.831295
C	-5.134987	-2.845553	0.124756
C	-4.419282	-1.790838	-0.442894
C	-0.690288	0.856073	1.331792
C	-0.663496	2.258891	1.327020
C	-1.235180	2.975925	2.382662
C	-1.840243	2.306808	3.448470
C	-1.876692	0.906331	3.476414
C	-1.298907	0.197345	2.408033
H	-2.287163	2.873179	4.262098
O	-1.944013	-0.903137	-2.692605

C	-3.123465	1.024458	-1.244936
C	-3.255837	1.690551	-2.473098
C	-4.055447	2.831466	-2.570913
C	-4.725930	3.317323	-1.446744
C	-4.594303	2.660178	-0.220231
C	-3.794509	1.522130	-0.115309
H	-1.211326	4.063132	2.369613
H	-0.199173	2.798830	0.505958
H	-1.340482	-0.888802	2.428280
C	-2.499938	0.166869	4.639073
H	3.078896	0.523905	2.776470
H	-1.005114	-1.131040	-2.888906
H	3.180269	2.777171	1.854311
H	3.229033	0.783041	-1.967590
H	4.297396	4.737814	0.842667
H	4.354713	2.743560	-2.970315
H	4.889617	4.729419	-1.571810
H	1.884937	-2.657759	0.887766
H	5.241424	0.008236	0.431374
H	3.433706	-4.589390	0.962245
H	6.779406	-1.924282	0.484972
H	5.884489	-4.226846	0.751794
H	-2.745300	1.306803	-3.349477
H	-3.694408	1.024954	0.844063
H	-4.156190	3.336058	-3.527279
H	-5.113517	3.033882	0.657241
H	-5.348869	4.203486	-1.524789
H	-1.277324	-2.706112	0.509301
H	-4.948508	-1.015665	-0.987459
H	-2.553231	-4.576290	1.516243
H	-6.214688	-2.883845	0.015595
H	-5.026072	-4.666524	1.274277
H	-2.944078	-0.780847	4.319124
H	-1.751170	-0.069669	5.405666
H	-3.279611	0.764191	5.121232

Table S167. XYZ Coordinates of H_meta_III_Me

66

scf done: -4731.407623

C	-4.733305	2.269761	-0.323802
C	-3.471504	1.861748	-0.788324
C	-2.633778	2.799070	-1.420844
C	-3.052825	4.119231	-1.585890
C	-4.307809	4.516618	-1.117828
C	-5.146194	3.592101	-0.489351
P	-2.874692	0.138412	-0.650635
O	-3.305456	-0.601506	-2.060161
Pd	-0.536727	0.041347	-0.254526
C	-0.424947	-1.855266	-0.877254
C	0.084849	-2.135227	-2.147718

C	0.130817	-3.456676	-2.625420
C	-0.343352	-4.485680	-1.800451
C	-0.845670	-4.203598	-0.529511
C	-0.891596	-2.886783	-0.056192
H	-0.318143	-5.512565	-2.156035
P	1.759364	0.118885	0.170193
C	2.407112	-0.932148	1.515877
C	3.767234	-1.275611	1.584854
C	4.229936	-2.066627	2.637153
C	3.348358	-2.513368	3.625690
C	1.995248	-2.174242	3.560575
C	1.522569	-1.393737	2.503613
C	2.068143	1.851759	0.686857
C	1.977706	2.239683	2.033888
C	2.119133	3.582406	2.387450
C	2.349273	4.547437	1.403560
C	2.438772	4.167923	0.061492
C	2.293405	2.828007	-0.299058
O	2.650301	-0.132827	-1.154494
C	-3.909704	-0.643390	0.636165
C	-4.719877	-1.749290	0.343988
C	-5.454881	-2.366773	1.359188
C	-5.379698	-1.890864	2.669035
C	-4.564565	-0.794423	2.968183
C	-3.828199	-0.174949	1.959402
Br	5.678182	0.613906	-1.255311
C	0.712005	-3.755073	-3.988602
H	0.458744	-1.333998	-2.778355
H	-1.295514	-2.681155	0.929752
H	-1.212150	-5.009699	0.100947
H	-4.221562	-0.418993	-2.325674
H	3.627775	0.132873	-1.121591
H	4.461978	-0.923215	0.826360
H	0.466067	-1.146093	2.444736
H	5.281647	-2.332882	2.686004
H	1.306288	-2.524676	4.323148
H	3.714978	-3.128299	4.442277
H	2.373424	2.536525	-1.341466
H	1.812355	1.496338	2.807761
H	2.624951	4.913979	-0.705180
H	2.057193	3.872419	3.432019
H	2.462042	5.590942	1.681811
H	-4.774810	-2.131157	-0.670071
H	-3.197310	0.675956	2.203043
H	-6.083188	-3.220344	1.123592
H	-4.501972	-0.422560	3.986308
H	-5.950620	-2.373379	3.456509
H	-1.657432	2.495846	-1.789711
H	-5.390449	1.559863	0.167329
H	-2.399819	4.835581	-2.074704

H	-6.122415	3.899845	-0.127372
H	-4.631862	5.545542	-1.241668
H	0.289978	-4.672513	-4.409027
H	0.525036	-2.937877	-4.691658
H	1.799094	-3.891881	-3.931457

Table S168. XYZ Coordinates of H_meta_IV_Me

91

scf done: -5611.946314

C	-1.435220	-3.545500	-1.246824
C	-1.538619	-2.179157	-1.545374
C	-1.652052	-1.774564	-2.884936
C	-1.665252	-2.723261	-3.907793
C	-1.560389	-4.083013	-3.604196
C	-1.445938	-4.491481	-2.273392
P	-1.476362	-0.895629	-0.237239
C	-2.227292	-1.698697	1.228349
C	-3.549624	-2.174871	1.190666
C	-4.101514	-2.786327	2.316180
C	-3.342523	-2.936279	3.481203
C	-2.030987	-2.460562	3.526253
C	-1.477898	-1.836373	2.405905
Pd	0.581790	0.154547	0.243651
P	2.821057	0.913565	0.476816
C	4.041485	-0.121641	1.366417
C	4.258300	0.100251	2.736383
C	5.150583	-0.705542	3.446245
C	5.829742	-1.738883	2.798234
C	5.611781	-1.969142	1.437207
C	4.718160	-1.170967	0.722681
P	-0.462260	2.209042	1.037604
O	0.398406	2.948297	2.232434
C	-0.609526	3.485862	-0.272733
C	-1.041653	3.135540	-1.562562
C	-1.127183	4.109440	-2.559672
C	-0.769306	5.431502	-2.283149
C	-0.330068	5.781931	-1.003907
C	-0.251165	4.815005	-0.000031
C	-2.088538	2.191357	1.885150
C	-3.254738	2.685619	1.285895
C	-4.468123	2.647871	1.975353
C	-4.527073	2.118703	3.266326
C	-3.367550	1.624330	3.869369
C	-2.154230	1.655542	3.181674
C	1.494705	-1.594557	-0.416460
C	1.911180	-1.738817	-1.748426
C	2.538103	-2.916418	-2.169721
C	2.751822	-3.964367	-1.271908
C	2.343684	-3.847436	0.062893
C	1.717532	-2.656923	0.469732

H	3.237153	-4.877406	-1.608509
O	-2.503708	0.245943	-0.775806
O	2.856036	2.322130	1.382838
C	3.614302	1.397390	-1.096459
C	5.009220	1.503862	-1.228611
C	5.566163	1.961126	-2.423234
C	4.739908	2.316256	-3.492436
C	3.351972	2.217957	-3.367211
C	2.790104	1.760812	-2.175097
Br	-5.377630	-0.484916	-1.825319
H	2.857924	-3.016022	-3.204346
H	1.752815	-0.939856	-2.466682
H	1.410946	-2.571639	1.509812
C	2.601882	-4.955707	1.057968
H	-3.411511	-0.061225	-1.094667
H	1.360115	2.823526	2.074589
H	-4.151430	-2.052086	0.293370
H	-0.463938	-1.449095	2.447967
H	-5.125258	-3.147320	2.283885
H	-1.441425	-2.566580	4.431981
H	-3.775899	-3.417841	4.352880
H	-1.748546	-0.721045	-3.126918
H	-1.350190	-3.874829	-0.216688
H	-1.762397	-2.400264	-4.940103
H	-1.370625	-5.547522	-2.031581
H	-1.571730	-4.821000	-4.400865
H	0.093372	5.085615	0.992738
H	-1.326709	2.110664	-1.777830
H	-0.048861	6.808164	-0.786315
H	-1.469558	3.833931	-3.552851
H	-0.829988	6.185463	-3.062445
H	5.660914	1.227656	-0.406266
H	1.710737	1.684818	-2.080562
H	6.644856	2.038413	-2.518118
H	2.707520	2.494066	-4.196047
H	5.177276	2.668929	-4.421596
H	3.740449	0.904983	3.247704
H	4.550279	-1.367943	-0.330693
H	5.315327	-0.520930	4.503385
H	6.137129	-2.771680	0.928585
H	6.525859	-2.362874	3.350584
H	-3.219920	3.101855	0.285264
H	-1.257665	1.272390	3.658796
H	-5.365702	3.036106	1.503342
H	-3.406707	1.213204	4.873699
H	-5.471970	2.092363	3.801019
H	3.727491	2.742967	1.462347
H	1.778588	-5.053798	1.772752
H	2.737130	-5.919524	0.558563
H	3.511466	-4.756616	1.638879

Table S169. XYZ Coordinates of H_meta_V_Me

90
scf done: -5611.492858

C	3.453602	0.877751	-1.972693
C	3.599622	1.468607	-0.708047
C	4.330726	2.660182	-0.591941
C	4.918961	3.241395	-1.717242
C	4.781368	2.639284	-2.970831
C	4.047888	1.456573	-3.096668
P	2.834077	0.749341	0.812015
O	2.882954	1.856363	1.923442
Pd	0.614812	0.010826	0.360343
C	1.362663	-1.896242	0.013161
C	1.606169	-2.358072	-1.289137
C	2.084678	-3.652940	-1.555944
C	2.313739	-4.515539	-0.476689
C	2.067660	-4.085279	0.829249
C	1.604050	-2.788417	1.072159
H	2.681910	-5.522714	-0.659136
P	-1.568284	-0.846353	-0.069252
O	-2.441784	0.261291	-0.891970
C	-2.481015	-1.226063	1.479435
C	-1.760002	-1.449391	2.662340
C	-2.427307	-1.753430	3.851226
C	-3.821701	-1.819831	3.869964
C	-4.546651	-1.585851	2.697510
C	-3.883965	-1.294701	1.504409
C	-1.780054	-2.368697	-1.078425
C	-1.781045	-2.258145	-2.478102
C	-1.897793	-3.396982	-3.276110
C	-2.007544	-4.658995	-2.685661
C	-2.004949	-4.776301	-1.293718
C	-1.893898	-3.637739	-0.492926
P	-0.179986	2.276479	0.785114
O	0.731271	3.048609	1.877671
C	4.082885	-0.494699	1.362371
C	4.242248	-0.693349	2.742394
C	5.167248	-1.621812	3.224434
C	5.946523	-2.362286	2.332343
C	5.798241	-2.166303	0.956787
C	4.872146	-1.239289	0.474458
C	-1.855121	2.572266	1.491412
C	-2.928432	3.075178	0.744021
C	-4.174399	3.274115	1.343016
C	-4.362367	2.972299	2.693351
C	-3.297268	2.470619	3.446247
C	-2.052282	2.270822	2.849088
C	-0.118575	3.335732	-0.718187
C	0.489480	4.598258	-0.654600

C	0.573593	5.397483	-1.796746
C	0.051427	4.943958	-3.010665
C	-0.551908	3.685286	-3.081458
C	-0.631601	2.879043	-1.943722
Br	-5.372479	-0.307409	-1.964005
C	2.304873	-4.111104	-2.980490
H	1.404036	-1.708747	-2.139400
H	1.447421	-2.474551	2.101322
H	2.247677	-4.759784	1.663802
H	-3.360395	-0.005638	-1.199436
H	1.676986	2.571738	1.946825
H	-4.459529	-1.113631	0.599212
H	-0.676363	-1.378218	2.653279
H	-5.631865	-1.631552	2.711673
H	-1.859127	-1.927828	4.760291
H	-4.343049	-2.048760	4.794944
H	-1.706939	-1.280540	-2.943894
H	-1.902456	-3.741260	0.587355
H	-1.907922	-3.297911	-4.357676
H	-2.097406	-5.753573	-0.828838
H	-2.099963	-5.545019	-3.306941
H	0.899602	4.943755	0.288929
H	-1.102418	1.902238	-2.000809
H	1.047316	6.373392	-1.738393
H	-0.957759	3.328410	-4.023862
H	0.117692	5.566384	-3.898373
H	4.427001	3.126775	0.383616
H	2.877074	-0.035543	-2.078670
H	5.482858	4.164620	-1.616238
H	3.932915	0.987508	-4.069842
H	5.238317	3.092163	-3.846044
H	3.651051	-0.105333	3.437358
H	4.772434	-1.095793	-0.596482
H	5.282302	-1.762367	4.295664
H	6.407255	-2.732157	0.257279
H	6.668386	-3.082934	2.705936
H	-2.795993	3.316747	-0.304734
H	-1.228388	1.887233	3.443171
H	-4.996737	3.668419	0.753022
H	-3.434479	2.235703	4.497811
H	-5.332028	3.128437	3.157176
H	2.966679	-4.981362	-3.024096
H	1.356591	-4.395114	-3.454128
H	2.744651	-3.318370	-3.594826

Table S170. XYZ Coordinates of H_meta_TS2_Me

90

scf done: -5611.454364

C	1.817404	0.639237	-2.660937
C	2.464044	1.322928	-1.616446

C	2.826580	2.667017	-1.798391
C	2.555593	3.312541	-3.006506
C	1.918702	2.626195	-4.043609
C	1.550948	1.289602	-3.867946
P	2.851592	0.555978	0.023914
O	3.254878	1.611603	1.081222
Pd	0.490119	0.167440	0.343635
C	2.030498	-1.311103	0.757382
C	2.241592	-1.372618	2.147071
C	2.468182	-2.593152	2.805628
C	2.513944	-3.761764	2.036441
C	2.336826	-3.713549	0.648262
C	2.108440	-2.499548	0.005172
H	2.704572	-4.715619	2.521670
P	-1.509533	-1.064237	-0.257612
C	-1.357008	-2.122368	-1.762954
C	-1.512137	-1.540747	-3.032865
C	-1.298262	-2.292390	-4.189613
C	-0.916077	-3.633430	-4.096102
C	-0.754473	-4.219519	-2.838361
C	-0.972425	-3.470546	-1.679669
C	-2.245992	-2.225773	0.972141
C	-1.460793	-2.634529	2.061184
C	-1.976370	-3.515042	3.017035
C	-3.286603	-3.981379	2.901061
C	-4.079899	-3.568413	1.825462
C	-3.565386	-2.699164	0.862994
O	-2.725127	-0.024557	-0.659891
P	-0.050561	2.348713	1.115290
O	1.259142	3.104890	1.750686
C	4.453916	-0.305894	-0.364646
C	5.385000	-0.441292	0.677272
C	6.610076	-1.071669	0.456991
C	6.920932	-1.579825	-0.807461
C	6.002272	-1.446397	-1.850095
C	4.776499	-0.811513	-1.631567
C	-0.749709	3.640380	-0.003431
C	-0.269911	4.958046	0.036429
C	-0.804290	5.928508	-0.813848
C	-1.823692	5.593473	-1.708980
C	-2.303012	4.281670	-1.755417
C	-1.765792	3.305322	-0.912929
C	-1.227414	2.395861	2.537205
C	-0.841225	2.939172	3.771020
C	-1.731929	2.948094	4.848053
C	-3.015509	2.417446	4.703390
C	-3.405773	1.872712	3.476175
C	-2.517407	1.855794	2.399646
Br	-5.487288	-0.894460	-2.036466
H	2.377175	-4.631494	0.067630

H	1.976022	-2.482059	-1.071598
H	2.245000	-0.452622	2.726695
C	2.648584	-2.633820	4.305568
H	-3.544695	-0.409071	-1.083208
H	2.080451	2.550991	1.526599
H	-4.195064	-2.384430	0.033479
H	-0.445498	-2.261962	2.160595
H	-5.101882	-3.926267	1.735844
H	-1.356884	-3.828000	3.852721
H	-3.691446	-4.660255	3.646255
H	-1.820164	-0.503172	-3.115035
H	-0.851340	-3.941900	-0.709179
H	-1.435292	-1.831289	-5.163742
H	-0.465249	-5.263565	-2.757003
H	-0.749988	-4.217895	-4.996350
H	0.524625	5.214536	0.729698
H	-2.135312	2.284144	-0.965047
H	-0.424611	6.945976	-0.776862
H	-3.091124	4.014993	-2.454272
H	-2.238156	6.349055	-2.370353
H	3.315332	3.202333	-0.990333
H	1.513088	-0.395696	-2.539535
H	2.843420	4.351843	-3.137063
H	1.052933	0.750237	-4.668370
H	1.709030	3.129717	-4.982656
H	5.149930	-0.039718	1.657499
H	4.083298	-0.704923	-2.459289
H	7.322901	-1.163605	1.271719
H	6.239212	-1.829923	-2.838509
H	7.874289	-2.071130	-0.979277
H	-2.826715	1.414127	1.456019
H	0.155471	3.353164	3.880491
H	-4.401763	1.454930	3.358178
H	-1.421696	3.371962	5.799265
H	-3.706789	2.424571	5.541246
H	3.156904	-3.549193	4.621133
H	3.231548	-1.778036	4.660365
H	1.680781	-2.600509	4.820829

Table S171. XYZ Coordinates of H_meta_VI_Me

90

scf done: -5611.487536

C	-2.243002	3.350497	-1.327495
C	-0.841705	3.451542	-1.279809
C	-0.192561	4.353520	-2.136690
C	-0.930877	5.148650	-3.017326
C	-2.323801	5.054091	-3.051275
C	-2.975963	4.154242	-2.203386
P	0.121879	2.370042	-0.128366
C	-0.166547	3.205376	1.499485

C	-1.421816	3.127847	2.127942
C	-1.625998	3.739896	3.366500
C	-0.582853	4.423062	3.999114
C	0.668754	4.492795	3.384373
C	0.876980	3.888681	2.141056
Pd	0.009250	0.011618	-0.312994
P	2.264603	-0.639186	-0.097682
O	3.201743	0.665140	-0.533716
C	-2.058922	-0.806201	-0.927944
C	-1.104133	-1.878376	-0.898759
C	-0.640870	-2.420417	-2.139795
C	-1.048799	-1.895243	-3.342123
C	-1.968927	-0.801752	-3.396366
C	-2.463149	-0.295639	-2.214553
P	-3.240886	-0.515561	0.431852
O	-3.552194	0.943384	0.710710
H	-0.686231	-2.326304	-4.272278
C	-2.629332	-1.376263	1.929910
C	-2.103682	-0.593157	2.968867
C	-1.653341	-1.193863	4.146883
C	-1.722458	-2.581105	4.296695
C	-2.250955	-3.367388	3.269011
C	-2.709093	-2.768745	2.094040
C	-4.767950	-1.411627	-0.052454
C	-5.994690	-0.925101	0.425719
C	-7.186207	-1.577253	0.105118
C	-7.163549	-2.721534	-0.696618
C	-5.947637	-3.208804	-1.182180
C	-4.754350	-2.555813	-0.865542
O	1.652361	2.896829	-0.479305
C	2.944715	-1.980176	-1.164261
C	3.426019	-1.685070	-2.449891
C	3.826532	-2.710199	-3.309741
C	3.742707	-4.043552	-2.902269
C	3.259543	-4.347528	-1.626152
C	2.862320	-3.324382	-0.763768
C	2.866929	-1.122185	1.575760
C	4.233978	-1.215889	1.891226
C	4.632262	-1.586415	3.175764
C	3.676436	-1.876987	4.155298
C	2.317723	-1.785617	3.850429
C	1.915405	-1.402240	2.567811
Br	6.359832	0.656369	-0.819760
H	-1.376668	-3.047566	5.214526
H	-2.317944	-4.444903	3.386117
H	-3.141673	-3.389328	1.315436
H	-2.060407	0.484729	2.856781
H	-1.254985	-0.577962	4.947781
H	4.199746	0.562426	-0.584845
H	2.272233	2.131718	-0.523555

H	-6.010122	-0.028196	1.036975
H	-3.816872	-2.935442	-1.260785
H	-8.130668	-1.191165	0.477369
H	-5.927385	-4.092864	-1.812678
H	-8.090834	-3.228613	-0.946935
H	-0.979148	-2.475391	-0.001322
H	-3.191217	0.510784	-2.245225
H	0.040383	-3.266003	-2.114535
C	-2.394898	-0.247469	-4.734693
H	0.888849	4.430120	-2.108689
H	-2.761242	2.639144	-0.688859
H	-0.415611	5.843764	-3.674783
H	-4.059295	4.071363	-2.229072
H	-2.896809	5.672690	-3.736259
H	3.506408	-0.651431	-2.770820
H	2.496614	-3.575229	0.228225
H	4.207392	-2.465036	-4.297202
H	3.198121	-5.381560	-1.298706
H	4.053941	-4.839993	-3.571869
H	4.986604	-0.984725	1.141041
H	0.857413	-1.314186	2.335007
H	5.690329	-1.650638	3.413886
H	1.569647	-2.004138	4.606776
H	3.992409	-2.168903	5.152805
H	-2.234879	2.579773	1.658327
H	1.846971	3.950566	1.659176
H	-2.601573	3.678196	3.841411
H	1.485615	5.020555	3.869243
H	-0.744406	4.893532	4.964867
H	-3.145718	0.539303	-4.621485
H	-1.542668	0.177626	-5.278878
H	-2.818568	-1.031551	-5.373706

Table S172. XYZ Coordinates of H_meta_VII_Me

90

scf done: -5611.488678

C	2.175726	2.286375	1.598067
C	1.425756	3.474150	1.540563
C	1.634533	4.468410	2.506870
C	2.587252	4.282793	3.512996
C	3.336726	3.105752	3.561094
C	3.129291	2.109364	2.601695
P	0.158900	3.656569	0.219377
O	-0.506635	5.147451	0.600192
Pd	-1.396284	1.986342	0.031947
P	-3.136513	0.455797	-0.007903
O	-4.575603	1.146024	0.385196
C	-2.971714	-0.933050	1.197674
C	-2.256651	-2.097281	0.873437
C	-2.052298	-3.093317	1.830986

C	-2.556107	-2.938412	3.125424
C	-3.268003	-1.782860	3.456807
C	-3.471656	-0.785075	2.501484
C	-3.410796	-0.429721	-1.601437
C	-4.527885	-1.253973	-1.823428
C	-4.687394	-1.903728	-3.047667
C	-3.734574	-1.746616	-4.060119
C	-2.623161	-0.928128	-3.849196
C	-2.466983	-0.267419	-2.626881
C	1.160703	4.121941	-1.250754
C	0.690252	3.750655	-2.520214
C	1.389264	4.118834	-3.671809
C	2.567630	4.860577	-3.564274
C	3.045616	5.233469	-2.304275
C	2.348510	4.864856	-1.153245
Br	-7.267721	-0.523315	0.882194
O	1.881228	-0.403643	-0.590712
P	2.600849	-1.729133	-0.423197
C	4.136899	-1.776148	-1.415761
C	4.589020	-0.570298	-1.970654
C	5.752896	-0.543717	-2.742130
C	6.470723	-1.720472	-2.964223
C	6.023438	-2.926785	-2.416490
C	4.860711	-2.957023	-1.646569
C	3.064700	-2.088544	1.305678
C	4.384432	-1.940972	1.758243
C	4.715773	-2.116290	3.108809
C	3.688287	-2.436216	4.007612
C	2.370097	-2.581528	3.571719
C	2.052035	-2.409200	2.225014
C	6.145549	-1.988878	3.579722
C	1.573545	-3.135752	-0.976148
C	0.560213	-2.870768	-1.910223
C	-0.241591	-3.906440	-2.392992
C	-0.034945	-5.215180	-1.948935
C	0.970544	-5.486403	-1.017325
C	1.770925	-4.451635	-0.528823
H	3.923652	-2.571959	5.060199
H	-0.659338	-6.021058	-2.323205
H	1.127623	-6.501205	-0.664358
H	2.534083	-4.671555	0.211426
H	0.394465	-1.851354	-2.243373
H	-1.029046	-3.688201	-3.108139
H	-5.354968	0.532368	0.511740
H	-1.432284	5.009955	0.848211
H	4.020726	0.337987	-1.798386
H	4.519710	-3.902096	-1.234805
H	6.095889	0.393783	-3.169586
H	6.577799	-3.843835	-2.592027
H	7.375034	-1.700502	-3.565333

H	1.023304	-2.523207	1.896100
H	5.171107	-1.689370	1.053291
H	1.588008	-2.828318	4.283602
H	1.049213	5.381329	2.469766
H	2.015635	1.497475	0.865296
H	2.741760	5.058405	4.257922
H	3.704490	1.188356	2.639117
H	4.074824	2.961957	4.345037
H	-4.036141	0.104539	2.763151
H	-1.863687	-2.235501	-0.129240
H	-3.669142	-1.658263	4.458649
H	-1.506329	-3.992997	1.561197
H	-2.398952	-3.714563	3.868759
H	-5.280724	-1.376024	-1.047908
H	-1.609743	0.381304	-2.461094
H	-5.556221	-2.534532	-3.213519
H	-1.884731	-0.795548	-4.634725
H	-3.863346	-2.255252	-5.011306
H	-0.225905	3.170426	-2.598285
H	2.730920	5.152227	-0.178437
H	1.016596	3.824341	-4.648583
H	3.963095	5.808600	-2.218825
H	3.114840	5.145008	-4.458398
H	6.753514	-1.427982	2.864838
H	6.201721	-1.483671	4.548754
H	6.607644	-2.975964	3.702653

Table S173. XYZ Coordinates of H_meta_I_OMe

67

scf done: -4806.597887

C	2.029306	1.218077	2.724929
C	2.672402	1.605037	1.539573
C	3.916772	2.252996	1.614965
C	4.505647	2.502590	2.854075
C	3.856147	2.114319	4.030932
C	2.617764	1.473031	3.966477
P	1.875497	1.197219	-0.066346
Pd	-0.306113	0.459859	-0.002161
P	-2.517728	-0.182584	0.036063
O	-2.873417	-1.585962	-0.793904
O	2.975593	0.141552	-0.749825
C	2.213354	2.697843	-1.071577
C	3.093437	2.679312	-2.162248
C	3.299786	3.836771	-2.919240
C	2.630535	5.018036	-2.595669
C	1.748359	5.042135	-1.510335
C	1.537695	3.889002	-0.755315
C	-3.738249	0.996586	-0.669825
C	-5.121411	0.854330	-0.468729
C	-6.014060	1.745519	-1.063636

C	-5.535882	2.790940	-1.860819
C	-4.163022	2.942032	-2.064780
C	-3.268270	2.047544	-1.472234
C	-3.243017	-0.579125	1.677827
C	-3.276058	0.419193	2.666629
C	-3.783890	0.140249	3.934908
C	-4.257780	-1.141439	4.234189
C	-4.222251	-2.139207	3.258908
C	-3.716741	-1.861928	1.984998
C	2.343605	-3.308628	0.459879
C	1.324985	-2.848593	-0.395636
C	1.216286	-3.402250	-1.664790
C	2.077900	-4.397018	-2.124920
C	3.080572	-4.836529	-1.259754
C	3.226110	-4.306994	0.024938
O	2.381379	-2.718456	1.683998
Br	-0.175659	-2.774432	-2.836331
H	4.015018	-4.674475	0.668924
H	2.561762	-0.729325	-0.855085
H	-2.083643	-1.891177	-1.270088
H	0.645020	-2.071607	-0.049909
H	-5.500050	0.050487	0.155838
H	-2.197283	2.159022	-1.625121
H	-7.081932	1.628403	-0.903495
H	-3.788506	3.753959	-2.681305
H	-6.233435	3.486270	-2.318702
H	-3.692034	-2.636616	1.226176
H	-2.904873	1.417072	2.444926
H	-4.589120	-3.136190	3.485878
H	-3.807305	0.920435	4.690202
H	-4.649575	-1.359375	5.223392
H	4.422658	2.566140	0.706153
H	1.064494	0.719598	2.665393
H	5.468107	3.003476	2.904153
H	2.109959	1.172703	4.878377
H	4.315249	2.314541	4.994718
H	3.614777	1.762121	-2.413661
H	0.844513	3.915108	0.082264
H	3.985423	3.812360	-3.761473
H	1.222210	5.957467	-1.255348
H	2.791201	5.915203	-3.186240
H	1.971895	-4.814402	-3.118712
H	3.765429	-5.610369	-1.592873
C	3.407182	-3.111471	2.598458
H	3.259852	-2.500533	3.488631
H	3.318344	-4.170899	2.862556
H	4.402857	-2.918547	2.184464

Table S174. XYZ Coordinates of H_meta_TS1_OMe

67

scf done: -4806.567404

C	3.026654	1.557657	1.545764
C	2.252926	2.005732	0.460301
C	1.783537	3.329013	0.462957
C	2.092832	4.188824	1.520010
C	2.866123	3.736657	2.590719
C	3.331312	2.418176	2.600619
P	1.742750	0.840147	-0.873014
C	3.354549	0.158642	-1.461203
C	3.347988	-1.109467	-2.065662
C	4.521625	-1.650339	-2.595334
C	5.715744	-0.929393	-2.526566
C	5.734184	0.333305	-1.927400
C	4.562250	0.874598	-1.396868
Pd	-0.100601	-0.578606	-0.403262
C	0.719355	-2.066552	0.875958
C	2.064636	-2.198622	1.280468
C	2.345967	-2.085042	2.633607
C	1.337798	-1.884315	3.596681
C	0.007110	-1.818860	3.183230
C	-0.318102	-1.969375	1.815373
Br	0.114457	-3.274718	-0.878994
O	-1.063176	-1.671091	4.013704
P	-2.081525	0.507666	-1.185372
O	-2.069929	0.744820	-2.837732
C	-2.391737	2.198111	-0.525253
C	-2.301354	3.332756	-1.343737
C	-2.469680	4.610601	-0.801802
C	-2.724257	4.767427	0.561416
C	-2.809475	3.640714	1.385974
C	-2.640678	2.365347	0.848138
C	-3.701709	-0.329633	-0.908438
C	-3.708479	-1.724238	-0.741112
C	-4.912595	-2.413546	-0.583206
C	-6.122636	-1.716174	-0.589471
C	-6.127411	-0.328354	-0.756468
C	-4.925152	0.362152	-0.914685
O	1.324273	1.889276	-2.106727
H	1.605841	-1.812549	4.643044
H	3.380383	-2.137507	2.963152
H	2.854180	-2.329656	0.550582
H	-1.360037	-2.064214	1.532439
H	-2.708815	1.497558	1.499570
H	-2.095978	3.220298	-2.403523
H	-3.007621	3.755837	2.447643
H	-2.401285	5.481238	-1.447683
H	-2.853658	5.760488	0.981440
H	3.395793	0.536868	1.566615

H	1.176669	3.688280	-0.362135
H	3.935704	2.060012	3.429080
H	1.727965	5.211832	1.504261
H	3.105454	4.405452	3.412214
H	-2.766833	-2.267137	-0.734022
H	-4.904800	-3.491638	-0.452164
H	-7.059387	-2.250768	-0.461793
H	-7.066884	0.216611	-0.759612
H	-4.940452	1.441221	-1.034862
H	-2.917945	1.074605	-3.176916
H	4.592127	1.853044	-0.927265
H	6.661476	0.896088	-1.871450
H	6.629888	-1.350089	-2.935200
H	4.503479	-2.633548	-3.056391
H	2.421964	-1.676820	-2.109724
H	2.044445	2.495463	-2.345487
C	-0.819107	-1.586325	5.416769
H	-1.797837	-1.481864	5.885668
H	-0.329923	-2.492576	5.791960
H	-0.203292	-0.713598	5.662540

Table S175. XYZ Coordinates of H_meta_IIa_OMe

67

scf done: -4806.641845

C	-1.400952	3.061619	-1.584219
C	-0.992070	1.735160	-1.779430
C	-1.765042	0.886543	-2.590127
C	-2.927489	1.360250	-3.196761
C	-3.330785	2.684458	-2.997004
C	-2.567006	3.531964	-2.192763
P	0.558124	1.071645	-1.070017
C	1.300462	2.441364	-0.114172
C	0.995045	2.592664	1.247737
C	1.556457	3.640956	1.977901
C	2.433900	4.535073	1.358365
C	2.747413	4.382565	0.005574
C	2.182614	3.340614	-0.732253
Pd	0.527792	-0.907018	0.093792
Br	0.918856	-3.162584	1.268118
P	-1.735195	-0.944846	1.007256
O	-1.722633	-1.830980	2.401431
C	-2.541328	0.570720	1.646154
C	-3.374962	1.363133	0.841656
C	-3.923516	2.544097	1.345774
C	-3.642372	2.949622	2.651958
C	-2.808361	2.169292	3.456777
C	-2.257733	0.987964	2.958284
C	-2.999890	-1.756061	-0.041900
C	-4.356431	-1.789815	0.324591
C	-5.278939	-2.482020	-0.459126

C	-4.858294	-3.148945	-1.614177
C	-3.511840	-3.127459	-1.982483
C	-2.585880	-2.436204	-1.197996
C	2.489060	-1.022124	-0.522607
C	2.832328	-1.678953	-1.722944
C	4.176312	-1.805294	-2.091972
C	5.199404	-1.287487	-1.294066
C	4.860922	-0.629898	-0.103447
C	3.511195	-0.495432	0.269540
O	5.765946	-0.078984	0.757276
O	1.445367	1.003043	-2.456158
H	6.231786	-1.397374	-1.602772
H	-4.692548	-1.272432	1.217766
H	2.123494	0.302422	-2.378798
H	-0.945711	-2.433654	2.356708
H	4.434421	-2.320037	-3.014131
H	2.062986	-2.117645	-2.353387
H	3.295229	0.031306	1.194889
H	-1.536669	-2.428385	-1.480092
H	-6.325456	-2.501186	-0.169921
H	-3.181352	-3.647839	-2.876376
H	-5.579677	-3.685333	-2.223510
H	-1.624106	0.377222	3.592514
H	-3.607441	1.059792	-0.173655
H	-2.589612	2.475983	4.475355
H	-4.571692	3.144665	0.714927
H	-4.072280	3.867554	3.041723
H	2.428127	3.218596	-1.781950
H	0.321101	1.896190	1.737873
H	3.432344	5.073565	-0.476597
H	1.314402	3.754432	3.030253
H	2.875566	5.345882	1.929932
H	-1.459229	-0.142943	-2.748708
H	-0.813625	3.729564	-0.963943
H	-3.517727	0.697402	-3.822068
H	-2.875330	4.561405	-2.037790
H	-4.237279	3.052655	-3.467987
C	7.150853	-0.171736	0.433091
H	7.683352	0.327256	1.243532
H	7.480185	-1.215628	0.372392
H	7.376514	0.333432	-0.513468

Table S176. XYZ Coordinates of H_meta_IIb_OMe

67

scf done: -4806.643018

C	-3.697746	1.372032	-0.742330
C	-2.969702	0.680271	-1.725096
C	-3.052555	1.095747	-3.063076
C	-3.859288	2.180697	-3.413228
C	-4.586531	2.859634	-2.433718

C	-4.504418	2.452685	-1.099131
P	-1.861222	-0.701742	-1.274570
O	-1.674472	-1.446974	-2.730156
Pd	0.240804	-0.223450	-0.305098
C	-0.712059	0.994779	1.013256
C	-0.696677	2.384542	0.794802
C	-1.341552	3.237136	1.691278
C	-2.014734	2.740912	2.813347
C	-2.029885	1.358247	3.031316
C	-1.380891	0.493055	2.130444
O	-2.649509	0.747546	4.087312
P	2.286996	0.327695	0.726661
O	2.101056	0.919376	2.259884
C	3.193043	1.654884	-0.155452
C	3.622738	1.450229	-1.478679
C	4.271300	2.473285	-2.169601
C	4.488726	3.710329	-1.554717
C	4.054449	3.921996	-0.245311
C	3.406441	2.900234	0.453518
C	3.496139	-1.029567	0.950611
C	3.007156	-2.309161	1.263455
C	3.890590	-3.353559	1.533628
C	5.269898	-3.132082	1.486091
C	5.763933	-1.864001	1.172532
C	4.882704	-0.813865	0.907388
Br	1.434382	-1.678084	-2.122033
C	-2.895065	-1.864282	-0.307748
C	-2.261228	-2.742367	0.586976
C	-3.002348	-3.697534	1.284165
C	-4.383629	-3.783469	1.094872
C	-5.021899	-2.916337	0.204177
C	-4.283305	-1.961389	-0.495742
H	-2.508500	3.424620	3.493264
H	-1.324046	4.310719	1.519071
H	-0.181637	2.803456	-0.064905
H	-1.426828	-0.571611	2.336930
H	2.946071	0.992527	2.732038
H	-0.718821	-1.666552	-2.832731
H	3.065889	3.073107	1.468898
H	3.444915	0.496142	-1.966127
H	4.217602	4.881702	0.235871
H	4.604979	2.305411	-3.189281
H	4.991660	4.505785	-2.096483
H	1.935797	-2.486016	1.284191
H	5.276126	0.168030	0.664821
H	3.504007	-4.339618	1.772817
H	6.835034	-1.690179	1.133383
H	5.957762	-3.947218	1.690228
H	-2.497503	0.561292	-3.826034
H	-3.636755	1.070124	0.298201

H	-3.921116	2.490423	-4.452281
H	-5.067743	2.977414	-0.333291
H	-5.215045	3.701809	-2.707821
H	-1.186933	-2.677143	0.737185
H	-4.788892	-1.291699	-1.183723
H	-2.502767	-4.369374	1.975618
H	-6.095272	-2.982747	0.053833
H	-4.961650	-4.523557	1.640290
C	-3.333675	1.566885	5.029969
H	-3.750193	0.887350	5.774600
H	-2.651136	2.269248	5.523320
H	-4.148140	2.129514	4.558099

Table S177. XYZ Coordinates of H_meta_III_OMe

67

scf done: -4806.613052

C	-3.857858	0.145260	2.034804
C	-3.915976	-0.541937	0.809427
C	-4.691485	-1.705007	0.706111
C	-5.415157	-2.164353	1.809485
C	-5.362940	-1.472682	3.020592
C	-4.582597	-0.317395	3.132204
P	-2.897242	0.045670	-0.588567
Pd	-0.566026	0.091128	-0.175509
P	1.724138	0.260399	0.275032
O	2.645083	-0.173396	-0.980113
C	-3.541249	1.703851	-1.009475
C	-4.818085	2.148980	-0.627724
C	-5.261503	3.414237	-1.013673
C	-4.439106	4.243443	-1.781183
C	-3.169147	3.807750	-2.167754
C	-2.719709	2.544764	-1.782348
O	-3.298965	-0.927616	-1.857414
C	-0.400924	-1.865915	-0.550814
C	0.121080	-2.271284	-1.785201
C	0.199711	-3.642045	-2.078544
C	-0.234226	-4.590258	-1.138217
C	-0.742776	-4.163930	0.083086
C	-0.833893	-2.798211	0.393320
O	0.687417	-4.147016	-3.248730
C	2.342250	-0.583266	1.771907
C	3.702270	-0.897724	1.927540
C	4.138655	-1.526351	3.094382
C	3.231099	-1.838262	4.110860
C	1.878175	-1.527035	3.959843
C	1.432068	-0.909826	2.789458
C	2.020299	2.049994	0.547450
C	1.863622	2.627714	1.818340
C	1.996978	4.007228	1.982416
C	2.284504	4.820728	0.883309

C	2.439546	4.252068	-0.383820
C	2.302609	2.874174	-0.555351
Br	5.683221	0.522688	-1.122188
H	-0.161296	-5.644482	-1.384864
H	0.469689	-1.535017	-2.497654
H	-1.241987	-2.486128	1.348376
H	-1.077879	-4.898992	0.810096
H	-4.216727	-0.812416	-2.153178
H	3.624102	0.087383	-0.961137
H	4.417007	-0.649262	1.146568
H	0.376420	-0.684896	2.663703
H	5.190252	-1.771374	3.210639
H	1.169249	-1.773586	4.744464
H	3.577621	-2.327101	5.016595
H	2.433786	2.435530	-1.539308
H	1.653233	2.003200	2.681274
H	2.670424	4.880047	-1.239044
H	1.883644	4.444521	2.969831
H	2.390973	5.893356	1.014298
H	-4.728880	-2.253157	-0.229462
H	-3.254883	1.044338	2.132421
H	-6.016828	-3.063689	1.719933
H	-4.538278	0.222508	4.073113
H	-5.924991	-1.832617	3.876996
H	-1.730991	2.208741	-2.084725
H	-5.462711	1.512880	-0.030263
H	-2.528582	4.450494	-2.763627
H	-6.249015	3.752264	-0.714845
H	-4.787372	5.228638	-2.076400
C	1.149650	-3.236292	-4.242674
H	1.486589	-3.850291	-5.078614
H	0.346766	-2.571371	-4.582803
H	1.988106	-2.632789	-3.875844

Table S178. XYZ Coordinates of H_meta_VI_OMe

92

scf done: -5687.152054

C	-2.460152	2.283054	2.082509
C	-3.343195	1.958876	1.038263
C	-4.713714	2.232871	1.185898
C	-5.188620	2.815834	2.361095
C	-4.304473	3.130957	3.395942
C	-2.940111	2.866103	3.255312
P	-2.644306	1.299795	-0.516860
O	-2.539806	2.648385	-1.504771
Pd	-0.499071	0.304406	-0.266781
C	-1.591859	-1.310848	0.451789
C	-2.044637	-1.349469	1.782783
C	-2.796527	-2.435567	2.235574
C	-3.109624	-3.506104	1.391101

C	-2.655313	-3.473659	0.067628
C	-1.904393	-2.377439	-0.392902
O	-2.891230	-4.453692	-0.856457
P	1.427595	-0.952973	0.259337
O	2.598698	0.088855	0.696022
C	1.356442	-2.131976	1.662190
C	1.062250	-3.490283	1.473826
C	0.975515	-4.348788	2.571121
C	1.181424	-3.860780	3.863584
C	1.476408	-2.509040	4.057607
C	1.561753	-1.647151	2.963718
C	2.046001	-1.949193	-1.148138
C	3.269478	-2.636617	-1.059012
C	3.729625	-3.383730	-2.143014
C	2.975163	-3.459524	-3.318095
C	1.762617	-2.774936	-3.414884
C	1.302981	-2.016513	-2.335885
P	0.766229	2.212720	-1.108995
C	2.368036	1.988408	-1.973534
C	3.588880	2.395249	-1.419325
C	4.777882	2.207627	-2.126634
C	4.758019	1.614709	-3.390777
C	3.543725	1.206269	-3.948707
C	2.354219	1.387261	-3.242761
C	-3.992061	0.361974	-1.326308
C	-4.209872	0.528486	-2.703882
C	-5.204356	-0.206838	-3.351694
C	-5.984904	-1.114678	-2.633734
C	-5.766424	-1.290358	-1.264820
C	-4.772308	-0.561965	-0.611636
O	-0.016725	3.030673	-2.305600
C	1.076059	3.484573	0.177737
C	1.497135	3.110120	1.464532
C	1.704089	4.084135	2.443456
C	1.479879	5.432238	2.152144
C	1.052745	5.807825	0.876046
C	0.852205	4.839825	-0.109820
Br	5.392946	-0.889134	1.745324
H	-3.693700	-4.337469	1.766953
H	-3.148983	-2.454446	3.264106
H	-1.820536	-0.538543	2.468529
H	-1.589103	-2.394045	-1.431815
H	3.473285	-0.301583	1.018023
H	-0.986847	3.003918	-2.151065
H	3.870077	-2.571599	-0.155312
H	0.367776	-1.470673	-2.418993
H	4.677813	-3.908152	-2.070662
H	1.177891	-2.824184	-4.328549
H	3.335762	-4.046838	-4.157465
H	1.806357	-0.601613	3.120469

H	0.904318	-3.881928	0.474683
H	1.645669	-2.125374	5.059367
H	0.752221	-5.399920	2.414211
H	1.116260	-4.531366	4.715339
H	0.517263	5.130375	-1.100166
H	1.680478	2.064596	1.690333
H	0.875750	6.854531	0.646594
H	2.037329	3.788769	3.434034
H	1.635457	6.186984	2.917381
H	-5.411369	1.988709	0.391669
H	-1.398709	2.078948	1.977374
H	-6.249096	3.022146	2.467758
H	-2.250131	3.110594	4.056979
H	-4.678370	3.581958	4.310171
H	-3.613103	1.236814	-3.268897
H	-4.605726	-0.716248	0.449046
H	-5.368790	-0.065286	-4.415505
H	-6.369228	-1.996678	-0.702231
H	-6.759820	-1.683979	-3.138152
H	3.615801	2.859888	-0.439956
H	1.415139	1.069328	-3.685120
H	5.718596	2.528744	-1.689438
H	3.521531	0.745583	-4.931828
H	5.684215	1.471669	-3.939504
H	-3.356348	3.168027	-1.584680
C	-3.646035	-5.592036	-0.452125
H	-3.712267	-6.237265	-1.329010
H	-3.149886	-6.135991	0.360516
H	-4.656806	-5.311698	-0.132370

Table S179. XYZ Coordinates of H_meta_V_OMe

91

scf done: -5686.698653

C	-0.780411	2.782683	-2.059880
C	-0.323537	3.340687	-0.854520
C	0.191132	4.645843	-0.852163
C	0.237754	5.386332	-2.035116
C	-0.228730	4.831197	-3.229423
C	-0.738583	3.530164	-3.239148
P	-0.343643	2.366764	0.706938
C	-2.034871	2.633632	1.388301
C	-3.140626	2.960172	0.591434
C	-4.398730	3.141597	1.169689
C	-4.567038	2.998412	2.548636
C	-3.469766	2.674089	3.350623
C	-2.212200	2.490613	2.774273
Pd	0.551150	0.110030	0.460462
P	-1.569503	-0.877575	0.010617
C	-1.657255	-2.427678	-0.974139
C	-1.637670	-2.341472	-2.375253

C	-1.662287	-3.499009	-3.154764
C	-1.699138	-4.755338	-2.543763
C	-1.716498	-4.848596	-1.150230
C	-1.696675	-3.691869	-0.368487
P	2.728346	0.986736	0.874118
C	4.069001	-0.152948	1.432660
C	4.353498	-0.216314	2.804742
C	5.356572	-1.062761	3.282928
C	6.087875	-1.854314	2.394457
C	5.814122	-1.792448	1.025143
C	4.811343	-0.947407	0.546720
C	3.431285	1.702671	-0.678185
C	3.240508	1.100235	-1.930569
C	3.791247	1.669250	-3.081563
C	4.526166	2.854271	-2.993283
C	4.707685	3.468988	-1.751141
C	4.162829	2.897174	-0.599741
O	2.714935	2.128122	1.950546
C	1.386893	-1.786623	0.334754
C	1.748731	-2.345434	-0.904062
C	2.301158	-3.632564	-0.980922
C	2.484110	-4.391157	0.184492
C	2.117618	-3.847286	1.411877
C	1.580515	-2.555179	1.492151
O	2.690975	-4.240336	-2.147053
O	-2.473449	0.160974	-0.868460
C	-2.513773	-1.283255	1.532675
C	-1.855077	-1.268150	2.771008
C	-2.543319	-1.590750	3.943171
C	-3.899700	-1.915483	3.886790
C	-4.567054	-1.917146	2.657684
C	-3.880720	-1.607382	1.483318
O	0.524623	3.244556	1.754948
Br	-5.348393	-0.569948	-1.991894
H	2.906400	-5.388276	0.104810
H	1.579361	-1.785222	-1.816437
H	1.325206	-2.154355	2.469441
H	2.255856	-4.432775	2.318249
H	-3.373968	-0.156130	-1.181776
H	1.479051	2.805761	1.886773
H	-4.411672	-1.603506	0.534027
H	-0.803596	-0.999537	2.814878
H	-5.624499	-2.161559	2.613906
H	-2.022585	-1.580203	4.896209
H	-4.438149	-2.161861	4.797378
H	-1.621959	-1.369067	-2.857256
H	-1.718638	-3.777897	0.713028
H	-1.659372	-3.418855	-4.237977
H	-1.752256	-5.821851	-0.669422
H	-1.719145	-5.655849	-3.150542

H	0.558818	5.071146	0.076079
H	-1.179776	1.773124	-2.070064
H	0.639053	6.395809	-2.023903
H	-1.100797	3.094342	-4.166000
H	-0.191650	5.408190	-4.148954
H	4.292180	3.375475	0.366469
H	2.659253	0.186473	-2.005938
H	5.271873	4.394796	-1.679864
H	3.640849	1.191546	-4.045630
H	4.949474	3.300172	-3.888744
H	3.797309	0.413008	3.492152
H	4.611957	-0.908357	-0.519056
H	5.569357	-1.099233	4.347810
H	6.384708	-2.399476	0.327765
H	6.869641	-2.511337	2.764874
H	-3.024058	3.076303	-0.480161
H	-1.362763	2.248383	3.405402
H	-5.246385	3.398252	0.540906
H	-3.591352	2.565020	4.424522
H	-5.546309	3.141372	2.996248
C	2.512132	-3.535115	-3.369900
H	2.887397	-4.193087	-4.155413
H	1.454316	-3.315543	-3.557401
H	3.081978	-2.597759	-3.383900

Table S180. XYZ Coordinates of H_meta_TS2_OMe

91

scf done: -5686.659765

C	-2.485410	1.548944	2.635124
C	-1.219634	2.147263	2.754121
C	-0.810604	2.641163	4.001450
C	-1.655482	2.543869	5.110412
C	-2.915735	1.955312	4.984772
C	-3.328408	1.459657	3.744318
P	-0.106648	2.250952	1.284569
C	-0.881943	3.617830	0.315385
C	-0.410330	4.933933	0.428958
C	-1.002238	5.960536	-0.310276
C	-2.070649	5.683157	-1.167006
C	-2.541843	4.372903	-1.287016
C	-1.947990	3.341398	-0.555867
Pd	0.447080	0.171171	0.276400
P	-1.549475	-1.055107	-0.346009
O	-2.816752	-0.019786	-0.556540
P	2.784399	0.667510	-0.127134
C	4.393723	-0.082974	-0.683981
C	5.375709	-0.311148	0.292926
C	6.609312	-0.862255	-0.055533
C	6.878394	-1.196566	-1.385648
C	5.908983	-0.969409	-2.364233

C	4.674343	-0.414428	-2.016824
C	2.287701	1.593750	-1.650366
C	1.594493	1.008318	-2.724091
C	1.247100	1.775522	-3.838282
C	1.579651	3.131731	-3.890434
C	2.262594	3.720354	-2.822990
C	2.614751	2.957925	-1.707484
O	3.207037	1.611683	1.024008
C	2.052753	-1.293132	0.407321
C	2.349087	-1.510338	1.769636
C	2.644482	-2.802214	2.229203
C	2.670714	-3.875023	1.325459
C	2.405413	-3.650178	-0.024515
C	2.109518	-2.368862	-0.494889
O	2.928483	-3.111126	3.527399
C	-1.458312	-1.929691	-1.969308
C	-1.681352	-1.206025	-3.153345
C	-1.518420	-1.814129	-4.399232
C	-1.119392	-3.151060	-4.482184
C	-0.889390	-3.877186	-3.311263
C	-1.056684	-3.272035	-2.063458
C	-2.178915	-2.372670	0.781359
C	-1.323507	-2.865921	1.778476
C	-1.756785	-3.864697	2.655583
C	-3.054701	-4.367336	2.553325
C	-3.917762	-3.872054	1.570110
C	-3.485165	-2.883767	0.685353
O	1.214249	2.980492	1.928149
Br	-5.626793	-0.823869	-1.877663
H	2.913656	-4.865891	1.695584
H	2.426010	-4.486857	-0.717696
H	1.907727	-2.219812	-1.549966
H	2.360482	-0.666971	2.449786
H	-3.647909	-0.382263	-0.976605
H	2.035690	2.472972	1.614133
H	-4.168078	-2.506136	-0.072596
H	-0.317901	-2.465720	1.868203
H	-4.930243	-4.258439	1.491900
H	-1.083103	-4.241502	3.419935
H	-3.395987	-5.138532	3.238058
H	-2.001992	-0.170349	-3.098365
H	-0.883091	-3.851588	-1.162074
H	-1.708190	-1.244731	-5.304811
H	-0.586263	-4.918972	-3.367008
H	-0.992992	-3.623944	-5.451741
H	0.422395	5.145500	1.091889
H	-2.310926	2.322376	-0.665272
H	-0.628770	6.976697	-0.216901
H	-3.368128	4.151252	-1.956925
H	-2.529646	6.482346	-1.742086

H	3.139402	3.417242	-0.875460
H	1.316989	-0.040777	-2.696444
H	2.523145	4.774337	-2.857791
H	0.713804	1.311523	-4.662837
H	1.307057	3.726135	-4.757436
H	5.173683	-0.044258	1.324947
H	3.940135	-0.233368	-2.794796
H	7.361181	-1.027826	0.711034
H	6.112781	-1.217481	-3.402070
H	7.838438	-1.625845	-1.657361
H	-2.811490	1.147054	1.679438
H	0.167890	3.099719	4.096635
H	-4.306199	0.997399	3.640825
H	-1.328034	2.930107	6.071824
H	-3.571311	1.879833	5.847589
C	2.931910	-2.059432	4.490067
H	3.175495	-2.527602	5.444325
H	3.688885	-1.302141	4.254741
H	1.949310	-1.578574	4.562899

Table S181. XYZ Coordinates of H_meta_VI_OMe

91

scf done: -5686.690250

C	2.020155	-1.187583	2.738017
C	2.948683	-0.987770	1.705533
C	4.322482	-1.048994	1.998883
C	4.750205	-1.313197	3.300150
C	3.817013	-1.527470	4.320263
C	2.451660	-1.464877	4.038345
P	2.307226	-0.643076	0.011463
C	2.970409	-2.065780	-0.955935
C	3.403197	-1.880944	-2.278855
C	3.784725	-2.974124	-3.059805
C	3.729249	-4.267553	-2.535211
C	3.293312	-4.462436	-1.221339
C	2.915371	-3.370794	-0.437888
Pd	0.049756	0.002847	-0.195037
C	-1.123055	-1.946937	-0.565137
C	-2.070793	-0.876728	-0.683983
C	-2.501692	-0.492332	-1.994997
C	-2.008980	-1.116849	-3.125098
C	-1.094394	-2.200677	-3.001405
C	-0.680812	-2.603099	-1.748835
P	-3.204922	-0.422207	0.673822
C	-4.751373	-1.351805	0.340445
C	-5.959008	-0.797868	0.793136
C	-7.164744	-1.469862	0.586994
C	-7.175229	-2.701239	-0.073362
C	-5.978468	-3.256583	-0.532783
C	-4.771105	-2.584407	-0.330943

O	-2.467795	-0.645603	-4.326638
P	0.142827	2.360592	-0.277439
O	1.668325	2.840948	-0.712413
C	-0.838201	3.296747	-1.537382
C	-2.241149	3.206208	-1.540296
C	-2.988105	3.896989	-2.496855
C	-2.348860	4.670554	-3.469993
C	-0.954704	4.751853	-3.480729
C	-0.202151	4.070304	-2.520423
C	-0.118979	3.390750	1.240410
C	-1.355783	3.374499	1.909053
C	-1.538717	4.134218	3.066578
C	-0.492432	4.907007	3.579244
C	0.741162	4.917651	2.925350
C	0.927888	4.165215	1.762191
O	3.231530	0.623771	-0.547515
O	-3.495832	1.061843	0.792619
C	-2.546729	-1.117109	2.236140
C	-1.956070	-0.232939	3.151945
C	-1.473294	-0.703136	4.375365
C	-1.574230	-2.059680	4.693827
C	-2.166379	-2.945538	3.789373
C	-2.657386	-2.476764	2.569490
Br	6.385048	0.594968	-0.906289
H	-0.732481	-2.722163	-3.878876
H	-1.203592	-2.424093	5.647469
H	-2.258249	-3.998589	4.038105
H	-3.140138	-3.171274	1.889032
H	-1.889052	0.822201	2.910482
H	-1.024980	-0.009285	5.080336
H	4.227977	0.518205	-0.613192
H	2.286453	2.074290	-0.682964
H	-5.948897	0.165120	1.294062
H	-3.848835	-3.018981	-0.704872
H	-8.094399	-1.031609	0.938087
H	-5.984361	-4.209347	-1.053968
H	-8.113604	-3.223818	-0.234517
H	-0.962882	-2.436448	0.389337
H	-3.228957	0.303842	-2.117458
H	0.000073	-3.443814	-1.655299
H	0.880215	4.137372	-2.527049
H	-2.749017	2.588678	-0.803215
H	-0.449484	5.348575	-4.235501
H	-4.072433	3.824102	-2.486732
H	-2.932841	5.201131	-4.216769
H	3.461669	-0.878439	-2.691050
H	2.586505	-3.536657	0.584387
H	4.128605	-2.813252	-4.077729
H	3.253752	-5.464363	-0.803219
H	4.025583	-5.117044	-3.143585

H	5.058050	-0.875506	1.216935
H	0.957057	-1.117713	2.522617
H	5.813468	-1.353641	3.519707
H	1.721171	-1.622094	4.826414
H	4.155554	-1.736343	5.331127
H	-2.170867	2.759905	1.534865
H	1.883600	4.180606	1.249285
H	-2.500155	4.117605	3.573061
H	1.560672	5.514303	3.316958
H	-0.637246	5.492350	4.482869
C	-2.037831	-1.288975	-5.522209
H	-2.534232	-0.763789	-6.339187
H	-0.952067	-1.214778	-5.655995
H	-2.333000	-2.345059	-5.541881

Table S182. XYZ Coordinates of H_meta_VII_OMe

91

scf done: -5686.693689

C	2.090895	2.382739	1.385759
C	1.271303	3.508774	1.576813
C	1.572352	4.413968	2.604597
C	2.684487	4.202955	3.424786
C	3.502085	3.088819	3.225154
C	3.202987	2.180600	2.204585
P	-0.195083	3.728374	0.488711
O	-0.903963	5.092386	1.157651
Pd	-1.630123	1.956817	0.277569
P	-3.239545	0.290092	0.184164
O	-4.714430	0.820759	0.680192
C	-2.917723	-1.174028	1.261927
C	-2.124922	-2.244636	0.817852
C	-1.804804	-3.295275	1.680758
C	-2.269464	-3.289122	2.998856
C	-3.058451	-2.227714	3.449060
C	-3.377749	-1.175352	2.588577
C	-3.500856	-0.480090	-1.470030
C	-4.553321	-1.375811	-1.727347
C	-4.706521	-1.930224	-2.998429
C	-3.810902	-1.605619	-4.023203
C	-2.763551	-0.715544	-3.776773
C	-2.614581	-0.150473	-2.506619
C	0.529808	4.461258	-1.034410
C	-0.084252	4.179319	-2.264886
C	0.399858	4.745897	-3.446080
C	1.504306	5.599610	-3.407581
C	2.124064	5.885308	-2.187051
C	1.642445	5.317900	-1.006966
C	2.595038	-2.476185	3.164104
C	2.174681	-2.248238	1.857442
C	3.127239	-1.930881	0.869825

C	4.478237	-1.844693	1.210408
C	4.894313	-2.078335	2.532206
C	3.947556	-2.395016	3.513675
P	2.533392	-1.524547	-0.810235
C	3.999775	-1.427634	-1.898446
C	4.392613	-0.157727	-2.346244
C	5.502688	-0.018998	-3.181763
C	6.226044	-1.146745	-3.575351
C	5.837345	-2.415743	-3.135915
C	4.727688	-2.558155	-2.302467
O	6.234272	-1.975055	2.750997
C	1.561678	-2.970889	-1.358714
C	0.487379	-2.740535	-2.232056
C	-0.279217	-3.808115	-2.702074
C	0.024046	-5.113751	-2.306849
C	1.090757	-5.350445	-1.435919
C	1.855871	-4.283938	-0.958988
O	1.724511	-0.242441	-0.860934
Br	-7.224642	-1.127312	1.120197
H	4.246435	-2.579336	4.538373
H	-0.573003	-5.944400	-2.671441
H	1.323079	-6.363290	-1.120867
H	2.668617	-4.477664	-0.265585
H	0.245031	-1.724476	-2.526400
H	-1.114845	-3.617545	-3.368759
H	-5.432800	0.132460	0.781351
H	-1.768067	4.840108	1.513748
H	3.819694	0.712585	-2.043114
H	4.428654	-3.550050	-1.977840
H	5.799483	0.967290	-3.525815
H	6.394981	-3.294411	-3.445839
H	7.088503	-1.039308	-4.226582
H	1.120835	-2.320028	1.607509
H	5.234152	-1.602902	0.471419
H	1.865584	-2.724020	3.929475
H	0.933757	5.277250	2.760812
H	1.862654	1.658379	0.605768
H	2.909669	4.909289	4.218993
H	3.830440	1.306884	2.051739
H	4.364437	2.924658	3.865031
H	-4.001441	-0.360315	2.942451
H	-1.761454	-2.267738	-0.205000
H	-3.429777	-2.219295	4.469933
H	-1.198768	-4.121061	1.318839
H	-2.022166	-4.107711	3.668489
H	-5.261551	-1.627821	-0.941158
H	-1.807992	0.552566	-2.310116
H	-5.525719	-2.617235	-3.191331
H	-2.070152	-0.453871	-4.570905
H	-3.934822	-2.040193	-5.011066

H	-0.942356	3.511956	-2.288575
H	2.135306	5.538578	-0.064859
H	-0.082158	4.518864	-4.392555
H	2.984753	6.547195	-2.155790
H	1.884500	6.038959	-4.325321
C	6.722833	-2.197670	4.072873
H	7.803454	-2.063611	4.018660
H	6.302410	-1.475274	4.781914
H	6.499838	-3.214528	4.415925

Table S183. XYZ Coordinates of H_mono_I

63

scf done: -4692.072337

C	0.991295	3.306368	1.254854
C	0.381008	3.281548	2.511364
C	-0.973774	3.600090	2.646801
C	-1.723866	3.948264	1.518645
C	-1.127816	3.976641	0.253126
C	0.222022	3.651648	0.144605
H	-1.440355	3.586622	3.626710
Br	1.046956	3.685997	-1.591558
O	-2.772581	0.663382	1.698697
P	-2.303890	-0.415123	0.508950
C	-3.430641	0.027317	-0.878204
C	-4.491710	0.929459	-0.713731
C	-5.320440	1.245795	-1.793677
C	-5.098759	0.663298	-3.043492
C	-4.039292	-0.232803	-3.215985
C	-3.204767	-0.542832	-2.141675
Pd	-0.059580	-0.498129	0.002473
P	2.186268	-0.558729	-0.504671
C	2.843406	-2.085671	-1.285497
C	4.219825	-2.350727	-1.373623
C	4.674544	-3.504111	-2.012820
C	3.760694	-4.407612	-2.564659
C	2.390256	-4.154771	-2.478205
C	1.933688	-2.998414	-1.841301
C	3.335129	-0.281006	0.908057
C	3.108241	-0.964719	2.113868
C	3.954034	-0.770291	3.206137
C	5.026618	0.122214	3.111496
C	5.250424	0.815802	1.920460
C	4.410198	0.615248	0.821289
O	2.730994	0.558609	-1.620784
C	-3.062700	-1.939000	1.200509
C	-4.452523	-2.133605	1.253215
C	-4.979679	-3.291788	1.824933
C	-4.125954	-4.270026	2.344345
C	-2.742577	-4.087541	2.292746
C	-2.213653	-2.927214	1.722887

H	-2.167300	1.422394	1.717341
H	2.172422	1.354572	-1.593373
H	2.040620	3.058196	1.144915
H	4.936006	-1.659751	-0.939147
H	0.867620	-2.796675	-1.768910
H	5.740737	-3.700988	-2.077200
H	1.677786	-4.855761	-2.903120
H	4.117646	-5.307387	-3.057293
H	4.583146	1.153987	-0.104798
H	2.263441	-1.644468	2.199236
H	6.079552	1.513500	1.844095
H	3.771762	-1.307023	4.132688
H	5.679974	0.279855	3.964627
H	-5.123311	-1.384662	0.843325
H	-1.137241	-2.780484	1.676203
H	-6.055848	-3.433796	1.861666
H	-2.076236	-4.846577	2.692016
H	-4.539438	-5.173002	2.784083
H	-4.660581	1.384918	0.256804
H	-2.368674	-1.223774	-2.284460
H	-6.138564	1.947324	-1.657376
H	-3.857430	-0.681244	-4.188394
H	-5.743248	0.911313	-3.881760
H	0.970325	3.015673	3.383351
H	-1.704409	4.246208	-0.624111
H	-2.773517	4.207521	1.617338

Table S184. XYZ Coordinates of H_mono_TS1

63

scf done: -4692.041611

C	-2.447254	3.029795	-1.458362
C	-2.502841	2.021803	-0.485521
C	-2.724012	2.379847	0.855792
C	-2.898647	3.716792	1.211328
C	-2.847666	4.715939	0.233711
C	-2.621691	4.369760	-1.099270
P	-2.180252	0.258750	-0.908461
C	-3.772785	-0.551756	-0.447113
C	-3.743746	-1.905558	-0.074066
C	-4.926110	-2.582599	0.232375
C	-6.150264	-1.913304	0.170713
C	-6.190912	-0.566045	-0.199884
C	-5.010535	0.112065	-0.506532
Pd	-0.130420	-0.669326	-0.091829
Br	0.069901	-3.390842	-0.188912
C	0.750401	-1.977231	1.359679
C	2.118760	-2.045498	1.687640
C	2.508456	-1.756996	2.990401
C	1.557091	-1.449141	3.979674
C	0.202829	-1.458105	3.660911

C	-0.221200	-1.772552	2.357871
H	1.878646	-1.239204	4.994961
P	1.670889	0.697450	-0.808175
O	1.203795	1.553559	-2.167636
C	3.279666	-0.035922	-1.340644
C	3.275573	-1.370036	-1.781174
C	4.444121	-1.962932	-2.264012
C	5.631380	-1.228788	-2.310909
C	5.647452	0.099179	-1.875312
C	4.480161	0.692892	-1.392449
C	2.193041	2.048682	0.331819
C	1.704157	3.352913	0.155109
C	2.020707	4.359104	1.071427
C	2.821140	4.074592	2.179220
C	3.305922	2.776773	2.367978
C	2.993824	1.770309	1.453841
O	-2.234073	0.256638	-2.577336
H	3.566111	-1.768210	3.239847
H	2.854500	-2.276566	0.925948
H	-1.276489	-1.890326	2.138192
H	-0.544023	-1.272054	4.427842
H	-2.766762	1.612843	1.625232
H	-2.265167	2.769921	-2.496302
H	-3.075045	3.979299	2.250401
H	-2.580673	5.140396	-1.863677
H	-2.981728	5.757120	0.511772
H	3.378726	0.767672	1.613261
H	1.075641	3.582037	-0.699626
H	3.931200	2.548259	3.226304
H	1.640109	5.364744	0.917256
H	3.065965	4.857052	2.891418
H	-2.790981	-2.426037	-0.023725
H	-4.890235	-3.628822	0.521639
H	-7.069989	-2.437600	0.413249
H	-7.141326	-0.042408	-0.246320
H	-5.054415	1.161026	-0.783871
H	-3.093846	0.537839	-2.930029
H	4.507586	1.723314	-1.051296
H	6.569286	0.672550	-1.910085
H	6.541910	-1.689603	-2.682644
H	4.427793	-2.996233	-2.598094
H	2.354892	-1.946093	-1.734722
H	1.911679	2.119082	-2.517004

Table S185. XYZ Coordinates of H_mono_IIa

63

scf done: -4692.115961

C	3.102362	1.385946	-0.725205
C	2.303662	0.596074	-1.566516
C	2.071356	1.019999	-2.886602

C	2.638369	2.205138	-3.356497
C	3.437740	2.982968	-2.514854
C	3.667464	2.571034	-1.200713
P	1.478969	-0.927431	-0.971583
C	2.695034	-1.728787	0.140384
C	4.066138	-1.769050	-0.166471
C	4.952225	-2.452707	0.665245
C	4.480370	-3.104090	1.809434
C	3.119325	-3.075792	2.118786
C	2.229809	-2.393372	1.285807
Pd	-0.823612	-0.920215	-0.159099
C	-2.805264	-1.073301	0.381191
C	-3.182004	-1.764150	1.547642
C	-4.534757	-1.939075	1.875850
C	-5.531267	-1.425708	1.044435
C	-5.169052	-0.735092	-0.115582
C	-3.819853	-0.555771	-0.440478
H	-6.578993	-1.559821	1.297638
P	-0.930439	1.055757	1.002114
O	-1.865458	0.975671	2.357276
C	-1.664679	2.407615	0.014489
C	-1.353270	2.531111	-1.348791
C	-1.908680	3.566211	-2.102216
C	-2.786220	4.474699	-1.504364
C	-3.106360	4.349736	-0.150175
C	-2.547635	3.321232	0.610544
C	0.577238	1.753295	1.768291
C	1.326180	0.930126	2.626561
C	2.453668	1.431839	3.275182
C	2.846067	2.758555	3.070419
C	2.106697	3.580422	2.218315
C	0.975661	3.082097	1.567443
Br	-1.140530	-3.164282	-1.378216
O	1.546024	-1.810387	-2.366080
H	4.441640	-1.263447	-1.050714
H	-2.546270	0.284695	2.244517
H	0.772841	-2.419576	-2.362865
H	-4.802105	-2.480969	2.779500
H	-2.422882	-2.189984	2.200352
H	-3.562954	-0.007731	-1.343362
H	-5.937267	-0.328617	-0.768785
H	1.169192	-2.379451	1.521364
H	6.010290	-2.477335	0.422134
H	2.749321	-3.584018	3.004154
H	5.173545	-3.633725	2.456263
H	1.465375	0.411176	-3.549145
H	3.294541	1.077789	0.297057
H	2.459627	2.516852	-4.381347
H	4.288300	3.169751	-0.541272
H	3.880653	3.903942	-2.882261

H	-2.799271	3.220538	1.661113
H	-0.680428	1.822107	-1.822190
H	-3.791844	5.051743	0.314972
H	-1.662317	3.657774	-3.155696
H	-3.223265	5.275117	-2.093849
H	1.028817	-0.100979	2.790332
H	0.406882	3.730567	0.910372
H	3.025041	0.788819	3.937609
H	2.406664	4.611623	2.058773
H	3.725168	3.148550	3.574608

Table S186. XYZ Coordinates of H_mono_IIb

63

scf done: -4692.117551

C	3.352939	1.644832	-1.381625
C	3.076127	1.640856	-0.002916
C	3.383341	2.773786	0.764891
C	3.974464	3.889079	0.165673
C	4.257791	3.883652	-1.200943
C	3.945501	2.760139	-1.972841
P	2.241313	0.196622	0.756989
Pd	0.079797	-0.158452	-0.112035
Br	1.017258	-1.322502	-2.259471
C	3.431103	-1.188870	0.621762
C	2.944566	-2.497560	0.779222
C	3.823023	-3.580384	0.774644
C	5.193956	-3.367085	0.604801
C	5.685176	-2.069537	0.444748
C	4.809670	-0.981877	0.455123
O	2.246395	0.537137	2.376106
P	-2.127494	-0.420531	-0.914205
C	-3.088288	-1.729912	-0.068182
C	-2.388517	-2.753464	0.591745
C	-3.077705	-3.813523	1.182728
C	-4.472316	-3.859718	1.120215
C	-5.176474	-2.847531	0.462357
C	-4.490143	-1.787286	-0.130783
C	-0.686001	0.837288	1.486170
C	-0.663031	2.240635	1.496804
C	-1.176400	2.956502	2.584048
C	-1.726419	2.281476	3.676713
C	-1.756147	0.885012	3.674041
C	-1.238738	0.167494	2.587635
H	-2.127324	2.837072	4.519870
O	-2.121052	-0.878610	-2.495189
C	-3.232538	1.034269	-0.975921
C	-3.419667	1.712460	-2.190305
C	-4.221491	2.854853	-2.240766
C	-4.839660	3.330363	-1.082703
C	-4.653592	2.661096	0.130096

C	-3.851405	1.521331	0.187701
H	-1.145229	4.043364	2.573603
H	-0.239209	2.786964	0.657950
H	-1.277551	-0.917826	2.607748
H	-2.182865	0.346849	4.517084
H	3.140996	0.521554	2.753087
H	-1.192615	-1.107540	-2.734984
H	3.159890	2.786883	1.826388
H	3.100318	0.780596	-1.988915
H	4.211576	4.760130	0.769209
H	4.161344	2.752964	-3.037092
H	4.716890	4.751467	-1.664983
H	1.877613	-2.664818	0.894277
H	5.200661	0.022640	0.329579
H	3.438191	-4.588541	0.894960
H	6.749599	-1.901908	0.310968
H	5.877192	-4.211101	0.594338
H	-2.949853	1.336940	-3.092617
H	-3.710217	1.014264	1.136673
H	-4.364862	3.368731	-3.186675
H	-5.132325	3.026406	1.033724
H	-5.464326	4.217784	-1.123825
H	-1.303463	-2.718037	0.641018
H	-5.046221	-1.005074	-0.637165
H	-2.527313	-4.598385	1.692734
H	-6.260556	-2.882937	0.411022
H	-5.009833	-4.681913	1.583242

Table S187. XYZ Coordinates of H_mono_III

63

scf done: -4692.087580

C	-4.691420	2.286195	0.039948
C	-3.436320	1.960380	-0.500966
C	-2.585199	2.992928	-0.937294
C	-2.984876	4.325353	-0.835272
C	-4.233473	4.639839	-0.292931
C	-5.084761	3.620929	0.142326
P	-2.860709	0.236250	-0.706202
O	-3.271556	-0.197202	-2.243131
Pd	-0.531507	0.048846	-0.317191
C	-0.467911	-1.749769	-1.186278
C	-0.026530	-1.869151	-2.508539
C	-0.018196	-3.127081	-3.123430
C	-0.440210	-4.260582	-2.424556
C	-0.874264	-4.134097	-1.103460
C	-0.891160	-2.880098	-0.478950
H	-0.429372	-5.234378	-2.905244
P	1.779128	0.002017	0.065300
C	2.412868	-1.231215	1.252794
C	3.747579	-1.666217	1.212174

C	4.200149	-2.602752	2.142681
C	3.333867	-3.104983	3.117758
C	2.005862	-2.674489	3.161908
C	1.542890	-1.747153	2.226630
C	2.187258	1.644575	0.769917
C	2.384263	1.841025	2.145922
C	2.609093	3.125371	2.645241
C	2.638298	4.221228	1.779636
C	2.440697	4.033189	0.408561
C	2.209604	2.753583	-0.095418
O	2.621155	-0.133267	-1.308748
C	-3.925402	-0.776477	0.378306
C	-4.741649	-1.788547	-0.145139
C	-5.502378	-2.587725	0.712018
C	-5.446986	-2.387410	2.092147
C	-4.625905	-1.386005	2.620389
C	-3.863852	-0.586223	1.769953
Br	5.669219	0.532490	-1.405585
H	0.324324	-3.214197	-4.151182
H	0.316411	-0.999744	-3.059711
H	-1.237425	-2.797250	0.546409
H	-1.204631	-5.008725	-0.549339
H	-4.180444	0.049041	-2.480785
H	3.605825	0.101782	-1.275003
H	4.431443	-1.270859	0.465099
H	0.504811	-1.426288	2.251636
H	5.232090	-2.939152	2.106980
H	1.328539	-3.066694	3.914484
H	3.692278	-3.833475	3.839008
H	2.064317	2.612372	-1.162329
H	2.375294	0.995028	2.825594
H	2.468020	4.881785	-0.268280
H	2.769505	3.267130	3.709762
H	2.816846	5.218218	2.170981
H	-4.782241	-1.956268	-1.216288
H	-3.228999	0.189331	2.190479
H	-6.135468	-3.366803	0.298427
H	-4.578764	-1.228423	3.693563
H	-6.038107	-3.010703	2.756202
H	-1.613167	2.756792	-1.362650
H	-5.358942	1.502478	0.382026
H	-2.321723	5.115100	-1.174448
H	-6.056110	3.864444	0.561726
H	-4.542361	5.677382	-0.208739

Table S188. XYZ Coordinates of H_mono_IV

88

scf done: -5572.626270

C	-1.609040	-3.623739	-1.009860
C	-1.592781	-2.273817	-1.389014

C	-1.620057	-1.942453	-2.753225
C	-1.666343	-2.946477	-3.720581
C	-1.680243	-4.289881	-3.336556
C	-1.651958	-4.625695	-1.981198
P	-1.480685	-0.921125	-0.156022
C	-2.311998	-1.587140	1.334606
C	-3.664248	-1.969698	1.288934
C	-4.284463	-2.473898	2.431852
C	-3.564993	-2.609184	3.623320
C	-2.223633	-2.226717	3.676003
C	-1.601099	-1.710153	2.537381
Pd	0.633210	0.035549	0.286371
P	2.909732	0.688985	0.470053
C	4.070468	-0.333468	1.448819
C	4.292455	-0.012370	2.798312
C	5.141537	-0.800453	3.577376
C	5.771948	-1.914280	3.019609
C	5.547997	-2.242913	1.680173
C	4.697695	-1.462600	0.896776
P	-0.296920	2.197498	0.917422
O	0.599574	2.971116	2.063217
C	-0.367025	3.384483	-0.480887
C	-0.797841	2.965139	-1.750292
C	-0.823676	3.869588	-2.813916
C	-0.407512	5.189786	-2.623796
C	0.030401	5.608364	-1.364699
C	0.050054	4.711585	-0.295038
C	-1.924225	2.329673	1.752094
C	-3.057771	2.847500	1.111457
C	-4.273467	2.927930	1.793060
C	-4.367249	2.493523	3.116948
C	-3.240479	1.975613	3.760967
C	-2.025080	1.888984	3.081734
C	1.446551	-1.810838	-0.224423
C	1.867315	-2.090769	-1.533442
C	2.420966	-3.334583	-1.861926
C	2.553987	-4.325953	-0.886882
C	2.134660	-4.061891	0.418786
C	1.591552	-2.814241	0.746434
H	2.980010	-5.292350	-1.141691
O	-2.426045	0.245698	-0.783103
O	3.020680	2.162608	1.258700
C	3.728750	1.002196	-1.133683
C	5.127587	1.018068	-1.268017
C	5.709927	1.347931	-2.492172
C	4.905811	1.665669	-3.589520
C	3.514653	1.658332	-3.462700
C	2.927166	1.328750	-2.241167
Br	-5.320169	-0.346704	-1.862567
H	2.745563	-3.525325	-2.881959

H	1.770528	-1.340695	-2.313088
H	1.288043	-2.632856	1.773902
H	2.232424	-4.823808	1.188277
H	-3.344374	-0.022004	-1.107379
H	1.553952	2.790418	1.915775
H	-4.235796	-1.856963	0.370791
H	-0.563012	-1.395637	2.586769
H	-5.330690	-2.762408	2.392451
H	-1.663662	-2.321656	4.601501
H	-4.051785	-3.006549	4.509123
H	-1.623032	-0.900962	-3.058615
H	-1.592796	-3.897060	0.039827
H	-1.696013	-2.679139	-4.772742
H	-1.669262	-5.668089	-1.677089
H	-1.716765	-5.071001	-4.090159
H	0.393991	5.034934	0.682035
H	-1.128645	1.942171	-1.897999
H	0.356853	6.633214	-1.214177
H	-1.165198	3.541191	-3.791176
H	-0.422034	5.889276	-3.454424
H	5.763619	0.770947	-0.424471
H	1.845030	1.326216	-2.147750
H	6.791298	1.355500	-2.587669
H	2.886979	1.906641	-4.312924
H	5.362727	1.918902	-4.541406
H	3.812646	0.855180	3.239157
H	4.524741	-1.735399	-0.138714
H	5.310965	-0.539486	4.617550
H	6.033950	-3.109426	1.242343
H	6.434357	-2.524800	3.625826
H	-2.995454	3.190471	0.084718
H	-1.153822	1.488591	3.590580
H	-5.145450	3.333709	1.288915
H	-3.306862	1.637682	4.790835
H	-5.313779	2.558967	3.645354
H	3.915650	2.535853	1.312291

Table S189. XYZ Coordinates of H_mono_V

87

scf done: -5572.173126

C	3.433952	0.919438	-2.051243
C	3.645455	1.374772	-0.740778
C	4.417298	2.528021	-0.534803
C	4.980361	3.204576	-1.618820
C	4.776735	2.737154	-2.920056
C	4.002667	1.593753	-3.134552
P	2.916971	0.524272	0.728711
O	3.021818	1.517500	1.938974
Pd	0.670627	-0.131785	0.280020
C	1.368090	-2.013684	-0.250239

C	1.566544	-2.373768	-1.594140
C	2.010850	-3.652242	-1.952908
C	2.248897	-4.615868	-0.970062
C	2.045064	-4.283219	0.371463
C	1.618953	-2.996528	0.722754
H	2.586468	-5.611522	-1.245367
P	-1.541655	-0.886375	-0.193363
O	-2.363922	0.314729	-0.930126
C	-2.465593	-1.345727	1.326746
C	-1.747041	-1.748641	2.462807
C	-2.419110	-2.126714	3.627514
C	-3.814022	-2.086424	3.671006
C	-4.534969	-1.672160	2.546977
C	-3.868869	-1.307654	1.375969
C	-1.815464	-2.323386	-1.307865
C	-1.859546	-2.109705	-2.694614
C	-2.022835	-3.184555	-3.569426
C	-2.136528	-4.484368	-3.070085
C	-2.092242	-4.704379	-1.691269
C	-1.934685	-3.630359	-0.813364
P	-0.068707	2.094368	0.951693
O	0.885799	2.726661	2.095073
C	4.161605	-0.784620	1.113096
C	4.373830	-1.108044	2.461978
C	5.298973	-2.091021	2.819755
C	6.025599	-2.761453	1.832898
C	5.824716	-2.440831	0.487698
C	4.898555	-1.459343	0.129354
C	-1.718235	2.339532	1.734972
C	-2.835242	2.813962	1.033636
C	-4.064362	2.961244	1.679724
C	-4.192285	2.636087	3.031873
C	-3.083347	2.163935	3.738867
C	-1.854559	2.014761	3.094603
C	-0.033412	3.317365	-0.423341
C	0.548730	4.576765	-0.214997
C	0.607644	5.505946	-1.255578
C	0.086777	5.186283	-2.512159
C	-0.489672	3.931711	-2.727422
C	-0.544913	2.996298	-1.691399
Br	-5.342196	0.080152	-1.976119
H	2.160395	-3.895506	-3.002571
H	1.356055	-1.659372	-2.386794
H	1.495156	-2.763267	1.777888
H	2.224451	-5.022085	1.149494
H	-3.304684	0.132805	-1.233270
H	1.826899	2.235343	2.080472
H	-4.441777	-0.990296	0.507536
H	-0.661313	-1.759781	2.436390
H	-5.620053	-1.634920	2.581096

H	-1.853480	-2.441314	4.499700
H	-4.338475	-2.372191	4.578218
H	-1.781936	-1.101558	-3.089191
H	-1.910042	-3.813540	0.256110
H	-2.064038	-3.005701	-4.639968
H	-2.187717	-5.711754	-1.296556
H	-2.263303	-5.320653	-3.751356
H	0.959912	4.817548	0.760102
H	-0.996131	2.023401	-1.860687
H	1.061343	6.478297	-1.085453
H	-0.893650	3.678417	-3.703564
H	0.133857	5.909735	-3.321025
H	4.564298	2.890284	0.478122
H	2.825579	0.037637	-2.225193
H	5.576158	4.097055	-1.448444
H	3.836409	1.229751	-4.144574
H	5.213946	3.264444	-3.763084
H	3.824035	-0.573992	3.230612
H	4.757800	-1.218798	-0.919394
H	5.455398	-2.328556	3.868396
H	6.392954	-2.951863	-0.284527
H	6.747479	-3.524492	2.109757
H	-2.750405	3.071096	-0.016070
H	-0.994995	1.657285	3.652848
H	-4.921027	3.332436	1.124651
H	-3.173322	1.913329	4.791982
H	-5.149126	2.752526	3.532570

Table S190. XYZ Coordinates of H_mono_TS2

87

scf done: -5572.134444

C	-2.513361	2.227961	2.065385
C	-1.202923	2.705557	2.236383
C	-0.868749	3.378383	3.420557
C	-1.830486	3.575648	4.415371
C	-3.133291	3.105623	4.237034
C	-3.471986	2.432649	3.059091
P	0.063711	2.412455	0.925740
C	-0.474683	3.583442	-0.395658
C	0.065792	4.875677	-0.473963
C	-0.349756	5.752854	-1.477958
C	-1.309183	5.349130	-2.410469
C	-1.847474	4.061550	-2.339703
C	-1.428883	3.177462	-1.342225
Pd	0.520196	0.128903	0.448735
P	-1.511499	-1.062504	-0.127350
O	-2.634332	-0.016012	-0.730574
P	2.916722	0.370905	0.236510
C	4.489114	-0.601457	0.026184
C	5.375911	-0.647257	1.113286

C	6.574870	-1.355469	1.021687
C	6.902632	-2.032099	-0.156392
C	6.027527	-1.989436	-1.243451
C	4.828706	-1.277043	-1.154389
C	2.668964	0.992926	-1.490827
C	2.033942	0.254690	-2.504834
C	1.882323	0.800457	-3.781631
C	2.353890	2.086324	-4.058471
C	2.979722	2.827024	-3.052593
C	3.136719	2.285838	-1.775114
O	3.313244	1.505733	1.210594
C	1.961205	-1.362395	1.106907
C	2.080933	-1.287969	2.508821
C	2.193614	-2.451617	3.277624
C	2.214910	-3.701411	2.656670
C	2.129928	-3.785088	1.261065
C	2.017832	-2.628582	0.490956
C	-1.345218	-2.312215	-1.476280
C	-1.403547	-1.893546	-2.816538
C	-1.175707	-2.799205	-3.854209
C	-0.876569	-4.134283	-3.569363
C	-0.811454	-4.559169	-2.240133
C	-1.042984	-3.655740	-1.200459
C	-2.385594	-2.013288	1.189885
C	-1.685182	-2.330345	2.363992
C	-2.305895	-3.052515	3.387613
C	-3.637070	-3.449420	3.253059
C	-4.346063	-3.125592	2.091456
C	-3.726741	-2.415425	1.062483
O	1.370751	3.171473	1.563247
Br	-5.375377	-0.885959	-2.146398
H	2.312171	-4.605139	3.251086
H	2.147963	-4.755717	0.772734
H	1.957804	-2.716328	-0.589036
H	2.097620	-0.317867	2.997200
H	2.275251	-2.376547	4.358521
H	-3.454545	-0.403427	-1.150112
H	2.170375	2.554060	1.464577
H	-4.291244	-2.168154	0.165950
H	-0.653742	-2.010082	2.477574
H	-5.384109	-3.428972	1.986779
H	-1.751099	-3.296139	4.289253
H	-4.123429	-4.004736	4.050031
H	-1.647365	-0.861246	-3.047306
H	-0.997092	-4.002345	-0.172607
H	-1.237383	-2.462449	-4.885277
H	-0.587125	-5.597057	-2.010382
H	-0.699968	-4.838607	-4.377056
H	0.814518	5.184881	0.248293
H	-1.842264	2.172701	-1.300483

H	0.075659	6.751281	-1.531981
H	-2.588646	3.741091	-3.066748
H	-1.631082	6.032286	-3.191356
H	3.616618	2.864153	-0.991834
H	1.648689	-0.740744	-2.306731
H	3.347803	3.827467	-3.261518
H	1.392350	0.219626	-4.557652
H	2.233018	2.508564	-5.051643
H	5.128459	-0.115536	2.026103
H	4.170619	-1.243662	-2.016322
H	7.254093	-1.376421	1.869353
H	6.278386	-2.504657	-2.166362
H	7.835534	-2.583767	-0.227878
H	-2.784510	1.689856	1.160876
H	0.143136	3.745272	3.555814
H	-4.482996	2.061498	2.915699
H	-1.560288	4.098642	5.328704
H	-3.879729	3.259080	5.011122

Table S191. XYZ Coordinates of H_mono_VI

87

scf done: -5572.167470

C	-2.256123	3.350788	-1.471587
C	-0.855828	3.451239	-1.399464
C	-0.191659	4.357213	-2.240400
C	-0.914584	5.156935	-3.129616
C	-2.306732	5.062950	-3.187944
C	-2.973693	4.159115	-2.355935
P	0.086661	2.364427	-0.236107
C	-0.226241	3.194377	1.389420
C	-1.488942	3.106608	2.001340
C	-1.713371	3.714802	3.238271
C	-0.683171	4.403969	3.885462
C	0.575724	4.483880	3.287004
C	0.804216	3.883798	2.045291
Pd	-0.029636	0.003901	-0.420454
P	2.219595	-0.641749	-0.134579
O	3.164440	0.644197	-0.608232
C	-2.074575	-0.813093	-1.081649
C	-1.115439	-1.885210	-1.034128
C	-0.615200	-2.429191	-2.260606
C	-0.990066	-1.904450	-3.475844
C	-1.908502	-0.818023	-3.531913
C	-2.445314	-0.300410	-2.377185
P	-3.290467	-0.526012	0.250007
O	-3.599613	0.932843	0.531156
H	-0.604588	-2.330831	-4.397632
C	-2.722942	-1.400406	1.756990
C	-2.237173	-0.625795	2.821413
C	-1.823357	-1.237025	4.007399

C	-1.888556	-2.626240	4.139367
C	-2.377759	-3.404023	3.086152
C	-2.800168	-2.794999	1.903140
C	-4.808553	-1.409484	-0.282313
C	-6.044986	-0.920549	0.167811
C	-7.230981	-1.562789	-0.190722
C	-7.193164	-2.699468	-1.002671
C	-5.967442	-3.189169	-1.460180
C	-4.779460	-2.546070	-1.105638
O	1.624325	2.885884	-0.561249
C	2.922650	-2.027556	-1.124947
C	3.456653	-1.788173	-2.401023
C	3.876745	-2.850766	-3.204353
C	3.759276	-4.166096	-2.749643
C	3.222521	-4.414742	-1.482870
C	2.806012	-3.354215	-0.676805
C	2.783019	-1.050487	1.571467
C	4.140739	-1.198313	1.906324
C	4.509508	-1.505854	3.215932
C	3.532310	-1.679564	4.202180
C	2.182753	-1.534007	3.878321
C	1.810772	-1.213766	2.569499
Br	6.331352	0.635186	-0.769405
H	-1.570175	-3.100938	5.062819
H	-2.442123	-4.483120	3.189185
H	-3.203500	-3.409432	1.104206
H	-2.196874	0.453503	2.722123
H	-1.457037	-0.627701	4.828413
H	4.164417	0.545253	-0.612793
H	2.241585	2.118345	-0.600064
H	-6.071967	-0.029586	0.787270
H	-3.834137	-2.927641	-1.479706
H	-8.182905	-1.174792	0.159864
H	-5.935155	-4.067268	-2.098427
H	-8.116205	-3.198706	-1.282636
H	-1.013956	-2.483589	-0.134350
H	-3.175043	0.502254	-2.432213
H	0.065119	-3.274375	-2.212117
H	-2.206116	-0.413474	-4.495217
H	0.889153	4.433227	-2.193564
H	-2.785476	2.636653	-0.845156
H	-0.387955	5.855059	-3.774743
H	-4.056435	4.076795	-2.400685
H	-2.867711	5.685112	-3.879610
H	3.562232	-0.768598	-2.757560
H	2.398259	-3.561895	0.308796
H	4.298764	-2.648854	-4.184835
H	3.134121	-5.434521	-1.118969
H	4.085673	-4.991553	-3.375449
H	4.909441	-1.054346	1.151018

H	0.760743	-1.085390	2.319439
H	5.560737	-1.612088	3.468714
H	1.419020	-1.663420	4.639516
H	3.824982	-1.923434	5.219500
H	-2.292111	2.554322	1.519614
H	1.779946	3.953107	1.576145
H	-2.694494	3.645413	3.700500
H	1.382509	5.016368	3.783414
H	-0.860368	4.871334	4.849975

Table S192. XYZ Coordinates of H_mono_VII

87
scf done: -5572.167978

C	3.846964	-2.381227	2.377207
C	2.701567	-2.262203	1.576735
C	1.434124	-2.450797	2.156083
C	1.318208	-2.755971	3.511298
C	2.464905	-2.874308	4.302942
C	3.726522	-2.687412	3.735648
P	2.765576	-1.807999	-0.192242
C	2.231085	-3.292819	-1.112723
C	1.606247	-3.101901	-2.355054
C	1.193012	-4.200197	-3.109989
C	1.402116	-5.496368	-2.631349
C	2.019739	-5.693733	-1.393928
C	2.430242	-4.596929	-0.633394
H	2.373763	-3.111107	5.358889
O	1.910288	-0.598102	-0.516145
C	4.521682	-1.525184	-0.614444
C	4.976786	-0.197726	-0.649315
C	6.308545	0.080311	-0.960978
C	7.193943	-0.963187	-1.241346
C	6.746163	-2.286612	-1.213256
C	5.414739	-2.568706	-0.903630
C	2.210833	2.096981	1.706156
C	1.522207	3.314911	1.568991
C	1.735669	4.336037	2.505637
C	2.633569	4.146881	3.560434
C	3.322955	2.939387	3.687457
C	3.110111	1.915969	2.758163
P	0.329381	3.502897	0.181278
C	1.419375	3.891623	-1.247795
C	1.001248	3.495781	-2.528104
C	1.770147	3.803603	-3.652547
C	2.966486	4.509122	-3.506641
C	3.392642	4.906099	-2.235444
C	2.625795	4.597577	-1.111382
Pd	-1.280733	1.890378	-0.045897
P	-3.115850	0.478061	-0.129220
C	-3.460404	-0.316473	-1.756013

C	-4.687957	-0.936076	-2.047262
C	-4.897244	-1.522199	-3.296175
C	-3.885979	-1.506417	-4.262387
C	-2.663696	-0.893733	-3.980687
C	-2.456023	-0.293690	-2.735712
C	-3.048124	-0.967225	1.018942
C	-3.478009	-0.812025	2.347019
C	-3.355006	-1.860745	3.260036
C	-2.793304	-3.076870	2.861806
C	-2.357914	-3.239143	1.543615
C	-2.483246	-2.191704	0.627784
O	-4.500659	1.250914	0.301804
O	-0.300563	5.025860	0.488628
Br	-7.277291	-0.263736	0.826107
H	1.079262	-6.350813	-3.218810
H	2.175422	-6.699911	-1.016495
H	2.893940	-4.760033	0.334848
H	1.436005	-2.093280	-2.718495
H	0.706123	-4.044530	-4.068034
H	-5.314070	0.685064	0.436367
H	-1.239573	4.925290	0.702153
H	4.284316	0.611310	-0.439843
H	5.074210	-3.599491	-0.896980
H	6.652867	1.109828	-0.988023
H	7.430940	-3.098870	-1.437756
H	8.229566	-0.746099	-1.485944
H	0.536414	-2.359051	1.551423
H	4.831976	-2.237346	1.945774
H	0.333838	-2.900328	3.946386
H	4.618806	-2.777985	4.347815
H	1.196356	5.272504	2.407797
H	2.042971	1.288912	0.996332
H	2.792097	4.943487	4.281972
H	3.637337	0.971256	2.857615
H	4.018106	2.793050	4.509231
H	-3.926502	0.125374	2.661388
H	-2.149561	-2.335178	-0.395681
H	-3.702079	-1.729031	4.280928
H	-1.928035	-4.184166	1.224017
H	-2.701762	-3.894255	3.571157
H	-5.483015	-0.953592	-1.304827
H	-1.511457	0.199661	-2.517348
H	-5.850740	-1.994251	-3.516245
H	-1.877090	-0.873197	-4.729539
H	-4.053696	-1.966074	-5.232269
H	0.071062	2.943136	-2.635507
H	2.968095	4.903201	-0.127255
H	1.437602	3.490417	-4.637948
H	4.323994	5.453018	-2.120048
H	3.567915	4.746489	-4.379414

Table S193. XYZ Coordinates of H_para_I_P(O)Ph$_2$

86
scf done: -5571.382643

C	0.424284	3.464091	-4.399167
C	1.473882	3.030521	-3.586930
C	1.217156	2.558663	-2.296223
C	-0.097132	2.515430	-1.809833
C	-1.149491	2.943230	-2.636427
C	-0.889212	3.421081	-3.920790
P	-0.489978	1.882335	-0.127177
C	-0.683924	3.419567	0.859409
C	0.080819	4.572202	0.616564
C	-0.072844	5.700765	1.422063
C	-0.994363	5.692628	2.473879
C	-1.761629	4.552346	2.720920
C	-1.607248	3.421062	1.916626
Pd	-2.169402	0.315825	0.027879
P	-3.863658	-1.229522	0.243946
C	-4.607309	-1.402648	1.915689
C	-4.389553	-2.543915	2.699915
C	-4.916015	-2.621166	3.992821
C	-5.658965	-1.561039	4.514558
C	-5.877927	-0.418178	3.738380
C	-5.354008	-0.337705	2.448672
O	1.013946	1.392322	0.412943
O	-3.433104	-2.817408	-0.037135
C	-5.339177	-1.018714	-0.831690
C	-6.519272	-1.755764	-0.634735
C	-7.598999	-1.600245	-1.503219
C	-7.514956	-0.703458	-2.573893
C	-6.347567	0.034596	-2.776992
C	-5.263349	-0.124437	-1.910114
Br	-0.442995	-3.183488	-1.959997
C	1.094208	-2.534561	-1.017998
C	1.011184	-2.286740	0.352219
C	2.276802	-2.335931	-1.727865
C	3.402364	-1.868933	-1.047630
C	3.348751	-1.602564	0.327973
H	1.051515	0.423516	0.436271
H	-2.563613	-2.841459	-0.468342
H	0.088561	-2.458425	0.894109
C	2.147507	-1.821056	1.019418
P	4.761099	-0.934348	1.288813
H	-6.595081	-2.447447	0.199262
H	-4.349953	0.445917	-2.062427
H	-8.507415	-2.174116	-1.344584
H	-6.279963	0.732319	-3.606584
H	-8.359471	-0.580974	-3.245851
H	-3.816589	-3.370499	2.293877

H	-5.531826	0.554748	1.853639
H	-4.744936	-3.512441	4.589821
H	-6.456492	0.409724	4.137803
H	-6.065540	-1.622370	5.519751
H	0.793184	4.589960	-0.202727
H	-2.203834	2.530762	2.101226
H	0.523025	6.587803	1.227667
H	-2.480529	4.543697	3.534976
H	-1.115315	6.574711	3.095974
H	2.033566	2.222693	-1.665175
H	-2.174499	2.898455	-2.275300
H	2.495325	3.059527	-3.955640
H	-1.709977	3.751830	-4.550634
H	0.626177	3.829048	-5.401977
H	2.107043	-1.638426	2.088621
H	2.325143	-2.544386	-2.790282
H	4.324169	-1.733394	-1.604026
O	4.533097	-1.107354	2.773580
C	6.246458	-1.776221	0.648221
C	4.903664	0.829074	0.830861
C	4.719307	1.774645	1.849418
C	4.783306	3.139841	1.561719
C	5.030520	3.567711	0.256164
C	5.216643	2.629701	-0.764584
C	5.153142	1.265340	-0.480699
H	4.527242	1.431740	2.860957
H	4.640664	3.866124	2.356197
H	5.080695	4.629095	0.031982
H	5.415126	2.960424	-1.779574
H	5.307793	0.549756	-1.282514
C	7.464555	-1.089857	0.518886
C	8.621184	-1.773307	0.140334
C	8.573563	-3.146784	-0.107875
C	7.367446	-3.839799	0.026133
C	6.209814	-3.160087	0.404218
H	7.511661	-0.022493	0.708734
H	9.557153	-1.232562	0.038290
H	9.473767	-3.676612	-0.404798
H	7.327582	-4.907793	-0.165156
H	5.277762	-3.708058	0.504681

Table S194. XYZ Coordinates of H_para_TS1_P(O)Ph_2

86
scf done: -5571.355924

C	-2.817308	-2.566620	1.673772
C	-3.615655	-1.410971	1.617462
C	-4.277717	-0.983162	2.776810
C	-4.153640	-1.704212	3.968206
C	-3.364185	-2.854295	4.014137
C	-2.695967	-3.284416	2.863082

P	-3.675772	-0.429596	0.061911
O	-4.904638	0.661162	0.347033
Pd	-1.631042	0.658435	-0.573664
Br	-1.414930	1.580426	-3.163669
P	-0.981312	2.071708	1.216906
O	-2.309627	2.310304	2.202190
C	0.256524	1.415262	2.412991
C	-0.179281	0.675312	3.525139
C	0.744138	0.095848	4.398077
C	2.114219	0.241566	4.169900
C	2.557328	0.970226	3.062073
C	1.637508	1.549373	2.186263
C	-0.385977	3.790039	0.905828
C	-0.650590	4.363090	-0.348657
C	-0.288006	5.685256	-0.616654
C	0.339876	6.450178	0.368158
C	0.605643	5.891203	1.621785
C	0.247424	4.569678	1.889711
C	0.057561	0.643542	-1.859104
C	1.264392	1.354343	-1.681448
C	2.446388	0.648965	-1.511394
C	2.473069	-0.763795	-1.555390
C	1.278081	-1.451182	-1.806388
C	0.078130	-0.761817	-2.012986
P	4.012060	-1.731861	-1.463691
O	4.353325	-2.459942	-2.746158
C	-4.438171	-1.612554	-1.129525
C	-4.139316	-1.464598	-2.494179
C	-4.732463	-2.298324	-3.444468
C	-5.629378	-3.289852	-3.040995
C	-5.934261	-3.445559	-1.685736
C	-5.342925	-2.613351	-0.734571
C	5.345310	-0.574833	-0.981841
C	6.260644	-0.196920	-1.975546
C	7.303181	0.682191	-1.675872
C	7.440859	1.187086	-0.381125
C	6.537111	0.808896	0.616073
C	5.494260	-0.069485	0.319177
C	3.787413	-2.887755	-0.065373
C	4.401536	-4.146386	-0.151564
C	4.286259	-5.059048	0.898193
C	3.559280	-4.720886	2.042600
C	2.942226	-3.470498	2.134328
C	3.051003	-2.557667	1.083461
H	3.362965	1.203103	-1.335863
H	1.259739	2.437705	-1.639136
H	-0.810031	-1.299093	-2.326526
H	1.275818	-2.534353	-1.895779
H	-2.293954	-2.912245	0.785719
H	-4.887643	-0.085874	2.751379

H	-2.081428	-4.179464	2.891729
H	-4.675436	-1.364757	4.858320
H	-3.267725	-3.412920	4.940384
H	1.998469	2.118115	1.335248
H	-1.242230	0.559752	3.712881
H	3.621027	1.100034	2.885318
H	0.391094	-0.465314	5.258415
H	2.832129	-0.204430	4.851716
H	4.956066	-4.407724	-1.047501
H	4.760508	-6.033043	0.821905
H	3.469888	-5.431479	2.859076
H	2.372871	-3.204738	3.020006
H	2.555362	-1.594572	1.160551
H	4.806542	-0.365579	1.105023
H	6.647782	1.193057	1.625783
H	8.253109	1.868883	-0.146825
H	8.007915	0.968593	-2.450890
H	6.155097	-0.604189	-2.975948
H	-3.438926	-0.695701	-2.809704
H	-4.491481	-2.175639	-4.496280
H	-6.087624	-3.941866	-3.778848
H	-6.629626	-4.217366	-1.369007
H	-5.580952	-2.750054	0.315999
H	-5.761252	0.233020	0.507314
H	0.469266	4.145856	2.864598
H	1.094036	6.483732	2.389885
H	0.623542	7.477840	0.160820
H	-0.494296	6.114700	-1.592619
H	-1.134633	3.767909	-1.118229
H	-2.109766	2.856041	2.980161

Table S195. XYZ Coordinates of H_para_IIa_P(O)Ph$_2$

86

scf done: -5571.429715

C	4.439353	2.276033	3.651411
C	3.751057	1.060447	3.720660
C	2.759080	0.766763	2.786170
C	2.443791	1.689956	1.774538
C	3.136776	2.906663	1.710470
C	4.130758	3.196135	2.648150
P	1.087007	1.275596	0.621645
C	0.932277	2.696051	-0.517028
C	0.122318	3.791451	-0.180005
C	0.007482	4.870126	-1.058655
C	0.699890	4.863953	-2.272093
C	1.506361	3.774421	-2.611994
C	1.618703	2.689478	-1.741722
Pd	1.096329	-0.732937	-0.491593
Br	0.668752	-2.923438	-1.768146
P	3.483827	-1.222673	-0.665196

C	4.719431	0.085472	-1.005689
C	4.939941	0.480153	-2.336735
C	5.834152	1.511339	-2.626726
C	6.513090	2.163184	-1.594086
C	6.294084	1.780110	-0.269383
C	5.400250	0.749128	0.026820
C	-0.943992	-0.478672	-0.516002
C	-1.756264	-1.035988	0.488814
C	-3.151806	-0.935145	0.430385
C	-3.771221	-0.267972	-0.636061
C	-2.966576	0.287522	-1.643467
C	-1.575387	0.186991	-1.580205
O	-0.171387	1.443722	1.673312
O	3.730786	-2.157197	-2.003737
C	4.183998	-2.198491	0.718439
C	5.554677	-2.501249	0.792103
C	6.041373	-3.308629	1.819529
C	5.167033	-3.823447	2.782014
C	3.802929	-3.534691	2.712882
C	3.312948	-2.727939	1.683476
H	6.241376	-2.103192	0.051591
H	-0.930469	0.909044	1.375542
H	2.879195	-2.605497	-2.209860
H	-3.747534	-1.397951	1.212171
H	-1.307536	-1.578098	1.317852
H	-0.981603	0.631216	-2.374507
H	-3.436217	0.794238	-2.481753
P	-5.574911	-0.028088	-0.762234
H	2.249856	-2.511896	1.624895
H	7.101998	-3.535866	1.870388
H	3.120790	-3.936368	3.456107
H	5.549827	-4.449880	3.582270
H	4.428149	-0.034183	-3.143069
H	5.245104	0.460399	1.061017
H	6.003659	1.800711	-3.659639
H	6.818897	2.281362	0.538104
H	7.210616	2.963935	-1.821151
H	-0.418677	3.793313	0.760437
H	2.238534	1.840843	-2.015808
H	-0.623500	5.713639	-0.795373
H	2.041425	3.764789	-3.556677
H	0.607496	5.704017	-2.953908
H	2.229988	-0.179327	2.845556
H	2.903104	3.629557	0.936766
H	3.987241	0.342113	4.499697
H	4.661373	4.141724	2.592888
H	5.213240	2.503463	4.378362
O	-6.020710	0.209479	-2.189125
C	-6.336296	-1.499728	0.007801
C	-5.988129	1.399616	0.305688

C	-6.841924	2.378467	-0.222632
C	-7.187876	3.496890	0.539016
C	-6.682248	3.644779	1.832030
C	-5.827153	2.674507	2.363789
C	-5.479065	1.556976	1.604838
H	-7.222846	2.258485	-1.231734
H	-7.848818	4.250998	0.122063
H	-6.949667	4.514946	2.424348
H	-5.429197	2.790012	3.367524
H	-4.807159	0.815528	2.026794
C	-7.395149	-1.390450	0.921898
C	-8.023075	-2.536653	1.414192
C	-7.601533	-3.800403	0.996602
C	-6.550562	-3.919365	0.082402
C	-5.922118	-2.776588	-0.410879
H	-7.729562	-0.412774	1.253104
H	-8.839324	-2.440874	2.123913
H	-8.089297	-4.690938	1.381893
H	-6.220030	-4.900787	-0.244135
H	-5.104516	-2.879021	-1.118640

Table S196. XYZ Coordinates of H_para_IIb_P(O)Ph_2

86

scf done: -5571.432295

C	0.104383	5.009975	3.352947
C	-0.565774	3.790514	3.472400
C	-0.984433	3.109818	2.327982
C	-0.735763	3.643296	1.052569
C	-0.066333	4.873439	0.942272
C	0.351419	5.550803	2.088142
P	-1.395119	2.766939	-0.413444
O	-2.754559	3.602161	-0.813644
Pd	-1.892563	0.483768	-0.050949
P	-2.295006	-1.812719	0.308162
C	-3.561901	-2.238861	1.558773
C	-3.647150	-1.440777	2.712202
C	-4.534644	-1.774090	3.734436
C	-5.351591	-2.901563	3.610267
C	-5.276045	-3.697137	2.464997
C	-4.383551	-3.370981	1.441802
C	0.091039	0.080296	-0.180554
C	0.611159	-0.398831	-1.394412
C	1.966832	-0.703943	-1.524321
C	2.844413	-0.531316	-0.442154
C	2.333743	-0.037096	0.767438
C	0.972936	0.259187	0.896092
Br	-4.445584	1.025325	-0.049368
O	-0.951143	-2.618078	0.837897
C	-2.779194	-2.704669	-1.217203
C	-1.949633	-3.699180	-1.755919

C	-2.304074	-4.340418	-2.945540
C	-3.482830	-3.991393	-3.606345
C	-4.309038	-2.994370	-3.078229
C	-3.959865	-2.348166	-1.892861
C	-0.840587	3.615145	-3.022180
C	-0.009273	3.905396	-4.105993
C	1.376811	3.797127	-3.976491
C	1.932796	3.396895	-2.758355
C	1.107463	3.103599	-1.672611
P	4.592823	-0.980683	-0.693580
H	2.350845	-1.066036	-2.473854
H	-0.041829	-0.539781	-2.251565
H	0.611186	0.641446	1.845865
H	2.992907	0.129141	1.615000
H	-1.153055	-3.517395	1.142724
H	-3.527227	3.010872	-0.655023
H	-1.028777	-3.970766	-1.250856
H	-4.598938	-1.565620	-1.494511
H	-1.656850	-5.111181	-3.353123
H	-5.225371	-2.717720	-3.591052
H	-3.756455	-4.490754	-4.530961
H	-3.025717	-0.554444	2.801094
H	-4.331689	-3.995242	0.555772
H	-4.594987	-1.151144	4.621679
H	-5.911303	-4.572099	2.365823
H	-6.047716	-3.157087	4.403592
C	-0.287492	3.216273	-1.795415
H	-1.916305	3.711399	-3.120505
H	1.551380	2.792431	-0.732509
H	-0.446730	4.219769	-5.048919
H	3.010057	3.312389	-2.651790
H	2.021936	4.024458	-4.819952
H	-1.508077	2.162738	2.425227
H	0.132450	5.300886	-0.035105
H	-0.760674	3.367922	4.453434
H	0.869899	6.500129	1.993247
H	0.433614	5.538329	4.242739
C	5.576487	0.064857	0.440931
C	4.785039	-2.702689	-0.105335
O	5.029757	-0.822134	-2.132685
C	6.104903	1.257309	-0.078786
C	6.855276	2.108127	0.733464
C	7.085882	1.774779	2.070859
C	6.570548	0.585995	2.592929
C	5.821766	-0.269255	1.781574
H	5.936792	1.505954	-1.122104
H	7.262624	3.027210	0.322677
H	7.671220	2.436364	2.702659
H	6.756649	0.319813	3.629145
H	5.440911	-1.198907	2.193130

C	5.792550	-3.480235	-0.698072
C	5.997870	-4.798081	-0.288260
C	5.198739	-5.350808	0.716118
C	4.189567	-4.585718	1.305719
C	3.979117	-3.267528	0.895119
H	6.402450	-3.052720	-1.487816
H	6.777413	-5.393585	-0.754157
H	5.357896	-6.377027	1.034008
H	3.561265	-5.015706	2.080059
H	3.181249	-2.686712	1.347883

Table S197. XYZ Coordinates of H_para_III_P(O)Ph$_2$

86

scf done: -5571.401352

C	4.533688	5.687159	-1.471030
C	4.406033	4.386473	-1.965055
C	3.325201	3.596760	-1.573270
C	2.359506	4.105321	-0.685666
C	2.493859	5.415212	-0.195767
C	3.578298	6.199770	-0.589230
P	0.945076	3.028555	-0.265739
C	0.211403	3.733110	1.251092
C	-1.117120	4.179955	1.273512
C	-1.674336	4.659102	2.461801
C	-0.914384	4.687483	3.632105
C	0.408288	4.233187	3.617536
C	0.969581	3.753258	2.435057
Pd	1.578864	0.747050	-0.106175
P	2.338045	-1.463211	0.011947
C	4.165290	-1.325031	0.027106
C	4.853345	-1.238515	-1.195697
C	6.233673	-1.037138	-1.209983
C	6.936670	-0.907256	-0.009000
C	6.257038	-0.984780	1.209291
C	4.876867	-1.191208	1.230894
C	-0.296107	0.130359	-0.375079
C	-0.710243	-0.307345	-1.639306
C	-2.031093	-0.715976	-1.834369
C	-2.952667	-0.697738	-0.775562
C	-2.523984	-0.269247	0.488731
C	-1.203687	0.147362	0.690035
O	1.947716	-2.328558	-1.293829
C	1.887941	-2.414802	1.503746
C	1.917988	-3.819019	1.511802
C	1.585305	-4.512254	2.676193
C	1.228588	-3.816786	3.835119
C	1.196792	-2.420684	3.830921
C	1.516244	-1.720323	2.666006
O	-0.179241	3.277918	-1.445225
Br	3.346060	-5.039672	-1.921618

P	-4.656345	-1.248701	-1.131870
H	-2.342969	-1.068786	-2.813029
H	-0.011857	-0.347597	-2.468440
H	-0.897099	0.476615	1.677579
H	-3.209400	-0.264566	1.331147
H	-0.318900	4.217613	-1.647275
H	2.440272	-3.202899	-1.442954
H	2.204829	-4.367587	0.617936
H	1.473950	-0.634267	2.659496
H	1.606714	-5.597993	2.678745
H	0.913930	-1.877173	4.727315
H	0.971518	-4.362608	4.738074
H	4.312688	-1.348147	-2.130370
H	4.360472	-1.262674	2.183236
H	6.759629	-0.984489	-2.158451
H	6.801236	-0.891989	2.144299
H	8.010887	-0.749554	-0.022709
H	-1.716288	4.152915	0.369415
H	1.998634	3.403356	2.434394
H	-2.702453	5.008002	2.469924
H	1.002090	4.252662	4.526228
H	-1.350222	5.059165	4.554472
H	3.225861	2.586170	-1.960712
H	1.758132	5.820805	0.490688
H	5.147003	3.986630	-2.650273
H	3.676778	7.211241	-0.207292
H	5.377365	6.300858	-1.771978
C	-5.343996	-1.874539	0.443153
C	-5.615024	0.248547	-1.561532
O	-4.704897	-2.287008	-2.229521
C	-5.232205	-3.251794	0.692847
C	-5.726233	-3.794148	1.879582
C	-6.337096	-2.966884	2.825845
C	-6.458901	-1.597061	2.580985
C	-5.968203	-1.051337	1.392836
H	-4.769757	-3.894738	-0.049596
H	-5.637819	-4.860661	2.063969
H	-6.722816	-3.389743	3.748826
H	-6.941659	-0.953540	3.310375
H	-6.082883	0.011826	1.205524
C	-6.701502	0.089646	-2.436288
C	-7.481011	1.189050	-2.797561
C	-7.181718	2.455881	-2.289432
C	-6.097945	2.623062	-1.423962
C	-5.313622	1.525374	-1.063178
H	-6.922941	-0.893389	-2.839952
H	-8.317961	1.057937	-3.476993
H	-7.787775	3.311661	-2.571900
H	-5.858055	3.608020	-1.034711
H	-4.464345	1.669461	-0.402186

Table S198. XYZ Coordinates of H_para_IV_P(O)Ph$_2$

111

scf done: -6451.940371

C	6.776726	-1.256006	-2.083537
C	6.734528	-1.055909	-0.702125
C	5.758868	-0.229894	-0.141048
C	4.815790	0.402663	-0.961840
C	4.859042	0.194085	-2.349897
C	5.838281	-0.628462	-2.907000
P	3.514147	1.507306	-0.294748
C	4.086910	1.990093	1.379717
C	4.871312	3.141664	1.547472
C	5.282296	3.528858	2.824183
C	4.917502	2.770059	3.939232
C	4.136630	1.622694	3.777119
C	3.714422	1.234986	2.503930
Pd	1.164973	0.871052	-0.350447
P	1.641672	-1.430179	-0.099171
C	0.384854	-2.499565	0.698047
C	0.318309	-2.535593	2.100137
C	-0.652419	-3.308456	2.737868
C	-1.568399	-4.045730	1.983409
C	-1.508017	-4.012421	0.588232
C	-0.535872	-3.243649	-0.053894
C	-0.842463	0.334851	-0.450086
C	-1.650287	0.241195	0.695997
C	-2.993624	-0.131977	0.608114
C	-3.569162	-0.430902	-0.636882
C	-2.771797	-0.338192	-1.785502
C	-1.431235	0.045981	-1.690729
P	0.331886	3.094149	-0.426066
C	-0.888569	3.543394	-1.712568
C	-0.423563	3.985348	-2.962579
C	-1.328714	4.285739	-3.981654
C	-2.700928	4.143814	-3.765110
C	-3.168416	3.698519	-2.526220
C	-2.268937	3.394167	-1.504264
O	1.576489	4.161508	-0.761464
C	-0.342604	3.696236	1.160144
C	-1.139311	4.851929	1.238545
C	-1.560971	5.327969	2.479873
C	-1.193064	4.658997	3.650329
C	-0.396828	3.513596	3.581369
C	0.028806	3.033923	2.342307
O	2.931726	-1.554335	0.882169
C	2.033152	-2.275732	-1.675813
C	2.597826	-3.563275	-1.676639
C	2.884804	-4.196111	-2.885940
C	2.605046	-3.559764	-4.099375

C	2.048029	-2.279911	-4.104332
C	1.769822	-1.636755	-2.896190
O	3.839692	2.819595	-1.235791
Br	4.015603	-4.323749	1.882988
P	-5.290791	-0.998998	-0.849501
H	-3.585243	-0.184589	1.518164
H	-1.238167	0.456819	1.677192
H	-0.851374	0.122290	-2.606184
H	-3.208639	-0.564310	-2.753633
H	3.222330	-2.481901	1.160107
H	3.093102	3.456331	-1.187195
H	2.830138	-4.062142	-0.738645
H	1.354199	-0.633849	-2.902754
H	3.326646	-5.188139	-2.881307
H	1.837116	-1.778534	-5.044122
H	2.826474	-4.059411	-5.037880
H	1.035115	-1.975333	2.691880
H	-0.495238	-3.228918	-1.138036
H	-0.690169	-3.336860	3.822851
H	-2.218636	-4.584216	-0.000716
H	-2.327702	-4.642952	2.478777
H	5.151582	3.732119	0.681270
H	3.114069	0.339578	2.378797
H	5.887240	4.422391	2.947146
H	3.852081	1.030491	4.641866
H	5.238687	3.073118	4.931414
H	-1.435675	5.378060	0.336947
H	0.651505	2.145654	2.291881
H	-2.177524	6.219929	2.532063
H	-0.107383	2.993537	4.489208
H	-1.526092	5.031064	4.614494
H	0.640581	4.106366	-3.137749
H	-2.642122	3.046259	-0.546998
H	-0.959690	4.633470	-4.941650
H	-4.234454	3.587479	-2.353556
H	-3.403946	4.379563	-4.558207
H	5.736976	-0.079066	0.932504
H	4.135194	0.679689	-2.996903
H	7.463146	-1.539417	-0.058351
H	5.866601	-0.779730	-3.981916
H	7.537777	-1.897934	-2.517247
O	-5.642885	-1.156348	-2.313655
C	-5.438110	-2.588697	0.045006
C	-6.362175	0.180549	0.044299
C	-7.638330	-0.226255	0.470382
C	-8.517184	0.691853	1.045180
C	-8.133675	2.026970	1.198170
C	-6.870430	2.442761	0.772044
C	-5.989989	1.525389	0.195896
H	-7.945064	-1.261864	0.358936

H	-9.498817	0.365233	1.375019
H	-8.817639	2.740413	1.648230
H	-6.568927	3.479348	0.888919
H	-5.009723	1.855741	-0.130985
C	-5.463421	-3.761285	-0.724628
C	-5.530473	-5.010689	-0.104647
C	-5.574964	-5.097679	1.289056
C	-5.555255	-3.933266	2.062445
C	-5.486473	-2.683523	1.444790
H	-5.440589	-3.684451	-1.807040
H	-5.554506	-5.913010	-0.708462
H	-5.631761	-6.068861	1.771796
H	-5.599826	-3.997536	3.145523
H	-5.488113	-1.787210	2.057601
H	1.323991	5.099042	-0.779654

Table S199. XYZ Coordinates of H_para_V_P(O)Ph$_2$

110

scf done: -6451.489131

C	6.703186	-1.599830	-1.970764
C	6.667135	-1.307413	-0.605889
C	5.712601	-0.421662	-0.100791
C	4.782733	0.181449	-0.957864
C	4.824045	-0.119257	-2.329292
C	5.779421	-1.002507	-2.832688
P	3.503437	1.375409	-0.386141
C	4.052122	1.883177	1.294754
C	4.745245	3.091390	1.461276
C	5.137730	3.508099	2.735195
C	4.842411	2.723016	3.852285
C	4.148963	1.519928	3.693354
C	3.748166	1.102228	2.422462
Pd	1.143226	0.787165	-0.447962
P	1.542106	-1.537070	-0.075120
C	0.253336	-2.547807	0.758500
C	0.181729	-2.527158	2.160801
C	-0.812375	-3.244177	2.827404
C	-1.750583	-3.982332	2.101506
C	-1.687029	-4.005812	0.706366
C	-0.690096	-3.293542	0.036921
C	-0.888211	0.362864	-0.509197
C	-1.650064	0.225878	0.666980
C	-3.010512	-0.085889	0.632241
C	-3.661387	-0.289245	-0.595122
C	-2.916108	-0.166576	-1.776266
C	-1.558224	0.161320	-1.728188
P	0.630930	3.082155	-0.843159
C	-0.966298	3.482805	-1.676602
C	-0.980618	3.493247	-3.080249
C	-2.153602	3.788135	-3.777228

C	-3.328289	4.079700	-3.079631
C	-3.320961	4.079609	-1.682546
C	-2.148374	3.780948	-0.984253
O	1.712965	3.796671	-1.725763
C	0.531963	4.002145	0.754541
C	1.161732	5.252142	0.848398
C	1.123292	5.974994	2.042636
C	0.456830	5.456180	3.155800
C	-0.166541	4.208195	3.073242
C	-0.123040	3.481132	1.881391
O	2.840864	-1.724838	0.895476
C	1.875920	-2.452887	-1.630518
C	2.438434	-3.740828	-1.616251
C	2.678792	-4.409950	-2.816627
C	2.354212	-3.809466	-4.037259
C	1.796738	-2.529859	-4.058163
C	1.565306	-1.851438	-2.859155
O	3.848038	2.640134	-1.335281
Br	3.820147	-4.544160	1.961320
P	-5.412193	-0.767507	-0.740332
H	-3.556346	-0.173287	1.568246
H	-1.177552	0.342148	1.638989
H	-1.024431	0.269863	-2.668703
H	-3.408746	-0.323382	-2.731575
H	3.076521	-2.658335	1.184531
H	2.958609	3.179080	-1.545498
H	2.702946	-4.214486	-0.673462
H	1.147753	-0.849018	-2.874322
H	3.119436	-5.402599	-2.799663
H	1.549656	-2.056157	-5.003718
H	2.540423	-4.336698	-4.968436
H	0.913614	-1.964521	2.731999
H	-0.647277	-3.324743	-1.047019
H	-0.852124	-3.227539	3.912714
H	-2.414310	-4.578221	0.138343
H	-2.528493	-4.535675	2.618680
H	4.967649	3.700729	0.591278
H	3.213601	0.165016	2.301706
H	5.673414	4.445617	2.854550
H	3.916983	0.906750	4.559568
H	5.148026	3.048240	4.842566
H	1.684759	5.645793	-0.017683
H	-0.600798	2.508255	1.828055
H	1.614783	6.941937	2.104747
H	-0.681300	3.797708	3.937362
H	0.428059	6.018444	4.084695
H	-0.065656	3.284554	-3.626027
H	-2.157836	3.793856	0.100761
H	-2.148384	3.794924	-4.863600
H	-4.227128	4.321367	-1.134239

H	-4.241127	4.312572	-3.620182
H	5.696874	-0.202063	0.961031
H	4.112502	0.342291	-3.007596
H	7.384320	-1.765794	0.068885
H	5.801590	-1.224029	-3.895787
H	7.446863	-2.288356	-2.361378
O	-5.846356	-0.873450	-2.187609
C	-5.601946	-2.369762	0.126130
C	-6.382984	0.439316	0.230647
C	-7.669735	0.094567	0.679203
C	-8.471371	1.038668	1.320737
C	-7.998710	2.338907	1.519191
C	-6.724376	2.693173	1.071209
C	-5.920759	1.749838	0.427803
H	-8.044716	-0.914073	0.533168
H	-9.461940	0.759612	1.667313
H	-8.622112	3.072755	2.021522
H	-6.354123	3.702692	1.223587
H	-4.932378	2.033772	0.081426
C	-5.690781	-3.524438	-0.665668
C	-5.790755	-4.783345	-0.069945
C	-5.805304	-4.898378	1.322433
C	-5.722187	-3.752164	2.118300
C	-5.620012	-2.493038	1.524396
H	-5.691265	-3.425876	-1.746607
H	-5.863695	-5.671098	-0.691272
H	-5.887839	-5.876769	1.786614
H	-5.743310	-3.837605	3.200682
H	-5.572188	-1.610285	2.154813

Table S200. XYZ Coordinates of H_para_TS2_P(O)Ph$_2$

110

scf done: -6451.447989

C	1.993433	2.723745	2.143781
C	1.677878	3.332556	0.916261
C	2.538990	4.310580	0.393141
C	3.691520	4.678168	1.089934
C	3.998124	4.073688	2.311814
C	3.147347	3.097249	2.836255
P	0.183300	2.909011	-0.083887
O	0.240858	3.487033	-1.515371
Pd	1.066461	0.643018	-0.281132
P	2.563394	1.084106	-2.078117
O	2.094328	2.390321	-2.954497
C	-0.994282	1.139819	0.195270
C	-1.786752	0.941768	-0.956119
C	-3.063766	0.387791	-0.860879
C	-3.595393	0.043257	0.390424
C	-2.821225	0.259538	1.542884
C	-1.546844	0.812802	1.450144

P	-5.261435	-0.676269	0.611703
O	-5.571354	-0.898527	2.076247
C	-5.292811	-2.245683	-0.326772
C	-5.317986	-2.306302	-1.729634
C	-5.299788	-3.541550	-2.379167
C	-5.259729	-4.724737	-1.634956
C	-5.240117	-4.671354	-0.239409
C	-5.255911	-3.437005	0.412738
C	-6.423961	0.450308	-0.233631
C	-6.165681	1.829053	-0.303100
C	-7.114933	2.697558	-0.844176
C	-8.331328	2.199955	-1.316941
C	-8.600571	0.830734	-1.244975
C	-7.653987	-0.039854	-0.704544
P	1.530133	-1.489766	0.753826
C	0.614592	-2.975358	0.158220
C	1.080694	-4.283172	0.378571
C	0.353959	-5.374995	-0.097564
C	-0.845912	-5.178051	-0.788880
C	-1.315025	-3.882781	-1.013199
C	-0.582551	-2.786156	-0.549449
C	-1.124831	3.927600	0.756466
C	-2.204816	4.365193	-0.027162
C	-3.222153	5.136839	0.535567
C	-3.176391	5.478627	1.890401
C	-2.103668	5.051830	2.674715
C	-1.081910	4.281967	2.111745
C	1.281765	-1.570748	2.581099
C	2.288035	-1.085850	3.434291
C	2.086434	-1.037973	4.814672
C	0.873223	-1.463052	5.363255
C	-0.135141	-1.941147	4.523142
C	0.066130	-1.994678	3.141893
O	3.127177	-1.857773	0.578798
C	4.352966	1.424063	-1.779260
C	5.060533	0.664499	-0.834007
C	6.417498	0.910685	-0.610265
C	7.075046	1.922401	-1.314882
C	6.371637	2.688334	-2.248528
C	5.017357	2.440543	-2.481772
C	2.635031	-0.221550	-3.380147
Br	4.621292	-4.268557	2.065073
H	-3.231056	-0.009956	2.511406
H	-0.978483	0.974773	2.360255
H	-1.406760	1.240246	-1.928647
H	-3.643811	0.248310	-1.768303
H	3.485185	-2.635209	1.095484
H	2.012059	-4.451342	0.914984
H	-0.940802	-1.778646	-0.739164
H	0.723147	-6.382492	0.073373

H	-2.244631	-3.723911	-1.551779
H	-1.410145	-6.031793	-1.153352
H	3.237550	-0.763653	3.018078
H	-0.723032	-2.377953	2.502358
H	2.879093	-0.673011	5.461802
H	-1.078264	-2.280411	4.942244
H	0.716946	-1.425634	6.437391
H	4.467927	3.037419	-3.202648
H	6.877293	3.479308	-2.795683
H	6.958899	0.316767	0.120997
H	8.128374	2.116731	-1.133773
H	2.305001	4.774514	-0.559787
H	1.350435	1.956524	2.563271
H	4.349281	5.437927	0.677741
H	3.380029	2.621988	3.784621
H	4.894967	4.361168	2.852377
H	-2.238405	4.110669	-1.081371
H	-0.250061	3.972800	2.735641
H	-4.047198	5.475506	-0.084801
H	-2.054825	5.321641	3.725775
H	-3.968575	6.078389	2.328941
H	-5.371627	-1.396954	-2.320441
H	-5.325229	-3.580330	-3.464020
H	-5.250120	-5.684628	-2.142832
H	-5.215812	-5.588639	0.341251
H	-5.249204	-3.386710	1.496839
H	-7.871656	-1.102418	-0.655615
H	-9.545379	0.440425	-1.611094
H	-9.067515	2.876750	-1.740434
H	-6.903080	3.761200	-0.897795
H	-5.223449	2.227647	0.059798
H	1.356619	2.859608	-2.443473
C	2.290833	0.066817	-4.708575
C	2.333734	-0.935642	-5.681485
C	2.721824	-2.232474	-5.338308
C	3.064373	-2.526332	-4.014906
C	3.017004	-1.529894	-3.038658
H	1.992374	1.075637	-4.973168
H	2.065271	-0.701057	-6.707924
H	2.754447	-3.010705	-6.095401
H	3.363669	-3.534170	-3.740990
H	3.271367	-1.773407	-2.010370
H	4.552837	-0.116658	-0.273552

Table S201. XYZ Coordinates of H_para_VI_P(O)Ph$_2$

110
scf done: -6451.482394

C	0.697338	-2.822648	-2.627925
P	-0.163985	-3.234403	-1.064881
Pd	0.896134	-0.282973	0.131843

P	2.316680	-1.030168	1.889363
C	1.628775	-1.982281	3.317771
C	1.699883	-1.469193	4.621964
C	1.160434	-2.185647	5.693638
C	0.546352	-3.420754	5.476115
C	0.468064	-3.934347	4.178459
C	0.997981	-3.219762	3.101981
C	0.015964	-2.467018	-3.803314
C	0.726007	-2.193616	-4.973708
C	2.120470	-2.287514	-4.985970
C	2.804074	-2.656308	-3.824898
C	2.096963	-2.921055	-2.649677
C	-1.652107	-4.186370	-1.558796
C	-1.562547	-5.587155	-1.561799
C	-2.656792	-6.363109	-1.946807
C	-3.850251	-5.747107	-2.332415
C	-3.949754	-4.353644	-2.326525
C	-2.857546	-3.574960	-1.937966
C	-0.801044	-1.686959	-0.328569
C	-0.956629	-0.473268	-1.096200
C	-1.965172	0.468267	-0.719178
C	-2.765465	0.270489	0.390514
C	-2.575928	-0.908792	1.185714
C	-1.649575	-1.853303	0.828684
O	0.713711	-4.060018	-0.143638
P	-3.937153	1.549490	0.931373
O	-3.362038	2.568501	1.894179
P	1.772301	1.875003	-0.270140
O	2.394567	2.437265	1.163657
C	0.665181	3.251412	-0.790455
C	-0.005089	4.022346	0.172436
C	-0.922972	4.999530	-0.218769
C	-1.192600	5.210529	-1.572916
C	-0.537273	4.440250	-2.537676
C	0.384812	3.466819	-2.150151
C	3.152271	1.991784	-1.483497
C	3.978643	3.125339	-1.579947
C	5.010284	3.161099	-2.518134
C	5.222639	2.076631	-3.376119
C	4.406299	0.948355	-3.286863
C	3.380359	0.903236	-2.338665
O	2.938301	0.267504	2.705005
C	3.859411	-1.976041	1.500544
C	3.787505	-3.233980	0.876918
C	4.957539	-3.930538	0.566687
C	6.208615	-3.379517	0.859967
C	6.284784	-2.125606	1.469229
C	5.117319	-1.426705	1.789225
C	-4.592259	2.355731	-0.576872
C	-4.760138	3.747492	-0.540299

C	-5.308586	4.421256	-1.633675
C	-5.692673	3.709611	-2.772076
C	-5.526662	2.322193	-2.817173
C	-4.980747	1.646714	-1.725084
C	-5.353306	0.664296	1.683531
C	-5.870291	1.169845	2.884644
C	-6.975335	0.564404	3.487035
C	-7.571151	-0.550102	2.893242
C	-7.059253	-1.061891	1.696915
C	-5.955727	-0.458349	1.093357
Br	3.578205	5.333425	1.622562
H	2.670420	-2.084690	-5.900193
H	0.189769	-1.919940	-5.877415
H	-1.068432	-2.417655	-3.816470
H	2.626028	-3.216980	-1.750296
H	3.886431	-2.745209	-3.833996
H	2.761235	3.373364	1.210009
H	2.765597	1.101800	2.209197
H	-0.638097	-6.062676	-1.249414
H	-2.952352	-2.493304	-1.923316
H	-2.579034	-7.446361	-1.942636
H	-4.878610	-3.872228	-2.617770
H	-4.702028	-6.351050	-2.631201
H	-0.572143	-0.414445	-2.109581
H	-1.552758	-2.758550	1.420937
H	-2.099329	1.344999	-1.345316
H	-3.193910	-1.072389	2.062580
H	2.179410	-0.511695	4.792298
H	0.909276	-3.620274	2.095041
H	1.223135	-1.777393	6.698705
H	-0.014150	-4.892052	4.001443
H	0.127286	-3.976789	6.309992
H	0.205582	3.872340	1.226277
H	0.896412	2.883573	-2.910780
H	-1.425707	5.596492	0.536599
H	-0.737392	4.602175	-3.593056
H	-1.908076	5.969487	-1.874988
H	3.828345	3.971849	-0.914024
H	2.756184	0.017055	-2.257740
H	5.649219	4.037371	-2.582276
H	4.567751	0.101173	-3.946838
H	6.024792	2.111890	-4.107760
H	2.821732	-3.664700	0.624582
H	5.176466	-0.455934	2.269721
H	4.891581	-4.904676	0.089486
H	7.252834	-1.689199	1.699849
H	7.116230	-3.922576	0.612217
H	-4.452562	4.293207	0.345796
H	-5.433635	5.499428	-1.596477
H	-6.118073	4.233065	-3.623317

H	-5.819847	1.766659	-3.703028
H	-4.847765	0.570553	-1.777983
H	-5.396042	2.032883	3.340949
H	-7.367910	0.961318	4.418610
H	-8.429948	-1.022119	3.361513
H	-7.517069	-1.932374	1.236505
H	-5.560729	-0.873944	0.171074

Table S202. XYZ Coordinates of H_para_VII_P(O)Ph$_2$

110

scf done: -6451.479640

C	0.922233	2.619217	-1.620089
C	1.519792	2.769681	-0.359389
C	0.791387	3.399712	0.664971
C	-0.497694	3.878212	0.430007
C	-1.082500	3.728824	-0.831242
C	-0.370230	3.097618	-1.853917
P	3.177704	2.060832	0.042749
O	3.904421	3.251971	0.913146
Pd	3.146146	0.053495	1.200477
P	3.253845	-1.752179	2.603540
O	4.242187	-1.489600	3.932030
C	4.027362	2.039317	-1.592170
C	4.385619	3.216081	-2.272335
C	5.036504	3.142767	-3.504643
C	5.330738	1.900221	-4.075357
C	4.979599	0.725973	-3.405881
C	4.337007	0.796845	-2.166386
C	3.956609	-3.337275	1.991871
C	4.736166	-3.315053	0.824719
C	5.328310	-4.485335	0.344665
C	5.145285	-5.689597	1.027563
C	4.369459	-5.722318	2.190501
C	3.775709	-4.554200	2.669336
C	1.689236	-2.281015	3.413258
C	1.569390	-2.358319	4.807986
C	0.353670	-2.728503	5.390606
C	-0.749085	-3.022269	4.586402
C	-0.634975	-2.943889	3.194576
C	0.574573	-2.570988	2.606965
Br	3.834154	6.334788	0.025871
C	-2.268713	-0.208818	-0.924941
C	-3.282561	0.415147	-0.199292
C	-4.247913	-0.350425	0.469859
C	-4.169145	-1.749816	0.419629
C	-3.149968	-2.374228	-0.298954
C	-2.194893	-1.609109	-0.986549
P	-5.618260	0.388294	1.438725
O	-6.059316	-0.514657	2.566516
P	-0.748721	-2.390062	-1.790159

C	-1.279391	-4.043799	-2.357997
C	-0.401273	-5.112313	-2.125048
C	-0.721297	-6.396006	-2.571261
C	-1.919623	-6.620638	-3.251852
C	-2.801210	-5.560863	-3.484433
C	-2.484443	-4.276740	-3.040881
C	-6.965675	0.727112	0.251985
C	-6.747521	1.033842	-1.100075
C	-7.829461	1.295869	-1.942881
C	-9.133830	1.246354	-1.445135
C	-9.358210	0.930593	-0.102612
C	-8.279604	0.670912	0.743463
C	-5.006236	2.004264	2.032222
C	-4.394633	2.036811	3.295995
C	-3.889332	3.234959	3.801144
C	-3.992246	4.409630	3.050561
C	-4.607417	4.386041	1.796345
C	-5.116516	3.188827	1.288224
O	0.462109	-2.474955	-0.882008
C	-0.400773	-1.390693	-3.281102
C	-1.392497	-1.031228	-4.207922
C	-1.056487	-0.291266	-5.341619
C	0.269375	0.096779	-5.558384
C	1.259433	-0.253219	-4.637962
C	0.925987	-0.995291	-3.502397
H	0.527416	0.674135	-6.441264
H	-1.828831	-0.014366	-6.052807
H	-2.428766	-1.312823	-4.047173
H	1.684320	-1.260350	-2.772943
H	2.288698	0.054258	-4.796357
H	3.828236	4.181219	0.552826
H	4.463276	-0.547438	3.960496
H	0.525253	-4.929062	-1.590596
H	-3.185843	-3.467315	-3.218521
H	-0.036733	-7.218151	-2.385384
H	-3.736549	-5.734305	-4.007855
H	-2.169320	-7.619190	-3.598160
H	-1.531648	0.403877	-1.434494
H	-3.104140	-3.458141	-0.323364
H	-3.303724	1.498985	-0.149061
H	-4.898567	-2.348496	0.955695
H	2.426578	-2.126397	5.431537
H	0.645657	-2.505492	1.522775
H	0.270270	-2.785359	6.472349
H	-1.492816	-3.162866	2.564951
H	-1.694342	-3.305787	5.040323
H	1.241147	3.527884	1.645377
H	1.463979	2.134632	-2.425942
H	-1.047402	4.365354	1.230183
H	-0.815001	2.982462	-2.838384

H	-2.084870	4.104928	-1.014485
H	4.167224	4.188597	-1.836215
H	4.075456	-0.114339	-1.632899
H	5.314877	4.056841	-4.021631
H	5.214504	-0.241815	-3.839727
H	5.837689	1.848887	-5.034701
H	4.873875	-2.375416	0.295248
H	3.167983	-4.590003	3.568567
H	5.927714	-4.456706	-0.560650
H	4.224447	-6.658659	2.721597
H	5.602393	-6.601484	0.654285
H	-4.327433	1.126029	3.882898
H	-3.419494	3.252934	4.779881
H	-3.601645	5.342639	3.445901
H	-4.699330	5.299582	1.216694
H	-5.606143	3.184430	0.319427
H	-8.450438	0.411516	1.783557
H	-10.371681	0.883042	0.284360
H	-9.973708	1.447143	-2.103649
H	-7.653130	1.531573	-2.988034
H	-5.739796	1.058948	-1.503208

Table S203. XYZ Coordinates of H_para_I_Me

66

scf done: -4731.392468

C	4.837637	-1.094561	-3.473607
C	4.032794	0.048990	-3.449732
C	3.331442	0.385620	-2.291809
C	3.438839	-0.409421	-1.138680
C	4.244104	-1.556840	-1.171011
C	4.938947	-1.896881	-2.335369
P	2.488295	0.066797	0.364882
O	2.787919	-1.220066	1.386319
Pd	0.275435	0.599878	0.023508
P	-1.946164	1.059597	-0.367048
C	-2.365251	2.690235	-1.101715
C	-3.675510	3.195785	-1.116579
C	-3.949748	4.421840	-1.722764
C	-2.918942	5.158931	-2.314267
C	-1.612284	4.666923	-2.300234
C	-1.336877	3.437925	-1.696370
O	-2.704605	0.063280	-1.475714
C	-3.073765	0.949961	1.083560
C	-4.305387	0.282983	1.017019
C	-5.129958	0.213801	2.143399
C	-4.733364	0.810240	3.342203
C	-3.503902	1.472218	3.417090
C	-2.675105	1.535171	2.296561
C	3.562865	1.328589	1.156147
C	4.964120	1.233765	1.148611

C	5.737726	2.195842	1.797945
C	5.121613	3.265474	2.455399
C	3.729340	3.370667	2.464446
C	2.953367	2.406045	1.817503
C	-1.450915	-3.062109	-2.061116
C	-2.815082	-3.330462	-1.863544
C	-3.231670	-3.716158	-0.580449
C	-2.326594	-3.828516	0.476128
C	-0.981948	-3.548660	0.243623
C	-0.526997	-3.166978	-1.015765
C	-3.807857	-3.180933	-2.991708
Br	0.266147	-3.714344	1.696211
H	-2.244905	-0.791890	-1.511121
H	2.020008	-1.816729	1.395802
H	0.523071	-2.962408	-1.188961
H	5.451212	0.412316	0.631743
H	1.868742	2.483784	1.818814
H	6.820750	2.114470	1.787941
H	3.247796	4.201794	2.971290
H	5.726920	4.015917	2.955538
H	4.320912	-2.180411	-0.286140
H	2.692366	1.265635	-2.284368
H	5.559089	-2.788646	-2.351171
H	3.945225	0.672476	-4.334821
H	5.377423	-1.360943	-4.377620
H	-4.480525	2.635536	-0.650614
H	-0.320756	3.051045	-1.679247
H	-4.966063	4.804855	-1.730260
H	-0.808568	5.238443	-2.755161
H	-3.134805	6.115749	-2.780741
H	-4.610236	-0.184804	0.086514
H	-1.711007	2.034082	2.364594
H	-6.081556	-0.306651	2.082987
H	-3.187153	1.930138	4.349675
H	-5.374934	0.754100	4.216731
H	-1.095409	-2.779339	-3.048532
H	-2.665561	-4.131358	1.460339
H	-4.279927	-3.937613	-0.400387
H	-3.347698	-3.398276	-3.959585
H	-4.664470	-3.847098	-2.858663
H	-4.193350	-2.155052	-3.035160

Table S204. XYZ Coordinates of H_para_TS1_Me

66

scf done: -4731.361452

C	3.134797	-1.135675	-2.164792
C	4.277187	-1.665836	-2.768715
C	5.438096	-0.895448	-2.863451
C	5.454758	0.405987	-2.353531
C	4.314499	0.936210	-1.748289

C	3.140109	0.170418	-1.647883
P	1.562074	0.828720	-0.950427
C	2.148734	2.075189	0.274464
C	1.620641	3.376215	0.267179
C	1.990625	4.297436	1.250347
C	2.884449	3.929447	2.257634
C	3.409596	2.633846	2.277690
C	3.044626	1.712342	1.295904
Pd	-0.180537	-0.626641	-0.259445
P	-2.281820	0.315133	-0.912102
O	-2.427869	0.418234	-2.572417
C	0.783438	-2.009349	1.055212
C	2.163658	-2.068967	1.330258
C	2.594280	-1.878707	2.635697
C	1.687369	-1.676392	3.700540
C	0.323390	-1.698249	3.411633
C	-0.146941	-1.914686	2.103889
C	2.194358	-1.476731	5.108615
Br	0.072354	-3.326041	-0.578749
O	1.001799	1.803489	-2.189604
C	-3.835246	-0.553239	-0.423900
C	-3.765755	-1.924103	-0.126795
C	-4.921199	-2.639587	0.194772
C	-6.158249	-1.992117	0.223542
C	-6.239044	-0.628100	-0.071657
C	-5.085535	0.088447	-0.392783
C	-2.611966	2.040643	-0.359402
C	-2.736866	2.309999	1.014696
C	-2.920022	3.616736	1.465361
C	-2.973078	4.674019	0.550978
C	-2.841883	4.416257	-0.814301
C	-2.659638	3.106526	-1.268693
H	3.662713	-1.882565	2.839974
H	2.877993	-2.217400	0.528351
H	-1.208217	-2.039278	1.919551
H	-0.400370	-1.595990	4.216634
H	-2.696528	1.497123	1.735750
H	-2.551320	2.915669	-2.331655
H	-3.021327	3.810649	2.529234
H	-2.880994	5.232514	-1.529901
H	-3.113426	5.691738	0.902978
H	3.462935	0.710981	1.323525
H	0.921648	3.670084	-0.509502
H	4.108291	2.341384	3.056307
H	1.579055	5.302420	1.226645
H	3.170998	4.645805	3.021920
H	-2.802758	-2.427596	-0.148027
H	-4.854341	-3.698874	0.424963
H	-7.056841	-2.546627	0.477816
H	-7.199550	-0.121647	-0.047764

H	-5.159715	1.149733	-0.611127
H	-3.315274	0.689904	-2.857673
H	4.343521	1.945587	-1.349089
H	6.356343	1.007443	-2.425237
H	6.328107	-1.307266	-3.330261
H	4.260814	-2.678674	-3.160508
H	2.234851	-1.739813	-2.083179
H	1.675757	2.421923	-2.515723
H	1.369362	-1.403516	5.822161
H	2.840610	-2.304209	5.423304
H	2.789585	-0.559318	5.189996

Table S205. XYZ Coordinates of H_para_IIa_Me

66

scf done: -4731.435641

C	-2.017043	4.784975	-1.601567
C	-1.232950	3.776034	-2.167206
C	-0.845759	2.677651	-1.398364
C	-1.231874	2.589665	-0.051729
C	-2.021541	3.604100	0.511703
C	-2.412448	4.696560	-0.264395
P	-0.714052	1.154769	0.955014
C	0.811766	1.666930	1.823963
C	1.427088	0.737124	2.679293
C	2.563005	1.093250	3.404887
C	3.097361	2.379711	3.279997
C	2.490983	3.307193	2.431095
C	1.351707	2.954833	1.703571
Pd	-0.734954	-0.811280	-0.226184
Br	-1.187890	-3.021237	-1.467326
C	-2.747306	-0.775881	0.209282
C	-3.261571	-1.421195	1.346700
C	-4.642634	-1.464247	1.590498
C	-5.553580	-0.864660	0.713714
C	-5.037285	-0.220091	-0.421080
C	-3.662638	-0.169566	-0.666890
C	-7.039986	-0.887988	0.985491
P	1.593812	-1.018901	-0.949355
O	1.624730	-1.873064	-2.363300
C	2.693449	-1.956203	0.179187
C	4.068178	-2.099559	-0.076617
C	4.861454	-2.881861	0.761890
C	4.292439	-3.530293	1.862632
C	2.926745	-3.399969	2.121046
C	2.130048	-2.618653	1.281075
C	2.582245	0.430194	-1.477875
C	2.469984	0.884811	-2.803141
C	3.167463	2.019236	-3.220370
C	3.978591	2.715449	-2.320731
C	4.089821	2.272779	-1.001071

C	3.394498	1.138244	-0.578300
O	-1.736353	1.166072	2.247818
H	4.519397	-1.597187	-0.926577
H	-2.476505	0.550221	2.082451
H	0.802932	-2.413915	-2.394066
H	-5.010080	-1.979168	2.475690
H	-2.591981	-1.919264	2.044872
H	-3.307440	0.345850	-1.555761
H	-5.720715	0.253836	-1.123100
H	1.065650	-2.526929	1.477864
H	5.922981	-2.985529	0.557852
H	2.480810	-3.905434	2.972370
H	4.913442	-4.136894	2.515163
H	1.854639	0.338073	-3.509589
H	3.496625	0.803856	0.448715
H	3.080756	2.354773	-4.249601
H	4.719339	2.807762	-0.296578
H	4.522739	3.596740	-2.647167
H	-2.332422	3.532154	1.548699
H	-0.245958	1.890864	-1.846156
H	-3.026194	5.476945	0.175354
H	-0.929248	3.839217	-3.207720
H	-2.323499	5.635537	-2.203088
H	1.018908	-0.263934	2.778756
H	0.887023	3.684233	1.049410
H	3.030804	0.368430	4.064085
H	2.901804	4.307447	2.333476
H	3.983379	2.656361	3.843509
H	-7.609789	-1.097249	0.074189
H	-7.297766	-1.647202	1.729285
H	-7.390242	0.079071	1.367384

Table S206. XYZ Coordinates of H_para_IIb_Me

66

scf done: -4731.436947

C	5.771158	-1.805362	0.891428
C	5.320085	-3.041869	1.357652
C	3.956277	-3.248530	1.585032
C	3.045650	-2.221510	1.341156
C	3.492200	-0.972485	0.876546
C	4.863109	-0.771365	0.653310
P	2.254853	0.364646	0.683156
O	2.163993	1.009982	2.203527
Pd	0.154024	-0.259967	-0.178015
P	-2.001978	-0.825734	-0.959898
O	-1.908033	-1.660537	-2.375618
C	-0.725674	1.031380	1.124352
C	-0.758223	2.399785	0.816439
C	-1.357171	3.317491	1.685117
C	-1.946343	2.904226	2.888124

C	-1.916099	1.536095	3.189007
C	-1.314076	0.612603	2.325374
C	-2.568880	3.902142	3.837738
Br	1.241212	-1.814750	-1.982712
C	-3.165653	0.509030	-1.414551
C	-3.344349	0.842317	-2.766132
C	-4.192501	1.892310	-3.124866
C	-4.866177	2.617969	-2.140570
C	-4.689058	2.292645	-0.792876
C	-3.840689	1.247226	-0.427697
C	-2.940371	-1.938306	0.151662
C	-2.224017	-2.745543	1.050792
C	-2.891301	-3.666143	1.860090
C	-4.280495	-3.787692	1.779131
C	-5.000724	-2.991263	0.884808
C	-4.336134	-2.071154	0.073005
C	3.083529	1.663890	-0.309118
C	3.406989	2.905457	0.257165
C	4.001837	3.899708	-0.523814
C	4.273587	3.663876	-1.872390
C	3.945412	2.430630	-2.444165
C	3.348149	1.435381	-1.671206
H	-1.367604	4.372726	1.418702
H	-0.315953	2.764261	-0.107536
H	-1.321516	-0.438863	2.598030
H	-2.371587	1.180980	4.111553
H	3.033223	1.082828	2.629197
H	-0.958569	-1.874389	-2.533108
H	3.191179	3.099110	1.302593
H	3.083597	0.483587	-2.122956
H	4.250359	4.857197	-0.075895
H	4.151371	2.244566	-3.494034
H	4.735287	4.438157	-2.477808
H	1.984472	-2.389541	1.501228
H	5.223659	0.186469	0.292879
H	3.602169	-4.210507	1.943089
H	6.829770	-1.642956	0.712543
H	6.028509	-3.844277	1.540827
H	-2.831008	0.271247	-3.531808
H	-3.706925	1.008703	0.622457
H	-4.328356	2.138183	-4.173913
H	-5.210980	2.853655	-0.023430
H	-5.526889	3.432974	-2.421019
H	-1.143201	-2.651335	1.115380
H	-4.904760	-1.456272	-0.616952
H	-2.328295	-4.283459	2.553642
H	-6.080454	-3.085907	0.818798
H	-4.801105	-4.500773	2.411436
H	-3.360627	3.443281	4.437747
H	-1.825339	4.305639	4.536984

H	-2.999046	4.752550	3.299604

Table S207. XYZ Coordinates of H_para_III_Me

66

scf done: -4731.407301

C	2.758219	4.567109	1.041293
C	2.610378	3.660735	2.093848
C	2.354600	2.313762	1.830393
C	2.246206	1.863040	0.505130
C	2.388293	2.782170	-0.550045
C	2.649385	4.125404	-0.280502
P	1.794660	0.131833	0.102075
O	2.649499	-0.272677	-1.209492
Pd	-0.510172	0.172751	-0.294611
P	-2.842484	0.341121	-0.682237
C	-3.906699	-0.547260	0.506794
C	-4.816125	-1.526870	0.085845
C	-5.584167	-2.218860	1.025734
C	-5.445106	-1.942059	2.386669
C	-4.531658	-0.972564	2.812821
C	-3.760775	-0.281096	1.879366
C	-0.486806	-1.733740	-0.896502
C	-0.116582	-2.044647	-2.209055
C	-0.141397	-3.375162	-2.639660
C	-0.523236	-4.415895	-1.780908
C	-0.888910	-4.081619	-0.470924
C	-0.873286	-2.754348	-0.023782
C	-0.515282	-5.853110	-2.248482
C	2.375324	-0.882701	1.504855
C	3.697216	-1.352105	1.571942
C	4.107348	-2.114509	2.666571
C	3.211355	-2.408185	3.698359
C	1.895699	-1.943663	3.635197
C	1.474997	-1.190945	2.537181
O	-3.270744	-0.234105	-2.167332
C	-3.403021	2.082569	-0.644450
C	-2.569568	3.052637	-1.232320
C	-2.956693	4.392265	-1.257055
C	-4.174234	4.777376	-0.689866
C	-5.006816	3.821116	-0.103067
C	-4.626416	2.478749	-0.079107
Br	5.720800	0.268589	-1.358336
H	0.144115	-3.602269	-3.664530
H	0.191212	-1.265842	-2.899526
H	-1.170254	-2.533295	0.996648
H	-1.195523	-4.865722	0.217877
H	-4.168791	0.021520	-2.433688
H	3.641007	-0.066197	-1.198865
H	4.404015	-1.117531	0.779762
H	0.446147	-0.844855	2.480094

H	5.129549	-2.478104	2.714388
H	1.195164	-2.175072	4.431875
H	3.536951	-3.001668	4.547566
H	2.312730	2.442733	-1.578630
H	2.253817	1.615641	2.655357
H	2.769755	4.825704	-1.101562
H	2.701847	3.999059	3.121589
H	2.960397	5.613455	1.249457
H	-4.922489	-1.755140	-0.969593
H	-3.050239	0.467788	2.220065
H	-6.288446	-2.974586	0.691550
H	-4.417988	-0.757250	3.870881
H	-6.042295	-2.482206	3.115019
H	-1.621366	2.760894	-1.676216
H	-5.279707	1.744037	0.379280
H	-2.307600	5.133127	-1.713505
H	-5.953918	4.119218	0.336098
H	-4.473079	5.821165	-0.703894
H	-1.188766	-6.471823	-1.648409
H	-0.820135	-5.934633	-3.296449
H	0.487692	-6.290949	-2.169405

Table S208. XYZ Coordinates of H_para_IV_Me

91

scf done: -5611.945939

C	0.594470	-5.606597	-1.681695
C	0.986171	-5.073133	-2.912249
C	1.268546	-3.709191	-3.022788
C	1.155028	-2.875984	-1.907940
C	0.770365	-3.410039	-0.667176
C	0.487676	-4.780675	-0.561347
P	0.586504	-2.321184	0.798832
C	2.221161	-2.340202	1.630169
C	3.398657	-2.710711	0.967242
C	4.617363	-2.709720	1.648383
C	4.670545	-2.340344	2.994084
C	3.499844	-1.968776	3.660408
C	2.280877	-1.964175	2.982038
Pd	-0.552017	-0.222782	0.301497
P	1.449244	0.959434	-0.112688
O	2.516882	-0.079696	-0.768467
C	-1.539970	1.566801	-0.090226
C	-2.024436	1.875485	-1.369689
C	-2.698620	3.076565	-1.617074
C	-2.900843	4.020017	-0.600519
C	-2.414582	3.711616	0.676760
C	-1.750580	2.506611	0.929519
C	-3.594388	5.333567	-0.879261
P	-2.756001	-1.086666	0.479417
O	-2.725117	-2.601151	1.194626

C	-3.567999	-1.393520	-1.129040
C	-4.959586	-1.553362	-1.241920
C	-5.530079	-1.875209	-2.473701
C	-4.720651	-2.040873	-3.600350
C	-3.335964	-1.888723	-3.495551
C	-2.760541	-1.567269	-2.266059
C	-3.991846	-0.223256	1.517994
C	-4.158375	-0.621835	2.854613
C	-5.065534	0.046331	3.679054
C	-5.809645	1.116814	3.179526
C	-5.641485	1.522710	1.853161
C	-4.733776	0.862678	1.024876
O	-0.224895	-3.248560	1.892885
C	2.199278	1.666944	1.401398
C	3.474166	2.259241	1.364402
C	4.032473	2.783992	2.529780
C	3.326794	2.730562	3.735945
C	2.063517	2.138516	3.780341
C	1.504656	1.601954	2.618293
C	1.416508	2.348636	-1.309373
C	1.490546	2.057012	-2.680963
C	1.430463	3.083903	-3.623387
C	1.291369	4.410356	-3.206987
C	1.216174	4.706706	-1.844205
C	1.278373	3.682097	-0.897876
Br	5.335425	0.880471	-1.779424
H	-3.071546	3.280807	-2.618934
H	-1.889327	1.178512	-2.191839
H	-1.406429	2.308534	1.941001
H	-2.560447	4.419371	1.490554
H	3.404783	0.297589	-1.067572
H	-1.193639	-3.141843	1.767937
H	4.037036	2.291672	0.434879
H	0.529224	1.125869	2.658012
H	5.019603	3.235471	2.496686
H	1.516177	2.086885	4.716755
H	3.764514	3.144805	4.639454
H	1.613725	1.030603	-3.011486
H	1.222526	3.925423	0.157733
H	1.497504	2.847914	-4.681323
H	1.114832	5.736556	-1.514786
H	1.245796	5.209323	-3.941144
H	0.179470	-5.193278	0.393766
H	1.382782	-1.818414	-1.993561
H	0.371973	-6.665917	-1.592894
H	1.573890	-3.291566	-3.977714
H	1.068663	-5.717608	-3.782467
H	-5.598595	-1.423493	-0.374882
H	-1.683488	-1.450273	-2.189122
H	-6.606193	-1.994890	-2.552465

H	-2.704305	-2.017886	-4.368963
H	-5.168464	-2.288142	-4.558119
H	-3.590145	-1.457485	3.249637
H	-4.604360	1.195703	0.000712
H	-5.191056	-0.274141	4.708770
H	-6.216063	2.356125	1.460745
H	-6.517119	1.633849	3.820776
H	3.368604	-3.002241	-0.076697
H	1.375586	-1.678301	3.508492
H	5.523865	-3.001633	1.126629
H	3.534480	-1.681892	4.707195
H	5.619676	-2.342292	3.521873
H	-3.582990	-3.053883	1.240884
H	-4.094000	5.720395	0.014057
H	-2.879134	6.098472	-1.207511
H	-4.342402	5.231175	-1.671493

Table S209. XYZ Coordinates of H_para_V_Me

90
scf done: -5611.492460

C	1.672567	1.717792	2.551329
C	2.406424	1.465094	1.382572
C	3.800900	1.636152	1.393275
C	4.442313	2.058957	2.558161
C	3.703406	2.323180	3.715577
C	2.317709	2.153626	3.711248
P	1.517951	0.923402	-0.131419
Pd	-0.608135	-0.048599	0.334364
P	-2.784410	-0.927265	0.743608
C	-4.163747	0.231781	1.147596
C	-4.453793	0.454448	2.502159
C	-5.483894	1.320635	2.875116
C	-6.238579	1.973206	1.897587
C	-5.960214	1.751734	0.546078
C	-4.929466	0.887184	0.172801
C	1.635847	2.386700	-1.238922
C	1.678366	2.185122	-2.627588
C	1.727304	3.274721	-3.498056
C	1.728587	4.577035	-2.992145
C	1.685404	4.785040	-1.611467
C	1.640177	3.696627	-0.738218
O	2.457765	-0.180379	-0.882193
P	0.323817	-2.231329	0.898541
C	1.988483	-2.368032	1.675283
C	3.129685	-2.785232	0.977061
C	4.364017	-2.865799	1.625419
C	4.472677	-2.529499	2.976420
C	3.339866	-2.111295	3.679749
C	2.106160	-2.029006	3.033286
C	-1.480633	1.789227	-0.083358

C	-1.746352	2.209083	-1.396636
C	-2.315307	3.458628	-1.668379
C	-2.627855	4.354160	-0.637347
C	-2.348660	3.949600	0.675148
C	-1.798805	2.691537	0.945294
C	-3.264414	5.694728	-0.926440
C	0.391013	-3.390411	-0.529845
C	-0.099289	-4.696916	-0.385082
C	-0.080954	-5.579802	-1.466751
C	0.426067	-5.166483	-2.701379
C	0.910783	-3.864659	-2.853520
C	0.888163	-2.975594	-1.776380
O	-0.569064	-2.996851	2.011240
O	-2.802540	-1.965345	1.920186
C	-3.398704	-1.798063	-0.765989
C	-4.020663	-3.046264	-0.615456
C	-4.490547	-3.741371	-1.731792
C	-4.341994	-3.197614	-3.010487
C	-3.715076	-1.958919	-3.170234
C	-3.238862	-1.265790	-2.054827
Br	5.348363	0.442093	-2.012926
H	-2.508281	3.744066	-2.701307
H	-1.488755	1.569149	-2.237658
H	-1.624814	2.419342	1.984107
H	-2.571084	4.623514	1.501088
H	3.361223	0.114420	-1.209197
H	-1.546639	-2.586609	2.024755
H	4.386570	1.433476	0.499242
H	0.596896	1.567930	2.552978
H	5.521290	2.184358	2.562114
H	1.739844	2.350579	4.609531
H	4.207860	2.655491	4.618279
H	1.689939	1.176217	-3.027659
H	1.614644	3.871450	0.332595
H	1.768314	3.105724	-4.570205
H	1.693375	5.794932	-1.211731
H	1.767302	5.424880	-3.669830
H	-0.499027	-5.011219	0.573643
H	1.268156	-1.965671	-1.896627
H	-0.463398	-6.589390	-1.345482
H	1.303720	-3.538671	-3.812440
H	0.439535	-5.854026	-3.542187
H	-4.124418	-3.466829	0.380101
H	-2.742090	-0.309799	-2.187175
H	-4.970502	-4.707734	-1.604020
H	-3.590933	-1.534931	-4.162763
H	-4.706752	-3.738989	-3.878673
H	-3.880650	-0.068298	3.261234
H	-4.728984	0.723171	-0.880769
H	-5.699598	1.480550	3.927944

H	-6.549352	2.248720	-0.219638
H	-7.041945	2.645115	2.185935
H	3.059146	-3.052926	-0.071256
H	1.228985	-1.712820	3.589364
H	5.239615	-3.195222	1.073493
H	3.415455	-1.850769	4.731571
H	5.433405	-2.594427	3.478988
H	-2.964477	6.448043	-0.190926
H	-2.990300	6.062728	-1.920134
H	-4.360089	5.633758	-0.895557

Table S210. XYZ Coordinates of H_para_TS2_Me

90

scf done: -5611.454766

C	-1.405217	-2.671090	2.013934
C	-2.177319	-2.284699	0.907421
C	-3.475164	-2.805431	0.762309
C	-3.981390	-3.698782	1.707010
C	-3.200803	-4.088861	2.800314
C	-1.911869	-3.575617	2.951883
P	-1.452784	-1.088285	-0.295445
O	-2.695874	-0.094479	-0.728470
Pd	0.471961	0.219920	0.385947
P	2.812564	0.749072	0.153644
C	4.468986	-0.007417	-0.232116
C	5.390019	-0.124125	0.820840
C	6.652109	-0.677652	0.602214
C	7.010738	-1.127368	-0.671620
C	6.102200	-1.012217	-1.725310
C	4.839631	-0.453674	-1.508375
P	-0.207038	2.342337	1.210911
C	-0.929942	3.634109	0.107499
C	-0.527627	4.973927	0.213260
C	-1.078732	5.943407	-0.627300
C	-2.037331	5.584959	-1.578871
C	-2.438887	4.251263	-1.691210
C	-1.884379	3.276346	-0.858229
C	-1.433243	2.285039	2.590177
C	-1.113285	2.799097	3.855075
C	-2.038664	2.729413	4.900173
C	-3.291321	2.148324	4.692376
C	-3.615593	1.632446	3.433866
C	-2.692271	1.694185	2.388826
O	1.039773	3.139706	1.918474
C	2.079567	-1.188249	0.784868
C	2.259999	-1.308395	2.175125
C	2.532849	-2.550990	2.757248
C	2.661725	-3.703861	1.974415
C	2.509542	-3.575040	0.581376
C	2.238683	-2.343606	-0.007081

C	2.984271	-5.042618	2.592802
C	-1.216346	-2.129853	-1.801491
C	-1.357141	-1.545538	-3.071862
C	-1.082967	-2.280816	-4.226415
C	-0.654211	-3.607458	-4.129797
C	-0.506189	-4.195714	-2.871378
C	-0.784078	-3.463240	-1.715021
O	3.131037	1.772191	1.270644
C	2.436437	1.577936	-1.460433
C	2.748848	2.942360	-1.567758
C	2.484257	3.634901	-2.751050
C	1.904409	2.975294	-3.837758
C	1.587644	1.618052	-3.736913
C	1.847508	0.920820	-2.554832
Br	-5.381408	-1.061226	-2.187896
H	2.601193	-4.457006	-0.048805
H	2.130177	-2.289295	-1.085741
H	2.196832	-0.426330	2.806187
H	2.656890	-2.618545	3.835511
H	-3.486694	-0.508170	-1.178168
H	1.895792	2.635652	1.706336
H	-4.094714	-2.508895	-0.081412
H	-0.407404	-2.261696	2.141752
H	-4.987008	-4.093168	1.589811
H	-1.302419	-3.870387	3.801459
H	-3.599173	-4.786370	3.531661
H	-1.701865	-0.519664	-3.156492
H	-0.672304	-3.936282	-0.744225
H	-1.209425	-1.818463	-5.201373
H	-0.180737	-5.228881	-2.787952
H	-0.441478	-4.179303	-5.028332
H	0.220607	5.248725	0.949696
H	-2.192420	2.238780	-0.960609
H	-0.759334	6.978283	-0.539112
H	-3.179131	3.966840	-2.434025
H	-2.464659	6.339861	-2.232791
H	3.193240	3.456549	-0.721266
H	1.583421	-0.130183	-2.491170
H	2.732668	4.689939	-2.823579
H	1.134938	1.099062	-4.576788
H	1.699638	3.515309	-4.757424
H	5.117751	0.232379	1.808784
H	4.154986	-0.359046	-2.344691
H	7.356300	-0.755353	1.425899
H	6.375559	-1.350301	-2.720900
H	7.992802	-1.558983	-0.842079
H	-2.950453	1.274836	1.419985
H	-0.140518	3.252323	4.013324
H	-4.587252	1.175958	3.266724
H	-1.779583	3.131441	5.875869

H	-4.009692	2.094216	5.505360
H	2.313030	-5.824389	2.221329
H	4.006086	-5.356203	2.346774
H	2.900126	-5.010238	3.682301

Table S211. XYZ Coordinates of H_para_VI_Me

90

scf done: -5611.487763

C	-2.193936	-0.064978	2.952129
C	-2.716733	-1.008397	2.054419
C	-2.825506	-2.349639	2.455964
C	-2.400425	-2.741843	3.726635
C	-1.876264	-1.797127	4.613263
C	-1.777358	-0.458440	4.226041
P	-3.283565	-0.412652	0.416634
C	-4.797488	-1.380640	0.042070
C	-6.038383	-0.819239	0.381694
C	-7.220872	-1.517919	0.133918
C	-7.174975	-2.783987	-0.455697
C	-5.944676	-3.347461	-0.802802
C	-4.760274	-2.648507	-0.559420
C	-2.061939	-0.924558	-0.836642
C	-1.105518	-1.970884	-0.606709
C	-0.597253	-2.715245	-1.718940
C	-0.948509	-2.419707	-3.018870
C	-1.869973	-1.350071	-3.246745
C	-2.416914	-0.643351	-2.204614
C	-0.407299	-3.204229	-4.188921
Pd	-0.017835	0.009385	-0.311716
P	0.090422	2.365944	-0.527088
O	1.629847	2.833507	-0.921148
O	-3.600266	1.071477	0.435452
P	2.228007	-0.589880	0.065558
C	2.920956	-2.093441	-0.744117
C	3.360416	-2.031979	-2.076660
C	3.763188	-3.189905	-2.745041
C	3.722056	-4.426882	-2.096758
C	3.280558	-4.499026	-0.772520
C	2.882130	-3.341754	-0.100994
C	2.795023	-0.775953	1.808885
C	4.154806	-0.827579	2.163169
C	4.525079	-0.966638	3.500933
C	3.548028	-1.065424	4.497471
C	2.196316	-1.014212	4.154242
C	1.822231	-0.862456	2.816008
O	3.177101	0.620970	-0.570840
C	-0.243092	3.461828	0.928490
C	-1.510662	3.471376	1.536953
C	-1.749536	4.281747	2.648988
C	-0.729473	5.079933	3.175639

C	0.534080	5.065037	2.581888
C	0.777084	4.261794	1.463936
C	-0.844010	3.232686	-1.868514
C	-0.175239	3.977874	-2.851576
C	-0.893402	4.608803	-3.871032
C	-2.285230	4.504946	-3.919279
C	-2.956687	3.759699	-2.945632
C	-2.243869	3.119305	-1.929779
Br	6.345737	0.552143	-0.768912
H	-1.556486	-2.101964	5.605427
H	-2.490042	-3.781421	4.027484
H	-3.255323	-3.089440	1.787884
H	-2.126132	0.975956	2.655364
H	-1.381729	0.281073	4.916022
H	4.175087	0.509388	-0.580042
H	2.249236	2.073284	-0.823061
H	-6.071442	0.170056	0.827332
H	-3.810810	-3.090007	-0.847304
H	-8.176333	-1.073652	0.397282
H	-5.906099	-4.327508	-1.269159
H	-8.095235	-3.327364	-0.649340
H	-1.005018	-2.406679	0.382331
H	-3.142415	0.139474	-2.406877
H	0.085486	-3.536370	-1.517147
H	-2.159959	-1.110808	-4.267160
H	0.905342	4.061676	-2.812839
H	-2.775658	2.523313	-1.191674
H	-0.363352	5.183551	-4.625779
H	-4.039161	3.669542	-2.981282
H	-2.842452	4.996027	-4.712085
H	3.403761	-1.074100	-2.585760
H	2.549610	-3.411891	0.930866
H	4.111689	-3.124175	-3.771891
H	3.252954	-5.455805	-0.258653
H	4.034834	-5.327109	-2.617636
H	4.923574	-0.742649	1.398771
H	0.769880	-0.805116	2.550127
H	5.577744	-0.999584	3.767634
H	1.432161	-1.084918	4.922690
H	3.842070	-1.177464	5.537212
H	-2.306694	2.838667	1.151780
H	1.756471	4.256608	0.997378
H	-2.734192	4.285360	3.108927
H	1.333227	5.681141	2.985225
H	-0.918026	5.704941	4.043933
H	-1.214739	-3.682317	-4.756902
H	0.127678	-2.552094	-4.889887
H	0.284966	-3.984469	-3.861417

Table S212. XYZ Coordinates of H_para_VII_Me

90
scf done: -5611.488616

C	-3.488455	-1.064725	2.245498
C	-3.065684	-1.071625	0.906168
C	-2.431462	-2.217994	0.401127
C	-2.229264	-3.333667	1.217017
C	-2.656728	-3.318777	2.547620
C	-3.288805	-2.181900	3.058410
P	-3.227706	0.466076	-0.104135
O	-4.640996	1.125008	0.414476
Pd	-1.449025	1.938243	0.089594
P	0.156305	3.530414	0.455032
O	-0.457242	5.020181	0.917522
C	1.358180	3.193077	1.805453
C	1.597352	4.115470	2.833737
C	2.502211	3.811090	3.855099
C	3.173496	2.586785	3.856461
C	2.935159	1.661322	2.835130
C	2.027792	1.957065	1.816615
C	1.236871	4.051277	-0.938886
C	0.796966	3.801287	-2.248502
C	1.557990	4.214895	-3.343881
C	2.768945	4.879979	-3.139815
C	3.217260	5.131097	-1.839481
C	2.457753	4.717632	-0.744380
C	-3.559528	-0.192228	-1.792975
C	-4.755981	-0.851255	-2.124355
C	-4.956072	-1.329020	-3.420019
C	-3.965937	-1.165020	-4.394361
C	-2.774626	-0.512125	-4.073082
C	-2.576869	-0.020426	-2.779928
Br	-7.342246	-0.563582	0.794704
O	1.802233	-0.452559	-0.772423
P	2.773454	-1.600505	-0.576362
C	4.485963	-1.118410	-0.999223
C	4.820906	0.240897	-0.896692
C	6.113376	0.671102	-1.200259
C	7.079730	-0.251262	-1.609132
C	6.751673	-1.605101	-1.718515
C	5.459241	-2.038931	-1.418175
C	2.355969	-3.036514	-1.627081
C	1.703806	-2.788429	-2.845004
C	1.373579	-3.845619	-3.693600
C	1.693032	-5.157577	-3.333330
C	2.338099	-5.412268	-2.120653
C	2.665922	-4.356990	-1.266833
C	2.796322	-2.216840	1.140551
C	1.559523	-2.443630	1.770765
C	1.509584	-2.899524	3.084514

C	2.686003	-3.139574	3.813887
C	3.914098	-2.911932	3.180147
C	3.974249	-2.454591	1.861523
C	2.621152	-3.610847	5.246233
H	1.434675	-5.980210	-3.993620
H	2.579540	-6.431738	-1.835078
H	3.152380	-4.565412	-0.318702
H	1.447288	-1.769064	-3.115830
H	0.865282	-3.646361	-4.632290
H	-5.426012	0.511208	0.496761
H	-1.394504	4.907468	1.132321
H	4.065310	0.956069	-0.587607
H	5.211227	-3.090986	-1.519704
H	6.364040	1.724719	-1.120946
H	7.499052	-2.322740	-2.043396
H	8.084875	0.084239	-1.847109
H	0.632522	-2.264170	1.233959
H	4.942110	-2.286878	1.400255
H	0.542786	-3.072588	3.550388
H	4.837874	-3.092997	3.723267
H	1.072517	5.065272	2.832890
H	1.839759	1.225718	1.032644
H	2.680667	4.531367	4.648619
H	3.449515	0.704363	2.835471
H	3.874575	2.351340	4.652086
H	-3.990320	-0.189637	2.646896
H	-2.102617	-2.247281	-0.633526
H	-3.630691	-2.165238	4.089375
H	-1.745240	-4.216815	0.809888
H	-2.504600	-4.189111	3.179317
H	-5.535051	-0.982382	-1.376348
H	-1.657519	0.503918	-2.528511
H	-5.885834	-1.832226	-3.670512
H	-2.004887	-0.376590	-4.827451
H	-4.126341	-1.540934	-5.400862
H	-0.144180	3.279260	-2.402398
H	2.817080	4.910803	0.261898
H	1.208204	4.014724	-4.352478
H	4.159905	5.646356	-1.678910
H	3.364407	5.198935	-3.990394
H	3.583936	-4.004500	5.582388
H	2.346848	-2.786996	5.916183
H	1.865667	-4.392584	5.373703

Table S213. XYZ Coordinates of H_para_I_OMe

67

scf done: -4806.596849

C	5.000383	-0.360459	-3.958228
C	4.869459	0.776578	-3.154429
C	4.175526	0.703739	-1.947021

C	3.605536	-0.509852	-1.525351
C	3.738427	-1.645053	-2.337299
C	4.435814	-1.568839	-3.546447
P	2.614368	-0.552650	0.021632
O	2.588300	-2.200993	0.281272
Pd	0.550214	0.466770	-0.048515
P	-1.520184	1.463140	-0.181658
C	-1.620300	3.116283	-0.975980
C	-2.756546	3.934359	-0.863830
C	-2.805233	5.164374	-1.519815
C	-1.718561	5.593357	-2.288718
C	-0.583270	4.788604	-2.403235
C	-0.534746	3.555026	-1.749755
O	-2.652811	0.636702	-1.089746
C	-2.425375	1.715183	1.401110
C	-3.717984	1.210779	1.599140
C	-4.366810	1.397642	2.823462
C	-3.732296	2.089195	3.856814
C	-2.440973	2.592203	3.666898
C	-1.788743	2.401411	2.449021
C	3.812300	0.043327	1.281919
C	5.152146	-0.380566	1.297287
C	6.017917	0.060116	2.297345
C	5.557801	0.933284	3.289239
C	4.229201	1.361325	3.281763
C	3.359331	0.915248	2.283265
C	-2.340544	-2.620284	-1.965172
C	-3.705915	-2.769039	-1.658402
C	-4.087964	-3.102408	-0.351131
C	-3.113880	-3.288503	0.634249
C	-1.769316	-3.139950	0.313198
C	-1.369933	-2.807628	-0.983037
O	-4.566894	-2.573004	-2.689163
Br	-0.444921	-3.401769	1.680279
C	-5.967523	-2.698336	-2.438798
H	-2.362253	-0.281293	-1.218250
H	1.721483	-2.459411	0.636731
H	-0.320609	-2.697086	-1.229762
H	5.518749	-1.051348	0.525637
H	2.322039	1.242052	2.272355
H	7.051792	-0.272712	2.302899
H	3.869593	2.039611	4.050011
H	6.235882	1.278518	4.064330
H	3.305278	-2.586211	-2.016191
H	4.084406	1.592759	-1.328028
H	4.537779	-2.456433	-4.164366
H	5.309187	1.719177	-3.467253
H	5.540227	-0.302952	-4.898833
H	-3.600705	3.612873	-0.261005
H	0.347313	2.924352	-1.832845

H	-3.687985	5.790554	-1.428568
H	0.263519	5.120166	-2.997030
H	-1.757223	6.554140	-2.793797
H	-4.209753	0.672933	0.795289
H	-0.780425	2.785787	2.313198
H	-5.368575	1.002752	2.967147
H	-1.940930	3.127163	4.469109
H	-4.237469	2.232496	4.807507
H	-2.055157	-2.375291	-2.983203
H	-3.414527	-3.547369	1.643193
H	-5.130586	-3.223852	-0.085330
H	-6.458848	-2.498568	-3.391013
H	-6.223171	-3.709312	-2.102369
H	-6.305715	-1.968701	-1.694533

Table S214. XYZ Coordinates of H_para_TS1_OMe

67

scf done: -4806.565865

C	3.089801	-0.760888	-2.489657
C	4.240437	-1.200062	-3.148082
C	5.397480	-0.418021	-3.128093
C	5.401325	0.804003	-2.449693
C	4.252028	1.243878	-1.791046
C	3.081972	0.465605	-1.804382
P	1.492116	1.013566	-1.040562
C	2.055324	2.141657	0.304358
C	1.553236	3.449303	0.395518
C	1.910427	4.273058	1.466378
C	2.766082	3.799598	2.462464
C	3.264840	2.495564	2.384350
C	2.911407	1.671196	1.316088
Pd	-0.210998	-0.546272	-0.486208
P	-2.343856	0.441084	-0.924908
O	-2.551656	0.791978	-2.545045
C	0.801555	-2.063615	0.632509
C	2.192979	-2.142404	0.848803
C	2.675798	-2.128221	2.145887
C	1.793719	-2.086612	3.249323
C	0.415577	-2.092125	3.033010
C	-0.084852	-2.131965	1.718400
O	2.392409	-2.077303	4.474799
C	1.557340	-2.046645	5.629387
Br	0.044743	-3.173460	-1.137923
O	0.908760	2.093585	-2.178258
C	-3.874604	-0.506165	-0.517777
C	-3.785178	-1.904068	-0.417794
C	-4.924499	-2.671317	-0.165042
C	-6.165030	-2.049228	-0.008911
C	-6.265699	-0.658386	-0.107783
C	-5.128350	0.109656	-0.359707

C	-2.661978	2.061890	-0.108711
C	-2.771693	2.119446	1.291604
C	-2.938108	3.343527	1.938359
C	-2.990492	4.528373	1.196662
C	-2.875424	4.480864	-0.193380
C	-2.708966	3.254610	-0.844185
H	3.744621	-2.143279	2.336837
H	2.879334	-2.170216	0.010378
H	-1.151200	-2.240901	1.555471
H	-0.284943	-2.115626	3.859139
H	-2.733462	1.205697	1.879646
H	-2.613258	3.227170	-1.924954
H	-3.027851	3.373423	3.020382
H	-2.914886	5.396973	-0.775701
H	-3.118361	5.481470	1.701253
H	3.306517	0.660794	1.269527
H	0.882398	3.824031	-0.370919
H	3.932575	2.120608	3.154724
H	1.518402	5.284723	1.519523
H	3.042733	4.440213	3.294635
H	-2.819229	-2.387591	-0.537818
H	-4.842320	-3.751502	-0.087470
H	-7.050903	-2.644601	0.192024
H	-7.228895	-0.171879	0.015527
H	-5.217219	1.190058	-0.424737
H	-3.449088	1.097840	-2.754699
H	4.269714	2.192179	-1.262579
H	6.299777	1.414105	-2.432020
H	6.294295	-0.759843	-3.636526
H	4.234056	-2.151965	-3.671016
H	2.193236	-1.375631	-2.496657
H	1.578013	2.737641	-2.461818
H	2.230270	-2.021501	6.487043
H	0.921651	-1.153297	5.639851
H	0.925188	-2.940376	5.690749

Table S215. XYZ Coordinates of H_para_IIa_OMe

67

scf done: -4806.640204

C	-1.787895	4.816943	-1.589422
C	-1.010442	3.803766	-2.156717
C	-0.634845	2.699115	-1.391131
C	-1.026103	2.608915	-0.046127
C	-1.808777	3.627707	0.519061
C	-2.188063	4.726555	-0.253812
P	-0.523236	1.165667	0.956182
C	1.008343	1.659082	1.825867
C	1.614166	0.720469	2.678336
C	2.754874	1.062150	3.403378
C	3.303510	2.342794	3.280783

C	2.706515	3.279039	2.434831
C	1.562494	2.941185	1.707900
Pd	-0.567095	-0.796540	-0.227230
Br	-1.045061	-3.005817	-1.462196
C	-2.580608	-0.731166	0.209113
C	-3.108293	-1.403700	1.329333
C	-4.482789	-1.437722	1.575842
C	-5.373359	-0.797133	0.702602
C	-4.869460	-0.119479	-0.414529
C	-3.486977	-0.088969	-0.644388
O	-6.698453	-0.887581	1.027180
C	-7.644452	-0.258875	0.167779
P	1.756256	-1.032407	-0.951797
O	1.776044	-1.887242	-2.365481
C	2.847372	-1.981022	0.175380
C	4.220545	-2.137455	-0.081072
C	5.006398	-2.928357	0.756359
C	4.431392	-3.572467	1.856520
C	3.067087	-3.429194	2.115485
C	2.277809	-2.639191	1.276652
C	2.758585	0.406297	-1.482469
C	2.648403	0.861690	-2.807647
C	3.356193	1.989148	-3.226413
C	4.175714	2.677581	-2.328397
C	4.285038	2.234104	-1.008846
C	3.579395	1.106532	-0.584527
O	-1.543519	1.184353	2.250901
H	4.676364	-1.638595	-0.930638
H	-2.296418	0.587573	2.076537
H	0.949022	-2.420314	-2.393977
H	-4.882433	-1.962952	2.438503
H	-2.446980	-1.926823	2.016457
H	-3.125969	0.452035	-1.515395
H	-5.532172	0.389256	-1.105336
H	1.214349	-2.537250	1.473598
H	6.066809	-3.042128	0.551900
H	2.616512	-3.931304	2.966347
H	5.046647	-4.185829	2.508174
H	2.026385	0.320877	-3.512807
H	3.680145	0.771361	0.442369
H	3.270883	2.325308	-4.255556
H	4.921087	2.763035	-0.305650
H	4.727844	3.553453	-2.656009
H	-2.123198	3.554293	1.554886
H	-0.039765	1.909353	-1.839977
H	-2.796465	5.510371	0.187260
H	-0.702800	3.868689	-3.195965
H	-2.085334	5.672463	-2.188433
H	1.195014	-0.276217	2.775870
H	1.105244	3.677282	1.055998

H	3.215295	0.330575	4.060302
H	3.128425	4.274845	2.339047
H	4.193305	2.608163	3.843773
H	-8.624871	-0.456864	0.602772
H	-7.484334	0.824792	0.116108
H	-7.607571	-0.676190	-0.845629

Table S216. XYZ Coordinates of H_para_IIb_OMe

67

scf done: -4806.641639

C	-5.975335	-1.189724	-1.222121
C	-5.639684	-2.366929	-1.894009
C	-4.298422	-2.671898	-2.143682
C	-3.295217	-1.803050	-1.716770
C	-3.624992	-0.614096	-1.043932
C	-4.973866	-0.312880	-0.799615
P	-2.264673	0.533333	-0.609522
O	-2.081358	1.428392	-1.988841
Pd	-0.254383	-0.423332	0.154858
P	1.823160	-1.269573	0.890810
O	1.615070	-2.327543	2.134971
C	0.769430	1.008825	-0.868041
C	0.890599	2.293727	-0.326843
C	1.581933	3.313872	-0.995840
C	2.174731	3.050872	-2.235929
C	2.063079	1.767815	-2.791327
C	1.367922	0.764347	-2.115202
O	2.875507	3.965322	-2.976224
C	3.017596	5.281335	-2.452914
Br	-1.516862	-2.194440	1.608320
C	3.049620	-0.119990	1.609439
C	3.178827	-0.027290	3.003832
C	4.076436	0.882190	3.567492
C	4.848972	1.706214	2.746845
C	4.721687	1.620667	1.357589
C	3.824614	0.716550	0.788547
C	2.737424	-2.234846	-0.368991
C	2.016121	-2.805022	-1.430835
C	2.659367	-3.614505	-2.368351
C	4.029423	-3.860988	-2.253697
C	4.754313	-3.300837	-1.198340
C	4.113633	-2.492278	-0.258738
C	-2.979231	1.711370	0.598852
C	-3.153198	3.063147	0.268645
C	-3.657486	3.957905	1.216022
C	-3.986658	3.511815	2.496936
C	-3.807004	2.166570	2.834170
C	-3.300621	1.269167	1.894523
H	1.648185	4.295218	-0.539904
H	0.440249	2.527456	0.634560

H	1.304763	-0.218244	-2.573423
H	2.527069	1.572953	-3.754175
H	-2.932895	1.663595	-2.391093
H	0.648483	-2.502504	2.218282
H	-2.891648	3.418544	-0.722485
H	-3.150466	0.227965	2.165596
H	-3.790708	5.002391	0.950507
H	-4.058040	1.816376	3.830982
H	-4.377779	4.208914	3.231969
H	-2.252619	-2.049924	-1.895437
H	-5.244706	0.600242	-0.279742
H	-4.034365	-3.588784	-2.661907
H	-7.016380	-0.951517	-1.025821
H	-6.420700	-3.047352	-2.220085
H	2.588124	-0.675629	3.641533
H	3.731078	0.663840	-0.291310
H	4.173316	0.941693	4.647521
H	5.320571	2.258854	0.714740
H	5.547988	2.411534	3.186528
H	0.950264	-2.613578	-1.521829
H	4.686034	-2.060283	0.555649
H	2.092809	-4.048049	-3.186952
H	5.819105	-3.492907	-1.106354
H	4.531684	-4.487399	-2.984913
H	3.602282	5.837249	-3.187139
H	2.044872	5.770695	-2.320845
H	3.550067	5.279232	-1.494000

Table S217. XYZ Coordinates of H_para_III_OMe

67

scf done: -4806.611725

C	-2.471544	-4.882421	0.829221
C	-2.152411	-4.063818	1.915854
C	-1.975850	-2.691757	1.732215
C	-2.121109	-2.126458	0.454224
C	-2.434903	-2.955758	-0.635841
C	-2.614687	-4.326260	-0.444731
P	-1.771102	-0.349895	0.160887
O	-2.650367	0.087676	-1.122063
Pd	0.527921	-0.233542	-0.240591
P	2.869551	-0.237894	-0.608079
C	3.874554	0.317596	0.813185
C	4.685240	1.458085	0.730027
C	5.398834	1.893123	1.849744
C	5.301995	1.199451	3.056941
C	4.486541	0.066824	3.148364
C	3.771440	-0.371239	2.034710
C	0.403078	1.725058	-0.631817
C	-0.091996	2.161056	-1.861279
C	-0.135152	3.528755	-2.168605

C	0.313641	4.468192	-1.232722
C	0.802593	4.028109	0.006561
C	0.848380	2.666591	0.305760
O	0.315796	5.821021	-1.427943
C	-0.163394	6.320183	-2.673062
C	-2.403102	0.533882	1.629106
C	-3.757366	0.888034	1.744045
C	-4.206205	1.548028	2.888665
C	-3.316979	1.852300	3.923447
C	-1.969776	1.501758	3.812923
C	-1.510834	0.852891	2.664827
O	3.321441	0.731933	-1.862807
C	3.483373	-1.907977	-1.026743
C	2.658660	-2.724221	-1.822348
C	3.086680	-3.994661	-2.207645
C	4.338054	-4.462268	-1.798378
C	5.163361	-3.657648	-1.008141
C	4.741411	-2.385020	-0.622209
Br	-5.702174	-0.518452	-1.337352
H	-0.523125	3.837895	-3.132127
H	-0.458225	1.450751	-2.595394
H	1.239710	2.352976	1.268341
H	1.147318	4.765192	0.725670
H	4.238768	0.586638	-2.146348
H	-3.636810	-0.143851	-1.123604
H	-4.458372	0.646557	0.948552
H	-0.458617	0.597697	2.571315
H	-5.253228	1.823751	2.973107
H	-1.274676	1.742214	4.611689
H	-3.673007	2.365797	4.811681
H	-2.557298	-2.526242	-1.624820
H	-1.740849	-2.062745	2.585579
H	-2.869869	-4.958002	-1.290225
H	-2.047802	-4.491204	2.908553
H	-2.611719	-5.949199	0.975374
H	4.757773	2.007343	-0.202837
H	3.140974	-1.252858	2.116556
H	6.027935	2.774941	1.775820
H	4.407468	-0.474516	4.086168
H	5.856567	1.540295	3.925938
H	1.684236	-2.363170	-2.141890
H	5.388042	-1.768185	-0.006977
H	2.443860	-4.618383	-2.821056
H	6.136377	-4.020651	-0.691565
H	4.669498	-5.453273	-2.093530
H	-0.069346	7.405418	-2.618820
H	0.434534	5.946845	-3.513138
H	-1.215289	6.056072	-2.834926

Table S218. XYZ Coordinates of H_para_IV_OMe

92

scf done: -5687.150901

C	0.862869	3.688496	-0.973274
C	1.185476	2.377343	-1.351616
C	1.310636	2.066797	-2.715238
C	1.119300	3.053732	-3.682646
C	0.798417	4.358344	-3.299206
C	0.671492	4.673082	-1.944376
P	1.395420	1.033710	-0.121201
C	2.040676	1.869185	1.375882
C	3.222432	2.629578	1.323860
C	3.702285	3.250968	2.476568
C	3.009338	3.127332	3.685019
C	1.839436	2.368322	3.744702
C	1.360707	1.735557	2.595337
Pd	-0.439526	-0.386270	0.305535
P	-2.515638	-1.521632	0.454702
C	-3.865376	-0.829316	1.479697
C	-3.996488	-1.254640	2.812009
C	-4.991833	-0.713051	3.627537
C	-5.860289	0.256882	3.123313
C	-5.728934	0.689267	1.801129
C	-4.734170	0.155610	0.981750
P	0.941715	-2.316691	0.840209
O	0.238011	-3.316165	1.945858
C	1.277008	-3.401822	-0.601124
C	1.607243	-2.846742	-1.848256
C	1.838629	-3.679463	-2.945035
C	1.728159	-5.065786	-2.810125
C	1.390770	-5.621391	-1.573272
C	1.167091	-4.794894	-0.470698
C	2.555715	-2.116274	1.687552
C	3.777983	-2.355256	1.045364
C	4.978661	-2.189803	1.738327
C	4.969241	-1.785617	3.075033
C	3.753635	-1.544603	3.720590
C	2.551952	-1.704295	3.030224
C	-1.645796	1.265659	-0.091415
C	-2.156607	1.524962	-1.374991
C	-2.971670	2.628527	-1.624625
C	-3.289862	3.521371	-0.590469
C	-2.786701	3.287392	0.693954
C	-1.979773	2.164820	0.928053
O	-4.088953	4.576774	-0.935537
C	-4.443539	5.509026	0.080615
O	2.593408	0.128063	-0.750398
O	-2.304857	-3.028064	1.157718
C	-3.255983	-1.915532	-1.168520
C	-4.613204	-2.251777	-1.307344

C	-5.115623	-2.634676	-2.551333
C	-4.271711	-2.686668	-3.663610
C	-2.919924	-2.359377	-3.532601
C	-2.412642	-1.976162	-2.290947
Br	5.258563	1.430700	-1.797450
H	-3.367290	2.817625	-2.618582
H	-1.926164	0.860843	-2.202791
H	-1.619280	2.006965	1.940759
H	-3.014026	3.956798	1.515790
H	3.423369	0.612228	-1.061629
H	-0.732382	-3.347939	1.795404
H	3.776533	2.718184	0.392839
H	0.459882	1.131285	2.646557
H	4.618118	3.832860	2.431956
H	1.303458	2.262056	4.683082
H	3.385062	3.617310	4.578486
H	1.575412	1.059471	-3.020210
H	0.763942	3.945889	0.075856
H	1.226997	2.804496	-4.734173
H	0.428096	5.686892	-1.640509
H	0.651440	5.126578	-4.052608
H	0.901268	-5.225364	0.489281
H	1.701284	-1.770680	-1.952608
H	1.301765	-6.698445	-1.465716
H	2.101278	-3.244524	-3.904901
H	1.901923	-5.710800	-3.666395
H	-5.278858	-2.211488	-0.451518
H	-1.361088	-1.722865	-2.191129
H	-6.165885	-2.890735	-2.650931
H	-2.261612	-2.400264	-4.394843
H	-4.667173	-2.982060	-4.630751
H	-3.331143	-2.013600	3.210208
H	-4.636165	0.508480	-0.039279
H	-5.088416	-1.053634	4.653916
H	-6.400647	1.444624	1.404904
H	-6.636026	0.675130	3.757634
H	3.796869	-2.672453	0.008663
H	1.611943	-1.518234	3.540388
H	5.920581	-2.380972	1.232961
H	3.739706	-1.231404	4.760287
H	5.904704	-1.659491	3.612064
H	-3.095879	-3.591395	1.171733
H	-5.074397	6.256549	-0.402124
H	-5.007035	5.028533	0.889621
H	-3.559119	6.001178	0.502895

Table S219. XYZ Coordinates of H_para_V_OMe

91

scf done: -5686.697246

C	-1.466810	-2.136357	-2.689282

C	-1.380408	-3.176350	-3.615396
C	-1.219741	-4.493510	-3.177674
C	-1.149835	-4.766256	-1.809675
C	-1.239880	-3.727641	-0.880648
C	-1.399985	-2.403099	-1.312324
P	-1.467984	-0.991834	-0.135460
O	-2.592555	-0.015152	-0.806803
Pd	0.510305	0.251700	0.328605
P	-0.686044	2.295631	0.898728
C	-0.901784	3.421999	-0.540277
C	-0.521779	4.767764	-0.429266
C	-0.644094	5.626878	-1.523540
C	-1.146314	5.150448	-2.736956
C	-1.522379	3.809555	-2.855380
C	-1.395299	2.944661	-1.766150
C	1.600156	-1.462598	-0.116260
C	1.913466	-1.825279	-1.439329
C	2.631579	-2.983051	-1.739067
C	3.046477	-3.841292	-0.711361
C	2.737445	-3.516938	0.613518
C	2.032309	-2.335429	0.890795
O	3.738164	-4.959918	-1.103492
C	4.179727	-5.857570	-0.092819
P	2.561238	1.382619	0.758961
O	2.444328	2.414388	1.935640
C	3.094717	2.321943	-0.740798
C	3.605531	3.618176	-0.576813
C	4.013823	4.362667	-1.685397
C	3.914315	3.820881	-2.969840
C	3.398464	2.533863	-3.142896
C	2.983687	1.790890	-2.034955
C	4.061356	0.390236	1.178684
C	4.369726	0.209769	2.535475
C	5.490764	-0.531146	2.916666
C	6.318875	-1.098422	1.945493
C	6.022208	-0.917764	0.591711
C	4.900744	-0.179039	0.210215
O	0.105654	3.179517	2.000696
C	-2.350174	2.215880	1.684036
C	-3.532223	2.589339	1.031036
C	-4.759771	2.510631	1.692863
C	-4.820218	2.057839	3.012365
C	-3.646096	1.682683	3.671039
C	-2.419708	1.759131	3.010602
C	-2.210347	-1.726240	1.375401
C	-3.532490	-2.202687	1.386711
C	-4.063841	-2.757575	2.551397
C	-3.284245	-2.851024	3.708723
C	-1.971435	-2.376390	3.704827
C	-1.439530	-1.808904	2.544459

Br	-5.383845	-1.072920	-1.879648
H	2.869152	-3.244959	-2.766721
H	1.579077	-1.207789	-2.269540
H	1.834421	-2.105348	1.935116
H	3.042629	-4.158076	1.433206
H	-3.448703	-0.438274	-1.118765
H	1.125053	2.887148	2.023719
H	-4.150194	-2.128915	0.494367
H	-0.424286	-1.422957	2.544091
H	-5.088138	-3.119078	2.555784
H	-1.364568	-2.438875	4.603390
H	-3.702047	-3.287507	4.611352
H	-1.603764	-1.117115	-3.036938
H	-1.191619	-3.953726	0.179626
H	-1.442330	-2.957946	-4.677577
H	-1.031341	-5.788760	-1.462971
H	-1.153154	-5.302945	-3.898774
H	-0.126148	5.131723	0.513345
H	-1.690447	1.904061	-1.860671
H	-0.346416	6.667313	-1.428156
H	-1.912040	3.434739	-3.797654
H	-1.240707	5.819386	-3.587514
H	3.672364	4.036606	0.422869
H	2.573362	0.796280	-2.176945
H	4.407659	5.365842	-1.547311
H	3.312677	2.110443	-4.139716
H	4.231207	4.400769	-3.831928
H	3.738105	0.667965	3.289885
H	4.686165	-0.045231	-0.844967
H	5.719434	-0.659522	3.971086
H	6.666919	-1.348765	-0.169196
H	7.192701	-1.672456	2.240344
H	-3.499697	2.945654	0.007325
H	-1.511987	1.469935	3.531845
H	-5.668058	2.807111	1.176194
H	-3.684398	1.330683	4.697909
H	-5.775801	1.998508	3.525291
H	4.700677	-6.664887	-0.609931
H	4.871794	-5.371534	0.606134
H	3.338210	-6.276426	0.472993

Table S220. XYZ Coordinates of H_para_TS2_OMe

91

scf done: -5686.660280

C	-2.965029	1.129217	2.514217
C	-1.827478	1.926546	2.726256
C	-1.565870	2.416353	4.014032
C	-2.430014	2.118922	5.071470
C	-3.563055	1.332345	4.853551
C	-3.828393	0.839743	3.572140

P	-0.675624	2.286834	1.328439
C	-1.660020	3.478350	0.318948
C	-1.512237	4.861918	0.497938
C	-2.261687	5.755610	-0.269990
C	-3.166655	5.276759	-1.221151
C	-3.315189	3.899695	-1.406222
C	-2.561606	3.001723	-0.646236
Pd	0.364065	0.375819	0.372536
P	-1.318128	-1.220652	-0.355309
O	-2.746445	-0.459025	-0.671394
P	2.556616	1.337893	0.146685
C	4.316383	0.921193	-0.297703
C	5.261314	0.900648	0.740421
C	6.598716	0.601036	0.478294
C	7.011636	0.313136	-0.825572
C	6.079533	0.334011	-1.864598
C	4.740598	0.637936	-1.603567
C	2.010300	2.184114	-1.409321
C	1.533664	1.496415	-2.538886
C	1.131009	2.202467	-3.674736
C	1.192038	3.598427	-3.693757
C	1.658306	4.287658	-2.571378
C	2.064462	3.586664	-1.433985
O	2.711753	2.330782	1.325245
C	2.199516	-0.746615	0.645000
C	2.454241	-0.928373	2.021648
C	2.975636	-2.124268	2.506346
C	3.283093	-3.169031	1.620823
C	3.067139	-2.995748	0.243613
C	2.543111	-1.792624	-0.229376
O	3.795562	-4.296105	2.185724
C	4.138427	-5.388326	1.334284
C	-0.945412	-2.076789	-1.948348
C	-1.241520	-1.433314	-3.162129
C	-0.877690	-2.011014	-4.379750
C	-0.203314	-3.234896	-4.403650
C	0.100442	-3.879795	-3.202254
C	-0.266785	-3.305798	-1.982996
C	-1.747557	-2.626212	0.759908
C	-0.867289	-2.945532	1.805417
C	-1.150538	-4.004424	2.673303
C	-2.324067	-4.742668	2.513576
C	-3.212813	-4.422353	1.481968
C	-2.928079	-3.374088	0.606330
O	0.422208	3.264119	2.055399
Br	-5.273938	-1.807212	-2.110989
H	3.297786	-3.786414	-0.460634
H	2.386964	-1.686791	-1.298308
H	2.249348	-0.120759	2.718725
H	3.163694	-2.264815	3.566433

H	-3.464886	-0.983149	-1.127179
H	1.351612	2.931603	1.811718
H	-3.629738	-3.134868	-0.189835
H	0.040021	-2.364325	1.941051
H	-4.129537	-4.992409	1.358656
H	-0.458470	-4.244531	3.475496
H	-2.549452	-5.561409	3.191052
H	-1.774989	-0.487892	-3.153362
H	-0.031623	-3.822596	-1.057612
H	-1.125638	-1.507055	-5.309718
H	0.617659	-4.835158	-3.212515
H	0.079208	-3.684208	-5.351246
H	-0.805354	5.231106	1.233995
H	-2.673986	1.932031	-0.804479
H	-2.138855	6.825548	-0.125472
H	-4.012960	3.522813	-2.148998
H	-3.748571	5.972995	-1.818425
H	2.419908	4.123039	-0.559697
H	1.468326	0.412741	-2.538186
H	1.707251	5.372849	-2.580135
H	0.767492	1.659776	-4.542526
H	0.876967	4.145247	-4.577538
H	4.946133	1.132765	1.752326
H	4.036274	0.663630	-2.428410
H	7.318838	0.595418	1.291825
H	6.392636	0.120764	-2.882826
H	8.052579	0.079849	-1.030104
H	-3.173587	0.726888	1.526460
H	-0.686824	3.029838	4.180503
H	-4.706589	0.224542	3.396718
H	-2.217268	2.504447	6.064871
H	-4.233817	1.101431	5.676166
H	4.523791	-6.169293	1.990356
H	3.261959	-5.766769	0.795913
H	4.913340	-5.105513	0.612613

Table S221. XYZ Coordinates of H_para_VI_OMe

91

scf done: -5686.691250

C	-2.113844	0.498371	3.081122
C	-2.637811	-0.573491	2.342728
C	-2.718813	-1.842743	2.938365
C	-2.263071	-2.038788	4.243196
C	-1.737280	-0.967256	4.970602
C	-1.667218	0.301463	4.389947
P	-3.253917	-0.228985	0.652323
C	-4.799158	-1.206900	0.493847
C	-6.011475	-0.580629	0.823895
C	-7.214475	-1.282887	0.740259
C	-7.218137	-2.617703	0.326499

C	-6.017059	-3.247007	-0.009089
C	-4.812132	-2.545122	0.070244
C	-2.095018	-0.963880	-0.547554
C	-1.147823	-1.984512	-0.197328
C	-0.681549	-2.885828	-1.193356
C	-1.070379	-2.750668	-2.514676
C	-1.983783	-1.723116	-2.885121
C	-2.487256	-0.875336	-1.923688
O	-0.551792	-3.654478	-3.399178
C	-0.970017	-3.598564	-4.760562
Pd	-0.002427	0.031232	-0.233216
P	0.131200	2.295941	-0.889834
O	1.665056	2.637719	-1.417713
O	-3.538411	1.247326	0.446582
P	2.226002	-0.553168	0.228215
C	2.886143	-2.185159	-0.319674
C	3.363154	-2.351745	-1.629847
C	3.740821	-3.614243	-2.092324
C	3.637268	-4.729601	-1.257877
C	3.157004	-4.575085	0.045900
C	2.782785	-3.313463	0.511255
C	2.829749	-0.448233	1.967387
C	4.196400	-0.465188	2.298040
C	4.595649	-0.383983	3.632198
C	3.640800	-0.294690	4.650922
C	2.282565	-0.275474	4.331253
C	1.879832	-0.344342	2.994394
O	3.184884	0.513786	-0.619716
C	-0.122145	3.640913	0.358617
C	-1.352060	3.761155	1.029161
C	-1.534914	4.761173	1.986784
C	-0.494160	5.641458	2.298207
C	0.732984	5.518342	1.643453
C	0.919044	4.524725	0.677976
C	-0.832822	2.946324	-2.328866
C	-0.189272	3.491500	-3.450220
C	-0.935215	3.957108	-4.536539
C	-2.329791	3.885905	-4.514930
C	-2.976695	3.339224	-3.402654
C	-2.236219	2.864053	-2.318335
Br	6.354661	0.363300	-0.847036
H	-1.394000	-1.119151	5.989723
H	-2.330483	-3.023915	4.695045
H	-3.151372	-2.677561	2.395523
H	-2.069376	1.484873	2.632457
H	-1.270752	1.139196	4.956019
H	4.179816	0.383704	-0.622360
H	2.273721	1.904357	-1.167361
H	-6.007973	0.460041	1.132741
H	-3.886553	-3.040804	-0.206795

H	-8.147534	-0.788430	0.994233
H	-6.017279	-4.281737	-0.338905
H	-8.154540	-3.163982	0.260603
H	-1.005477	-2.261488	0.841590
H	-3.210604	-0.119786	-2.215898
H	-0.003443	-3.687873	-0.921124
H	-2.305089	-1.611055	-3.913455
H	0.893547	3.550689	-3.466308
H	-2.749784	2.420107	-1.468623
H	-0.424548	4.378084	-5.398493
H	-4.061407	3.275089	-3.382120
H	-2.908426	4.248436	-5.359881
H	3.457192	-1.489547	-2.282430
H	2.418398	-3.207346	1.529451
H	4.118659	-3.724589	-3.104929
H	3.079682	-5.436212	0.703757
H	3.930425	-5.711098	-1.618846
H	4.948850	-0.523963	1.515070
H	0.822524	-0.312013	2.744835
H	5.653669	-0.391777	3.879042
H	1.535376	-0.200826	5.115837
H	3.957162	-0.235885	5.688502
H	-2.162182	3.069010	0.811375
H	1.870262	4.433249	0.164104
H	-2.491125	4.849008	2.495809
H	1.547728	6.197250	1.880558
H	-0.638261	6.414166	3.048071
H	-0.449430	-4.412656	-5.266323
H	-2.051828	-3.747767	-4.857675
H	-0.691865	-2.648101	-5.230686

Table S222. XYZ Coordinates of H_para_VII_OMe

91

scf done: -5686.695529

C	0.382222	2.659952	2.413526
C	1.453760	2.946228	1.553467
C	1.707222	4.278088	1.189742
C	0.904846	5.307108	1.687538
C	-0.158844	5.014252	2.544972
C	-0.421531	3.689794	2.905261
P	2.478704	1.546307	0.977237
O	1.710695	0.237556	1.011259
C	3.072185	2.004358	-0.678837
C	2.130112	2.358908	-1.667125
C	2.529253	2.622063	-2.968035
C	3.888112	2.534037	-3.322542
C	4.836562	2.179167	-2.353514
C	4.421036	1.916843	-1.045811
O	4.176752	2.812885	-4.618630
C	5.537140	2.736831	-5.046845

C	3.942410	1.480575	2.073501
C	4.346127	0.222689	2.544127
C	5.453388	0.109092	3.387547
C	6.162878	1.250471	3.766438
C	5.763473	2.508051	3.303860
C	4.657089	2.624895	2.462230
C	3.314542	-1.950401	-2.303049
C	3.622786	-2.800127	-3.370272
C	2.845310	-3.936837	-3.599939
C	1.763623	-4.228557	-2.763876
C	1.453214	-3.382494	-1.689596
C	2.232780	-2.233728	-1.467868
P	0.021155	-3.707972	-0.581825
C	0.809841	-4.458328	0.900283
C	0.201167	-4.258266	2.149327
C	0.733831	-4.843190	3.300257
C	1.882171	-5.633016	3.212687
C	2.496870	-5.836903	1.973372
C	1.966372	-5.251406	0.823428
Pd	-1.489495	-2.012563	-0.289051
P	-3.165056	-0.416200	-0.148035
C	-3.471826	0.280272	1.530546
C	-4.564570	1.118771	1.811599
C	-4.752652	1.615661	3.101718
C	-3.852783	1.289777	4.122295
C	-2.765642	0.455960	3.852278
C	-2.581043	-0.051511	2.562691
C	-2.883273	1.094789	-1.170358
C	-2.183862	2.202727	-0.666202
C	-1.890549	3.291383	-1.491260
C	-2.290387	3.286820	-2.830195
C	-2.988109	2.188681	-3.339976
C	-3.279534	1.098676	-2.517930
O	-4.616093	-0.977116	-0.680890
O	-0.637888	-5.079665	-1.285231
Br	-7.142940	0.949231	-1.128480
H	-0.784762	5.815633	2.926333
H	1.106155	6.334571	1.399520
H	2.517784	4.514951	0.507251
H	0.171562	1.629250	2.679800
H	-1.254349	3.455477	3.561516
H	-5.347264	-0.302418	-0.782350
H	-1.519726	-4.855396	-1.615774
H	3.783929	-0.658098	2.250994
H	4.350352	3.608114	2.118543
H	5.758848	-0.868361	3.748914
H	6.310681	3.397415	3.601775
H	7.022987	1.162756	4.423741
H	1.075785	2.434312	-1.416544
H	5.169322	1.644806	-0.308603

H	1.808313	2.899491	-3.730135
H	5.888620	2.107096	-2.600289
H	1.155973	-5.109398	-2.943500
H	1.995934	-1.555562	-0.649919
H	3.077886	-4.597984	-4.430098
H	3.910761	-1.059454	-2.126899
H	4.461106	-2.572953	-4.022696
H	-3.832389	0.254616	-2.918791
H	-1.872776	2.225524	0.373647
H	-3.309448	2.181101	-4.377662
H	-1.356794	4.145061	-1.083169
H	-2.064348	4.134704	-3.470235
H	-5.275879	1.370969	1.028158
H	-1.743814	-0.712016	2.348017
H	-5.602637	2.258514	3.312822
H	-2.068780	0.192936	4.642931
H	-4.004230	1.679076	5.125090
H	-0.691026	-3.639836	2.211765
H	2.454784	-5.408455	-0.133616
H	0.255485	-4.679705	4.261582
H	3.391402	-6.449158	1.904027
H	2.300188	-6.086168	4.106964
H	5.530756	2.994777	-6.105928
H	6.166891	3.449289	-4.502495
H	5.938329	1.725088	-4.921006

Table S223. XYZ Coordinates of H_meta_I_P(O)Ph_2

86

scf done: -5571.384987

P	-1.555800	-2.386980	-0.112318
O	0.092860	-2.497128	-0.349127
Pd	-2.404506	-0.244983	0.002258
P	-3.319484	1.867612	0.143926
C	-4.246792	2.274711	1.675517
C	-5.121039	3.370770	1.758481
C	-5.769165	3.663745	2.958482
C	-5.556207	2.863236	4.085265
C	-4.691825	1.769041	4.011325
C	-4.040486	1.475813	2.811146
C	-2.203415	-3.475554	-1.443125
C	-2.002393	-4.865935	-1.441176
C	-2.471532	-5.645640	-2.498076
C	-3.150290	-5.047121	-3.564766
C	-3.357237	-3.666368	-3.574442
C	-2.885106	-2.883980	-2.517831
C	-1.749386	-3.421664	1.393407
C	-0.645945	-3.963346	2.066823
C	-0.832494	-4.718331	3.228835
C	-2.117935	-4.937512	3.726645
C	-3.223040	-4.397122	3.060820

C	-3.040407	-3.640292	1.903993
O	-2.232332	3.134992	0.105330
C	-4.490569	2.339976	-1.192325
C	-4.360421	3.543476	-1.899354
C	-5.261082	3.860079	-2.920546
C	-6.296915	2.980779	-3.241642
C	-6.429952	1.776743	-2.542552
C	-5.528416	1.454580	-1.528184
C	4.583122	1.877092	-0.899341
C	4.715155	2.773164	-1.970891
C	3.600725	3.134841	-2.727684
C	2.340902	2.611250	-2.426195
C	2.225471	1.729995	-1.354448
C	3.320741	1.355380	-0.581354
H	5.688636	3.193975	-2.199933
Br	0.477523	1.005515	-0.913122
H	-1.368025	2.802068	-0.185411
H	0.449696	-1.606141	-0.493870
H	3.190466	0.680409	0.256961
H	-1.483830	-5.339237	-0.612557
H	-3.044169	-1.808161	-2.517369
H	-2.311947	-6.719934	-2.489293
H	-3.886144	-3.199117	-4.399960
H	-3.518626	-5.657837	-4.383934
H	0.352931	-3.793830	1.679517
H	-3.904507	-3.216908	1.397330
H	0.029021	-5.135012	3.742814
H	-4.225406	-4.561457	3.445393
H	-2.260144	-5.522862	4.630316
H	-5.299159	3.992101	0.885924
H	-3.369358	0.622714	2.745319
H	-6.443243	4.513521	3.014413
H	-4.527017	1.143190	4.883479
H	-6.066431	3.091105	5.016617
H	-3.554267	4.225385	-1.649628
H	-5.627571	0.509952	-0.998173
H	-5.151871	4.794499	-3.463630
H	-7.230353	1.086551	-2.792943
H	-6.994735	3.228118	-4.036251
P	6.100298	1.452640	0.040833
H	1.471030	2.889007	-3.010808
H	3.706816	3.829380	-3.555133
C	5.549984	0.975765	1.715994
C	6.788389	-0.046401	-0.744505
O	7.084258	2.598432	0.060762
C	5.558680	1.972683	2.704671
C	5.136199	1.679419	4.001523
C	4.702722	0.389970	4.321244
C	4.699090	-0.608780	3.344486
C	5.124980	-0.320423	2.046259

H	5.907807	2.969879	2.455802
H	5.148030	2.454732	4.761690
H	4.374641	0.162193	5.331105
H	4.372515	-1.613975	3.593344
H	5.134542	-1.108453	1.299518
C	8.177074	-0.235613	-0.658411
C	8.768737	-1.365194	-1.224362
C	7.979898	-2.313138	-1.881503
C	6.598831	-2.127204	-1.978098
C	6.003148	-0.996456	-1.415689
H	8.789336	0.509280	-0.159784
H	9.843593	-1.503371	-1.155796
H	8.441182	-3.191541	-2.323045
H	5.985034	-2.857381	-2.496801
H	4.931214	-0.854724	-1.512663

Table S224. XYZ Coordinates of H_meta_TS1_P(O)Ph$_2$

86

scf done: -5571.356290

C	2.607898	3.513830	2.134743
C	1.484357	3.110128	1.399817
C	0.248997	2.978631	2.057576
C	0.140203	3.258188	3.419349
C	1.264654	3.665888	4.144497
C	2.496251	3.792643	3.500182
P	1.618142	2.637707	-0.373703
O	3.133871	3.186676	-0.800737
Pd	1.330879	0.304083	-0.863974
Br	0.852881	-0.411236	-3.484038
C	0.040542	-1.200407	-1.622538
C	0.163905	-2.594726	-1.438427
C	-0.829474	-3.277130	-0.752048
C	-1.981305	-2.612918	-0.271789
C	-2.122802	-1.242537	-0.527779
C	-1.150189	-0.537761	-1.245486
H	-2.806767	-3.128939	0.200603
P	2.946451	-0.898526	0.388737
C	2.410530	-1.622911	1.995546
C	2.489758	-0.848694	3.165434
C	2.010370	-1.345877	4.379311
C	1.440136	-2.619231	4.441162
C	1.350683	-3.394191	3.280987
C	1.827042	-2.900074	2.065481
C	3.920979	-2.265067	-0.377237
C	3.948222	-2.350970	-1.778671
C	4.726669	-3.319970	-2.416223
C	5.489411	-4.211431	-1.659441
C	5.472486	-4.133449	-0.263626
C	4.692640	-3.168581	0.374654
O	4.130420	0.173573	0.879610

C	0.492134	3.844202	-1.196285
C	-0.107204	3.470591	-2.410655
C	-0.924559	4.366314	-3.103174
C	-1.152982	5.644415	-2.588728
C	-0.561269	6.026799	-1.381424
C	0.256031	5.133427	-0.687976
H	-0.706915	-4.341860	-0.580814
H	1.044095	-3.117604	-1.795288
H	-1.331080	0.489840	-1.540643
P	-3.609002	-0.369037	-0.009891
H	-0.632819	2.663985	1.504952
H	3.570711	3.607799	1.642866
H	-0.821417	3.159192	3.914400
H	3.373393	4.108993	4.057273
H	1.179411	3.881712	5.205371
H	1.756642	-3.517730	1.175801
H	2.934792	0.140754	3.128029
H	0.921370	-4.390910	3.323290
H	2.087408	-0.738552	5.276498
H	1.072378	-3.007875	5.386092
H	0.066700	2.475150	-2.810852
H	-1.384195	4.064850	-4.039810
H	-1.792310	6.340162	-3.124205
H	-0.739130	7.019315	-0.977923
H	0.702738	5.439110	0.253328
H	3.230474	4.147664	-0.700967
H	4.681594	-3.126472	1.459711
H	6.064725	-4.825605	0.327785
H	6.094090	-4.966172	-2.153681
H	4.735035	-3.378433	-3.500657
H	3.351394	-1.661361	-2.369446
H	4.813490	-0.243709	1.429432
O	-3.489339	1.266938	-0.456284
C	-5.093593	-1.129315	-0.833034
C	-3.791074	-0.496092	1.836811
C	-6.371960	-0.616167	-0.583118
C	-7.489394	-1.188419	-1.202689
C	-7.328462	-2.273819	-2.072175
C	-6.050096	-2.786967	-2.322092
C	-4.932661	-2.214715	-1.702521
H	-6.497142	0.228120	0.093218
H	-8.483781	-0.789263	-1.008290
H	-8.197667	-2.718949	-2.554113
H	-5.924914	-3.631254	-2.998428
H	-3.938275	-2.613871	-1.896920
C	-4.879428	0.109432	2.476208
C	-5.016472	0.013799	3.866198
C	-4.065163	-0.687357	4.616792
C	-2.976809	-1.292881	3.977395
C	-2.839765	-1.197248	2.587404

H	-5.619410	0.654831	1.892353
H	-5.863055	0.484810	4.363557
H	-4.171763	-0.761745	5.698005
H	-2.236827	-1.838280	4.561249
H	-1.993182	-1.668259	2.090045

Table S225. XYZ Coordinates of H_meta_IIa_P(O)Ph$_2$

86

scf done: -5571.429455

C	-5.468258	2.007007	2.314762
C	-4.678421	2.589537	3.306963
C	-3.386639	2.107881	3.541489
C	-2.888694	1.048791	2.783920
C	-3.681216	0.453081	1.787236
C	-4.974068	0.942451	1.556929
P	-2.971810	-0.952668	0.851365
O	-2.820202	-2.022049	2.101677
Pd	-0.881157	-0.879279	-0.380092
Br	-0.685910	-3.384846	0.163897
C	0.906741	-1.083356	-1.384100
C	0.918276	-1.456361	-2.740376
C	2.122879	-1.709834	-3.411572
C	3.338438	-1.588284	-2.741628
C	3.350911	-1.205726	-1.391067
C	2.139801	-0.958928	-0.723755
H	4.272715	-1.798914	-3.252274
P	-0.733366	1.332006	-0.986160
C	0.280452	2.320549	0.170261
C	0.694614	3.615355	-0.188444
C	1.478531	4.365579	0.687153
C	1.853202	3.833065	1.925532
C	1.447791	2.546807	2.286129
C	0.667811	1.789408	1.408624
C	-4.355000	-1.613706	-0.151552
C	-5.319437	-2.454365	0.427127
C	-6.359062	-2.966453	-0.350005
C	-6.446101	-2.642605	-1.707084
C	-5.487800	-1.809836	-2.289530
C	-4.441125	-1.301963	-1.517225
O	-0.038936	1.559203	-2.463682
C	-2.245652	2.320347	-1.269108
C	-2.930458	2.170916	-2.487701
C	-4.087963	2.907464	-2.736211
C	-4.575361	3.794278	-1.771792
C	-3.902686	3.941146	-0.557685
C	-2.742616	3.206681	-0.302965
H	-5.247280	-2.712604	1.478814
H	0.554988	0.813646	-2.666541
H	-2.164205	-2.699786	1.823695
H	2.105678	-2.011332	-4.455471

H	-0.018206	-1.576322	-3.280246
H	2.159460	-0.683466	0.326261
P	4.983355	-1.055521	-0.579064
H	-3.692870	-0.664204	-1.978268
H	-7.099941	-3.618697	0.102767
H	-5.549000	-1.562157	-3.345023
H	-7.255682	-3.043264	-2.309905
H	-1.885423	0.680177	2.975369
H	-5.599880	0.493688	0.793205
H	-2.768473	2.556048	4.313624
H	-6.472712	2.376141	2.130503
H	-5.065860	3.415042	3.896655
H	0.405136	4.034342	-1.147200
H	0.364224	0.782917	1.682016
H	1.793953	5.365868	0.406307
H	1.740945	2.129539	3.244569
H	2.462138	4.420897	2.605752
H	-2.546165	1.498896	-3.248296
H	-2.228176	3.331951	0.643401
H	-4.604682	2.793426	-3.684358
H	-4.277049	4.628188	0.194650
H	-5.475320	4.369377	-1.968066
C	4.713040	-1.384454	1.199751
O	6.010244	-1.988024	-1.180417
C	5.482652	0.697632	-0.723786
C	4.904910	-2.702553	1.644004
C	4.698027	-3.031085	2.984155
C	4.300585	-2.046081	3.892474
C	4.117039	-0.730526	3.459555
C	4.325098	-0.397900	2.119126
H	5.227181	-3.462732	0.939203
H	4.850463	-4.052564	3.319728
H	4.141523	-2.301494	4.935972
H	3.819446	0.039199	4.165479
H	4.190201	0.630211	1.797893
C	6.857159	0.968832	-0.812556
C	7.309473	2.284874	-0.915240
C	6.393493	3.340022	-0.930623
C	5.023832	3.077157	-0.849992
C	4.567371	1.761110	-0.751542
H	7.564099	0.145032	-0.813736
H	8.374256	2.486254	-0.986133
H	6.745821	4.364189	-1.011107
H	4.308128	3.893381	-0.867283
H	3.499703	1.569554	-0.704766

Table S226. XYZ Coordinates of H_meta_IIb_P(O)Ph$_2$

86

scf done: -5571.432189

C	1.405944	4.142789	1.255819

C	0.517102	3.053946	1.261325
C	0.366556	2.296237	2.433369
C	1.098257	2.614981	3.579189
C	1.981454	3.696115	3.563720
C	2.132547	4.460583	2.402684
P	-0.525484	2.666640	-0.191952
Pd	-1.678491	0.612426	-0.040460
Br	-3.824610	1.883658	0.727321
C	0.528329	3.000265	-1.645907
C	1.864660	2.570778	-1.685781
C	2.634707	2.777594	-2.830569
C	2.079075	3.408912	-3.946467
C	0.749098	3.834250	-3.914356
C	-0.026272	3.629683	-2.771360
O	-1.591679	3.916432	-0.300008
P	-2.773875	-1.473341	0.041691
C	-3.818787	-1.804853	1.507927
C	-4.957180	-2.624297	1.444554
C	-5.673451	-2.919517	2.606054
C	-5.258558	-2.404138	3.836084
C	-4.124564	-1.590215	3.907148
C	-3.409296	-1.288504	2.748782
C	-0.013962	-0.323475	-0.730110
C	0.077963	-0.675602	-2.085641
C	1.203260	-1.343980	-2.579269
C	2.265462	-1.660680	-1.731635
C	2.197862	-1.301889	-0.377191
C	1.057845	-0.641507	0.114311
P	3.569555	-1.573014	0.797337
C	4.792466	-0.248840	0.474561
C	5.218556	0.522311	1.564943
C	6.136399	1.559089	1.379166
C	6.633571	1.831596	0.103577
C	6.211910	1.067275	-0.989392
C	5.294025	0.032579	-0.806904
O	-1.720641	-2.748601	0.026750
C	-3.850632	-1.773540	-1.410476
C	-3.529877	-2.775889	-2.338019
C	-4.327516	-2.967443	-3.469027
C	-5.444652	-2.159118	-3.684634
C	-5.764042	-1.152762	-2.767678
C	-4.970972	-0.954679	-1.637893
O	3.104853	-1.546656	2.238288
C	4.362381	-3.145825	0.311969
C	3.558596	-4.269836	0.053842
C	4.141624	-5.509313	-0.208579
C	5.533140	-5.641135	-0.213145
C	6.339785	-4.531561	0.047690
C	5.758566	-3.289583	0.310744
H	3.126526	-2.193519	-2.123125

H	1.247617	-1.622781	-3.628818
H	-0.735844	-0.441817	-2.767004
H	1.025243	-0.387630	1.168960
H	-2.161607	-3.595090	0.204916
H	-2.468223	3.583006	0.001530
H	-2.660097	-3.404423	-2.179111
H	-5.217256	-0.163395	-0.936152
H	-4.073167	-3.748685	-4.179071
H	-6.631030	-0.520102	-2.932949
H	-6.063450	-2.309419	-4.564236
H	-2.537397	-0.643481	2.802891
H	-5.287702	-3.029059	0.493382
H	-3.802211	-1.185039	4.861527
H	-6.554977	-3.550844	2.548480
H	-5.819036	-2.633732	4.737431
H	-1.055338	3.971658	-2.745060
H	2.308995	2.080596	-0.825836
H	0.313501	4.329541	-4.777069
H	3.668890	2.446989	-2.845911
H	2.680663	3.570139	-4.836107
H	-0.325456	1.458796	2.448371
H	1.533697	4.738463	0.357456
H	0.978065	2.020141	4.479503
H	2.817664	5.302915	2.390319
H	2.551689	3.944249	4.453983
H	4.822022	0.305039	2.551480
H	6.460827	2.151940	2.229229
H	7.346944	2.637514	-0.041652
H	6.598693	1.276351	-1.982390
H	4.972345	-0.550531	-1.664430
H	6.394032	-2.433163	0.511759
H	7.421119	-4.630722	0.045285
H	5.986511	-6.606104	-0.419794
H	3.511572	-6.370156	-0.411241
H	2.476629	-4.175776	0.051455

Table S227. XYZ Coordinates of H_meta_III_P(O)Ph$_2$

86

scf done: -5571.403933

C	6.545221	-0.677882	2.493199
C	6.003011	-0.144766	3.665452
C	4.627298	0.079042	3.762589
C	3.794634	-0.225366	2.685988
C	4.332687	-0.762427	1.501316
C	5.717001	-0.986540	1.413533
P	3.173725	-1.176471	0.148883
C	4.202661	-1.370011	-1.347160
C	4.316567	-2.605416	-1.999057
C	5.076745	-2.712669	-3.166417
C	5.718965	-1.590198	-3.691316

C	5.599446	-0.352720	-3.050542
C	4.840910	-0.239226	-1.886385
Pd	1.420689	0.410098	-0.011874
P	-0.258045	2.040320	-0.095765
O	-1.669876	1.494372	0.475753
C	0.306050	-0.832086	-1.105245
C	-0.713388	-1.561275	-0.486238
C	-1.490957	-2.456956	-1.239001
C	-1.233771	-2.626600	-2.607180
C	-0.213773	-1.896958	-3.216711
C	0.557066	-0.995797	-2.471800
H	-1.823047	-3.333865	-3.181776
C	-0.548922	2.824049	-1.719209
C	-1.770383	3.446062	-2.025104
C	-1.949361	4.045029	-3.272863
C	-0.918109	4.034960	-4.216283
C	0.297840	3.417326	-3.915946
C	0.479741	2.804441	-2.674748
C	0.277174	3.383803	1.029857
C	0.012416	3.264567	2.405373
C	0.480483	4.231772	3.294964
C	1.225789	5.316331	2.824071
C	1.498993	5.435308	1.459068
C	1.028861	4.474247	0.562824
O	2.608463	-2.687753	0.488539
Br	-4.037907	3.415909	1.139944
P	-2.867166	-3.417903	-0.509345
H	-0.902168	-1.438630	0.574593
H	1.349188	-0.438500	-2.961432
H	-0.008587	-2.027946	-4.275567
H	3.303623	-3.299924	0.779808
H	-2.391728	2.174781	0.681974
H	-2.574684	3.468907	-1.293860
H	1.419976	2.307515	-2.450248
H	-2.896181	4.522172	-3.507803
H	1.099191	3.401787	-4.648450
H	-1.063889	4.503435	-5.185102
H	-0.573389	2.428642	2.774588
H	1.235201	4.582903	-0.497457
H	0.260641	4.139554	4.354259
H	2.071445	6.280482	1.088981
H	1.589625	6.067725	3.518334
H	3.811499	-3.479705	-1.601875
H	4.752546	0.727171	-1.396425
H	5.163520	-3.673840	-3.663795
H	6.095337	0.522965	-3.457936
H	6.308137	-1.676188	-4.599238
H	2.724883	-0.052792	2.770580
H	6.148205	-1.396673	0.506647
H	4.203697	0.493034	4.672350

H	7.613899	-0.853833	2.418454
H	6.651995	0.097019	4.501747
C	-2.417579	-3.700411	1.241326
C	-4.328374	-2.321264	-0.518984
O	-3.115580	-4.710142	-1.253213
C	-1.694444	-4.868043	1.536031
C	-1.297134	-5.138592	2.845719
C	-1.619607	-4.247350	3.873055
C	-2.346752	-3.088893	3.589243
C	-2.748103	-2.816191	2.279327
H	-1.456262	-5.565986	0.739285
H	-0.740740	-6.045016	3.065164
H	-1.311820	-4.459141	4.892777
H	-2.609323	-2.400267	4.386767
H	-3.325856	-1.920544	2.074017
C	-5.578074	-2.916157	-0.751390
C	-6.736693	-2.137817	-0.754521
C	-6.656086	-0.762487	-0.521418
C	-5.414755	-0.161580	-0.295080
C	-4.252363	-0.935983	-0.301410
H	-5.633393	-3.983856	-0.939233
H	-7.699986	-2.604444	-0.938704
H	-7.559293	-0.158993	-0.520794
H	-5.339607	0.906599	-0.108379
H	-3.294966	-0.448517	-0.142844

Table S228. XYZ Coordinates of H_meta_IV_P(O)Ph$_2$

111

scf done: -6451.940187

C	-4.105578	-2.868269	3.988636
C	-3.019087	-2.071246	4.358216
C	-2.665035	-0.967305	3.582172
C	-3.404336	-0.645431	2.432395
C	-4.492485	-1.449001	2.065766
C	-4.839543	-2.555851	2.842332
P	-2.898579	0.817769	1.450874
C	-4.412070	1.353749	0.562545
C	-5.315226	2.228666	1.186673
C	-6.455738	2.659004	0.507139
C	-6.705036	2.218056	-0.795310
C	-5.809165	1.347326	-1.420414
C	-4.661256	0.920208	-0.749394
Pd	-0.874489	0.762694	0.096048
P	-1.149642	-1.516727	-0.463162
O	-2.745245	-1.746498	-0.675565
C	0.925979	0.723993	-0.946396
C	0.994209	0.936437	-2.330187
C	2.220520	0.920030	-3.006301
C	3.402828	0.682962	-2.309501
C	3.362404	0.464837	-0.921979

C	2.126254	0.492430	-0.253922
H	4.350477	0.671079	-2.840525
P	-0.515362	3.110700	0.168870
O	-1.568552	3.810494	1.265786
C	-0.919582	3.942403	-1.407503
C	-0.453652	5.232892	-1.712407
C	-0.855883	5.861893	-2.890847
C	-1.723992	5.213079	-3.772563
C	-2.196357	3.932822	-3.474098
C	-1.797302	3.299415	-2.297073
C	1.091058	3.804454	0.707578
C	1.253292	4.195595	2.047028
C	2.473374	4.714243	2.484451
C	3.540752	4.841779	1.593336
C	3.388878	4.442066	0.262661
C	2.173390	3.920348	-0.180485
O	-2.760719	1.881367	2.703235
C	-0.566833	-2.706473	0.799566
C	-0.983666	-4.048821	0.764425
C	-0.526708	-4.943843	1.731524
C	0.350704	-4.514271	2.732100
C	0.765288	-3.182134	2.772459
C	0.302232	-2.278636	1.813292
C	-0.413315	-2.111744	-2.032625
C	-1.071992	-1.822210	-3.238354
C	-0.520187	-2.223447	-4.455131
C	0.695202	-2.912313	-4.480614
C	1.354514	-3.204022	-3.284394
C	0.804042	-2.807070	-2.064142
Br	-3.922663	-4.430066	-1.787396
H	2.248236	1.090849	-4.079183
H	0.088408	1.117226	-2.900896
H	2.110146	0.344648	0.821231
P	4.958147	0.280365	-0.049666
H	-3.047184	-2.640934	-1.037641
H	-2.326894	2.702613	2.385511
H	-1.671776	-4.391246	-0.005261
H	0.612848	-1.239376	1.856400
H	-0.855481	-5.978521	1.703788
H	1.442670	-2.841494	3.548972
H	0.705759	-5.216454	3.480708
H	-2.022376	-1.298154	-3.225326
H	1.317619	-3.050581	-1.139617
H	-1.041676	-2.002300	-5.381643
H	2.294585	-3.747384	-3.299062
H	1.123870	-3.224746	-5.428177
H	-5.119232	2.574446	2.196468
H	-3.970998	0.236403	-1.232248
H	-7.149038	3.338036	0.994698
H	-6.000372	1.002657	-2.432383

H	-7.593397	2.554076	-1.321914
H	0.223622	5.746415	-1.038152
H	-2.167742	2.304978	-2.066569
H	-0.489551	6.857795	-3.119879
H	-2.872506	3.426835	-4.156211
H	-2.031826	5.704788	-4.690383
H	0.427540	4.108832	2.745321
H	2.070568	3.612185	-1.215526
H	2.584690	5.022708	3.519395
H	4.218074	4.531522	-0.431728
H	4.487225	5.251470	1.932877
H	-5.070985	-1.212975	1.179695
H	-1.823037	-0.350172	3.879062
H	-5.686573	-3.170299	2.552363
H	-2.446124	-2.308081	5.249763
H	-4.379519	-3.728322	4.592500
H	-1.549942	4.781263	1.293284
C	4.583525	-0.081552	1.704614
C	5.740474	-1.219452	-0.740441
O	5.852261	1.494183	-0.187001
C	7.142344	-1.257701	-0.793628
C	7.797267	-2.385794	-1.289603
C	7.057622	-3.484527	-1.734733
C	5.661478	-3.451777	-1.690036
C	5.003585	-2.322503	-1.198621
H	7.712064	-0.396956	-0.457625
H	8.882233	-2.406219	-1.331316
H	7.567537	-4.362267	-2.120787
H	5.084887	-4.301953	-2.042200
H	3.918298	-2.297278	-1.185282
C	4.448332	1.011243	2.577463
C	4.185034	0.803720	3.932004
C	4.066067	-0.496685	4.430319
C	4.208464	-1.588373	3.570170
C	4.462190	-1.383599	2.211922
H	4.558747	2.021432	2.195961
H	4.082050	1.655032	4.598307
H	3.871449	-0.658797	5.486388
H	4.131306	-2.600275	3.956611
H	4.576903	-2.239703	1.555023

Table S229. XYZ Coordinates of H_meta_V_P(O)Ph$_2$

110

scf done: -6451.488185

C	7.005014	0.425213	-0.758569
C	6.282498	1.071234	-1.764984
C	4.994741	1.543823	-1.508606
C	4.414362	1.379170	-0.240337
C	5.146812	0.731966	0.764525
C	6.434632	0.258322	0.505278

P	2.705685	2.021263	0.014392
C	2.647910	2.448994	1.802666
C	2.902371	3.766724	2.210884
C	2.838732	4.108732	3.563231
C	2.521262	3.139415	4.518206
C	2.261625	1.826286	4.116974
C	2.316275	1.480674	2.764657
Pd	0.738785	0.752557	-0.682964
P	1.825734	-1.351335	-0.409189
O	2.691329	-1.329782	0.972042
C	-0.980737	-0.249269	-1.269077
C	-1.898511	-0.754061	-0.331034
C	-3.054865	-1.451221	-0.726910
C	-3.296889	-1.670938	-2.093675
C	-2.392852	-1.187592	-3.036642
C	-1.255738	-0.482065	-2.626553
H	-4.182976	-2.209525	-2.417584
P	-0.363720	2.820767	-1.106217
O	0.607116	3.867895	-1.756237
C	-1.006446	3.559998	0.458057
C	-0.880064	4.944154	0.649758
C	-1.338784	5.539232	1.826447
C	-1.925249	4.756776	2.824997
C	-2.047301	3.376627	2.645209
C	-1.584777	2.779539	1.469994
C	-1.841460	2.824072	-2.211226
C	-1.631778	3.011410	-3.585996
C	-2.709684	3.026042	-4.473446
C	-4.011747	2.856905	-3.996980
C	-4.229789	2.677155	-2.628476
C	-3.151977	2.657956	-1.740387
O	2.818490	3.440658	-0.753930
C	2.980523	-1.688901	-1.798348
C	4.073900	-2.558320	-1.653218
C	4.914805	-2.806801	-2.738865
C	4.668862	-2.204228	-3.976452
C	3.583627	-1.338964	-4.126295
C	2.748273	-1.074759	-3.038329
C	0.875351	-2.921630	-0.285114
C	0.529366	-3.422264	0.979158
C	-0.192140	-4.612395	1.092534
C	-0.584160	-5.307683	-0.053567
C	-0.251165	-4.809438	-1.315623
C	0.476054	-3.624282	-1.432387
Br	4.394700	-3.757330	2.069081
P	-4.194127	-2.212563	0.477500
H	-1.713652	-0.614546	0.730300
H	-0.582525	-0.100863	-3.390274
H	-2.575435	-1.352488	-4.095690
H	3.198838	-2.158230	1.232967

H	1.896176	3.674503	-1.219006
H	4.275603	-3.034036	-0.696040
H	1.917554	-0.383249	-3.148501
H	5.762927	-3.474843	-2.619152
H	3.392077	-0.862008	-5.083085
H	5.324471	-2.405395	-4.818736
H	0.846319	-2.897312	1.873951
H	0.741984	-3.256455	-2.418302
H	-0.439997	-4.999148	2.076781
H	-0.549277	-5.347835	-2.210517
H	-1.144312	-6.233677	0.035958
H	3.141607	4.518172	1.465340
H	2.115071	0.459050	2.456555
H	3.036173	5.131852	3.870542
H	2.012116	1.070302	4.856124
H	2.472183	3.406903	5.569807
H	-0.411712	5.544171	-0.124526
H	-1.672797	1.704546	1.342484
H	-1.235687	6.611800	1.965697
H	-2.494731	2.761497	3.419919
H	-2.279486	5.219289	3.741780
H	-0.622497	3.162677	-3.955781
H	-3.338277	2.527742	-0.679056
H	-2.532550	3.175760	-5.534833
H	-5.241720	2.563502	-2.249786
H	-4.851764	2.872852	-4.685368
H	4.718038	0.597475	1.750956
H	4.443905	2.055794	-2.291087
H	6.991717	-0.239275	1.293730
H	6.720987	1.209768	-2.749077
H	8.007454	0.057221	-0.957253
C	-3.868884	-1.444582	2.108287
C	-5.877951	-1.688322	-0.008023
O	-4.086393	-3.721114	0.558337
C	-3.067838	-2.159369	3.012718
C	-2.798269	-1.640209	4.280420
C	-3.337054	-0.408455	4.660089
C	-4.143975	0.304322	3.768506
C	-4.406073	-0.207817	2.496242
H	-2.669389	-3.126035	2.722041
H	-2.177647	-2.200894	4.973130
H	-3.137120	-0.008925	5.649967
H	-4.578536	1.253852	4.066742
H	-5.043260	0.350267	1.817416
C	-6.932944	-2.573319	0.263271
C	-8.246477	-2.222214	-0.050954
C	-8.517419	-0.983958	-0.638935
C	-7.472107	-0.100321	-0.919025
C	-6.156424	-0.450573	-0.608455
H	-6.714988	-3.539169	0.708320

H	-9.056248	-2.914714	0.159209
H	-9.539552	-0.710764	-0.884284
H	-7.679803	0.858199	-1.385415
H	-5.348565	0.233137	-0.850528

Table S230. XYZ Coordinates of H_meta_TS2_P(O)Ph$_2$

110

scf done: -6451.449226

C	0.017674	-3.170208	-0.196810
C	-1.331087	-3.036879	-0.559582
C	-2.125848	-4.191226	-0.672063
C	-1.569897	-5.448138	-0.430106
C	-0.219505	-5.571221	-0.084053
C	0.577210	-4.430940	0.031381
P	-1.989825	-1.340533	-0.854018
O	-3.505663	-1.330734	-0.204885
Pd	-0.710529	0.540792	-0.013794
P	0.639140	2.513382	-0.429965
C	1.883873	3.242817	-1.603952
C	3.213614	3.331148	-1.161272
C	4.201745	3.868156	-1.987157
C	3.875816	4.320846	-3.268985
C	2.555482	4.240996	-3.714709
C	1.563705	3.707132	-2.886988
P	-1.466496	1.171216	2.150366
C	-3.111149	1.971371	2.399703
C	-3.248319	3.076794	3.252438
C	-4.497067	3.674769	3.434867
C	-5.619577	3.173401	2.770547
C	-5.487924	2.074881	1.916903
C	-4.239359	1.478403	1.724613
C	-1.507243	-0.184959	3.401984
C	-0.736476	-0.105658	4.570896
C	-0.762470	-1.145083	5.504808
C	-1.557282	-2.271425	5.281555
C	-2.325999	-2.357117	4.116914
C	-2.299447	-1.323730	3.178852
O	-0.442703	2.237762	2.860994
C	1.175138	0.534164	-1.096759
C	2.156087	0.006502	-0.234518
C	3.129841	-0.887405	-0.707233
C	3.146420	-1.228548	-2.069008
C	2.204531	-0.676147	-2.940547
C	1.232614	0.202849	-2.464399
H	3.897478	-1.911740	-2.454274
C	-2.316794	-1.340114	-2.670499
C	-3.362132	-0.549410	-3.178539
C	-3.568804	-0.443920	-4.554822
C	-2.728278	-1.117646	-5.445543
C	-1.683221	-1.901316	-4.950854

C	-1.477473	-2.012149	-3.573562
O	1.152413	2.955065	0.959852
C	-0.900199	3.402645	-0.939955
C	-1.265961	4.530866	-0.188380
C	-2.420474	5.246862	-0.510092
C	-3.222917	4.844076	-1.580702
C	-2.867414	3.719558	-2.329960
C	-1.714455	2.997974	-2.012417
Br	-5.894702	-3.235234	-1.172192
H	2.223635	-0.939803	-3.994119
H	0.508880	0.614531	-3.160172
H	2.155250	0.299585	0.810292
P	4.269868	-1.747997	0.433250
H	-4.174697	-1.969674	-0.583672
H	0.222561	2.546386	2.160637
H	-3.177603	-4.110601	-0.938369
H	0.632883	-2.282672	-0.089825
H	-2.191290	-6.335568	-0.514153
H	1.627104	-4.504429	0.301225
H	0.205852	-6.554210	0.098404
H	-4.026347	-0.031473	-2.493493
H	-0.667311	-2.632757	-3.203294
H	-4.389279	0.160713	-4.931000
H	-1.030305	-2.434295	-5.636358
H	-2.889565	-1.035440	-6.516451
H	-2.374163	3.467027	3.763619
H	-4.142787	0.631979	1.049071
H	-4.593126	4.531835	4.095873
H	-6.356782	1.685765	1.393185
H	-6.590291	3.639796	2.912897
H	-0.647028	4.840066	0.648021
H	-1.460984	2.121601	-2.600686
H	-2.691936	6.120126	0.076066
H	-3.487262	3.399625	-3.162383
H	-4.120393	5.402486	-1.829540
H	3.467340	2.990130	-0.162948
H	0.540924	3.668777	-3.247034
H	5.224857	3.937248	-1.628384
H	2.291298	4.600359	-4.705207
H	4.644843	4.738225	-3.912457
H	-2.890490	-1.408970	2.270883
H	-0.120040	0.770094	4.742598
H	-2.943883	-3.232177	3.935663
H	-0.162514	-1.072095	6.407864
H	-1.576056	-3.078962	6.007809
O	3.901722	-3.201001	0.652231
C	5.939146	-1.560671	-0.283184
C	4.248226	-0.808998	2.001905
C	3.569113	-1.379937	3.088486
C	3.498000	-0.703224	4.308087

C	4.107960	0.545136	4.450705
C	4.793720	1.115710	3.374021
C	4.865452	0.442625	2.153956
H	3.106914	-2.354978	2.972866
H	2.968966	-1.151430	5.143677
H	4.055034	1.070838	5.399524
H	5.275848	2.082218	3.485660
H	5.409673	0.891836	1.328948
C	6.858995	-2.597686	-0.063750
C	8.160971	-2.498782	-0.555940
C	8.554113	-1.364105	-1.270278
C	7.642121	-0.330415	-1.497903
C	6.337669	-0.427079	-1.009681
H	6.544803	-3.482473	0.481110
H	8.865823	-3.307057	-0.385455
H	9.567132	-1.287963	-1.654230
H	7.942824	0.547774	-2.061283
H	5.631891	0.373447	-1.210409

Table S231. XYZ Coordinates of H_meta_VI_P(O)Ph$_2$

110

scf done: -6451.483400

C	-6.521309	-1.543878	0.938443
C	-6.137845	-2.889342	0.934565
C	-4.787077	-3.228051	0.844116
C	-3.820580	-2.222174	0.756298
C	-4.195443	-0.870308	0.773393
C	-5.558645	-0.537190	0.863476
P	-2.871273	0.403782	0.669660
C	-3.073546	1.337171	2.243626
C	-2.784004	2.711224	2.278263
C	-2.815827	3.411370	3.485620
C	-3.124567	2.747594	4.675679
C	-3.406580	1.379082	4.651841
C	-3.381308	0.677692	3.444769
Pd	-0.712723	-0.313349	0.030792
P	-0.766216	0.078297	-2.316101
O	-1.880912	1.242997	-2.685801
P	1.720930	-2.628530	0.253135
C	3.254711	-3.108161	1.137425
C	4.157266	-3.953661	0.473404
C	5.330643	-4.368409	1.104843
C	5.613186	-3.942927	2.405490
C	4.723163	-3.096940	3.071550
C	3.550102	-2.677283	2.440560
C	1.270190	-0.987244	0.922244
C	0.278848	-0.818881	1.958434
C	0.388665	0.301924	2.848046
C	1.364106	1.250428	2.683632
C	2.319760	1.129973	1.620626

C	2.276330	0.034758	0.782745
H	1.444213	2.080551	3.378154
O	1.937892	-2.670634	-1.247733
C	0.449613	-3.833151	0.790183
C	0.377202	-4.327849	2.102617
C	-0.595363	-5.267450	2.448475
C	-1.495488	-5.731992	1.485287
C	-1.421210	-5.254312	0.174674
C	-0.454162	-4.307794	-0.172366
O	-3.404100	1.499142	-0.461414
C	-1.309398	-1.306491	-3.418334
C	-0.526383	-2.467373	-3.542888
C	-0.954838	-3.518653	-4.355983
C	-2.169949	-3.432282	-5.042622
C	-2.955548	-2.285442	-4.913955
C	-2.528241	-1.226628	-4.107213
C	0.682794	0.745078	-3.250710
C	0.560657	1.891739	-4.051578
C	1.662074	2.378610	-4.759959
C	2.893957	1.724592	-4.681298
C	3.021426	0.584105	-3.883507
C	1.927037	0.098660	-3.163064
Br	-6.077214	3.176299	-0.214674
H	-2.244646	-6.471045	1.753706
H	-0.642305	-5.644451	3.465732
H	1.085702	-3.995348	2.854872
H	-0.390230	-3.943647	-1.191881
H	-2.110746	-5.621466	-0.579754
H	-4.271953	1.983200	-0.304310
H	-2.471386	1.406964	-1.913110
H	3.941140	-4.272481	-0.541437
H	2.874176	-2.007793	2.964052
H	6.024585	-5.019641	0.581521
H	4.943836	-2.757666	4.079268
H	6.526778	-4.265027	2.896563
H	-0.296910	-1.674525	2.297060
H	3.033011	-0.090159	0.013536
H	-0.315810	0.380731	3.670645
P	3.582973	2.435538	1.503753
H	-0.396833	2.396532	-4.118388
H	2.042371	-0.782335	-2.535628
H	1.554980	3.266745	-5.376713
H	3.977492	0.072501	-3.815311
H	3.748702	2.102153	-5.235295
H	-2.551870	3.237078	1.357345
H	-3.612515	-0.383591	3.439369
H	-2.601473	4.476253	3.495301
H	-3.654430	0.857481	5.571937
H	-3.147871	3.293047	5.614524
H	-5.869384	0.504904	0.857998

H	-2.770032	-2.486320	0.668096
H	-7.573059	-1.279266	1.001624
H	-4.482033	-4.270230	0.835091
H	-6.891624	-3.669008	0.997362
H	0.408731	-2.556230	-2.995575
H	-3.133775	-0.330993	-4.016387
H	-0.340020	-4.409804	-4.449817
H	-3.901155	-2.209891	-5.443894
H	-2.501717	-4.254174	-5.670665
C	4.986964	1.754539	0.548065
C	2.856831	3.779243	0.497786
O	4.022120	2.931539	2.864367
C	3.209699	5.098029	0.822278
C	2.693018	6.166146	0.087304
C	1.820557	5.925380	-0.977234
C	1.460527	4.615113	-1.302848
C	1.971498	3.544252	-0.565987
H	3.877288	5.279093	1.658863
H	2.968135	7.184198	0.346989
H	1.418236	6.756669	-1.548825
H	0.782849	4.422538	-2.128880
H	1.673030	2.531626	-0.820889
C	6.050866	1.206213	1.282833
C	7.159537	0.671237	0.626490
C	7.217558	0.681665	-0.769705
C	6.166618	1.231764	-1.507097
C	5.055645	1.769329	-0.853071
H	6.010394	1.214711	2.367526
H	7.978311	0.251327	1.203253
H	8.081738	0.267946	-1.281133
H	6.212955	1.250353	-2.591836
H	4.252190	2.203144	-1.439501

Table S232. XYZ Coordinates of H_meta_VII_P(O)Ph$_2$

110

scf done: -6451.480096

C	-0.448283	-0.382840	-2.209630
C	-1.120041	-1.610142	-2.080621
C	-1.860797	-2.108866	-3.162846
C	-1.935860	-1.384079	-4.354419
C	-1.268826	-0.163215	-4.474972
C	-0.524098	0.336155	-3.402636
P	-0.981669	-2.488821	-0.485598
O	0.373215	-2.304639	0.164761
C	-2.329926	-1.843914	0.574161
C	-3.502803	-1.284192	0.055794
C	-4.501109	-0.791064	0.912572
C	-4.311112	-0.867319	2.299108
C	-3.138248	-1.417645	2.821770
C	-2.150364	-1.901384	1.966150

H	-5.072163	-0.490727	2.974232
C	-1.357775	-4.246564	-0.807965
C	-0.274830	-5.096499	-1.086178
C	-0.494334	-6.448247	-1.351797
C	-1.793974	-6.961808	-1.340831
C	-2.874829	-6.122971	-1.059333
C	-2.659779	-4.769701	-0.790404
C	2.267210	-3.482805	3.856502
C	3.194609	-3.702323	4.880564
C	4.534281	-3.361750	4.686857
C	4.949327	-2.803409	3.474048
C	4.023860	-2.582880	2.444678
C	2.673701	-2.922540	2.644995
P	4.520420	-1.787912	0.861579
C	4.286337	-3.142981	-0.358707
C	3.892876	-2.806350	-1.662946
C	3.733937	-3.797536	-2.634346
C	3.969211	-5.135385	-2.309321
C	4.363982	-5.480649	-1.012575
C	4.519307	-4.491242	-0.041384
Pd	3.655700	0.286048	0.418173
P	3.016202	2.492046	0.100373
C	3.209157	3.138803	-1.615225
C	3.631325	4.448283	-1.890850
C	3.770819	4.878646	-3.213743
C	3.491020	4.011320	-4.271721
C	3.074799	2.703413	-4.005536
C	2.943722	2.268447	-2.685882
C	1.249753	2.867567	0.489042
C	0.551430	3.959322	-0.052690
C	-0.770168	4.208653	0.320688
C	-1.412425	3.369482	1.236733
C	-0.731591	2.274848	1.774915
C	0.590895	2.026163	1.401158
O	3.887425	3.522884	1.039958
O	6.189512	-1.788940	1.015170
Br	3.286036	6.638231	1.406255
H	-1.325731	0.396818	-5.403688
H	-2.509988	-1.776884	-5.188112
H	-2.373631	-3.062078	-3.082691
H	0.137382	0.002030	-1.380200
H	0.001247	1.281893	-3.495887
H	3.589882	4.476085	1.085171
H	6.497274	-0.872820	0.962499
H	0.735430	-4.699391	-1.084675
H	-3.506142	-4.130021	-0.560421
H	0.348189	-7.099510	-1.564130
H	-3.884766	-6.521292	-1.042806
H	-1.963627	-8.014611	-1.546269
H	-3.636996	-1.215163	-1.019530

H	-1.231544	-2.312401	2.372222
P	-6.060238	-0.174884	0.176263
H	-2.995071	-1.463998	3.896757
H	5.991185	-2.542197	3.320970
H	1.941625	-2.753560	1.857916
H	5.259864	-3.530648	5.477635
H	1.223650	-3.747778	4.002903
H	2.872887	-4.135033	5.823529
H	1.122487	1.171157	1.812588
H	1.038382	4.617445	-0.764961
H	-1.229090	1.613330	2.478122
H	-1.297894	5.060006	-0.099752
H	-2.439430	3.569671	1.528755
H	3.840116	5.137784	-1.077242
H	2.643187	1.243257	-2.481953
H	4.098251	5.894684	-3.415749
H	2.865055	2.019907	-4.823370
H	3.602796	4.349154	-5.297953
H	3.707393	-1.763799	-1.908910
H	4.816453	-4.768771	0.965555
H	3.425086	-3.526015	-3.639585
H	4.547219	-6.520680	-0.758480
H	3.844759	-5.908049	-3.062527
C	-6.936383	0.741896	1.492740
C	-5.560638	1.051458	-1.081329
O	-6.923439	-1.273310	-0.403185
C	-6.397196	1.208822	-2.197467
C	-6.093418	2.155787	-3.176200
C	-4.952356	2.952291	-3.048901
C	-4.111563	2.797061	-1.944137
C	-4.411691	1.849705	-0.963580
H	-7.275770	0.579391	-2.298991
H	-6.743860	2.268879	-4.038363
H	-4.715642	3.687885	-3.811961
H	-3.218000	3.406068	-1.845700
H	-3.737750	1.727006	-0.121351
C	-8.131055	0.191286	1.979077
C	-8.850607	0.846444	2.980639
C	-8.381918	2.054459	3.500931
C	-7.193854	2.610882	3.017239
C	-6.473572	1.960012	2.015618
H	-8.491410	-0.743816	1.562569
H	-9.775455	0.414851	3.351612
H	-8.941754	2.564805	4.279118
H	-6.830729	3.552884	3.416967
H	-5.558058	2.408121	1.641556

Table S233. XYZ Coordinates of H_meta_I_Me

66

scf done: -4731.393090

C	2.439892	-2.670728	-1.902149
C	3.227381	-1.682092	-1.290953
C	4.626357	-1.786805	-1.353790
C	5.222923	-2.856713	-2.021560
C	4.430305	-3.835435	-2.629028
C	3.038285	-3.742069	-2.568994
P	2.376246	-0.272570	-0.475136
Pd	0.157987	-0.560123	0.068363
P	-2.064349	-0.841988	0.602849
O	-2.655241	0.070028	1.872765
O	2.730564	0.922119	-1.590617
C	3.502229	0.153516	0.917561
C	4.483191	1.148824	0.800289
C	5.312849	1.450574	1.883730
C	5.171975	0.760833	3.089891
C	4.192524	-0.229169	3.215518
C	3.356977	-0.525810	2.138240
C	-2.595116	-2.501541	1.183676
C	-3.946827	-2.875720	1.256966
C	-4.302978	-4.134362	1.741409
C	-3.314322	-5.034439	2.151618
C	-1.967663	-4.672975	2.078538
C	-1.609736	-3.411773	1.596388
C	-3.264747	-0.456379	-0.740744
C	-2.991923	-0.901285	-2.044642
C	-3.875764	-0.618855	-3.087187
C	-5.033601	0.126362	-2.841331
C	-5.303993	0.584855	-1.550329
C	-4.425280	0.294410	-0.502681
C	1.411474	4.092039	-1.041446
C	0.750162	3.702651	-2.210425
C	-0.571089	3.231021	-2.170243
C	-1.215011	3.150627	-0.925787
C	-0.538377	3.540546	0.226898
C	0.770226	4.015314	0.197698
C	-1.301559	2.842628	-3.433484
Br	-1.438935	3.420209	1.923545
H	1.260906	3.776484	-3.166485
H	2.055207	1.619617	-1.557628
H	-2.205120	0.932280	1.897469
H	-2.233432	2.782243	-0.863625
H	-4.720910	-2.187524	0.931151
H	-0.562584	-3.125206	1.534573
H	-5.350743	-4.415351	1.795157
H	-1.197103	-5.370515	2.393518
H	-3.594681	-6.015640	2.523568
H	-4.633495	0.652813	0.500380

H	-2.081578	-1.461877	-2.244363
H	-6.199103	1.168887	-1.356220
H	-3.657534	-0.971585	-4.091031
H	-5.716972	0.353464	-3.654302
H	5.250445	-1.036851	-0.877423
H	1.356421	-2.595096	-1.848342
H	6.305747	-2.929398	-2.064920
H	2.419247	-4.502192	-3.036524
H	4.897769	-4.669826	-3.143771
H	4.589137	1.687025	-0.136176
H	2.582795	-1.282193	2.246115
H	6.068542	2.224579	1.784237
H	4.073291	-0.762053	4.154433
H	5.816839	0.997722	3.931088
H	1.275851	4.314552	1.108121
H	2.430893	4.462448	-1.092002
H	-1.926174	1.958077	-3.278430
H	-1.960721	3.653525	-3.765918
H	-0.601258	2.632854	-4.246155

Table S234. XYZ Coordinates of H_meta_TS1_Me

66

scf done: -4731.361863

C	-2.389479	3.165097	-1.473979
C	-2.453596	2.115306	-0.547053
C	-2.690839	2.413604	0.806075
C	-2.873026	3.733248	1.217931
C	-2.813145	4.774518	0.285789
C	-2.570881	4.487730	-1.058374
P	-2.128845	0.371245	-1.041896
C	-3.743141	-0.444192	-0.674042
C	-3.740463	-1.813548	-0.361648
C	-4.939925	-2.490483	-0.130469
C	-6.154673	-1.805813	-0.207479
C	-6.168889	-0.442844	-0.517667
C	-4.971507	0.235294	-0.748947
Pd	-0.113138	-0.614857	-0.211000
Br	0.042366	-3.337338	-0.417670
C	0.719494	-1.989046	1.201956
C	2.079293	-2.098812	1.552883
C	2.431857	-1.869190	2.875776
C	1.458839	-1.579825	3.849846
C	0.105036	-1.539970	3.517604
C	-0.270747	-1.802564	2.180786
H	1.763345	-1.417360	4.879867
P	1.721007	0.749586	-0.841086
O	1.256094	1.733838	-2.111725
C	3.291612	0.005286	-1.464297
C	3.249662	-1.316928	-1.936227
C	4.388357	-1.912995	-2.483187

C	5.581843	-1.192809	-2.565940
C	5.635053	0.124470	-2.100951
C	4.498704	0.720252	-1.552183
C	2.316170	1.985090	0.390897
C	1.819975	3.298754	0.365406
C	2.190898	4.216471	1.351239
C	3.053439	3.832771	2.379897
C	3.546744	2.525274	2.417934
C	3.181427	1.607196	1.432923
O	-2.129758	0.446930	-2.710138
H	3.480458	-1.908952	3.158891
H	2.829625	-2.312228	0.800547
H	-1.321309	-1.898474	1.925553
C	-0.958509	-1.275624	4.557312
H	-2.739404	1.613506	1.540791
H	-2.194584	2.951307	-2.520077
H	-3.061705	3.949621	2.265447
H	-2.522460	5.291336	-1.787596
H	-2.952670	5.802229	0.607804
H	3.575642	0.596682	1.474386
H	1.146047	3.605456	-0.428384
H	4.221146	2.220477	3.213052
H	1.804787	5.231067	1.313046
H	3.340597	4.546414	3.146494
H	-2.795325	-2.346497	-0.299335
H	-4.924762	-3.549008	0.112163
H	-7.087693	-2.330476	-0.023602
H	-7.111964	0.092779	-0.575582
H	-4.994705	1.296214	-0.979565
H	-2.981115	0.737335	-3.075516
H	4.556658	1.741179	-1.186763
H	6.562080	0.687004	-2.163485
H	6.468815	-1.655732	-2.988559
H	4.343384	-2.937721	-2.840294
H	2.323537	-1.881389	-1.864231
H	1.966245	2.325275	-2.409921
H	-1.735479	-2.047801	4.535719
H	-0.532182	-1.249771	5.563490
H	-1.456079	-0.315120	4.378872

Table S235. XYZ Coordinates of H_meta_IIa_Me

66

scf done: -4731.435935

C	3.230130	1.368669	-0.807507
C	2.400814	0.582615	-1.622400
C	2.128680	1.007133	-2.934667
C	2.686661	2.189107	-3.423120
C	3.516691	2.963019	-2.607958
C	3.786133	2.550470	-1.301550
P	1.587270	-0.935560	-0.999022

C	2.836344	-1.743381	0.070867
C	4.195219	-1.794066	-0.284959
C	5.105705	-2.482444	0.515901
C	4.670555	-3.128072	1.677800
C	3.321794	-3.089085	2.035939
C	2.407819	-2.401884	1.233923
Pd	-0.685606	-0.915395	-0.108551
C	-2.648626	-1.057379	0.501348
C	-2.986405	-1.731660	1.687031
C	-4.332449	-1.888004	2.053309
C	-5.346538	-1.375357	1.247864
C	-5.035757	-0.694286	0.059764
C	-3.686043	-0.540718	-0.291605
H	-6.386492	-1.500312	1.539867
P	-0.743048	1.062576	1.055089
O	-1.642584	0.990764	2.433665
C	-1.489502	2.423095	0.088572
C	-1.188889	2.563082	-1.275592
C	-1.752796	3.605227	-2.012704
C	-2.628159	4.504492	-1.397771
C	-2.937259	4.363214	-0.042702
C	-2.369869	3.327538	0.701872
C	0.793159	1.744926	1.777607
C	1.559315	0.913144	2.611978
C	2.710829	1.402371	3.227048
C	3.110184	2.725449	3.012469
C	2.353406	3.556222	2.184610
C	1.198352	3.070270	1.567410
Br	-1.056186	-3.157782	-1.316401
O	1.602026	-1.819140	-2.394751
H	4.542174	-1.293038	-1.183370
H	-2.319212	0.290998	2.344819
H	0.826670	-2.424897	-2.364131
H	-4.581987	-2.416757	2.969983
H	-2.211296	-2.159024	2.319314
H	-3.448195	-0.004964	-1.208019
C	-6.135278	-0.136048	-0.814716
H	1.356462	-2.379172	1.507357
H	6.154144	-2.515308	0.234997
H	2.980306	-3.592740	2.935268
H	5.382681	-3.661530	2.300459
H	1.498584	0.401180	-3.577032
H	3.453391	1.059757	0.208221
H	2.476969	2.501325	-4.441935
H	4.430995	3.146101	-0.662675
H	3.952512	3.881429	-2.989966
H	-2.612172	3.214363	1.753329
H	-0.516791	1.862386	-1.762185
H	-3.620644	5.058177	0.435951
H	-1.514352	3.709838	-3.066803

H	-3.071703	5.310529	-1.974612
H	1.256552	-0.115164	2.783272
H	0.616308	3.725702	0.929106
H	3.295467	0.752457	3.870928
H	2.658474	4.584796	2.017883
H	4.008130	3.105677	3.490229
H	-5.728484	0.367595	-1.695902
H	-6.807421	-0.929861	-1.160674
H	-6.750861	0.586831	-0.266969

Table S236. XYZ Coordinates of H_meta_IIb_Me

66

scf done: -4731.437304

C	3.471992	1.641941	-1.348775
C	3.146160	1.637564	0.019206
C	3.441823	2.763534	0.801562
C	4.069821	3.872088	0.227901
C	4.401845	3.866834	-1.127702
C	4.101237	2.750408	-1.914392
P	2.267938	0.200500	0.742679
Pd	0.146946	-0.155214	-0.221232
Br	1.181162	-1.334312	-2.318435
C	3.455564	-1.191711	0.664860
C	2.956414	-2.496795	0.812877
C	3.828391	-3.584161	0.849534
C	5.205960	-3.379248	0.730294
C	5.709790	-2.085483	0.579539
C	4.840365	-0.993225	0.548932
O	2.202149	0.543921	2.360037
P	-2.021885	-0.428936	-1.118121
C	-3.022826	-1.729932	-0.306133
C	-2.358075	-2.744197	0.402334
C	-3.076550	-3.798613	0.968138
C	-4.465689	-3.848559	0.831295
C	-5.134987	-2.845553	0.124756
C	-4.419282	-1.790838	-0.442894
C	-0.690288	0.856073	1.331792
C	-0.663496	2.258891	1.327020
C	-1.235180	2.975925	2.382662
C	-1.840243	2.306808	3.448470
C	-1.876692	0.906331	3.476414
C	-1.298907	0.197345	2.408033
H	-2.287163	2.873179	4.262098
O	-1.944013	-0.903137	-2.692605
C	-3.123465	1.024458	-1.244936
C	-3.255837	1.690551	-2.473098
C	-4.055447	2.831466	-2.570913
C	-4.725930	3.317323	-1.446744
C	-4.594303	2.660178	-0.220231
C	-3.794509	1.522130	-0.115309

H	-1.211326	4.063132	2.369613
H	-0.199173	2.798830	0.505958
H	-1.340482	-0.888802	2.428280
C	-2.499938	0.166869	4.639073
H	3.078896	0.523905	2.776470
H	-1.005114	-1.131040	-2.888906
H	3.180269	2.777171	1.854311
H	3.229033	0.783041	-1.967590
H	4.297396	4.737814	0.842667
H	4.354713	2.743560	-2.970315
H	4.889617	4.729419	-1.571810
H	1.884937	-2.657759	0.887766
H	5.241424	0.008236	0.431374
H	3.433706	-4.589390	0.962245
H	6.779406	-1.924282	0.484972
H	5.884489	-4.226846	0.751794
H	-2.745300	1.306803	-3.349477
H	-3.694408	1.024954	0.844063
H	-4.156190	3.336058	-3.527279
H	-5.113517	3.033882	0.657241
H	-5.348869	4.203486	-1.524789
H	-1.277324	-2.706112	0.509301
H	-4.948508	-1.015665	-0.987459
H	-2.553231	-4.576290	1.516243
H	-6.214688	-2.883845	0.015595
H	-5.026072	-4.666524	1.274277
H	-2.944078	-0.780847	4.319124
H	-1.751170	-0.069669	5.405666
H	-3.279611	0.764191	5.121232

Table S237. XYZ Coordinates of H_meta_III_Me

66

scf done: -4731.407623

C	-4.733305	2.269761	-0.323802
C	-3.471504	1.861748	-0.788324
C	-2.633778	2.799070	-1.420844
C	-3.052825	4.119231	-1.585890
C	-4.307809	4.516618	-1.117828
C	-5.146194	3.592101	-0.489351
P	-2.874692	0.138412	-0.650635
O	-3.305456	-0.601506	-2.060161
Pd	-0.536727	0.041347	-0.254526
C	-0.424947	-1.855266	-0.877254
C	0.084849	-2.135227	-2.147718
C	0.130817	-3.456676	-2.625420
C	-0.343352	-4.485680	-1.800451
C	-0.845670	-4.203598	-0.529511
C	-0.891596	-2.886783	-0.056192
H	-0.318143	-5.512565	-2.156035
P	1.759364	0.118885	0.170193

C	2.407112	-0.932148	1.515877
C	3.767234	-1.275611	1.584854
C	4.229936	-2.066627	2.637153
C	3.348358	-2.513368	3.625690
C	1.995248	-2.174242	3.560575
C	1.522569	-1.393737	2.503613
C	2.068143	1.851759	0.686857
C	1.977706	2.239683	2.033888
C	2.119133	3.582406	2.387450
C	2.349273	4.547437	1.403560
C	2.438772	4.167923	0.061492
C	2.293405	2.828007	-0.299058
O	2.650301	-0.132827	-1.154494
C	-3.909704	-0.643390	0.636165
C	-4.719877	-1.749290	0.343988
C	-5.454881	-2.366773	1.359188
C	-5.379698	-1.890864	2.669035
C	-4.564565	-0.794423	2.968183
C	-3.828199	-0.174949	1.959402
Br	5.678182	0.613906	-1.255311
C	0.712005	-3.755073	-3.988602
H	0.458744	-1.333998	-2.778355
H	-1.295514	-2.681155	0.929752
H	-1.212150	-5.009699	0.100947
H	-4.221562	-0.418993	-2.325674
H	3.627775	0.132873	-1.121591
H	4.461978	-0.923215	0.826360
H	0.466067	-1.146093	2.444736
H	5.281647	-2.332882	2.686004
H	1.306288	-2.524676	4.323148
H	3.714978	-3.128299	4.442277
H	2.373424	2.536525	-1.341466
H	1.812355	1.496338	2.807761
H	2.624951	4.913979	-0.705180
H	2.057193	3.872419	3.432019
H	2.462042	5.590942	1.681811
H	-4.774810	-2.131157	-0.670071
H	-3.197310	0.675956	2.203043
H	-6.083188	-3.220344	1.123592
H	-4.501972	-0.422560	3.986308
H	-5.950620	-2.373379	3.456509
H	-1.657432	2.495846	-1.789711
H	-5.390449	1.559863	0.167329
H	-2.399819	4.835581	-2.074704
H	-6.122415	3.899845	-0.127372
H	-4.631862	5.545542	-1.241668
H	0.289978	-4.672513	-4.409027
H	0.525036	-2.937877	-4.691658
H	1.799094	-3.891881	-3.931457

Table S238. XYZ Coordinates of H_meta_IV_Me

91

scf done: -5611.946314

C	-1.435220	-3.545500	-1.246824
C	-1.538619	-2.179157	-1.545374
C	-1.652052	-1.774564	-2.884936
C	-1.665252	-2.723261	-3.907793
C	-1.560389	-4.083013	-3.604196
C	-1.445938	-4.491481	-2.273392
P	-1.476362	-0.895629	-0.237239
C	-2.227292	-1.698697	1.228349
C	-3.549624	-2.174871	1.190666
C	-4.101514	-2.786327	2.316180
C	-3.342523	-2.936279	3.481203
C	-2.030987	-2.460562	3.526253
C	-1.477898	-1.836373	2.405905
Pd	0.581790	0.154547	0.243651
P	2.821057	0.913565	0.476816
C	4.041485	-0.121641	1.366417
C	4.258300	0.100251	2.736383
C	5.150583	-0.705542	3.446245
C	5.829742	-1.738883	2.798234
C	5.611781	-1.969142	1.437207
C	4.718160	-1.170967	0.722681
P	-0.462260	2.209042	1.037604
O	0.398406	2.948297	2.232434
C	-0.609526	3.485862	-0.272733
C	-1.041653	3.135540	-1.562562
C	-1.127183	4.109440	-2.559672
C	-0.769306	5.431502	-2.283149
C	-0.330068	5.781931	-1.003907
C	-0.251165	4.815005	-0.000031
C	-2.088538	2.191357	1.885150
C	-3.254738	2.685619	1.285895
C	-4.468123	2.647871	1.975353
C	-4.527073	2.118703	3.266326
C	-3.367550	1.624330	3.869369
C	-2.154230	1.655542	3.181674
C	1.494705	-1.594557	-0.416460
C	1.911180	-1.738817	-1.748426
C	2.538103	-2.916418	-2.169721
C	2.751822	-3.964367	-1.271908
C	2.343684	-3.847436	0.062893
C	1.717532	-2.656923	0.469732
H	3.237153	-4.877406	-1.608509
O	-2.503708	0.245943	-0.775806
O	2.856036	2.322130	1.382838
C	3.614302	1.397390	-1.096459
C	5.009220	1.503862	-1.228611
C	5.566163	1.961126	-2.423234

C	4.739908	2.316256	-3.492436
C	3.351972	2.217957	-3.367211
C	2.790104	1.760812	-2.175097
Br	-5.377630	-0.484916	-1.825319
H	2.857924	-3.016022	-3.204346
H	1.752815	-0.939856	-2.466682
H	1.410946	-2.571639	1.509812
C	2.601882	-4.955707	1.057968
H	-3.411511	-0.061225	-1.094667
H	1.360115	2.823526	2.074589
H	-4.151430	-2.052086	0.293370
H	-0.463938	-1.449095	2.447967
H	-5.125258	-3.147320	2.283885
H	-1.441425	-2.566580	4.431981
H	-3.775899	-3.417841	4.352880
H	-1.748546	-0.721045	-3.126918
H	-1.350190	-3.874829	-0.216688
H	-1.762397	-2.400264	-4.940103
H	-1.370625	-5.547522	-2.031581
H	-1.571730	-4.821000	-4.400865
H	0.093372	5.085615	0.992738
H	-1.326709	2.110664	-1.777830
H	-0.048861	6.808164	-0.786315
H	-1.469558	3.833931	-3.552851
H	-0.829988	6.185463	-3.062445
H	5.660914	1.227656	-0.406266
H	1.710737	1.684818	-2.080562
H	6.644856	2.038413	-2.518118
H	2.707520	2.494066	-4.196047
H	5.177276	2.668929	-4.421596
H	3.740449	0.904983	3.247704
H	4.550279	-1.367943	-0.330693
H	5.315327	-0.520930	4.503385
H	6.137129	-2.771680	0.928585
H	6.525859	-2.362874	3.350584
H	-3.219920	3.101855	0.285264
H	-1.257665	1.272390	3.658796
H	-5.365702	3.036106	1.503342
H	-3.406707	1.213204	4.873699
H	-5.471970	2.092363	3.801019
H	3.727491	2.742967	1.462347
H	1.778588	-5.053798	1.772752
H	2.737130	-5.919524	0.558563
H	3.511466	-4.756616	1.638879

Table S239. XYZ Coordinates of H_meta_V_Me

90

scf done: -5611.492858

C	3.453602	0.877751	-1.972693
C	3.599622	1.468607	-0.708047

C	4.330726	2.660182	-0.591941
C	4.918961	3.241395	-1.717242
C	4.781368	2.639284	-2.970831
C	4.047888	1.456573	-3.096668
P	2.834077	0.749341	0.812015
O	2.882954	1.856363	1.923442
Pd	0.614812	0.010826	0.360343
C	1.362663	-1.896242	0.013161
C	1.606169	-2.358072	-1.289137
C	2.084678	-3.652940	-1.555944
C	2.313739	-4.515539	-0.476689
C	2.067660	-4.085279	0.829249
C	1.604050	-2.788417	1.072159
H	2.681910	-5.522714	-0.659136
P	-1.568284	-0.846353	-0.069252
O	-2.441784	0.261291	-0.891970
C	-2.481015	-1.226063	1.479435
C	-1.760002	-1.449391	2.662340
C	-2.427307	-1.753430	3.851226
C	-3.821701	-1.819831	3.869964
C	-4.546651	-1.585851	2.697510
C	-3.883965	-1.294701	1.504409
C	-1.780054	-2.368697	-1.078425
C	-1.781045	-2.258145	-2.478102
C	-1.897793	-3.396982	-3.276110
C	-2.007544	-4.658995	-2.685661
C	-2.004949	-4.776301	-1.293718
C	-1.893898	-3.637739	-0.492926
P	-0.179986	2.276479	0.785114
O	0.731271	3.048609	1.877671
C	4.082885	-0.494699	1.362371
C	4.242248	-0.693349	2.742394
C	5.167248	-1.621812	3.224434
C	5.946523	-2.362286	2.332343
C	5.798241	-2.166303	0.956787
C	4.872146	-1.239289	0.474458
C	-1.855121	2.572266	1.491412
C	-2.928432	3.075178	0.744021
C	-4.174399	3.274115	1.343016
C	-4.362367	2.972299	2.693351
C	-3.297268	2.470619	3.446247
C	-2.052282	2.270822	2.849088
C	-0.118575	3.335732	-0.718187
C	0.489480	4.598258	-0.654600
C	0.573593	5.397483	-1.796746
C	0.051427	4.943958	-3.010665
C	-0.551908	3.685286	-3.081458
C	-0.631601	2.879043	-1.943722
Br	-5.372479	-0.307409	-1.964005
C	2.304873	-4.111104	-2.980490

H	1.404036	-1.708747	-2.139400
H	1.447421	-2.474551	2.101322
H	2.247677	-4.759784	1.663802
H	-3.360395	-0.005638	-1.199436
H	1.676986	2.571738	1.946825
H	-4.459529	-1.113631	0.599212
H	-0.676363	-1.378218	2.653279
H	-5.631865	-1.631552	2.711673
H	-1.859127	-1.927828	4.760291
H	-4.343049	-2.048760	4.794944
H	-1.706939	-1.280540	-2.943894
H	-1.902456	-3.741260	0.587355
H	-1.907922	-3.297911	-4.357676
H	-2.097406	-5.753573	-0.828838
H	-2.099963	-5.545019	-3.306941
H	0.899602	4.943755	0.288929
H	-1.102418	1.902238	-2.000809
H	1.047316	6.373392	-1.738393
H	-0.957759	3.328410	-4.023862
H	0.117692	5.566384	-3.898373
H	4.427001	3.126775	0.383616
H	2.877074	-0.035543	-2.078670
H	5.482858	4.164620	-1.616238
H	3.932915	0.987508	-4.069842
H	5.238317	3.092163	-3.846044
H	3.651051	-0.105333	3.437358
H	4.772434	-1.095793	-0.596482
H	5.282302	-1.762367	4.295664
H	6.407255	-2.732157	0.257279
H	6.668386	-3.082934	2.705936
H	-2.795993	3.316747	-0.304734
H	-1.228388	1.887233	3.443171
H	-4.996737	3.668419	0.753022
H	-3.434479	2.235703	4.497811
H	-5.332028	3.128437	3.157176
H	2.966679	-4.981362	-3.024096
H	1.356591	-4.395114	-3.454128
H	2.744651	-3.318370	-3.594826

Table S240. XYZ Coordinates of H_meta_TS2_Me

90

scf done: -5611.454364

C	1.817404	0.639237	-2.660937
C	2.464044	1.322928	-1.616446
C	2.826580	2.667017	-1.798391
C	2.555593	3.312541	-3.006506
C	1.918702	2.626195	-4.043609
C	1.550948	1.289602	-3.867946
P	2.851592	0.555978	0.023914
O	3.254878	1.611603	1.081222

Pd	0.490119	0.167440	0.343635
C	2.030498	-1.311103	0.757382
C	2.241592	-1.372618	2.147071
C	2.468182	-2.593152	2.805628
C	2.513944	-3.761764	2.036441
C	2.336826	-3.713549	0.648262
C	2.108440	-2.499548	0.005172
H	2.704572	-4.715619	2.521670
P	-1.509533	-1.064237	-0.257612
C	-1.357008	-2.122368	-1.762954
C	-1.512137	-1.540747	-3.032865
C	-1.298262	-2.292390	-4.189613
C	-0.916077	-3.633430	-4.096102
C	-0.754473	-4.219519	-2.838361
C	-0.972425	-3.470546	-1.679669
C	-2.245992	-2.225773	0.972141
C	-1.460793	-2.634529	2.061184
C	-1.976370	-3.515042	3.017035
C	-3.286603	-3.981379	2.901061
C	-4.079899	-3.568413	1.825462
C	-3.565386	-2.699164	0.862994
O	-2.725127	-0.024557	-0.659891
P	-0.050561	2.348713	1.115290
O	1.259142	3.104890	1.750686
C	4.453916	-0.305894	-0.364646
C	5.385000	-0.441292	0.677272
C	6.610076	-1.071669	0.456991
C	6.920932	-1.579825	-0.807461
C	6.002272	-1.446397	-1.850095
C	4.776499	-0.811513	-1.631567
C	-0.749709	3.640380	-0.003431
C	-0.269911	4.958046	0.036429
C	-0.804290	5.928508	-0.813848
C	-1.823692	5.593473	-1.708980
C	-2.303012	4.281670	-1.755417
C	-1.765792	3.305322	-0.912929
C	-1.227414	2.395861	2.537205
C	-0.841225	2.939172	3.771020
C	-1.731929	2.948094	4.848053
C	-3.015509	2.417446	4.703390
C	-3.405773	1.872712	3.476175
C	-2.517407	1.855794	2.399646
Br	-5.487288	-0.894460	-2.036466
H	2.377175	-4.631494	0.067630
H	1.976022	-2.482059	-1.071598
H	2.245000	-0.452622	2.726695
C	2.648584	-2.633820	4.305568
H	-3.544695	-0.409071	-1.083208
H	2.080451	2.550991	1.526599
H	-4.195064	-2.384430	0.033479

H	-0.445498	-2.261962	2.160595
H	-5.101882	-3.926267	1.735844
H	-1.356884	-3.828000	3.852721
H	-3.691446	-4.660255	3.646255
H	-1.820164	-0.503172	-3.115035
H	-0.851340	-3.941900	-0.709179
H	-1.435292	-1.831289	-5.163742
H	-0.465249	-5.263565	-2.757003
H	-0.749988	-4.217895	-4.996350
H	0.524625	5.214536	0.729698
H	-2.135312	2.284144	-0.965047
H	-0.424611	6.945976	-0.776862
H	-3.091124	4.014993	-2.454272
H	-2.238156	6.349055	-2.370353
H	3.315332	3.202333	-0.990333
H	1.513088	-0.395696	-2.539535
H	2.843420	4.351843	-3.137063
H	1.052933	0.750237	-4.668370
H	1.709030	3.129717	-4.982656
H	5.149930	-0.039718	1.657499
H	4.083298	-0.704923	-2.459289
H	7.322901	-1.163605	1.271719
H	6.239212	-1.829923	-2.838509
H	7.874289	-2.071130	-0.979277
H	-2.826715	1.414127	1.456019
H	0.155471	3.353164	3.880491
H	-4.401763	1.454930	3.358178
H	-1.421696	3.371962	5.799265
H	-3.706789	2.424571	5.541246
H	3.156904	-3.549193	4.621133
H	3.231548	-1.778036	4.660365
H	1.680781	-2.600509	4.820829

Table S241. XYZ Coordinates of H_meta_VI_Me

90

scf done: -5611.487536

C	-2.243002	3.350497	-1.327495
C	-0.841705	3.451542	-1.279809
C	-0.192561	4.353520	-2.136690
C	-0.930877	5.148650	-3.017326
C	-2.323801	5.054091	-3.051275
C	-2.975963	4.154242	-2.203386
P	0.121879	2.370042	-0.128366
C	-0.166547	3.205376	1.499485
C	-1.421816	3.127847	2.127942
C	-1.625998	3.739896	3.366500
C	-0.582853	4.423062	3.999114
C	0.668754	4.492795	3.384373
C	0.876980	3.888681	2.141056
Pd	0.009250	0.011618	-0.312994

P	2.264603	-0.639186	-0.097682
O	3.201743	0.665140	-0.533716
C	-2.058922	-0.806201	-0.927944
C	-1.104133	-1.878376	-0.898759
C	-0.640870	-2.420417	-2.139795
C	-1.048799	-1.895243	-3.342123
C	-1.968927	-0.801752	-3.396366
C	-2.463149	-0.295639	-2.214553
P	-3.240886	-0.515561	0.431852
O	-3.552194	0.943384	0.710710
H	-0.686231	-2.326304	-4.272278
C	-2.629332	-1.376263	1.929910
C	-2.103682	-0.593157	2.968867
C	-1.653341	-1.193863	4.146883
C	-1.722458	-2.581105	4.296695
C	-2.250955	-3.367388	3.269011
C	-2.709093	-2.768745	2.094040
C	-4.767950	-1.411627	-0.052454
C	-5.994690	-0.925101	0.425719
C	-7.186207	-1.577253	0.105118
C	-7.163549	-2.721534	-0.696618
C	-5.947637	-3.208804	-1.182180
C	-4.754350	-2.555813	-0.865542
O	1.652361	2.896829	-0.479305
C	2.944715	-1.980176	-1.164261
C	3.426019	-1.685070	-2.449891
C	3.826532	-2.710199	-3.309741
C	3.742707	-4.043552	-2.902269
C	3.259543	-4.347528	-1.626152
C	2.862320	-3.324382	-0.763768
C	2.866929	-1.122185	1.575760
C	4.233978	-1.215889	1.891226
C	4.632262	-1.586415	3.175764
C	3.676436	-1.876987	4.155298
C	2.317723	-1.785617	3.850429
C	1.915405	-1.402240	2.567811
Br	6.359832	0.656369	-0.819760
H	-1.376668	-3.047566	5.214526
H	-2.317944	-4.444903	3.386117
H	-3.141673	-3.389328	1.315436
H	-2.060407	0.484729	2.856781
H	-1.254985	-0.577962	4.947781
H	4.199746	0.562426	-0.584845
H	2.272233	2.131718	-0.523555
H	-6.010122	-0.028196	1.036975
H	-3.816872	-2.935442	-1.260785
H	-8.130668	-1.191165	0.477369
H	-5.927385	-4.092864	-1.812678
H	-8.090834	-3.228613	-0.946935
H	-0.979148	-2.475391	-0.001322

H	-3.191217	0.510784	-2.245225
H	0.040383	-3.266003	-2.114535
C	-2.394898	-0.247469	-4.734693
H	0.888849	4.430120	-2.108689
H	-2.761242	2.639144	-0.688859
H	-0.415611	5.843764	-3.674783
H	-4.059295	4.071363	-2.229072
H	-2.896809	5.672690	-3.736259
H	3.506408	-0.651431	-2.770820
H	2.496614	-3.575229	0.228225
H	4.207392	-2.465036	-4.297202
H	3.198121	-5.381560	-1.298706
H	4.053941	-4.839993	-3.571869
H	4.986604	-0.984725	1.141041
H	0.857413	-1.314186	2.335007
H	5.690329	-1.650638	3.413886
H	1.569647	-2.004138	4.606776
H	3.992409	-2.168903	5.152805
H	-2.234879	2.579773	1.658327
H	1.846971	3.950566	1.659176
H	-2.601573	3.678196	3.841411
H	1.485615	5.020555	3.869243
H	-0.744406	4.893532	4.964867
H	-3.145718	0.539303	-4.621485
H	-1.542668	0.177626	-5.278878
H	-2.818568	-1.031551	-5.373706

Table S242. XYZ Coordinates of H_meta_VII_Me

90

scf done: -5611.488678

C	2.175726	2.286375	1.598067
C	1.425756	3.474150	1.540563
C	1.634533	4.468410	2.506870
C	2.587252	4.282793	3.512996
C	3.336726	3.105752	3.561094
C	3.129291	2.109364	2.601695
P	0.158900	3.656569	0.219377
O	-0.506635	5.147451	0.600192
Pd	-1.396284	1.986342	0.031947
P	-3.136513	0.455797	-0.007903
O	-4.575603	1.146024	0.385196
C	-2.971714	-0.933050	1.197674
C	-2.256651	-2.097281	0.873437
C	-2.052298	-3.093317	1.830986
C	-2.556107	-2.938412	3.125424
C	-3.268003	-1.782860	3.456807
C	-3.471656	-0.785075	2.501484
C	-3.410796	-0.429721	-1.601437
C	-4.527885	-1.253973	-1.823428
C	-4.687394	-1.903728	-3.047667

C	-3.734574	-1.746616	-4.060119
C	-2.623161	-0.928128	-3.849196
C	-2.466983	-0.267419	-2.626881
C	1.160703	4.121941	-1.250754
C	0.690252	3.750655	-2.520214
C	1.389264	4.118834	-3.671809
C	2.567630	4.860577	-3.564274
C	3.045616	5.233469	-2.304275
C	2.348510	4.864856	-1.153245
Br	-7.267721	-0.523315	0.882194
O	1.881228	-0.403643	-0.590712
P	2.600849	-1.729133	-0.423197
C	4.136899	-1.776148	-1.415761
C	4.589020	-0.570298	-1.970654
C	5.752896	-0.543717	-2.742130
C	6.470723	-1.720472	-2.964223
C	6.023438	-2.926785	-2.416490
C	4.860711	-2.957023	-1.646569
C	3.064700	-2.088544	1.305678
C	4.384432	-1.940972	1.758243
C	4.715773	-2.116290	3.108809
C	3.688287	-2.436216	4.007612
C	2.370097	-2.581528	3.571719
C	2.052035	-2.409200	2.225014
C	6.145549	-1.988878	3.579722
C	1.573545	-3.135752	-0.976148
C	0.560213	-2.870768	-1.910223
C	-0.241591	-3.906440	-2.392992
C	-0.034945	-5.215180	-1.948935
C	0.970544	-5.486403	-1.017325
C	1.770925	-4.451635	-0.528823
H	3.923652	-2.571959	5.060199
H	-0.659338	-6.021058	-2.323205
H	1.127623	-6.501205	-0.664358
H	2.534083	-4.671555	0.211426
H	0.394465	-1.851354	-2.243373
H	-1.029046	-3.688201	-3.108139
H	-5.354968	0.532368	0.511740
H	-1.432284	5.009955	0.848211
H	4.020726	0.337987	-1.798386
H	4.519710	-3.902096	-1.234805
H	6.095889	0.393783	-3.169586
H	6.577799	-3.843835	-2.592027
H	7.375034	-1.700502	-3.565333
H	1.023304	-2.523207	1.896100
H	5.171107	-1.689370	1.053291
H	1.588008	-2.828318	4.283602
H	1.049213	5.381329	2.469766
H	2.015635	1.497475	0.865296
H	2.741760	5.058405	4.257922

H	3.704490	1.188356	2.639117
H	4.074824	2.961957	4.345037
H	-4.036141	0.104539	2.763151
H	-1.863687	-2.235501	-0.129240
H	-3.669142	-1.658263	4.458649
H	-1.506329	-3.992997	1.561197
H	-2.398952	-3.714563	3.868759
H	-5.280724	-1.376024	-1.047908
H	-1.609743	0.381304	-2.461094
H	-5.556221	-2.534532	-3.213519
H	-1.884731	-0.795548	-4.634725
H	-3.863346	-2.255252	-5.011306
H	-0.225905	3.170426	-2.598285
H	2.730920	5.152227	-0.178437
H	1.016596	3.824341	-4.648583
H	3.963095	5.808600	-2.218825
H	3.114840	5.145008	-4.458398
H	6.753514	-1.427982	2.864838
H	6.201721	-1.483671	4.548754
H	6.607644	-2.975964	3.702653

Table S243. XYZ Coordinates of H_meta_I_OMe

67

scf done: -4806.597887

C	2.029306	1.218077	2.724929
C	2.672402	1.605037	1.539573
C	3.916772	2.252996	1.614965
C	4.505647	2.502590	2.854075
C	3.856147	2.114319	4.030932
C	2.617764	1.473031	3.966477
P	1.875497	1.197219	-0.066346
Pd	-0.306113	0.459859	-0.002161
P	-2.517728	-0.182584	0.036063
O	-2.873417	-1.585962	-0.793904
O	2.975593	0.141552	-0.749825
C	2.213354	2.697843	-1.071577
C	3.093437	2.679312	-2.162248
C	3.299786	3.836771	-2.919240
C	2.630535	5.018036	-2.595669
C	1.748359	5.042135	-1.510335
C	1.537695	3.889002	-0.755315
C	-3.738249	0.996586	-0.669825
C	-5.121411	0.854330	-0.468729
C	-6.014060	1.745519	-1.063636
C	-5.535882	2.790940	-1.860819
C	-4.163022	2.942032	-2.064780
C	-3.268270	2.047544	-1.472234
C	-3.243017	-0.579125	1.677827
C	-3.276058	0.419193	2.666629
C	-3.783890	0.140249	3.934908

C	-4.257780	-1.141439	4.234189
C	-4.222251	-2.139207	3.258908
C	-3.716741	-1.861928	1.984998
C	2.343605	-3.308628	0.459879
C	1.324985	-2.848593	-0.395636
C	1.216286	-3.402250	-1.664790
C	2.077900	-4.397018	-2.124920
C	3.080572	-4.836529	-1.259754
C	3.226110	-4.306994	0.024938
O	2.381379	-2.718456	1.683998
Br	-0.175659	-2.774432	-2.836331
H	4.015018	-4.674475	0.668924
H	2.561762	-0.729325	-0.855085
H	-2.083643	-1.891177	-1.270088
H	0.645020	-2.071607	-0.049909
H	-5.500050	0.050487	0.155838
H	-2.197283	2.159022	-1.625121
H	-7.081932	1.628403	-0.903495
H	-3.788506	3.753959	-2.681305
H	-6.233435	3.486270	-2.318702
H	-3.692034	-2.636616	1.226176
H	-2.904873	1.417072	2.444926
H	-4.589120	-3.136190	3.485878
H	-3.807305	0.920435	4.690202
H	-4.649575	-1.359375	5.223392
H	4.422658	2.566140	0.706153
H	1.064494	0.719598	2.665393
H	5.468107	3.003476	2.904153
H	2.109959	1.172703	4.878377
H	4.315249	2.314541	4.994718
H	3.614777	1.762121	-2.413661
H	0.844513	3.915108	0.082264
H	3.985423	3.812360	-3.761473
H	1.222210	5.957467	-1.255348
H	2.791201	5.915203	-3.186240
H	1.971895	-4.814402	-3.118712
H	3.765429	-5.610369	-1.592873
C	3.407182	-3.111471	2.598458
H	3.259852	-2.500533	3.488631
H	3.318344	-4.170899	2.862556
H	4.402857	-2.918547	2.184464

Table S244. XYZ Coordinates of H_meta_TS1_OMe

67

scf done: -4806.567404

C	3.026654	1.557657	1.545764
C	2.252926	2.005732	0.460301
C	1.783537	3.329013	0.462957
C	2.092832	4.188824	1.520010
C	2.866123	3.736657	2.590719

C	3.331312	2.418176	2.600619
P	1.742750	0.840147	-0.873014
C	3.354549	0.158642	-1.461203
C	3.347988	-1.109467	-2.065662
C	4.521625	-1.650339	-2.595334
C	5.715744	-0.929393	-2.526566
C	5.734184	0.333305	-1.927400
C	4.562250	0.874598	-1.396868
Pd	-0.100601	-0.578606	-0.403262
C	0.719355	-2.066552	0.875958
C	2.064636	-2.198622	1.280468
C	2.345967	-2.085042	2.633607
C	1.337798	-1.884315	3.596681
C	0.007110	-1.818860	3.183230
C	-0.318102	-1.969375	1.815373
Br	0.114457	-3.274718	-0.878994
O	-1.063176	-1.671091	4.013704
P	-2.081525	0.507666	-1.185372
O	-2.069929	0.744820	-2.837732
C	-2.391737	2.198111	-0.525253
C	-2.301354	3.332756	-1.343737
C	-2.469680	4.610601	-0.801802
C	-2.724257	4.767427	0.561416
C	-2.809475	3.640714	1.385974
C	-2.640678	2.365347	0.848138
C	-3.701709	-0.329633	-0.908438
C	-3.708479	-1.724238	-0.741112
C	-4.912595	-2.413546	-0.583206
C	-6.122636	-1.716174	-0.589471
C	-6.127411	-0.328354	-0.756468
C	-4.925152	0.362152	-0.914685
O	1.324273	1.889276	-2.106727
H	1.605841	-1.812549	4.643044
H	3.380383	-2.137507	2.963152
H	2.854180	-2.329656	0.550582
H	-1.360037	-2.064214	1.532439
H	-2.708815	1.497558	1.499570
H	-2.095978	3.220298	-2.403523
H	-3.007621	3.755837	2.447643
H	-2.401285	5.481238	-1.447683
H	-2.853658	5.760488	0.981440
H	3.395793	0.536868	1.566615
H	1.176669	3.688280	-0.362135
H	3.935704	2.060012	3.429080
H	1.727965	5.211832	1.504261
H	3.105454	4.405452	3.412214
H	-2.766833	-2.267137	-0.734022
H	-4.904800	-3.491638	-0.452164
H	-7.059387	-2.250768	-0.461793
H	-7.066884	0.216611	-0.759612

H	-4.940452	1.441221	-1.034862
H	-2.917945	1.074605	-3.176916
H	4.592127	1.853044	-0.927265
H	6.661476	0.896088	-1.871450
H	6.629888	-1.350089	-2.935200
H	4.503479	-2.633548	-3.056391
H	2.421964	-1.676820	-2.109724
H	2.044445	2.495463	-2.345487
C	-0.819107	-1.586325	5.416769
H	-1.797837	-1.481864	5.885668
H	-0.329923	-2.492576	5.791960
H	-0.203292	-0.713598	5.662540

Table S245. XYZ Coordinates of H_meta_IIa_OMe

67

scf done: -4806.641845

C	-1.400952	3.061619	-1.584219
C	-0.992070	1.735160	-1.779430
C	-1.765042	0.886543	-2.590127
C	-2.927489	1.360250	-3.196761
C	-3.330785	2.684458	-2.997004
C	-2.567006	3.531964	-2.192763
P	0.558124	1.071645	-1.070017
C	1.300462	2.441364	-0.114172
C	0.995045	2.592664	1.247737
C	1.556457	3.640956	1.977901
C	2.433900	4.535073	1.358365
C	2.747413	4.382565	0.005574
C	2.182614	3.340614	-0.732253
Pd	0.527792	-0.907018	0.093792
Br	0.918856	-3.162584	1.268118
P	-1.735195	-0.944846	1.007256
O	-1.722633	-1.830980	2.401431
C	-2.541328	0.570720	1.646154
C	-3.374962	1.363133	0.841656
C	-3.923516	2.544097	1.345774
C	-3.642372	2.949622	2.651958
C	-2.808361	2.169292	3.456777
C	-2.257733	0.987964	2.958284
C	-2.999890	-1.756061	-0.041900
C	-4.356431	-1.789815	0.324591
C	-5.278939	-2.482020	-0.459126
C	-4.858294	-3.148945	-1.614177
C	-3.511840	-3.127459	-1.982483
C	-2.585880	-2.436204	-1.197996
C	2.489060	-1.022124	-0.522607
C	2.832328	-1.678953	-1.722944
C	4.176312	-1.805294	-2.091972
C	5.199404	-1.287487	-1.294066
C	4.860922	-0.629898	-0.103447

C	3.511195	-0.495432	0.269540
O	5.765946	-0.078984	0.757276
O	1.445367	1.003043	-2.456158
H	6.231786	-1.397374	-1.602772
H	-4.692548	-1.272432	1.217766
H	2.123494	0.302422	-2.378798
H	-0.945711	-2.433654	2.356708
H	4.434421	-2.320037	-3.014131
H	2.062986	-2.117645	-2.353387
H	3.295229	0.031306	1.194889
H	-1.536669	-2.428385	-1.480092
H	-6.325456	-2.501186	-0.169921
H	-3.181352	-3.647839	-2.876376
H	-5.579677	-3.685333	-2.223510
H	-1.624106	0.377222	3.592514
H	-3.607441	1.059792	-0.173655
H	-2.589612	2.475983	4.475355
H	-4.571692	3.144665	0.714927
H	-4.072280	3.867554	3.041723
H	2.428127	3.218596	-1.781950
H	0.321101	1.896190	1.737873
H	3.432344	5.073565	-0.476597
H	1.314402	3.754432	3.030253
H	2.875566	5.345882	1.929932
H	-1.459229	-0.142943	-2.748708
H	-0.813625	3.729564	-0.963943
H	-3.517727	0.697402	-3.822068
H	-2.875330	4.561405	-2.037790
H	-4.237279	3.052655	-3.467987
C	7.150853	-0.171736	0.433091
H	7.683352	0.327256	1.243532
H	7.480185	-1.215628	0.372392
H	7.376514	0.333432	-0.513468

Table S246. XYZ Coordinates of H_meta_IIb_OMe

67

scf done: -4806.643018

C	-3.697746	1.372032	-0.742330
C	-2.969702	0.680271	-1.725096
C	-3.052555	1.095747	-3.063076
C	-3.859288	2.180697	-3.413228
C	-4.586531	2.859634	-2.433718
C	-4.504418	2.452685	-1.099131
P	-1.861222	-0.701742	-1.274570
O	-1.674472	-1.446974	-2.730156
Pd	0.240804	-0.223450	-0.305098
C	-0.712059	0.994779	1.013256
C	-0.696677	2.384542	0.794802
C	-1.341552	3.237136	1.691278
C	-2.014734	2.740912	2.813347

C	-2.029885	1.358247	3.031316
C	-1.380891	0.493055	2.130444
O	-2.649509	0.747546	4.087312
P	2.286996	0.327695	0.726661
O	2.101056	0.919376	2.259884
C	3.193043	1.654884	-0.155452
C	3.622738	1.450229	-1.478679
C	4.271300	2.473285	-2.169601
C	4.488726	3.710329	-1.554717
C	4.054449	3.921996	-0.245311
C	3.406441	2.900234	0.453518
C	3.496139	-1.029567	0.950611
C	3.007156	-2.309161	1.263455
C	3.890590	-3.353559	1.533628
C	5.269898	-3.132082	1.486091
C	5.763933	-1.864001	1.172532
C	4.882704	-0.813865	0.907388
Br	1.434382	-1.678084	-2.122033
C	-2.895065	-1.864282	-0.307748
C	-2.261228	-2.742367	0.586976
C	-3.002348	-3.697534	1.284165
C	-4.383629	-3.783469	1.094872
C	-5.021899	-2.916337	0.204177
C	-4.283305	-1.961389	-0.495742
H	-2.508500	3.424620	3.493264
H	-1.324046	4.310719	1.519071
H	-0.181637	2.803456	-0.064905
H	-1.426828	-0.571611	2.336930
H	2.946071	0.992527	2.732038
H	-0.718821	-1.666552	-2.832731
H	3.065889	3.073107	1.468898
H	3.444915	0.496142	-1.966127
H	4.217602	4.881702	0.235871
H	4.604979	2.305411	-3.189281
H	4.991660	4.505785	-2.096483
H	1.935797	-2.486016	1.284191
H	5.276126	0.168030	0.664821
H	3.504007	-4.339618	1.772817
H	6.835034	-1.690179	1.133383
H	5.957762	-3.947218	1.690228
H	-2.497503	0.561292	-3.826034
H	-3.636755	1.070124	0.298201
H	-3.921116	2.490423	-4.452281
H	-5.067743	2.977414	-0.333291
H	-5.215045	3.701809	-2.707821
H	-1.186933	-2.677143	0.737185
H	-4.788892	-1.291699	-1.183723
H	-2.502767	-4.369374	1.975618
H	-6.095272	-2.982747	0.053833
H	-4.961650	-4.523557	1.640290

C	-3.333675	1.566885	5.029969
H	-3.750193	0.887350	5.774600
H	-2.651136	2.269248	5.523320
H	-4.148140	2.129514	4.558099

Table S247. XYZ Coordinates of H_meta_III_OMe

67

scf done: -4806.613052

C	-3.857858	0.145260	2.034804
C	-3.915976	-0.541937	0.809427
C	-4.691485	-1.705007	0.706111
C	-5.415157	-2.164353	1.809485
C	-5.362940	-1.472682	3.020592
C	-4.582597	-0.317395	3.132204
P	-2.897242	0.045670	-0.588567
Pd	-0.566026	0.091128	-0.175509
P	1.724138	0.260399	0.275032
O	2.645083	-0.173396	-0.980113
C	-3.541249	1.703851	-1.009475
C	-4.818085	2.148980	-0.627724
C	-5.261503	3.414237	-1.013673
C	-4.439106	4.243443	-1.781183
C	-3.169147	3.807750	-2.167754
C	-2.719709	2.544764	-1.782348
O	-3.298965	-0.927616	-1.857414
C	-0.400924	-1.865915	-0.550814
C	0.121080	-2.271284	-1.785201
C	0.199711	-3.642045	-2.078544
C	-0.234226	-4.590258	-1.138217
C	-0.742776	-4.163930	0.083086
C	-0.833893	-2.798211	0.393320
O	0.687417	-4.147016	-3.248730
C	2.342250	-0.583266	1.771907
C	3.702270	-0.897724	1.927540
C	4.138655	-1.526351	3.094382
C	3.231099	-1.838262	4.110860
C	1.878175	-1.527035	3.959843
C	1.432068	-0.909826	2.789458
C	2.020299	2.049994	0.547450
C	1.863622	2.627714	1.818340
C	1.996978	4.007228	1.982416
C	2.284504	4.820728	0.883309
C	2.439546	4.252068	-0.383820
C	2.302609	2.874174	-0.555351
Br	5.683221	0.522688	-1.122188
H	-0.161296	-5.644482	-1.384864
H	0.469689	-1.535017	-2.497654
H	-1.241987	-2.486128	1.348376
H	-1.077879	-4.898992	0.810096
H	-4.216727	-0.812416	-2.153178

H	3.624102	0.087383	-0.961137
H	4.417007	-0.649262	1.146568
H	0.376420	-0.684896	2.663703
H	5.190252	-1.771374	3.210639
H	1.169249	-1.773586	4.744464
H	3.577621	-2.327101	5.016595
H	2.433786	2.435530	-1.539308
H	1.653233	2.003200	2.681274
H	2.670424	4.880047	-1.239044
H	1.883644	4.444521	2.969831
H	2.390973	5.893356	1.014298
H	-4.728880	-2.253157	-0.229462
H	-3.254883	1.044338	2.132421
H	-6.016828	-3.063689	1.719933
H	-4.538278	0.222508	4.073113
H	-5.924991	-1.832617	3.876996
H	-1.730991	2.208741	-2.084725
H	-5.462711	1.512880	-0.030263
H	-2.528582	4.450494	-2.763627
H	-6.249015	3.752264	-0.714845
H	-4.787372	5.228638	-2.076400
C	1.149650	-3.236292	-4.242674
H	1.486589	-3.850291	-5.078614
H	0.346766	-2.571371	-4.582803
H	1.988106	-2.632789	-3.875844

Table S248. XYZ Coordinates of H_meta_VI_OMe

92

scf done: -5687.152054

C	-2.460152	2.283054	2.082509
C	-3.343195	1.958876	1.038263
C	-4.713714	2.232871	1.185898
C	-5.188620	2.815834	2.361095
C	-4.304473	3.130957	3.395942
C	-2.940111	2.866103	3.255312
P	-2.644306	1.299795	-0.516860
O	-2.539806	2.648385	-1.504771
Pd	-0.499071	0.304406	-0.266781
C	-1.591859	-1.310848	0.451789
C	-2.044637	-1.349469	1.782783
C	-2.796527	-2.435567	2.235574
C	-3.109624	-3.506104	1.391101
C	-2.655313	-3.473659	0.067628
C	-1.904393	-2.377439	-0.392902
O	-2.891230	-4.453692	-0.856457
P	1.427595	-0.952973	0.259337
O	2.598698	0.088855	0.696022
C	1.356442	-2.131976	1.662190
C	1.062250	-3.490283	1.473826
C	0.975515	-4.348788	2.571121

C	1.181424	-3.860780	3.863584
C	1.476408	-2.509040	4.057607
C	1.561753	-1.647151	2.963718
C	2.046001	-1.949193	-1.148138
C	3.269478	-2.636617	-1.059012
C	3.729625	-3.383730	-2.143014
C	2.975163	-3.459524	-3.318095
C	1.762617	-2.774936	-3.414884
C	1.302981	-2.016513	-2.335885
P	0.766229	2.212720	-1.108995
C	2.368036	1.988408	-1.973534
C	3.588880	2.395249	-1.419325
C	4.777882	2.207627	-2.126634
C	4.758019	1.614709	-3.390777
C	3.543725	1.206269	-3.948707
C	2.354219	1.387261	-3.242761
C	-3.992061	0.361974	-1.326308
C	-4.209872	0.528486	-2.703882
C	-5.204356	-0.206838	-3.351694
C	-5.984904	-1.114678	-2.633734
C	-5.766424	-1.290358	-1.264820
C	-4.772308	-0.561965	-0.611636
O	-0.016725	3.030673	-2.305600
C	1.076059	3.484573	0.177737
C	1.497135	3.110120	1.464532
C	1.704089	4.084135	2.443456
C	1.479879	5.432238	2.152144
C	1.052745	5.807825	0.876046
C	0.852205	4.839825	-0.109820
Br	5.392946	-0.889134	1.745324
H	-3.693700	-4.337469	1.766953
H	-3.148983	-2.454446	3.264106
H	-1.820536	-0.538543	2.468529
H	-1.589103	-2.394045	-1.431815
H	3.473285	-0.301583	1.018023
H	-0.986847	3.003918	-2.151065
H	3.870077	-2.571599	-0.155312
H	0.367776	-1.470673	-2.418993
H	4.677813	-3.908152	-2.070662
H	1.177891	-2.824184	-4.328549
H	3.335762	-4.046838	-4.157465
H	1.806357	-0.601613	3.120469
H	0.904318	-3.881928	0.474683
H	1.645669	-2.125374	5.059367
H	0.752221	-5.399920	2.414211
H	1.116260	-4.531366	4.715339
H	0.517263	5.130375	-1.100166
H	1.680478	2.064596	1.690333
H	0.875750	6.854531	0.646594
H	2.037329	3.788769	3.434034

H	1.635457	6.186984	2.917381
H	-5.411369	1.988709	0.391669
H	-1.398709	2.078948	1.977374
H	-6.249096	3.022146	2.467758
H	-2.250131	3.110594	4.056979
H	-4.678370	3.581958	4.310171
H	-3.613103	1.236814	-3.268897
H	-4.605726	-0.716248	0.449046
H	-5.368790	-0.065286	-4.415505
H	-6.369228	-1.996678	-0.702231
H	-6.759820	-1.683979	-3.138152
H	3.615801	2.859888	-0.439956
H	1.415139	1.069328	-3.685120
H	5.718596	2.528744	-1.689438
H	3.521531	0.745583	-4.931828
H	5.684215	1.471669	-3.939504
H	-3.356348	3.168027	-1.584680
C	-3.646035	-5.592036	-0.452125
H	-3.712267	-6.237265	-1.329010
H	-3.149886	-6.135991	0.360516
H	-4.656806	-5.311698	-0.132370

Table S249. XYZ Coordinates of H_meta_V_OMe

91

scf done: -5686.698653

C	-0.780411	2.782683	-2.059880
C	-0.323537	3.340687	-0.854520
C	0.191132	4.645843	-0.852163
C	0.237754	5.386332	-2.035116
C	-0.228730	4.831197	-3.229423
C	-0.738583	3.530164	-3.239148
P	-0.343643	2.366764	0.706938
C	-2.034871	2.633632	1.388301
C	-3.140626	2.960172	0.591434
C	-4.398730	3.141597	1.169689
C	-4.567038	2.998412	2.548636
C	-3.469766	2.674089	3.350623
C	-2.212200	2.490613	2.774273
Pd	0.551150	0.110030	0.460462
P	-1.569503	-0.877575	0.010617
C	-1.657255	-2.427678	-0.974139
C	-1.637670	-2.341472	-2.375253
C	-1.662287	-3.499009	-3.154764
C	-1.699138	-4.755338	-2.543763
C	-1.716498	-4.848596	-1.150230
C	-1.696675	-3.691869	-0.368487
P	2.728346	0.986736	0.874118
C	4.069001	-0.152948	1.432660
C	4.353498	-0.216314	2.804742
C	5.356572	-1.062761	3.282928

C	6.087875	-1.854314	2.394457
C	5.814122	-1.792448	1.025143
C	4.811343	-0.947407	0.546720
C	3.431285	1.702671	-0.678185
C	3.240508	1.100235	-1.930569
C	3.791247	1.669250	-3.081563
C	4.526166	2.854271	-2.993283
C	4.707685	3.468988	-1.751141
C	4.162829	2.897174	-0.599741
O	2.714935	2.128122	1.950546
C	1.386893	-1.786623	0.334754
C	1.748731	-2.345434	-0.904062
C	2.301158	-3.632564	-0.980922
C	2.484110	-4.391157	0.184492
C	2.117618	-3.847286	1.411877
C	1.580515	-2.555179	1.492151
O	2.690975	-4.240336	-2.147053
O	-2.473449	0.160974	-0.868460
C	-2.513773	-1.283255	1.532675
C	-1.855077	-1.268150	2.771008
C	-2.543319	-1.590750	3.943171
C	-3.899700	-1.915483	3.886790
C	-4.567054	-1.917146	2.657684
C	-3.880720	-1.607382	1.483318
O	0.524623	3.244556	1.754948
Br	-5.348393	-0.569948	-1.991894
H	2.906400	-5.388276	0.104810
H	1.579361	-1.785222	-1.816437
H	1.325206	-2.154355	2.469441
H	2.255856	-4.432775	2.318249
H	-3.373968	-0.156130	-1.181776
H	1.479051	2.805761	1.886773
H	-4.411672	-1.603506	0.534027
H	-0.803596	-0.999537	2.814878
H	-5.624499	-2.161559	2.613906
H	-2.022585	-1.580203	4.896209
H	-4.438149	-2.161861	4.797378
H	-1.621959	-1.369067	-2.857256
H	-1.718638	-3.777897	0.713028
H	-1.659372	-3.418855	-4.237977
H	-1.752256	-5.821851	-0.669422
H	-1.719145	-5.655849	-3.150542
H	0.558818	5.071146	0.076079
H	-1.179776	1.773124	-2.070064
H	0.639053	6.395809	-2.023903
H	-1.100797	3.094342	-4.166000
H	-0.191650	5.408190	-4.148954
H	4.292180	3.375475	0.366469
H	2.659253	0.186473	-2.005938
H	5.271873	4.394796	-1.679864

H	3.640849	1.191546	-4.045630
H	4.949474	3.300172	-3.888744
H	3.797309	0.413008	3.492152
H	4.611957	-0.908357	-0.519056
H	5.569357	-1.099233	4.347810
H	6.384708	-2.399476	0.327765
H	6.869641	-2.511337	2.764874
H	-3.024058	3.076303	-0.480161
H	-1.362763	2.248383	3.405402
H	-5.246385	3.398252	0.540906
H	-3.591352	2.565020	4.424522
H	-5.546309	3.141372	2.996248
C	2.512132	-3.535115	-3.369900
H	2.887397	-4.193087	-4.155413
H	1.454316	-3.315543	-3.557401
H	3.081978	-2.597759	-3.383900

Table S250. XYZ Coordinates of H_meta_TS2_OMe

91

scf done: -5686.659765

C	-2.485410	1.548944	2.635124
C	-1.219634	2.147263	2.754121
C	-0.810604	2.641163	4.001450
C	-1.655482	2.543869	5.110412
C	-2.915735	1.955312	4.984772
C	-3.328408	1.459657	3.744318
P	-0.106648	2.250952	1.284569
C	-0.881943	3.617830	0.315385
C	-0.410330	4.933933	0.428958
C	-1.002238	5.960536	-0.310276
C	-2.070649	5.683157	-1.167006
C	-2.541843	4.372903	-1.287016
C	-1.947990	3.341398	-0.555867
Pd	0.447080	0.171171	0.276400
P	-1.549475	-1.055107	-0.346009
O	-2.816752	-0.019786	-0.556540
P	2.784399	0.667510	-0.127134
C	4.393723	-0.082974	-0.683981
C	5.375709	-0.311148	0.292926
C	6.609312	-0.862255	-0.055533
C	6.878394	-1.196566	-1.385648
C	5.908983	-0.969409	-2.364233
C	4.674343	-0.414428	-2.016824
C	2.287701	1.593750	-1.650366
C	1.594493	1.008318	-2.724091
C	1.247100	1.775522	-3.838282
C	1.579651	3.131731	-3.890434
C	2.262594	3.720354	-2.822990
C	2.614751	2.957925	-1.707484
O	3.207037	1.611683	1.024008

C	2.052753	-1.293132	0.407321
C	2.349087	-1.510338	1.769636
C	2.644482	-2.802214	2.229203
C	2.670714	-3.875023	1.325459
C	2.405413	-3.650178	-0.024515
C	2.109518	-2.368862	-0.494889
O	2.928483	-3.111126	3.527399
C	-1.458312	-1.929691	-1.969308
C	-1.681352	-1.206025	-3.153345
C	-1.518420	-1.814129	-4.399232
C	-1.119392	-3.151060	-4.482184
C	-0.889390	-3.877186	-3.311263
C	-1.056684	-3.272035	-2.063458
C	-2.178915	-2.372670	0.781359
C	-1.323507	-2.865921	1.778476
C	-1.756785	-3.864697	2.655583
C	-3.054701	-4.367336	2.553325
C	-3.917762	-3.872054	1.570110
C	-3.485165	-2.883767	0.685353
O	1.214249	2.980492	1.928149
Br	-5.626793	-0.823869	-1.877663
H	2.913656	-4.865891	1.695584
H	2.426010	-4.486857	-0.717696
H	1.907727	-2.219812	-1.549966
H	2.360482	-0.666971	2.449786
H	-3.647909	-0.382263	-0.976605
H	2.035690	2.472972	1.614133
H	-4.168078	-2.506136	-0.072596
H	-0.317901	-2.465720	1.868203
H	-4.930243	-4.258439	1.491900
H	-1.083103	-4.241502	3.419935
H	-3.395987	-5.138532	3.238058
H	-2.001992	-0.170349	-3.098365
H	-0.883091	-3.851588	-1.162074
H	-1.708190	-1.244731	-5.304811
H	-0.586263	-4.918972	-3.367008
H	-0.992992	-3.623944	-5.451741
H	0.422395	5.145500	1.091889
H	-2.310926	2.322376	-0.665272
H	-0.628770	6.976697	-0.216901
H	-3.368128	4.151252	-1.956925
H	-2.529646	6.482346	-1.742086
H	3.139402	3.417242	-0.875460
H	1.316989	-0.040777	-2.696444
H	2.523145	4.774337	-2.857791
H	0.713804	1.311523	-4.662837
H	1.307057	3.726135	-4.757436
H	5.173683	-0.044258	1.324947
H	3.940135	-0.233368	-2.794796
H	7.361181	-1.027826	0.711034

H	6.112781	-1.217481	-3.402070
H	7.838438	-1.625845	-1.657361
H	-2.811490	1.147054	1.679438
H	0.167890	3.099719	4.096635
H	-4.306199	0.997399	3.640825
H	-1.328034	2.930107	6.071824
H	-3.571311	1.879833	5.847589
C	2.931910	-2.059432	4.490067
H	3.175495	-2.527602	5.444325
H	3.688885	-1.302141	4.254741
H	1.949310	-1.578574	4.562899

Table S251. XYZ Coordinates of H_meta_VI_OMe

91

scf done: -5686.690250

C	2.020155	-1.187583	2.738017
C	2.948683	-0.987770	1.705533
C	4.322482	-1.048994	1.998883
C	4.750205	-1.313197	3.300150
C	3.817013	-1.527470	4.320263
C	2.451660	-1.464877	4.038345
P	2.307226	-0.643076	0.011463
C	2.970409	-2.065780	-0.955935
C	3.403197	-1.880944	-2.278855
C	3.784725	-2.974124	-3.059805
C	3.729249	-4.267553	-2.535211
C	3.293312	-4.462436	-1.221339
C	2.915371	-3.370794	-0.437888
Pd	0.049756	0.002847	-0.195037
C	-1.123055	-1.946937	-0.565137
C	-2.070793	-0.876728	-0.683983
C	-2.501692	-0.492332	-1.994997
C	-2.008980	-1.116849	-3.125098
C	-1.094394	-2.200677	-3.001405
C	-0.680812	-2.603099	-1.748835
P	-3.204922	-0.422207	0.673822
C	-4.751373	-1.351805	0.340445
C	-5.959008	-0.797868	0.793136
C	-7.164744	-1.469862	0.586994
C	-7.175229	-2.701239	-0.073362
C	-5.978468	-3.256583	-0.532783
C	-4.771105	-2.584407	-0.330943
O	-2.467795	-0.645603	-4.326638
P	0.142827	2.360592	-0.277439
O	1.668325	2.840948	-0.712413
C	-0.838201	3.296747	-1.537382
C	-2.241149	3.206208	-1.540296
C	-2.988105	3.896989	-2.496855
C	-2.348860	4.670554	-3.469993
C	-0.954704	4.751853	-3.480729

C	-0.202151	4.070304	-2.520423
C	-0.118979	3.390750	1.240410
C	-1.355783	3.374499	1.909053
C	-1.538717	4.134218	3.066578
C	-0.492432	4.907007	3.579244
C	0.741162	4.917651	2.925350
C	0.927888	4.165215	1.762191
O	3.231530	0.623771	-0.547515
O	-3.495832	1.061843	0.792619
C	-2.546729	-1.117109	2.236140
C	-1.956070	-0.232939	3.151945
C	-1.473294	-0.703136	4.375365
C	-1.574230	-2.059680	4.693827
C	-2.166379	-2.945538	3.789373
C	-2.657386	-2.476764	2.569490
Br	6.385048	0.594968	-0.906289
H	-0.732481	-2.722163	-3.878876
H	-1.203592	-2.424093	5.647469
H	-2.258249	-3.998589	4.038105
H	-3.140138	-3.171274	1.889032
H	-1.889052	0.822201	2.910482
H	-1.024980	-0.009285	5.080336
H	4.227977	0.518205	-0.613192
H	2.286453	2.074290	-0.682964
H	-5.948897	0.165120	1.294062
H	-3.848835	-3.018981	-0.704872
H	-8.094399	-1.031609	0.938087
H	-5.984361	-4.209347	-1.053968
H	-8.113604	-3.223818	-0.234517
H	-0.962882	-2.436448	0.389337
H	-3.228957	0.303842	-2.117458
H	0.000073	-3.443814	-1.655299
H	0.880215	4.137372	-2.527049
H	-2.749017	2.588678	-0.803215
H	-0.449484	5.348575	-4.235501
H	-4.072433	3.824102	-2.486732
H	-2.932841	5.201131	-4.216769
H	3.461669	-0.878439	-2.691050
H	2.586505	-3.536657	0.584387
H	4.128605	-2.813252	-4.077729
H	3.253752	-5.464363	-0.803219
H	4.025583	-5.117044	-3.143585
H	5.058050	-0.875506	1.216935
H	0.957057	-1.117713	2.522617
H	5.813468	-1.353641	3.519707
H	1.721171	-1.622094	4.826414
H	4.155554	-1.736343	5.331127
H	-2.170867	2.759905	1.534865
H	1.883600	4.180606	1.249285
H	-2.500155	4.117605	3.573061

H	1.560672	5.514303	3.316958
H	-0.637246	5.492350	4.482869
C	-2.037831	-1.288975	-5.522209
H	-2.534232	-0.763789	-6.339187
H	-0.952067	-1.214778	-5.655995
H	-2.333000	-2.345059	-5.541881

Table S252. XYZ Coordinates of H_meta_VII_OMe

91

scf done: -5686.693689

C	2.090895	2.382739	1.385759
C	1.271303	3.508774	1.576813
C	1.572352	4.413968	2.604597
C	2.684487	4.202955	3.424786
C	3.502085	3.088819	3.225154
C	3.202987	2.180600	2.204585
P	-0.195083	3.728374	0.488711
O	-0.903963	5.092386	1.157651
Pd	-1.630123	1.956817	0.277569
P	-3.239545	0.290092	0.184164
O	-4.714430	0.820759	0.680192
C	-2.917723	-1.174028	1.261927
C	-2.124922	-2.244636	0.817852
C	-1.804804	-3.295275	1.680758
C	-2.269464	-3.289122	2.998856
C	-3.058451	-2.227714	3.449060
C	-3.377749	-1.175352	2.588577
C	-3.500856	-0.480090	-1.470030
C	-4.553321	-1.375811	-1.727347
C	-4.706521	-1.930224	-2.998429
C	-3.810902	-1.605619	-4.023203
C	-2.763551	-0.715544	-3.776773
C	-2.614581	-0.150473	-2.506619
C	0.529808	4.461258	-1.034410
C	-0.084252	4.179319	-2.264886
C	0.399858	4.745897	-3.446080
C	1.504306	5.599610	-3.407581
C	2.124064	5.885308	-2.187051
C	1.642445	5.317900	-1.006966
C	2.595038	-2.476185	3.164104
C	2.174681	-2.248238	1.857442
C	3.127239	-1.930881	0.869825
C	4.478237	-1.844693	1.210408
C	4.894313	-2.078335	2.532206
C	3.947556	-2.395016	3.513675
P	2.533392	-1.524547	-0.810235
C	3.999775	-1.427634	-1.898446
C	4.392613	-0.157727	-2.346244
C	5.502688	-0.018998	-3.181763
C	6.226044	-1.146745	-3.575351

C	5.837345	-2.415743	-3.135915
C	4.727688	-2.558155	-2.302467
O	6.234272	-1.975055	2.750997
C	1.561678	-2.970889	-1.358714
C	0.487379	-2.740535	-2.232056
C	-0.279217	-3.808115	-2.702074
C	0.024046	-5.113751	-2.306849
C	1.090757	-5.350445	-1.435919
C	1.855871	-4.283938	-0.958988
O	1.724511	-0.242441	-0.860934
Br	-7.224642	-1.127312	1.120197
H	4.246435	-2.579336	4.538373
H	-0.573003	-5.944400	-2.671441
H	1.323079	-6.363290	-1.120867
H	2.668617	-4.477664	-0.265585
H	0.245031	-1.724476	-2.526400
H	-1.114845	-3.617545	-3.368759
H	-5.432800	0.132460	0.781351
H	-1.768067	4.840108	1.513748
H	3.819694	0.712585	-2.043114
H	4.428654	-3.550050	-1.977840
H	5.799483	0.967290	-3.525815
H	6.394981	-3.294411	-3.445839
H	7.088503	-1.039308	-4.226582
H	1.120835	-2.320028	1.607509
H	5.234152	-1.602902	0.471419
H	1.865584	-2.724020	3.929475
H	0.933757	5.277250	2.760812
H	1.862654	1.658379	0.605768
H	2.909669	4.909289	4.218993
H	3.830440	1.306884	2.051739
H	4.364437	2.924658	3.865031
H	-4.001441	-0.360315	2.942451
H	-1.761454	-2.267738	-0.205000
H	-3.429777	-2.219295	4.469933
H	-1.198768	-4.121061	1.318839
H	-2.022166	-4.107711	3.668489
H	-5.261551	-1.627821	-0.941158
H	-1.807992	0.552566	-2.310116
H	-5.525719	-2.617235	-3.191331
H	-2.070152	-0.453871	-4.570905
H	-3.934822	-2.040193	-5.011066
H	-0.942356	3.511956	-2.288575
H	2.135306	5.538578	-0.064859
H	-0.082158	4.518864	-4.392555
H	2.984753	6.547195	-2.155790
H	1.884500	6.038959	-4.325321
C	6.722833	-2.197670	4.072873
H	7.803454	-2.063611	4.018660
H	6.302410	-1.475274	4.781914

H	6.499838	-3.214528	4.415925
